# 31st Annual Meeting and Associated Programs of the Society for Immunotherapy of Cancer (SITC 2016): part two: National Harbor, MD, USA. 9-13 November 2016

**DOI:** 10.1186/s40425-016-0173-6

**Published:** 2016-11-16

**Authors:** Casey Ager, Matthew Reilley, Courtney Nicholas, Todd Bartkowiak, Ashvin Jaiswal, Michael Curran, Tina C. Albershardt, Anshika Bajaj, Jacob F. Archer, Rebecca S. Reeves, Lisa Y. Ngo, Peter Berglund, Jan ter Meulen, Caroline Denis, Hormas Ghadially, Thomas Arnoux, Fabien Chanuc, Nicolas Fuseri, Robert W. Wilkinson, Nicolai Wagtmann, Yannis Morel, Pascale Andre, Michael B. Atkins, Matteo S. Carlino, Antoni Ribas, John A. Thompson, Toni K. Choueiri, F. Stephen Hodi, Wen-Jen Hwu, David F. McDermott, Victoria Atkinson, Jonathan S. Cebon, Bernie Fitzharris, Michael B. Jameson, Catriona McNeil, Andrew G. Hill, Eric Mangin, Malidi Ahamadi, Marianne van Vugt, Mariëlle van Zutphen, Nageatte Ibrahim, Georgina V. Long, Robyn Gartrell, Zoe Blake, Ines Simoes, Yichun Fu, Takuro Saito, Yingzhi Qian, Yan Lu, Yvonne M. Saenger, Sadna Budhu, Olivier De Henau, Roberta Zappasodi, Kyle Schlunegger, Bruce Freimark, Jeff Hutchins, Christopher A. Barker, Jedd D. Wolchok, Taha Merghoub, Elena Burova, Omaira Allbritton, Peter Hong, Jie Dai, Jerry Pei, Matt Liu, Joel Kantrowitz, Venus Lai, William Poueymirou, Douglas MacDonald, Ella Ioffe, Markus Mohrs, William Olson, Gavin Thurston, Cristian Capasso, Federica Frascaro, Sara Carpi, Siri Tähtinen, Sara Feola, Manlio Fusciello, Karita Peltonen, Beatriz Martins, Madeleine Sjöberg, Sari Pesonen, Tuuli Ranki, Lukasz Kyruk, Erkko Ylösmäki, Vincenzo Cerullo, Fabio Cerignoli, Biao Xi, Garret Guenther, Naichen Yu, Lincoln Muir, Leyna Zhao, Yama Abassi, Víctor Cervera-Carrascón, Mikko Siurala, João Santos, Riikka Havunen, Suvi Parviainen, Akseli Hemminki, Angus Dalgleish, Satvinder Mudan, Mark DeBenedette, Ana Plachco, Alicia Gamble, Elizabeth W. Grogan, John Krisko, Irina Tcherepanova, Charles Nicolette, Pooja Dhupkar, Ling Yu, Eugenie S. Kleinerman, Nancy Gordon, Italia Grenga, Lauren Lepone, Sofia Gameiro, Karin M. Knudson, Massimo Fantini, Kwong Tsang, James Hodge, Renee Donahue, Jeffrey Schlom, Elizabeth Evans, Holm Bussler, Crystal Mallow, Christine Reilly, Sebold Torno, Maria Scrivens, Cathie Foster, Alan Howell, Leslie Balch, Alyssa Knapp, John E. Leonard, Mark Paris, Terry Fisher, Siwen Hu-Lieskovan, Antoni Ribas, Ernest Smith, Maurice Zauderer, William Fogler, Marilyn Franklin, Matt Thayer, Dan Saims, John L. Magnani, Jian Gong, Michael Gray, Jeff Hutchins, Bruce Freimark, George Fromm, Suresh de Silva, Louise Giffin, Xin Xu, Jason Rose, Taylor H. Schreiber, Massimo Fantini, Sofia R. Gameiro, Karin M. Knudson, Paul E. Clavijo, Clint T. Allen, Renee Donahue, Lauren Lepone, Italia Grenga, James W. Hodge, Kwong Y. Tsang, Jeffrey Schlom, Michael Gray, Jian Gong, Jeff Hutchins, Bruce Freimark, Jane Grogan, Nicholas Manieri, Eugene Chiang, Patrick Caplazi, Mahesh Yadav, Patrick Hagner, Hsiling Chiu, Michelle Waldman, Anke Klippel, Anjan Thakurta, Michael Pourdehnad, Anita Gandhi, Ian Henrich, Laura Quick, Rob Young, Margaret Chou, Andrew Hotson, Stephen Willingham, Po Ho, Carmen Choy, Ginna Laport, Ian McCaffery, Richard Miller, Kimberly A. Tipton, Kenneth R. Wong, Victoria Singson, Chihunt Wong, Chanty Chan, Yuanhiu Huang, Shouchun Liu, Jennifer H. Richardson, W. Michael Kavanaugh, James West, Bryan A. Irving, Kimberly A. Tipton, Kenneth R. Wong, Victoria Singson, Chihunt Wong, Chanty Chan, Yuanhiu Huang, Shouchun Liu, Jennifer H. Richardson, W. Michael Kavanaugh, James West, Bryan A. Irving, Ritika Jaini, Matthew Loya, Charis Eng, Melissa L. Johnson, Alex A. Adjei, Mateusz Opyrchal, Suresh Ramalingam, Pasi A. Janne, George Dominguez, Dmitry Gabrilovich, Laura de Leon, Jeannette Hasapidis, Scott J. Diede, Peter Ordentlich, Scott Cruickshank, Michael L. Meyers, Matthew D. Hellmann, Pawel Kalinski, Amer Zureikat, Robert Edwards, Ravi Muthuswamy, Nataša Obermajer, Julie Urban, Lisa H. Butterfield, William Gooding, Herbert Zeh, David Bartlett, Olga Zubkova, Larissa Agapova, Marina Kapralova, Liudmila Krasovskaia, Armen Ovsepyan, Maxim Lykov, Artem Eremeev, Vladimir Bokovanov, Olga Grigoryeva, Andrey Karpov, Sergey Ruchko, Charles Nicolette, Alexandr Shuster, Danny N. Khalil, Luis Felipe Campesato, Yanyun Li, Taha Merghoub, Jedd D. Wolchok, Adam S. Lazorchak, Troy D. Patterson, Yueyun Ding, Pottayil Sasikumar, Naremaddepalli Sudarshan, Nagaraj Gowda, Raghuveer Ramachandra, Dodheri Samiulla, Sanjeev Giri, Rajesh Eswarappa, Murali Ramachandra, David Tuck, Timothy Wyant, Jasmin Leshem, Xiu-fen Liu, Tapan Bera, Masaki Terabe, Birgit Bossenmaier, Gerhard Niederfellner, Yoram Reiter, Ira Pastan, Leiming Xia, Yang Xia, Yangyang Hu, Yi Wang, Yangyi Bao, Fu Dai, Shiang Huang, Elaine Hurt, Robert E. Hollingsworth, Lawrence G. Lum, Alfred E. Chang, Max S. Wicha, Qiao Li, Thomas Mace, Neil Makhijani, Erin Talbert, Gregory Young, Denis Guttridge, Darwin Conwell, Gregory B. Lesinski, Rodney JM Macedo Gonzales, Austin P. Huffman, Ximi K. Wang, Ran Reshef, Andy MacKinnon, Jason Chen, Matt Gross, Gisele Marguier, Peter Shwonek, Natalija Sotirovska, Susanne Steggerda, Francesco Parlati, Amani Makkouk, Mark K. Bennett, Jason Chen, Ethan Emberley, Matt Gross, Tony Huang, Weiqun Li, Andy MacKinnon, Gisele Marguier, Silinda Neou, Alison Pan, Jing Zhang, Winter Zhang, Francesco Parlati, Netonia Marshall, Thomas U. Marron, Judith Agudo, Brian Brown, Joshua Brody, Christopher McQuinn, Thomas Mace, Matthew Farren, Hannah Komar, Reena Shakya, Gregory Young, Thomas Ludwug, Gregory B. Lesinski, Y. Maurice Morillon, Scott A. Hammond, Jeffrey Schlom, John W. Greiner, Pulak R. Nath, Anthony L. Schwartz, Dragan Maric, David D. Roberts, Nataša Obermajer, David Bartlett, Pawel Kalinski, Aung Naing, Kyriakos P. Papadopoulos, Karen A. Autio, Deborah J. Wong, Manish Patel, Gerald Falchook, Shubham Pant, Patrick A. Ott, Melinda Whiteside, Amita Patnaik, John Mumm, Filip Janku, Ivan Chan, Todd Bauer, Rivka Colen, Peter VanVlasselaer, Gail L. Brown, Nizar M. Tannir, Martin Oft, Jeffrey Infante, Evan Lipson, Ajay Gopal, Sattva S. Neelapu, Philippe Armand, Stephen Spurgeon, John P. Leonard, F. Stephen Hodi, Rachel E. Sanborn, Ignacio Melero, Thomas F. Gajewski, Matthew Maurer, Serena Perna, Andres A. Gutierrez, Raphael Clynes, Priyam Mitra, Satyendra Suryawanshi, Douglas Gladstone, Margaret K. Callahan, James Crooks, Sheila Brown, Audrey Gauthier, Marc Hillairet de Boisferon, Andrew MacDonald, Laura Rosa Brunet, William T. Rothwell, Peter Bell, James M. Wilson, Fumi Sato-Kaneko, Shiyin Yao, Shannon S. Zhang, Dennis A. Carson, Cristina Guiducci, Robert L. Coffman, Kazutaka Kitaura, Takaji Matsutani, Ryuji Suzuki, Tomoko Hayashi, Ezra E. W. Cohen, David Schaer, Yanxia Li, Julie Dobkin, Michael Amatulli, Gerald Hall, Thompson Doman, Jason Manro, Frank Charles Dorsey, Lillian Sams, Rikke Holmgaard, Krishnadatt Persaud, Dale Ludwig, David Surguladze, John S. Kauh, Ruslan Novosiadly, Michael Kalos, Kyla Driscoll, Hardev Pandha, Christy Ralph, Kevin Harrington, Brendan Curti, Rachel E. Sanborn, Wallace Akerley, Sumati Gupta, Alan Melcher, David Mansfield, David R. Kaufman, Emmett Schmidt, Mark Grose, Bronwyn Davies, Roberta Karpathy, Darren Shafren, Katerina Shamalov, Cyrille Cohen, Naveen Sharma, James Allison, Tala Shekarian, Sandrine Valsesia-Wittmann, Christophe Caux, Aurelien Marabelle, Brian M. Slomovitz, Kathleen M. Moore, Hagop Youssoufian, Marshall Posner, Poonam Tewary, Alan D. Brooks, Ya-Ming Xu, Kithsiri Wijeratne, Leslie A. A. Gunatilaka, Thomas J. Sayers, John P. Vasilakos, Tesha Alston, Simon Dovedi, James Elvecrog, Iwen Grigsby, Ronald Herbst, Karen Johnson, Craig Moeckly, Stefanie Mullins, Kristen Siebenaler, Julius SternJohn, Ashenafi Tilahun, Mark A. Tomai, Katharina Vogel, Robert W. Wilkinson, Eveline E. Vietsch, Anton Wellstein, Martin Wythes, Stefano Crosignani, Joseph Tumang, Shilpa Alekar, Patrick Bingham, Sandra Cauwenberghs, Jenny Chaplin, Deepak Dalvie, Sofie Denies, Coraline De Maeseneire, JunLi Feng, Kim Frederix, Samantha Greasley, Jie Guo, James Hardwick, Stephen Kaiser, Katti Jessen, Erick Kindt, Marie-Claire Letellier, Wenlin Li, Karen Maegley, Reece Marillier, Nichol Miller, Brion Murray, Romain Pirson, Julie Preillon, Virginie Rabolli, Chad Ray, Kevin Ryan, Stephanie Scales, Jay Srirangam, Jim Solowiej, Al Stewart, Nicole Streiner, Vince Torti, Konstantinos Tsaparikos, Xianxian Zheng, Gregory Driessens, Bruno Gomes, Manfred Kraus, Chunxiao Xu, Yanping Zhang, Giorgio Kradjian, Guozhong Qin, Jin Qi, Xiaomei Xu, Bo Marelli, Huakui Yu, Wilson Guzman, Rober Tighe, Rachel Salazar, Kin-Ming Lo, Jessie English, Laszlo Radvanyi, Yan Lan, Roberta Zappasodi, Sadna Budhu, Matthew D. Hellmann, Michael Postow, Yasin Senbabaoglu, Billel Gasmi, Hong Zhong, Yanyun Li, Cailian Liu, Daniel Hirschhorhn-Cymerman, Jedd D. Wolchok, Taha Merghoub, Yuanyuan Zha, Gregory Malnassy, Noreen Fulton, Jae-Hyun Park, Wendy Stock, Yusuke Nakamura, Thomas F. Gajewski, Hongtao Liu, Xiaoming Ju, Rachelle Kosoff, Kimberly Ramos, Brandon Coder, Robert Petit, Michael Princiotta, Kyle Perry, Jun Zou, Ainhoa Arina, Christian Fernandez, Wenxin Zheng, Michael A. Beckett, Helena J. Mauceri, Yang-Xin Fu, Ralph R. Weichselbaum, Mark DeBenedette, Whitney Lewis, Alicia Gamble, Charles Nicolette, Yanyan Han, Yeting Wu, Chou Yang, Jing Huang, Dongyun Wu, Jin Li, Xiaoling Liang, Xiangjun Zhou, Jinlin Hou, Raffit Hassan, Thierry Jahan, Scott J. Antonia, Hedy L. Kindler, Evan W. Alley, Somayeh Honarmand, Weiqun Liu, Meredith L. Leong, Chan C. Whiting, Nitya Nair, Amanda Enstrom, Edward E. Lemmens, Takahiro Tsujikawa, Sushil Kumar, Lisa M. Coussens, Aimee L. Murphy, Dirk G. Brockstedt, Sven D. Koch, Martin Sebastian, Christian Weiss, Martin Früh, Miklos Pless, Richard Cathomas, Wolfgang Hilbe, Georg Pall, Thomas Wehler, Jürgen Alt, Helge Bischoff, Michael Geissler, Frank Griesinger, Jens Kollmeier, Alexandros Papachristofilou, Fatma Doener, Mariola Fotin-Mleczek, Madeleine Hipp, Henoch S. Hong, Karl-Josef Kallen, Ute Klinkhardt, Claudia Stosnach, Birgit Scheel, Andreas Schroeder, Tobias Seibel, Ulrike Gnad-Vogt, Alfred Zippelius, Ha-Ram Park, Yong-Oon Ahn, Tae Min Kim, Soyeon Kim, Seulki Kim, Yu Soo Lee, Bhumsuk Keam, Dong-Wan Kim, Dae Seog Heo, Shari Pilon-Thomas, Amy Weber, Jennifer Morse, Krithika Kodumudi, Hao Liu, John Mullinax, Amod A. Sarnaik, Luke Pike, Andrew Bang, Patrick A. Ott, Tracy Balboni, Allison Taylor, Alexander Spektor, Tyler Wilhite, Monica Krishnan, Daniel Cagney, Brian Alexander, Ayal Aizer, Elizabeth Buchbinder, Mark Awad, Leena Ghandi, F. Stephen Hodi, Jonathan Schoenfeld, Anthony L. Schwartz, Pulak R. Nath, Elizabeth Lessey-Morillon, Lisa Ridnour, David D. Roberts, Neil H. Segal, Manish Sharma, Dung T. Le, Patrick A. Ott, Robert L. Ferris, Andrew D. Zelenetz, Sattva S. Neelapu, Ronald Levy, Izidore S. Lossos, Caron Jacobson, Radhakrishnan Ramchandren, John Godwin, A. Dimitrios Colevas, Roland Meier, Suba Krishnan, Xuemin Gu, Jaclyn Neely, Satyendra Suryawanshi, John Timmerman, Claire I. Vanpouille-Box, Silvia C. Formenti, Sandra Demaria, Erik Wennerberg, Aranzazu Mediero, Bruce N. Cronstein, Silvia C. Formenti, Sandra Demaria, Michael P. Gustafson, AriCeli DiCostanzo, Courtney Wheatley, Chul-Ho Kim, Svetlana Bornschlegl, Dennis A. Gastineau, Bruce D. Johnson, Allan B. Dietz, Cameron MacDonald, Mark Bucsek, Guanxi Qiao, Bonnie Hylander, Elizabeth Repasky, William J. Turbitt, Yitong Xu, Andrea Mastro, Connie J. Rogers, Sita Withers, Ziming Wang, Lam T. Khuat, Cordelia Dunai, Bruce R. Blazar, Dan Longo, Robert Rebhun, Steven K. Grossenbacher, Arta Monjazeb, William J. Murphy, Scott Rowlinson, Giulia Agnello, Susan Alters, David Lowe, Nicole Scharping, Ashley V. Menk, Ryan Whetstone, Xue Zeng, Greg M. Delgoffe, Patricia M. Santos, Ashley V. Menk, Jian Shi, Greg M. Delgoffe, Lisa H. Butterfield, Ryan Whetstone, Ashley V. Menk, Nicole Scharping, Greg Delgoffe, Misako Nagasaka, Ammar Sukari, Miranda Byrne-Steele, Wenjing Pan, Xiaohong Hou, Brittany Brown, Mary Eisenhower, Jian Han, Natalie Collins, Robert Manguso, Hans Pope, Yashaswi Shrestha, Jesse Boehm, W. Nicholas Haining, Kyle R. Cron, Ayelet Sivan, Keston Aquino-Michaels, Thomas F. Gajewski, Marco Orecchioni, Davide Bedognetti, Wouter Hendrickx, Claudia Fuoco, Filomena Spada, Francesco Sgarrella, Gianni Cesareni, Francesco Marincola, Kostas Kostarelos, Alberto Bianco, Lucia Delogu, Wouter Hendrickx, Jessica Roelands, Sabri Boughorbel, Julie Decock, Scott Presnell, Ena Wang, Franco M. Marincola, Peter Kuppen, Michele Ceccarelli, Darawan Rinchai, Damien Chaussabel, Lance Miller, Davide Bedognetti, Andrew Nguyen, J. Zachary Sanborn, Charles Vaske, Shahrooz Rabizadeh, Kayvan Niazi, Steven Benz, Shashank Patel, Nicholas Restifo, James White, Sam Angiuoli, Mark Sausen, Sian Jones, Maria Sevdali, John Simmons, Victor Velculescu, Luis Diaz, Theresa Zhang, Jennifer S. Sims, Sunjay M. Barton, Robyn Gartrell, Angela Kadenhe-Chiweshe, Filemon Dela Cruz, Andrew T. Turk, Yan Lu, Christopher F. Mazzeo, Andrew L. Kung, Jeffrey N. Bruce, Yvonne M. Saenger, Darrell J. Yamashiro, Eileen P. Connolly, Jason Baird, Marka Crittenden, David Friedman, Hong Xiao, Rom Leidner, Bryan Bell, Kristina Young, Michael Gough, Zhen Bian, Koby Kidder, Yuan Liu, Emily Curran, Xiufen Chen, Leticia P. Corrales, Justin Kline, Cordelia Dunai, Ethan G. Aguilar, Lam T. Khuat, William J. Murphy, Jennifer Guerriero, Alaba Sotayo, Holly Ponichtera, Alexandra Pourzia, Sara Schad, Ruben Carrasco, Suzan Lazo, Roderick Bronson, Anthony Letai, Richard S. Kornbluth, Sachin Gupta, James Termini, Elizabeth Guirado, Geoffrey W. Stone, Christina Meyer, Laura Helming, Joseph Tumang, Nicholas Wilson, Robert Hofmeister, Laszlo Radvanyi, Natalie J. Neubert, Laure Tillé, David Barras, Charlotte Soneson, Petra Baumgaertner, Donata Rimoldi, David Gfeller, Mauro Delorenzi, Silvia A. Fuertes Marraco, Daniel E. Speiser, Tara S. Abraham, Bo Xiang, Michael S. Magee, Scott A. Waldman, Adam E. Snook, Wojciech Blogowski, Ewa Zuba-Surma, Marta Budkowska, Daria Salata, Barbara Dolegowska, Teresa Starzynska, Leo Chan, Srinivas Somanchi, Kelsey McCulley, Dean Lee, Nico Buettner, Feng Shi, Paisley T. Myers, Stuart Curbishley, Sarah A. Penny, Lora Steadman, David Millar, Ellen Speers, Nicola Ruth, Gabriel Wong, Robert Thimme, David Adams, Mark Cobbold, Remy Thomas, Wouter Hendrickx, Mariam Al-Muftah, Julie Decock, Michael KK Wong, Michael Morse, David F. McDermott, Joseph I. Clark, Howard L. Kaufman, Gregory A. Daniels, Hong Hua, Tharak Rao, Janice P. Dutcher, Kai Kang, Yogen Saunthararajah, Vamsidhar Velcheti, Vikas Kumar, Firoz Anwar, Amita Verma, Zinal Chheda, Gary Kohanbash, John Sidney, Kaori Okada, Shruti Shrivastav, Diego A. Carrera, Shuming Liu, Naznin Jahan, Sabine Mueller, Ian F. Pollack, Angel M. Carcaboso, Alessandro Sette, Yafei Hou, Hideho Okada, Jessica J. Field, Weiping Zeng, Vincent FS Shih, Che-Leung Law, Peter D. Senter, Shyra J. Gardai, Nicole M. Okeley, Sarah A. Penny, Jennifer G. Abelin, Abu Z. Saeed, Stacy A. Malaker, Paisley T. Myers, Jeffrey Shabanowitz, Stephen T. Ward, Donald F. Hunt, Mark Cobbold, Pam Profusek, Laura Wood, Dale Shepard, Petros Grivas, Kerstin Kapp, Barbara Volz, Detlef Oswald, Burghardt Wittig, Manuel Schmidt, Julian P. Sefrin, Lars Hillringhaus, Valeria Lifke, Alexander Lifke, Anna Skaletskaya, Jose Ponte, Thomas Chittenden, Yulius Setiady, Sandrine Valsesia-Wittmann, Eva Sivado, Vincent Thomas, Meddy El Alaoui, Sébastien Papot, Charles Dumontet, Mike Dyson, John McCafferty, Said El Alaoui, Amita Verma, Vikas Kumar, Praveen K. Bommareddy, Howard L. Kaufman, Andrew Zloza, Frederick Kohlhapp, Ann W. Silk, Sachin Jhawar, Tomas Paneque, Praveen K. Bommareddy, Frederick Kohlhapp, Jenna Newman, Pedro Beltran, Andrew Zloza, Howard L. Kaufman, Felicia Cao, Bang-Xing Hong, Tania Rodriguez-Cruz, Xiao-Tong Song, Stephen Gottschalk, Hugo Calderon, Sam Illingworth, Alice Brown, Kerry Fisher, Len Seymour, Brian Champion, Emma Eriksson, Jessica Wenthe, Ann-Charlotte Hellström, Gabriella Paul-Wetterberg, Angelica Loskog, Emma Eriksson, Ioanna Milenova, Jessica Wenthe, Magnus Ståhle, Justyna Jarblad-Leja, Gustav Ullenhag, Anna Dimberg, Rafael Moreno, Ramon Alemany, Angelica Loskog, Emma Eriksson, Ioanna Milenova, Rafael Moreno, Ramon Alemany, Angelica Loskog, Sachin Jhawar, Sharad Goyal, Praveen K. Bommareddy, Tomas Paneque, Howard L. Kaufman, Andrew Zloza, Howard L. Kaufman, Ann Silk, Janice Mehnert, Nashat Gabrail, Jennifer Bryan, Daniel Medina, Praveen K. Bommareddy, Darren Shafren, Mark Grose, Andrew Zloza, Leah Mitchell, Kader Yagiz, Fernando Lopez, Daniel Mendoza, Anthony Munday, Harry Gruber, Douglas Jolly, Steven Fuhrmann, Sasa Radoja, Wei Tan, Aldo Pourchet, Alan Frey, Ian Mohr, Matthew Mulvey, Tuuli Ranki, Sari Pesonen, Cristian Capasso, Erkko Ylösmäki, Vincenzo Cerullo, Robert H. I. Andtbacka, Merrick Ross, Sanjiv Agarwala, Kenneth Grossmann, Matthew Taylor, John Vetto, Rogerio Neves, Adil Daud, Hung Khong, Stephanie M. Meek, Richard Ungerleider, Scott Welden, Maki Tanaka, Matthew Williams, Robert H. I. Andtbacka, Brendan Curti, Sigrun Hallmeyer, Bernard Fox, Zipei Feng, Christopher Paustian, Carlo Bifulco, Mark Grose, Darren Shafren, Sadia Zafar, Suvi Parviainen, Mikko Siurala, Otto Hemminki, Riikka Havunen, Siri Tähtinen, Simona Bramante, Lotta Vassilev, Hongjie Wang, Andre Lieber, Silvio Hemmi, Tanja de Gruijl, Anna Kanerva, Akseli Hemminki, Tameem Ansari, Srividya Sundararaman, Diana Roen, Paul Lehmann, Anja C. Bloom, Lewis H. Bender, Ian B. Walters, Masaki Terabe, Jay A. Berzofsky, Fanny Chapelin, Hideho Okada, Eric T. Ahrens, Jeff DeFalco, Michael Harbell, Amy Manning-Bog, Alexander Scholz, Danhui Zhang, Gilson Baia, Yann Chong Tan, Jeremy Sokolove, Dongkyoon Kim, Kevin Williamson, Xiaomu Chen, Jillian Colrain, Gregg Espiritu Santo, Ngan Nguyen, Wayne Volkmuth, Norman Greenberg, William Robinson, Daniel Emerling, Charles G. Drake, Daniel P. Petrylak, Emmanuel S. Antonarakis, Adam S. Kibel, Nancy N. Chang, Tuyen Vu, Dwayne Campogan, Heather Haynes, James B. Trager, Nadeem A. Sheikh, David I. Quinn, Peter Kirk, Murali Addepalli, Thomas Chang, Ping Zhang, Marina Konakova, Katsunobu Hagihara, Steven Pai, Laurie VanderVeen, Palakshi Obalapur, Peiwen Kuo, Phi Quach, Lawrence Fong, Deborah H. Charych, Jonathan Zalevsky, John L. Langowski, Murali Addepalli, Yolanda Kirksey, Ravi Nutakki, Shalini Kolarkar, Rhoneil Pena, Ute Hoch, Jonathan Zalevsky, Stephen K. Doberstein, Deborah H. Charych, John Cha, Zach Mallon, Myra Perez, Amanda McDaniel, Snjezana Anand, Darrin Uecker, Richard Nuccitelli, Amanda McDaniel, Snjezana Anand, John Cha, Darrin Uecker, Richard Nuccitelli, Nataša Obermajer, Julie Urban, Eva Wieckowski, Ravikumar Muthuswamy, Roshni Ravindranathan, David Bartlett, Pawel Kalinski, Ariana N. Renrick, Menaka Thounaojam, Portia Thomas, Samuel Pellom, Anil Shanker, Samuel Pellom, Menaka Thounaojam, Duafalia Dudimah, Alan Brooks, Thomas J. Sayers, Anil Shanker, Yu-Lin Su, Tomasz Adamus, Qifang Zhang, Sergey Nechaev, Marcin Kortylewski, Spencer Wei, James Allison, Clark Anderson, Chad Tang, Jonathan Schoenhals, Efrosini Tsouko, John Heymach, Patricia de Groot, Joe Chang, Kenneth R. Hess, Adi Diab, Padmanee Sharma, James Allison, Aung Naing, David Hong, James Welsh, Tina C. Albershardt, Andrea J. Parsons, Jardin Leleux, Rebecca S. Reeves, Jan ter Meulen, Peter Berglund, Stephane Ascarateil, Marie Eve Koziol, Sarah A. Penny, Stacy A. Malaker, Lora Steadman, Paisley T. Myers, Dina Bai, Jeffrey Shabanowitz, Donald F. Hunt, Mark Cobbold, Peihong Dai, Weiyi Wang, Ning Yang, Stewart Shuman, Taha Merghoub, Jedd D. Wolchok, Liang Deng, Patrick Dillon, Gina Petroni, David Brenin, Kim Bullock, Walter Olson, Mark E. Smolkin, Kelly Smith, Carmel Nail, Craig L. Slingluff, Meenu Sharma, Faisal Fa’ak, Louise Janssen, Hiep Khong, Zhilan Xiao, Yared Hailemichael, Manisha Singh, Christina Vianden, Adi Diab, Jonathan Zalevsky, Ute Hoch, Willem W. Overwijk, Andrea Facciabene, Pierini Stefano, Fang Chongyung, Stavros Rafail, Yared Hailemichael, Michael Nielsen, Faisal Fa’ak, Peter Vanderslice, Darren G. Woodside, Robert V. Market, Ronald J. Biediger, Upendra K. Marathi, Willem W. Overwijk, Kevin Hollevoet, Nick Geukens, Paul Declerck, Nathalie Joly, Laura McIntosh, Eustache Paramithiotis, Magnus Rizell, Malin Sternby, Bengt Andersson, Alex Karlsson-Parra, Rui Kuai, Lukasz Ochyl, Anna Schwendeman, James Moon, Weiwen Deng, Thomas E. Hudson, Edward E. Lemmens, Bill Hanson, Chris S. Rae, Joel Burrill, Justin Skoble, George Katibah, Aimee L. Murphy, Michele deVries, Dirk G. Brockstedt, Meredith L. Leong, Peter Lauer, Thomas W. Dubensky, Chan C. Whiting, Xin Chen, Yangyang Hu, Yang Xia, Li Zhou, Yangyi Bao, Shiang Huang, Xiubao Ren, Elaine Hurt, Robert E. Hollingsworth, Alfred E. Chang, Max S. Wicha, Qiao Li, Charu Aggarwal, Drishty Mangrolia, Roger Cohen, Gregory Weinstein, Matthew Morrow, Joshua Bauml, Kim Kraynyak, Jean Boyer, Jian Yan, Jessica Lee, Laurent Humeau, Sandra Oyola, Susan Duff, David Weiner, Zane Yang, Mark Bagarazzi, Douglas G. McNeel, Jens Eickhoff, Robert Jeraj, Mary Jane Staab, Jane Straus, Brian Rekoske, Glenn Liu, Marit Melssen, Gina Petroni, William Grosh, Nikole Varhegyi, Kim Bullock, Mark E. Smolkin, Kelly Smith, Nadejda Galeassi, Donna H. Deacon, Elizabeth Gaughan, Craig L. Slingluff, Maurizio Ghisoli, Minal Barve, Robert Mennel, Gladice Wallraven, Luisa Manning, Neil Senzer, John Nemunaitis, Masahiro Ogasawara, Shuichi Ota, Kaitlin M. Peace, Diane F. Hale, Timothy J. Vreeland, Doreen O. Jackson, John S. Berry, Alfred F. Trappey, Garth S. Herbert, Guy T. Clifton, Mark O. Hardin, Anne Toms, Na Qiao, Jennifer Litton, George E. Peoples, Elizabeth A. Mittendorf, Lila Ghamsari, Emilio Flano, Judy Jacques, Biao Liu, Jonathan Havel, Vladimir Makarov, Taha Merghoub, Jedd D. Wolchok, Matthew D. Hellmann, Timothy A. Chan, Jessica B. Flechtner, Pierini Stefano, Andrea Facciabene, John Facciponte, Stefano Ugel, Francesco De Sanctis, George Coukos, Sébastien Paris, Agnes Pottier, Laurent Levy, Bo Lu, Federica Cappuccini, Emily Pollock, Richard Bryant, Freddie Hamdy, Adrian Hill, Irina Redchenko, Hussein Sultan, Takumi Kumai, Valentyna Fesenkova, Esteban Celis, Kwong Tsang, Massimo Fantini, Ingrid Fernando, Claudia Palena, Justin M. David, James Hodge, Elizabeth Gabitzsch, Frank Jones, James L. Gulley, Jeffrey Schlom, Mireia Uribe Herranz, Stavros Rafail, Stefano Ugel, John Facciponte, Pierini Stefano, Andrea Facciabene, Hiroshi Wada, Atsushi Shimizu, Toshihiro Osada, Satoshi Fukaya, Eiji Sasaki, Milad Abolhalaj, David Askmyr, Kristina Lundberg, Ann-Sofie Albrekt, Lennart Greiff, Malin Lindstedt, Dallas B. Flies, Tomoe Higuchi, Wojciech Ornatowski, Jaryse Harris, Sarah F. Adams, Todd Aguilera, Marjan Rafat, Laura Castellini, Hussein Shehade, Mihalis Kariolis, Dadi Jang, Rie vonEbyen, Edward Graves, Lesley Ellies, Erinn Rankin, Albert Koong, Amato Giaccia, Reham Ajina, Shangzi Wang, Jill Smith, Mariaelena Pierobon, Sandra Jablonski, Emanuel Petricoin, Louis M. Weiner, Lorcan Sherry, John Waller, Mark Anderson, Alison Bigley, Chantale Bernatchez, Cara Haymaker, Nizar M. Tannir, Harriet Kluger, Michael Tetzlaff, Natalie Jackson, Ivan Gergel, Mary Tagliaferri, Jonathan Zalevsky, Ute Hoch, Patrick Hwu, Mario Snzol, Michael Hurwitz, Adi Diab, Theresa Barberi, Allison Martin, Rahul Suresh, David Barakat, Sarah Harris-Bookman, Charles Drake, Alan Friedman, Sara Berkey, Stephanie Downs-Canner, Greg M. Delgoffe, Robert P. Edwards, Tyler Curiel, Kunle Odunsi, David Bartlett, Nataša Obermajer, Tullia C. Bruno, Brandon Moore, Olivia Squalls, Peggy Ebner, Katherine Waugh, John Mitchell, Wilbur Franklin, Daniel Merrick, Martin McCarter, Brent Palmer, Jeffrey Kern, Dario Vignali, Jill Slansky, Anissa S. H. Chan, Xiaohong Qiu, Kathryn Fraser, Adria Jonas, Nadine Ottoson, Keith Gordon, Takashi O. Kangas, Steven Leonardo, Kathleen Ertelt, Richard Walsh, Mark Uhlik, Jeremy Graff, Nandita Bose, Ravi Gupta, Nitin Mandloi, Kiran Paul, Ashwini Patil, Rekha Sathian, Aparna Mohan, Malini Manoharan, Amitabha Chaudhuri, Yu Chen, Jing Lin, Yun-bin Ye, Chun-wei Xu, Gang Chen, Zeng-qing Guo, Andrey Komarov, Alex Chenchik, Michael Makhanov, Costa Frangou, Yi Zheng, Carla Coltharp, Darryn Unfricht, Ryan Dilworth, Leticia Fridman, Linying Liu, Milind Rajopadhye, Peter Miller, Fernando Concha-Benavente, Julie Bauman, Sumita Trivedi, Raghvendra Srivastava, James Ohr, Dwight Heron, Uma Duvvuri, Seungwon Kim, William Gooding, Robert L. Ferris, Heather Torrey, Toshi Mera, Yoshiaki Okubo, Eva Vanamee, Rosemary Foster, Denise Faustman, Robyn Gartrell, Edward Stack, Yan Lu, Daisuke Izaki, Kristen Beck, Dan Tong Jia, Paul Armenta, Ashley White-Stern, Yichun Fu, Zoe Blake, Douglas Marks, Howard L. Kaufman, Bret Taback, Basil Horst, Yvonne M. Saenger, Laura Hix Glickman, David B. Kanne, Kelsey S. Gauthier, Anthony L. Desbien, Brian Francica, George Katibah, Leticia P. Corrales, Justin L. Leong, Leonard Sung, Ken Metchette, Shailaja Kasibhatla, Anne Marie Pferdekamper, Lianxing Zheng, Charles Cho, Yan Feng, Jeffery M. McKenna, John Tallarico, Steven Bender, Chudi Ndubaku, Sarah M. McWhirter, Charles G. Drake, Thomas F. Gajewski, Thomas W. Dubensky, Elena Gonzalez Gugel, Charles J. M. Bell, Adiel Munk, Luciana Muniz, Nina Bhardwaj, Fei Zhao, Kathy Evans, Christine Xiao, Alisha Holtzhausen, Brent A. Hanks, Nathalie Scholler, Catherine Yin, Pien Van der Meijs, Andrew M. Prantner, Cecile M. Krejsa, Leia Smith, Brian Johnson, Daniel Branstetter, Paul L. Stein, Juan C. Jaen, Joanne BL Tan, Ada Chen, Yu Chen, Timothy Park, Jay P. Powers, Holly Sexton, Guifen Xu, Steve W. Young, Ulrike Schindler, Wentao Deng, David John Klinke, Hannah M. Komar, Thomas Mace, Gregory Serpa, Omar Elnaggar, Darwin Conwell, Philip Hart, Carl Schmidt, Mary Dillhoff, Ming Jin, Michael C. Ostrowski, Gregory B. Lesinski, Madhuri Koti, Katrina Au, Nichole Peterson, Peter Truesdell, Gillian Reid-Schachter, Charles Graham, Andrew Craig, Julie-Ann Francis, Beatrix Kotlan, Timea Balatoni, Emil Farkas, Laszlo Toth, Mihaly Ujhelyi, Akos Savolt, Zoltan Doleschall, Szabolcs Horvath, Klara Eles, Judit Olasz, Orsolya Csuka, Miklos Kasler, Gabriella Liszkay, Eytan Barnea, Sushil Kumar, Takahiro Tsujikawa, Collin Blakely, Patrick Flynn, Reid Goodman, Raphael Bueno, David Sugarbaker, David Jablons, V. Courtney Broaddus, Brian West, Lisa M. Coussens, Paul R. Kunk, Joseph M. Obeid, Kevin Winters, Patcharin Pramoonjago, Mark E. Smolkin, Edward B. Stelow, Todd W. Bauer, Craig L. Slingluff, Osama E. Rahma, Adam Lamble, Yoko Kosaka, Fei Huang, Kate A. Saser, Homer Adams, Christina E. Tognon, Ted Laderas, Shannon McWeeney, Marc Loriaux, Jeffery W. Tyner, Brian J. Druker, Evan F. Lind, Zhuqing Liu, Shanhong Lu, Lawrence P. Kane, Robert L. Ferris, Zhuqing Liu, Gulidanna Shayan, Shanhong Lu, Robert L. Ferris, Julia Femel, Takahiro Tsujikawa, Ryan Lane, Jamie Booth, Amanda W. Lund, Marit Melssen, Anthony Rodriguez, Craig L. Slingluff, Victor H. Engelhard, Alessandra Metelli, Bill X. Wu, Caroline W. Fugle, Rachidi Saleh, Shaoli Sun, Jennifer Wu, Bei Liu, Zihai Li, Zachary S. Morris, Emily I. Guy, Clinton Heinze, Jasdeep Kler, Monica M. Gressett, Lauryn R. Werner, Stephen D. Gillies, Alan J. Korman, Hans Loibner, Jacquelyn A. Hank, Alexander L. Rakhmilevich, Paul M. Harari, Paul M. Sondel, Jenna Newman, Andrew Zloza, Erica Huelsmann, Joseph Broucek, Howard L. Kaufman, Dorothee Brech, Tobias Straub, Martin Irmler, Johannes Beckers, Florian Buettner, Elke Schaeffeler, Matthias Schwab, Elfriede Noessner, Snjezana Anand, Amanda McDaniel, John Cha, Darrin Uecker, Richard Nuccitelli, Peter Ordentlich, Alison Wolfreys, Andre Da Costa, John Silva, Andrea Crosby, Ludovicus Staelens, Graham Craggs, Annick Cauvin, Sean Mason, Alison M. Paterson, Andrew C. Lake, Caroline M. Armet, Rachel W. O’Connor, Jonathan A. Hill, Emmanuel Normant, Ammar Adam, Detlev M. Biniszkiewicz, Scott C. Chappel, Vito J. Palombella, Pamela M. Holland, Jay P. Powers, Annette Becker, Ada Chen, Manmohan R. Leleti, Eric Newcomb, Holly Sexton, Ulrike Schindler, Joanne B. L. Tan, Steve W. Young, Juan C. Jaen, Suthee Rapisuwon, Arash Radfar, Kellie Gardner, Geoffrey Gibney, Michael Atkins, Keith R. Rennier, Robert Crowder, Ping Wang, Russell K. Pachynski, Rosa M. Santana Carrero, Sarai Rivas, Figen Beceren-Braun, Scott Anthony, Kimberly S. Schluns, Deepali Sawant, Maria Chikina, Hiroshi Yano, Creg Workman, Dario Vignali, Elise Salerno, Davide Bedognetti, Ileana Mauldin, Donna Deacon, Sofia Shea, Joel Pinczewski, Joseph M. Obeid, George Coukos, Ena Wang, Thomas Gajewski, Franco M. Marincola, Craig L. Slingluff, Stefani Spranger, Brendan Horton, Thomas F. Gajewski, Akiko Suzuki, Pamela Leland, Bharat H. Joshi, Raj K. Puri, Randy F. Sweis, Riyue Bao, Jason Luke, Thomas F. Gajewski, Marie-Nicole Theodoraki, Frances-Mary Mogundo, Robert P. Edwards, Pawel Kalinski, Haejung Won, Dayson Moreira, Chan Gao, Xingli Zhao, Priyanka Duttagupta, Jeremy Jones, Massimo D’Apuzzo, Sumanta Pal, Marcin Kortylewski

**Affiliations:** 1 Department of Immunology, University of Texas MD Anderson Cancer Center, Houston, TX USA; 2 Department of Cancer Medicine, University of Texas MD Anderson Cancer Center, Houston, TX USA; 3 Immune Design, Seattle, WA USA; 4 Innate Pharma, Marseille, Provence-Alpes-Cote d’Azur France; 5 MedImmune, Cambridge, England UK; 6 Georgetown-Lombardi Comprehensive Cancer Center, Washington, DC USA; 7 Westmead and Blacktown Hospitals, Melanoma Institute Australia, and the University of Sydney, Westmead, New South Wales Australia; 8 University of California, Los Angeles, CA USA; 9 University of Washington, Seattle, WA USA; 10 Dana-Farber Cancer Institute/Brigham and Women’s Hospital, Harvard University, Boston, MA USA; 11 University of Texas MD Anderson Cancer Center, Houston, TX USA; 12 Beth Israel Deaconess Medical Center, Boston, MA USA; 13 Gallipoli Medical Research Foundation, Greenslopes Private Hospital, and the University of Queensland, Greenslopes, Queensland Australia; 14 Olivia Newton-John Cancer Research Institute, Heidelberg, Victoria Australia; 15 Canterbury District Health Board, Christchurch Hospital, Christchurch, New Zealand; 16 Waikato Hospital Regional Cancer Centre, Hamilton, New Zealand; 17 Royal Prince Alfred Hospital, Melanoma Institute Australia, the University of Sydney, and Chris O’Brien Lifehouse, Camperdown, New South Wales Australia; 18 Tasman Oncology Research, Southport Gold Coast, Queensland Australia; 19 Merck & Co., Inc., Kenilworth, NJ USA; 20 Quantitative Solutions, A Certara Company, Oss, Netherlands; 21 Melanoma Institute Australia, the University of Sydney, Mater Hospital, and Royal North Shore Hospital, Wollstonecraft, New South Wales Australia; 22 Columbia University Medical Center, New York, NY USA; 23 Institut d’Investigacions Biomediques August Pi i Sunyer, Barcelona, Catalonia Spain; 24 Icahn School of Medicine at Mount Sinai, New York, NY USA; 25 New York Presbyterian/Columbia University Medical Center, New York, NY USA; 26 Memorial Sloan Kettering Cancer Center, New York, NY USA; 27 Peregrine Pharmaceuticals, Inc., Tustin, CA USA; 28 Regeneron, Tarrytown, NY USA; 29 University of Helsinki, Helsinki, Uusimaa Finland; 30 University of Siena, Supersano (LE), Puglia Italy; 31 University of Pisa, Pisa, Toscana Italy; 32 University of Napoli Federico II, Helsinki, Uusimaa Finland; 33 PeptiCRAd Oy, Helsinki, Uusimaa Finland; 34 ACEA Biosciences Inc., San Diego, CA USA; 35 TILT Biotherapeutics, Helsinki, Uusimaa Finland; 36 University of Helsinki, Helsinki, Uusimaa Finland; 37 St George’s University of London, London, UK; 38 The Royal Marsden Hospital and Imperial College London, London, UK; 39 Argos Therapeutics Inc., Durham, NC USA; 40 University of Texas MD Anderson Cancer Center, Houston, TX USA; 41 Laboratory of Tumor Immunology and Biology, National Cancer Institute, National Institutes of Health, Bethesda, MD USA; 42 Vaccinex, Rochester, NY USA; 43 University of California, Los Angeles, CA USA; 44 GlycoMimetics, Inc., Rockville, MD USA; 45 MI Bioresearch, Ann Arbor, MI USA; 46 Peregrine Pharmaceuticals, Tustin, CA USA; 47 Heat Biologics, Inc., Durham, NC USA; 48 National Cancer Institute, National Institutes of Health, Bethesda, MD USA; 49 National Institute on Deafness and Other Communication Disorders, National Institutes of Health, Bethesda, MD USA; 50 Peregrine Pharmaceuticals, Tustin, CA USA; 51 Genentech, South San Francisco, CA USA; 52 Celgene Corporation, Summit, NJ USA; 53 Celgene Corporation, San Francisco, CA USA; 54 University of Pennsylvania, Philadelphia, PA USA; 55 Children’s Hospital of Pennsylvania, Philadelphia, PA USA; 56 Corvus Pharmaceuticals, Burlingame, CA USA; 57 CytomX Therapeutics, Inc, South San Francisco, CA USA; 58 CytomX Therapeutics, Inc, South San Francisco, CA USA; 59 Lerner Research Institute, Cleveland Clinic, Cleveland, OH USA; 60 Sarah Cannon Research Institute, Nashville, TN USA; 61 Mayo Clinic, Rochester, MN USA; 62 Roswell Park Cancer Institute, Buffalo, NY USA; 63 Emory University, Atlanta, GA USA; 64 Dana-Farber Cancer Institute, Boston, MA USA; 65 The Wistar Institute, Philadelphia, PA USA; 66 Syndax Pharmaceuticals, Inc, Waltham, MA USA; 67 Merck Research Laboratories, North Wales, PA USA; 68 Syndax Pharmaceuticals, Inc, New York, NY USA; 69 Department of Medicine, Memorial Sloan Kettering Cancer Center, New York, NY USA; 70 Department of Surgery, University of Pittsburgh Cancer Institute, Pittsburgh, PA USA; 71 Department of Infectious Diseases and Microbiology, University of Pittsburgh, Pittsburgh, PA USA; 72 University of Pittsburgh Cancer Institute, Pittsburgh, PA USA; 73 University of Pittsburgh, Pittsburgh, PA USA; 74 Department of Surgery, University of Pittsburgh, Pittsburgh, PA USA; 75 LLC Cellthera Pharm, Volginsky, Vladimir Russia; 76 LLC IBC Generium, Volginsky, Vladimir Russia; 77 Argos Therapeutics Inc., Durham, NC USA; 78 Memorial Sloan Kettering Cancer Center, New York, NY USA; 79 Ludwig Collaborative Laboratory, Memorial Sloan Kettering Cancer Center, New York, NY USA; 80 Department of Medicine, Memorial Sloan Kettering Cancer Center, New York, NY USA; 81 Curis, Inc., Lexington, MA USA; 82 Aurigene Discovery Technologies Limited, Bangalore, Karnataka India; 83 National Cancer Institute, NIH, Bethesda, MD USA; 84 Roche Pharmaceutical Research &Early Development, Discovery Oncology, Innovation Center Penzberg, Roche Diagnostics GmbH, Penzberg, Germany; 85 Technion Institute, Haifa, Iceland; 86 University of Michigan, Ann Arbor, MI USA; 87 The No.1 People’s Hospital of Hefei, Hefei, Anhui People’s Republic of China; 88 Union Hospital, Tongji Medical College, Huazhong University of Science and Technology, Wuhan, Hubei People’s Republic of China; 89 MedImmune Inc, Gaithersburg, MD USA; 90 University of Virginia Cancer Center, Charlottesville, VA USA; 91 University of Michigan Medical School, Ann Arbor, MI USA; 92 University of Michigan Medical Center, Ann Arbor, MI USA; 93 The Ohio State University, Columbus, OH USA; 94 Columbia Center for Translational Immunology, Columbia University Medical Center, New York, NY USA; 95 Calithera Biosciences, South San Francisco, CA USA; 96 Calithera Biosciences, South San Francisco, CA USA; 97 Icahn School of Medicine at Mount Sinai, New York, NY USA; 98 The Ohio State University, Columbus, OH USA; 99 Laboratory of Tumor Immunology and Biology, Center for Cancer Research, National Cancer Institute, Bethesda, MD USA; 100 MedImmune LLC, Gaithersburg, MD USA; 101 National Cancer Institute, National Institutes of Health, Bethesda, MD USA; 102 National Institute of Neurological Disorders and Stroke, National Institutes of Health, Bethesda, MD USA; 103 Department of Surgery, University of Pittsburgh, Pittsburgh, PA USA; 104 Department of Surgery, University of Pittsburgh Cancer Institute, Pittsburgh, PA USA; 105 Department of Infectious Diseases and Microbiology, University of Pittsburgh, Pittsburgh, PA USA; 106 University of Texas MD Anderson Cancer Center, Houston, TX USA; 107 South Texas Accelerated Research Therapeutics, LLC, San Antonio, TX USA; 108 Memorial Sloan Kettering Cancer Center, New York, NY USA; 109 UCLA, Los Angeles, CA USA; 110 Sarah Cannon Research Institute/Florida Cancer Specialists, Sarasota, FL USA; 111 SCRI at HealthONE, Denver, CO USA; 112 Oklahoma University, Oklahoma City, OK USA; 113 Dana-Farber Cancer Institute, Boston, MA USA; 114 ARMO BioSciences, Redwood City, CA USA; 115 Sarah Cannon Research Institute, Nashville, TN USA; 116 Sidney Kimmel Comprehensive Cancer Center, Johns Hopkins University School of Medicine, Baltimore, MD USA; 117 Seattle Cancer Care Alliance, University of Washington, Seattle, WA USA; 118 University of Texas MD Anderson Cancer Center, Houston, TX USA; 119 Dana-Farber Cancer Institute, Harvard University, Boston, MA USA; 120 Center for Hematologic Malignancies, Oregon Health and Sciences University, Portland, OR USA; 121 New York Presbyterian Hospital, Weill Cornell Medical College, New York, NY USA; 122 Robert W. Franz Cancer Research Center, Earle A. Chiles Research Institute, Providence Cancer Center, Portland, OR USA; 123 Center for Applied Medical Research (CIMA), University of Navarra, Pamplona, Navarra Spain; 124 University of Chicago Medical Center, Chicago, IL USA; 125 Bristol-Myers Squibb, Princeton, NJ USA; 126 Bristol-Myers Squibb, Lawrence Township, NJ USA; 127 Memorial Sloan Kettering Cancer Center, New York, NY USA; 128 MCCIR, University of Manchester, Manchester, England UK; 129 Oncodesign, Dijon, Bourgogne France; 130 Immodulon Therapeutics Ltd, Uxbridge, England UK; 131 University of Pennsylvania Perelman School of Medicine, Philadelphia, PA USA; 132 Moores Cancer Center, University of California, San Diego, La Jolla, CA USA; 133 Dynavax Technologies, Berkeley, CA USA; 134 Repertoire Genesis Incorporation, Ibaraki, Osaka Japan; 135 Eli Lilly and Company, New York, NY USA; 136 Eli Lilly and Company, Indianapolis, IN USA; 137 Eli Lilly and Company, Bridgewater, NJ USA; 138 University of Surrey, Guildford, England UK; 139 St James University Hospital, Leeds, England UK; 140 Institute for Cancer Research, London, England UK; 141 Providence Cancer Center, Portland, OR USA; 142 Robert W. Franz Cancer Research Center, Earle A. Chiles Research Institute, Providence Cancer Center, Portland, Oregon USA; 143 Huntsman Cancer Institute, Salt Lake City, UT USA; 144 Institute for Cancer Research, London, England UK; 145 Merck & Co., Inc., Kenilworth, NJ USA; 146 Viralytics Ltd., Sydney, New South Wales Australia; 147 Bar Ilan University, Ramat Gan, HaMerkaz, Iceland; 148 University of Texas MD Anderson Cancer Center, Houston, TX USA; 149 Université Claude Bernard Lyon 1, Centre Leon Berard, Lyon, Rhone-Alpes France; 150 Centre Leon Berard, Innovations in Immunotherapy Platform, Lyon, Rhone-Alpes France; 151 Institue Goustave Roussy, Université Claude Bernard Lyon 1, Centre Leon Berard, Villejuif, Ile-de-France France; 152 Sylvester Comprehensive Cancer Center/University of Miami, Miami, FL USA; 153 Stephenson Oklahoma Cancer Center, Oklahoma City, OK USA; 154 Advaxis Immunotherapies, Princeton, NJ USA; 155 Icahn School of Medicine at Mount Sinai, Mount Sinai Medical Center, New York, NY USA; 156 CIP, Center for Cancer Research, BSP, Leidos Biomed Research Inc, National Cancer Institute-Frederick, Frederick, MD USA; 157 CIP, Center for Cancer Research, Leidos Biomed Research Inc, National Cancer Institute-Frederick, Frederick, MD USA; 158 University of Arizona, Southwest Center for Natural Products Research and Commercialization, Tucson, AZ USA; 159 University of Arizona, Tucson, AZ USA; 160 3M Company, St. Paul, MN USA; 161 Medimmune, Cambridge, England UK; 162 Medimmune, Gaithersburg, MD USA; 163 Georgetown University/Medical School/Lombardi Cancer Center, Washington, DC USA; 164 Pfizer, San Diego, CA USA; 165 iTeos, 6041 Gosselies, Brussels, Hoofdstedelijk Gewest Belgium; 166 iTeos, Rue Auguste Piccard 48, Brussels, Hoofdstedelijk Gewest Belgium; 167 EMD Serono, Belmont, MA USA; 168 EMD Serono, Billerica, MA USA; 169 Ludwig Collaborative Laboratory, Memorial Sloan Kettering Cancer Center, New York, NY USA; 170 Department of Medicine, Memorial Sloan Kettering Cancer Center, New York, NY USA; 171 University of Chicago, OSRF-HIM, Chicago, IL USA; 172 University of Chicago, Chicago, IL USA; 173 Section of Hematology/Oncology, University of Chicago, Chicago, IL USA; 174 University of Chicago Medical Center, Chicago, IL USA; 175 Advaxis Immunotherapies, Princeton, NJ USA; 176 The University of Chicago, Chicago, IL USA; 177 UT Southwestern, Dallas, TX USA; 178 Argos Therapeutics, Durham, NC USA; 179 R&D Department, HRYZ Biotech Co., Shenzhen, Guangdong People’s Republic of China; 180 Department of Infectious Diseases and Hepatology Unit, Nanfang Hospital, Guangzhou, Guangdong People’s Republic of China; 181 HRYZ Biotech Co., Shenzhen, Guangdong People’s Republic of China; 182 Thoracic and GI Oncology Branch, National Cancer Institute, Bethesda, MD USA; 183 Department of Medicine, Division of Hematology Oncology, UCSF, San Francisco, CA USA; 184 H. Lee Moffitt Cancer Center, Tampa, FL USA; 185 Gastrointestinal Oncology and Mesothelioma Programs, Section of Hematology/Oncology, University of Chicago, Chicago, IL USA; 186 Penn Prebyterian Medical Center, University of Pennsylvania, Philadelphia, PA USA; 187 Aduro Biotech, Inc., Berkeley, CA USA; 188 Oregon Health & Science University, Portland, OR USA; 189 CureVac AG, Tubingen, Baden-Wurttemberg Germany; 190 University Hospital Frankfurt, Medical Clinic II, Goethe University, Frankfurt, Hessen Germany; 191 Klinikum Darmstadt GmbH, Darmstadt, Hessen Germany; 192 Kantonsspital St. Gallen, St. Gallen, Switzerland; 193 Kantonsspital Winterthur, Winterthur, Zurich Switzerland; 194 Kantonsspital Graubünden, Chur, Graubunden Switzerland; 195 Wilhelminenspital Wien, Wien, Austria; 196 Fachkliniken Wangen, Wangen (Allgäu), Baden-Wurttemberg Germany; 197 J. Gutenberg University Hospital Mainz, Mainz, Rheinland-Pfalz Germany; 198 Thoraxklinik Heidelberg gGmbH, Heidelberg, Baden-Wurttemberg Germany; 199 Klinikum Esslingen GmbH, Esslingen, Baden-Wurttemberg Germany; 200 Pius Hospital Oldenburg, Oldenburg, Niedersachsen Germany; 201 Heckeshorn Lung Clinic, Berlin, Germany; 202 University Hospital Basel, Basel-Stadt, Switzerland; 203 CureVac AG, Frankfurt am Main, Hessen Germany; 204 SNU Cancer Research Institute, Seoul, Republic of Korea; 205 Seoul National University Hospital, SNU Cancer Research Institute, Seoul, Republic of Korea; 206 H. Lee Moffitt Cancer Center, Tampa, FL USA; 207 Brigham and Women’s/Dana-Farber Cancer Center, Harvard University, Boston, MA USA; 208 Harvard Radiation Oncology Program, Boston, MA USA; 209 Dana-Farber Cancer Institute, Harvard University, Boston, MA USA; 210 National Cancer Institute, Bethesda, MD USA; 211 National Cancer Institute, Frederick, MD USA; 212 Department of Pathology, National Cancer Institute, Bethesda, MD USA; 213 Memorial Sloan Kettering Cancer Center, New York, NY USA; 214 University of Chicago Medicine, Chicago, IL USA; 215 Sidney Kimmel Comprehensive Cancer Center at Johns Hopkins University, Baltimore, MD USA; 216 Dana-Farber Cancer Institute, Boston, MA USA; 217 University of Pittsburgh, Pittsburgh, PA USA; 218 University of Texas MD Anderson Cancer Center, Houston, TX USA; 219 Stanford University School of Medicine, Stanford, CA USA; 220 University of Miami Miller School of Medicine, Sylvester Comprehensive Cancer Center, Miami, FL USA; 221 Karmanos Cancer Institute, Detroit, MI USA; 222 Earle A. Chiles Research Institute, Providence Cancer Center, Portland, OR USA; 223 Bristol-Myers Squibb, Princeton, NJ USA; 224 UCLA Medical Center, Los Angeles, CA USA; 225 Department of Radiation Oncology, Weill Cornell Medicine, New York, NY USA; 226 Weill Cornell Medical College, New York, NY USA; 227 New York University Langone Medical Center, New York, NY USA; 228 Department of Radiation Oncology, Weill Cornell Medicine, New York, NY USA; 229 Mayo Clinic, Rochester, MN USA; 230 Roswell Park Cancer Institute, Buffalo, NY USA; 231 Pennsylvania State University, University Park, PA USA; 232 University of California, Davis, Sacramento, CA USA; 233 University of Minnesota, Minneapolis, MN USA; 234 Dana-Farber Cancer Institute, Boston, MA USA; 235 Aeglea BioTherapeutics, Austin, TX USA; 236 University of Pittsburgh, Pittsburgh, PA USA; 237 University of Pittsburgh Cancer Institute, Pittsburgh, PA USA; 238 Karmanos Cancer Institute, Detroit, MI USA; 239 iRepertoire, Inc, Huntsville, AL USA; 240 Dana-Farber Cancer Institute, Boston, MA USA; 241 Broad Institute, Cambridge, MA USA; 242 University of Chicago, Chicago, IL USA; 243 University of Chicago Medical Center, Chicago, IL USA; 244 University of Sassari, Sassari, Sardegna Italy; 245 Sidra Medical and Research Center, Doha, Ad Dawhah Qatar; 246 University of Roma Tor Vergata, Roma, Lazio Italy; 247 Research Branch, Sidra Medical and Research Center, Doha, Ad Dawhah Qatar; 248 Faculty of Medical & Human Sciences, University of Manchester, Manchester, England UK; 249 CNRS, Institute de Biologie Moléculaire et Cellulaire, Strasbourg, Alsace France; 250 Sidra Medical and Research Center, Doha, Ad Dawhah Qatar; 251 Qatar Biomedical Research Institute (QBRI), Doha, Ar Rayyan Qatar; 252 Benaroya Research Institute, Seattle, WA USA; 253 Leiden University Medical Center, Leiden, Zuid-Holland Netherlands; 254 Qatar Computing Research Institute, Doha, Ad Dawhah Qatar; 255 Wake Forest School of Medicine, Winston-Salem, NC USA; 256 NantOmics, San Jose, CA USA; 257 NantOmics, Santa Cruz, CA USA; 258 National Cancer Institute, Bethesda, MD USA; 259 Personal Genome Diagnostics, Baltimore, MD USA; 260 Columbia University Medical Center, New York, NY USA; 261 Fordham University, New York, NY USA; 262 New York Presbyterian/Columbia University Medical Center, New York, NY USA; 263 Earle A. Chiles Research Institute, Robert W. Franz Cancer Center, Providence Portland Medical Center, Portland, OR USA; 264 Earle A. Chile Research Institute, Portland, OR USA; 265 Providence Portland Medical Center, Portland, OR USA; 266 Georgia State University, Atlanta, GA USA; 267 University of Chicago, Chicago, IL USA; 268 Department of Medicine, University of Chicago, Chicago, IL USA; 269 Committee on Immunology and Department of Medicine, University of Chicago, Chicago, IL USA; 270 University of California, Davis, Sacramento, CA USA; 271 Dana-Farber Cancer Institute, Boston, MA USA; 272 Evelo Biosciences, Cambridge, MA USA; 273 Harvard Medical School, Boston, MA USA; 274 Receptome, LLC, La Jolla, CA USA; 275 Miller School of Medicine, University of Miami, Miami, FL USA; 276 EMD Serono, Billerica, MA USA; 277 Merck KGaA, Darmstadt, Hessen Germany; 278 Compass Therapeutics, Cambridge, MA USA; 279 Agenus Inc., Lexington, MA USA; 280 TCR2 Therapeutics, Cambridge, MA USA; 281 University of Lausanne, Ludwig Cancer Research, Epalinges, Vaud Switzerland; 282 SIB Swiss Institute of Bioinformatics, Lausanne, Vaud Switzerland; 283 Thomas Jefferson University, Philadelphia, PA USA; 284 Department of Internal Medicine, University of Zielona Gora, Zielona Gora, Lubuskie Poland; 285 Department of Medical Biotechnology, Jagiellonian University, Krakow, Malopolskie Poland; 286 Department of Medical Analytics, Pomeranian Medical University, Szczecin, Zachodniopomorskie Poland; 287 Department of Microbiology and Immune Diagnostics, Pomeranian Medical University, Szczecin, Zachodniopomorskie Poland; 288 Department of Gastroenterology, Pomeranian Medical University, Szczecin, Zachodniopomorskie Poland; 289 Nexcelom Bioscience, Lawrence, MA USA; 290 University of Texas MD Anderson Cancer Center, Houston, TX USA; 291 Nexcelom Bioscience, Houston, MA USA; 292 Universitätsklinikum Freiburg, Freiburg, Germany; 293 Massachusetts General Hospital Cancer Center, Boston, MA USA; 294 University of Virginia, Charlottesville, VA USA; 295 University of Birmingham, Birmingham, England UK; 296 Qatar Biomedical Research Instititute (QBRI), Doha, Ar Rayyan Qatar; 297 Sidra Medical and Research Center, Doha, Ad Dawhah Qatar; 298 University of Texas MD Anderson Cancer Center, Houston, TX USA; 299 Duke University Medical Center, Durham, NC USA; 300 Beth Israel Deaconess Medical Center, Boston, MA USA; 301 Loyola University Medical Center, Maywood, IL USA; 302 Rutgers Cancer Institute of New Jersey, New Brunswick, NJ USA; 303 UC San Diego Moores Cancer Center, La Jolla, CA USA; 304 Prometheus Laboratories Inc., San Diego, CA USA; 305 Cancer Research Foundation, Chappaqua, NY USA; 306 Taussig Cancer Institute, Cleveland Clinic, Cleveland, OH USA; 307 Cleveland Clinic, Cleveland, OH USA; 308 Sam Higginbotham Institute of Agriculture, Technology & Sciences, Allahabad, Uttar Pradesh India; 309 King Abdulaziz University, Jeddah, Afghanistan; 310 University of California, San Francisco, San Francisco, CA USA; 311 La Jolla Institute for Allergy and Immunology, La Jolla, CA USA; 312 University of Pittsburgh, Pittsburgh, PA USA; 313 Hospital Sant Joan de Déu Barcelona, Barcelona, Spain; 314 Seattle Genetics, Inc., Bothell, WA USA; 315 University of Birmingham, Birmingham, England UK; 316 University of Virginia, Charlottesville, VA USA; 317 Stanford University, Stanford, CA USA; 318 Massachusetts General Hospital Cancer Center, Boston, MA USA; 319 Cleveland Clinic, Cleveland, OH USA; 320 Mologen AG, Berlin, Germany; 321 Foundation Institute Molecular Biology and Bioinformatics, Freie Universitaet Berlin, Berlin, Germany; 322 Roche Diagnostics GmbH, Penzberg, Bayern Germany; 323 ImmunoGen, Inc., Waltham, MA USA; 324 Centre Léon Bérard, Innovations in immunotherapy platform, Lyon, Rhone-Alpes France; 325 Covalab, Villeurbanne, Rhone-Alpes France; 326 IC2MP, Poitiers, Limousin France; 327 CRCL, Lyon, Rhone-Alpes France; 328 IONTAS, Cambridge, England UK; 329 Sam Higginbotham Institute of Agriculture, Technology & Sciences, Allahabad, Uttar Pradesh India; 330 Rutgers University, New Brunswick, NJ USA; 331 Rutgers Cancer Institute of New Jersey, New Brunswick, NJ USA; 332 Rutgers Robert Wood Johnson Medical School, Somerset, NJ USA; 333 Rutgers University, New Brunswick, NJ USA; 334 Rutgers Cancer Institute of New Jersey, New Brunswick, NJ USA; 335 Center for Cell and Gene Therapy, Texas Children’s Hospital, Houston Methodist, Baylor College of Medicine, Houston, TX USA; 336 PsiOxus Therapeutics Ltd, Abingdon, England UK; 337 Oxford University, Oxford, England UK; 338 Uppsala University, Uppsala, Sweden; 339 Uppsala University, Lokon Pharma AB, Uppsala, Sweden; 340 Uppsala University, Uppsala, Sweden; 341 Uppsala University, Amsterdam, Netherlands; 342 Uppsala University, Immuneed AB, Uppsala, Sweden; 343 Uppsala University, Uppsala University Hospital, Uppsala, Sweden; 344 Institut Catalá d’Oncologia, Barcelona, Spain; 345 Uppsala University, Lokon Pharma AB, Uppsala, Sweden; 346 Uppsala University, Uppsala, Sweden; 347 Uppsala University, Amsterdam, Netherlands; 348 Institut Catalá d’Oncologia, Barcelona, Spain; 349 Uppsala University, Lokon Pharma AB, Uppsala, Sweden; 350 Rutgers University, New Brunswick, NJ USA; 351 Rutgers Cancer Institute of New Jersey, New Brunswick, NJ USA; 352 Rutgers Robert Wood Johnson Medical School, Somerset, NJ USA; 353 Rutgers Cancer Institute of New Jersey, New Brunswick, NJ USA; 354 Gabrail Cancer Center, Canton, OH USA; 355 Viralytics Limited, Sydney, New South Wales Australia; 356 Tocagen Inc, San Diego, CA USA; 357 BeneVir Biopharm, Inc, Gaithersburg, MD USA; 358 New York University Langone School of Medicine, New York, NY USA; 359 PeptiCRAd Oy, Helsinki, Uusimaa Finland; 360 University of Helsinki, Helsinki, Uusimaa Finland; 361 University of Utah, Huntsman Cancer Institute, Salt Lake City, UT USA; 362 Univesity of Texas MD Anderson Cancer Center, Houston, TX USA; 363 St. Luke’s Hospital, Easton, PA USA; 364 Oregon Health & Science University, Portland, OR USA; 365 Knight Cancer Institute, Oregon Health and Science University, Portland, OR USA; 366 Pennsylvania State University, Hershey Cancer Institute, Hershey, PA USA; 367 UCSF Helen Diller Family Comprehensive Cancer Center, San Francisco, CA USA; 368 University of Utah School of Medicine, Salt Lake City, UT USA; 369 Theradex, Princeton, NJ USA; 370 Takara Bio, Inc, Otsu, Shiga Japan; 371 University of Utah, Salt Lake City, UT USA; 372 University of Utah, Huntsman Cancer Institute, Salt Lake City, UT USA; 373 Providence Cancer Center, Portland, OR USA; 374 Oncology Specialists, Chicago, IL USA; 375 Robert W. Franz Cancer Research Center, Earle A. Chiles Research Institute, Providence Cancer Center, Portland, OR USA; 376 Viralytics Limited, Sydney, New South Wales Australia; 377 University of Helsinki, Helsinki, Uusimaa Finland; 378 University of Washington, Seattle, WA USA; 379 University of Zurich, Zurich, Switzerland; 380 VU University Medical Center, Amsterdam, Netherlands; 381 Cellular Technology Ltd, Shaker Hts, OH USA; 382 National Cancer Institute, Bethesda, MD USA; 383 Intensity Therapeutics, Inc., Westport, CT USA; 384 University of California, San Diego, La Jolla, CA USA; 385 University of California, San Francisco, San Francisco, CA USA; 386 Atreca, Redwood City, CA USA; 387 Stanford University School of Medicine, Stanford, CA USA; 388 Johns Hopkins University Cancer Center, Baltimore, MD USA; 389 Yale School of Medicine, New Haven, CT USA; 390 Johns Hopkins University, Sidney Kimmel Cancer Center, Baltimore, MD USA; 391 Harvard Medical School, Dana-Farber Cancer Center, and Brigham and Women’s Hospital, Boston, MA USA; 392 Dendreon Pharmaceuticals, Inc, Seattle, WA USA; 394 Nektar Therapeutics, San Francisco, CA USA; 395 University of California, San Francisco School of Medicine, San Francisco, CA USA; 396 Hellen Diller Family Comprehensive Cancer Center, San Francisco, CA USA; 397 Nektar Therapeutics, San Francisco, CA USA; 398 Pulse Biosciences, Burlingame, CA USA; 399 Pulse Biosciences, Burlingame, CA USA; 400 Department of Surgery, University of Pittsburgh, Pittsburgh, PA USA; 401 University of Pittsburgh, Pittsburgh, PA USA; 402 Department of Surgery, University of Pittsburgh Cancer Institute, Pittsburgh, USA; 403 Department of Infectious Diseases and Microbiology, University of Pittsburgh, Pittsburgh, PA USA; 404 Meharry Medical College, Nashville, TN USA; 405 Medical College of Georgia, Augusta, GA USA; 406 Meharry Medical College School of Medicine, Nashville, TN USA; 407 Meharry Medical College, Nashville, TN USA; 408 Medical College of Georgia, Augusta, GA USA; 409 Meharry Medical College School of Medicine, Nashville, TN USA; 410 National Cancer Institute-Frederick, Frederick, MD USA; 411 CIP, Center for Cancer Research, BSP, Leidos Biomed Research Inc, National Cancer Institute-Frederick, Frederick, MD USA; 412 Beckman Research Institute/City of Hope, Monrovia, CA USA; 413 City of Hope, Duarte, CA USA; 414 University of Texas MD Anderson Cancer Center, Houston, TX USA; 415 University of Texas MD Anderson Cancer Center, Houston, TX USA; 416 Immune Design, Seattle, WA USA; 417 Seppic, Puteaux, Ile-de-France France; 418 Seppic Inc., Fairfield, NJ USA; 419 University of Birmingham, Birmingham, England UK; 420 Stanford University, Stanford, CA USA; 421 University of Virginia, Charlottesville, VA USA; 422 Massachusetts General Hospital Cancer Center, Boston, MA USA; 423 Memorial Sloan Kettering Cancer Center, New York, NY USA; 424 Ludwig Collaborative Laboratory, Memorial Sloan Kettering Cancer Center, New York, NY USA; 425 Department of Medicine, Memorial Sloan Kettering Cancer Center, New York, NY USA; 426 University of Virginia, Charlottesville, VA USA; 427 Department of Public Health Sciences, University of Virginia, Charlottesville, VA USA; 428 Division of Surgical Oncology, University of Virginia, Charlottesville, VA USA; 429 University of Texas MD Anderson Cancer Center, Houston, TX USA; 430 Department of Melanoma Medical Oncology, University of Texas MD Anderson Cancer Center, Houston, TX USA; 431 Nektar Therapeutics, San Francisco, CA USA; 432 University of Pennsylvania, Philadelphia, PA USA; 433 Department of Melanoma Medical Oncology, University of Texas MD Anderson Cancer Center, Houston, TX USA; 434 Texas Heart Institute, Houston, TX USA; 435 Department of Molecular Cardiology, Texas Heart Institute, Houston, TX USA; 436 7 Hills Pharma LLC, Houston, TX USA; 437 KU Leuven, Leuven, Vlaams-Brabant Belgium; 438 Caprion Biosciences Inc., Montreal, PQ Canada; 439 Transplant Institute, Sahlgrenska Academy at University of Gothenburg, Gothenburg, Sweden; 440 Department of Clinical Immunology, Gothenburg University, Gothenburg, Sweden; 441 Department of Immunology, Genetics and Pathology, Uppsala University, Uppsala, Sweden; 442 University of Michigan, Ann Arbor, MI USA; 443 Aduro Biotech, Inc., Berkeley, CA USA; 444 University of Michigan Cancer Center, Ann Arbor, MI USA; 445 University of Michigan, Ann Arbor, MI USA; 446 The Third Affiliated Hospital of Anhui Medical University, Heifei, Anhui People’s Republic of China; 447 Union Hospital, Tongji Medical College, Huazhong University of Science and Technology, Wuhan, Hubei People’s Republic of China; 448 Tianjin University Cancer Institute and Hospital, Tianjin, Hebei People’s Republic of China; 449 MedImmune Inc, Gaithersburg, MD USA; 450 University of Michigan Medical School, Ann Arbor, MI USA; 451 University of Michigan Medical Center, 1150 W. Medical Center Dr, Ann Arbor, MI USA; 452 University of Pennsylvania, Philadelphia, PA USA; 453 Inovio Pharmaceuticals, Plymouth Meeting, PA USA; 454 Inovio Pharmaceuticals, Philadelphia, PA USA; 455 Inovio Pharmaceuticals, San Diego, CA USA; 456 The Wistar Institute, Philadelphia, PA USA; 457 University of Wisconsin Carbone Cancer Center, Madison, WI USA; 458 University of Wisconsin, Madison, WI USA; 459 University of Virginia, Charlottesville, VA USA; 460 Department of Surgery, University of Virginia, Charlottesville, VA USA; 461 Division of Surgical Oncology, University of Virginia, Charlottesville, VA USA; 462 Mary Crowley Cancer Research Centers, Texas Oncology, P.A, Dallas, TX USA; 463 Texas Oncology, P.A, Baylor University Medical Center, Dallas, TX USA; 464 Gradalis, Inc, Carrollton, TX USA; 465 Gradalis, Inc., Dallas, TX USA; 466 Mary Crowley Cancer Research Centers, Gradalis, Inc, Dallas, TX USA; 467 Sapporo Hokuyu Hospital, Sapporo, Hokkaido Japan; 468 Brooke Army Medical Center, San Antonio, TX USA; 469 Womack Army Medical Center, Fayetteville, NC USA; 470 Department of Colon and Rectal Surgery, Washington University, St Louis, MO USA; 471 Madigan Army Medical Center, Tacoma, WA USA; 472 University of Texas MD Anderson Cancer Center, Houston, TX USA; 473 Cancer Vaccine Development Program, San Antonio, TX USA; 474 Genocea Biosciences, Cambridge, MA USA; 475 Memorial Sloan Kettering Cancer Center, New York, NY USA; 476 Ludwig Collaborative Laboratory, Memorial Sloan Kettering Cancer Center, New York, NY USA; 477 Department of Medicine, Memorial Sloan Kettering Cancer Center, New York, NY USA; 478 University of Pennsylvania, Philadelphia, PA USA; 479 Nanobiotix, Paris, Ile-de-France France; 480 Thomas Jefferson University, Philadelphia, PA USA; 481 The Jenner Institute, University of Oxford, Oxford, England UK; 482 Nuffield Department of Surgical Sciences, University of Oxford, Oxford, England UK; 483 Augusta University, Georgia Cancer Center, Augusta, GA USA; 484 Laboratory of Tumor Immunology and Biology, National Cancer Institute, National Institutes of Health, Bethesda, MD USA; 485 Etubics Corporation, Seattle, WA USA; 486 Genitourinary Malignancies Branch, Center for Cancer Research, National Cancer Institute, National Institutes of Health, Bethesda, MD USA; 487 Center for Cancer Research, National Cancer Institute, National Institutes of Health, Bethesda, MD USA; 488 University of Pennsylvania, Philadelphia, PA USA; 489 Taiho Pharmaceutical Co., Ltd, Tsukuba, Ibaraki Japan; 490 Department of Immunotechnology, Lund University, Lund, Skane Lan Sweden; 491 ENT Department, Lund University Hospital, Lund, Skane Lan Sweden; 492 NextCure, Beltsville, MD USA; 493 University of New Mexico Comprehensive Cancer Center, Beltsville, MD USA; 494 University of New Mexico Comprehensive Cancer Center, Albuquerque, NM USA; 495 University of New Mexico, Albuquerque, NM USA; 496 Stanford Department of Radiation Oncology, Stanford University School of Medicine, Stanford, CA USA; 497 Department of Pathology, University of California, San Diego, La Jolla, CA USA; 498 Georgetown Lombardi Comprehensive Cancer Center, Washington, DC USA; 499 George Mason University, Manassas, VA USA; 500 OracleBio, Newhouse, Scotland UK; 501 University of Texas MD Anderson Cancer Center, Houston, TX USA; 502 Yale Medical Oncology, New Haven, CT USA; 503 Nektar Therapeutics, San Francisco, CA USA; 504 Johns Hopkins University, Baltimore, MD USA; 505 Department of Surgery, University of Pittsburgh, Pittsburgh, PA USA; 506 University of Pittsburgh, Pittsburgh, PA USA; 507 University of Texas Health Science Center at San Antonio, San Antonio, TX USA; 508 Roswell Park Cancer Institute, Buffalo, NY USA; 509 Department of Immunology, University of Pittsburgh, Pittsburgh, PA USA; 510 Department of Immunology and Microbiology, University of Colorado, Aurora, CO USA; 511 Department of Cardiothoracic Surgery, University of Colorado, Aurora, CO USA; 512 Department of Pathology, University of Colorado, Aurora, CO USA; 513 Department of Surgery, University of Colorado, Aurora, CO USA; 514 Department of Medicine, University of Colorado, Aurora, CO USA; 515 National Jewish Health, Denver, CO USA; 516 University of Pittsburgh, Pittsburgh, PA USA; 517 Biothera Pharmaceuticals Inc, Eagan, MN USA; 518 MedGenome Inc, Foster City, CA USA; 519 Fujian Provincial Cancer Hospital, Fuzhou, Fujian People’s Republic of China; 520 Cellecta, Inc, Mountain View, CA USA; 521 PerkinElmer, Hopkinton, MA USA; 522 University of Pittsburgh, Pittsburgh, PA USA; 523 Massachusetts General Hospital, Harvard Medical, Boston, MA USA; 524 Columbia University Medical Center, New York, NY USA; 525 Perkin Elmer, Hopkinton, MA USA; 526 Columbia University, New York, NY USA; 527 Columbia University College of Physicians and Surgeons, New York, NY USA; 528 Rutgers Cancer Institute of New Jersey, New Brunswick, NJ USA; 529 New York Presbyterian, Columbia University Medical Center, New York, NY USA; 530 Aduro Biotech, Berkeley, CA USA; 531 Johns Hopkins University School of Medicine, Baltimore, MD USA; 532 The University of Chicago, Chicago, IL USA; 533 Genomics Institute of the Novartis Research Foundation (GNF), San Diego, CA USA; 534 Novartis Institutes for BioMedical Research, Inc., Cambridge, MA USA; 535 Johns Hopkins University Cancer Center, Baltimore, MD USA; 536 University of Chicago Medical Center, Chicago, IL USA; 537 Tish Cancer Institute, Icahn School of Medicine at Mount Sinai, New York, NY USA; 538 Murray Edwards College and School of Clinical Medicine, University of Cambridge, Cambridge, England UK; 539 Duke University Medical Center, Durham, NC USA; 540 Lineberger Comprehensive Cancer Center, University of North Carolina, Chapel Hill, NC USA; 541 Stanford Research Institute, Menlo Park, CA USA; 542 Rijksuniversiteit Groningen, Amsterdam, Groningen Netherlands; 543 University of Pennsylvania, Philadelphia, PA USA; 544 Acerta Pharma, Bellevue, WA USA; 545 University of Washington, Seattle, WA USA; 546 Cynosure Clinical Research LLC, North Bend, WA USA; 547 Arcus Biosciences, Hayward, CA USA; 548 West Virginia University, Morgantown, WV USA; 549 The Ohio State University, Columbus, OH USA; 550 The Ohio State University Wexner Medical Center, Columbus, OH USA; 551 Queen’s University, Kingston, ON Canada; 552 National Institute of Oncology, Budapest, Hungary; 553 BioIncept, Cherry Hill, NJ USA; 554 Oregon Health & Science University, Portland, OR USA; 555 University of California, San Francisco School of Medicine, San Francisco, CA USA; 556 Brigham and Women’s Hospital, Boston, MA USA; 557 Baylor Clinic, Houston, TX USA; 558 UCSF Helen Diller Family Comprehensive Cancer Center, San Francisco, CA USA; 559 Plexxikon Inc., Berkeley, CA USA; 560 University of Virginia, Charlottesville, VA USA; 561 Department of Public Health Sciences, University of Virginia, Charlottesville, VA USA; 562 Division of Surgical Oncology, University of Virginia, Charlottesville, VA USA; 563 Dana Farber Cancer Institute, Harvard University, Boston, MA USA; 564 Oregon Health & Science University, Portland, OR USA; 565 Janssen Pharmaceutical R&D, Spring House, PA USA; 566 Knight Cancer Institute, Howard Hughes Medical Institute, Portland, OR USA; 567 University of Pittsburgh Medical Center, Hillman Cancer Center, Pittsburgh, PA USA; 568 Department of Immunology, University of Pittsburgh, Pittsburgh, PA USA; 569 University of Pittsburgh, Pittsburgh, PA USA; 570 University of Pittsburgh Medical Center, Hillman Cancer Center, Pittsburgh, PA USA; 571 University of Pittsburgh, Pittsburgh, PA USA; 572 Oregon Health & Science University, Portland, OR USA; 573 University of Virginia, Charlottesville, VA USA; 574 Division of Surgical Oncology, University of Virginia, Charlottesville, VA USA; 575 Medical University of South Carolina, Charleston, SC USA; 576 University of Wisconsin School of Medicine and Public Health, Madison, WI USA; 577 Provenance Biopharmaceuticals Corp., Carlisle, MA USA; 578 Bristol-Myers Squibb, Princeton, NJ USA; 579 Apeiron Biologics, Vienna, Austria; 580 Rutgers University, New Brunswick, NJ USA; 581 Rutgers Cancer Institute of New Jersey, New Brunswick, NJ USA; 582 Rush University, Chicago, IL USA; 583 Helmholtz Zentrum München, Immunoanalytics- Tissue Control of Immunocytes, Munich, Bayern Germany; 584 Bioinformatics Core Unit, Biomedical Center, Ludwig-Maximilians-University, Planegg-Martinsried Munich, Munich, Bayern Germany; 585 Helmholtz Zentrum München, Institute of Experimental Genetics, Neuherberg, Bayern Germany; 586 Dr. Margarete Fischer-Bosch-Institute of Clinical Pharmacology, Stuttgart, Baden-Wurttemberg Germany; 587 Dr. Margarete Fischer-Bosch-Institute of Clinical Pharmacology, Stuttgart, University of Tuebingen, Stuttgart, Baden-Wurttemberg Germany; 588 Department of Clinical Pharmacology, University Hospital Tuebingen, Stuttgart, Baden-Wurttemberg Germany; 589 Helmholtz Zentrum München, Immunoanalytics-Tissue Control of Immunocytes, Munich, Bayern Germany; 590 Pulse Biosciences, Burlingame, CA USA; 591 Syndax Pharmaceuticals, Inc, Waltham, MA USA; 592 UCB Biopharma, Slough, England UK; 593 UCB Biopharma, Braine-l’Alleud, Brabant Wallon Belgium; 594 Surface Oncology, Cambridge, MA USA; 595 Arcus Biosciences, Hayward, CA USA; 596 Georgetown Lombardi Comprehensive Cancer Center, Washington, DC USA; 597 Washington Hospital Center, Washington, DC USA; 598 Lombardi Comprehensive Cancer Center, Washington, DC USA; 599 Washington University School of Medicine in St. Louis, St. Louis, MO USA; 600 The University of Texas Graduate School of Biomedical Sciences at Houston, Houston, TX USA; 601 Department of Immunology, University of Texas MD Anderson Cancer Center, Houston, TX USA; 602 Department of Microbiology, University of Iowa, Iowa City, IA USA; 603 University of Pittsburgh, Pittsburgh, PA USA; 604 University of Alabama, Birmingham, AL USA; 605 Sidra Medical and Research Center, Doha, Ad Dawhah Qatar; 606 University of Virginia, Charlottesville, VA USA; 607 Hunter Holmes McGuire Veterans Administration Medical Center, Richmond, VA USA; 608 Dorevitch Pathology, Heidelberg West, Victoria Australia; 609 Department of Surgery, University of Virginia, Charlottesville, VA USA; 610 Ludwig Institute for Cancer Research, Epalinges, Vaud Switzerland; 611 The University of Chicago, Chicago, IL USA; 612 Department of Pathology, The University of Chicago, Chicago, IL USA; 613 University of Chicago Medical Center, Chicago, IL USA; 614 Center for Biologics Evaluation and Research, Food and Drug Administration, Silver Spring, MD USA; 615 University of Chicago, Chicago, IL USA; 616 University of Pittsburgh, Pittsburgh, PA USA; 617 Magee-Womens Research Institute, Ovarian Cancer Center of Excellence, and Peritoneal/Ovarian Cancer Specialty Care Center, Hillman Cancer Center, University of Pittsburgh, Pittsburgh, PA USA; 618 Department of Surgery, University of Pittsburgh Cancer Institute, Pittsburgh, PA USA; 619 Department of Infectious Diseases and Microbiology, University of Pittsburgh, Pittsburgh, PA USA; 620 City of Hope, Duarte, CA USA

## Combinations: Immunotherapy/Immunotherapy

### P189 Rational combinations of intratumoral T cell and myeloid agonists mobilize abscopal responses in prostate cancer

#### Casey Ager^1^, Matthew Reilley^2^, Courtney Nicholas^1^, Todd Bartkowiak^1^, Ashvin Jaiswal^1^, Michael Curran^1^

##### ^1^Department of Immunology, University of Texas MD Anderson Cancer Center, Houston, TX, USA; ^2^Department of Cancer Medicine, University of Texas MD Anderson Cancer Center, Houston, TX, USA

###### **Correspondence:** Casey Ager (crager@mdanderson.org)

**Background**

Despite the success of checkpoint blockade immunotherapy in characteristically immunogenic cancers such as melanoma, these antibodies remain ineffective against poorly T cell-infiltrated malignancies including prostate cancer. Sensitizing these “cold” tumors to immunotherapy will require interventions which enhance tumor antigen presentation and T cell priming, while suppressing microenvironmental signals which constrain T cell expansion, survival, and effector function independent of coinhibitory signaling. We investigated whether intratumoral administration of either the STING agonist c-di-GMP (CDG) or dendritic cell (DC) growth factor Flt3-ligand can potentiate the therapeutic effects of T cell checkpoint modulation with αCTLA-4, αPD-1, and α4-1BB in a bilateral subcutaneous model of prostate adenocarcinoma. Additionally, we tested whether intratumoral delivery of low-dose checkpoint modulators with CDG at a single lesion can achieve abscopal control of distal lesions.

**Methods**

Male C57BL/6 mice were challenged subcutaneously on both flanks with TRAMP-C2 prostate adenocarcinoma, and treatment was administered intraperitoneally and/or intratumorally for 3 doses every 4 days, beginning on day 14 post-implantation for survival experiments or day 31 for flow analysis experiments.

**Results**

Intratumoral delivery of STING agonist CDG alone potently rejects all injected TRAMP-C2 tumors, but fails to generate systemic control of uninjected lesions. Systemic administration of αCTLA-4, αPD-1, and α4-1BB cures 40 % of mice with bilateral TRAMP-C2, and concurrent administration of CDG at one or both flanks enhances survival to 75 %. Similar effects are observed with intratumoral Flt3L, although administration at both flanks is required for full effect. Intratumoral low-dose αCTLA-4, αPD-1, and α4-1BB at a single flank induces abscopal effects in 20 % of mice, and concurrent administration of CDG enhances systemic immunity to cure up to 50 % of mice. We observe that the level of STING activation required to mediate rejection without inducing ulcerative toxicity is proportional to initial tumor size. Functionally, local STING activation complements intratumoral checkpoint modulation to reduce local MDSC infiltration, enhance CD8:Treg ratios, and downregulate the M2 macrophage marker CD206. In contrast, local Flt3L robustly enhances immune infiltration of injected and distal tumors, but therapeutic benefit is attenuated due to concomitant induction of FoxP3+ Treg.

**Conclusions**

Intratumoral STING activation via CDG or DC expansion with Flt3L potentiates the therapeutic effects of systemically-delivered αCTLA-4, αPD-1, and α4-1BB against multi-focal TRAMP-C2 prostate cancer. The abscopal potential of CDG alone is weak, in contrast to prior observations, but combining CDG with low-dose checkpoint blockade intratumorally can induce systemic immunity, suggesting an alternative approach for clinical implementation of combination immunotherapies at reduced doses.

### P190 Multi-genome reassortant dendritic cell-tropic vector platform (ZVex®-Multi) allows flexible co-expression of multiple antigens and immune modulators for optimal induction of anti-tumor CD8+ T cell responses

#### Tina C Albershardt, Anshika Bajaj, Jacob F Archer, Rebecca S Reeves, Lisa Y Ngo, Peter Berglund, Jan ter Meulen

##### Immune Design, Seattle, WA, USA

###### **Correspondence:** Tina C Albershardt (tina.albershardt@immunedesign.com)

**Background**

Induction of immune responses against multiple antigens expressed from conventional vector platforms is often ineffective for reasons not well understood. Common methods of expressing multiple antigens within a single vector construct include the use of fusion proteins, endoprotease cleavage sites, or internal ribosome entry sites. These methods often lead to decreased expression of antigens-of-interest and/or reduced induction of T cell responses against the encoded antigens. Circumventing these limitations, we have developed a novel production process for our integration-deficient, dendritic cell-targeting lentiviral vector platform, ZVex, enabling highly flexible and effective multigene delivery *in vivo*, making it possibly the most versatile vector platform in the industry.

**Methods**

Up to five vector genome plasmids, each encoding one full-length antigen or immuno-modulator, were mixed with four constant plasmids, each encoding vector particle proteins, prior to transfection of production cells. Due to the propensity of lentiviruses forming genomic reassortants, the resulting vector preparations hypothetically contain a mix of homozygous and heterozygous vector particles. qRT-PCR was used to determine total and antigen-specific titers of ZVex-Multi vectors, defined as vector genome counts. Mice were immunized with ZVex-Multi vectors or monozygous vectors expressing multiple antigens from the same backbone to compare immunogenicity via intracellular cytokine staining. Two tumor models were used to evaluate therapeutic efficacy: 1) a B16 melanoma model, where tumor cells were inoculated in the flank and measured 2–3 times per week; and 2) a metastatic CT26 colon carcinoma model, where tumor cells were inoculated intravenously, and lung nodules were enumerated 17–19 days post-tumor inoculation.

**Results**

Titrations by qRT-PCR of multiple ZVex-Multi vector lots demonstrated that production yields of ZVex-Multi expressing up to four different tumor-associated antigens (e.g., NY-ESO-1, MAGE-A3) and two immuno-modulators (e.g., IL-12, anti-CTLA-4 or anti-PD-L1) were highly reproducible. Compared to mice immunized with vectors expressing multiple antigens from the same backbone, mice immunized with ZVex-Multi vectors consistently developed T cells against all targeted TAAs and exhibited improved tumor growth control and survival.

**Conclusions**

ZVex-Multi is a next generation DC-tropic vector platform designed to overcome limitations of single-genome vector platforms with respect to efficient co-expression of any combination of desired genes. Unlike other vector platforms, ZVex-Multi eliminates multiple cloning steps modifying the vector backbone, which can often result in unpredictable expression patterns of coded gene products. Its versatility and agility makes ZVex-Multi potentially the best-in-class vector platform for co-expression of multiple tumor antigens and immuno-modulators for enhanced cancer immunotherapy against a broad range of tumors.

### P191 NK, T cells and IFN-gamma are required for the anti-tumor efficacy of combination-treatment with NKG2A and PD-1/PD-L1 checkpoint inhibitors in preclinical models

#### Caroline Denis^1^, Hormas Ghadially^2^, Thomas Arnoux^1^, Fabien Chanuc^1^, Nicolas Fuseri^1^, Robert W Wilkinson^2^, Nicolai Wagtmann^1^, Yannis Morel^1^, Pascale Andre^1^

##### ^1^Innate Pharma, Marseille, Provence-Alpes-Cote d'Azur, France; ^2^MedImmune, Cambridge, England, UK

###### **Correspondence:** Pascale Andre (pascale.andre@innate-pharma.fr)

**Background**

Monalizumab (IPH2201) is a first-in-class humanized IgG_4_ targeting NKG2A, which is expressed as heterodimer with CD94 on the surface of NK, γδT and tumor infiltrating CD8+ T cells. This inhibitory receptor binds to HLA-E in humans and to Qa-1^b^ in mice. HLA-E is frequently up-regulated on cancer cells, protecting from killing by NKG2A^+^ cells. Monalizumab blocks binding of CD94-NKG2A to HLA-E, reducing inhibitory signaling thereby enhancing NK and T cell responses. PD-1/PD-L1 inhibitors are successfully being used to treat patients with a wide variety of cancers. Combined blockade of NKG2A/HLA-E and PD-1/PD-L1 may be a promising strategy to better fight cancer by activating both the adaptive and innate immune systems.

**Methods**

To assess the effect of combined blockade of NKG2A/HLA-E and PD-1/PD-L1 *in vivo*, anti-mouse NKG2A and PD-1 antibodies were used in mice engrafted with A20 mouse B lymphoma cell line. For *in vitro* assays, anti-PD-L1 antibody durvalumab, and monalizumab were tested in human PBMC staphylococcal enterotoxin b assays.

**Results**

When cultured *in vitro*, the A20 cells express ligands for PD-1 but not for NKG2A. Exposure to IFN-γ *in vitro*, or subcutaneous injection into mice, induced expression of Qa-1^b^, resulting in a tumor model co-expressing PD-L1 and Qa-1^b^. Monotherapy with PD-1 or NKG2A blockers resulted in moderate anti-tumor efficacy while treatment with combination of NKG2A and PD-1 blockers resulted in a significantly higher anti-tumor immunity, and an increased rate of complete tumor regression. Depletion of either NK, or CD8+ T cells, or IFN-γ was enough to abrogate the efficacy of PD-1 and NKG2A blockade, indicating that both of these effector populations contribute to the efficacy of the combination treatment. To further explore this possibility and to assess the potential therapeutic relevance in humans, well-validated PBMC-based assays were used which showed that blocking both axes with a combination of durvalumab and monalizumab led to increased production of cytokines by both T and NK cells. Furthermore, the magnitude of the increase in cytokine secretion was dependent on the production of high levels of IFN-γ. Since IFN-γ is known to induce HLA-E this suggests that blockade of NKG2A could have a beneficial role in activation of immune cells through the combined blockade of PD-1/PD-L1.

**Conclusions**

Together, these data indicate that blocking NKG2A in conjunction with PD-1/PD-L1 checkpoint inhibitors provides increased anti-tumor efficacy mediated by IFN-γ and support the rationale for assessing this combination in clinical trials.

### P192 Pharmacokinetics and immunogenicity of pembrolizumab when given in combination with ipilimumab: data from KEYNOTE-029

#### Michael B Atkins^1^, Matteo S Carlino^2^, Antoni Ribas^3^, John A Thompson^4^, Toni K Choueiri^5^, F Stephen Hodi^5^, Wen-Jen Hwu^6^, David F McDermott^7^, Victoria Atkinson^8^, Jonathan S Cebon^9^, Bernie Fitzharris^10^, Michael B Jameson^11^, Catriona McNeil^12^, Andrew G Hill^13^, Eric Mangin^14^, Malidi Ahamadi^14^, Marianne van Vugt^15^, Mariëlle van Zutphen^15^, Nageatte Ibrahim^14^, Georgina V Long^16^

##### ^1^Georgetown-Lombardi Comprehensive Cancer Center, Washington, DC, USA; ^2^Westmead and Blacktown Hospitals, Melanoma Institute Australia, and the University of Sydney, Westmead, New South Wales, Australia; ^3^University of California, Los Angeles, CA, USA; ^4^University of Washington, Seattle, WA, USA; ^5^Dana-Farber Cancer Institute/Brigham and Women’s Hospital, Harvard University, Boston, MA, USA; ^6^University of Texas MD Anderson Cancer Center, Houston, TX, USA; ^7^Beth Israel Deaconess Medical Center, Boston, MA, USA; ^8^Gallipoli Medical Research Foundation, Greenslopes Private Hospital, and the University of Queensland, Greenslopes, Queensland, Australia; ^9^Olivia Newton-John Cancer Research Institute, Heidelberg, Victoria, Australia; ^10^Canterbury District Health Board, Christchurch Hospital, Christchurch, New Zealand; ^11^Waikato Hospital Regional Cancer Centre, Hamilton, New Zealand; ^12^Royal Prince Alfred Hospital, Melanoma Institute Australia, the University of Sydney, and Chris O’Brien Lifehouse, Camperdown, New South Wales, Australia; ^13^Tasman Oncology Research, Southport Gold Coast, Queensland, Australia; ^14^Merck & Co., Inc., Kenilworth, NJ, USA; ^15^Quantitative Solutions, a Certara company, Oss, Netherlands; ^16^Melanoma Institute Australia, the University of Sydney, Mater Hospital, and Royal North Shore Hospital, Wollstonecraft, New South Wales, Australia

###### **Correspondence:** Michael B Atkins (mba41@georgetown.edu)

**Background**

The pharmacokinetics of pembrolizumab given as monotherapy are well characterized. Consistent with other monoclonal antibodies, pembrolizumab has low clearance (0.202 L/day), limited central (3.53 L) and peripheral (3.85 L) volume of distribution, and low variability in the central volume of distribution (19 % coefficient of variation). Pembrolizumab monotherapy has low immunogenicity potential, with an observed incidence of treatment-emergent anti-drug antibodies (ADA) of < 1 %. We present data on the pharmacokinetics and immunogenicity of pembrolizumab when given in combination with ipilimumab in the phase I KEYNOTE-029 study.

**Methods**

KEYNOTE-029 included 2 cohorts that assessed the safety and antitumor activity of pembrolizumab plus ipilimumab: a safety run-in that included patients with advanced melanoma or renal cell carcinoma (RCC) (N = 22) and a melanoma expansion cohort (N = 153). In both cohorts, patients received 4 doses of pembrolizumab 2 mg/kg plus ipilimumab 1 mg/kg Q3W followed by pembrolizumab 2 mg/kg Q3W for up to 2 years. Pembrolizumab serum concentration was quantified with an electrochemiluminescence-based immunoassay (lower limit of quantitation, 10 ng/mL). A validated bridging electrochemiluminescence immunoassay using a standard 3-tiered approach (drug tolerance level, 124 μg/mL) was used to detect ADA in serum.

**Results**

**Fig. 1 (abstract P192).**
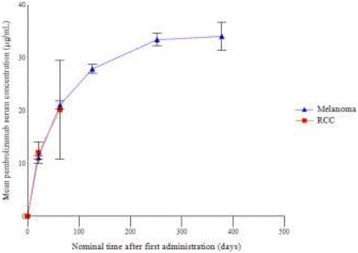
Arithmetic mean (SE) predose serum concentration-time profile of pembrolizumab following multiple doses of pembrolizumab plus ipilimumab (linear-linear scale)

**Fig. 2 (abstract P192).**
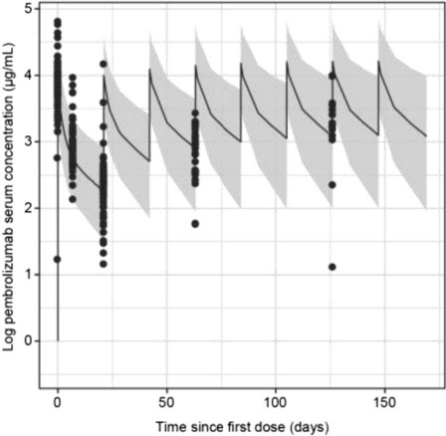
Observed pembrolizumab serum concentrations from patients with melanoma treated with pembrolizumab plus ipilimumab in relation to the predicted concentration interval (gray) for pembrolizumab 2 mg/kg Q3W monotherapy (log scale)

Across cohorts, 175 patients received pembrolizumab plus ipilimumab: 165 with melanoma and 10 with RCC. At least 1 evaluable sample for pharmacokinetic assessment was available for all 10 patients with RCC and 162 patients with melanoma. The predose serum concentration versus time profiles for pembrolizumab were similar in patients with RCC and melanoma (Fig. 1). Observed serum concentrations were within the range predicted for pembrolizumab 2 mg/kg Q3W given as monotherapy (Fig. 2). Of the 160 patients with melanoma who provided postdose ADA samples, 156 (97.5 %) were negative, 2 (1.3 %) were inconclusive, and 2 (1.3 %) were positive for treatment-emergent ADA. Best overall response in the ADA-positive patients was stable disease in one and progressive disease in the other. No patient with RCC had treatment-emergent ADA.

**Conclusions**

The addition of ipilimumab does not appear to impact pembrolizumab serum concentration or increase the risk of developing ADA in patients with advanced melanoma or RCC.

**Trial Registration**

ClinicalTrials.gov identifier NCT02089685.

### P193 Establishing a model for successful immunotherapy with T-Vec combined with BRAF inhibition and anti-PD-1 in genetically engineered murine melanoma

#### Robyn Gartrell^1^, Zoe Blake^1^, Ines Simoes^2^, Yichun Fu^1^, Takuro Saito^3^, Yingzhi Qian^1^, Yan Lu^1^, Yvonne M Saenger^4^

##### ^1^Columbia University Medical Center, New York, NY, USA; ^2^Institut d'Investigacions Biomediques August Pi i Sunyer, Barcelona, Catalonia, Spain; ^3^Icahn School of Medicine at Mount Sinai, New York, NY, USA; ^4^New York Presbyterian/Columbia University Medical Center, New York, NY, USA

###### **Correspondence:** Zoe Blake (zb2161@cumc.columbia.edu)

**Background**

Talimogene laherparepvec (T-Vec) is the first oncolytic virus to be U.S. Food and Drug Administration (FDA) approved for the treatment of cancer. T-Vec, a modified herpes simplex type I (HSV I) virus, has two proposed mechanisms of action: direct cell lysis and immune activation. Combination immunotherapy using T-Vec and checkpoint blockade has shown promise in clinical trials. In preliminary work, our laboratory has shown that T-Vec causes up-regulation of programmed cell death protein 1 (PD-1) on infiltrating T cells in mice, suggesting potential synergy of T-Vec and anti-PD-1 (αPD-1).

**Methods**

In a temporally and spatially regulated murine model of BRAF^CA^ PTEN^−/−^ spontaneous melanoma [1], tumors are induced on right flank. When tumors reach >5 mm in diameter, mice are randomized into 6 treatment groups comparing combinations of BRAF inhibition (BRAFi), αPD-1, and T–Vec (Table 1). Tumor growth is measured twice a week until end of study. Flow cytometry is performed on tumor, lymph node, and spleen to assess immune microenvironment.

**Results**

**Fig. 3 (abstract P193).**
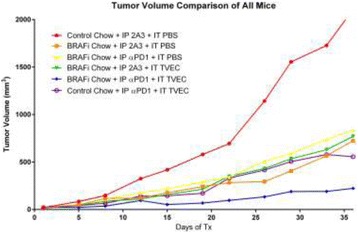
Tumor volume comparison of all mice

**Fig. 4 (abstract P193).**
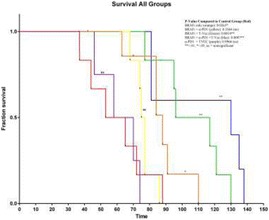
Survival comparison of treatment groups

**Fig. 5 (abstract P193).**
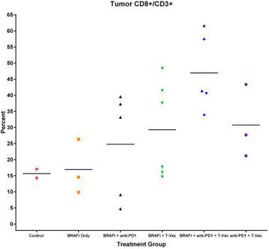
Flow cytometry data of CD8+ cells per CD3+ cell populations

**Fig. 6 (abstract P193).**
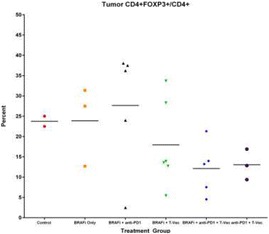
Flow cytometry data of CD4+/FOXP3+ cells per CD4+ cell populations

Mean tumor volume and survival was plotted to compare groups (Figs. 3 and 4). Mice treated with triple combination have decreased tumor growth. Mice treated with combination T-Vec + BRAFi with or without αPD-1 have longer survival compared to mice treated with control or single drug arms. Flow cytometry shows increase in percent CD3+/CD45+ cells in tumors of mice treated with combination αPD-1 + T-Vec compared to the control and single drug arms. Percent CD8+/CD3+ cells in tumors treated with immunotherapy appears to be increased compared to the control and BRAFi only group (Fig. 5). Additionally, percent of FOXP3+/CD4+ cells in tumors appears to be decreased in groups receiving T-Vec (Fig. 6) while no change in FOXP3+/CD4+ populations was observed in tumors from groups receiving αPD-1 without T-Vec or in draining lymph node or spleen.

**Conclusions**

Initial findings show that combination therapy of BRAFi + αPD-1 + T-Vec is more effective than any single treatment. Combination immunotherapy increases infiltration of T cells into tumor. Furthermore, oncolytic virus appears to decrease regulatory T cells infiltrating tumor. This study is ongoing and further analysis will continue as we further evaluate the immune microenvironment using flow cytometry and immunohistochemistry.

**Acknowledgements**

The study was funded by the Melanoma Research Alliance and Amgen (Amgen-CUMC-MRA Established Investigator Academic-Industry Partnership Award). **Reference**

1. Dankort, Curley, Cartlidge, *et al.*: **Braf(V600E) cooperates with Pten loss to induce metastatic melanoma.** *Nature Genetics* 2009, **41**:544–552.

**Table 1 (abstract P193).** Treatment groups

Group	Treatment
Group 1 (Red)	Control Chow + IP 2A3 + IT PBS
Group 2 (Orange)	BRAFi Chow + IP 2A3 + IT PBS
Group 3 (Yellow)	BRAFi Chow + Ip α-PD1 + IT PBS
Group 4 (Green)	BRAFi Chow + IP 2A3 + IT T-Vec
Group 5 (Blue)	BRAFi Chow + IP α-PD1 + IT T-Vec
Group 6 (Purple)	Control Chow + IP α=PD1 + IT T-Vec

*IP* intraperitoneal, *IT* intratumoral, *BRAFi* brief inhibiotor, *α-PD1* anti programmed cell death 1, *T-Vec* talimogene Leherparepvec

### P194 Phosphatidylserine targeting antibody in combination with checkpoint blockade and tumor radiation therapy promotes anti-cancer activity in mouse melanoma

#### Sadna Budhu^1^, Olivier De Henau^1^, Roberta Zappasodi^1^, Kyle Schlunegger^2^, Bruce Freimark^2^, Jeff Hutchins^2^, Christopher A Barker^1^, Jedd D Wolchok^1^, Taha Merghoub^1^

##### ^1^Memorial Sloan Kettering Cancer Center, New York, NY, USA; ^2^Peregrine Pharmaceuticals, Inc., Tustin, CA, USA

###### **Correspondence:** Sadna Budhu (budhus@mskcc.org)

**Background**

Phosphatidylserine (PS) is a phospholipid that is exposed on the surface of apoptotic cells, some tumor cells and tumor endothelium. PS has been shown to promote anti-inflammatory and immunosuppressive signals in the tumor microenvironment. Antibodies that target PS have been shown to reactivate anti-tumor immunity by repolarizing tumor associated macrophages to a M1-like phenotype, reducing the number of MDSCs in tumors and promote the maturation of dendritic cells into functional APCs. In a B16 melanoma model, targeting PS in combination with immune checkpoint blockade has been shown to have a significantly greater anti-cancer effect than either agent alone. This combination was shown to enhance CD4+ and CD8+ T cell infiltration and activation in the tumors of treated animals. Radiation therapy is an effective focal treatment of primary solid tumors, but is less effective in treating metastatic solid tumors as a monotherapy. There is evidence that radiation induces immunogenic tumor cell death and enhances tumor-specific T cell infiltration in irradiated tumors. In addition, the abscopal effect, a phenomenon in which tumor regression occurs outside the site of radiation therapy, has been observed in both preclinical and clinical trials with the combination of radiation therapy and immunotherapy.

**Methods**

We examined the effects of combining tumor radiation therapy with an antibody that targets PS (1 N11) and an immune checkpoint blockade (anti-PD-1) using the mouse B16 melanoma model. Tumor surface area and overall survival of mice were used to determine efficacy of the combinations.

**Results**

We examined the expression of PS on immune cells infiltrating B16 melanomas. CD11b + myeloid cells expressed the highest levels of PS on their surface whereas T cells and B16 tumor cells express little to no PS. These data suggest that targeting PS in B16 melanoma would induce a pro-inflammatory myeloid tumor microenvironment. We hypothesize that therapies that induce apoptotic cell death on tumor cells would enhance the activity of PS-targeting antibodies. We therefore examined the effects of combining a PS-targeting antibody with local tumor radiation. We found that the PS-targeting antibody synergizes with both anti-PD-1 and radiation therapy to improve anti-cancer activity and overall survival. In addition, the triple combination of the PS-targeting antibody, tumor radiation and anti-PD-1 treatment displayed even greater anti-cancer and survival benefit.

**Conclusions**

This finding highlights the potential of combining these three agents to improve outcome in patients with advanced-stage melanoma and may inform the design of future clinical trials with PS targeting in melanoma and other cancers.

### P195 A novel anti-human LAG-3 antibody in combination with anti-human PD-1 (REGN2810) shows enhanced anti-tumor activity in PD-1 x LAG-3 dual-humanized mice and favorable pharmacokinetic and safety profiles in cynomolgus monkeys

#### Elena Burova, Omaira Allbritton, Peter Hong, Jie Dai, Jerry Pei, Matt Liu, Joel Kantrowitz, Venus Lai, William Poueymirou, Douglas MacDonald, Ella Ioffe, Markus Mohrs, William Olson, Gavin Thurston

##### Regeneron, Tarrytown, NY, USA

###### **Correspondence:** Elena Burova (elena.burova@regeneron.com)

**Background**

In the tumor microenvironment, T cell inhibitory checkpoint receptors trigger signals that suppress T cell effector function, resulting in tumor immune evasion. Clinical antibodies blocking one of these receptors, PD-1, yield positive responses in multiple cancers; however, their efficacy is limited. Simultaneously targeting more than one inhibitory checkpoint receptor has emerged as a promising therapeutic strategy. In support of this concept, mice deficient in PD-1 and LAG-3, an inhibitory checkpoint receptor often co-expressed with PD-1 in the tumor microenvironment, exhibit enhanced anti-tumor activity. Here, we demonstrate increased anti-tumor efficacy of a combined anti–human PD-1 (hPD-1) and anti–human LAG-3 (hLAG-3) therapy using fully human monoclonal antibodies in dual humanized PD-1 x LAG-3 mice. The pharmacokinetics and toxicology of the novel anti-hLAG-3 antibody were assessed in non-human primates to support clinical development.

**Methods**

REGN2810, a high affinity anti-hPD-1 monoclonal antibody that blocks PD-1 interaction with PD-L1 and PD-L2, and a novel high affinity monoclonal anti–hLAG-3 antibody, which blocks the LAG-3/MHC II interaction were generated. Dual humanized PD-1 x LAG-3 mice were engineered by replacing the extracellular domains of mouse *Pdcd1* and *Lag3* with the corresponding regions of *hPD-1* and *hLAG-3* and were used for testing antibody efficacy in a MC38.ova syngeneic tumor model. Expression of humanized PD-1 and LAG-3 were analyzed by flow cytometry. Binding of hLAG-3 to mouse MHC II was confirmed with a cell adhesion assay, and binding of hPD-1 to mouse PD-L1 was confirmed using surface plasmon resonance. The pharmacokinetics of anti-hLAG-3 antibody following a single i.v. dose, and the safety profile in a 4-week weekly i.v. dose regimen of up to 50 mg/kg/dose, were determined in cynomolgus monkeys.

**Results**

Treatment of MC38.ova tumor-bearing humanized mice with a combination of anti-hPD-1 and anti-hLAG-3 antibodies triggered activation of intratumoral and peripheral T cells. Importantly, the combination treatment exhibited an additive, dose dependent anti-tumor effect compared to the respective monotherapies. Anti-hLAG-3 antibody pharmacokinetics in cynomolgus monkeys followed a standard mean concentration-time profile characterized by an initial brief distribution phase and a linear beta elimination phase. Exposure to anti-hLAG-3 increased in a dose-proportional manner, with elimination half-lives ranging from 10.8 to 11.5 days. Anti-hLAG-3 antibody was well tolerated, and no-observed-adverse-effect level (NOAEL) could be established up to 50 mg/kg.

**Conclusions**

Preclinical anti-tumor efficacy of combined REGN2810 and anti-hLAG-3 antibody treatment, together with favorable pharmacokinetic and safety data for anti-hLAG-3 antibody in cynomolgus monkeys, support clinical development of this cancer combination immunotherapy.

### P196 Combination of PD-L1 blockade with oncolytic vaccines re-shapes the functional state of tumor infiltrating lymphocytes

#### Cristian Capasso^1^, Federica Frascaro^2^, Sara Carpi^3^, Siri Tähtinen^1^, Sara Feola^4^, Manlio Fusciello^1^, Karita Peltonen^1^, Beatriz Martins^1^, Madeleine Sjöberg^1^, Sari Pesonen^5^, Tuuli Ranki^5^, Lukasz Kyruk^1^, Erkko Ylösmäki^1^, Vincenzo Cerullo^1^

##### ^1^University of Helsinki, Helsinki, Uusimaa, Finland; ^2^University of Siena, Supersano (LE), Puglia, Italy; ^3^University of Pisa, Pisa, Toscana, Italy; ^4^University of Napoli Federico II, Helsinki, Uusimaa, Finland; ^5^PeptiCRAd Oy, Helsinki, Uusimaa, Finland

###### **Correspondence:** Cristian Capasso (cristian.capasso@helsinki.fi)

**Background**

The immunological escape of tumors represents one of the main obstacles to the treatment of malignancies. The blockade of PD-1 or CTLA-4 receptors represented a milestone in the history of immunotherapy. However, immune checkpoint inhibitors seem to be effective in specific cohorts of patients. It has been proposed that their efficacy relies on the presence of an immunological response. Thus, we hypothesized that disruption of the PD-L1/PD-1 axis would synergize with our oncolytic vaccine platform PeptiCRAd.

**Methods**

We used murine B16OVA *in vivo* tumor models and flow cytometry analysis to investigate the immunological background.

**Results**

**Fig. 7 (abstract P196).**
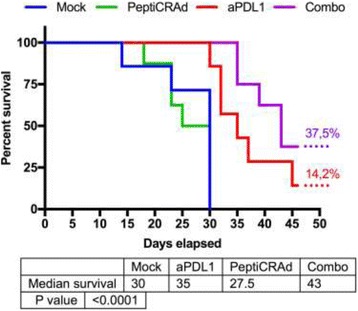
Survival of C57 mice bearing B16OVA tumors and treated on day 6 post-implantation with either PBS, PDL1 blockade, OVA-targeting PeptiCRAd or the combination of PDL1-blockade and OVA-PeptiCRAd.

First, we found that high-burden B16OVA tumors were refractory to combination immunotherapy. However, with a more aggressive schedule, tumors with a lower burden were more susceptible to the combination of PeptiCRAd and PD-L1 blockade. The therapy significantly increased the median survival of mice (Fig. 7). Interestingly, the reduced growth of contralaterally injected B16F10 cells suggested the presence of a long lasting immunological memory also against non-targeted antigens. Concerning the functional state of tumor infiltrating lymphocytes (TILs), we found that all the immune therapies would enhance the percentage of activated (PD-1^pos^ TIM-3^neg^) T lymphocytes and reduce the amount of exhausted (PD-1^pos^ TIM-3^pos^) cells compared to placebo. As expected, we found that PeptiCRAd monotherapy could increase the number of antigen specific CD8+ T cells compared to other treatments. However, only the combination with PD-L1 blockade could significantly increase the ratio between activated and exhausted pentamer positive cells (p = 0.0058), suggesting that by disrupting the PD-1/PD-L1 axis we could decrease the amount of dysfunctional antigen specific T cells. We observed that the anatomical location deeply influenced the state of CD4+ and CD8+ T lymphocytes. In fact, TIM-3 expression was increased by 2 fold on TILs compared to splenic and lymphoid T cells. In the CD8+ compartment, the expression of PD-1 on the surface seemed to be restricted to the tumor micro-environment, while CD4+ T cells had a high expression of PD-1 also in lymphoid organs. Interestingly, we found that the levels of PD-1 were significantly higher on CD8+ T cells than on CD4+ T cells into the tumor microenvironment (p < 0.0001).

**Conclusions**

In conclusion, we demonstrated that the efficacy of immune checkpoint inhibitors might be strongly enhanced by their combination with cancer vaccines. PeptiCRAd was able to increase the number of antigen-specific T cells and PD-L1 blockade prevented their exhaustion, resulting in long-lasting immunological memory and increased median survival.

### P197 In vitro evaluation of immunotherapy protocols through a label-free impedance-based technology allows dynamic monitoring of immune response and reagent efficacy

#### Fabio Cerignoli, Biao Xi, Garret Guenther, Naichen Yu, Lincoln Muir, Leyna Zhao, Yama Abassi

##### ACEA Biosciences Inc., San Diego, CA, USA

###### **Correspondence:** Fabio Cerignoli (fcerignoli@aceabio.com)

**Background**

*In vitro* characterization of reagent efficacy in the context of cancer immunotherapy is a necessary step before moving to more expensive animal models and clinical studies. However, current *in vitro* assays like Chromium-51, ATP-based luminescence or flow cytometry are either difficult to implement in high throughput environments or are mainly based on endpoint methodologies that are unable to capture the full dynamic of the immune response. Here, we present the adaptation of an impedance-based platform to monitor cytotoxic activity of immune cells activated trough different means.

**Methods**

Impedance technology detects cell death and proliferation of adherent cells by measuring changes in conductance of microelectrodes embedded in 96 and 384-wells cell culture plates. We utilized adherent and B cell leukemia/lymphoma cell lines as well as primary tumor cells as *in vitro* models for immunotherapy reagent evaluation. We seeded the cells on electrodes coated 96-well plates and monitored cell adhesion and proliferation for 24 hours. The following day effector cells were added at multiple effector:target ratios in presence of BiTEs antibodies and/or anti PD-1/PD-L1 antibodies. Impedance signal was monitored for up to seven days. Control wells were set up with effector cells only or with target plus effector cells but without antibodies. We adapted such adhesion-based technology to monitor non-adherent B-leukemia/lymphoma cells, by developing a strategy where the wells are coated with an anti-CD40 antibody. The coating allows specific adhesion and retention of B cells and measurement of changes in impedance that are proportional to cell number.

**Results**

Using increasing concentrations of EpCAM/CD3 BiTE, we demonstrated the suitability of an impedance-based approach to quantitatively monitor the efficacy of immune cells-mediated cancer cell killing both under different effector:target ratios and antibody concentrations. Combination treatments with checkpoint reduced timing and increased amount of killed cancer cells. Similar results were also obtained with engineered CAR-T cells against CD19 or NK cell lines, demonstrating specific killing of tumor B cells at very low effector:target ratios. The results were also confirmed by flow cytometry.

**Conclusions**

Overall, our results demonstrate the value of an impedance-based approach in measuring the cytotoxic response across the temporal scale, an aspect that is otherwise very difficult to assess with more canonical end point assays. Furthermore, the availability of 384-well format and minimal sample handling place the technology in an ideal spot for applications in large reagent validation screening or personalized medicine, like therapeutic protocol validation directly on patient samples.

### P198 Tumor necrosis factor alpha and interleukin-2 expressing adenovirus plus PD-1 blockade as a boost for T cell therapy in the context of solid tumor therapies

#### Víctor Cervera-Carrascón^1^, Mikko Siurala^1^, João Santos^1^, Riikka Havunen^2^, Suvi Parviainen^1^, Akseli Hemminki^1^

##### ^1^TILT Biotherapeutics, Helsinki, Uusimaa, Finland; ^2^University of Helsinki, Helsinki, Uusimaa, Finland

###### **Correspondence:** Víctor Cervera-Carrascón (victor@tiltbio.com)

**Background**

Because of the immunosuppressive tumor microenvironment, the immune system is unable to develop effective responses against tumor cells. This phenomenon also acts against the effectiveness of adoptive T cell therapy. In order to overcome this situation in the tumor, an attractive therapeutic combination is the combination of oncolytic viruses and immune checkpoint inhibitors. In this case, besides the last two therapies mentioned above, combinations with T cell therapy were also included. The virus used was engineered to express tumor necrosis factor α (TNFα) and interleukin (IL)-2, two cytokines that will boost the immunogenicity of the virus and thus its antitumor properties. On the other hand, the use of anti-PD-1 will avoid exhaustion on tumor infiltrating T cells and hence remove the barriers that could dampen the desired immune response against the tumor.

**Methods**

In the study of the antitumor effect of this three armed treatment we used an *in vivo* model of subcutaneous B16-OVA melanoma-bearing mice. Two experiments were carried out; the first one (n = 47) to establish the differences between the triple, double, and single armed combination therapies and the second experiment (n = 84) was focused on study the differences between the groups that showed the best outcomes in the first one and also optimize viral and anti-PD-1 administration regimes.

**Results**

Preliminary results show a statistically significant positive effect coming out from the combination of virus therapy and immune checkpoint blockade with regard to both tumor progression and overall survival, with up to 43 % complete tumor regression achieved in some of the groups after 96 days post treatment. On the other hand, the effect of adoptive cell therapy in this combination is not completely clear. More results will be presented after analyzing biological samples collected during both experiments.

**Conclusions**

Preclinical studies are a key step to detect which combinations are more suitable for success in human trials. In this study we developed a rationale for the combination relying on two concepts: to make silent tumors more visible to the immune system and to counter immunosuppressive mechanisms to unleash the full potential of T cells against the tumor, rendering in a modification of the tumor microenvironment that makes it more susceptible for T cell mediated killing. According to the results displayed from these experiments, the combination of this genetically modified adenovirus and PD-1 blockade is an efficient combination to be considered for future application in humans.

### P199 IMM-101 primes for increased complete responses following checkpoint inhibitors in metastatic melanoma; 3 case reports

#### Angus Dalgleish^1^, Satvinder Mudan^2^

##### ^1^St George's University of London, London, UK; ^2^The Royal Marsden Hospital and Imperial College London, London, UK

###### **Correspondence:** Angus Dalgleish (dalgleis@sgul.ac.uk)

**Background**

IMM-101, a heat-killed borate-buffered whole cell product of Mycobacterium obuense has been shown to enhance cell mediated cytokine responses and innate immune responses involving NK and gamma delta cells [1]. Complete responses (CR) in patients with melanoma lung metastases demonstrated. Follow up of original publication [2] has shown a 30 % 5-year survival. Combined with gemcitabine in metastatic pancreatic cancer a significant survival advantage over gemcitabine monotherapy is seen [3].

**Methods**

We present 3 patients with metastatic melanoma, progressed after initial stabilisation with IMM-101, who showed CR after check point inhibitors (CPI) ipilimumab (n = 2), pembrolizumab (n = 1). Patient 1: 2006 46 M melanoma left forearm, BT 3.7 mm, 1 positive lymph node. Recurrent disease treated with surgery, Aldara and low dose IL-2. 2010 pulmonary mets, commenced IMM-101, no response (initial SD). 2011 given Ipilimumab. Patient 2: 2011 50 F axillary lump removed, melanoma (no primary). Concomitant mediastinal, lung, gastric and peritoneal deposits. Gastric surgery, decarbazine. Commenced IMM-101 with cyberknife to lung lesion. 2013 Small bowel obstruction from new disease. Started ipilimumab. Patient 3: 2014 79 M melanoma, left cheek, BT 2.4 mm. Regional lymph node recurrence, treated with a left neck dissection in April 2014. Developed paracardiac nodes, adrenal, lung and multiple large subcutaneous deposits. Commenced IMM-101 with initial shrinkage. However, new large subcutaneous lesions. Commenced pembrolizumab.

**Results**

Patient 1 - CR on Pet CT, maintained through 2016. Patient 2 - CR maintained for 2 years. Patient 3 - CR of subcutaneous deposits four days after first injection.

**Conclusions**

The CR rate to CPI’s is disappointing, < 1 % for Ipilimumab. PDL-1 expression is predictive for PD-1 responses and although CPI combinations are clearly needed, most are very toxic. IMM-101 is relatively free of toxicity, enhances PD-1 expression in pre-clinical models but may also prime tumour response to check point inhibitors by its action on macrophage function. Based on these observations, we speculate that IMM-101 primes for CPI’s and propose a trial priming with IMM-101, followed by anti-PD-1 antibodies.

**References**

1. Fowler D, *et al.*: **Mycobacteria activate γδ T-cell anti-tumour responses via cytokines from type 1 myeloid dendritic cells: a mechanism of action for cancer immunotherapy**. *CeII* 2012, **61(4)**:535–547.

2. Stebbing J, *et al.*: **An intra-patient placebo-controlled phase I trial to evaluate the safety and tolerability of intradermal IMM-101 in melanoma**. *Ann Oncol* 2012, **23(5)**:1314–1319.

3. Dalgleish, *et al.*: **Randomised open-label, phase II study of Gemcitabine with and without IMM-101 for advanced pancreatic cancer (IMAGE-1 Trial)**. *BJC* 2016, in press.

### P200 Immunological impact of checkpoint blockade on dendritic cell driven T cell responses: a cautionary tale

#### Mark DeBenedette, Ana Plachco, Alicia Gamble, Elizabeth W Grogan, John Krisko, Irina Tcherepanova, Charles Nicolette

##### Argos Therapeutics Inc., Durham, NC, USA

###### **Correspondence:** Mark DeBenedette (mdebenedette@argostherapeutics.com)

**Background**

AGS-003 is an individualized, autologous, tumor antigen-loaded, dendritic cell (DC) immunotherapy currently in phase III development for the treatment of metastatic renal cell carcinoma (mRCC) in combination with standard-of-care. Antibodies to PD-1 on activated T cells or PD-L1 expressed on APCs have now been approved for treatment of several cancer indications including RCC. While there is a strong mechanistic rationale for the potential synergy of these agents in combination, data supporting the importance of sequencing the administration of these agents are limited. Since the DC-based immunotherapy, AGS-003, expresses high levels of PD-L1, combinations with checkpoint blockade may remove a critical signal protecting DCs during the early CTL activation phase *in vivo*. Concurrent administration of checkpoint inhibitors with AGS-003 may, therefore, impede the proposed mechanism of action of AGS-003, which is the induction of tumor-specific CTL responses. Results derived from *in vitro* modeling of DCs inducing T cell responses can demonstrate how to better mobilize the immune system to overcome the immunosuppressive environment of cancer. Therefore, it was of interest to test anti-PD-1/anti-PD-L1 antibody therapy *in vitro* in combination with DCs representative of AGS-003, to observe the effects combination therapy would have on antigen-specific CTL proliferation and functional responses.

**Methods**

DCs derived from monocytes were co-electroporated with MART-1 RNA and CD40 ligand RNA to represent AGS-003 DC products. *In vitro* co-cultures were set up with autologous CTLs and MART-1/CD40L DCs in the presence of anti-PD-1 or anti-PD-L1 antibodies. In some instances, PD-1 expression was hyper expressed on CTLs by electroporating MART-1-specfic CTLs with PD-1 RNA. Subsequent expansion of MART-1-specific CTLs and multi-functional responses in the presence of checkpoint blockade were mapped using multi-color flow cytometry.

**Results**

Combination with anti-PD-1 antibody did not did not negatively affect the expansion of MART-1-specific CTL responses; however, if PD-1 was hyper-expressed on previously stimulated MART-1-specific CTLs responses were diminished. Anti-PD-1 antibody blocking restored CTL function in the presence of high levels of PD-1 expression. Interestingly, anti-PD-L1 antibody blocking resulted in suppression of early MART-1-specific CTL expansion and subsequent downstream effector function.

**Conclusions**

Our results suggest that the sequencing of AGS-003 therapy and checkpoint blockade is important to allow full CTL activation by the DCs prior to anti-PD-1/PD-L1 therapy. Moreover the high expression of PD-L1 on DCs may serve as a “don’t kill the messenger” signal, critical to prevent deletion of the DC prior to full signal delivery during early phases of CTL activation.

### P201 Targeting the PD-1/PD-L1 signaling pathway for the treatment of OS lung metastasis

#### Pooja Dhupkar, Ling Yu, Eugenie S Kleinerman, Nancy Gordon

##### University of Texas MD Anderson Cancer Center, Houston, TX, USA

###### **Correspondence:** Pooja Dhupkar (pmdhupkar@mdanderson.org)

**Background**

Osteosarcoma (OS) is a primary bone malignancy, commonly culminating into aggressive pulmonary metastasis. Despite chemotherapy advances, the 5-year survival of pulmonary metastatic OS remains 25-30 %. Immunotherapy is one of the promising novel approaches to target minimal residual and relapsed disease. The objective of this study is to determine if blocking the PD-1/PD-L1 immunosuppressive signaling pathway using a PD-1 checkpoint inhibitor will have an effect in OS lung metastasis. Anti-PD-1 and anti-PD-L1 antibodies have exhibited therapeutic benefit in melanoma, and non-small cell lung carcinoma. We hypothesize that disruption of the PD-1/PD-L1 signaling pathway using anti-PD-1 antibody has an effect against OS lung metastasis and improves overall survival.

**Methods**

Flow cytometry and western blotting were used to analyze PD-L1 expression in 7 different OS cell lines. Immunohistochemistry (IHC) analysis was used to determine PD-L1 expression in OS lung metastases from patients and mice. LM7 human OS mouse model was used to test the effect of blocking murine PD-1 in OS lung metastases. Therapeutic effect of anti-PD-1 treatment was measured by the number of macro and micro-metastases. IHC was used to measure cell proliferation (Ki-67), apoptosis (TUNEL) and cleaved-caspase 3 expression in addition to NK cells and macrophages infiltration. Western blotting was used to address the downstream components of the signaling pathway such as p-Stat3 and p-Erk1/2. The Simple PCI software was used to quantify the IHC data.

**Results**

Our studies revealed surface and total PD-L1 expression in five out of seven human OS cell lines. Primary and metastatic OS lung tumor samples from patients demonstrated membranous and cytoplasmic PD-L1 expression. Using a human OS mouse model we demonstrated therapeutic effect of anti-PD-1 therapy as the number of macro and micro-metastases decreased in the anti-PD-1 treated group as compared to the untreated. Anti-PD-1 treatment led to a significant increase in the number of NK cells and macrophages in the OS lung tumors suggesting these cells to have a potential therapeutic benefit against OS lung metastases. In addition, anti-PD-1 therapy caused a decrease in PD-L1 expression in the lung tumors, possibly due to a decrease in p-ERK1/2 and p-Stat3 expression.

**Conclusions**

We conclude that targeting the PD-1/PD-L1 axis could be used to treat OS lung metastasis. Therapeutic efficacy of anti-PD-1 may be due to an increased activity of NK cells and/or macrophages in the lung tumors and that inhibition of the p-Stat3/PD-L1 pathway may be the mechanism implicated in OS lung metastases after anti-PD-1 treatment.

### P202 Effect of the class I-HDAC inhibitor entinostat and the pan-HDAC inhibitor vorinostat on peripheral immune cell subsets

#### Italia Grenga, Lauren Lepone, Sofia Gameiro, Karin M Knudson, Massimo Fantini, Kwong Tsang, James Hodge, Renee Donahue, Jeffrey Schlom

##### Laboratory of Tumor Immunology and Biology, National Cancer Institute, National Institutes of Health, Bethesda, MD, USA

###### **Correspondence:** Renee Donahue (renee.donahue@nih.gov)

**Background**

Cancer immunotherapy requires effective recognition and elimination of tumor cells identified as non-self; however, tumors can evade host immune surveillance through multiple mechanisms, including epigenetic silencing of genes involved in antigen processing and immune recognition. Epigenetic therapy with histone deacetylase (HDAC) inhibitors has shown limited benefit as a monotherapy in patients with solid tumors; however, recent reports suggest the potential for synergy when combined with immunotherapy. Entinostat is a class I-HDAC inhibitor undergoing trials for the treatment of various cancers, while vorinostat is a pan-HDAC inhibitor approved in the United States for the treatment of cutaneous T cell lymphoma. The aim of this study was to extensively evaluate the effects of entinostat and vorinostat on human peripheral immune cell subsets in order to examine the potential for combination of HDAC inhibitors with cancer immunotherapy.

**Methods**

Peripheral blood mononuclear cells (PBMCs) from metastatic breast cancer patients (n = 7) were exposed *in vitro* for 48 hours to clinically relevant exposures of entinostat, vorinostat, or vehicle control. PBMCs were then analyzed by multicolor flow cytometry using 27 unique markers to identify 123 immune cell subsets, which included 9 classic cell types [CD4^+^ and CD8^+^ T cells, regulatory T cells (Treg), B cells, conventional dendritic cells (cDC), plasmacytoid dendritic cells (pDC), natural killer cells (NK), natural killer T cells (NKT), and myeloid derived suppressor cells (MDSC)], and 114 refined subsets relating to their maturation and function.

**Results**

Treatment with entinostat and vorinostat induced several notable alterations in peripheral immune cells, suggesting mainly immune activating properties. Exposure to entinostat increased the frequency of activated CD4^+^ T cells, activated mature NK cells, antigen presenting cells (cDCs), and highly immature MDSCs, as well as decreased total Tregs and those with a suppressive phenotype. Exposure to vorinostat induced fewer changes than entinostat, including increasing the frequency of activated CD4^+^ T cells, highly immature MDSCs, and NKT cells.

**Conclusions**

These findings show that while entinostat and vorinostat have overall immune activating properties, entinostat induced a greater number changes than vorinostat. This study supports the combination of HDAC inhibitors with immunotherapy, including therapeutic cancer vaccines and/or checkpoint inhibitors.

### P203 Shifting the balance of tumor-mediated immune suppression and augmenting immunotherapy with antibody blockade of semaphorin 4D to facilitate immune-mediated tumor rejection

#### Elizabeth Evans^1^, Holm Bussler^1^, Crystal Mallow^1^, Christine Reilly^1^, Sebold Torno^1^, Maria Scrivens^1^, Cathie Foster^1^, Alan Howell^1^, Leslie Balch^1^, Alyssa Knapp^1^, John E Leonard^1^, Mark Paris^1^, Terry Fisher^1^, Siwen Hu-Lieskovan^2^, Antoni Ribas^2^, Ernest Smith^1^, Maurice Zauderer^1^

##### ^1^Vaccinex, Rochester, NY, USA; ^2^University of California, Los Angeles, Los Angeles, CA, USA

###### **Correspondence:** Elizabeth Evans (eevans@vaccinex.com)

**Background**

We report a novel role for semaphorin 4D (SEMA4D, CD100) in modulating the tumor microenvironment (TME) to exclude activated antigen presenting cells and cytotoxic T lymphocytes so as to promote tumor growth. Antibody blockade reduces expansion of MDSC, shifts the balance of M1/M2, T effector/T regulatory cells and associated cytokines and chemokines, and augments tumor rejection with immune checkpoint inhibition.

**Methods**

Anti-SEMA4D antibodies were evaluated, alone and in combination with immune checkpoint antibodies. Immune response was characterized by immunohistochemistry, flow cytometry, functional assays, and cytokine, chemokine and gene expression analysis. Anti-tumor activity was evaluated in various preclinical models. A phase I trial for single agent VX15/2503 was completed.

**Results**

SEMA4D restricts migration of macrophages and promotes expansion of suppressive myeloid cells *in vitro*. Strong expression of SEMA4D at the invasive margins of actively growing tumors *in vivo* modulates the infiltration and polarization of leukocytes in the TME. Antibody neutralization facilitated recruitment of activated APCs and T lymphocytes into the TME in preclinical models. M-MDSCs were significantly reduced in both tumor and blood following treatment. This was accompanied by a significant shift towards increased Th1 cytokines and CTL-recruiting chemokines, with concurrent reduction in Treg-, MDSC-, and M2-macrophage promoting chemokines (CCL2, CXCL1, CXCL5). Accordingly, an increase in Teff:Treg ratio (3x, p < 0.005) and CTL activity (4x, p < 0.0001) was observed. NanoString gene expression analysis of on-treatment tumors confirms an increase in the gamma-inflammatory gene signature (Ribas, ASCO 2015), including significant increases in CXCL9, Gzmb, CCR5, Stat1, Lag3, Ptprc, Ciita, Pdcd1 (PD-1), and Itga1. These coordinated changes in the tumoral immune context are associated with durable tumor rejection and immunologic memory in preclinical colon, breast, and melanoma models. Importantly, anti-SEMA4D antibody can further enhance activity of immune checkpoint inhibitors and chemotherapy. Strikingly, the combination of anti-SEMA4D with anti-CTLA-4 acts synergistically, with maximal increase in survival (p < 0.01) and complete tumor regression in 100 % of mice, as compared to 22 % with monotherapy (p < 0.01). SEMA4D antibody treatment was well tolerated in nonclinical and clinical studies; including a phase I multiple ascending dose trial in patients with advanced refractory solid tumors. Patients with the longest duration of treatment, 48–55 weeks, included colorectal, breast, and a papillary thyroid patient, who had a partial response by RECIST.

**Conclusions**

Inhibition of SEMA4D represents a novel mechanism and therapeutic strategy to promote functional immune infiltration into the tumor and inhibit tumor progression. Phase Ib/IIa trials of combination therapy with immune checkpoint inhibition are planned.

### P204 Combination of a glycomimetic antagonist to E-selectin and CXCR4, GMI-1359, with an anti-PD-L1 antibody attenuates regulatory T cell infiltration and accelerates time to complete response in the murine CT26 tumor model

#### William Fogler^1^, Marilyn Franklin^2^, Matt Thayer^2^, Dan Saims^2^, John L. Magnani^1^

##### ^1^GlycoMimetics, Inc., Rockville, MD, USA; ^2^MI Bioresearch, Ann Arbor, MI, USA

###### **Correspondence:** William Fogler (wfogler@glycomimetics.com)

**Background**

Regulatory T cells (T_reg_) modulate anti-tumor immunity by suppressing T cell activation. T_reg_ are induced and maintained by immunoregulatory receptors, such as PD-L1, and respond to homing signals within the inflamed tumor microenvironment that include the endothelial cell protein, E-selectin, and the CXCR4 ligand, SDF-1. GMI-1359 is a small molecule glycomimetic beginning clinical evaluation with dual inhibitory activity against E-selectin and SDF-1. The aim of the current study was to determine if GMI-1359 alone or in combination with anti-mPD-L1 antibody affected the *in vivo* growth of CT26 colon carcinoma and to assess percentages of infiltrative intratumoral cells expressing immune markers.

**Methods**

Female Balb/c mice were implanted subcutaneously with 5x10^5^ CT26.WT tumor cells. Three days post tumor injection, mice (n = 15/group) were treated with saline, GMI-1359 (40 mg/kg for 12 consecutive days), isotype control antibody (anti-KLH) or anti-mPD-L1 antibody (10 F.9G2, 10 mg/kg on days 3, 6, 10, 13, and 17), or the combination of GMI-1359 and anti-mPD-L1 or anti-KLH. On day 15, tumors and spleens (n = 5/group) were excised and T cells (total CD4+ and CD8+, and CCR7+/CD62L+ subsets of each), regulatory T cells (T_reg_; CD4/CD25/FoxP3), and myeloid derived suppressor cells (MDSC; CD11b+/Gr1+) were determined by flow cytometry. The remaining mice were followed for tumor response.

**Results**

Treatments were well tolerated. Mice in control groups and single agent GMI-1359 were all identified with progressive disease. In contrast, treatment with anti-mPD-L1 alone or in combination with GMI-1359 produced a 40 % complete response (CR) rate. The median time to CR was shorter when anti-mPD-L1 was combined with GMI-1359 compared to anti-mPD-L1 alone (14 vs. 23 days, respectively, p < 0.0471). Evaluation of tumor infiltrating cells showed that combination therapy with GMI-1359 and anti-mPD-L1 reduced the percentage of T_reg_ compared to treatment with saline, GMI-1359 or anti-mPD-L1 as single treatments (0.9 % vs. 3.3 %, 2.9 % and 1.9 %, respectively). No other T cell subsets were affected. In spleens, the median percentage of T_reg_ were unaffected by any of the treatments and suggest that the reduction in intratumoral T_reg_ by combined treatment with anti-PD-L1 and GMI-1359 was an attenuated response to maintenance and homing signals in the tumor microenvironment.

**Conclusions**

In conclusion, these studies demonstrate that the dual E-selectin/CXCR4 antagonist, GMI-1359, in combination with anti-mPD-L1 antibody attenuates the induction and distribution of intratumoral T_reg_ and this reduction in T_reg_ is associated with a more rapid immunotherapeutic anti-tumor response.

### P205 Antibody targeting of phosphatidylserine enhances the anti-tumor responses of ibrutinib and anti-PD-1 therapy in a mouse triple negative breast tumor model

#### Jian Gong, Michael Gray, Jeff Hutchins, Bruce Freimark

##### Peregrine Pharmaceuticals, Tustin, CA, USA

###### **Correspondence:** Bruce Freimark (bfreimark@peregrineinc.com)

**Background**

Phosphatidylserine (PS) is a phospholipid normally residing in the inner leaflet of the plasma membrane that becomes exposed on vascular endothelial cells and tumor cells in the tumor microenvironment, particularly in response to chemotherapy and irradiation. Binding of antibodies targeting PS induces the recruitment of immune cells and engages the immune system to destroy tumor and associated vasculature and by blocking the immunosuppressive action of PS. Recent studies have demonstrated that PS-targeting antibodies enhance the anti-tumor activity of immune checkpoint antibody blockade to CTLA-4 and PD-1 in mouse breast and melanoma tumor models. Ibrutinib is an approved anticancer drug targeting B cell malignancies that is a selective, covalent inhibitor Bruton's tyrosine kinase (BTK) in B cell tumors. Data from recent mouse tumor studies demonstrate that ibrutinib in combination with anti-PD-1 antibody blockade inhibits growth of solid tumors, lacking BTK expression, suggesting that ibrutinib may inhibit interleukin-2 inducible T cell kinase (ITK) and promote Th1 anti-tumor responses.

**Methods**

The present study was conducted to evaluate a combination therapy including PS-targeting antibody mch1N11, ibrutinib and anti-PD-1 antibody in C57Bl/6 mice bearing triple negative E0771 breast tumors. Tumors were staged to an initial volume of ~100 mm^3^ and randomized to treatment groups (N = 10) with mch1N11 or isotype control at 10 mg/kg qw, anti-PD-1 at 2.5 mg/kg qw or ibrutinib 6 mg/kg or vehicle qd x 8. Tumor volumes were measured twice per week to determine tumor growth inhibition (TGI) relative to control treated animals. The *in vitro* sensitivity of E0771 tumor cells to ibrutinib was compared to the drug sensitive Jeko-1 cell line in a 72 hour growth and viability assay.

**Results**

The E0771 cell line is resistant *in vitro* to 10 mM ibrutinib. Tumor bearing mice treated with mch1N11, ibrutinib or anti-PD-1 alone had 22.2 %, 23.5 % and 32.6 % TGI respectively. The TGI for mch1N11 and ibrutinib was 30.5 %, ibrutinib and anti-PD-1 was 34.5 %, mch1N11 and anti-PD-1 was 36.1 %. The triple combination therapy had statistically greater TGI compared to control treated mice (59.9 %, p = 0.0084).

**Conclusions**

Treatment of solid tumors with a combination of inhibitors that target PS, ITK and the PD-1/PD-L1 axis in the tumor microenvironment provides a novel treatment for solid tumors, including triple negative breast cancer.

### P206 Gp96-Ig/costimulator (OX40L, ICOSL, or 4-1BBL) combination vaccine improves T cell priming and enhances immunity, memory, and tumor elimination

#### George Fromm, Suresh de Silva, Louise Giffin, Xin Xu, Jason Rose, Taylor H Schreiber

##### Heat Biologics, Inc., Durham, NC, USA

###### **Correspondence:** George Fromm (gfromm@heatbio.com)

**Background**

The excitement in the field of immuno-oncology over the last several years, driven largely by the clinical success of the first-wave of checkpoint inhibitors, is tempered by the fact that only 10-40 % of patients respond to these drugs given as monotherapy. It is widely believed that to improve efficacy and patient outcome, new approaches that combine treatments with more than one functionality are needed. Novel approaches that provide combination therapy in a single product, will likely lead the way.

**Methods**

We have developed a next generation cellular vaccine platform – referred to as *Com*PACT (COMbination Pan-Antigen Cytotoxic Therapy), that incorporates a tumor antigen chaperone (gp96-Ig) with T cell costimulation (Fc-OX40L), into a single tumor cell line that secretes them both (recently published in *Cancer Immunology Research* 2016).

**Results**

The current data extend these findings in additional preclinical settings. Specifically, *Com*PACT is capable of priming antigen-specific CD8+ T cells (peak: 13.3 % of total CD8+), even more so than a leading OX40 agonist antibody (8.4 %) or vaccine alone (5.6 %), and this is associated with increased CD127 + KLRG-1- memory precursor cells and antigen-specific CD4+ proliferation, with reduced off-target inflammation. Importantly, vaccine-expressed Fc-OX40L stimulated IFNγ+, TNFα+, granzyme-b + and IL-2+ by antigen-specific CD8+ T cells. This pharmacodynamic signature of an anti-tumor immune response predicted enhanced rejection of established MC38, CT26 and B16.F10 tumors. Additionally, tetramer analysis of antigen-specific CD8+ T cells (in all 3 tumor models), identified significant accumulation of tumor infiltrating lymphocytes (TIL), suggesting that *Com*PACT is not only capable of amplifying antigen-specific T cells, but these T cells can efficiently target and eliminate tumors. We have expanded our repertoire of ‘*Com*PACT’ vaccines to secrete gp96-Ig along with either Fc-TL1A, Fc-4-1BBL or Fc-ICOSL. Each costimulator/vaccine has a unique functionality, which may be context or tumor dependent. We are currently exploring these mechanistic differences.

**Conclusions**

Taken together, we show that the magnitude and specificity of vaccination can be enhanced by locally secreted costimulatory molecules when delivered within a single product. This may simplify clinical translation and importantly, provide significant patient benefit by improving safety and lowering costs.

### P207 Modulation of antibody-dependent cell-mediated cytotoxicity (ADCC) mediated by the anti-PD-L1 antibody avelumab on human lung and prostate carcinoma cell lines using the HDAC inhibitors vorinostat and entinostat

#### Massimo Fantini^1^, Sofia R Gameiro^1^, Karin M Knudson^1^, Paul E Clavijo^2^, Clint T Allen^2^, Renee Donahue^1^, Lauren Lepone^1^, Italia Grenga^1^, James W Hodge^1^, Kwong Y Tsang^1^, Jeffrey Schlom^1^

##### ^1^National Cancer Institute, National Institutes of Health, Bethesda, MD, USA; ^2^National Institute on Deafness and Other Communication Disorders, National Institutes of Health, Bethesda, MD, USA

###### **Correspondence:** Sofia R Gameiro (gameirosr@mail.nih.com)

**Background**

Chromatin deacetylation is a major determinant in epigenetic silencing of immune-associated genes, a key factor in tumor evasion of host immune surveillance. Deregulation of epigenetic enzymes, including aberrant expression of histone deacetylases (HDACs), has been associated with poor prognosis in several cancer types, including of prostate and lung origin. Vorinostat is a pan-HDAC inhibitor currently approved in the United States for the treatment of cutaneous T cell lymphoma. Entinostat is a class I HDAC inhibitor under clinical investigation for the treatment of various malignancies. HDAC inhibitors have been shown to delete immunosuppressive elements and promote synergistic antitumor effects in combination with various immunotherapies. Checkpoint inhibitors targeting PD-1/PD-L1 interactions are promising immunotherapies shown to elicit objective responses against multiple tumors. Avelumab is a fully human IgG1 mAb monoclonal antibody that inhibits PD-1/PD-L1 interaction by targeting PD-L1, and mediates ADCC against PD-L1-expressing tumor cells *in vitro*. We examined the sensitivity of human lung and prostate carcinoma cells to avelumab-mediated ADCC following clinically-relevant exposure to vorinostat or entinostat.

**Methods**

Carcinoma cells were exposed daily to vorinostat (3uM) or DMSO for 4 consecutive days, or to entinostat (500 nM) or DMSO for 72 h, prior to being examined for (a) cell-surface PD-L1 expression or (b) used as target cells lysis assay where NK cells from healthy donors were used as effectors. To examine the effect of HDAC inhibitors on PD-L1 expression *in vivo*, female nu/nu mice were implanted with NCI-H460 (lung) or PC-3 (prostate) carcinoma cells. When tumors reached 0.5-1 cm^3^, animals received 4 daily doses of DMSO or vorinostat (150 mg/kg, p.o.). Alternatively, animals received a single dose of entinostat (20 mg/kg, p.o.) or DMSO 72 h prior to tumor excision. Frozen specimens were examined for cell-surface expression of PD-L1 by immunofluorescence.

**Results**

Our results show that 1) vorinostat and entinostat significantly increase the sensitivity of human lung and prostate carcinoma cells to ADCC mediated by avelumab; 2) the anti-CD16 neutralizing mAb significantly decreases avelumab-mediated lysis of target cells exposed to either HDAC inhibitor; 3) both HDAC inhibitors can enhance tumor PD-L1 expression *in vitro* and *in vivo* in prostate and/or lung xenograft models; 4) increased avelumab-mediated ADCC of tumor targets exposed to HDAC inhibitors can occur without increased tumor PD-L1 expression.

**Conclusions**

These studies provide a rationale for combining vorinostat or entinostat with mAbs targeting PD-L1, including for patients that have failed monotherapy regimens with HDAC or checkpoint inhibitors.

### P208 Monoclonal antibodies targeting phosphatidylserine enhance combinational activity of the immune checkpoint targeting agents LAG3 and PD-1 in murine breast tumors

#### Michael Gray, Jian Gong, Jeff Hutchins, Bruce Freimark

##### Peregrine Pharmaceuticals, Tustin, CA, USA

###### **Correspondence:** Michael Gray (mgray@peregrineinc.com)

**Background**

Our previous work demonstrated that the addition of phosphatidylserine (PS) targeting antibodies to anti-programmed death ligand 1 (PD-1) therapy in murine triple negative breast cancers (TNBC) significantly enhanced immune system activation and tumor growth inhibition. In these studies, NanoString immune profile analysis showed that intratumoral levels of lymphocyte activation gene 3 (LAG3) mRNA increased in response to PS and PD-1 treatments. This suggests LAG3 may act to attenuate T cell activation in TNBC during I/O therapeutic regimens; however, it is unknown if PD-1 and LAG3 function cooperatively in regulating T cell anergy, and whether adding PS blocking antibodies can further enhance the effectiveness of LAG3 and/or LAG3 + PD-1 therapies.

**Methods**

Animal studies utilized C57bl/6 mice implanted with the murine TNBC model E0771. Immunoprofiling analysis was performed by flow cytometry and the NanoString nCounter® PanCancer Immune Profiling Panel. Antibody treatments utilized a specific phosphatidylserine targeting antibody (ch1N11), anti-PD-1, or anti-LAG3 alone or in combination. All statistical analysis utilized the student t-test (significant with p < 0.05).

**Results**

LAG3 and PD-1 were co-expressed on T cells in E0771. Mice treated with antibodies targeting PS, PD-1, and LAG3 alone in combination with each other demonstrated that the addition of PS blocking antibodies to anti-PD-1 therapy or LAG3 had significantly greater anti-tumor activity than either single agent. Comparison of PD-1 + LAG3 combinational therapy vs. single PD-1 or LAG3 treatments showed moderately more anti-tumor activity than single treatments; however, the addition of PS blocking antibodies to either checkpoint inhibitor was as equally effective in inhibiting tumor growth as observed in the combination of LAG3 + PD-1 treatment. Further comparison of PD-1 + LAG3 vs. PS + PD-1 + LAG3 treatments demonstrated that the addition of PS blocking antibodies resulted in a significant decrease in tumor growth accompanied by complete tumor regression in a greater number of animals than observed in the PD-1 + LAG3 treatment group. FACS and NanoString immunoprofiling analysis on each treatment group showed that the addition of PS blocking antibodies to all checkpoint treatment groups, including the combination of PD-1 + LAG3, resulted in enhanced tumor infiltrating lymphocytes (TILs), a reduction of myeloid derived suppressor cells (MDSCs), and enhanced cytokines associated with immune system activation.

**Conclusions**

Overall, our data demonstrate that while PS, LAG3, and PD-1 therapies each have efficacy in TNBC as single agents, I/O treatments that include PS blocking antibodies offer significantly improved growth inhibition and are capable of increasing TILs compared to single and combinational treatments by T cell checkpoint targeting inhibitors alone.

### P209 The immunoreceptor TIGIT regulates anti-tumor immunity

#### Jane Grogan, Nicholas Manieri, Eugene Chiang, Patrick Caplazi, Mahesh Yadav

##### Genentech, South San Francisco, CA, USA

###### **Correspondence:** Jane Grogan (grogan.jane@gene.com)

**Background**

Strategies to re-activate exhausted anti-tumor immune responses with antibody blockade of key T cell co-inhibitory receptors such as PD-1/PD-L1 or CTLA-4 have demonstrated transformational potential in the clinic. TIGIT (a PVR-nectin family member) is a dominant immuno-inhibitory receptor on tumor-specific T and NK cells, shown to regulate anti-tumor immunity. Activation of TIGIT on T and NK cells limits proliferation, effector cytokine production, and killing of target tumor cells. The high affinity receptor for TIGIT is PVR, and the counter agonist receptor is CD226, all of which are members of the PVR-nectin family. TIGIT is elevated in the tumor microenvironment in many human tumors and coordinately expressed with other checkpoint immune receptors such as PD-1. However, the spatial and coordinate expression of these receptors and ligands required for these functions, and the cell-types involved in anti-tumor immunity, remains unknown.

**Methods**

TIGIT, CD226 and PD-L1 blockade will be assessed in preclinical syngeneic tumor model CT26 and MC38. To determine which immune cells are important for allowing tumor progression early and late in disease mice with cell-specific gene ablation for these family members were challenged with tumors. Tumor growth was determined and tumor sections labeled and probed by fluorescence microscopy to assess TIGIT, CD226 and PVR cellular expression.

**Results**

In mouse models of both cancer, antibody co-blockade of TIGIT and PD-L1 enhanced CD8^+^ T cell effector function, resulting in significant tumor clearance. TIGIT is expressed on CD8+ T cell, Treg and NK cells. Specific ablation of TIGIT on CD8+ T cells resulted in tumor clearance, and was dependent on PVR in the host tissue. Immunofluorescence studies will be presented.

**Conclusions**

Therapeutic blockade of TIGIT may result in improved eradication of malignancies when used in conjunction with other anti-cancer therapies including those that modulate anti-tumor immune responses, and is currently being tested in phase I clinical trials. Models indicate that inhibition of TIGIT with a blocking mAb may release CD226 to activate tumor-specific T cells. Another mechanism could involve regulation of T cell suppression by TIGIT on regulatory T cells. A better understanding of the coordinate interaction between these receptors and ligands in tumors will be informative for the appropriate application of checkpoint-therapy combinations.

### P210 CC-122 in combination with immune checkpoint blockade synergistically activates T cells and enhances immune mediated killing of HCC cells

#### Patrick Hagner^1^, Hsiling Chiu^1^, Michelle Waldman^1^, Anke Klippel^1^, Anjan Thakurta^1^, Michael Pourdehnad^2^, Anita Gandhi^1^

##### ^1^Celgene Corporation, Summit, NJ, USA; ^2^Celgene Corporation, San Francisco, CA, USA

###### **Correspondence:** Patrick Hagner (phagner@celgene.com)

**Background**

CC-122 binds the E3 ubiquitin ligase CRL4^CRBN^ resulting in the degradation of the transcription factor Aiolos and activation of T cells. Preclinical and clinical data obtained in hematologic malignancies indicate that CC-122 exerts immunomodulatory activity through enhanced antibody dependent cell-mediated cytotoxicity and a shift in T cell subsets from a naïve to effector and memory subsets. CC-122 is in clinical development in multiple hematologic diseases and in solid tumors such as hepatocellular carcinoma (HCC) as a single agent (NCT01421524) and in combination with nivolumab (nivo). The effects of combining CC-122 with immune checkpoint antibodies in *in vitro* models of T cell activation and immune co-culture models with HCC cells were examined.

**Methods**

Carboxyfluorescein succinimidyl ester (CFSE) based proliferation, cytokine production and immune co-culture assays were performed with stimulated peripheral blood mononuclear cells (PBMC) from healthy donors followed by drug treatment. Drug combinations were investigated in mixed lymphocyte reactions (MLR) with monocyte derived dendritic cells and T cells from separate donors. Apoptosis was measured via Annexin V/ToPro3 staining. Synergy calculations were performed with the fractional product method.

**Results**

In a 3-day CD3-stimulated PBMC assay, CC-122 (1-10 μM) treatment elevated HLA-DR, a marker of T cell activation, by 3.4-5.5 and 3.2-5.3 fold in CD4^+^ and CD8^+^ T cells, respectively. Proliferation of CD4^+^ and CD8^+^ T cells from CD3-stimulated PBMC treated with vehicle, CC-122 (50nM), nivo (50 μg/ml) or the combination was assessed via CFSE staining. The percentage of proliferating vehicle-treated CD4^+^ and CD8^+^ cells was 37 % and 40 %, compared to nivo (45 % and 47 %), CC-122 (54 % and 68 %) and the combination (61 % and 74 %). SEB stimulated PBMC were treated with CC-122 (40nM), and nivo or α-PD-L1 (0.1-100 μg/ml) resulting in secretion of 424, 160 and 154 ng/ml IL-2, respectively. The combination of CC-122 with either nivo or α-PD-L1 (10 μg/ml) resulted in synergistic IL-2 secretion levels of 873 and 813 ng/ml, respectively. In an MLR assay, the combination of CC-122 (100nM) with nivo (10 μg/ml) or α-PD-L1 (10 μg/ml) resulted in synergistic IL-2 and IFNγ secretion. Finally, the combination of CC-122 and nivo or CC-122 and α-PD-L1 significantly increased PBMC-mediated cytotoxicity of HCC cells compared to either single agent or isotype control (p ≤ 0.05).

**Conclusions**

CC-122 in combination with nivo or anti-PD-L1 antibodies results in synergistic activation of T cells and significantly enhanced immune mediated cytotoxicity against HCC cells. Given the novel mechanism of immunomodulation by CC-122 and synergistic combination with checkpoint blockade, clinical investigation in HCC is currently in progress.

### P211 Ubiquitin-specific protease 6 (USP6) oncogene confers dramatic sensitivity of sarcoma cells to the immunostimulatory effects of interferon

#### Ian Henrich^1^, Laura Quick^2^, Rob Young^2^, Margaret Chou^2^

##### ^1^University of Pennsylvania, Philadelphia, PA, USA; ^2^Children's Hospital of Pennsylvania, Philadelphia, PA, USA

###### **Correspondence:** Ian Henrich (ihenrich@mail.med.upenn.edu)

**Background**

Bone and soft tissue tumors (BSTTs) represent a heterogeneous class of neoplasms that disproportionately affect children. Compared to other malignancies, BSTTs are poorly understood, which has hampered the development of effective therapies. Our lab previously discovered that the oncogenic de-ubiquitylating enzyme USP6 is the key etiologic agent in several benign BSTTs, and is selectively overexpressed in multiple sarcomas, a malignant class of BSTTs [1]. USP6 drives tumorigenesis by directly de-ubiquitylating the Jak1 kinase, leading to its stabilization and activation of STAT transcription factors [2]. Since the Jak1-STAT pathway is a central mediator of interferon (IFN) signaling, we hypothesized that USP6 overexpression in sarcomas would render them hypersensitive to the immune stimulatory effects of IFN, which could be exploited for therapeutic benefit.

**Methods**

USP6 was expressed in a doxycycline-inducible manner in various patient-derived sarcoma cell lines, including Ewing sarcoma, rhabdomyosarcoma, leiomyosarcoma, and liposarcoma. USP6 expression levels were confirmed to approximate those in primary patient tumor samples.

**Results**

USP6 conferred exquisite sensitivity of sarcoma cells to the immuno-modulatory effects of IFN. Activation of STAT1 and STAT3 were both enhanced and prolonged in sarcoma cells expressing USP6 upon IFN treatment. RNA-sequencing confirmed that USP6 induces an IFN response signature by itself, and that it synergizes with IFN to dramatically induce interferon-stimulated gene (ISG) expression. The ISGs synergistically induced by USP6 and IFN include a large group of anti-tumor and immunomodulatory genes: the pro-apoptotic ligand TRAIL was dramatically elevated and mediated apoptosis of USP6-expressing sarcoma cells. Immunomodulatory factors synergistically induced by USP6 and IFN included chemokines and cytokines that drive migration and differentiation of T cells.

**Conclusions**

USP6 overexpression sensitized sarcoma cells to IFN, simultaneously inducing TRAIL-mediated death and stimulating sarcoma cells to produce immune stimulatory/anti-tumorigenic chemokines and cytokines. This dual mechanism of action may position IFN as an extremely effective therapeutic agent for treatment of sarcomas that overexpress USP6.

**References**

1. Oliveira A, Chou M: **The TRE17/USP6 oncogene: a riddle wrapped in a mystery inside an enigma.** *Front Biosci (Schol Ed)* 2012, **4**:321–340.

2. Quick L, Young R, Henrich I, Wang X, Asmann Y, Oliveira A, Chou M: **Jak1-STAT3 signals are essential effectors of the USP6/TRE17 oncogene in tumorigenesis.** *Cancer Res* 2016, in press.

**Fig. 8 (abstract P211).**
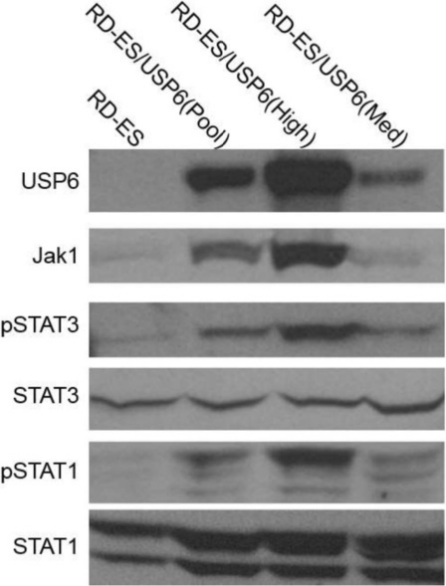
USP6 Expression in RD-ES Cell Line. USP6 was expressed in a doxycycline-inducible in the patient derived Ewing sarcoma cell line. Clonal lines that express high or medium amounts of USP6 were isolated from the initial pooled population. Expression of USP6 increased Jak1 levels and activation of downstream effectors STAT1 and STAT3 in an USP6 dose dependent manner. Note: Similar lines were/are being created for other sarcomas. RD-ES is used as an example

**Fig. 9 (abstract P211).**
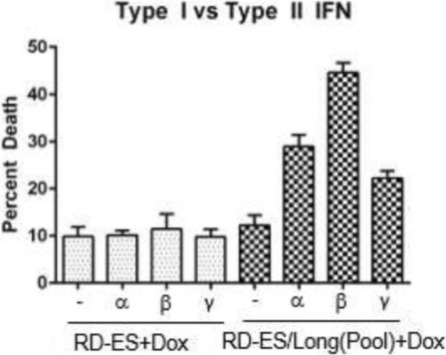
USP6 Sensitizes Cells to IFN-Induced Death. RD-ES were treated with 1000 U/mL IFNa, IFNB, or IFNy for 24 hours with or without USP6 expression. IFNB was most effective in inducing death (~50%). Death was monitored via trypan blue exclusion

**Fig. 10 (abstract P211).**
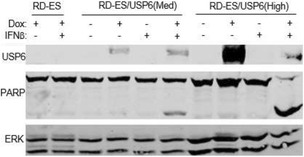
USP6 Expression Determines Sensitivity to IFN-Induced Death. RD-ES, RD-ES/USP6(Med), and RD-ES/USP6(High) were treated with 1000 U/mL IFNB overnight. Higher USP6 increases the sensitivity of the cells to IFNB induced death. Death was monitored by PARP cleavage

**Fig. 11 (abstract P211).**
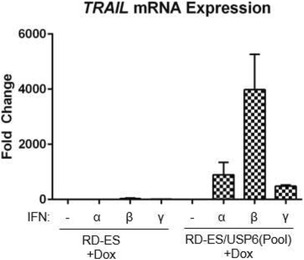
USP6 Synergizes with IFN to Massively Upregulate TRAIL. RD-ES were treated with 1000 U/mL IFNa, IFNB, or IFNy for 24 hours with or without USP6 expression. TRAIL was found to be synergistically induced by IFN in the presence of USP6. IFNB was the most potent at nearl5 5000-fold over baseline

**Fig. 12 (abstract P211).**
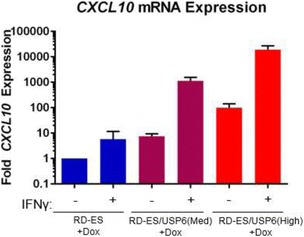
USP6 Synergizes with IFNy to Increase Chemokine Expression. RD-ES, RD-ES/USP6(Med), and RD-ES/USP6(High) were treated with 10 U/mL IFNy overnight. The chemokine CXCL10 was synergistically induced in an USP6 dose-dependent manner. Similar expression patterns were seen for other chemokines like CXCL9 and CXCL11

**Fig. 13 (abstract P211).**
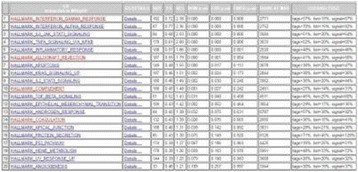
USP6 Induces an IFN-Response In Vitro. RNA-sequencing was performed on RD-ES/USP6(Pooled) were treated with or without dox. The resulting gene expression data was analyzed using Gene Sequencing Enrichment Analysis (GSEA) using the Hallmark dataset (the hallmark dataset contains curated gene sets that are known to be part of key cellular pathways). USP6 induces a strong IFN-response and Jak-STAT gene expression

**Fig. 14 (abstract P211).**
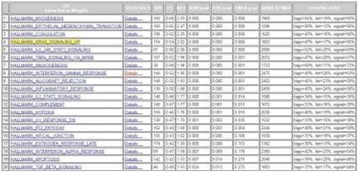
USP6 Induces an IFN-Response In Vivo (Ewings Sarcoma). The Ewing sarcoma patient microarray dataset (GSE37371) was sorted based on USP6 expression and the top 5 USP6 expressing patient samples were compared to the bottom 5 using GSEA. Similar to the *in vitro* results, high USP6 expression correlates with activation of Jak-STAT and an IFN-response signature

**Fig. 15 (abstract P211).**
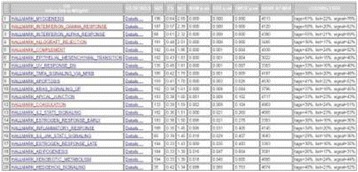
USP6 Induces an IFN-Response In Vivo (Rhabdomyosarcoma). The Ewing sarcoma patient microarray dataset (GSE66533) was sorted based on USP6 expression and the top 5 USP6 expressing patient samples were compared to the bottom 5 using GSEA. Similar to the *in vitro* results, high USP6 expression correlates with activation of Jak-STAT and an IFN-response signature

**Fig. 16 (abstract P211).**
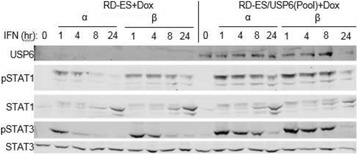
USP6 Potentiates IFN Signaling In Vitro. RD_ES/USP6(Pooled) or RD-ES lines were treated with 1000 U/mL IFNa of IFNB for the indicated time period. USP6 extended the duration and amplified the magnitude of STAT1 and STAT3 activation

### P212 CPI-444: a potent and selective inhibitor of adenosine 2A receptor (A2AR) induces anti-tumor responses alone and in combination with anti-PD-L1

#### Andrew Hotson, Stephen Willingham, Po Ho, Carmen Choy, Ginna Laport, Ian McCaffery, Richard Miller

##### Corvus Pharmaceuticals, Burlingame, CA, USA

###### **Correspondence:** Andrew Hotson (ahotson@corvuspharma.com)

**Background**

Elevated extracellular adenosine in the tumor microenvironment is immunosuppressive promoting tumor growth and metastasis through signaling via A2AR on immune cells. CPI-444 is a potent, oral, selective A2AR antagonist that has been well tolerated in phase I/Ib studies.

**Methods**

Preclinical studies were performed with MC38 mouse tumor models and primary human PBMCs. Based on these results, we have initiated a phase Ib trial to examine safety, tolerability, biomarkers, and efficacy of CPI-444 as single agent and in combination with anti-PD-L1 antibody, atezolizumab, in patients with selected solid tumors. Peripheral blood and tumor biopsies are collected pre- and post-treatment for biomarker analysis.

**Results**

Pre-clinical studies demonstrated cross-talk between adenosine and PD-1/PD-L1 pathways, providing rationale for combination therapy. In MC38, anti-PD-L1 treatment resulted in elevated CD39 and CD73 expression on T cells, consistent with increased capacity to generate adenosine. The adenosine analog NECA inhibited TCR-mediated ERK phosphorylation and production of IL-2 and IFNγ in human PBMCs; these inhibitory effects were blocked by CPI-444. Treatment of MC38 with CPI-444 led to inhibition of tumor growth, with tumor elimination in ~30 % of mice. Combining CPI-444 with anti-PD-L1 synergistically inhibited tumor growth and eliminated tumors in 90 % of mice. When cured mice were re-challenged with MC38, tumors were uniformly rejected, indicating CPI-444 induced systemic anti-tumor memory. CD8^+^ depletion abrogated efficacy of CPI-444 ± anti-PD-L1 treatment. Biomarker results from the ongoing phase Ib trial demonstrate CPI-444 neutralizes A2AR signaling and activates markers of an immune response. To measure A2AR inhibition, peripheral blood samples were activated with NECA and pCREB quantified using flow cytometry. A2AR signaling was robustly inhibited in 8 of 9 patients in an exposure dependent manner. Of the patients evaluated so far, immune activation was observed by flow cytometry analysis of PD-1/CD8 frequency in all continuously treated patients and a subset of patients on the 14 day schedule. IHC and gene expression of pathway markers in serial tumor biopsies will be discussed.

**Conclusions**

In total, this shows that CPI-444 exhibits functional inhibition of adenosine signaling, and treatment is associated with activation of markers of anti-tumor immunity. This is the first demonstration of immune modulation in cancer patients receiving an adenosine antagonist.

### P213 Probody^TM^ therapeutics targeting the PD-1/L1 axis provide preclinical anti-tumor efficacy while minimizing induction of autoimmunity as single agents and in combination with CTLA-4 blockade

#### Kimberly A Tipton, Kenneth R Wong, Victoria Singson, Chihunt Wong, Chanty Chan, Yuanhiu Huang, Shouchun Liu, Jennifer H Richardson, W Michael Kavanaugh, James West, Bryan A Irving

##### CytomX Therapeutics, Inc., South San Francisco, CA, USA

###### **Correspondence:** Bryan A Irving (birving@cytomx.com)

**Background**

Immunotherapy has transformed cancer treatment by unleashing potent and durable anti-tumor immunity against many cancers. However, because many of the same mechanisms control anti-tumor immunity and self-tolerance, these therapies can also induce systemic autoimmunity by activating autoreactive T cells in normal tissues. Combinations of checkpoint inhibitors targeting PD-1 and CTLA-4 increase clinical response rates, but similarly increase toxicities, thereby reducing their clinical potential. New approaches are therefore needed that provide anti-tumor activity without dysregulating systemic immunity.

**Methods**

CytomX has developed Probody therapeutics (Pb-Tx) that are proteolytically-activated antibodies designed to widen the therapeutic index by minimizing drug interaction with normal tissue while retaining anti-tumor activity. Pb-Tx are “masked” to attenuate binding to target in healthy tissue, but can become “unmasked” in the tumor microenvironment by tumor-specific protease activity.

**Results**

*In vitro*, the masked PD-1 Pb-Tx had reduced affinity for mouse PD-1 relative to the parental antibody. Binding affinity was completely restored following addition of appropriate proteases. In mice, single-agent antibodies to CTLA-4 and to PD-1, and the PD-1 Pb-Tx induced 10 %, 30 %, and 20 % complete tumor regressions (CRs) against established MC38 tumors, respectively. In combination with anti-CTLA-4, both PD-1 antibody and Pb-Tx induced 80 % CRs and generated effective T cell memory against tumor re-challenge. In 10-week-old NOD mice, a 1 or 10 mpk single dose of anti-PD-1 antibody induced diabetes in 43 % and 57 % of mice, respectively, while a 10 mpk dose of PD-1 Pb-Tx yielded only 14 % disease incidence with delayed onset. In younger NOD mice, the CTLA-4/PD-1 antibody combination induced diabetes in 50 % of mice. In contrast, mice administered the PD-1 Pb-Tx/CTLA-4 antibody combination were completely protected. Similar data were generated with a PD-L1-targeted Pb-Tx.

**Conclusions**

A PD-1 targeted Pb-Tx provided equivalent anti-tumor efficacy in mice to that of its parental antibody while protecting from anti-PD-1-mediated autoimmunity, both as a single agent and in combination with a CTLA-4 antibody. These results demonstrate that PD-1 Pb-Tx retain anti-tumor efficacy with improved safety profiles preclinically and therefore have promise to enable safer combination immunotherapies.

### P214 Enhancement of target expression on breast tumors via hormone receptor antagonism: a novel strategy for enhancing immunotherapeutic efficacy

#### Ritika Jaini, Matthew Loya, Charis Eng

##### Lerner Research Institute, Cleveland Clinic, Cleveland, OH, USA

###### **Correspondence:** Ritika Jain (jainir@ccf.org)

**Background**

Immunotherapy has historically been successful in highly antigenic tumors but mostly failed in non-antigenic tumors. Our studies in autoimmunity have shown that increased antigen load within a tissue enhances immune reactivity against it. We hypothesize that enhancing protein target expression on breast tumors can increase the efficacy of targeted immunotherapy. Lactation proteins have recently been shown to be effective immunotherapeutic targets on breast tumors. Since lactation proteins are negatively regulated by signaling via the estrogen receptor (ER), we hypothesize that target lactation protein expression on breast tumors can be increased by antagonism of the ER in order to enhance efficacy of antigen specific immunotherapy/vaccination.

**Methods**

Enhancement of target protein expression in human breast tumors was tested *in vitro* by treatment of ER+ (MCF7 cells) and ER + PR+ (T47D cells) with different doses of the clinically approved ER modulator, tamoxifen. *In vivo* modulation of target antigen expression was tested by inoculating 6–7 week old Balb/cJ female mice with 4 T1 breast tumors followed by oral treatment with tamoxifen. Increase in lactation protein expression (e.g. alpha lactalbumin) was assayed by *in vitro* Luciferase assays followed by confirmation by immunoblotting and immunohistochemistry at different time points post treatment. Effect of increased antigen expression on efficacy of targeted immunotherapy was assessed by antigen specific immunization of 4 T1 tumor bearing mice with or without tamoxifen administration and comparing tumor growth.

**Results**

Our *in vitro* studies on human tumors and *in vivo* murine studies show that antagonism of the ER via tamoxifen treatment can substantially increase expression of target lactation proteins such as alpha lactalbumin on breast tumors. We show that whereas at least a 2–3 fold increased expression of the target protein can be achieved on tumors, normal breast tissue remains unaffected. Tumor progression studies revealed that in spite of increased target expression, no enhancement in efficacy of immunotherapy was achieved via active immunization protocols. However, efficacy of cell based targeted immunotherapies can possibly be enhanced when applied in combination with our proposed strategy to increase target expression.

Conclusions

Singular increase in target antigen expression on tumors is not effective in enhancing efficacy of immunotherapy probably due to associated limiting factors such as DC trafficking, antigen presentation and effective priming. However, the efficacy of cell-based targeted immunotherapeutic strategies that circumvent the limitations around active priming can be enhanced by using our combinatorial strategy of enhancing antigen expression on tumors via hormone receptor antagonism.

**Acknowledgements**

The PhRMA Foundation for funding support.

### P215 Dose escalation/confirmation results of ENCORE 601, a phase Ib/II, open-label study of entinostat (ENT) in combination with pembrolizumab (PEMBRO) in patients with non-small cell lung cancer (NSCLC)

#### Melissa L Johnson^1^, Alex A Adjei^2^, Mateusz Opyrchal^3^, Suresh Ramalingam^4^, Pasi A Janne^5^, George Dominguez^6^, Dmitry Gabrilovich^6^, Laura de Leon^7^, Jeannette Hasapidis^7^, Scott J Diede^8^, Peter Ordentlich^7^, Scott Cruickshank^7^, Michael L Meyers^9^, Matthew D Hellmann^10^

##### ^1^Sarah Cannon Research Institute, Nashville, TN, USA; ^2^Mayo Clinic, Rochester, MN, USA; ^3^Roswell Park Cancer Institute, Buffalo, NY, USA; ^4^Emory University, Atlanta, GA, USA; ^5^Dana-Farber Cancer Institute, Boston, MA, USA; ^6^The Wistar Institute, Philadelphia, PA, USA; ^7^Syndax Pharmaceuticals, Inc., Waltham, MA, USA; ^8^Merck Research Laboratories, North Wales, PA, USA; ^9^Syndax Pharmaceuticals, Inc., New York, NY, USA; ^10^Department of Medicine, Memorial Sloan Kettering Cancer Center, New York, NY, USA

###### **Correspondence:** Melissa L Johnson (mjohnson@tnonc.com)

**Background**

ENT is an oral, class I selective histone deacetylase (HDAC) inhibitor shown in animal models to reduce immunosuppressive myeloid derived suppressor cells (MDSCs) and regulatory T cells (Tregs) and produce synergistic anti-tumor responses when combined with immune checkpoint inhibition. ENCORE 601 is a phase Ib/II study designed to evaluate ENT plus PEMBRO in patients with advanced NSCLC. The objective of the phase Ib dose escalation/confirmation portion was to determine the recommended phase II dose (RP2D).

**Methods**

Patients with stage III/IV NSCLC (previous anti-PD-1/PD-L1 therapy was permitted) were enrolled in a 3 + 3 dose escalation phase. ENT 3 mg and 5 mg QW PO + PEMBRO 200 mg Q3W IV in 21-day cycles were explored to determine the safety and RP2D, followed by a dose-confirmation cohort (n = 9). Pre-treatment biopsies were required. Correlative studies included tumor PD-L1 expression and phenotypic and functional evaluation of immune cell subsets in peripheral blood and tumor tissue.

**Results**

Twenty-two NSCLC patients (9 of which progressed on prior anti-PD-1/PD-L1 therapy) were treated with ENT plus PEMBRO; 13 in the dose escalation phase (6 at ENT 3 mg and 7 at ENT 5 mg) and 9 in the dose confirmation phase (ENT 5 mg). Of 20 patients with PD-L1 expression results, 7 patients (35 %) had PD-L1 < 1 %, 8 (40 %) had PD-L1 1-49 %, and 5 (25 %) had PD-L1 ≥ 50 %. During dose escalation, 1 patient previously treated with anti-PD-1 therapy experienced a DLT at Cycle 2 Day 15 (ENT 3 mg, Grade 3 immune-mediated hepatitis), and no other DLTs were observed. Among all 22 patients treated, Grade 3/4 treatment-related AEs included hypophosphatemia (9 %), neutropenia (5 %), anemia (5 %), acute respiratory failure (5 %), elevated alkaline phosphatase (5 %) and immune-mediated hepatitis (5 %). Of 6 evaluable patients previously exposed to anti-PD-1/PD-L1, best response includes 3 SD and 3 PD. Of 11 evaluable anti-PD-1/PD-L1 naïve patients, best response includes 1 PR, 1 SD and 9 PD. A reduction in peripheral MDSC levels was observed between pre-treatment and Cycle 2 Day 1 in 7 of 11 patients assessed (median decrease of 40.7 % PMN-MDSCs; 67.3 % M-MDSCs).

**Conclusions**

5 mg ENT weekly combined with PEMBRO has a manageable safety profile, expected pharmacodynamic effects on reducing MDSCs, and will be further explored in the phase II expansion cohorts including NSCLC and melanoma.

**Trial Registration**

ClinicalTrials.gov identifier NCT02437136.

### P216 Combinatorial reprograming of chemokine environment in colorectal and ovarian cancer patients to promote intratumoral CTL infiltration

#### Pawel Kalinski^1^, Amer Zureikat^2^, Robert Edwards^3^, Ravi Muthuswamy^3^, Nataša Obermajer^4^, Julie Urban^3^, Lisa H Butterfield^2^, William Gooding^2^, Herbert Zeh^3^, David Bartlett^4^

##### ^1^Department of Surgery; University of Pittsburgh Cancer Institute; Department of Infectious Diseases and Microbiology, University of Pittsburgh, Pittsburgh, PA, USA; ^2^University of Pittsburgh Cancer Institute, Pittsburgh, PA, USA; ^3^University of Pittsburgh, Pittsburgh, PA, USA; ^4^Department of Surgery, University of Pittsburgh, Pittsburgh, PA, USA

###### **Correspondence:** Pawel Kalinski (kalinskip@upmc.edu)

**Background**

Since the infiltration of tumor tissues with effector CD8^+^ T cells (CTLs) is associated with improved clinical outcomes and predicts patients’ responsiveness to checkpoint blockers, we developed a combinatorial approach to selectively enhance the production of CTL chemokines in tumor lesions, while avoiding the activation of healthy tissues. Our preliminary data from human *ex vivo* tissue culture models demonstrate that a) TLR3-based combinatorial adjuvants selectively induce CTL-attracting chemokines in tumor-associated stromal and myeloid cells, while avoiding undesirable activation of cancer cells and surrounding non-tumor tissues; b) combination of TLR3 ligands with IFNa synergistically amplifies the production of CTL-attracting chemokines and allows to uniformly induce their production in all tumor lesions; and c) that the inclusion of COX2 blockers prevents the induction of Treg-attractants.

**Methods**

Based on these preclinical data, we developed a phase I/II clinical trial (UPCI 10-131/NCT01545141) to determine the safety and local effectiveness of intravenous infusion of rintatolimod (Ampligen; selective TLR3 ligand) combined with Intron A and oral celecoxib, in patients with resectable recurrent colorectal cancer. Our phase I/II trial UPCI 11–128 (NCT02432378) evaluates the safety, feasibility and local effectiveness of the intraperitoneal delivery of rintatolimod and Intron A (with oral celecoxib) in cisplatin-treated patients with recurrent ovarian cancer.

**Results**

In the completed phase I of UPCI 10–131, we observed very good safety profile of this combination and, in accordance with our expectations, selective disappearance of CTL-, TH1- and NK cell markers from circulation, lasting 24–48 hours after rintatolimod/Intron A infusion. Comparison of the resected tumors demonstrated enhanced intratumoral ratios of CXCL10 (CTL-attractant) to CCL22 (Treg-attractant) and CD8α (CTL marker) to FoxP3 (Treg marker), in the treatment cohort, compared to patients receiving standard care at our center (non-randomized control). The randomized phase II portion of this clinical study is ongoing. Our recently-implemented study UPCI 11–128 provides preliminary indication of the feasibility and local effectiveness of intraperitoneal modulation of tumor microenvironments in cisplatin-treated ovarian cancer patients.

**Conclusions**

Our data provide early indications of the safety and feasibility of using combinatorial adjuvants to selectively enhance intratumoral CTL infiltration. Verification of these results in randomized phase II portions of our trials may provide new means to enhance the clinical effectiveness of checkpoint inhibitors, therapeutic vaccines and adoptive T cell therapies (ACT) against “cold tumors”, enhancing the scope of their applications.

**Acknowledgements**

This project was supported by the NIH grants P01 CA132714 and P50 CA159981. Rintatolimod was provided under MTA by Hemispherx Bio.

**Trial Registration**

ClinicalTrials.gov identifier NCT01545141.

### P217 A model system to characterize the personalized cell immunotherapy, AGS-003, and predict functional activity in combination with PD-1 checkpoint inhibitor and sunitinib

#### Olga Zubkova^1^, Larissa Agapova^1^, Marina Kapralova^1^, Liudmila Krasovskaia^1^, Armen Ovsepyan^2^, Maxim Lykov^2^, Artem Eremeev^1^, Vladimir Bokovanov^1^, Olga Grigoryeva^1^, Andrey Karpov^1^, Sergey Ruchko^1^, Charles Nicolette^3^, Alexandr Shuster^2^

##### ^1^LLC Cellthera Pharm, Volginsky, Vladimir, Russia; ^2^LLC IBC Generium, Volginsky, Vladimir, Russia; ^3^Argos Therapeutics Inc., Durham, NC, USA

###### **Correspondence:** Andrey Karpov (apkarpov@cellthera.ru)

**Background**

AGS003 is an immunotherapy consisting of autologous dendritic cells (DCs) electroporated with amplified total tumor RNA plus synthetic CD40L RNA and is currently being tested in combination with standard of care to extend survival of newly diagnosed metastatic RCC patients in the phase III ADAPT clinical trial. We set out to establish an animal model system to more thoroughly study the AGS003 mechanism of action and assess the functionality in combination with other therapeutic agents.

**Methods**

Mouse DC precursors were processed in a similar manner to how human monocytes are processed to manufacture AGS003. Bone marrow cells from 7–10 week old Balb/c mice were incubated with GMCSF and IL4 and matured with TNFa, IFNg and PGE2. Mature DCs were electroporated with total tumor RNA from RENCA tumor plus synthetic mCD40L RNA and injected i.p. to treat syngeneic BALB/c mice in an orthotopic RCC model. This model system was utilized to test the AGS003-like mouse DCs as a single agent or in combination with the mPD-1 checkpoint inhibitor.

**Results**

Here we report on successfully developing murine DCs with similar properties to AGS003, including phenotype (CD80, CD83, CD86, MHCI, CCR7), secretion of IL12 induced by CD40L RNA and induction of CD8 + CD28 + CD45RA- memory T cells *in vivo*. Results showed that, as a single agent, the DC therapy was superior to PBS controls at increasing median survival, slowing tumor growth and decreasing lung metastases. These effects were dependent on inclusion of the amplified total RENCA cell RNA based on comparison with irrelevant RNA controls. In addition, the greatest control of tumor growth rate and median survival occurred when combined with the PD-1 checkpoint inhibitor and sunitinib.

**Conclusions**

These data demonstrate the importance of amplified total tumor RNA in directing immune responses against the corresponding tumor target and support the strategy of generating and/or augmenting preexisting antitumor immune responses with active immunotherapy to maximize clinical benefit when combined with PD-1 checkpoint inhibition and sunitinib as the standard therapy drug for renal cell carcinoma. This model system may be useful to explore additional combination therapies with other therapeutic agents.

**Fig. 17 (abstract P217).**
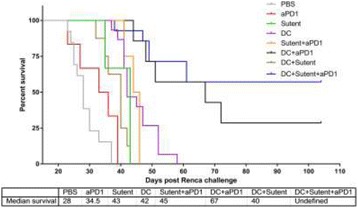
Survival curve of DC-based combination therapy in RCC mouse model. DC monotherapy showed a 50% increase in median OS compared to the PBS group (42 vs 28 days). DC + Sutent combination therapy did not show increased OS compared to monotherapy with DCs or Sutent (40 vs 42 or 43 days). DC + aPD-1 combination therapy showed significantly increased median OS compared to monotherapy with DCs or aPD-1 (67 vs 42 or 34.5 days). DC + Sutent + aPD-1 combination therapy showed significantly increased media OS (>104 days) compared to all other groups tested

### P218 Local intratumoral treatment with low-dose CD40 and TLR4 agonists overcomes resistance to PD-1 blockade to control tumors systemically

#### Danny N. Khalil^1^, Luis Felipe Campesato^1^, Yanyun Li^2^, Taha Merghoub^2^, Jedd D. Wolchok^3^

##### ^1^Memorial Sloan Kettering Cancer Center, New York, NY, USA; ^2^Ludwig Collaborative Laboratory, Memorial Sloan Kettering Cancer Center, New York, NY, USA; ^3^Department of Medicine, Memorial Sloan Kettering Cancer Center, New York, NY, USA

###### **Correspondence:** Danny N. Khalil (khalild@mskcc.org)

**Background**

Multiple cancer types resistant to immune checkpoint blockade (i.e., anti-PD-1, PD-L1, and/or CTLA-4) also demonstrate impaired antigen presenting cell (APC) activation. We hypothesized that intratumoral administration of agents designed to enforce APC activation would convert a living tumor into a source of immunogenic APCs capable of priming anti-tumor T cells.

**Methods**

Using a checkpoint-blockade resistant syngeneic B16 murine model with established (0.5 - 1 cm) bilateral tumors, we screened and characterized agents associated with APC activation for their ability to overcome resistance to anti-PD-1 therapy. Such agents were administered either systemically, or intratumorally to one of the two tumors, allowing us to distinguish the effect at the injected tumor from that at the contralateral tumor.

**Results**

In the setting of PD-1 blockade, we found that intratumoral treatment with the TLR4 agonist monophosphoryl lipid A (MPL) and low-dose CD40 agonist monoclonal antibody (mAb) induces an anti-tumor T cell response. CD8+ T cells subsequently infiltrate and control noninjected tumors at a distant site. Interestingly, locally injected tumors were heavily infiltrated with neutrophils expressing costimulatory markers including CD86 within 3 hours of treatment, and then rapidly regressed. In addition, there was persistence of activated dendritic cells and monocytes in the injected-tumor’s draining lymph node. Within 1 week, distant tumors were infiltrated with activated CD8+ T cells, and showed a marked increase in the T effector to T regulatory ratio. The control of distant tumors was abolished in RAG1-deficient animals lacking lymphocytes. Cured animals fully resisted tumor re-implantation at 90 days, developing fur depigmentation at both the site of initial treatment and the untreated tumor reimplantation site, but not elsewhere, suggesting a highly specific anti-tumor response. Notably, systemic administration of MPL and CD40 at 25-fold higher dose was less effective than intratumoral treatment.

**Conclusions**

In conclusion, low-dose intratumoral treatment with combined TLR4 and CD40 agonists induces anti-tumor T cells which in turn infiltrate tumors at distant sites and provide durable immunity such that animals are resistant to tumor re-implantation. Given that this regimen relies on agents that are FDA-approved for other indications, or in clinical development, it can readily be translated into clinical trials across a broad range of malignancies that are currently refractory to immunotherapy.

### P219 CA-170, a first in class oral small molecule immune checkpoint antagonist, promotes T cell immune activation and inhibits tumor growth in pre-clinical models of cancer

#### Adam S Lazorchak^1^, Troy D Patterson^1^, Yueyun Ding^1^, Pottayil Sasikumar^2^, Naremaddepalli Sudarshan^2^, Nagaraj Gowda^2^, Raghuveer Ramachandra^2^, Dodheri Samiulla^2^, Sanjeev Giri^2^, Rajesh Eswarappa^2^, Murali Ramachandra^2^, David Tuck^1^, Timothy Wyant^1^

##### ^1^Curis, Inc., Lexington, MA, USA; ^2^Aurigene Discovery Technologies Limited, Bangalore, Karnataka, India

###### **Correspondence:** Adam S Lazorchak (alazorchak@curis.com)

**Background**

Antibody-mediated immune checkpoint blockade has transformed cancer therapy. However, the majority of patients fail to respond to antibody therapies targeting single immune checkpoint pathways and antibodies exhibit a long *in vivo* half-life which may contribute to the emergence of immune-related adverse events. Additionally, antibody therapies must be administered by intravenous infusion in a hospital or clinic setting, which places additional burden on patients who may have mobility challenges. CA-170 is a small molecule, orally bioavailable antagonist of the PD-L1, PD-L2 and VISTA/PD-1H immune checkpoint pathways, currently undergoing phase I clinical testing. CA-170 was developed through a rational design and a screening strategy which identified small molecules that could antagonize T cell suppression independently mediated by PD-L1, PD-L2 and VISTA/PD-1H in functional assays.

**Methods**

CA-170 inhibition of the PD-1/PD-L1/2 or VISTA/PD-1H signaling has been inferred though *in vitro* T cell effector function rescue studies using human, monkey or mouse cells stimulated in the presence of inhibitory PD-L1, PD-L2 or VISTA/PD-1H proteins. CA-170 selectivity was tested against the related inhibitory immune checkpoint pathways CTLA-4, LAG-3, BTLA or the immune co-stimulatory B7/CD28 pathway in functional assays. CA-170 *in vivo* antitumor activity and immune stimulatory activity was tested in multiple syngeneic mouse tumor models. Definitive toxicology and pharmacokinetic profiling studies were performed in mouse and cynomolgus monkey.

**Results**

CA-170 exhibits potent immune rescue activity, comparable to that of blocking PD-1 or VISTA/PD-1H antibodies when tested in cell culture assays that measure the proliferation or IFN-γ secretion of T lymphocytes stimulated in the presence of inhibitory PD-L1, PD-L2 or VISTA/PD-1H proteins. CA-170 does not exhibit off target activity against CTLA-4, LAG-3, BTLA pathways or the B7/CD28 pathway in functional assays. In immune competent mice, orally administered CA-170 inhibits the growth of syngeneic tumors, enhances peripheral T cell activation, and promotes the immune activation of tumor infiltrating CD8^+^ T cells in a dose dependent manner. In preclinical safety studies conducted in rodents and non-human primates, orally administered CA-170 shows no signs of toxicity when dosed up to 1000 mg/kg for 28 consecutive days. CA-170 exhibits an oral bioavailability of approximately 40 % and <10 % in mouse and monkey, respectively, and the plasma half-life ranges from approximately 0.5 hours for mouse to approximately 3.25-4.0 hours for cynomolgus monkey.

**Conclusions**

These non-clinical data provide a strong rationale for the continued clinical development of CA-170, the first oral, small molecule immune checkpoint antagonist for the treatment of advanced cancers.

### P220 Combination of local immunotoxins with CTLA-4 blockade eradicates murine tumors by promoting anti-cancer immunity

#### Jasmin Leshem^1^, Xiu-fen Liu^1^, Tapan Bera^1^, Masaki Terabe^1^, Birgit Bossenmaier^2^, Gerhard Niederfellner^2^, Yoram Reiter^3^, Ira Pastan^1^

##### ^1^National Cancer Institute, NIH, Bethesda, MD, USA; ^2^Roche Pharmaceutical Research &Early Development, Discovery Oncology, Innovation Center Penzberg, Roche Diagnostics GmbH, Penzberg, Germany; ^3^Technion Institute, Haifa, Iceland

###### **Correspondence:** Jasmin Leshem (jasmin.leshem@nih.gov)

**Background**

Immune check point blockade therapy using antibodies to cytotoxic T-lymphocyte-associated protein 4 (CTLA-4) benefits only a limited number of cancer patients. Combination therapies are being pursued to augment the immune activation and drug efficacy. SS1P and RG7787 are immunotoxins that consist of an anti-mesothelin antibody fragment genetically fused to a portion of Pseudomonas exotoxin A. We previously observed in patients delayed onset of responses to SS1P treatment that persisted long after discontinuation of the drug. This observation led us to hypothesize that immunotoxins elicit anti-tumor immunity that can be further potentiated by adding anti-CTLA-4 antibodies (aCTLA-4).

**Methods**

To test our hypothesis, we constructed 66C14-M murine breast cancer cell line expressing human mesothelin on its cell surface. The cells were grown in BALB/c mice transgenic for human mesothelin, because they were rejected by wild type mice. RG7787 or SS1P were injected directly into established tumors (average size >80 mm^3^) and aCTLA-4 was administered IP.

**Results**

We found that the combination of aCTLA-4 with RG7787 or SS1P induced complete remissions in 23 out of 38 mice treated (60 %) providing a significant survival benefit compared to mono-therapy (P < 0.001). No cures were obtained when aCTLA-4, RG7787, or SS1P were given separately. In addition, we found that responding mice treated with aCTLA-4 and SS1P had more abundant tumor-infiltrating CD8+ T cells compared to mice treated with aCTLA-4 or SS1P alone (P < 0.05) and that the response was blocked when mice were treated with anti-CD8 antibodies. Furthermore, 22 out of the 23 surviving mice rejected an additional tumor challenge with the same number of 66C14-M or the parental cells (no human mesothelin) implanted 45 days after the mice were cured. These findings point to immune mediated tumor regression. To explore the mechanism responsible for the anti-tumor effect, we combined aCTLA-4 with a mutant RG7787 that is unable to kill 66C14-M cells and found that the survival of mice was not significantly better than that achieved with aCTLA-4 monotherapy. Some bacterial products activate the immune system by receptors that directly recognize microbe associated molecular patterns (MAMPs). However, our result indicates that MAMP recognition does not explain our findings.

**Conclusions**

Combining intra-tumoral injection of immunotoxins with systemic administration of aCTLA-4 induced a high rate of immune mediated tumor regression. Our findings provide the first preclinical evidence to support use of this combination in patients.

### P221 Adjuvant effect of anti-PD-L1 in boosting HER2-targeted T cell adoptive immunotherapy

#### Leiming Xia^1^, Yang Xia^1^, Yangyang Hu^1^, Yi Wang^2^, Yangyi Bao^2^, Fu Dai^2^, Shiang Huang^3^, Elaine Hurt^4^, Robert E Hollingsworth^4^, Lawrence G Lum^5^, Alfred E Chang^1^, Max S Wicha^6^, Qiao Li^7^

##### ^1^University of Michigan, Ann Arbor, MI, USA; ^2^The No.1 People’s Hospital of Hefei, Hefei, Anhui, People’s Republic of China; ^3^Union Hospital, Tongji Medical College, Huazhong University of Science and Technology, Wuhan, Hubei, People’s Republic of China; ^4^MedImmune Inc., Gaithersburg, MD, USA; ^5^University of Virginia Cancer Center, Charlottesville, VA, USA; ^6^University of Michigan Medical School, Ann Arbor, MI, USA; ^7^University of Michigan Medical Center, Ann Arbor, MI, USA

###### **Correspondence:** Qiao Li (qiaoli@med.umich.edu)

**Background**

Adoptive immunotherapy utilizing anti-CD3 x anti-HER2 bispecific antibody (HER2Bi)-armed T cells benefited both HER2^+^ patients and patients with 1 or 2^+^ HER2 expression, ones that would be considered “HER2-negative” by classical criteria. We have also shown that the level of cancer stem cell (CSC) marker ALDH in HER2^+^ breast cancer cells (ALDH^high^HER2^+^) is much higher than that in HER2^−^ breast cancer cells (ALDH^low^HER2^−^), and that in luminal breast cancers that are considered HER2^−^, HER2 is actually selectively expressed in the ALDH^high^ CSC population. These observations might account for the surprising result that HER2Bi-armed T cells, while intended to target HER2, seemed to benefit HER2^−^ patients after adoptive transfer.

**Methods**

We tested the “mouse HER2” (*neu*) expression on ALDH^high^ vs. ALDH^low^ 4 T1 cells (mouse TNBC). For mHER2 targeting in animal models, we generated anti-mouse HER2-CD3 bispecific (mHER2Bi) that binds to mouse HER2 and mouse CD3.

**Results**

HER2Bi-armed T cells used in the clinical trial killed ALDH^high^ human breast CSCs isolated from MCF7 (HER2^−^) tumor significantly more than ALDH^low^ MCF7 cells *in vitro*, while the same HER2Bi-armed T cells killed ALDH^high^ human breast CSCs (ALDH^high^HER2^+^) isolated from BT474 (HER2^+^) tumor equally to ALDH^low^ BT474 cells (ALDH^low^HER2^+^). We also found that mHER2 was selectively expressed in the ALDH^high^ 4 T1 CSC population. These results replicated our findings in human breast cancers that HER2 is selectively expressed on CSCs, even in HER2^−^ murine tumors, such as 4 T1. *In vitro*, the mHER2Bi-armed T cells killed ALDH^high^ 4 T1 CSCs significantly more than ALDH^low^ 4 T1 cells. *In vivo,* adoptive transfer of mHER2Bi-armed T cells for HER2-targeted therapy showed antitumor effect in mHER2^−^ 4 T1-bearing host. Administration of anti-mouse PD-L1 during mHER2Bi-armed T cell adoptive transfer decreased metastases significantly more than the use of either strategy alone.

**Conclusions**

These studies have generated evidence providing proof of principle that due to the selective expression of HER2 on CSCs, HER2-targeted T cell therapy could benefit HER2^−^ hosts as well as HER2^+^ hosts via immune destruction of HER2^+^ CSCs, and use of anti-PD-L1 could significantly boost the efficacy of HER2-targeted T cell therapy.

### P222 Combining IL-6 blockade with novel targeted therapeutics in pancreatic cancer

#### Thomas Mace, Neil Makhijani, Erin Talbert, Gregory Young, Denis Guttridge, Darwin Conwell, Gregory B Lesinski

##### The Ohio State University, Columbus, OH, USA

###### **Correspondence:** Thomas Mace (thomas.mace@osumc.edu)

**Background**

Pancreatic ductal adenocarcinoma (PDAC) is the fourth leading cause of cancer in America with few efficacious therapeutic options other than surgery. PDAC is characterized by dense and heterogeneous stroma that secretes elevated levels of the proinflammatory cytokine interleukin-6 (IL-6). Our laboratory has previously reported that higher IL-6 in PDAC patients is strongly associated with poor overall survival. Additionally, patients with pancreatic and gastrointestinal cancers have the highest incidence of cachexia. This syndrome, characterized by the loss of skeletal muscle and adipose tissue, cannot be reversed by nutritional intervention and is mediated impart by IL-6 signaling. Further, work completed by our group and others have also shown that IL-6 and other factors can promote cross-talk between the STAT3 and MEK pathways. Thus, we hypothesized that IL-6 blockade can be utilized to enhance the efficacy of novel immune or targeted therapeutics (anti-PD-L1 and cobimetinib) in pancreatic cancer.

**Methods**

*In vivo* efficacy studies were conducted with antibodies (Ab) blocking IL-6, in combination with checkpoint immunotherapy (anti-PD-L1) or MEK inhibition (cobimetinib). Experiments were conducted in mice bearing subcutaneous KPC-derived MT5 tumors; orthotopically injected KPC-luciferase expressing tumor cells in the pancreas; and Colon26 tumor bearing CD2F1 mice to determine effects on cancer cachexia.

**Results**

IL-6 blockade combined with anti-PD-L1 (p < 0.02) or cobimetinib (p = 0.007) elicited anti-tumor efficacy in mice bearing subcutaneous KPC derived MT5 tumors, compared to vehicle controls. IL-6 blockade in combination with anti-PD-L1 antibodies limited tumor growth of orthotopic KPC-luciferase expressing tumor cells compared to isotype controls (p = 0.05). As a pancreatic cachexia model is not currently available, we tested IL-6 blockade in combination with cobimetinib on a classically accepted tumor cachexia model (CD2F1 mice bearing Colon26 tumors). Only mice treated with cobimetinib or the combination of IL-6 plus cobimetinib resulted in significant tumor inhibition compared to IL-6 alone or vehicle controls (p < 0.0001). Furthermore, mice administered IL-6 alone or in combination with cobimetinib prevented tumor-induced body weight loss (p < 0.005) and protected lean mass and hind limb muscles as compared to vehicle-treated mice (p < 0.05).

**Conclusions**

These pre-clinical results indicate that inhibition of IL-6 may affect the efficacy of novel targeted therapeutics on tumor progression, immunosuppression, and cachexia in pancreatic cancer.

### P223 Distinct chemokines and chemokine receptors control the trafficking of effector and regulatory cells into melanoma tumors in the setting of combined PD-1 and CTLA-4 blockade

#### Rodney JM Macedo Gonzales, Austin P Huffman, Ximi K Wang, Ran Reshef

##### Columbia Center for Translational Immunology, Columbia University Medical Center, New York, NY, New York, NY, USA

###### **Correspondence:** Rodney JM Macedo Gonzales (rjm2198@cumc.columbia.edu)

**Background**

Pharmacologic blockade of the CTLA-4 and PD-1 immune checkpoint molecules is an effective approach for cancer immunotherapy especially in melanoma, but only a subset of patients respond. Trafficking of immune regulatory cells into the tumor microenvironment creates an immunosuppressive environment which dampens the anti-tumor response. Thus, identifying the mechanisms involved in the trafficking of effector and regulatory cells is critical for the development of strategies that increase effectiveness of checkpoint blockade. We aimed to determine which trafficking molecules are involved in anti-tumor responses by studying both human and murine melanoma.

**Methods**

RNA sequencing data were obtained from 475 melanoma patients (The Cancer Genome Atlas database). Additionally, C57BL/6 mice were subcutaneously injected with 100,000 B16-F10 cells in the flank and sacrificed at day 14 for flow cytometry analysis. Anti-PD-1 + anti-CTLA-4 blocking antibodies or PBS were injected intraperitoneally at days 5, 8 and 11 after tumor inoculation.

**Results**

Analysis of RNA-seq data showed that inflammatory chemokines (CCL2, CCL5, CXCL9, CXCL10) and their receptors (CCR2, CCR5, CXCR3) were overexpressed in human melanoma tumors. Interestingly, unsupervised clustering demonstrated that CCR2, CCR5 and CCL2 were associated with CD68 and CD14 genes while CXCR3, CCL5, CXCL9 and CXCL10 were associated with CD8A, CD8B and T-bet genes. Moreover, immunophenotyping of tumor-infiltrating CD45+ cells from B16-F10 tumor-bearing mice revealed higher levels of CCR2. Interestingly, monocytic myeloid-derived suppressor cells (M-MDSCs) and inflammatory dendritic cells had the highest expression of these receptors. When B16-F10 tumor-bearing mice were treated with anti-PD-1/anti-CTLA-4 antibodies, we observed a significant reduction of tumor size and increased levels of CD45+ cells (p < 0.05), CD8+ T cells (p < 0.05) and increased CD8/Treg ratio (p < 0.01) in comparison to controls; however, the numbers of M-MDSC were not reduced. More importantly, CCR2 and CCR5 were still high within total CD45+ cells (26-30 %) and M-MDSCs (54-71 %) in both treated and control mice. Additionally, dual checkpoint blockade significantly increased the expression of CCR1 (p < 0.05) and CXCR3 (p < 0.05) in CD8+ T cells, without increasing levels of CCR2 and CCR5.

**Conclusions**

Our data suggest that dual checkpoint blockade increases the trafficking of CD8+ T cells into the tumor using the CXCL9/CXCL10-CXCR3 axis but does not affect the CCL2-CCR2 and CCL5-CCR5 axis that are critical for M-MDSCs trafficking into the melanoma microenvironment. These results are important for the development of novel immunotherapy combinations that harness trafficking mechanisms to improve the efficacy of immunotherapies.

### P224 Targeting tumor glutamine metabolism with CB-839 enhances the efficacy of immune checkpoint inhibitors

#### Andy MacKinnon, Jason Chen, Matt Gross, Gisele Marguier, Peter Shwonek, Natalija Sotirovska, Susanne Steggerda, Francesco Parlati

##### Calithera Biosciences, South San Francisco, CA, USA

###### **Correspondence:** Andy MacKinnon (amackinnon@calithera.com)

**Background**

T cell activation and proliferation are metabolically demanding processes that require essential nutrients such as glucose and glutamine. Within the tumor microenvironment, competition between tumor cells and immune cells for limited nutrients can lead to poor T cell activation and suppression of an anti-tumor immune response. Engagement of immune checkpoints such as PD-1 further suppresses T cell activation. While therapeutic blockade of immune checkpoints may partially relieve T cell suppression, low nutrient availability in the tumor microenvironment is expected to limit an optimal immune response. CB-839 is a glutaminase inhibitor currently in phase I oncology trials. CB-839 blocks glutamine consumption by tumors leading to elevated glutamine levels in the tumor microenvironment. Based on the high demand of T cells for glutamine, we hypothesized that CB-839 might synergize with immune checkpoint inhibitors to relieve immune suppression and lead to enhanced anti-tumor immune responses.

**Methods**

*Ex vivo* T cell activation was performed with anti-CD3/CD28 on CD3+ cells isolated from human PBMCs. Changes in mRNA expression after T cell activation was monitored by NanoString analysis. *In vivo* efficacy studies were conducted in syngeneic CT-26 or B16 tumor models.

**Results**

T cell activation in the absence of glutamine inhibited cell proliferation and the expression of cell surface activation markers. Analysis of mRNA expression also showed suppression of normal activation markers and induction of T cell exhaustion markers including PD-1, CTLA-4 and BTLA, suggesting that T cell activation in the absence of glutamine may be sufficient to induce an exhausted phenotype. Previous work showed that CB-839 blocks glutamine consumption in tumors leading to reduced cell proliferation. Surprisingly, CB-839 had only minimal impact on T cell proliferation, highlighting differences in glutamine utilization pathways between tumor cells and T cells. In mouse tumor models, administration of CB-839 elevated tumor glutamine levels, consistent with inhibition of tumor glutaminase. Combination of CB-839 with anti PD-1 or anti PD-L1 in the syngeneic CT-26 colon model augmented tumor regressions relative to checkpoint inhibition alone. CB-839 also enhanced the anti-tumor activity of checkpoint inhibitors in the B16 melanoma model. Depletion of CD8+ T cells from tumor-bearing animals reversed the anti-tumor effects of the combination, confirming an immune-mediated mechanism of action.

**Conclusions**

These data highlight a novel therapeutic approach to treat cancer by selectively targeting tumor metabolism as a means of enhancing the efficacy of checkpoint blockade. Our data provide a rationale for combining CB-839 with immune checkpoint inhibitors in the clinic.

### P225 Arginase inhibitor CB-1158 alleviates immunosuppression and enhances anti-tumor responses as a single agent and in combination with other immunotherapies

#### Amani Makkouk, Mark K Bennett, Jason Chen, Ethan Emberley, Matt Gross, Tony Huang, Weiqun Li, Andy MacKinnon, Gisele Marguier, Silinda Neou, Alison Pan, Jing Zhang, Winter Zhang, Francesco Parlati

##### Calithera Biosciences, South San Francisco, CA, USA

###### **Correspondence:** Amani Makkouk (amakkouk@calithera.com)

**Background**

T cells and natural killer (NK) cells require L-arginine for proliferation. Arginine depletion by arginase in the tumor microenvironment induces immunosuppression and is associated with tumor immune evasion. Arginase is expressed by myeloid-derived suppressor cells (MDSCs) and polymorphonuclear cells (PMNs), and its pharmacological inhibition is expected to restore arginine levels and relieve immunosuppression, leading to anti-tumor immune responses.

**Methods**

We developed CB-1158, a potent and selective small molecule inhibitor of arginase (IC_50_ = 98 nM). The activity of CB-1158 was examined *ex vivo* using immune cells isolated from healthy volunteers or cancer patients, and *in vivo* using murine syngeneic tumor models. Arginase abundance in cancer patient plasma and in tumor tissue microarrays was also examined.

**Results**

In a co-culture system of T cells with PMNs or MDSCs, CB-1158 reverses PMN- or MDSC-mediated immunosuppression by blocking arginine depletion, thereby allowing T cells to proliferate. T cells activated in the presence of PMN-conditioned media show suppressed production of cytokines involved in Th1-type adaptive immunity, and this effect is reversed by the addition of CB-1158. *In vivo,* CB-1158 has high oral bioavailability and is very well tolerated. In tumor-bearing mice, twice daily dosing of CB-1158 causes dose-dependent pharmacodynamic increases in plasma and tumor arginine levels associated with single agent anti-tumor efficacy in multiple syngeneic models. The anti-tumor efficacy of CB-1158 is abrogated in immunocompromised mice or via depletion of either CD8^+^ T cells or NK cells, confirming an immune-mediated mechanism of action. Moreover, CB-1158 enhances CD8^+^ T cell infiltration into tumors and increases expression of Th1 cytokines, T cell and NK cell activation markers, and interferon-inducible genes in the tumor. The immunomodulatory activity of CB-1158 supports the potential of its combination with other immunotherapies and/or standard-of-care therapies. CB-1158 enhances the anti-tumor efficacy of checkpoint inhibitors, including anti-PD-L1 and epacadostat in the B16F10 model. Moreover, CB-1158 enhances the anti-tumor efficacy of standard-of-care therapies such as chemotherapy. To assess the clinical potential of CB-1158, the abundance of arginase in tumors and plasma from cancer patients across multiple cancer histotypes was surveyed. Arginase-expressing PMN infiltrates are abundant in multiple tumor types. Plasma arginase levels are elevated in cancer patients compared to healthy controls, and are associated with decreased plasma arginine.

**Conclusions**

These results support the clinical development of CB-1158, a first-in-class arginase inhibitor, as a novel immunomodulatory agent antagonizing myeloid-mediated immunosuppression. A phase I clinical trial testing the clinical activity of CB-1158 in cancer patients has been initiated.

### P226 Revealing how adoptive T cell transfer into lymphodepleted host and checkpoint blockade therapy work together to treat blood cancers

#### Netonia Marshall, Thomas U Marron, Judith Agudo, Brian Brown, Joshua Brody

##### Icahn School of Medicine at Mount Sinai, New York, NY, USA

###### **Correspondence:** Netonia Marshall (Netonia.marshall@mssm.edu)

**Background**

Blood cancers, with an estimated 160,000 new cases, account for nearly 10 % of all cancer diagnoses and 9.4 % of all cancer deaths this year in the United States. Unfortunately, despite therapeutic advances, the mortality rate still continues to rise. Thus, novel, mechanistically distinct therapies, such as immunotherapy, may have a significant impact particularly in addressing aggressive lymphoma subtypes such as those being modeled in our transplant-based approach. Two classes of immunotherapies that have had great success in treating a wide array of cancers are: checkpoint blockade (e.g., anti-CTLA-4 antibody-based treatments and anti-PD-1 antibody-based treatments) and adoptive T cell (e.g., TIL) transfer lymphocytes into lymphodepleted hosts.

**Methods**

We have developed a novel therapy combining these approaches into 'checkpoint-blockade-primed immunotransplant' comprised of: treatment of tumor-bearing host with anti-CTLA-4 and/or anti-PD-1 antibodies, and splenocyte harvest and transfer to lymphodepleted recipient.

**Results**

Our results show that this combined therapy results in superior anti-tumor immunity compared to either individually as seen by increased production of IFN γ positive T cells. Treatment of both tumor-bearing donor and recipient with anti-PD-1 and anti-CTLA-4 antibodies induces cure of the majority of recipients, in a CD8, NK, and IFNγ-dependent manner, despite the finding that antibody therapy alone (without transplantation and T cell transfer) induces minimal anti-tumor effect. Furthermore, we have demonstrated that T cells exposed to checkpoint blockade and transfer into the lymphopenic hosts demonstrate: greater *in vivo* serum levels of IL-15 and IL-7, higher *ex vivo* levels of IL-15R and IL-7 -receptors expression on CD8s, *in vitro* STAT5 phosphorylation in response to common γ-chain cytokines, *in vivo* proliferation in response to exposure to cognate tumor antigen, and *in vitro* production of IFNγ and TNF production in response to exposure to cognate tumor antigen.

**Conclusions**

Ongoing studies will seek to assess the dependence of the above observations (cytokine production, proliferation, anti-tumor effect) on specific common γ-chain cytokines, the role of the lymphopenia in inducing T cell trafficking to tumor versus organs, and guide development of the immunotransplant model to optimize the amplification of anti-tumor immunity observed.

### P227 Immunomodulatory cytokine blockade in combination with CTLA-4 blockade in murine models of pancreatic cancer

#### Christopher McQuinn, Thomas Mace, Matthew Farren, Hannah Komar, Reena Shakya, Gregory Young, Thomas Ludwug, Gregory B Lesinski

##### The Ohio State University, Columbus, OH, USA

###### **Correspondence:** Christopher McQuinn (Christopher.mcquinn@osumc.edu)

**Background**

Pancreatic cancer remains a significant challenge with 5 year survival rates of less than 7 %. This devastating malignancy is expected to become the second leading cause of cancer death in the United States by 2030. Although effective in other malignancies, there has been a relative paucity of efficacy when immune checkpoint blockade has been applied in pancreatic cancer. We hypothesize this limited efficacy is due to local and systemic alterations in cytokine expression that shape the immune contexture in these patients. Although dysregulated cytokines represent attractive targets in pancreatic cancer, there are limited data to help prioritize among them for future translation. Prior studies from our group demonstrated that plasma interleukin-6 (IL-6), interleukin-10 (IL-10) and circulating CD8 + CTLA-4+ cells were correlated with overall survival in a population of n = 71 treatment naïve metastatic pancreatic cancer patients. We hypothesized that targeting IL-10 and IL-6 would augment the efficacy of antibodies targeting CTLA-4.

**Methods**

*In vivo* efficacy of blocking antibodies against IL-6, IL-10, and CTLA-4 were evaluated in C57BL/6 mice bearing syngeneic, subcutaneous murine pancreatic tumor cells derived from *LSL-Kras* ^*G12D*^ *;LSL- p53* ^*R172H*^ *;Pdx1-Cr* and in a highly aggressive genetically engineered mouse model, harboring *Kras* ^*G12D*^ *; p53* ^*R172H*^ *and Brca2 mutation* (KPC-BRCA2). Relevant immune biomarkers were analyzed using flow cytometry or IHC, as appropriate.

**Results**

*In vivo* studies demonstrated that combined blockade of IL-6 and CTLA-4 significantly decreased the rate of tumor growth in comparison to both isotype control (P = 0.0001) and anti-CTLA-4 alone (P = 0.0207). Treatment with antibody against IL-10, or IL-10 blockade in combination with anti-CTLA-4 slowed tumor growth in comparison to isotype control but were inferior to single agent anti-CTLA-4. FACS analysis of splenocytes from these mice revealed that combined IL-6 and CTLA-4 blockade increased the proportion of circulating CD4+ central memory cells (CD62L + CD44+). Blockade of IL-6 and CTLA-4 in combination, and as single agents, resulted in an increase in circulating Th1 cells while both isotype control and anti-IL-6 had significantly more naïve systemic T cells (CD4+/CD8 + CD62L + CD44-). IHC analysis revealed increased infiltrating CD3+ cells throughout the tumor foci of the combination group in comparison to both single agents and isotype control (all P’s < 0.01). Ongoing analysis will further delineate the proportion and detailed phenotype of infiltrating and systemic immune cells.

**Conclusions**

Antibodies targeting IL-6 but not IL-10 augment the efficacy of anti-CTLA-4 in murine models of pancreatic cancer, modulate T cell infiltration and immune biomarkers to promote Th1 immune responses.

### P228 Improvement of a therapeutic cancer vaccine in mice with the addition of a GITR-ligand fusion protein

#### Y Maurice Morillon^1^, Scott A Hammond^2^, Jeffrey Schlom^1^, John W Greiner^1^

##### ^1^Laboratory of Tumor Immunology and Biology, Center for Cancer Research, National Cancer Institute, Bethesda, MD, USA; ^2^MedImmune LLC, Gaithersburg, MD, USA

###### **Correspondence:**Y Maurice Morillon (yves.morillon@nih.gov)

**Background**

Breaking tolerance mechanisms to mount a durable adaptive immune response within tumors remains one of the preeminent challenges of immuno-oncology. Immunosuppressive hurdles include: 1) suppression from immunoregulatory and tumor cells, 2) insufficient intratumoral “immune space” and survival signals, and 3) exhaustion of tumor specific effector T cells (T_eff_). GITR, an activating receptor belonging to the tumor necrosis factor receptor (TNFR) super family, is constitutively expressed on FoxP3+ regulatory T cells (T_regs_) and to a lesser extent on quiescent T_eff_. Activation upregulates GITR on T_eff_ and T_regs_, which however remains highest among T_regs_. Thus, selectively targeting GITR can deliver activating signals to T_eff_ cells, while depleting high-GITR-expressing T_regs_, and may improve efficacy of a therapeutic cancer vaccine.

**Methods**

Recombinant poxviruses [modified vaccinia Ankara (rMVA-), fowlpox (rF-)] were engineered to express human *CEA* and murine costimulatory molecules, *B7.1*, *ICAM-1* and *LFA-3* (TRICOM); termed rMVA- or rF-*CEA*-TRICOM. A prime-boost strategy was utilized; the priming vaccine utilized rMVA-CEA-TRICOM while rF-CEA-TRICOM provided the boost. The diversified prime-boost vaccine regimen can break tolerance in transgenic mice expressing human *CEA*. GITR was targeted with a fusion protein (GITRL-FP) consisting of the extracellular domain of murine GITR-ligand molecularly fused to a trimerization domain and murine IgG2a-Fc. Murine colon adenocarcinoma cells expressing human *CEA* (MC32A) were implanted subcutaneously in CEA. Tg mice and treated with control IgG2a, GITRL-FP, rMVA/rF-CEA-TRICOM, or rMVA/rF-CEA-TRICOM + GITRL-FP.

**Results**

Initial studies paired twice weekly dosing of GITRL-FP concurrent with MVA/rF-CEA-TRICOM and modest improvements in antitumor effects resulted. GITRL-FP targets GITR expressing cells, delivering activating signals, while the IgG2a-Fc depletes via Fc-mediated effector functions. Investigation into mechanism revealed depletion of T_regs_ and T_eff_. To circumvent the problem of depleting vaccine induced T_eff_, administration of GITRL-FP was switched to a single dose given 2 days prior to vaccine. The short half-life of the fusion protein allowed for temporal intratumoral depletion of both T_regs_ and T_eff_. Single dose GITRL-FP abrogated the immunosuppressive constraints of T_regs_ and created a lymphopenic intratumoral T cell compartment. These events allowed for expansion of T_eff_ in response to MVA-CEA-TRICOM as shown by a 20 % increase of proliferating intratumoral CD4+ T_eff_ compared to GITRL-FP monotherapy, and a 2-fold increase in activated peripheral CD8+ T_eff_. Reduced tumor growth and improved survival was observed comparing combination to GITRL-FP monotherapy. Tumor-free mice were also protected against tumor rechallenge.

**Conclusions**

These data demonstrate the increased efficacy of utilizing targeted depletion of immunosuppression in combination with an immune boosting cancer vaccine.

### P229 Elucidating the role of CD47 in innate lymphoid cell-mediated tumor therapy

#### Pulak R. Nath^1^, Anthony L. Schwartz^1^, Dragan Maric^2^, David D. Roberts^1^

##### ^1^National Cancer Institute, National Institutes of Health, Bethesda, MD, USA; ^2^National Institute of Neurological Disorders and Stroke, National Institutes of Health, Bethesda, MD, USA

###### **Correspondence:** Pulak R. Nath (pulak.nath@nih.gov)

**Background**

CD47 is a ubiquitous cell surface receptor that interacts with the secreted protein thrombospondin-1 and its counter-receptor SIRPα on phagocytes and antigen presenting cells (APCs). CD47 is highly expressed across many cancer types, hence, representing a potential target for therapeutic intervention. We recently reported a direct role for thrombospondin-1/CD47 signaling on cytotoxic T lymphocytes (CTL) to limit target tumor cell killing [1]. Many tumors are not sufficiently immunogenic to induce protective adaptive immunity. However, innate lymphoid cells (ILCs) may also play functional roles in tumor regression [2]. Here we evaluated the role of CD47 in NK and other ILCs homeostasis within the lymphoid organs as well as among tumor infiltrating lymphocytes.

**Methods**

We analyzed tumor infiltration of NK and other ILCs following antisense suppression of CD47 alone or in combination with anti-CTLA-4 blockade. C57Bl/6 mice were injected with B16F10 melanoma in the hind limb, and once the tumors reached an average of 100 mm^3^, mice were treated with CD47-morpholino, anti-CTLA-4, or combined treatments.

**Results**

Treatment of mice with CD47-morpholino increased the frequencies of splenic Lin^−^CD3^−^NK1.1^+^ and Lin^−^CD3^−^CD127^+^ populations. Studies using CD47-null mice further validated this result. We observed higher granzyme B and perforin mRNA expression in the CD3^−^CD4^−^CD8^−^ cells compared to the CD8^+^ cells from spleens of CD47-null mice. Image cytometric analysis revealed that these are mononuclear lymphocytes. These cells express higher eomes and T-bet, but lower Gata3 and Rorγt as compared to their CD4^+^ counterparts, suggesting that they fall within the NK and ILC1 lineages. Indeed, lineage-depleted splenocytes from CD47-null mice showed higher frequencies of NK1.1^+^ and CD127^+^ cells compared to wildtype littermate controls. These cells infiltrated into tumors of B16F10-bearing mice, and their numbers further increased following treatment with a combination of CD47-morpholino and anti-CTLA-4 antibody, which resulted in enhanced therapeutic benefits.

**Conclusions**

Our data suggest that deficiency of CD47 in the tumor microenvironment or therapeutic blockade increases subtypes of ILCs with potent anti-tumor properties. The mechanism by which CD47 controls the homeostatic balance of ILCs or their development remains to be determined.

**References**

1. Soto-Pantoja DR, *et al.*: **CD47 in the tumor microenvironment limits cooperation between antitumor T-cell immunity and radiotherapy**. *Cancer Res* 2014, **74**:6771–6783.

2. Dadi S, *et al.*: **Cancer immunosurveillance by tissue-resident innate lymphoid cells and innate-like T cells.** *Cell* 2016, **164**:365–377.

### P230 Restoration of antitumor effectiveness of PD-1 inhibition in immunotherapy-resistant “cold” tumors by combinatorial treatment enhancing the numbers of tumor-specific CTLs in tumor tissues

#### Nataša Obermajer^1^, David Bartlett^1^, Pawel Kalinski^2^

##### ^1^Department of Surgery, University of Pittsburgh, Pittsburgh, PA, USA; ^2^Department of Surgery; University of Pittsburgh Cancer Institute; Department of Infectious Diseases and Microbiology, University of Pittsburgh, Pittsburgh, PA, USA

###### **Correspondence:** Nataša Obermajer (obermajern2@upmc.edu)

**Background**

Intratumoral accumulation of effector type-1 T cells (CTLs) is an independent prognostic factor of survival of patients with many cancer types and is required for the effectiveness of checkpoint blockade therapies.

**Methods**

In this study, we have tested whether the enhancement of the numbers of tumor-infiltrating CTLs by DC vaccines together with combinatorial reprogramming of tumor-associated chemokines, can be used to convert the nominally checkpoint-resistant “cold” tumors into PD-1-sensitive ones.

**Results**

In colorectal and ovarian cancer (transplantable MC38 and ID8 models) bearing mice, we observed only marginal therapeutic effect of PD-1 inhibition alone or combined with DC vaccine. However, combinatorial reprogramming of tumor-associated chemokines, using TLR3 ligand polyI:polyC_12_U, interferon-a and COX2 blockers, resulted in a striking increase in the numbers of tumor-infiltrating CTLs recognizing cancer-associated antigens and allowed for the conversion of these immunotherapy-resistant tumors into sensitive ones, resulting in high numbers of long-term surviving animals.

**Conclusions**

This combinatorial DC-based vaccination approach may be used to induce specific immune cells against different tumor-relevant antigens and may be included as a component of anti-tumor therapeutic approaches that, by themselves do not induce new effector cells, nor promote their intratumoral accumulation.

### P231 Immune activation by PEGylated human IL-10 (AM0010) and anti-tumor activity in renal cancer alone and in combination with anti-PD-1

#### Aung Naing^1^, Kyriakos P Papadopoulos^2^, Karen A Autio^3^, Deborah J Wong^4^, Manish Patel^5^, Gerald Falchook^6^, Shubham Pant^7^, Patrick A Ott^8^, Melinda Whiteside^9^, Amita Patnaik^2^, John Mumm^9^, Filip Janku^1^, Ivan Chan^9^, Todd Bauer^12^, Rivka Colen^1^, Peter VanVlasselaer^9^, Gail L Brown^9^, Nizar M Tannir^1^, Martin Oft^9^, Jeffrey Infante^10^

##### ^1^University of Texas MD Anderson Cancer Center, Houston, TX, USA; ^2^South Texas Accelerated Research Therapeutics, LLC, San Antonio, TX, USA; ^3^Memorial Sloan Kettering Cancer Center, New York, NY, USA; ^4^UCLA, Los Angeles, CA, USA; ^5^Sarah Cannon Research Institute/Florida Cancer Specialists, Sarasota, FL, USA; ^6^SCRI at HealthONE, Denver, CO, USA; ^7^Oklahoma University, Oklahoma Citu, OK, USA; ^8^Dana-Farber Cancer Institute, Boston, MA, USA; ^9^ARMO BioSciences, Redwood City, CA, USA; ^10^Sarah Cannon Research Institute, Nashville, TN, USA

###### **Correspondence:** Martin Oft (martin.oft@armobio.com)

**Background**

IL-10 is regarded as an anti-inflammatory cytokine, but it is at least equally important for the cytotoxicity and proliferation of antigen activated CD8+ T cells. Activation of CD8+ T cells through the T cell receptor elevates IL-10 receptors and PD-1 on the cells. This provides the mechanistic rationale for combining AM0010 and anti-PD-1 for the treatment of cancer patients. A phase I clinical trial investigated the tolerability and anti-tumor activity of AM0010 alone and in combination with anti-PD-1 immune checkpoint inhibitors.

**Methods**

Patients with advanced RCC were treated with AM0010 (daily SC) alone or in combination with pembrolizumab (q3wk IV) or nivolumab (q2wk IV). Tumor responses were monitored following irRC. Immune responses were measured by analysis of serum cytokines, and the activation and clonality of T cells in peripheral blood mononuclear cells. Nineteen patients with RCC (15 evaluable), were treated with AMO010 alone (20 mg/kg). Eight patients were treated in combination with pembrolizumab (2 mg/kg) and 15 patients with nivolumab (3 mg/kg).

**Results**

AM0010 alone or in combination with anti-PD-1 was tolerated well (observation periods exceeding 16 months). All TrAEs were transient and TrAEs leading to study discontinuation were not observed. There was no colitis, pneumonitis, or endocrine disruptions. G3/4 TrAEs in monotherapy included anemia (9), hypertriglyceridemia (3), thrombocytopenia (2), ALT/AST increase (2) and fatigue (2). AM0010 combination with anti-PD-1 did not increase TrAEs. Objective responses (PR/CR) were observed in 4 of 15 evaluable RCC patients in monotherapy (27 %), in 4 of 8 patients in AM0010/pembrolizumab (50 %). Progression-free survival (PFS) was 3 and 9.4 months, respectively. The AM0010/nivolumab cohort is currently in progress. AM0010 alone and also in combination with anti-PD-1 increased Th1 cytokines (IL-18, IFNg, TNFa), CD8+ T cell associated effector molecules such as FasL and LymphotoxinB as well as cytokines stimulating T cell proliferation (IL-4, IL-7). As a result, the number and proliferation of activated, PD-1+/LAG-3+ CD8+ T cells in the blood of patients were increased on AM0010. In contrast, the proliferation of FoxP3+ Tregs and TGFb was decreased. AM0010 alone or with anti-PD-1 induced oligoclonal expansion of T cell clones in the blood without affecting total lymphocyte counts. In particular, selected T cells clones previously not detected in the blood of patients before treatment were strongly expanded (*de novo* amplification).

**Conclusions**

AM0010 alone or in combination with anti-PD-1 is well-tolerated. The clinical activity and the observed CD8+ T cell activation encourages the continued exploration of AM0010 in phase III studies.

**Trial Registration**

ClinicalTrials.gov identifier NCT02009449.

**Fig. 18 (abstract P231).**
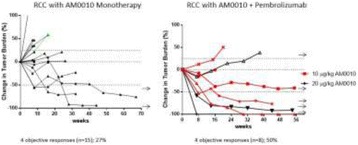
See text for description

**Fig. 19 (abstract P231).**
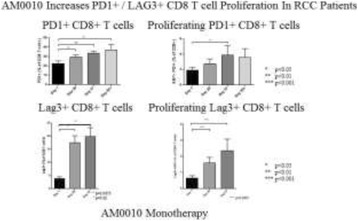
See text for description

### P232 Initial experience administering BMS-986016, a monoclonal antibody that targets lymphocyte activation gene (LAG)-3, alone and in combination with nivolumab to patients with hematologic and solid malignancies

#### Evan Lipson^1^, Ajay Gopal^2^, Sattva S Neelapu^3^, Philippe Armand^4^, Stephen Spurgeon^5^, John P Leonard^6^, F Stephen Hodi^4^, Rachel E Sanborn^7^, Ignacio Melero^8^, Thomas F Gajewski^9^, Matthew Maurer^10^, Serena Perna^10^, Andres A Gutierrez^11^, Raphael Clynes^10^, Priyam Mitra^10^, Satyendra Suryawanshi^10^, Douglas Gladstone^1^, Margaret K Callahan^12^

##### ^1^Sidney Kimmel Comprehensive Cancer Center, Johns Hopkins University School of Medicine, Baltimore, MD, USA; ^2^Seattle Cancer Care Alliance, University of Washington, Seattle, WA, USA; ^3^University of Texas MD Anderson Cancer Center, Houston, TX, USA; ^4^Dana-Farber Cancer Institute, Harvard University, Boston, MA, USA; ^5^Center for Hematologic Malignancies, Oregon Health and Sciences University, Portland, OR, USA; ^6^New York Presbyterian Hospital, Weill Cornell Medical College, New York, NY, USA; ^7^Robert W. Franz Cancer Research Center, Earle A. Chiles Research Institute, Providence Cancer Center, Portland, Oregon, USA, Portland, OR, USA; ^8^Center for Applied Medical Research (CIMA), University of Navarra, Pamplona, Navarra, Spain; ^9^University of Chicago Medical Center, Chicago, IL, USA; ^10^Bristol-Myers Squibb, Princeton, NJ, USA; ^11^Bristol-Myers Squibb, Lawrence Township, NJ, USA; ^12^Memorial Sloan Kettering Cancer Center, New York, NY, USA

###### **Correspondence:** Evan Lipson (evanlipson@jhmi.edu)

**Background**

LAG-3 is a transmembrane receptor that negatively regulates T cell activation. Signaling through LAG-3 and other T cell inhibitory receptors, including programmed death-1 (PD-1), can lead to T cell exhaustion and is a mechanism of immune escape for tumors. Preclinical data suggest that simultaneous blockade of LAG-3 and PD-1 may function synergistically to restore T cell activation and mediate tumor regressions. Here, we describe preliminary first-in-human phase I/IIa data for BMS-986016, a fully human IgG4 monoclonal antibody that targets LAG-3, alone and in combination with nivolumab (anti-PD-1) in patients with advanced B cell malignancies or solid tumors.

**Methods**

Sequential cohorts received BMS-986016 ± nivolumab every 14 days in 56-day cycles during dose escalation or expansion until disease progression, completion of 12 cycles, or prohibitive toxicity. Primary objectives included safety and tolerability.

**Results**

**Fig. 20 (abstract P232).**
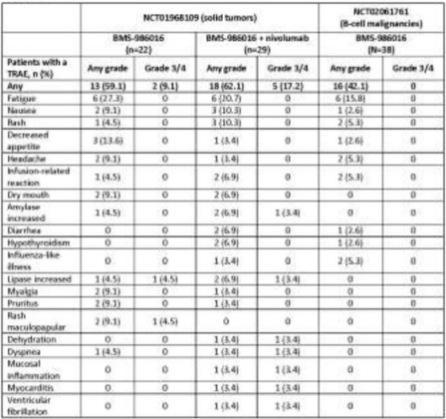
TRAEs reported in > 2 patients or any TRAE ≥ grade 3 reported in patients treated with BMS-986016 ± nivolumab

As of May 2016, 89 patients had received BMS-986016 alone (20 mg [n = 8], 80 mg [n = 13], 240 mg [n = 24], or 800 mg [n = 15]) or with nivolumab (BMS-986016/nivolumab; 20/80 mg [n = 7], 20/240 mg [n = 9], 80/240 mg [n = 9], or 240/240 mg [n = 4]). The pharmacokinetic characteristics of BMS-986016 were assessed across dose levels in patients treated with monotherapy and combination therapy. Anti-drug antibody assessments suggested low immunogenicity. Increases in peripheral blood T cell LAG-3 receptor occupancy (RO; 74-99 %) were observed with escalating BMS-986016 dose and exposure. The maximum tolerated dose (MTD) was not reached with BMS-986016 monotherapy; evaluations to determine the MTD for the combination are ongoing. Infrequent and manageable treatment-related adverse events (TRAEs) were observed across monotherapy doses (Fig. 20), and included toxicities typically associated with immune checkpoint blocking agents. DLTs among patients receiving combination therapy included grade (G)3 mucositis, G4 ventricular fibrillation, G4 elevated lipase, and G4 myocarditis. Most TRAEs were grade 1–2. TRAEs leading to discontinuation of therapy were reported in 3 % (BMS-986016) and 14 % (BMS-986016 + nivolumab) of patients. There were no treatment-related deaths. Objective tumor regression was observed with LAG-3 monotherapy, and with combination therapy in PD-1-naive patients and in patients with disease progression on nivolumab monotherapy.

**Conclusions**

BMS-986016 monotherapy was well tolerated at the dose levels tested. Emerging data characterizing the safety of the combination will be presented. BMS-986016 ± nivolumab demonstrated biological activity as evidenced by toxicities characteristic of immune checkpoint blockers and objective tumor regressions. These preliminary data support the ongoing evaluation of this combination in patients with solid tumors and hematologic malignancies.

**Trial Registration**

ClinicalTrials.gov identifier NCT02061761 and NCT01968109.

### P233 The effects of combination treatment of IMM-101, a heat-killed whole cell preparation of Mycobacterium obuense (NCTC 13365) with checkpoint inhibitors in pre-clinical models

#### James Crooks^1^, Sheila Brown^1^, Audrey Gauthier^2^, Marc Hillairet de Boisferon^2^, Andrew MacDonald^1^, Laura Rosa Brunet^3^

##### ^1^MCCIR, University of Manchester, Manchester, England, UK; ^2^Oncodesign, Dijon, Bourgogne, France; ^3^Immodulon Therapeutics Ltd, Uxbridge, England, UK

###### **Correspondence:** Rosa Brunet (lrb@immodulon.com)

**Background**

While harnessing the power of the immune system to control cancer is becoming established as an effective way of treating patients, it has become increasingly clear that transformed cells exploit a number of mechanisms to escape such control. Hence, while the clinical use of checkpoint inhibitors (CPI) has yielded significant success, there is mounting evidence to suggest that combination treatment of CPI with immunomodulating therapies may further benefit cancer patients. Immodulon Therapeutics is developing IMM-101, an immunotherapeutic agent based on a heat-killed whole cell preparation of *Mycobacterium obuense* (NCTC 13365), which modulates systemic immune responses, as an adjunctive immunotherapy for cancer. Based on exposure data in over 300 patients, alone and in combination, IMM-101 is well-tolerated. Additionally, extended overall survival and progression-free survival were observed in IMAGE-1, a randomized open-label, phase II, first-line, proof of concept study (NCT01303172), in combination with gemcitabine in advanced pancreatic ductal adenocarcinoma.

**Methods**

We found that *in vitro* exposure of IMM-101 primes *in vitro* generated murine dendritic cells (DC) and human monocyte-derived DC in a dose dependent manner and functionally affects DC by enhancing their ability to process and present antigen. Moreover, IMM-101 activated DC promote T cell secretion of IFN-γ following re-stimulation of draining lymph node cell preparations, 7 days after adoptive transfer of IMM-101 primed DC into naïve recipient mice. We also investigated whether the effects of IMM-101 on innate and adaptive immune responses indeed improve on the therapeutic benefit of CPI treatment (anti-CTLA-4 or anti-PD-1) in two murine xenograft models using B16-F10, a mouse melanoma cell line, and EMT6, a mouse breast cell line.

**Results**

We assessed effects on tumor burden and local and systemic immunological bias in treated mice. We report a significant benefit from combination treatment of CPI and IMM-101 on tumor burden. We also observed significant change to the CD8^+^/Treg ratio at the tumor site. We performed *in vitro* stimulation (antigenic as well as polyclonal) of immune cells present at the tumor site, in the draining lymph nodes and in the spleen. We report results at different time points over the course of the disease.

**Conclusions**

On the basis of these promising results, formal clinical evaluation of IMM-101 in combination treatment with anti-PD-1 treatment is being undertaken (EudraCT identifier 2016-001459-28).

### P234 Intrathecal AAV9.trastuzumab for both tumor prophylaxis and treatment extends survival in a murine xenograft model of HER2+ human breast cancer brain metastasis

#### William T. Rothwell, Peter Bell, James M. Wilson

##### University of Pennsylvania Perelman School of Medicine, Philadelphia, PA, USA

###### **Correspondence:** William T. Rothwell (rothw@mail.med.upenn.edu)

**Background**

Breast cancer brain metastases (BCBM) occur in up to 14.3 % of patients with human epidermal growth factor receptor 2 positive (HER2+) primary tumors [1]. Intravenous trastuzumab (anti-HER2 monoclonal antibody (mAb), Herceptin®) extends survival in patients with HER2+ systemic disease but does not cross the blood brain barrier (BBB) to treat HER2+ BCBM effectively [2]. Intrathecal (IT) trastuzumab can extend survival in patients with HER2+ BCBM [3] but requires regular IT infusions which carry risks and can compromise quality of life. Gene therapy offers a one-shot solution for mAb delivery across the BBB. Adeno-associated viral vectors, particularly serotype 9 (AAV9), can safely and efficiently deliver exogenous genes (transgenes) to central nervous system tissues after a single IT administration, resulting in constitutive, long-term expression of the transgene product [4].

**Methods**

We characterize a xenograft model of HER2+ BCBM using BT474.M1 human ductal carcinoma cells injected stereotaxically into the brain parenchyma of Rag1−/− mice. AAV9.trastuzumab is delivered IT as tumor prophylaxis (at least 21 days before tumor administration) or as tumor treatment (3 days post tumor administration).

**Results**

Median survival (MS) of Rag1−/− mice receiving IT AAV9.trastuzumab tumor prophylaxis (MS = 111 days, n = 7) is significantly greater after tumor administration than mice receiving vehicle (MS = 48.5 days, n = 8, p = 0.0012*), AAV9 expressing an irrelevant antibody (MS = 54.5 days, n = 10, p = 0.0027*), or AAV9 without a transgene (MS = 50 days, n = 4, p = 0.0069*). MS of mice bearing tumors treated with IT AAV9.trastuzumab (MS = 82 days, n = 6) is significantly greater than controls receiving vehicle (MS = 61 days, n = 7, p = 0.002*). *Log-rank (Mantel-Cox) test.

**Conclusions**

IT AAV9.trastuzumab as both tumor prophylaxis and treatment increases survival in a murine xenograft model of HER2+ BCBM, thus showing promise as HER2+ BCBM treatment and, more broadly, as a prophylactic measure for patients with HER2+ primary disease to extend survival in the case of BCBM.

**References**

1. Kennecke H, *et al.*: **Metastatic behavior of breast cancer subtypes**. *J Clin Oncol* 2010, **28**:3271–3277.

2. Koo T, Kim I: **Brain metastasis in human epidermal growth factor receptor 2-positive breast cancer: from biology to treatment**. *Radiat Oncol J* 2016, **34**:1–9.

3. Zagouri F, Sergentanis T: **Intrathecal administration of trastuzumab for the treatment of meningeal carcinomatosis in HER2-positive metastatic breast cancer: a systematic review and pooled analysis.** *Breast Cancer Res* 2013, **139**:13–22.

4. Hinderer C, *et al.*: **Widespread gene transfer in the central nervous system of cynomolgus macaques following delivery of AAV9 into the cisterna magna**. *Mol Ther Methods Clin Dev* 2014, **1**:14051.

### P235 Immunotherapy of head and neck squamous cell cancers with synthetic TLR agonists and checkpoint inhibitors in preclinical models

#### Fumi Sato-Kaneko^1^, Shiyin Yao^1^, Shannon S. Zhang^2^, Dennis A. Carson^1^, Cristina Guiducci^2^, Robert L. Coffman^2^, Kazutaka Kitaura^3^, Takaji Matsutani^3^, Ryuji Suzuki^3^, Tomoko Hayashi^1^, Ezra E.W. Cohen^1^

##### ^1^Moores Cancer Center, University of California, San Diego, La Jolla, CA, USA; ^2^Dynavax Technologies, Berkeley, CA, USA, Berkeley, CA, USA; ^3^Repertoire Genesis Incorporation, Osaka, Japan, Ibaraki, Osaka, Japan

###### **Correspondence:** Fumi Sato-Kaneko (fukaneko@ucsd.edu)

**Background**

Head and neck squamous cell cancers (HNSCC) constitute the sixth leading cancer by incidence worldwide. Though PD-1/PD-L1 blockade is effective in some patients, the majority do not benefit. We examined combination therapy with anti-PD-1 and synthetic agonists of toll-like receptors (TLR)7 and TLR9 in mouse models representing human papilloma virus (HPV)-positive and HPV-negative HNSCC, respectively. We hypothesized that the intratumoral treatment with TLR agonists could activate innate immune cells in the tumor microenvironment and enhance tumor specific adaptive immunity. Furthermore, this would be synergistic with checkpoint inhibitors that release negative signals on tumor infiltrating CD8^+^ T cells.

**Methods**

Syngeneic tumor mouse models, SCC-7 cells (HPV-negative)/C3H background and MEER cells (HPV-positive)/C57BL/6 background, were used. Mice were implanted with tumor cells subcutaneously into opposite flanks. Treatments were started with intratumoral injections into only the right side with TLR7 or TLR9 agonists with or without intraperitoneal injections of anti-PD-1 mAb. Lymphocytes were isolated from tumors and spleens on days 13 and 21 post tumor implantation, and were analyzed using flow cytometry. The T cell receptor (TCR) repertoire of CD8^+^ T cells in the tumor and the spleen was evaluated by unbiased high throughput quantitative sequencing.

**Results**

**Fig. 21 (abstract P235).**
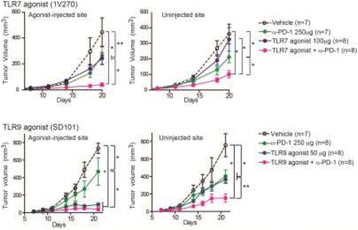
Therapeutic effect of intratumoral TLR7 and TLR9 agonist and anti-PD-1 in HPV negative HNC

**Fig. 22 (abstract P235).**
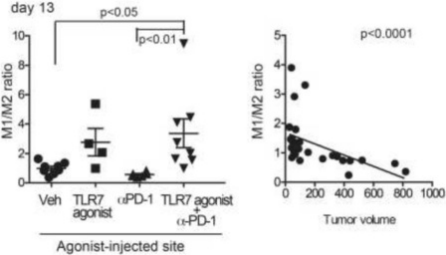
Increased M1/M2 ratio by combination therapy with TLR7 agonist and anti-PD-1 in HPV negative HNC

**Fig. 23 (abstract P235).**
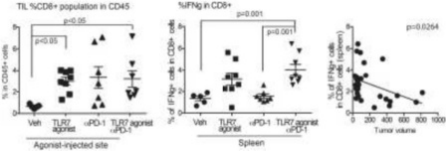
Increased IFNγ+ activated CD8+ T cell population by combination therapy

**Fig. 24 (abstract P235).**
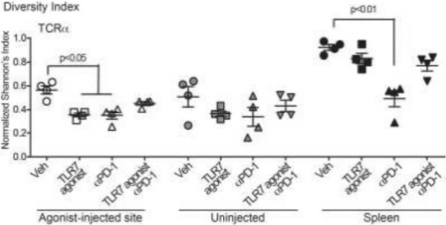
Anti-PD-1 increased clonal expansion of splenic CD8+ T cells

In both HPV-negative and HPV-positive models, the combination therapies of intratumoral TLR7 or TLR9 agonists with anti-PD-1 suppressed tumor progression both at agonist-injected and uninjected sites (abscopal-like effect) (Fig. 21). In the HPV-negative model, the combination treatment with TLR7 agonists and anti-PD-1 increased the M1/M2 ratio in CD11b^+^F4/80^+^ tumor infiltrating macrophages (Fig. 22). *Ex vivo* treatment with TLR7 agonist upregulated the expression of costimulatory molecules CD40, CD80, and decreased the expression level of CD206 (M2-macrophage marker). The combination therapy with TLR7 agonist increased the frequency of CD8^+^ T cells in both sides of tumors and spleen. Elevated IFNγ^+^ activated T cell population was observed in mice treated with the TLR7 ligand and anti-PD-1 therapy (Fig. 23). TCR repertoire analysis showed anti-PD-1 increased clonal expansion of splenic CD8^+^ T cells (Fig. 24).

**Conclusions**

The combination therapy with TLR agonists and anti-PD-1 suppressed progression of tumors in both injected and distant sites by two different mechanisms of action; clonal expansion of low frequency CD8^+^T cell population by anti-PD-1, and recruitment and activation of tumor specific T cells by intratumoral treatment with TLR ligands.

**Acknowledgements**

We thank Dr. John Lee in Sanford Research, who kindly provided us with HNC cells. This work was supported by the Fernanda and Ralph Whitworth Immunotherapy Foundation.

### P236 Modulating the intra-tumor immune balance through combinatorial blockade of CSF-1R and PD-L1 enhances anti-tumor efficacy

#### David Schaer^1^, Yanxia Li^1^, Julie Dobkin^1^, Michael Amatulli^1^, Gerald Hall^1^, Thompson Doman^2^, Jason Manro^2^, Frank Charles Dorsey^2^, Lillian Sams^2^, Rikke Holmgaard^1^, Krishnadatt Persaud^1^, Dale Ludwig^1^, David Surguladze^1^, John S Kauh^3^, Ruslan Novosiadly^1^, Michael Kalos^1^, Kyla Driscoll^1^

##### ^1^Eli Lilly and Company, New York, NY, USA, ^2^Eli Lilly and Company, Indianapolis, IN, USA; ^3^Eli Lilly and Company, Bridgewater, NJ, USA

###### **Correspondence:** David Schaer (schaer_david@lilly.com)

**Background**

Multiple mechanisms are involved in establishing an immunosuppressive tumor microenvironment. Although blockade of the PD-1/L1 axis alone has led to durable clinical responses in multiple malignancies, the majority of patients do not receive or maintain clinical benefit from monotherapy. Colony stimulating factor receptor 1 (CSF-1R) expressing tumor associated macrophages (TAMs) have been implicated as a poor prognostic factor in many cancers. TAMs and other suppressive myeloid cells may represent an additional suppressive axis present in many malignancies where PD-1/L1 blockade has shown some or little activity. CSF-1R has been implicated for maintaining TAM function and viability in tumor tissues, making it an attractive target to modulate TAM and possibly myeloid mediated suppression in cancer. As the CSF-1R Inhibitor LY3022855 has recently entered clinical testing in combination with PD-L1 blockade, it is important to understand how inhibiting two immune suppressive mechanisms will alter immune function to help guide rational clinical development.

**Methods**

To study and understand the immune modulating effects of CSF-1R inhibition, we developed an anti-mouse CSF-1R surrogate antibody CS7. CS7 blocks CSF-1 binding to CSF-1R and inhibits *in vitro* proliferation and differentiation of macrophages and depletes tissue resident macrophages *in vivo*.

**Results**

Monotherapy treatment with CS7 causes intra-tumor depletion of ~50-60 % of F4/80+ TAMs leading to a modest delay in tumor growth. This reduction was associated with an increased intra-tumor immune inflammation signature and reduced inhibitory metabolites, highlighting the role TAMs play suppressing the immune response inside the tumor microenvironment. Combining CSF-1R blockade with anti-PD-L1 enhances the control of tumor growth, displaying a late combinatorial effect leading to complete regressions in the majority of mice (~60 %). Mice achieving complete regressions developed immunologic memory resisting rechallenge over 60 days after cessation of therapy. Intra-tumor gene expression analysis demonstrated a synergistic increase in T cell activation and reduction of immune suppression late in the response, correlating with the time point of increased efficacy. Effects of CS7 were dose-dependent, suggesting that while lower doses of CS7 are able to cause TAM depletion, modulation of the microenvironment requires more complete block of CSF-1R.

**Conclusions**

Our results demonstrate that combination of CSF-1R blockade with PD-L1 checkpoint inhibition alters the tumor microenvironment in favor of enhanced immune activation. In addition, our data imply that the mechanism of CSF-1R blockade immunotherapy may extend beyond reduction of intra-tumor macrophages.

### P237 A combination study of an intravenously delivered oncolytic virus, coxsackievirus A21 in combination with pembrolizumab in advanced cancer patients: phase Ib KEYNOTE 200 (STORM study)

#### Hardev Pandha^1^, Christy Ralph^2^, Kevin Harrington^3^, Brendan Curti^4^, Rachel E Sanborn^5^, Wallace Akerley^6^, Sumati Gupta^6^, Alan Melcher^7^, David Mansfield^7^, David R Kaufman^8^, Emmett Schmidt^8^, Mark Grose^9^, Bronwyn Davies^9^, Roberta Karpathy^9^, Darren Shafren^9^

##### ^1^University of Surrey, Guildford, England, UK; ^2^St James University Hospital, Leeds, England, UK; ^3^Institute for Cancer Research, London, England, UK; ^4^Providence Cancer Center, Portland, OR, USA; ^5^Robert W. Franz Cancer Research Center, Earle A. Chiles Research Institute, Providence Cancer Center, Portland, Oregon, USA; ^6^Huntsman Cancer Institute, Salt Lake City, UT, USA; ^7^Institute for Cancer Research, London, England, UK; ^8^Merck & Co., Inc., Kenilworth, NJ, USA; ^9^Viralytics Ltd., Sydney, New South Wales, Australia

###### **Correspondence:** Darren Shafren (darren.shafren@viralytics.com)

**Background**

Coxsackievirus A21 (CVA21, CAVATAK) is a naturally-occurring ICAM-1 targeted oncolytic immunotherapeutic virus. Pembrolizumab is a human programmed death receptor-1 (PD-1) blocking antibody that has yielded significant solid tumor responses via reversal of tumor induced T cell suppression. Preclinical studies in immune-competent mouse models of non-small cell lung cancer (NSCLC) and melanoma confirmed that combinations of i.v. CVA21 + anti-PD-1 mAbs mediated significantly greater antitumor activity compared to use of either agent alone. We postulate that the combination of CVA21 + pembrolizumab may translate to a similar benefit in the clinic. We describe a phase Ib study assessing safety and efficacy of IV CVA21 ± pembrolizumab in advanced cancer patients (pts).

**Methods**

The phase I STORM (systemic treatment of resistant malignancies; KEYNOTE 200) primary objectives are to assess dose-limiting toxicities (DLT) of CVA21 ± pembrolizumab. Secondary objectives are to assess ORR by irRECIST 1.1 criteria, PFS, and OS. Treatment Part A: pts were infused with CVA21 in 100 mL saline in Cohort 1 (n = 3), at a dose of 1 x 10^8^ TCID_50_, in Cohort 2 (n = 3) at a dose of 3 x 10^8^ TCID_50_ and in Cohort 3 (n = 10) at a dose of 1 x 10^9^ TCID_50_ on study days 1, 3, 5, 22 and Q3W for 6 additional infusions. Part A enrollment is complete. Treatment Part B: pts are infused with CVA21 in 100 mL saline + pembrolizumab. In Cohort 1 (n = 3), CVA21 is administered at a dose of 1 x 10^8^ TCID_50_, in Cohort 2 (n = 3) at a dose of 3 x 10^8^ TCID_50_ and in Cohort 3 (n = ~80) at a dose of 1 x 10^9^ TCID_50_ on study days 1, 3, 5, 8, 29, and Q3W for 6 additional infusions. Pembrolizumab is given in all cohorts at 200 mg IV Q3W from Day 8 for up to 2 years. Treatment with CVA21 ± pembrolizumab will continue until confirmed CR or PD (whichever comes first) per irRECIST 1.1 or DLT. Part B, Cohort 1 enrollment is complete.

**Results**

IV delivery of CVA21 to all patients in Part A was generally well tolerated, with no Grade 3 or 4 product-related AE’s.

**Conclusions**

CVA21 tumor targeting in patients with melanoma, NSCLC, and bladder cancer patients in Part A Cohort 3 was confirmed by detection of CVA21 viral RNA in tumor biopsies at study Day 8.

**Trial Registration**

ClinicalTrials.gov identifier NCT02043665.

### P238 Mutational status of p53 can influence its recognition by human T cells

#### Katerina Shamalov, Cyrille Cohen

##### Bar Ilan University, Ramat Gan, HaMerkaz, Iceland

###### **Correspondence:** Katerina Shamalov (kate.shamalov@gmail.com)

**Background**

p53 was reported to be an attractive immunotherapy target because it is mutated in approximately half of human cancers, resulting in its inactivation and often accumulation in tumor cells. Peptides derived from p53 are presented by class I MHC molecules and may act as tumor-associated epitopes which could be targeted by p53-specific T cells. Interestingly, it was recently shown that there is a lack of significant correlation between p53 expression levels in tumors and their recognition by p53-TCR transduced T cells.

**Methods**

To better understand the influence of the mutational status of p53 on its presentation by the MHC system and on T cell anti-tumor reactivity, we generated several mutant p53 constructs and expressed them in HLA-A2+/p53- cells. Upon co-culture with p53-specific T cells, we measured the specific recognition of p53-expressing target cells by means of cytokine secretion, marker upregulation and cytotoxicity, and in parallel determined p53 expression levels by intracellular staining. We also examined the impact of mutant p53 expression on cell cycle dynamics and on the expression levels of the pro-apoptotic protein caspase-3.

**Results**

Our results show that selected p53 mutations altering protein stability can modulate p53 presentation to T cells, leading to a differential immune reactivity inversely correlated to measured p53 protein levels.

**Conclusions**

Thus, p53 may behave differently than other classical tumor antigens and its mutational status should therefore be taken into account when elaborating immunotherapy treatments of cancer patients targeting p53.

### P239 Fc gamma receptor IV mediated depletion of tumor infiltrating regulatory T cells by anti-CTLA-4 antibody is promoted by TLR1/2 agonist and hence its efficacy in combination treatment of melanoma

#### Naveen Sharma, James Allison

##### University of Texas MD Anderson Cancer Center, Houston, TX, USA

###### **Correspondence:** Naveen Sharma (nsharma1@mdanderson.org)

**Background**

Immune checkpoint blockade therapies have been successfully employed clinically to treat melanoma. Ipilimumab, which blocks inhibitory receptor CTLA-4 was one of the first checkpoint blockade therapies to get FDA approval for treating melanoma patients. Despite the effectiveness of these drugs, a significant number of cancer patients do not respond, and durable responses are only observed in a fraction of patients across tumor types. Therefore, combination therapies including checkpoint blockade antibodies are being studied to improve the outcome from immunotherapy treatment. Pattern recognition receptors like TLRs have been shown to have anti-tumor effects in various tumor models, through their effect on innate immunity.

**Methods**

In this study, we set out to combine the innate immune arm like TLR ligand with the adaptive immune arm for treatment of melanoma in a mouse model. We identified TLR1/2 ligand Pam3CSK4 as innate immune system modulator to combine with anti-CTLA-4 antibody in this combination therapy. Mice were injected intradermal (i.d.) on the right flank with 3 × 10^5^ B16/F10 and considered day 0. Initial B16/F10 challenge was doubled to 6 × 10^5^ in experiments where mice would be sacrificed on day 14. Mice were then treated with intraperitoneal (i.p) injection of 100 μg anti-CTLA-4 antibody and intratumoral injection with TLR1/2 ligand on every third after initial tumor challenge till day 12. The dose of anti-CTLA-4 antibody was doubled on day 3. In experiments where mice would be sacrificed on day 14, only two doses of anti-CTLA-4 antibody and TLR ligands were given on Day 9 and Day 12 after injection. These mice were either sacrificed on day 14 for obtaining lymphoid organs and tumors for phenotypic and functional analysis or tumor growth was analyzed.

**Results**

Our studies show that combining TLR1/2 ligand Pam3CSK4 with anti-CTLA-4 antibody decreases tumor burden and increases survival significantly, compared to anti-CTLA-4 antibody treatment alone. In our studies we found both CD4+ and CD8+ T cells to be important for this combination treatment efficacy. Most interestingly, we found that the mechanism of efficacy of combination treatment is due to an increased depletion of regulatory T cells modulated by enhanced FcγRIV expression on macrophages in combination therapy.

**Conclusions**

Our findings suggest that combining TLR1/2 ligand with anti-CTLA-4 antibody will be an interesting prospect for treatment of cancer and it also suggest that TLR1/2 ligand modulate FcγRIV expression, which can be used to modulate the efficacy of other antibody-based immunomodulatory therapies.

### P240 Immunostimulatory and oncolytic properties of rotavirus can overcome resistance to immune checkpoint blockade therapy

#### Tala Shekarian^1^, Sandrine Valsesia-Wittmann^2^, Christophe Caux^1^, Aurelien Marabelle^3^

##### ^1^Université Claude Bernard Lyon 1, Centre Leon Berard, Lyon, Rhone-Alpes, France; ^2^Centre Leon Berard, Innovations in Immunotherapy Platform, Lyon, Rhone-Alpes, France; ^3^Institue Goustave Roussy, Université Claude Bernard Lyon 1,Centre Leon Berard, Villejuif, Ile-de-France, France

###### **Correspondence:** Tala Shekarian (talashekarian@yahoo.com)

**Background**

Immune checkpoint targeted therapies against PD-1, PD-L1, and CTLA-4 are currently revolutionizing cancer care. However, a minority of patients generate objective tumor responses with these treatments. Therefore, new therapeutic interventions are needed to increase the immunogenicity of tumors in order to overcome the resistance to immune checkpoint blockade therapy. Pattern recognition receptors (PRR) such as toll-like receptor agonists have been shown to overcome resistance to immune checkpoint targeted therapy in pre-clinical models. Besides their intrinsic ability to stimulate PRR, the oncolytic properties of common viruses can be exploited also for the priming of anti-tumor immune responses. Hypothesis: Can anti-infectious vaccines be used as a source or PRR agonists and/or oncolytic viruses?

**Methods**

We tested a TLR-Luc transgenic cell line for screening of the TLR agonist activity of anti-infectious vaccines. Cytotoxic activity induced by the vaccines was determined by SRB test and IncuCyte imaging. Flow cytometry was performed to identify the type of cell death and to characterize different population of infiltrated immunity cells in the tumors. *In vivo* effect of immune checkpoints blockade were determined in monotherapy and in combination with the vaccines on tumor growth.

**Results**

We confirmed that commercially available anti-infectious vaccines do have PRR agonist properties. Interestingly, we discovered that rotavirus vaccines also have oncolytic properties. These attenuated viruses can directly kill cancer cells with features of immunogenic cell death such as upregulation of calreticulin on dying cancer cells. Moreover, they have pro-inflammatory properties and can activate the NF-Kb pathway in a TLR and IRF3 independent manner. These *in vitro* biological properties translate into *in vivo* anti-tumor activity. Intra-tumoral rotavirus therapy has anti-tumor effects which are partly immune mediated as demonstrated by their activity in NSG xenograft models of human tumors. Interestingly, in immunocompetent syngeneic murine tumor models of neuroblastoma and lymphoma, intra-tumoral rotavirus therapy can overcome resistance and synergize with immune checkpoint targeted therapy. This therapeutic effect relied on specific modifications of tumor immune infiltrates and immune activation pathways. Intratumoral rotavirus vaccines was associated to an increase of leukocytes in the tumor microenvironment and upregulation of activation markers such as OX40/CD137 and CD86 on T cells and APC, respectively.

**Conclusions**

Rotavirus vaccines are clinical grade products. Therefore, *in situ* immunization strategies with intra-tumoral attenuated rotavirus can be implemented quickly in the clinic. Intra-tumoral priming of the anti-tumor immunity with oncolytic and immunostimulatory rotavirus vaccines could be a feasible strategy to overcome resistance to anti-PD-1/anti-CTLA-4 therapy in patients with cancer.

### P241 A phase I/II study of durvalumab alone or in combination with AXAL in recurrent/persistent or metastatic cervical or human papillomavirus (HPV) + squamous cell cancer of the head and neck (SCCHN): preliminary phase I results

#### Brian M Slomovitz^1^, Kathleen M Moore^2^, Hagop Youssoufian^3^, Marshall Posner^4^

##### ^1^Sylvester Comprehensive Cancer Center/University of Miami, Miami, FL, USA; ^2^Stephenson Oklahoma Cancer Center, Oklahoma City, OK, USA; ^3^Advaxis Immunotherapies, Princeton, NJ, USA; ^4^Icahn School of Medicine at Mount Sinai, Mount Sinai Medical Center, New York, NY, USA

###### **Correspondence:** Brian M Slomovitz (bslomovitz@med.miami.edu)

**Background**

The success of immunotherapy for cervical cancer and HPV+ head and neck cancer may be enhanced by a combination of immune checkpoint blockade and tumor-selective vaccination. Axalimogene filolisbac (AXAL or ADXS11-001) is an irreversibly attenuated *Listeria monocytogenes-*listeriolysin O (*Lm*-LLO) immunotherapy bioengineered to secrete an HPV E7 tLLO fusion protein that induces HPV-specific cytotoxic T cells and reduces tumor-associated immune tolerance. Durvalumab is a selective, high-affinity human IgG1 mAb that blocks PD-L1 binding to PD-1 (IC_50_ 0.1 nM) and CD80 (B7.1; IC_50_ 0.04 nM). The PD-1/PD-L1 pathway is an important checkpoint used by tumor cells to inhibit antitumor responses. Preclinical mouse models demonstrate combination AXAL/anti–PD-1 treatment significantly reduces tumor growth and prolongs survival.

**Methods**

This is a phase I/II study (NCT02291055) of AXAL + durvalumab in patients (≥18 years) with either recurrent/metastatic cervical cancer or metastatic HPV+ SCCHN, who progressed on ≥1 platinum-based therapy. The primary objectives of the phase I, Part A dose escalation are to determine the safety/tolerability and establish the combination recommended phase II dose (RP2D) of AXAL (1 × 10^9^ colony-forming units q4wk) and durvalumab (3 mg/kg or 10 mg/kg q2wk) following a 3 + 3 design. Part A includes a SCCHN expansion cohort (N = 20) at the RP2D to evaluate efficacy. Part B will evaluate tumor response (RECIST and immune-related RECIST) of durvalumab monotherapy and AXAL + durvalumab combination therapy at the RP2D in recurrent/metastatic cervical cancer. Preliminary results of phase I dose escalation are reported.

**Results**

To date, 11 patients are enrolled in phase I (AXAL + durvalumab 3 mg/kg: N = 5; AXAL + durvalumab 10 mg/kg: N = 6); 91 % had ECOG performance status 0, 73 % had cervical cancer, of which 75 % received prior bevacizumab. No dose-limiting toxicities have been observed. The following adverse events (AEs) were reported (3 vs. 10 mg/kg): 100 % vs. 83 % of patients experienced AEs; 20 % vs. 50 % experienced SAEs; 60 % vs. 50 % experienced Grade 1 and Grade 2 treatment related AEs (TRAEs); Grade 3 TRAEs occurred in n = 1 (rigors) and n = 2 (rigors and neutropenia, respectively) patients. In the AXAL + durvalumab 3 mg/kg cohort, 2 patients with cervical cancer obtained an objective response; 1 CR that is ongoing (9 months follow-up) and 1 PR with subsequent disease progression. Tumor assessments from the AXAL + durvalumab 10 mg/kg cohort are not yet available. The RP2D was declared at durvalumab 10 mg/kg q2wk + AXAL 1 × 10^9^ colony-forming units q4wk.

**Conclusions**

The combination of AXAL/durvalumab appears safe and tolerable. Preliminary data indicate encouraging antitumor activity of the combination immunotherapy regimen.

**Trial Registration**

ClinicalTrials.gov identifier NCT02291055.

### P242 A specific 17-beta-hydroxywithanolide (LG-02) sensitizes cancer cells to apoptosis in response to TRAIL and toll-like receptor (TLR) ligands

#### Poonam Tewary^1^, Alan D Brooks^2^, Ya-Ming Xu^3^, Kithsiri Wijeratne^3^, Leslie AA Gunatilaka^4^, Thomas J Sayers^1^

##### ^1^CIP, Center for Cancer Research, BSP, Leidos Biomed Research Inc, National Cancer Institute-Frederick, Frederick, MD, USA; ^2^CIP, Center for Cancer Research, Leidos Biomed Research Inc, National Cancer Institute-Frederick, Frederick, MD, USA; ^3^University of Arizona, Southwest Center for Natural Products Research and Commercialization, Tucson, AZ, USA; ^4^University of Arizona, Tucson, AZ, USA

###### **Correspondence:** Poonam Tewary (tewaryp@mail.nih.gov)

**Background**

Despite many therapeutic successes, cancer is the second-most frequent cause of mortality in the United States. Strategies for cancer therapy aim to overcome excessive proliferation and avoidance of apoptosis. Therefore, methods of inducing apoptosis have become an important approach in the design of effective cancer therapies. Among these tumor necrosis factor-related apoptosis inducing ligand (TRAIL) has shown considerable promise as a nontoxic apoptotic inducer in cancer immunotherapy. However, many primary tumors are inherently resistant to TRAIL-mediated apoptosis and require additional sensitization. Therefore, there is an underlying interest in identifying agents that can be combined with TRAIL to improve its efficacy. Recent studies have also described a role of TLR3 signaling for initiating apoptosis in malignant cells and thus promote anticancer immune responses. We have previously shown that, withanolide E (WE), a 17β-hydroxywithanolide (17-BHW) and a natural product derived from the medicinal plant *Physalis peruviana* was capable of sensitizing tumor cells to TRAIL-mediated apoptosis by reducing cellular levels of the anti-apoptotic protein cFLIP.

**Methods**

Encouraged by this, we screened a small library of 17-BHWs and have identified several that are more potent than WE for their ability to promote death ligand-mediated cancer cell death.

**Results**

Among the 30 compounds tested, LG-02 was found to be 4–5 fold more potent than WE in sensitizing tumor cells to apoptotic signaling in response TRAIL as well as to the synthetic polynucleotide, poly(I:C), which is known to mimic anti-viral responses by activating TLR (toll-like receptor) signaling. Intra-tumor administration of LG-02 and poly(I:C) in a xenograft M14 melanoma model provided therapeutic benefit leading to complete tumor regression in 90 % of the mice as compared to mice treated with vehicle or compounds alone. Molecular studies in melanoma cells demonstrated decreases not only in the anti-apoptotic cFLIP proteins but also in a number of IAPs including livin following LG-02 treatment. To date there are no withanolides reported to have this dual activity on reducing levels of different anti-apoptotic proteins.

**Conclusions**

Thus, we hypothesized that 17-BHWs represent a unique NP scaffold, structural modification of which would lead to potent non-toxic sensitizers of apoptosis by TLR signaling that utilizes a downstream pathway similar to that of TNF death receptor signaling. Further studies with 17-BHWs could lead to the identification of novel and common therapeutic targets involved in apoptosis signaling in response to both TNF death receptor family members as well as TLR ligands.

**Acknowledgements**

Funded by NCI Contract HHSN261200800001E.

### P243 Intratumoral administration of the TLR7/8 agonist MEDI9197 inhibits tumor growth and modulates the tumor microenvironment

#### John P Vasilakos^1^, Tesha Alston^1^, Simon Dovedi^2^, James Elvecrog^1^, Iwen Grigsby^1^, Ronald Herbst^3^, Karen Johnson^1^, Craig Moeckly^1^, Stefanie Mullins^2^, Kristen Siebenaler^1^, Julius SternJohn^1^, Ashenafi Tilahun^1^, Mark A Tomai^1^, Katharina Vogel^2^, Robert W Wilkinson^2^

##### ^1^3M Company, St. Paul, MN, USA; ^2^Medimmune, Cambridge, England, UK; ^3^Medimmune, Gaithersburg, MD, USA

###### **Correspondence:** John P Vasilakos (jpvasilakos@mmm.com)

**Background**

Toll-like receptor (TLR) agonists, such as the TLR7 agonist imiquimod, have been evaluated topically and systemically for cancer. Topical administration has shown antitumor activity against various cancers, such as melanoma, squamous cell carcinoma, and cutaneous breast cancer. However, systemic administration of TLR agonists in cancer patients has resulted in limited efficacy, in part due to cytokine-induced systemic adverse effects, which limits the therapeutic window. Therefore, a lipophilic imidazoquinoline, MEDI9197, was designed to be retained within the tumor following injection with the primary objective of directing immune activation to the tumor.

**Methods**

The antitumor effects of intratumoral (IT) administered MEDI9197 were evaluated in 4 different mouse syngeneic subcutaneous implantation tumor models. Tumor and serum drug levels were quantified following IT administration. In addition, the tumor immune profile was assessed by qPCR, histology, and flow cytometry. Lastly, the antitumor effects of combination therapy using IT injected MEDI9197 in conjunction with CTLA-4 or PD-L1 antibodies were evaluated.

**Results**

MEDI9197 is a human TLR7/8 agonist. Following IT administration, pharmacokinetic analysis shows that the drug is retained in the tumor, and very low levels of the drug are detected in the serum. MEDI9197 mediates antitumor activity (tumor growth inhibition and enhanced survival) in B16F10 luc, B16-OVA, 4 T1, and CT-26 mouse tumor models. Administration of MEDI9197 by the IT route and by the SC route away from the tumor demonstrates that the antitumor effects of MEDI9197 require IT administration. IT dosed MEDI9197 modulates the local immune response characterized by an upregulation of genes involved in innate and adaptive immunity. IT dosed MEDI9197 induces tumor necrosis, leukocyte activation, and the formation of lymphoid aggregates evident by 7 days postdose. IT injected MEDI9197 increases the number of tumor infiltrating CD8+ T cells, while concomitantly decreasing the number of tumor infiltrating CD4+ T cells. Moreover, MEDI9197 induces prolonged activation of tumor T cells and NK cells. Additionally, combination of MEDI9197 with CTLA-4 and PD-L1 antibodies enhances the efficacy observed in syngeneic mouse tumor models.

**Conclusions**

The data presented shows IT administration of the TLR7/8 agonist MEDI9197 is retained in the tumor, modulates the tumor microenvironment in a manner consistent with an antitumor signature, and inhibits tumor growth in multiple mouse cancer models. Finally, the antitumor effects of MEDI9197 are further enhanced by combination therapy with checkpoint blockade therapies. MEDI9197 is currently being evaluated for safety and efficacy in human clinical trials (ClinicalTrials.gov Identifier: NCT02556463).

### P244 Impact of intratumoral clonal heterogeneity on checkpoint inhibitor response

#### Eveline E Vietsch, Anton Wellstein

##### Georgetown University/Medical School/Lombardi Cancer Center, Washington, DC, USA

###### **Correspondence:** Anton Wellstein (wellstea@georgetown.edu)

**Background**

Cancer cells are subjected to evolutionary selection of clonal populations by changes in the microenvironment as well as their response to drug treatment. We wished to evaluate how this heterogeneity impacts efficacy of checkpoint inhibition.

**Methods**

To understand the contribution of clonal subpopulations to the malignant progression and to the response to drugs, we established a model of tumor heterogeneity from six syngeneic, clonal primary cancer cells isolated from a mutant Kras/P53 mouse pancreatic cancer (KPC). The clones were characterized molecularly and tumors reconstituted from mixes of the clonal cell lines.

**Results**

These clonal cells formed invasive and metastatic lesions when grafted into hosts. The original tumor and clonal cell lines harbored common mutations in 99 genes suggesting their common ancestry. Additional unique mutations in the clonal lines were used to identify and quantitate clones in heterogeneous cell pools. The clones showed different levels of MAP kinase signaling, unique morphologies, different growth rates *in vitro* and tumor growth rates in immune competent mice. Moreover, the sensitivity to ~200 anticancer drugs revealed an up to 25-fold varying *in vitro* sensitivity of the clones to signal transduction inhibitors and cytotoxic drugs. To our surprise, drug sensitivity of individual clones when included in a heterogeneous cell population was strikingly different from their drug sensitivity when growing on their own. In particular, the sensitivity of clones to MEK or PI3K inhibition was not predictive of their sensitivity when grown in a pool with the other clones. Furthermore, the sensitivity of clones to an anti-PD-1 checkpoint inhibitor was distinct across the clonal cells growing in the heterogeneous mixture. Some clones were resistant and others highly sensitive to the checkpoint inhibition. We will discuss pathways and drivers of resistance in the different subpopulations.

**Conclusions**

We conclude that malignant progression and selection of checkpoint inhibitor sensitive cancer cell subpopulations is impacted by the crosstalk between clonal cell populations present in heterogeneous tumors and the host environment.

### P245 Discovery and characterization of PF-06840003, a novel brain penetrant IDO1 inhibitor

#### Martin Wythes^1^, Stefano Crosignani^2^, Joseph Tumang^1^, Shilpa Alekar^1^, Patrick Bingham^1^, Sandra Cauwenberghs^2^, Jenny Chaplin^1^, Deepak Dalvie^1^, Sofie Denies^2^, Coraline De Maeseneire^2^, JunLi Feng^1^, Kim Frederix^2^, Samantha Greasley^1^, Jie Guo^1^, James Hardwick^1^, Stephen Kaiser^1^, Katti Jessen^1^, Erick Kindt^1^, Marie-Claire Letellier^2^, Wenlin Li^1^, Karen Maegley^1^, Reece Marillier^2^, Nichol Miller^1^, Brion Murray^1^, Romain Pirson^2^, Julie Preillon^3^, Virginie Rabolli^2^, Chad Ray^1^, Kevin Ryan^1^, Stephanie Scales^1^, Jay Srirangam^1^, Jim Solowiej^1^, Al Stewart^1^, Nicole Streiner^1^, Vince Torti^1^, Konstantinos Tsaparikos^1^, Xianxian Zheng^1^, Gregory Driessens^2^, Bruno Gomes^2^, Manfred Kraus^1^

##### ^1^Pfizer, San Diego, CA, USA; ^2^iTeos, 6041 Gosselies, Brussels Hoofdstedelijk Gewest, Belgium; ^3^iTeos, Rue Auguste Piccard 48, Brussels Hoofdstedelijk Gewest, Belgium

###### B: Martin Wythes (martin.wythes@pfizer.com)

**Background**

Tumors use tryptophan-catabolizing enzymes such as indoleamine 2–3 dioxygenase (IDO1) to induce an immunosuppressive environment. IDO1 is induced in response to inflammatory stimuli and promotes immune tolerance, effector T cell anergy and enhanced Treg function. As such, IDO1 is a nexus for the induction of key immunosuppressive mechanisms and represents an important immunotherapeutic target in oncology.

**Methods**

We have identified and characterized a new, selective, orally bioavailable IDO1 inhibitor, PF-06840003.

**Results**

Key interactions of PF-06840003 with IDO1 will be presented, and rationalized using a novel X-ray crystal structure of PF-06840003 bound to human IDO1. In addition, binding studies with ferrous and ferric forms of human IDO1 have been performed. The results suggest that PF-06840003 is a tryptophan non-competitive, non-heme binding IDO1 inhibitor. Key *in vitro* and *in vivo* pharmacology data, including combination studies with checkpoint inhibitors, and ADME data of PF-06840003 will be discussed. PF-06840003 shows a very favorable ADME profile (solubility, human hepatocyte stability, low *in vivo* clearance in preclinical species, high permeability, and high fraction absorbed in preclinical species) leading to favorable predicted human pharmacokinetic properties, including a predicted t_1/2_ of 19 hours.

**Conclusions**

PF-06840003 is a selective IDO1 inhibitor with very favorable predicted human PK characteristics. Its prolonged projected human half-life should allow QD administration. CNS penetration suggests potential impact on brain metastases. Checkpoint antagonists against PD-L1 cause enhanced IDO1 expression and enhanced *in vivo* anti-tumor efficacy in combination with PF-06840003. These studies highlight the potential of PF-06840003 as a clinical candidate in Immuno-Oncology. A first in patient study for PF-06840003 in malignant gliomas is described at ClinicalTrials.gov (NCT02764151).

### P246 Enhanced anti-tumor effect of combination therapy with NHS-muIL-12 and anti-PD-L1 antibody (avelumab) in a preclinical cancer model

#### Chunxiao Xu^1^, Yanping Zhang^2^, Giorgio Kradjian^2^, Guozhong Qin^2^, Jin Qi^2^, Xiaomei Xu^2^, Bo Marelli^2^, Huakui Yu^2^, Wilson Guzman^2^, Rober Tighe^2^, Rachel Salazar^2^, Kin-Ming Lo^2^, Jessie English^2^, Laszlo Radvanyi^2^, Yan Lan^2^

##### ^1^EMD Serono, Belmont, MA, USA; ^2^EMD Serono, Billerica, MA, USA

###### **Correspondence:** Chunxiao Xu (chunxiao.xu@emdserono.com)

**Background**

Recent clinical studies have found that treatment with the immune checkpoint inhibitors anti-PD-1 or PD-L1 induce durable anti-tumor responses in some patients with advanced-stage cancers. However, many patients do not benefit from treatment because the induction, potency, and persistence of immune responses depend on a complex interplay between different immune cell populations. Thus, treatment with a combination of therapies that target distinct immune pathways may be a promising strategy to improve anti-tumor efficacy. NHS-IL-12 (MSB0010360N; M9241) is an investigational immunocytokine designed to target tumor necrotic regions as a method to deliver IL-12 into the tumor microenvironment. Avelumab* (MSB0010718C) is a fully human anti-PD-L1 IgG1 monoclonal antibody designed to selectively bind to PD-L1 and competitively inhibit it from binding to PD-1, which has shown antitumor activity in various malignancies in clinical trials.

**Methods**

In the pre-clinical studies described here, the anti-tumor efficacy of combination treatment with avelumab and the surrogate NHS-muIL-12 was investigated in an orthotopic EMT-6 breast cancer model’

**Results**

Treatment with NHS-muIL-12 and avelumab generated an enhanced anti-tumor effect relative to either monotherapy. Most mice treated with the combination therapy had complete tumor regression and generated tumor-specific immune memory, as demonstrated by their protection against rechallenge with EMT-6 tumor cells and the significant induction of effector and memory T cells. The combination treatment dose-dependently stimulated cytotoxic NK and CD8+ T cell proliferation. NHS-muIL-12 treatment induced CD8+ T cell infiltration into the tumor microenvironment consistent with the induction of chemoattractants. Also, avelumab monotherapy reversed T cell immunosuppression and restored the function of exhausted CD8+ T cells in the tumor microenvironment.

**Conclusions**

These preclinical findings indicate that the combination therapy with NHS-IL-12 and avelumab may provide a promising approach to treat patients with solid tumors. Asterisk (*) indicates a proposed nonproprietary name.

### P247 Opposing effects of CTLA-4 and PD-1 blockade on follicular helper-like T cells with immunosuppressive functions

#### Roberta Zappasodi^1^, Sadna Budhu^1^, Matthew D Hellmann^2^, Michael Postow^2^, Yasin Senbabaoglu^1^, Billel Gasmi^1^, Hong Zhong^1^, Yanyun Li^1^, Cailian Liu^1^, Daniel Hirschhorhn-Cymerman^1^, Jedd D Wolchok^2^, Taha Merghoub^1^

##### ^1^Ludwig Collaborative Laboratory, Memorial Sloan Kettering Cancer Center, New York, NY, USA; ^2^Department of Medicine, Memorial Sloan Kettering Cancer Center, New York, NY, USA

###### **Correspondence:** Roberta Zappasodi (zappasor@mskcc.org)

**Background**

The mechanism underlying the improved anti-tumor activity of combined CTLA-4 and PD-1 blockade is not yet well understood. We reported that expansion of CD4^+^Foxp3^−^ T cells expressing PD-1 (4PD-1^hi^) was associated with limited therapeutic improvement when a CTLA-4-blocking antibody was added to an anti-melanoma vaccine in B16-melanoma bearing mice. We went on to define functions and origin of 4PD-1^hi^ to clarify the significance of their modulation by checkpoint blockade in mouse tumor models and cancer patients.

**Methods**

4PD-1^hi^ frequency was monitored by flow cytometry. Their function was tested in standard *in vitro* suppression assays and 3D collagen-fibrin gel killing assays. RNAseq gene expression analyses were performed on a Proton sequencing system at the MSK Genomics Core Facility.

**Results**

Circulating and intra-tumor 4PD-1^hi^ frequencies positively correlated and both increased as a function of tumor burden in anti-CTLA-4-treated and naïve B16-bearing mice, suggesting a pro-tumor role of 4PD-1^hi^. Accordingly, the ratio between effector T cell (Teff) and 4PD-1^hi^ inversely correlated with tumor size. 4PD-1^hi^ from spleens and tumors of naïve and B16-bearing Foxp3-GFP-transgenic mice treated or not with CTLA-4 blockade suppressed Teff functions. RNAseq gene expression analysis revealed an enrichment of follicular helper T cell-(Tfh)-associated genes in 4PD-1^hi^ in comparison with regulatory T cells (Tregs) and CD4^+^Foxp3^−^PD-1^−^ T cells. We therefore immunized Foxp3-GFP transgenic mice with sheep red blood cells (sRBC) to boost development of Tfh and test their function *in vitro*. 4PD-1^hi^ from sRBC-treated animals inhibited Teff more efficiently than those isolated from untreated mice. However, in contrast to Tregs and according to their Tfh-like phenotype, 4PD-1^hi^ promoted activation and maturation of B cells *in vitro*. Moreover, concurrent PD-1 and CTLA-4 blockade, either alone or in combination with an anti-melanoma vaccine, prevented 4PD-1^hi^ expansion and significantly improved anti-tumor responses in mice. In cancer patients, ipilimumab increased, whereas PD-1 blockade reduced, circulating 4PD-1^hi^. We took advantage of differential CD25 expression in 4PD-1^hi^ and Tregs to isolate and compare these two cell subsets from healthy donors’ peripheral blood as well as patients’ tumors. Human 4PD-1^hi^ inhibited Teff functions *in vitro* and expressed Tfh-associated markers, thus confirming our observations in mice.

**Conclusions**

Our study describes T cell suppression functions of Tfh-like cells expanded by CTLA-4 blockade. Importantly, we show that these cells exist in healthy individuals and expand in the presence of tumor. We provide evidence that PD-1 blockade counteracts anti-CTLA-4-mediated 4PD-1^hi^ induction, thus underscoring one of the mechanisms potentially responsible for the improved therapeutic activity of combination checkpoint blockade.

**Consent**

Written informed consent was obtained from the patient for publication of this abstract and any accompanying images. A copy of the written consent is available for review by the Editor of this journal.

### P248 WT1 peptide vaccine in Montanide or poly-ICLC triggers different immune responses in patients with myeloid leukemia

#### Yuanyuan Zha^1^, Gregory Malnassy^2^, Noreen Fulton^2^, Jae-Hyun Park^2^, Wendy Stock^3^, Yusuke Nakamura^2^, Thomas F Gajewski^4^, Hongtao Liu^4^

##### ^1^University of Chicago, OSRF-HIM, Chicago, IL, USA; ^2^University of Chicago, Chicago, IL, USA; ^3^University of Chicago, Section of Hematology/Oncology, Chicago, IL, USA; ^4^University of Chicago Medical Center, Chicago, IL, USA

###### **Correspondence:** Yuanyuan Zha (yzha1@bsd.uchicago.edu)

**Background**

It has been well established that human T cells can recognize and destroy tumor cells. In solid tumors, it has been shown that peptide vaccine against tumor antigens can augment host anti-tumor immune response and achieve tumor control in some patients. WT1 is a defined leukemia-associated antigen, a transcription factor that over-expressed in AML, CML, ALL, and other tumors. WT1 is highly antigenic and is an attractive target for immunotherapy. However, the optimal strategy for vaccination to induce WT1-specific immune responses is not known.

**Methods**

In this pilot study, we randomized seven (4 males, 3 female ages 39 to 73) HLA-A02+ patients with myeloid leukemia in the minimal residual disease state to receive vaccination with WT1 126–134 peptide (RMFPNAPYL) in either Montanide or poly-ICLC (TLR3 agonist). Four patients were randomized to receive WT1 in Montanide and three were randomized to receive WT1 in poly-ICLC. The vaccine was administered every other week X 6 during the induction phase followed by monthly booster vaccinations X 6 months. Patients were monitored for disease and toxicity. Blood was collected to monitor WT1 transcript levels, antigen-specific CD8+ T cell responses, and TCR sequencing.

**Results**

After WT1 vaccination, three of four patients in the Montanide arm had deceased WT1 levels in circulation detected by qRT-PCR, and two of these demonstrated augmented WT1-specific CD8+ T cell responses detected by IFN-γ ELISPOT assay. All three patients had TCR clonal enrichment after WT1 vaccination suggested by TCR alpha and beta CDR3 sequencing. In contrast, in the two patients on the poly-ICLC arm, no increase in WT1-specific CD8+ T cell responses was detected by IFN-γ ELISPOT assay, and no clonal enrichment was detected by TCR alpha/beta sequencing. Interestingly, these two patients nonetheless demonstrated decreased WT1 transcript levels in circulation detected by qRT-PCR and remained in remission 3 years after the initiation of WT1 vaccination.

**Conclusions**

Our results show that vaccination with WT1 peptide emulsified in Montanide is a superior vaccine strategy based on increased WT1-specific CD8+ T cell responses with TCR clonal and specific TCR beta CDR3 enrichment and decreased WT1 transcripts as a measure of minimal residual disease. The fact that vaccination with WT1 peptide in poly-ICLC nonetheless was associated with decreased WT1 transcripts suggests that a distinct immune activation mechanism might be occurring, for example an effect on dendritic cells of poly-ICLC alone.

### P249 Combination of Listeria-based human papillomavirus (HPV) E7 cancer vaccine (AXAL) with CD137 agonist antibody provides an effective immunotherapy for HPV-positive tumors in a mouse model

#### Xiaoming Ju, Rachelle Kosoff, Kimberly Ramos, Brandon Coder, Robert Petit, Michael Princiotta, Kyle Perry, Jun Zou

##### Advaxis Immunotherapies, Princeton, NJ, USA

###### **Correspondence:** Jun Zou (zou@advaxis.com)

**Background**

HPV can cause cervical, anal, vulvar, vaginal, penile, and oropharyngeal cancers. AXAL is a genetically engineered *Listeria monocytogenes*-based therapeutic cancer vaccine currently in clinical trials for cervical (phase III), anal (phase II), and head and neck (phase I/II) cancers, either as monotherapy or in combination with checkpoint inhibitor (PD-1 or PD-L1) antibodies. To identify potentially synergistic immunotherapies, we evaluated AXAL ± antibodies for T cell co-inhibitory or co-stimulatory receptors (checkpoint inhibitors: CTLA-4, PD-1, TIM-3, LAG-3; co-stimulators: CD137, OX40, GITR, and CD40) in a mouse HPV-positive tumor model.

**Methods**

C57BL/6 female mice and TC1 cells (C57BL/6 mouse lung epithelial cells co-transfected with HPV16 E6 and E7 and activated *Ras*) were obtained from Jackson Laboratories and ATCC, respectively. All antibodies were obtained from Bio X Cell. Mice were subcutaneously injected on the hind-leg flank with TC1 cells. AXAL ± the respective antibodies was injected intraperitoneally at 5 × 10^7^ colony-forming units/mouse weekly for 3 total doses. For combinations with superior performance, the tumor microenvironment (TME) was further evaluated using flow cytometry to immunophenotype the tumor-infiltrating lymphocytes (TILs), spleen, and tumor-draining lymph node.

**Results**

Among 8 antibodies tested in combination with AXAL, CD137 and CTLA-4 antibodies were the most effective for tumor growth inhibition, tumor regression, and survival. Consistent with prior reports that CD137 is expressed on natural killer, dendritic, and T cells, and can potentiate antitumor responses by altering the cellular makeup of the TME [1], immunophenotyping revealed increased TILs and CD8/Treg ratio, and decreased levels of highly immunosuppressive CD103-positive Tregs after CD137 + AXAL treatment versus treatment with either agent alone. Additionally, increases were observed in PD-L1 expression on tumor cells and PD-1 expression on CD8-positive T cells. Mice with complete tumor regression after CD137 + AXAL treatment (n = 5) were subsequently rechallenged with TC1 cells. Two mice remained tumor free until study termination (an additional 6–7 weeks); the other 3 had delayed or slower tumor growth versus controls. CTLA-4 + AXAL treatment resulted in complete tumor regression in 3 mice evaluated. These mice remained tumor free even after rechallenge.

**Conclusions**

AXAL demonstrated strong anticancer activity in this preclinical model of HPV-positive cancer, especially in combination with CD137 and CTLA-4 antibodies. Moreover, these data suggest that addition of anti-PD-1 to anti-CD137 + AXAL could be a potent triple combination therapy.

**References**

1. Makkouk A, Chester C, Kohrt HE: **Rationale for anti-CD137 cancer immunotherapy.** *Eur J Cancer* 2016, **54**:112–119.

## Combinations: Immunotherapy/Standard of Care

### P250 Tumor-resident T cells survive and mediate the antitumoral effects in a murine model of cancer therapy with localized ionizing radiation

#### Ainhoa Arina^1^, Christian Fernandez^1^, Wenxin Zheng^1^, Michael A Beckett^1^, Helena J Mauceri^1^, Yang-Xin Fu^2^, Ralph R Weichselbaum^1^

##### ^1^The University of Chicago, Chicago, IL, USA; ^2^UT Southwestern, Dallas, TX, USA

###### Corespondence: aarina@bsd.uchicago.edu

**Background**

A role for T cells in the antitumor effects of radiation therapy is becoming increasingly clear [1]. Since lymphocytes are considered to be very sensitive to ionizing radiation (IR), and IR increases T cell infiltration of tumors, it is usually assumed that the newly infiltrating T cells mediate the therapeutic effects of IR. However, there is no clear data showing the contribution of tumor-resident vs. newly infiltrating T cells to the therapeutic effects of IR.

**Methods**

Longitudinal *in vivo* imaging of tumors using window chambers was performed as described [2]. “T cell reporter” mice were obtained by crossing CD4-Cre or Lck-cre mice with R26-stop-EYFP mice (Jackson).

**Results**

Panc02SIYCerulean cancer cells were injected s.c. into EYFP^+^ T cell reporter mice bearing dorsal window chambers. When tumors were established (around day 21), mice received 800 cGy of whole-body irradiation (WBI) while their tumor was shielded. This procedure depleted most peripheral T cells while preserving tumor-resident EYFP^+^ T cells. Following bone-marrow reconstitution with DsRed^+^Rag^−/−^ cells, EGFP^+^ 2C CD8^+^ T cells specific for the SIY antigen were adoptively transferred, to distinguish newly infiltrating T cells. 3–4 days after 2C transfer, some mice received local IR as follows. 2 experiments using (i) 5 doses of 1.8 Gy each, 24 hours apart (fractionated IR model), and (ii) a single dose of 20 Gy (SBRT model) showed that a significant proportion of tumor-resident EYFP^+^ T cells were still detected in the tumor and kept their motility even after 20 Gy of IR, for up to 2 weeks. 2C-EGFP^+^ T cells infiltrated IR-treated tumors with some delay, but eventually reached high numbers in IR-treated and untreated tumors. Treating MC38 tumor-bearing mice with increasing doses of WBI (1–10 Gy) showed that tumor-resident T cells were more resistant to IR than circulating T cells. Experiments treating MC38-bearing animals with 20 Gy local IR and systemic sphingosine 1-phosphate receptor agonist FTY720, suggest that tumor-resident T cells might suffice for the antitumoral effects of single high-dose IR.

**Conclusions**

Tumor-resident T cells show preferential survival to IR compared to circulating T cells and can contribute to the therapeutic effects of radiotherapy.

**References**

1. Burnette B, Fu YX, Weichselbaum RR: **The confluence of radiotherapy and immunotherapy**. *Front Oncol* 2012, **2**:143.

2. Schietinger A, Arina A, *et al.*: **Longitudinal confocal microscopy imaging of solid tumor destruction following adoptive T cell transfer**. *Oncoimmunology* 2013, **2**:e26677.

### P251 Multi-kinase inhibitors for the treatment of mRCC: implications for combined therapy with AGS-003, an autologous dendritic cell immunotherapy

#### Mark DeBenedette, Whitney Lewis, Alicia Gamble, Charles Nicolette

##### Argos Therapeutics, Durham, NC, USA

###### **Correspondence:** Mark DeBenedette (mdebenedette@argostherapeutics.com)

**Background**

AGS-003, is an autologous dendritic cell (DC) immunotherapy consisting of matured DCs co-electroporated with amplified autologous tumor RNA and CD40L RNA. AGS-003, is being evaluated in the pivotal ADAPT phase III clinical trial for the treatment of metastatic renal cell carcinoma (mRCC) in combination with standard-of-care, based on a phase II clinical trial suggesting that response combination of sunitinib + AGS-003 was greater than sunitinib alone. The standard-of-care tyrosine kinase inhibitors including sunitinib, sorafinib, axitinib, pazopanib, cabozantinib and tivozanib, and the mTOR inhibitors, everolimus and temsirolimus, are anti-angiogenic therapeutics targeting signaling pathways implicated in the progression of RCC. However, these same signaling pathways are essential for the activation of antigen-specific T cell responses. Combining kinase inhibitor therapy with an active immunotherapeutic, such as AGS-003, may be ineffective, if kinase inhibitor therapy impedes the induction of CTL responses *in vivo*, which is the proposed mechanism of action (MOA) of AGS-003. Therefore, it was of interest to test these combinations *in vitro* with DCs representative of AGS-003 to observe the effects of combination therapy on antigen-specific CTL proliferation and CTL functional responses.

**Methods**

DCs derived from normal donor monocytes were co-electroporated with MART-1 RNA and CD40L RNA to represent AGS-003 DC products. *In vitro* co-cultures were set up with autologous CD8^+^ T cells and MART-1/CD40L-DCs in the presence of various concentrations of the kinase inhibitors. Kinase inhibitor concentrations were chosen to represent steady-state concentrations reported in patients receiving active therapy. Subsequent expansion of MART-1 specific CTLs and multi-functional responses were mapped using multi-color flow cytometry.

**Results**

Our *in vitro* analysis demonstrated that sunitinib, axitinib, cabozantinib, tivozanib, everolimus and temsirolimus did not impact the priming nor proliferation of MART-1-specific CTL responses. Furthermore, these kinase inhibitors did not impact multi-functionality of CD28^+^/CD45RA^−^ effector/memory CTL. However, sorafenib, when present in the CTL/DC co-cultures, did significantly impair anti-MART-1-specific CTL expansion and CTL multi-functionality.

**Conclusions**

Autologous DCs co-electroporated with MART-1 RNA/CD40L RNA exhibit a similar MOA *in vitro* to AGS-003 administered *in vivo*, whereby both DC preparations induce antigen-specific multi-functional CTLs. Understanding the MOA of AGS-003 *in vitro*, allows for the testing of a broad range of potential combination therapies to provide feasibility data to support clinical trials of combination therapy for mRCC. Data provided show that most, but not all, kinase inhibitors are compatible with the MOA of AGS-003, the induction of effector/memory CTL responses, and that each therapeutic agent warrants testing.

**Trial Registration**

ClinicalTrials.gov identifier NCT01582672.

### P252 Multiple tumor antigen-activated T cell therapy elicits Individual and dynamic T cell responses in patients with hepatocellular carcinoma

#### Yanyan Han^1^, Yeting Wu^2^, Chou Yang^2^, Jing Huang^2^, Dongyun Wu^3^, Jin Li^3^, Xiaoling Liang^1^, Xiangjun Zhou^3^, Jinlin Hou^2^

##### ^1^R&D Department, HRYZ Biotech Co., Shenzhen, Guangdong, People’s Republic of China; ^2^Department of Infectious Diseases and Hepatology Unit, Nanfang Hospital, Guangzhou, Guangdong, People’s Republic of China; ^3^HRYZ Biotech Co., Shenzhen, Guangdong, People’s Republic of China

###### **Correspondence:** Yanyan Han (hanyy@thyx.com)

**Background**

We have previously reported that the immunotherapy with multiple tumor antigens activated autologous T cells (MASCT) was a safe treatment, which may improve the immunologic function and clinical outcome of the patients with hepatocellular carcinoma (HCC). In this study, we investigated the dynamics of MASCT-induced immune responses and demonstrated the mechanism and advantages of using multiple tumor antigens.

**Methods**

13 patients with stage B stage (BCLC) were treated with MASCT for three courses after tumor resection. During each course, the patients received two subcutaneous injections of mature dendritic cells (mDCs) pulsed with a peptide pool of multiple tumor antigens, and three i.v. injections of autologous T cells activated by mDCs described above. Each course lasted 14–15 weeks.

**Results**

After repeated treatment of MASCT, the frequency of regulatory T cells in the patients’ PBMCs was significantly decreased, while antigen peptide pool-triggered T cell proliferation and IFNγ-production were significantly enhanced in the patients’ PBMCs. Moreover, the specific immune responses of T cells against each kind of tumor antigen peptide in the pool were also measured by IFNγ ELISPOT assay. These specific immune responses could be detected in 11 out of 13 patients’ PBMCs but with individual and dynamic patterns during the treatments of MASCT. After 1 course of treatment, the best patient has specific immune responses against 9 tumor antigens out of 14 in the pool, and the worst patient has responses against 2 tumor antigens. These numbers have increased to 11 and 3 after the second course. The most immunogenic tumor antigens are survivin (7/13), cyclin D1 (CCND1, 6/13), carcinoembryonic antigen (CEA, 5/13), and HBV DNA polymerase (5/13). There were 7 patients left without progression 1 year after the immunotherapy initiation. And, the specific immune responses detected in these patients’ PBMCs were significantly stronger than that in the patients with progression.

**Conclusions**

Our study demonstrates that individual and dynamic tumor antigen-specific T cell responses can be induced in HCC patients after repeated treatments of MASCT, providing evidence to show the advantage of using multiple tumor antigens in immunotherapy instead of single antigen. In addition, these specific immune responses may correlate with the clinical outcomes.

### P253 Live, attenuated, double-deleted Listeria monocytogenes expressing mesothelin (CRS-207) with immuno-modulatory doses of cyclophosphamide, combined with chemotherapy as treatment for malignant pleural mesothelioma (MPM)

#### Raffit Hassan^1^, Thierry Jahan^2^, Scott J Antonia^3^, Hedy L Kindler^4^, Evan W Alley^5^, Somayeh Honarmand^6^, Weiqun Liu^6^, Meredith L Leong^6^, Chan C Whiting^6^, Nitya Nair^6^, Amanda Enstrom^6^, Edward E Lemmens^6^, Takahiro Tsujikawa^7^, Sushil Kumar^7^, Lisa M Coussens^7^, Aimee L Murphy^6^, Dirk G Brockstedt^6^

##### ^1^Thoracic and GI Oncology Branch, National Cancer Institute, Bethesda, MD, USA; ^2^Department of Medicine, Division of Hematology Oncology, UCSF, San Francisco, CA, USA; ^3^H. Lee Moffitt Cancer Center, Tampa, FL, USA; ^4^Gastrointestinal Oncology and Mesothelioma Programs, Section of Hematology/Oncology, University of Chicago, Chicago, IL, USA; ^5^Penn Prebyterian Medical Center, University of Pennsylvania, Philadelphia, PA, USA; ^6^Aduro Biotech, Inc., Berkeley, CA, USA; ^7^Oregon Health & Science University, Portland, OR, USA

###### **Correspondence:** Somayeh Honarmand (shonarmand@aduro.com)

**Background**

CRS-207 is live, attenuated, double-deleted *Listeria monocytogenes* (LADD) engineered to express mesothelin, a tumor-associated antigen over-expressed in several cancers, including MPM, an aggressive treatment-refractory disease with poor prognosis. CRS-207 activates innate and adaptive immunity and may act synergistically with chemotherapy to increase the susceptibility of the tumor microenvironment to immune-mediated killing. CRS-207 in combination with standard of care (SOC) pemetrexed/cisplatin demonstrated clinical activity in a phase Ib study. Low-dose cyclophosphamide (Cy) has been shown to decrease regulatory T cells and enhance vaccine-induced responses. Preclinical data demonstrate CRS-207 with low-dose Cy improves survival in a murine lung metastasis model.

**Methods**

60 patients were enrolled into 2 cohorts in this phase Ib study. Eligibility required unresectable, untreated MPM, ECOG 0 or 1, and adequate organ function. Patients in Cohort 1 received 2 CRS-207 2 weeks apart, 6 cycles pemetrexed/cisplatin 3 weeks apart, followed by 2 CRS-207 3 weeks apart. Clinically stable patients continued CRS-207 every 8 weeks. Patients in Cohort 2 received Cy (200 mg/m2) 1 day prior to each CRS-207. Safety, immunogenicity, tumor responses, survival and tumor markers were assessed.

**Results**

22 patients were enrolled into the Cy/CRS-207 cohort of this study; 77 % male, median age 70. The most common Cy/CRS-207 related adverse events (AEs) were grades 1/2 fever, chills, hypotension and nausea/vomiting, with no treatment-related serious AEs or deaths. As of August 2016, of 22 evaluable patients receiving Cy/CRS-207 + chemotherapy, 86 % (19/22) had disease control with 11 (50 %) whose best overall response was partial response (PR) and 8 (36 %) had stable disease. Tumor shrinkage was observed in 8/22 (36 %) patients including 3 PR after 2 doses of Cy/CRS-207 prior to chemotherapy initiation. Comprehensive immune profiling including multidimensional immunohistochemistry (IHC) analyses will be presented.

**Conclusions**

Addition of immune-modulating doses of Cy to a regimen of CRS-207 and SOC chemotherapy appears to be well-tolerated with no increase in toxicity compared to those receiving CRS-207 alone with chemotherapy. Preliminary results show signs of tumor activity following 2 doses of Cy/CRS-207 prior to chemotherapy (36 % tumor shrinkage) and the combination with chemotherapy resulted in 86 % disease control and 50 % response rate compared to published response rates of 20-41 % with chemotherapy alone. Immune analyses and further follow-up are warranted to evaluate the Cy/CRS-207 + chemotherapy regimen as a treatment for MPM.

**Trial Registration**

ClinicalTrials.gov identifier NCT01675765.

### P254 Phase Ib trial of RNActive® cancer vaccine BI1361849 (CV9202) and local radiotherapy in stage IV non-small cell lung cancer (NSCLC) patients with disease control after 1st-line therapy: updated clinical results and immune responses

#### Sven D Koch^1^, Martin Sebastian^2^, Christian Weiss^3^, Martin Früh^4^, Miklos Pless^5^, Richard Cathomas^6^, Wolfgang Hilbe^7^, Georg Pall^8^, Thomas Wehler^9^, Jürgen Alt^9^, Helge Bischoff^10^, Michael Geissler^11^, Frank Griesinger^12^, Jens Kollmeier^13^, Alexandros Papachristofilou^14^, Fatma Doener^1^, Mariola Fotin-Mleczek^1^, Madeleine Hipp^1^, Henoch S Hong^1^, Karl-Josef Kallen^1^, Ute Klinkhardt^15^, Claudia Stosnach^15^, Birgit Scheel^1^, Andreas Schroeder^15^, Tobias Seibel^15^, Ulrike Gnad-Vogt^15^, Alfred Zippelius^14^

##### ^1^CureVac AG, Tubingen, Baden-Wurttemberg, Germany; ^2^University Hospital Frankfurt, Medical Clinic II, Goethe University, Frankfurt, Hessen, Germany; ^3^Klinikum Darmstadt GmbH, Darmstadt, Hessen, Germany; ^4^Kantonsspital St. Gallen, St. Gallen, Switzerland; ^5^Kantonsspital Winterthur, Winterthur, Zurich, Switzerland; ^6^Kantonsspital Graubünden, Chur, Graubunden, Switzerland; ^7^Wilhelminenspital Wien, Wien, Austria; ^8^Fachkliniken Wangen, Wangen (Allgäu), Baden-Wurttemberg, Germany; ^9^J. Gutenberg University Hospital Mainz, Mainz, Rheinland-Pfalz, Germany; ^10^Thoraxklinik Heidelberg gGmbH, Heidelberg, Baden-Wurttemberg, Germany; ^11^Klinikum Esslingen GmbH, Esslingen, Baden-Wurttemberg, Germany; ^12^Pius Hospital Oldenburg, Oldenburg, Niedersachsen, Germany; ^13^Heckeshorn Lung Clinic, Berlin, Germany; ^14^University Hospital Basel, Basel-Stadt, Switzerland; ^15^CureVac AG, Frankfurt am Main, Hessen, Germany

###### **Correspondence:** Sven D Koch (sven.koch@curevac.com)

**Background**

Preclinical studies demonstrated that local radiotherapy (RT) acts synergistically with RNActive® mRNA vaccines to enhance anti-tumor effects and increase tumor-infiltrating lymphocytes. BI1361849 is a therapeutic vaccine comprising optimized mRNA constituents encoding six NSCLC-associated antigens. Interim data of a phase Ib study, employing local RT to increase the immune mediated tumor control by BI1361849, have been previously published [1]. Here we report results of immune response analyses as well as updated safety and efficacy data.

**Methods**

26 patients (pts) with stage IV NSCLC were enrolled in three cohorts based on histological and molecular NSCLC subtypes (squamous and non-squamous cell with/without activating EGFR mutations). Pts received two vaccinations with BI1361849 before local RT to a single tumor lesion was administered in four consecutive daily fractions of 5 GY. Vaccination was continued until start of subsequent anti-cancer therapy. Maintenance pemetrexed (mP) and EGFR-TKIs were allowed where indicated. Cellular and humoral immune responses were measured *ex vivo* by multifunctional intracellular cytokine staining, IFN-g ELISpot, and ELISA in pre- and post-treatment blood samples. The induction of humoral immune responses against 27 lung cancer antigens not encoded by the vaccine was measured by antibody array.

**Results**

26 pts were enrolled. 15 pts received mP, two received EGFR TKIs. Most frequent AEs were mild to moderate injection-site reactions and flu-like symptoms. No BI1361849-related SAEs were reported. Based on preliminary data following up to 110 weeks of exposure, one confirmed PR was observed in a pt on mP, 13 pts (52 %) experienced SD (8 pts on mP, 2 pts on EGFR-TKI and 3 pts without concomitant maintenance treatment, associated with 15 % tumor shrinkage outside the radiation field in one of them). 25 pts were available for immune response analysis. Preliminary data indicate that BI1361849 was capable of eliciting antigen-specific immune responses in of the majority of the patients including cellular and humoral immune responses. Moreover all encoded antigens were immunogenic and responses against multiple antigens were observed. Treatment induced immune responses against other lung cancer antigens were detected in several patients.

**Conclusions**

BI1361849 can be safely combined with local RT and mP treatment. Shrinkage of non-irradiated lesions and prolonged disease stablization was observed in a subset of pts, mainly in combination with mP. Data indicate immunogenicity of BI1361849. Analyses of cellular and humoral immune responses will be updated, as well as updated clinical data.

**References**

1. *J Clin Oncol* 2016, **34(supl)**:Abstr e20627.

### P255 Overcoming resistance to tyrosine kinase inhibitor by natural killer (NK) cells in non-small cell lung cancer (NSCLC) cells

#### Ha-Ram Park^1^, Yong-Oon Ahn^1^, Tae Min Kim^2^, Soyeon Kim^1^, Seulki Kim^1^, Yu Soo Lee^1^, Bhumsuk Keam^2^, Dong-Wan Kim^2^, Dae Seog Heo^2^

##### ^1^SNU Cancer Research Institute, Seoul, Republic of Korea; ^2^Seoul National University Hospital, SNU Cancer Research Institute, Seoul, Republic of Korea

###### **Correspondence:** Ha-Ram Park (halam92@naver.com)

**Background**

Receptor tyrosine kinase signals are altered in NSCLC and tyrosine kinase inhibitors (TKIs) have been used to treat NSCLC harboring driver mutations (e.g. ALK fusion and EGFR). Although TKIs are sensitive to NSCLC with driver mutations, acquired resistance to TKIs is inevitable by various mechanisms including gatekeeper mutation and alternative pathway activation. Considering immunotherapy is one of the main strategies that override drug resistance and cancer stemness, we evaluated an immunologic strategy to overcome acquired resistance to TKIs using NK cells in NSCLC.

**Methods**

TKI-resistant NSCLC cell lines (H3122CR1, H3122LR1, H3122CR1LR1, EBC-R1, EBC-R2, PC-9GR, and PC-9ER) were established from NCI-H3122 (*EML4-ALK* fusion), EBC-1 (*MET* amplification), and PC-9 (*EGFR* exon 19 deletion) after continuous exposure to crizotinib, ceritinib, capmatinib, gefitinib, and erlotinib. NK cytotoxicity and antibody-dependent cell-mediated cytotoxicity (ADCC) using anti-EGFR monoclonal antibody (mAb) cetuximab were measured using ‘off-the-shelf’ NK92-CD16 cell line as effectors and detected by ^51^chromium-release assay. Expression of the ligands for NK cell receptors and total EGFR were analyzed by flow cytometry.

**Results**

Most of TKI-resistant NSCLC cell lines were more susceptible to NK92-CD16 cells compared with their parental cell lines. The percentage of cytotoxicity was determined to be 0.2 % in H3122 and 13.4 %, 30.2 % and 39.1 % in TKI-resistant H3122 group with an effector:target ratio of 30:1. (PC-9: 18.2 % vs. 38.8 % vs. 24.8 %). The expression of ICAM-1, which is a ligand for LFA-1 in NK cells, is higher in TKI-resistant NSCLC cells than in parental cells. When we blocked ICAM1-CD11a interaction during a cytotoxic assay, the cytotoxicity was decreased about 10 %. Cetuximab-mediated ADCC was higher in resistant cells due to the increased expression level of total EGFR in resistant cells.

**Conclusions**

TKI-resistant NSCLC cells are more sensitive to NK92 cell-mediated cytotoxicity that is partially dependent on up-regulation of ICAM-1 via an immunological synapse. In addition, cetuximab, an EGFR-targeting mAb, significantly increases NK cell cytotoxicity in TKI-resistant NSCLC cells. Taken together, NK-cell based immunotherapy with cetuximab might be feasible to treat NSCLC patients with acquired resistance to TKIs.

### P256 Intralesional injection with Rose Bengal and systemic chemotherapy induces anti-tumor immunity in a murine model of pancreatic cancer

#### Shari Pilon-Thomas, Amy Weber, Jennifer Morse, Krithika Kodumudi, Hao Liu, John Mullinax, Amod A Sarnaik

##### H. Lee Moffitt Cancer Center, Tampa, FL, USA

###### **Correspondence:** Shari Pilon-Thomas (shari.pilon-thomas@moffitt.org)

**Background**

Rose Bengal is a xanthene dye that has been utilized for liver function studies and is currently used topically in ophthalmology. Intralesional (IL) Rose Bengal (PV-10) has been shown in murine models and melanoma clinical trials to induce regression of treated melanoma lesions and uninjected bystander lesions. This study was undertaken to measure whether IL PV-10 can induce systemic anti-tumor effects alone or in combination with gemcitabine (Gem) therapy in a murine model of pancreatic cancer.

**Methods**

C57BL/6 mice received Panc02 pancreatic tumor cells subcutaneously (SC) on one flank to establish a single tumor. On day 7, tumor was treated with IL PV-10. Control mice received IL phosphate buffered saline (PBS). Tumor growth was measured. Splenic T cells were collected and co-cultured with Panc02 or irrelevant B16 cells. Supernatants were collected to measure Panc02-specific T cell responses by IFN-gamma ELISA. To measure the effect of IL PV-10 on the growth of an untreated, bystander tumor, mice received Panc02 cells in bilateral flanks. The resulting right tumor was injected IL with PV-10 or PBS. Tumor sizes were measured for both the right (treated) and left (untreated/bystander) tumors. To determine the efficacy of combination therapy with IL PV-10 and systemic Gem, mice bearing a single or bilateral Panc02 tumors were treated with PV-10 alone or in combination with Gem. Mice received 60 mg/kg Gem intraperitoneally (IP) twice per week.

**Results**

C57BL/6 mice bearing Panc02 tumors treated with IL PV-10 had significantly smaller tumors than mice treated with PBS (p < 0.001). A significant increase in the IFN-gamma production in response to Panc02 was measured in the splenocytes of mice treated with PV-10 as compared to mice treated with PBS (p < 0.05). Mice with bilateral tumors had a significant regression of tumors injected IL with PV-10 and there was a reduction in the untreated (bystander) flank Panc02 tumor (p < 0.01). Gem therapy in combination with IL PV-10 injection led to enhanced tumor regression (p < 0.05) compared to IL PV-10 or Gem alone in both a single tumor model and a bilateral tumor model.

**Conclusions**

Regression of untreated pancreatic tumors by IL injection of PV-10 in concomitant tumor supports the induction of a systemic anti-tumor response. Addition of Gem chemotherapy enhances the effects of IL PV-10 therapy. Given that patients with metastatic pancreatic cancer have a dismal prognosis, combination therapy of IL PV-10 combined with Gem may benefit patients with metastatic pancreatic cancer.

### P257 Multi-institution evaluation of outcomes following radiation and PD-1 inhibition

#### Luke Pike^1^, Andrew Bang^2^, Patrick A. Ott^3^, Tracy Balboni^1^, Allison Taylor^1^, Alexander Spektor^1^, Tyler Wilhite^1^, Monica Krishnan^1^, Daniel Cagney^1^, Brian Alexander^1^, Ayal Aizer^1^, Elizabeth Buchbinder^1^, Mark Awad^1^, Leena Ghandi^1^, F Stephen Hodi^3^, Jonathan Schoenfeld^1^

##### ^1^Brigham and Women's/Dana-Farber Cancer Center, Harvard University, Boston, MA, USA; ^2^Harvard Radiation Oncology Program, Boston, MA, USA; ^3^Dana-Farber Cancer Institute, Harvard University, Boston, MA, USA

###### **Correspondence:** Jonathan Schoenfeld (jdschoenfeld@partners.org)

**Background**

Preclinical models suggest radiation may synergize with immunotherapy; for instance, increased responses and prolonged survival have been observed in mice treated with radiation and either PD-1 inhibition or combined CTLA-4/PD-1 blockade. We previously observed that radiation was associated with favorable responses in melanoma patients treated with ipilimumab [1]. However, clinical data are lacking in regards to combining radiation with PD-1 inhibitors with or without CTLA-4 blockade.

**Methods**

We conducted an IRB-approved retrospective multi-institution analysis of patients with metastatic melanoma, non-small cell lung cancer (NSCLC), and renal cell carcinoma (RCC) treated at 6 centers with palliative radiation and PD-1 inhibitors, either before, after, or concurrent with radiation.

**Results**

137 patients (NSCLC, n = 79; melanoma, n = 48; RCC, n = 10) received 279 courses of radiation (median 2, range 1–6) and a median of 4 PD-1 inhibitor cycles (range 1–66). Sixteen patients received concurrent PD-1/CTLA-4 blockade. Sites irradiated included the brain (n = 144), spine (n = 40), lungs (n = 38), pelvis (n = 20), and other (n = 32); these sites, and the use of WBRT/SRS were balanced before versus after the start of PD-1 therapy. Median survival following start of anti-PD-1 therapy was 192, 394, and 121 days in patients with NSCLC, melanoma, and RCC, respectively. On multivariate analyses adjusting for histology, targetable mutations and concurrent PD-1/CTLA-4 inhibition, there was a significant association between radiation administered following the start of PD-1 directed treatment and improved survival (HR 0.59, p = 0.02). There was a significant interaction between the impact of concurrent PD-1/CTLA-4 checkpoint blockade and subsequent radiation on survival (p = 0.03 for interaction, HR for radiation = 0.28). One-year survival was 71 % in patients treated with radiation following PD-1/CTLA-4 blockade, and 4/8 of these patients continued to receive PD-1 therapy after radiation. In all patients, median survival from first course of brain-directed radiation was 634 days.

**Conclusions**

In this multi-institution retrospective analysis, targeted radiotherapy administered following PD-1 blockade was associated with increased survival. Although this finding is retrospective and therefore subject to bias, particularly striking is survival observed following brain-directed radiation and in patients treated with concurrent CTLA-4/PD-1 blockade. These data suggest select use of radiation in patients treated with anti-PD-1 therapy may allow for continuation of effective systemic immunotherapy that could augment long-term survival.

**References**

1. Chandra RA, Wilhite TJ, Balboni TA, *et al.*: **A systematic evaluation of abscopal responses following radiotherapy in patients with metastatic melanoma treated with ipilimumab.** *Oncoimmunology* 2015, **4(11)**:e1046028.

### P258 Combined blockade of CTLA-4 and CD47 with tumor irradiation extends survival in melanoma

#### Anthony L Schwartz^1^, Pulak R Nath^1^, Elizabeth Lessey-Morillon^1^, Lisa Ridnour^2^, David D Roberts^3^

##### ^1^National Cancer Institute, Bethesda, MD, USA; ^2^National Cancer Institute, Frederick, MD, USA; ^3^Department of Pathology, National Cancer Institute, Bethesda, MD, USA

###### **Correspondence:** Anthony L Schwartz (anthony.schwartz@nih.gov)

**Background**

Irradiation (IR) combined with chemotherapy is the post-surgical standard of care treatment for melanoma, but metastasis still results in high mortality rates. Recently, immune checkpoint inhibitors such as antibodies targeting cytotoxic T lymphocyte antigen-4 (CTLA-4) have proven effective for immunotherapy of melanoma. CTLA-4 is up-regulated post-T cell activation, and antibody blockade enhances tumor responses in immunocompetent rodents and humans. Ongoing trials suggest that combinations of immune checkpoint inhibitors are more efficacious than single agents, but tumors in many patients remain resistant. Our laboratory is investigating CD47 blockade for the treatment of cancer in several immune competent mouse models. CD47 expression is frequently elevated in cancers and serves as an inhibitory receptor for thrombospondin-1 on immune cells in the tumor stroma. CD47 blockade on CD8+ T cells or tumor cells significantly enhances immune-targeted tumor cell killing post-irradiation compared to irradiation alone. Here we explore the potential for CD47 blockade to improve the response rates to anti-CTLA-4 therapy alone or in combination with irradiation using a syngeneic mouse melanoma model.

**Methods**

C57BL/6 mice were inoculated with 1x10^6^ B16F10 melanoma cells into the right hind limb and treated with local 10Gy irradiation combined with CTLA-4 blocking antibody, CD47 translational blocking morpholino, or the combination of CTLA-4 antibody and CD47 morpholino. Subjects were humanely euthanized, and tumors were analyzed using qPCR to evaluate granzyme B and FOXP3 mRNA expression. Tumors were sectioned and subjected to H&E and anti-CD8 staining.

**Results**

In non-irradiated tumors, histology revealed minimal tumor necrosis, while all irradiated groups showed increased necrosis. Tumor IR in combination with CTLA-4 or CD47 increased immune cell infiltration. However, the combination of irradiation with CTLA-4 and CD47 showed widespread tumor necrosis encompassing the entire field. All groups treated with the CD47 morpholino also exhibited focal hemorrhage, which was more extensive when combined with CTLA-4. FOXP3 mRNA expression showed a two-fold increase in CD47/CTLA-4-treated mice, which further increased to 4-fold when administered with IR. Granzyme B mRNA expression increased 3.5 fold with the CTLA-4/CD47/IR combination. Overall survival in IR/CTLA-4 was ~50 % while the combination of IR/CTLA-4/CD47 blockade was 75 % at 50 days.

**Conclusions**

The results described herein suggest that IR in combination with CTLA-4 and CD47 checkpoint blockade can provide a survival benefit by activating beneficial adaptive immune signaling pathways.

### P259 Assessing the potential for enhanced antibody-dependent cell-mediated cytotoxicity (ADCC) by combining the CD137 antibody urelumab with rituximab or cetuximab in patients with refractory lymphoma or select advanced solid tumors

#### Neil H Segal^1^, Manish Sharma^2^, Dung T Le^3^, Patrick A Ott^4^, Robert L Ferris^5^, Andrew D Zelenetz^1^, Sattva S Neelapu^6^, Ronald Levy^7^, Izidore S Lossos^8^, Caron Jacobson^4^, Radhakrishnan Ramchandren^9^, John Godwin^10^, A Dimitrios Colevas^7^, Roland Meier^11^, Suba Krishnan^11^, Xuemin Gu^11^, Jaclyn Neely^11^, Satyendra Suryawanshi^11^, John Timmerman^12^

##### ^1^Memorial Sloan Kettering Cancer Center, New York, NY, USA; ^2^University of Chicago Medicine, Chicago, IL, USA; ^3^Sidney Kimmel Comprehensive Cancer Center at Johns Hopkins University, Baltimore, MD, USA; ^4^Dana-Farber Cancer Institute, Boston, MA, USA; ^5^University of Pittsburgh, Pittsburgh, PA, USA; ^6^University of Texas MD Anderson Cancer Center, Houston, TX, USA; ^7^Stanford University School of Medicine, Stanford, CA, USA; ^8^University of Miami Miller School of Medicine, Sylvester Comprehensive Cancer Center, Miami, FL, USA; ^9^Karmanos Cancer Institute, Detroit, MI, USA; ^10^Earle A. Chiles Research Institute, Providence Cancer Center, Portland, OR, USA; ^11^Bristol-Myers Squibb, Princeton, NJ, USA; ^12^UCLA Medical Center, Los Angeles, CA, USA

###### **Correspondence:** Neil H Segal (segaln@mskcc.org)

**Background**

Urelumab, a fully human CD137 agonistic monoclonal antibody (mAb) with single-agent pharmacodynamic and clinical activity in patients with lymphoma, has potential to enhance cytotoxic activity of natural killer (NK) cells when combined with antibody-based targeted therapies. The potential of urelumab to enhance ADCC/phagocytosis and thus improve efficacy was evaluated in phase Ib studies of urelumab combined with rituximab (anti-CD20 mAb) in patients with refractory B cell non-Hodgkin lymphoma or cetuximab (anti-EGFR mAb) in patients with refractory colorectal cancer (CRC) or squamous cell carcinoma of the head and neck. Here we report safety/tolerability, pharmacokinetics, pharmacodynamics, and preliminary clinical efficacy results from these trials.

**Methods**

During escalation in NCT01775631, patients with relapsed/refractory B-NHL received urelumab at 0.1 or 0.3 mg/kg or a flat dose of 8 mg (equivalent to 0.1 mg/kg in an 80-kg patient; initiated based on available pharmacokinetic and safety data) Q3W plus rituximab 375 mg/m^2^ QW (4 doses); during expansion, patients with relapsed/refractory diffuse large B cell lymphoma (DLBCL) or follicular lymphoma (FL) were treated with urelumab 8 mg plus rituximab. The NCT02110082 study evaluated urelumab (0.1 mg/kg or 8 mg Q3W) plus cetuximab (400 mg/m^2^ on week 1 and 250 mg/m^2^ Q1W thereafter) in patients with metastatic CRC or SCCHN. Primary endpoints were safety/tolerability.

**Results**

**Fig. 25 (abstract P259).**
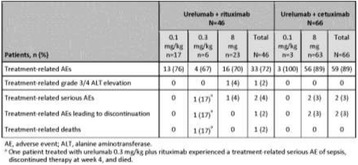
Treatment-related safety events

**Fig. 26 (abstract P259).**
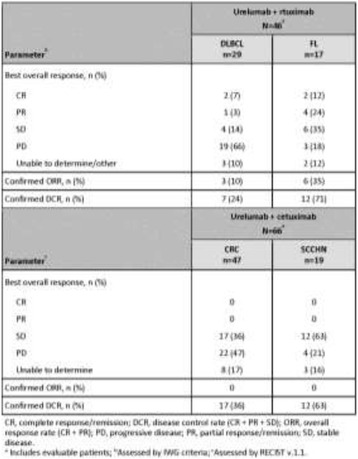
Efficacy of urelumab plus rituximab or urelumab plus cetuximab

Treated patients included those with DLBCL (n = 29), FL (n = 17), CRC (n = 47), and SCCHN (n = 19). Overall, 2–3 % of patients discontinued due to treatment-related AEs, and one patient experienced a grade 3/4 ALT elevation (Fig. 25). One patient who received urelumab plus rituximab experienced treatment-related sepsis and died. Apparent increases in IFNγ-induced cytokines were observed, and activated/proliferating CD8+ cells and cytotoxic NK cells appeared to increase in the periphery after 1 week of treatment with either regimen; however, in most tumors, the increase in CD8+ and NK cells was not observed. Overall response rates (ORRs) with urelumab plus rituximab were 10 % (3/29) in DLBCL and 35 % (6/17) in FL (Fig. 26). There were no confirmed responses with urelumab plus cetuximab in patients with CRC or SCCHN.

**Conclusions**

Urelumab is safe and well tolerated in combination with rituximab or cetuximab at doses of 0.1 mg/kg or 8 mg, with minimal evidence of liver toxicity. Although pharmacodynamic activity was observed in peripheral blood samples, urelumab with rituximab or cetuximab did not demonstrate substantial enhancement of clinical responses or lead to intratumoral immune modulation in these tumor settings.

**Trial Registration**

ClinicalTrials.gov identifier NCT01775631 and NCT02110082.

### P260 ATM is essential for the radiation-induced upregulation of the immunosuppressive cytokine activin-A by breast cancer cells

#### Claire I Vanpouille-Box, Silvia C Formenti, Sandra Demaria

##### Weill Cornell Medicine, Department of Radiation Oncology, New York, NY, USA

###### **Correspondence:** Claire I Vanpouille-Box (clv2002@med.cornell.edu)

**Background**

Activin A (actA) is a member of the transforming growth factor beta (TGFβ) superfamily. Recent evidence suggests that actA may facilitate tumorigenesis in part by suppressing immunity in the tumor microenvironment [1]. Treatment-induced DNA double-strand breaks (DSBs) induce actA mRNA and protein [2]. The ataxia telangiectasia-mutated (ATM) kinase is activated at DNA DSBs caused by genotoxic agents such as radiotherapy (RT) and is critical for DNA repair. Here we tested the hypothesis that induction of actA by RT limits RT-induced activation of anti-tumor immunity.

**Methods**

To test this hypothesis, 4 T1 mammary carcinoma cells were engineered to express a doxycycline (dox) inducible shRNA silencing inhibin A (*Inhba*, gene encoding for actA) (4T1^shInhba^). 4T1^shInhba^ or its non-silencing control (4T1^shNS^) were exposed to ionizing radiations to determine *Inhba* gene expression by RT-qPCR as well as secretion of actA by ELISA. To determine if ATM controls the expression of actA, derivatives with inducible knockdown of ATM were also generated (4T1^shATM^). 4T1^shInhba^, 4T1^shATM^ and 4T1^shNS^ were injected s.c. in syngeneic BALB/c mice on day 0. Knockdown of *ATM* and *Inhba* genes was induced by dox at day 8. Tumors were irradiated with 6 Gy repeated on days 13, 14, 15, 16 and 17. Mice were monitored and euthanized at day 22 and day 28 for evaluation of immune cells infiltration into the tumor.

**Results**

RT upregulated actA expression and secretion by 4 T1 cells. Secreted actA promoted CD4+ T cells conversion into regulatory T (Tregs) cells. *In vitro*, knockdown of ATM abolished both *Inhba* gene expression and actA secretion by tumor cells after RT. *In vivo*, this resulted in reduced Tregs infiltration in irradiated tumors, and increased activation of intra-tumoral CD8+ T cells. 4T1^shInhba^ and 4T1^shATM^ tumors showed an increased response to RT compared to 4T1^shNS^ tumors.

**Conclusions**

These data suggest that ATM plays a critical role in RT-induced actA secretion, which promotes an immunosuppressive environment in the irradiated tumor. Inhibition of ATM may increase tumor radiosensitivity and at the same time enhance *in situ* vaccination by radiation by hindering Treg generation.

**Acknowledgements**

Supported by DOD BCRP post-doctoral fellowship W81XWH-13-1-0012.

**References**

1. Loomans HA, *et al.*: *Cancers (Basel)* 2014.

2. Fordyce C, *et al.*: *Cancer Prev Res* 2010.

### P261 Adenosine regulates tumor response to radiation by hindering recruitment and activation of CD103+ DCs

#### Erik Wennerberg^1^, Aranzazu Mediero^2^, Bruce N Cronstein^2^, Silvia C Formenti^3^, Sandra Demaria^3^

##### ^1^Weill Cornell Medical College, New York, NY, USA; ^2^New York University Langone Medical Center, New York, NY, USA; ^3^ Department of Radiation Oncology, Weill Cornell Medicine, New York, NY, USA

###### **Correspondence:** Erik Wennerberg (erw2010@med.cornell.edu)

**Background**

Preclinical data and clinical observations support the concept that localized radiation therapy (RT) can be a powerful adjuvant to immunotherapeutic strategies by triggering *de novo* anti-tumor immune responses to poorly immunogenic tumors. We have previously shown that radiation induces the release of ATP in a dose-dependent manner [1]. ATP enhances recruitment and activation of dendritic cells (DCs), including CD103^+^ DCs recently identified as the key DC subset responsible for cross-presentation of tumor-derived antigens to CD8+ T cells. To determine whether rapid conversion of ATP to immunosuppressive adenosine could contribute to the limited ability of high dose RT to activate anti-tumor immunity we inhibited the rate-limiting CD73 ectonucleotidase.

**Methods**

Wild type (WT) or BATF3^−/−^ mice (ablated development of CD8a+/CD103+ DCs) were inoculated s.c. with the poorly immunogenic breast cancer cell line TSA on day 0 and assigned to treatment with: (1) control Ab; (2) anti-CD73 (TY/23 Ab); (3) RT (20 Gy); (4) RT + TY/23. TY/23 (200 μg) was administered i.p. on day 11, 14, 17 and 20. RT was given locally to the tumor as single 20 Gy dose on day 12. On day 18, some tumors were harvested for flow cytometry analysis of DCs and T cells. Mice were monitored for tumor progression by caliper measurements.

**Results**

In RT-treated mice, blockade of ADO generation by anti-CD73 resulted in increased infiltration of CD103 + DCs (8.9 ± 2.6 % of DCs in RT + TY/23 v. 3.5 ± 2.8 % of DCs in RT) expressing elevated levels of activation markers CD40 and CD86 compared to mice treated with RT alone. This change was associated with improved CD8 + T cell/Tregs ratio (5 ± 2.8 in RT + TY/23 v. 0.8 ± 0.2 in RT). Importantly, CD73 blockade had no effect by itself but improved significantly radiation-induced tumor control (tumor volume on day 57 post inoculation: 385 ± 525 mm^3^ in RT + TY/23 v. 1036 ± 727 mm^3^ in RT). Consistent with the hypothesis that CD103+ DC are essential for anti-tumor responses, the therapeutic effect of RT + CD73 blockade was abrogated in BATF3^−/−^ mice.

**Conclusions**

Our data indicate a key role of adenosine generated in the irradiated tumor in hindering development of anti-tumor immune responses and identify, as a mechanism of this effect, the inhibition of CD103+ DCs. Blockade of adenosine generation is a promising strategy to enhance radiation-induced anti-tumor immunity.

**References**

1. Golden EB, Frances D, Pellicciotta I, Demaria S, Helen Barcellos-Hoff M, Formenti SC: **Radiation fosters dose-dependent and chemotherapy-induced immunogenic cell death**. *Oncoimmunology* 2014, **3**:e28518.

## Diet, Exercise and/or Stress and Impact on the Immune System

### P262 The influence of exercise and fitness on the composition of leukocytes in peripheral blood; implications for cancer immunotherapy

#### Michael P Gustafson, AriCeli DiCostanzo, Courtney Wheatley, Chul-Ho Kim, Svetlana Bornschlegl, Dennis A Gastineau, Bruce D Johnson, Allan B Dietz

##### Mayo Clinic, Rochester, MN, USA

###### **Correspondence:** Michael P Gustafson (gustafson.michael@mayo.edu)

**Background**

Exercise immunology has become a growing field in the past 20 years, with an emphasis on understanding how different forms of exercise affect immune function. Overexertion may lead to suppressed immune function whereas moderate exercise may improve immunity. The improvement of immune function through exercise may benefit cancer patients receiving immunotherapy. To begin to test this hypothesis, we investigated the effects of acute and endurance exercise on the composition of peripheral blood leukocytes over time in a healthy male population of varying fitness.

**Methods**

Fifteen males participated in two cycling bouts; a short incremental exercise test to exhaustion on one visit and a 45 minute endurance exercise test (cycling at 60 % maximum workload) on the second visit. Lean body mass (LBM) and percent body fat (%BF) were calculated from DEXA (Dual-energy X-ray absorptiometry) scan on study visit 1. Flow-volume curves (FVC) were also collected on visit 1 with the average of 3 attempts within 150 mL of each other. Blood was collected at pre-exercise, immediately post-exercise, 3 hours post-exercise, and 24 hours post-exercise. Leukocytes were measured by multi-parameter flow cytometry of more than 50 immunophenotypes for each collection sample.

**Results**

We found a differential induction of leukocytosis dependent on exercise intensity and duration. Cytotoxic natural killer cells demonstrated the greatest increase (average of 5.6 fold) immediately post-maximal exercise whereas CD15^+^ granulocytes demonstrated the largest increase at 3 hours post-maximal exercise (1.6 fold). The longer, less intense endurance exercise resulted in an attenuated leukocytosis. Induction of leukocytosis did not differ in our limited study of active (n = 10) and sedentary (n = 5) subjects to exercise although we found that in baseline samples, sedentary individuals had elevated percentages of CD45RO^+^ memory CD4^+^ T cells and elevated proportions of CD4^+^ T cells expressing the negative immune regulator programmed death-1 (PD-1). Finally, we identified several leukocytes whose presence correlated with obesity related fitness parameters.

**Conclusions**

Taken together, our data suggests pre-existing compositional differences of leukocytes based on fitness and rapid and specific accumulation of leukocytes subsets into the blood dependent on the intensity and duration of to exercise.

**References**

1. Gustafson MP, Lin Y, Maas ML, Van Keulen VP, Johnson P, Peikert T, *et al.*: **A method for identification and analysis of non-overlapping myeloid immunophenotypes in humans.** *PloS One* 2015, **10(3)**:e0121546.

### P263 Blockade of β-adrenergic receptor signaling enhances anti-tumor immunity while increasing the sensitivity of tumor cells to radiation therapy

#### Cameron MacDonald, Mark Bucsek, Guanxi Qiao, Bonnie Hylander, Elizabeth Repasky

##### Roswell Park Cancer Institute, Buffalo, NY, USA

###### **Correspondence:** Cameron MacDonald (cameron.macdonald@roswellpark.org)

**Background**

Radiation therapy has evolved into an effective treatment for many cancers because of its ability to kill tumor cells and stimulate anti-tumor immunity, but radioresistance remains a major obstacle in cancer treatment. Recent studies indicate that norepinephrine (NE) released from sympathetic nerves suppresses immune cells and promotes tumor cell survival via β-adrenergic receptor (β-AR) activation. Work from our laboratory has shown that the cool housing temperature of laboratory mice is a significant source of cold stress which stimulates NE release. Moreover, we showed that β-AR signaling in mouse tumor models enhanced chemotherapeutic resistance, and treatment with β-AR antagonists (β-blockers) reversed this effect [1]. This finding led us to hypothesize that β-AR signaling promotes radioresistance in tumor cells but suppresses the anti-tumor immune response.

**Methods**

*In vitro,* we used Pan02 (abundant β-AR expression) and 4 T1 tumor cells (no β-AR expression). We treated cells with the pan-β-agonist, isoproterenol, and performed clonogenic assays examining survival at various radiation doses (0–8 Gy). *In vivo,* we implanted CT26.CL25 tumor cells subcutaneously into the leg of immunodeficient SCID mice and immune-competent BALB/c mice. When tumors became palpable, mice were randomized to receive daily β-blocker or PBS injections followed by radiation (6 Gy) 3 days later. Flow cytometry was used to analyze immune cells within tumors.

**Results**

*In vitro,* isoproterenol treatment significantly increased the survival of radiated Pan02 cells compared with controls but had no impact on radiated 4 T1 tumor cells suggesting that β-AR signaling enhances radioresistance. *In vivo,* radiation alone moderately slowed tumor growth in SCID mice. However, combining β-blockade with radiation significantly enhanced the effect of radiation, indicating that β-blockade was sensitizing tumor cells to radiation. Lastly, we repeated this experiment in immune-competent BALB/c mice and again observed a significant reduction of tumor growth in mice receiving β-blockade and radiation compared to radiation alone. To determine if β-blockade was enhancing anti-tumor immunity in radiated mice, we analyzed tumors using flow cytometry and observed a significant increase in CD4+ and CD8+ T cells expressing IFN-g and granzyme B in the group receiving β-blockade and radiation compared to those receiving radiation alone, indicating that β-blockade was also stimulating anti-tumor immunity.

**Conclusions**

Taken together, this data suggests that β-AR blockade decreases tumor cell radioresistance while simultaneously bolstering anti-tumor immunity.

**Acknowledgements**

Supported by: The Roswell Park Alliance Foundation.

**References**

1. Eng J, *et al.*: **Housing temperature-induced stress drives therapeutic resistance in murine tumour models through β2-adrenergic receptor activation.** *Nat Commun* 2015, **6**:6426.

### P264 The dual administration of immunotherapy is enhanced by activity-induced weight maintenance in the 4 T1.2 mammary tumor model

#### William J Turbitt, Yitong Xu, Andrea Mastro, Connie J Rogers

##### Pennsylvania State University, University Park, PA, USA

###### **Correspondence:** William J Turbitt (wjt5015@psu.edu)

**Background**

Lifestyle factors (e.g., body weight, physical activity) can impact breast cancer risk and response to therapy. Changes in metabolic, inflammatory, and immune mediators are possible factors underlying this relationship. Few studies have examined the effect of body weight and activity on the efficacy of immunomodulatory or immunotherapeutic strategies. Thus, the goal of the current study was to determine if preventing weight gain (via activity and diet) could delay primary tumor growth and metastatic progression in tumor-bearing mice and determine if there were any additive effects of weight maintenance and the dual administration of an allogeneic whole tumor cell vaccine and PD-1 checkpoint blockade on the aforementioned outcomes.

**Methods**

Female BALB/c mice were randomized to sedentary, weight gain (WG) or exercising, weight maintenance (WM) groups (n = 20-24/group). After 8 weeks, all mice were orthotopically injected with 5x10^4^ luciferase-transfected 4 T1.2 cells into the fourth mammary fat pad and continued on their intervention for 35 days. After injection, mice were further randomized into vaccination (n = 9-12/group) or vehicle control (n = 11-12/group) groups and administered irradiated 4 T1.2 cells (VAX) or vehicle (VEH) control at day 7, 14, 21, and 28 post-tumor injection. Current studies are investigating if the dual administration of VAX plus PD-1 checkpoint blockade (10 mg/kg/mouse) is enhanced in WM tumor-bearing mice.

**Results**

All WM groups weighed significantly less than WG groups over the course of the study (p < 0.0001). There was a significant effect of both WM and VAX alone on primary tumor growth (p < 0.0001) and splenic IFNγ production (p = 0.010) and an additive effect of WM + VAX on primary tumor growth (p < 0.05), metastatic burden in lung (p = 0.0267) and heart (p = 0.0492), and the accumulation of splenic MDSCs (p = 0.021). A pilot study showed an additive effect of the dual administration of VAX and anti-PD-1 in WG mice; however, experiments investigating the dual administration of VAX and PD-1 checkpoint blockade in WM cohorts are currently underway.

**Conclusions**

These results demonstrate that activity-induced weight maintenance in combination with an allogeneic whole tumor cell vaccine is highly effective at delaying primary tumor growth and metastases, and reducing splenic MDSC levels in this metastatic model. Interventions that maintain body weight may yield significant additive benefit in multimodal immunotherapy treatment strategies.

**Acknowledgements**

This work is supported by NIH grant R21 CA209144.

### P265 Multi-species assessment of the impact of aging and obesity on T cell exhaustion

#### Sita Withers^1^, Ziming Wang^1^, Lam T Khuat^1^, Cordelia Dunai^1^, Bruce R Blazar^2^, Dan Longo^3^, Robert Rebhun^1^, Steven K Grossenbacher^1^, Arta Monjazeb^1^, William J Murphy^1^

##### ^1^University of California, Davis, Sacramento, CA, USA; ^2^University of Minnesota, Minneapolis, MN, USA; ^3^Dana-Farber Cancer Institute, Boston, MA, USA

###### **Correspondence:** Sita Withers (sswithers@ucdavis.edu)

**Background**

Reinvigorating exhausted T lymphocytes with immune checkpoint inhibitors has proved to be a particularly successful strategy for the treatment of cancer. There remains however, a great deal of variability in response and toxicity between patients. Our previous murine studies revealed that obesity and age significantly enhanced toxicity to stimulatory immunotherapy as a consequence of their resting pro-inflammatory state. Here, we characterize the resting exhaustion phenotype and functional characteristics of T lymphocytes in obese and aged individuals, within a range of species, in order to understand the differential impact checkpoint inhibition might have on these populations.

**Methods**

Diet induced obese (DIO) mice within various age groups were fed a high fat diet long-term prior to analysis of splenocytes. Peripheral blood was collected from non-human primates (NHP) and laboratory beagles stratified into lean and obese groups based on weight and body condition score, respectively. Flow cytometry was used to quantify relative expression of T cell exhaustion markers, in addition to assessing T cell function as determined by intracellular cytokine expression and Ki67 positivity.

**Results**

We observed elevated PD-1 expression in CD4+ and CD8+ T cells in ≥12-month-old (mo) mice compared to 7mo mice. This coincided with altered expression of TNF-α and IFN-γ expression, and decreased proliferation following stimulation. Interestingly, PD-1 expression, proportions of naïve and effector memory cells, cytokine expression levels and proliferative responses of T cells from lean 13mo mice were similar to that of 7mo mice. Conversely, T cells from DIO 13mo mice appeared similar to 24mo mice in that they were phenotypically and functionally more exhausted. In NHPs, while PD-1 expression did not differ between small numbers of lean and obese animals, a trend towards decreased proliferation in CD4+ memory T cells of obese animals was observed. Similarly, decreased mitogen responses in T cells from obese dogs were also noted.

**Conclusions**

Both aging and obesity appear to impact PD-1 expression and T cell function. Data from dogs and NHPs suggest these findings may be consistent across species. Ultimately, these data add to our understanding of T cell function in differing physiologic states, and may provide the foundation for predicting toxicity and response to checkpoint inhibitors.

## Immune Metabolism

### P266 Disruption of the L-arginine balance in the tumor microenvironment with a recombinant human arginase 1 (AEB1102) sensitizes cancer to immunotherapy

#### Scott Rowlinson, Giulia Agnello, Susan Alters, David Lowe

##### Aeglea BioTherapeutics, Austin, TX, USA

###### **Correspondence:** Giulia Agnello (gagnello@aegleabio.com)

**Background**

Human arginase I (hArgI) is a Mn^2+^-dependent enzyme that displays low activity and low stability in serum. Myeloid-derived suppressor cells (MDSC) express human arginase I (hArgI) and nitric-oxide synthase (NOS), which control the availability of L-arginine in the tumor microenvironment and in turn regulate the function of T cells. Depletion of L-arginine by MDSC has been correlated to impairment of T cell anti-tumor function and tumor evasion of host immunity. Analysis of the expression of enzymes of the L-arginine biosynthetic pathway in peripheral blood mononuclear cells, bone marrow mononuclear cells and CD34^+^ cells showed low levels of ornithine transcarbamylase (OTC) and argininosuccinate synthase (ASS), suggestive of dependence of these cells on exogenous/extracellular L-arginine for physiological function. As a result, long term depletion of L-arginine may negatively impact the MDSC population and therefore enhance immune regulation of tumor growth. This hypothesis was tested using a recombinant hArgI (AEB1102), developed by replacement of the Mn^2+^ natural cofactor with Co^2+^ which results in significantly improved catalytic activity and serum stability as compared to endogenous hArgI.

**Methods**

Administration of AEB1102 results in chronic depletion of L-arginine in serum to levels below 1 μM. The murine CT26 colon-cancer model was dosed with AEB1102 alone and in combination with anti-PD-L1 and anti-PD-1 monoclonal antibodies (mAbs).

**Results**

*In vivo* treatment of CT26 mice with AEB1102 monotherapy resulted in an increased life span (ILS) (46 %, p < 0.001) as compared to the untreated control group, whereas standard monotherapy using immunomodulatory antibodies that target PD-1 and PD-L1 resulted in a 0 % (p = 0.5) and 29 % (p = 0.002) ILS respectively. Of significance, combination therapy of AEB1102 with anti-PD-1 (ILS 67 %, p < 0.001) or PD-L1 (ILS 67 %, p < 0.001) mAbs resulted in additive and synergistic anti-tumor effect compared to AEB1102 alone and immunotherapy alone.

**Conclusions**

Collectively these results demonstrate that disrupting the L-arginine physiological balance in the tumor microenvironment inhibits tumor growth and further sensitizes the tumor to immunotherapy. AEB1102 is currently in phase I (monotherapy) clinical trials. These data open the possibility of clinical combination of AEB1102 with anti-PD-1 and anti-PD-L1 mAbs to further improve outcomes in cancer patients.

### P267 Metformin treatment synergizes with PD-1 blockade therapy by reducing tumor hypoxia

#### Nicole Scharping^1^, Ashley V Menk^2^, Ryan Whetstone^1^, Xue Zeng^1^, Greg M Delgoffe^1^

##### ^1^University of Pittsburgh, Pittsburgh, PA, USA; ^2^University of Pittsburgh Cancer Institute, Pittsburgh, PA, USA

###### **Correspondence:** Ashley V Menk (avm9@pitt.edu)

**Background**

Tumors create a suppressive microenvironment that prevents antitumor immunity through a number of mechanisms like recruitment of regulatory T cells or through ligation of co-inhibitory molecules such as PD-1. PD-1 blockade therapy has become a major success in cancer treatment; however, most patients fail to respond to anti-PD-1 therapy. It has become clear in recent years that the lack of nutrients and oxygen in the tumor microenvironment may also play an immunosuppressive role. Since T cell effector function is dependent on these nutrients to produce the energy needed, we hypothesized that changing this environment would improve the efficacy of immunotherapy.

**Methods**

B16 and MC38 were injected into mice and were treated when tumors were palpable with either 0.2 mg anti-PD-1 or hamster IgG isotype control, injected every 4 days intraperitoneally, and 50 mg/kg metformin or PBS, injected every 2 days intraperitoneally. CD8+ T cells were isolated from lymph nodes and tumors and tested for hypoxia and cytokine production by flow cytometry and metabolic function with Seahorse XFe96 Bioanalyzer. Similar analysis was done *in vitro* with B16, MC38, and SIINFEKL stimulated OT-1 cells harvested from spleen and lymph nodes of mice.

**Results**

We show that B16, a melanoma resistant to PD-1 therapy, and MC38, a colon adenocarcinoma partially sensitive to PD-1 blockade, consume different amounts of oxygen and as a result, produce distinct hypoxic environments *in vivo*. The amount of hypoxia in the tumor microenvironment can be reduced by treating mice with metformin, a type II diabetes drug that inhibits mitochondrial complex I, which we show acts directly on the tumor cells to inhibit their oxygen consumption. As hypoxia can inhibit T cell responses *in vivo*, we hypothesized that metformin-induced mitigation of tumor hypoxia might improve immunotherapeutic responses. We also show that pairing metformin with PD-1 blockade therapy results in substantially improved antitumor immunity and tumor clearance, even in PD-1 insensitive tumor models.

**Conclusions**

Our data suggest that the degree of tumor hypoxia, partially a result of the degree of cancer cell metabolic deregulation, may determine whether T cells have a permissive microenvironment for effective immunotherapy. More importantly, our results suggest that metabolic remodeling of the tumor microenvironment may help patients better respond to immunotherapy.

### P268 Tumor-derived alpha-fetoprotein (tAFP) inhibits dendritic cell metabolism

#### Patricia M Santos^1^, Ashley V Menk^1^, Jian Shi^1^, Greg M Delgoffe^2^, Lisa H Butterfield^1^

##### ^1^University of Pittsburgh Cancer Institute, Pittsburgh, PA, USA; ^2^University of Pittsburgh, Pittsburgh, PA, USA

###### **Correspondence:** Patricia M Santos (santospm@upmc.edu)

**Background**

Previous studies have proposed an immune suppressive role for alpha-fetoprotein (AFP), an oncofetal antigen expressed by over 50 % of hepatocellular carcinomas (HCC). AFP-L3 is the major isoform present in the serum of HCC patients and is associated with poor patient prognosis. While tumor-derived AFP (tAFP) contains >80 % of AFP-L3, cord blood serum-derived AFP (nAFP) contains less than 5 % of AFP-L3. Our previous work shows that monocytes, cultured in the presence of AFP (in particular tAFP), differentiated into dendritic cells (DC), retained a monocyte-like morphology, had decreased expression of DC maturation markers, and exhibited limited production of inflammatory cytokines and chemokines. Importantly, monocyte-derived DC cultured in the presence of tAFP failed to stimulate antigen-specific T cell responses. In this study, we examined the effect of AFP on DC cellular metabolism.

**Methods**

Monocytes were isolated from healthy donor peripheral blood mononuclear cells and cultured for 5 days with IL-4 and GM-CSF in the presence or absence of 10 μg/mL ovalbumin (OVA), nAFP or tAFP (n = 3-5 HD per experiment). DC were collected and tested for 1) mitochondria levels and function (flow cytometry), 2) metabolic function by seahorse extracellular flux analyzer, and 3) expression of oxidative phosphorylation (OXPHOS)-related proteins, including electron transport chain proteins, and PGC1α levels (western blot and flow cytometry).

**Results**

DC cultured in the presence of nAFP and tAFP, had reduced mitochondrial mass and mitochondrial activity compared to OVA-DC. This was confirmed by a reduction in the basal oxygen consumption rate (OCR) in nAFP-DC and a more severe reduction in basal OCR in tAFP-DC. We also show that while mitochondrial metabolism is affected, glycolysis of DCs was not affected. Our data suggest that the changes observed in DC metabolism occur within 24 hours of AFP exposure and that tAFP inhibition of mitochondrial metabolism leads to a transient compensatory increase in glycolysis in the first 24–48 hours. We also show differences in the expression of mitochondrial electron transport proteins responsible for OXPHOS in DC exposed to nAFP and tAFP. Lastly, we show that there is reduced expression of a key mitochondrial regulator, PGC1α, in nAFP and tAFP-DC.

**Conclusions**

Collectively, these data show profound negative effects of AFP, specifically tAFP on mitochondrial metabolism. These novel findings elucidate a key mechanism of immune suppression in HCC and may lead to new therapeutic approaches to reverse tAFP effects.

### P269 Treg cells utilize lactic acid to fuel immune suppression in the tumor microenvironment

#### Ryan Whetstone^1^, Ashley V Menk^2^, Nicole Scharping^1^, Greg Delgoffe^1^

##### ^1^University of Pittsburgh, Pittsburgh, PA, USA; ^2^University of Pittsburgh Cancer Institute, Pittsburgh, PA, USA

###### **Correspondence:** Ryan Whetstone (rdw16@pitt.edu)

**Background**

The suppressive function of regulatory T (Treg) cells contributes significantly to the failure of cytotoxic T cells to eliminate cancer cells in the tumor microenvironment (TME). Within the TME, Treg cells retain their metabolic capacity as well as proliferative and suppressive functions, while conventional, effector T cells suffer reduced metabolic capacity in the energy substrate dearth environment. Thus, in the metabolically distinct TME, Treg cells may be bioenergetically supported by factors distinct from those that support conventional T cells. Lactic acid, a product of tumor glycolytic metabolism that is abundant in the TME has long been known to be immunosuppressive. We hypothesize that Treg cells in the TME utilize alternative metabolic substrates, particularly lactic acid, that allows them to maintain a significant level of suppression.

**Methods**

To test our hypothesis we employed lactic acid treatment of conventional and regulatory T cells, as well as generating a mouse line in which *Slc16a1*, encoding a lactate transporter, can be specifically deleted in Treg cells, constitutively or in a tamoxifen-induced manner. We also employed 13C-labeled lactic acid coupled to mass spectrometric analysis.

**Results**

Intratumoral Treg cells are highly proliferative in the tumor microenvironment. Lactic acid treatment of freshly isolated lymph node Treg cells supports enhanced proliferation, in striking contrast to the suppressive effects on conventional T cells. Intratumoral Treg cells upregulate monocarboxylate transporters (MCT 1, encoded by *Slc16a1* and MCT2, encoded by *Slc16a7*) which actively transport short chain carbons, including lactic acid, both into and out of cells. We found that MCT1 null Treg cells have a diminished suppressive capacity compared to MCT1 competent Treg cells. We also found that the pharmacologic inhibition of lactate metabolism in tumor-bearing animals results in dramatically decreased intratumoral Treg cell proliferation. Our ongoing studies seek to determine specifically how lactic acid is utilized by intratumoral Treg cells.

**Conclusions**

Our data strongly support a model in which tumor cells evade the immune response, in part, by metabolically supporting immunosuppressive populations. Targeting MCT1 in Treg cells and diminishing immune suppression may be an appealing target for therapeutic intervention, especially while combining MCT1 inhibition with a therapy that reestablishes cytotoxic T cell functions, such as anti-PD-1 checkpoint blockade.

## Immune-related Adverse Event Management: Evidence Based Strategies and Clinical Care

### P270 Management of immune-mediated diarrhea and the impact of infliximab on outcome

#### Alfonso Cortés Salgado^1^, Meghan Campo^2^, Anita Giobbie-Hurder^3^, Ainara Soria^1^, Donald P Lawrence^2^, Javier Cortés^1^, Ryan J Sullivan^2^

##### ^1^Medical Oncology Department, Hospital Universitario Ramón y Cajal, Madrid, Madrid, Spain; ^2^Medical Oncology Department, Massachusetts General Hospital, Boston, MA, USA; ^3^Department of Biostatistics & Computational Biology, Boston, MA, USA

###### **Correspondence:** Alfonso Cortés Salgado (acsalgado86@gmail.com)

**Background**

With the increasing use of immune checkpoint inhibitors (iCI), the recognition and prompt effective treatment of immune related adverse effects (irAEs), such as immune-mediated diarrhea (IMD) is crucial. IMD is commonly managed with steroid treatment and in severe cases, the TNF-alpha inhibitor infliximab (INF) however little is known on the impact INF has on patient outcomes.

**Methods**

We retrospectively reviewed 46 patients treated with iCI (anti-CTLA-4, anti-PD-1 or combination therapy) at Massachusetts General Hospital between 2008 and 2015 who developed grade 3–4 IMD with 96 % of cases confirmed on colonoscopy. The primary aim was to characterize patients who received intravenous INF 5 mg/kg (up to 3 doses) compared to those who did not and evaluate for impact of INF on outcomes. Kaplan-Meier and extended Cox regression were used to calculate OS and PFS.

**Results**

Of the 46 patients who developed IMD, 60.9 % were treated with CTLA-4 inhibitor monotherapy, 30.4 % with combination CTLA-4/PD-1 inhibition and 8.7 % with anti-PD-1 therapy alone. Median follow-up measured after the first dose of treatment causing diarrhea was 20.5 months (95 % CI: 13.0-25.1 months). Of the 46 patients, 2 (4.4 %) had neither steroids nor INF. Of the remaining 44, 17 (37 %) had both steroids and INF and 27 (59 %) had steroids alone. Of the 27 patients treated with steroids alone, 26 improved to grade 1 or better; median time of 25 days. For the 17 patients treated with steroids followed by INF, the median time on steroids prior to INF was 24 days; median time from start of symptoms to grade-1 resolution was 68 days. Of the 17 patients who received INF, 53 % required 1 dose, 35 % two doses, and 12 % three doses. When stratified by ECOG PS and adjusted for comorbidities, INF therapy was a significant predictor of PFS with a 76 % reduction compared with patients who had not yet started or never received the drug (HR: 0.24, 95 % CI: 0.1-0.8, p = 0.02). Treatment with INF was not a predictor of OS (HR: 0.97, 95 % CI: 0.3-3.7, p = 0.97).

**Conclusions**

Development of IMD is a common ir-AEs. The lag between development of IMD and the administration of INF in our study renders it difficult to determine if INF significantly shortens symptom duration however our data demonstrate the significance of INF use on PFS. Though this finding requires further validation in a larger patient cohort it does bolster the hypothesis that severe ir-AE, necessitating stronger immune suppression, may correlate with treatment efficacy.

### P271 Transaminitis on PD-1/L1 inhibitors; the difficulties in distinguishing among progression, tumor flare and autoimmune hepatitis

#### Misako Nagasaka, Ammar Sukari

##### Karmanos Cancer Institute, Detroit, MI, USA

###### **Correspondence:** Misako Nagasaka (nagasakm@karmanos.org)

**Background**

Autoimmune hepatitis is a well-documented rare but serious immune related event known to occur with anti PD-1/L1 inhibition and may mimic progression or tumor flare.

**Methods**

We evaluated three patients with liver metastasis who developed transaminitis with anti-PD-1/L1 inhibitors using CTCAE ver4.0 and RECIST ver1.1.

**Results**

Case 1: A 36 year old man with nasopharyngeal carcinoma with liver metastasis developed grade 2 transaminitis and RUQ pain after 2 doses of nivolumab. Ultrasound revealed stable liver metastasis but with hepatomegaly and new ascites. Nivolumab was discontinued and switched to taxol. His hepatomegaly and ascites resolved. Case 2: A 35 year old woman with nasopharyngeal carcinoma with liver metastasis developed grade 1 transaminitis after 3 doses of nivolumab. Progression of liver metastasis was seen on CT scan. Case 3: A 69 year old man with oropharyngeal cancer with liver metastasis was placed on a clinical trial utilizing tremelimumab and MEDI4736. He had grade 1 transaminitis with stable disease on CT scan post 4 cycles. He continued on MEDI4736, developed grade 2 transaminitis and CT scan showed progression of liver metastasis. He was switched to carboplatin/5FU with weekly cetuximab and his liver enzymes normalized soon after. These observations raise the issue of the difficulties in distinguishing autoimmune hepatitis vs tumor progression vs tumor flare in patients with liver metastasis on PD-1/L1 inhibitors. Case 1 appears to have had autoimmune hepatitis, whereas others had progression. Drug-related hepatitis is seen in 1 % to 2 % of patients on checkpoint inhibitors. Grade 3/4 transaminitis may require high-dose corticosteroids [1]. Toxicities with PD-1/L1 antibodies may also vary with the histology. For example, in NSCLC patients receiving nivolumab, pneumonitis was observed in 6 % [2] whereas only 1.9 % of melanoma patients had pneumonitis [3]. Similarly, liver metastasis patients on PD-1/L1 therapy may be more prone to autoimmune hepatitis.

**Conclusions**

Close monitoring of the liver enzymes as well as careful assessment of liver are imperative while we await further “big data” on better consensus to evaluate transaminitis following checkpoint inhibitors in patients with liver metastasis.

**References**

1. Weber JS, *et al.*: **Toxicities of Immunotherapy for the Practitioner.** *J Clin Oncol* 2015, **33**:2092–2099.

2. Brahmer J, *et al.*: **Nivolumab versus Docetaxel in Advanced Squamous-Cell Non–Small-Cell Lung Cancer.** *N Engl J Med* 2015, **373**:123–135.

3. Weber JS, *et al.*: **Nivolumab versus chemotherapy in patients with advanced melanoma who progressed after anti-CTLA-4 treatment (CheckMate037): a randomised, controlled, open-label, phase 3 trial.** *Lancet Oncol* 2015, **16**:375–384.

**Consent**

Informed consent was obtained from the patients.

## Immunogenomics and Oncogenetics

### P272 Cognate pairing of human T cell receptor alpha and beta V-regions from single cells

#### Miranda Byrne-Steele, Wenjing Pan, Xiaohong Hou, Brittany Brown, Mary Eisenhower, Jian Han

##### iRepertoire, Inc., Huntsville, AL, USA

###### **Correspondence:** Miranda Byrne-Steele (msteele@irepertoire.com)

**Background**

Immune repertoire amplification of T cell receptors (TCR) coupled to next-generation sequencing (NGS) provides detailed sequence-level insight into the immune system. However, information related to the cognate pairing of TCR alpha and beta chains is lost once RNA extraction is performed on the bulk sample. We describe a sensitive method that allows for the amplification of both human TCR alpha and beta chains from single cells using amplicon-rescue-multiplex-PCR in the same reaction tube.

**Methods**

During the first round of PCR, reverse transcription and PCR is performed with nested, multiplex primers covering both the alpha and beta locus with communal forward and reverse binding sites included on the 5’ end of the inside primers. Included on the C-region gene primer is an in-line 6 nucleotide barcode, which serves as a plate identifier so that multiple 96-well (or 384-well) plates may be multiplexed in the same sequencing flow cell. After RT-PCR1, the first round PCR1 products are rescued using SPRI beads. A second PCR is performed with dual-indexed primers that complete the sequencing adaptors introduced during PCR1 and provide plate positional information for the sequenced products. Libraries from multiple plates may be pooled together on the same MiSeq flow cell and sequenced directly using a 500 or 600 cycle kit (250 paired-end read). An iPair GUI was also developed to enhance visualization of paired-receptors on the plate and to aid with identification of “interesting” pairs that warrant further investigation.

**Results**

This method was utilized in the analysis of a breast cancer patient’s peripheral CD4+ lymphocytes as part of a longitudinal study of the immune repertoire pre- and post-treatment. Under optimal conditions, the amplification success rate is greater than 90 %, with successfully identified pairs as high as 60 % of those amplified. In the described experiments, bulk sequencing of each chain was also performed on RNA from remaining cells, and the observed single cell alpha and beta chains are typically represented in the top 1 and 5 % of the most frequent clones from the bulk sample, respectively.

**Conclusions**

We have developed an extremely sensitive method for the identification of cognate pairs from single cells in the same reaction tube. Future work involves the characterization of single cells for gene products beyond the variable gene region of TCRs, allowing for a more complete characterization of the cellular landscape. With the rise of immunological-based treatments for cancer, this type of technology holds promise for uncovering TCR cognate pairs of therapeutic significance.

### P273 Defining molecular mechanisms of resistance to tumor immunity

#### Natalie Collins^1^, Robert Manguso^1^, Hans Pope^1^, Yashaswi Shrestha^2^, Jesse Boehm^2^, W Nicholas Haining^1^

##### ^1^Dana-Farber Cancer Institute, Boston, MA, USA; ^2^Broad Institute, Cambridge, MA, USA

###### **Correspondence:** Natalie Collins (natalie_collins@dfci.harvard.edu)

**Background**

Recent success of immune checkpoint blockade solidifies the importance of the immune system in the defense against cancer. The clinical impact of the immune response is, however, very heterogeneous, with some patients achieving dramatic responses while others fail to respond. Known genomic correlates of response to immunotherapy are not perfectly predictive of clinical outcome, supporting the existence of unknown mechanisms of resistance. We hypothesize somatic mutations account for heterogeneity in the spontaneous immune response and response to immunotherapy. We have undertaken a systematic *in vivo* screen to identify novel mechanisms of resistance to tumor immunity in order to define a comprehensive set of therapeutic targets and provide biomarkers of sensitivity to immunotherapeutic strategies.

**Methods**

Mouse tumor cell lines (MC38 colon carcinoma, B16 melanoma) were engineered to express a library of barcoded open reading frames (ORFs) mutagenized to encode cancer-associated somatic mutations from the Pan-Cancer analysis within The Cancer Genome Atlas (TCGA) [1]. These cell lines form tumors when implanted subcutaneously in immunocompetent animals. Tumor-bearing animals were then subjected to immunotherapy with either therapeutic vaccination or anti-PD-1 checkpoint blockade. Barcode relative representation was measured by next generation sequencing at the time of tumor implantation and at tumor harvest post-immunotherapy. Barcoded mutant ORFs that confer immune resistance increased in representation under immune pressure in comparison to untreated or immunodeficient animals.

**Results**

A mutation in Phospho-Inositol 3 Kinase (PI3K), PIK3CA c.3140A > G, consistently increased in representation in both B16 and MC38 immunotherapy-treated tumors but not in immunodeficient animals. This suggests that activity of this mutant allele conferred selective growth advantage in the setting of tumor immunity. This mutation encodes a constitutively active catalytic domain of PI3K, PIK3CA H1047R. MC38 tumors homogenously expressing PIK3CA H1047R and implanted into wild type mice failed to respond to anti-PD-1 therapy, while tumors expressing a control gene regressed after treatment with anti-PD-1. Pharmacologic PI3K inhibition resensitized tumors to treatment with anti-PD-1. PD-1-treated PIK3CA H1047R tumors had fewer infiltrating CD8+ T cells as measured by immunohistochemistry and flow cytometry of tumor infiltrating lymphocytes.

**Conclusions**

PI3K has, in addition to its well-described oncogenic role, a role in tumor immune evasion. As such, activating mutations in PI3K may be useful as a predictor of poor response to immunotherapy. Importantly, these findings also provide a rationale for therapeutic combination trials of immune checkpoint blockade and PI3K inhibition.

**References**

1. Kandoth C, *et al.*: **Mutational landscape and significance across 12 major cancer types**. *Nature* 2013, **502**:333–339.

### P274 A germline polymorphism associated with the T cell-inflamed tumor microenvironment in metastatic melanoma

#### Kyle R Cron^1^, Ayelet Sivan^1^, Keston Aquino-Michaels^1^, Thomas F Gajewski^2^

##### ^1^University of Chicago, Chicago, IL, USA; ^2^University of Chicago Medical Center, Chicago, IL, USA

###### **Correspondence:** Kyle R Cron (krcron@uchicago.edu)

**Background**

Baseline tumor infiltration with CD8+ T cells and an associated chemokine/interferon gene signature are correlated with a favorable clinical outcome to immunotherapies. Recent work has indicated that differential presence of this phenotype can be influenced by specific oncogene pathways activated within the tumor cells, and also by host commensal microbiota. However, germline polymorphisms among the patient population also could influence the degree of the natural immune response against a tumor.

**Methods**

To investigate this possibility, TCGA RNAseq data were used to analyze the degree of expression of a predetermined T cell gene signature, and germline SNP data from the same patients were utilized to perform a GWAS against the gene signature as a phenotype. The top hit, a minor allele of SNP rs1483185, was significantly associated with increased T cell gene expression in the tumor (p = 8.812e^−08^). Interrogation using the GTEX database revealed lower gene expression in association with this SNP, arguing for a loss-of-function phenotype. To explore this concept mechanistically, gene-targeted mice were utilized lacking this gene. To focus gene deletion on hematopoietic cells, bone marrow (BM) chimeras were utilized.

**Results**

Mice reconstituted with knockout BM grew B16.SIY tumors more slowly and had higher numbers of SIY^+^ CD8^+^ T cells in both the tumor infiltrating lymphocyte fraction as well as the spleen compared to WT engrafted mice. Additionally, 80 % of splenic SIY^+^ CD8^+^ T cells had an activated CD44^+^ CD62L^−^ phenotype in knockout recipients versus 40 % in WT recipients. The TIL populations from KO recipients also showed higher expression of surface molecules indicating antigen experience, as well as expression of PD-1, Lag3, and 4-1BB indicating antigen-specificity. Mechanistically, T cells from KO mice showed no obvious increase in activation potential. However, a significant increase in CD11c^+^ CD8^+^ dendritic cells was observed in KO BM recipients, suggesting a possible effect at the level of APCs.

**Conclusions**

Together, our results have identified a functionally relevant SNP that can lead to improved spontaneous anti-tumor immunity. The development of pharmacologic approaches to phenocopy this loss of function genetic phenotype may be attractive to pursue as a cancer therapeutic.

### P275 Graphene oxide and amino functionalized graphene characterization for immunotherapy applications using new high-throughput analysis

#### Marco Orecchioni^1^, Davide Bedognetti^2^, Wouter Hendrickx^2^, Claudia Fuoco^3^, Filomena Spada^3^, Francesco Sgarrella^1^, Gianni Cesareni^3^, Francesco Marincola^4^, Kostas Kostarelos^5^, Alberto Bianco^6^, Lucia Delogu^1^

##### ^1^University of Sassari, Sassari, Sardegna, Italy; ^2^Sidra Medical and Research Center, Doha, Ad Dawhah, Qatar; ^3^University of Roma Tor Vergata, Roma, Lazio, Italy; ^4^Research Branch, Sidra Medical and Research Center, Doha, Ad Dawhah, Qatar; ^5^Faculty of Medical & Human Sciences, University of Manchester, Manchester, England, UK; ^6^CNRS, Institute de Biologie Moléculaire et Cellulaire, Strasbourg, Alsace, France

###### **Correspondence:** Lucia Delogu (lgdelogu@uniss.it)

**Background**

There is an enormous interest in exploring the potentialities of novel nanomaterials such as graphene and its derivatives in biomedical applications [1]. The understanding of the biomolecular interactions of graphene nanomaterials with human cells is critical for their implementation as diagnostic or therapeutic agents [1]. In this context the immune modulation mediated by graphene oxide (GO) or nanomaterials in general could be interesting also in immunotherapy applications. Indeed nanotechnology can enhance the efficacy of immunostimulatory small molecules and biologics by altering their co-localization, biodistribution, and release kinetics [2]. The impact exerted by graphene and GO exposure on the immune system is still unknown.

**Methods**

Here, we propose an integrative analytical pipeline encompassing molecular and cellular characterization of the impact of graphene nanomaterials on immune cells. For the first time in the context of nanotechnology, we employed single cell mass cytometry to deconvolute the effect of GO flakes (between 1–2 graphene layers) functionalized by us through the addition of 1,3 dipolar-cycloaddition of amino groups (GONH_2_) on 15 immune cell populations looking at 30 markers at single-cell level. We then used whole transcriptomic analysis for further functional molecular characterization.

**Results**

**Fig. 27 (abstract P275).**
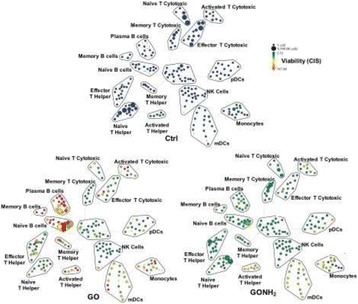
Viability analysis with single cell mass cytometry. The SPADE tree plot was constructed by using 17 cell-surface antigens in treated and untreated healthy human PBMCs. The size of each circle in the tree indicates relative frequency of cells that fall within the 17-dimensional confines of the node boundaries. Node color is scaled to the median intensity of marker expression of the cells within each node, expressed as a percentage of the maximum value in the data set (CIS is shown)

**Fig. 28 (abstract P275).**
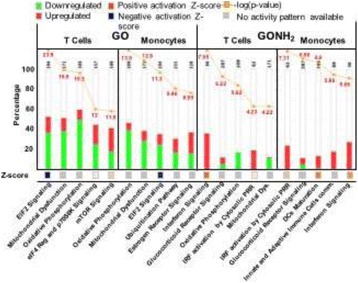
Microarray analysis. Top 5 first canonical pathways ranking according to significance level [Fisher exact test es log (p-value) reported in red] modulated by the GO and GONH2 in T cell (Jurkat cell lines) and monocytes (THP1 cell lines) cell lines identified using Ingenuity Pathway Analysis (IPA). The Z-score of each pathway is expressed under each column

Intriguingly functionalization is able to increase the biocompatibility of GO in all the sub-population analyzed (Fig. 27). GONH_2_ was found to be a potent immune-activator of monocytes and dendritic cells. This effect was confirmed and explained by the transcriptomic analysis. Notably, GO mainly modulated cell metabolism. GONH_2_ instead did not have an impact on the cell metabolism. Rather, they induce, in both monocytes and T cells, the activation of immunomodulatory pathways mainly related with innate and adaptive system and centered on interferon (IFN) signaling (Fig. 28). Immune activations resulted in increased expression of the T helper 1 chemokines (i.e., CXCR3 and CCR5 ligands), critical for the development of an effective anti-tumor immune response [3].

**Conclusions**

These idiosyncratic immune-modulatory properties of GONH_2_ represent the proof of principle to develop new nanoscale platforms in medicine as novel immunotherapeutic tools.

**References**

1. Orecchioni M, Bedognetti D, Sgarrella F, Marincola FM, Bianco A, Delogu LG: **Impact of carbon nanotubes and graphene on immune cells.** *J Transl Med* 2014, **12**:138.

2. Goldberg MS: **Immunoengineering: How nanotecnology can enhance cancer immunotherapy**. *Cell* 2015, **161(2)**:201–204. 3. Galon J, Angell HK, Bedognetti D, Marincola FM: **The continuum of cancer immunosurveillance: prognostic, predictive, and mechanistic signatures.** *Immunity* 2013, **39**:11–26.

### P276 Curated GEO dataset collection for discovery and validation of breast cancer immune phenotypes

#### Wouter Hendrickx^1^, Jessica Roelands^1^, Sabri Boughorbel^1^, Julie Decock^2^, Scott Presnell^3^, Ena Wang^5^, Franco M Marincola^1^, Peter Kuppen^4^, Michele Ceccarelli^5^, Darawan Rinchai^1^, Damien Chaussabel^1^, Lance Miller^6^, Davide Bedognetti^1^

##### ^1^Sidra Medical and Research Center, Doha, Ad Dawhah, Qatar; ^2^Qatar Biomedical Research Institute (QBRI), Doha, Ar Rayyan, Qatar; ^3^Benaroya Research Institute, Seattle, WA, USA; ^4^Leiden University Medical Center, Leiden, Zuid-Holland, Netherlands; ^5^Qatar Computing Research Institute, Doha, Ad Dawhah, Qatar; ^6^Wake Forest School of Medicine, Winston-Salem, NC, USA

###### **Correspondence:** Wouter Hendrickx (whendrickx@sidra.org)

**Background**

Sharing of -omics data has become the default modus operandi in recent years, with many funding sources and journals requiring authors to deposit their data in public repositories. This has led to a wealth of available datasets for *in silico* analysis or validation of new findings. To date, over 65,000 studies were deposited in the NCBI Gene Expression Omnibus (GEO) of which more than 1200 relate to breast cancer. However, these repositories are accompanied with minimal analysis and/or limited built-in visualization features in addition, offline analysis is relying on elevated bio-informatics skills. In order to make these datasets more accessible and interactive, we developed a web application called gene expression browser or GXB [1].

**Methods**

As a pilot project, we uploaded the GEO datasets we recently used to validate breast cancer immune subtypes to the GXB application and annotated the datasets collating data from the GEO database entries, the matching publications and added our own immune gene clustering assignments [2, 3].

**Results**

**Fig. 29 (abstract P276).**
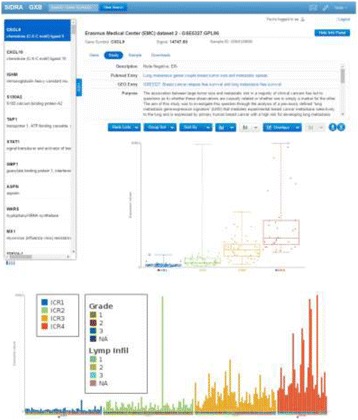
Representation of breast cancer transcriptome data by GXB. Example of CXCL9 gene expression visualization either by annotated bar chart or box-plot

A total of 2178 samples were imported as part of 14 GEO datasets, 1728 of which were used in our validation set. One dataset (GSE9151) was deposited in GEO in an incompatible format and had to be reprocessed from the raw CELL files into a normalized expression matrix. Adding group sets for clinical features, immunological gene clusters and generating matching gene rank lists will allow the users to quickly visualize differentially expressed genes. The addition of extra overlay data will enable the user to investigate correlations between phenotypic features. An example of the visualizations available in this application is demonstrated in Fig. 29.

**Conclusions**

The web application and the data set collection presented here demonstrate how data that is now secluded from many scientists can be made available in a more comprehensive and visually compelling environment. This approach will contribute to more effective data sharing and increased reproducibility of downstream bioinformatics pipelines.

**References**

1. Speake C, *et al.*: **An interactive web application for the dissemination of human systems immunology data**. *J Transl Med* 2015,**13**:196.

2. Miller LD, *et al.*: **Immunogenic subtypes of breast cancer delineated by gene classifiers of immune responsiveness**. *Cancer Immunol Res* 2016, **4(7)**:600–610.

3. http://breastcancer.gxbsidra.org/dm3/landing.gsp 4. Simeone I, *et al.*: **Toward the identification of genetic determinants of breast cancer immune responsiveness**. *J Immunother Cancer* 2015, **3(Suppl 1)**:P1.

### P277 Identifying patient-specific neoepitopes for vaccine immunotherapy reveals rarely shared recurrent neoepitopes

#### Andrew Nguyen^1^, J Zachary Sanborn^2^, Charles Vaske^2^, Shahrooz Rabizadeh^2^, Kayvan Niazi^2^, Steven Benz^2^

##### ^1^NantOmics, San Jose, CA, USA; ^2^NantOmics, Santa Cruz, CA, USA

###### **Correspondence:** Andrew Nguyen (Andy.Nguyen@nantomics.com)

**Background**

Targeted therapies for breast cancers such as trastuzumab and everolimus have durable clinical benefits for patients that express the relevant biomarkers (HER2 and mTOR respectively). Triple negative breast cancer patients lack these biomarkers and are left with few options. Recent advances in immunotherapy agents against PD-1/CTLA-4 for patients with melanoma have yielded amazing clinical benefits for a subset of patients and may have similar results in breast cancer patients, but again the vast majority of patients still undergo disease progression. We analyzed whole genome sequencing and RNA sequencing data from The Cancer Genome Atlas (TCGA) to identify neoepitopes across all cancer patients that could be used to develop next-generation, patient-specific cancer immunotherapies. Neoepitopes are tumor-specific markers that arise from mutations acquired from cancer and may represent a path to targeted therapies.

Our *in vitro* studies on human tumors and *in vivo* murine studies show that antagonism of the ER via tamoxifen

We analyzed 750 cancer patients from TCGA, containing a mixture of 23 different cancer classifications. These cancer patient samples were selected by the availability of whole genome sequencing (WGS) data, RNA-sequencing data as well as clinical outcome data.

**Results**

We identified an average of 680 potential neoepitopes per patient based solely on WGS data. To further refine and select high quality neoepitopes we restricted these neoepitopes based on gene expression yielding an average of 304 expressed neoepitopes per patient. We predicted each patient’s HLA typing using only omics data, which we then used to predict HLA-expressed neoepitope binding analysis resulting in an average of 11 high-quality tumor specific neoepitopes per patient. We identified few recurrent neoepitopes that were bound and expressed, indicating the need for a personalized medicine approach.

**Conclusions**

Within the TCGA dataset, the majority of neoepitopes among patients with breast cancer were unique to each patient. Rarely within subsets of breast cancers such as HER2+, do we identify neoepitopes that are shared between patients. For cancer patients who do not respond to targeted therapies, high-throughput identification of neoepitopes could serve as the basis for the development of next-generation, patient-specific immunotherapies.

### P278 A genome-scale CRISPR screen to identify essential genes for T cell based cancer therapies

#### Shashank Patel, Nicholas Restifo

##### National Cancer Institute, Bethesda, MD, USA

###### **Correspondence:** Shashank Patel (patelsj@mail.nih.gov)

**Background**

In responding to immune selection pressure mediated by T cells, somatic mutations can function in a bi-directional manner. On one hand, somatic mutations give rise to neoantigens capable of eliciting potent T cell responses. On the other hand, loss-of-function mutations or downregulation of gene expression in the tumor cell can also contribute to an immune evasion against T cells. Limited evidence exists on which gene perturbations in human cancer can directly or indirectly impair T cell mediated cytolysis.

**Methods**

To identify the genes essential in tumors to elicit a T cell-mediated cytolytic response, we developed a ‘two cell-type’ (2CT) CRISPR assay system consisting of human T cells as effectors and tumor cells as targets. We combined CRISPR-Cas9 screen datasets with TCGA gene expression datasets from >10,000 patient biopsies to study the patterns of immune sensitivity in human cancers.

**Results**

Using the 2CT genome-scale perturbation screen in melanoma cells, we identified and validated multiple genes whose loss impaired the effector function of T cells. Moreover, we uncover a group of core genes from these screens that correlates with cytolytic activity across the majority of the cancer types, reflecting context independence of these genes in the modulation of inherent T cell responses in multiple cancers. This dataset can be utilized to stratify patients for T cell based immunotherapies, and also for studying emergence of novel immune escape mechanisms.

**Conclusions**

This study demonstrates the broad applicability of 2CT CRISPR screens to study the interaction of cancer cells with immune cells and identify novel therapeutic targets for cellular therapies.

### P279 A non-invasive approach to assess tumor mutation load for treatment with cancer immunotherapy

#### James White, Sam Angiuoli, Mark Sausen, Sian Jones, Maria Sevdali, John Simmons, Victor Velculescu, Luis Diaz, Theresa Zhang

##### Personal Genome Diagnostics, Baltimore, MD, USA

###### **Correspondence:** Maria Sevdali (msevdali@personalgenome.com)

**Background**

Checkpoint inhibitors yield a significant clinical benefit for a subset of cancer patients. Given the high cost of these therapies and the time required to determine whether a therapy is efficacious, tests that can identify patients who are most likely to benefit are urgently needed. Supported by a strong biological rationale, tumor mutational load has emerged as a robust determinant of clinical benefit for multiple checkpoint inhibitors in multiple cancer types. However, existing approaches for assessment of tumor mutational load are expensive and rely on tumor specimens that are not readily available or may yield insufficient material for mutational load analyses.

**Methods**

We initiated the development of MutatorDetect, a plasma-based, non-invasive, cost effective method that can accurately identify late stage cancer patients whose tumors have high mutational load, regardless of the availability of tumor specimens. Previously, we have successfully developed an ultrasensitive platform for mutation detection using hybrid capture methodology and next generation sequencing. The platform is able to interrogate large exonic regions and detect cancer mutations from circulating cell free tumor DNA (ctDNA) in plasma with high sensitivity and specificity.

**Results**

We are currently implementing MutatorDetect using this platform and will present preliminary analytical and clinical validation data.

**Conclusions**

Further studies are warranted to examine MutatorDetect's performance in assessing tumor mutational load in large cohorts of late stage cancer patients and identifying patients who are likely to respond to checkpoint inhibitors.

### P280 Profiling abscopal regression in a pediatric fibrosarcoma with a novel EML4-NTRK3 fusion using immunogenomics and high-dimensional histopathology

#### Jennifer S Sims^1^, Sunjay M Barton^1^, Robyn Gartrell^1^, Angela Kadenhe-Chiweshe^1^, Filemon Dela Cruz^1^, Andrew T Turk^1^, Yan Lu^1^, Christopher F Mazzeo^2^, Andrew L Kung^1^, Jeffrey N Bruce^1^, Yvonne M Saenger^3^, Darrell J Yamashiro^1^, Eileen P Connolly^1^

##### ^1^Columbia University Medical Center, New York, NY, USA; ^2^Fordham University, New York, NY, USA; ^3^New York Presbyterian/Columbia University Medical Center, New York, NY, USA

###### **Correspondence:** Jennifer S Sims (jss2234@columbia.edu)

**Background**

The abscopal effect, or simultaneous tumor regression at multiple sites, is believed to result from the migration of T cells activated at the primary site to the distal sites. We have studied the case of a 1-year-old boy with congenital fibrosarcoma (forearm) bearing a novel EML4-NTRK3 fusion [1] who developed lung tumors 6 months after resection of the initial tumor. He received fractionated radiation therapy (RT) to the right lung only, but experienced complete regression of both right and left lung tumors.

**Methods**

Using whole exome sequencing (WES), high-throughput sequencing of peripheral and infiltrating T cell receptor repertoires (TCRseq), and multiplex immunohistochemistry (mIHC) using MANTRA (for biomarkers DAPI, CD3, CD4, CD8, CD68, HLA-DR, and Ki67), we investigated the relationship between tumor progression and metastasis, the pre- and post-treatment microenvironment, and the co-evolution of the tumor-infiltrating immune cell populations in this patient.

**Results**

Comparative WES of the initial fibrosarcoma and subsequent lung tumors identified the same EML4-NTRK3 fusion, validating their metastatic relationship, as well as the evolution of additional coding mutations upon recurrence. By both traditional and multiplex immunohistochemistry, the primary fibrosarcoma and pre-radiotherapy lung tumor were infiltrated by a very low density of CD4+ and CD8+ T cells. This infiltration was markedly increased (both CD4+ and CD8+ T cells) post-radiotherapy, particularly in the non-irradiated lung lesion, which is consistent with the model that abscopal regression depends on infiltration by trans-acting anti-tumor T cells. TCRseq of the TIL populations revealed little similarity between those of the primary fibrosarcoma and the lung lesions. However, the TCR repertoires of the irradiated and non-irradiated sites following radiotherapy showed substantial similarity, sharing a larger percentage of CDR3 sequences with one another (6.7 % of combined total) than with the primary tumor (≤0.6 % combined total) or the pre-radiotherapy lung tumor (≤1.6 % combined total). Furthermore, the breadth of the TIL repertoire and its binding-associated divergence from the peripheral blood was greatest in the irradiated lung tumor following radiotherapy, suggesting a direct link between the evolution of trans-acting immunity and antigen-driven immunogenicity at that site.

**Conclusions**

Our results, particularly anti-tumor T cell specificities shared inside and outside of the radiation field, offer key insights into the mechanisms underlying abscopal tumor regression, which may in turn be targeted for enhancement through immunotherapy.

**References**

1. Tannenbaum-Dvir S, *et al.*: **Characterization of a novel fusion gene EML4-NTRK3 in a case of recurrent congenital fibrosarcoma.** *Cold Spring Harb Mol Case Stud* 2015, **1(1)**:a000471.

## Inflammation, Innate Immunity, and the Microbiome

### P281 Cyclic dinucleotides activating STING are a safe and effective treatment for premalignant tumors

#### Jason Baird^1^, Marka Crittenden^2^, David Friedman^1^, Hong Xiao^3^, Rom Leidner^1^, Bryan Bell^1^, Kristina Young^2^, Michael Gough^2^

##### ^1^Earle A. Chiles Research Institute, Robert W. Franz Cancer Center, Providence Portland Medical Center, Portland, OR, USA; ^2^Earle A. Chile Research Institute, Portland, OR, USA; ^3^Providence Portland Medical Center, Portland, OR, USA

###### **Correspondence:** Jason Baird (jason.baird@providence.org)

**Background**

As our ability to detect cancer improves, there is an opportunity to treat early tumors before they become invasive and metastatic. Most cancers are known to exhibit a premalignant state, where an expanded population of mutated cells disrupts normal tissue organization. This premalignant state can exist for years and may remain, resolve, or progress into invasive cancer. Cancer screening programs, for example those based around the Pap smear for cervical carcinoma, aim to identify these abnormal cells for intervention before further malignant transformation. HPV+ tumors represent a large proportion of anal, vulvar, vaginal, cervical, and head and neck carcinomas. Vaccination against human papilloma virus (HPV) can prevent HPV infection and thus prevent HPV-associated malignancies. However, once tumors develop, vaccination against the virus does not impact tumor progression. Current therapies for a positive pap smear include excision of the abnormal region, or ablation by freezing or lasers. Immunotherapies, including interferon alpha and imiquimod have been added to excisional therapies to decrease the rate of recurrence; however, in randomized clinical trials it was found that neither approach impacts the rate of recurrence of cervical dysplasia.

**Methods**

In the transgenic mouse model carrying the mutant tumor-driving genes Kras(G12D) and p53(R172H) that are controlled by expression of Cre under a pancreas-specific promoter, this results in progressive carcinogenesis through pancreatic intraepithelial neoplasia to pancreatic ductal adenocarcinoma. In addition, due to leaky expression of Cre, at variable penetrance these mice spontaneously develop papilloma of the face and vulva. In these mice the normal skin undergoes epidermal thickening that closely resembles HPV-associated papilloma in humans. Since innate immune molecules, such as interferon alpha, and innate stimuli, such as imiquimod, have shown efficacy but failed in randomized studies, there is a need for novel approaches to treat premalignant disease and this mouse model represents a novel model system to test new therapies.

**Results**

Recently, we and others have demonstrated that novel agents that activate the STimulator of INterferon Genes (STING) are strong inducers of type I IFN and TNFα and can cause rapid regression of a range of advanced tumors. For these reasons, we tested the effect of STING ligands on papilloma in mice.

**Conclusions**

We tested the effect of STING ligands on papilloma in mice. Treatment with STING ligands causes rapid regression of spontaneous murine papilloma, and may represent an advance in treatment of virus-associated and premalignant diseases.

### P282 Inflammatory activation via PKC-Syk pathway in macrophages lacking CD47-SIRPα restraint initiates potent phagocytosis toward cancer

#### Zhen Bian, Koby Kidder, Yuan Liu

##### Georgia State University, Atlanta, GA, USA

###### **Correspondence:** Zhen Bian (zbian@gsu.edu)

**Background**

The CD47-SIRPα interaction serves as the critical self-recognition mechanism in macrophage phagocytosis toward self-cells through inhibitory signaling via SIRPα cytoplasmic ITIMs and tyrosine phosphatase SHP-1/2. This signaling axis, thus, prevents macrophage phagocytosis toward cancer cells, which exploit this mechanism by upregulating expression of CD47 to evade immunological eradication. Despite this mechanism being suggestively imperative, deficiency of CD47-SIRPα does not manifest enhanced macrophage phagocytosis toward self- or cancer cells. Therefore, this phenomenon suggests that the CD47-SIRPα mechanism alone provides little insight necessary to explain when macrophages would target self- or cancer cells, which bear no ‘eat-me’ signal and thus do not activate macrophage phagocytosis.

**Methods**

Establishment of SIRPα^−/−^ mice. *Ex vivo* and *in vivo* determination of macrophage phagocytosis toward self- and cancer cells (i.e., B16 melanoma, MC38 colon cancer, LLC lung cancer and EL4 lymphoma). Identification of signaling mechanisms that activate macrophage phagocytosis toward self-cells.

**Results**

Mice depleted of SIRPα display activated macrophage phagocytosis toward self-RBC or engrafted B16 and LLC tumors under inflammatory conditions and/or treatment by LPS/IL-17. *Ex vivo* phagocytosis assays demonstrate that macrophages from SIRPα^−/−^ or CD47^−/−^, and even WT mice, are generally incompetent to attack self-cells albeit aggressive toward bacteria, fungi, apoptotic cells and immune complexes. Treatment of macrophages with IL-17, LPS, IL-6, IL-1β or TNFα, but not IFNɣ, markedly initiates potent phagocytosis toward self which leads to macrophage-mediated eradication of B16, LLC, MC38, and EL4 cancer cells, providing an absence of the CD47-SIRPα restraint. Capitalizing on cytokine/LPS-activated SIRPα^−/−^ macrophages targeting CD47-expressing cells, we enlist these macrophages against B16 melanoma and demonstrate complete elimination of lung metastases in SIRPα^−/−^ mice and in WT mice into which SIRPα^−/−^ macrophage-induced cancer immunity has been adoptively transferred. Mechanistic studies suggest that a PKC-Syk-mediated signaling pathway, to which IL-10 conversely inhibits, is required for activating macrophages toward cancer cells.

**Conclusions**

These findings demonstrate for the first time that macrophage phagocytosis toward cancer requires activation by inflammatory cytokines/factors that stimulate the PKC-Syk pathway. The ramifications to arise from the combination of both phagocytic activation and perturbation of the CD47-SIRPα interaction would lead to the eradication of cancer, especially toward those which therapeutic antibodies are yet unavailable. (*The work is currently in press for publishing in PNAS.*)

### P283 Anti-leukemia immunity following STING agonist therapy requires a type I interferon-independent mechanism

#### Emily Curran^1^, Xiufen Chen^2^, Leticia P Corrales^1^, Justin Kline^3^

##### ^1^University of Chicago, Chicago, IL, USA; ^2^Department of Medicine, University of Chicago, Chicago, IL, USA; ^3^Committee on Immunology and Department of Medicine, University of Chicago, Chicago, IL, USA

###### **Correspondence:** Emily Curran (ecurran@medicine.bsd.uchicago.edu)

**Background**

The Stimulator of Interferon Genes (STING) pathway has been implicated as a major mechanism by which the host innate immune system “senses” cancer. STING pathway activation leads to the production of several downstream cytokines, most notably type I interferon (IFN), which is essential for bridging innate and adaptive immune responses against solid tumors. Administration of STING agonists induces broad immunity against transplanted solid malignancies in a type I IFN-dependent manner. We have recently demonstrated potent efficacy of systemic STING agonist therapy in murine AML models. Unlike the activity of STING agonists in solid tumor models, we observed a major type I IFN-independent effect in mice with AML, leading us to hypothesize that other cytokines may be important for the effectiveness of STING agonist therapy in mice with systemic leukemia.

**Methods**

C1498 AML cells engineered to express the model SIY peptide antigen (C1498.SIY) were inoculated intravenously into syngeneic C57BL/6 or *Ifnar* ^*−/−*^ mice and mice were treated with the selective murine STING agonist, DMXAA (5,6-dimethylxanthenone-4-acetic acid), or vehicle control.

**Results**

Surprisingly, SIY/K^b^ pentamer and intracellular cytokine staining followed by flow cytometric analysis revealed a similarly marked expansion of functional SIY-reactive CD8^+^ T cells in both wildtype and *Ifnar* ^*−/−*^ mice. Survival following DMXAA treatment was significantly prolonged in wild-type, and to a lesser extent, in *Ifnar* ^*−/−*^ mice, suggesting that other downstream cytokines, such as TNF-a or IL-6 may partially mediate STING-dependent immune effects in leukemia-bearing hosts. For example, STING pathway activation led to significantly increased TNF-a levels in both wildtype and *Ifnar* ^*−/−*^ mice (3-fold and 6-fold, respectively). Based on the known inflammatory and immune-activating properties of TNF-a, we speculate that it may also be contributing to the positive effects of STING activation in leukemia.

**Conclusions**

Collectively, our data demonstrate that STING-mediated enhancement of anti-leukemia immunity are at least partially independent of type I IFN. In addition to type I IFN, other cytokines/pathways appear to be required to mediate the powerful immunotherapeutic effect of STING agonists in AML.TNF-a is significantly increased following STING activation and may be mediating these effects, but further investigation is needed. Finally, these results imply that the mechanisms by which STING activation mediates anti-cancer effects may differ considerably between solid and hematologic malignancies.

### P284 Opposing roles of natural killer cell subsets in a mouse model of acute myeloid leukemia and hematopoietic stem cell transplant

#### Cordelia Dunai, Ethan G Aguilar, Lam T Khuat, William J Murphy

##### University of California, Davis, Sacramento, CA, USA

###### **Correspondence:** Cordelia Dunai (cdunai@ucdavis.edu)

**Background**

Natural killer (NK) cells are lymphocytes that bridge innate and adaptive immune responses. The activity of NK cells is controlled by the integration of activating and inhibitory signals. NK cells can be divided into subsets termed licensed and unlicensed based on the expression of inhibitory receptors with varying affinity for MHC class I molecules. In general, licensed NK cells have higher cytotoxic potential than unlicensed NK cells. Hematopoietic stem cell transplant (HSCT) is a treatment for a number of hematological malignancies, including acute myeloid leukemia (AML). Patients who are not eligible to receive intensive cytoreductive therapy, are at risk for primary refractory disease, or are experiencing relapse, are all candidates for HSCT. NK cells are the first lymphocytes to recover post-HSCT and have been shown to be critical for an anti-leukemia response. Here we investigate the contribution of NK cell subsets to anti-leukemia responses in the context of a mouse model of HSCT.

**Methods**

C1498 acute myelogenous leukemia cells were administered to control mice and mice that received total body irradiation followed by syngeneic HSCT. Monoclonal antibodies were used to deplete the licensed or unlicensed NK cell subsets (anti-Ly49C/I and anti-Ly49G2, respectively). Survival was monitored continuously and immune reconstitution was characterized by flow cytometry on days 17 and 24 post-C1498 administration.

**Results**

Control mice with intact immune systems survived with AML longer than HSCT mice. In control mice, the depletion of licensed or unlicensed NK cells did not significantly affect survival time. However, in HSCT mice, the depletion of the unlicensed NK cell subset resulted in statistically significant prolonged survival when compared to licensed-depleted and non-depleted controls, suggesting an antagonistic role for unlicensed NK cells in combating AML post-HSCT. Immune phenotyping showed that unlicensed depletion led to increased numbers of the licensed population post-HSCT, without the same reciprocal expansion of unlicensed NK cells with licensed depletion.

**Conclusions**

Unlicensed NK cells limit the expansion of licensed NK cell post-HSCT, which has a detrimental effect on survival after C1498 challenge. NK cell subsets have non-overlapping roles, with the licensed playing the dominant anti-leukemia role post-HSCT, and the unlicensed negatively regulating the response of the licensed. Modulation of these opposing roles may be advantageous for clinical use of HSCT and NK cell therapies.

### P285 Harnessing tumor associated macrophages with a novel compound to enhance chemotherapy and checkpoint blockade in breast cancer

#### Jennifer Guerriero^1^, Alaba Sotayo^1^, Holly Ponichtera^2^, Alexandra Pourzia^1^, Sara Schad^1^, Ruben Carrasco^1^, Suzan Lazo^1^, Roderick Bronson^3^, Anthony Letai^1^

##### ^1^Dana-Farber Cancer Institute, Boston, MA, USA; ^2^Evelo Biosciences, Cambridge, MA, USA; ^3^Harvard Medical School, Boston, MA, USA

###### **Correspondence:** Jennifer Guerriero (jennifer_guerriero@dfci.harvard.edu)

**Background**

The breast tumor environment is complex and includes both neoplastic and immune cells. The most abundant immune cell population within breast tumors is tumor associated macrophages (TAMs). Macrophages have the ability to polarize into either M1 cells, which have potent anti-tumor capabilities, or M2 macrophages which promote tumor progression by stimulating tumor vasculature, invasion, metastasis, and can enhance tumor resistance to chemotherapy. Generally TAMs in breast tumors are considered M2 macrophages and a high tumor density of TAMs clinically correlates to both overall worse prognosis and increased metastasis. Several therapeutic strategies exist to modulate TAMs clinically, focusing on depleting or inhibiting TAMs. However, macrophage are required for optimal tumor regression in response to both chemotherapy and immunotherapy. Their embedded location and their untapped potential provide impetus to the discovery of strategies to turn them against tumors and to harness for cancer therapy. Therefore here, we describe a novel method to polarize pro-tumor macrophages to an anti-tumor phenotype.

**Methods**

We recently reported that a first in class selective class IIa HDAC inhibitor (TMP195) influenced human monocyte responses to colony stimulating factors CSF-1 and CSF-2 *in vitro*. Here, we utilize a macrophage-dependent autochthonous mouse model of breast cancer to demonstrate that *in vivo* TMP195 treatment alters the tumor microenvironment and reduces tumor burden and pulmonary metastases through macrophage modulation.

**Results**

TMP195 induces recruitment and differentiation of highly phagocytic and stimulatory macrophages within tumors. Furthermore, combining TMP195 with chemotherapy regimens or T cell checkpoint blockade in this model significantly enhances the durability of tumor reduction.

**Conclusions**

These data introduce class IIa HDAC inhibition as a novel means to harness the anti-tumor potential of macrophages to enhance cancer therapy.

**Fig. 30 (abstract P285).**
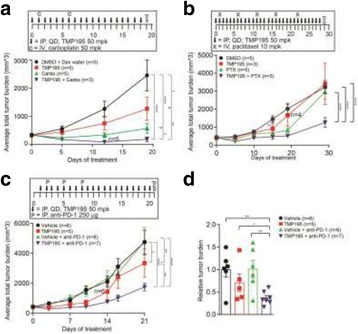
See text for description

### P286 Interferon production and immunity elicited by a constitutively active form of interferon-beta promoter stimulator-1 (IPS-1)

#### Richard S Kornbluth^1^, Sachin Gupta^2^, James Termini^2^, Elizabeth Guirado^2^, Geoffrey W Stone^2^

##### ^1^Receptome, LLC, La Jolla, CA, USA; ^2^Miller School of Medicine, University of Miami, Miami, FL, USA

###### **Correspondence:** Richard S Kornbluth (rkornbluth@receptome.com)

**Background**

Type I interferon (IFN) promotes the activity of anti-tumor CD8+ T cells. IFN induced by intratumoral injections of stimulator of interferon genes (STING) activators has shown remarkable efficacy as a “gas pedal” when combined with PD-1/PD-L1 checkpoint blockade in mouse models. Like STING, IPS-1 (also called MAVS, Cardif, or VISA) can lead to strong IFN production. IPS-1 is normally activated by viral RNAs bound to RIG-I which in turn modifies IPS-1 so that it clusters onto the surface of mitochondrial membranes. To circumvent the need for RIG-I, this study fused IPS-1 with the membrane clustering, N-terminal region of EBV latent membrane protein 1 (LMP1).

**Methods**

The N-terminus of the Epstein-Barr virus (EBV) LMP1 protein contains 6 transmembrane domains that self-associate and cluster in membranes. Genetically fusing this N-terminal region to IPS-1 forms LMP1-IPS-1 which is a constitutively activated form of IPS-1. The resulting nucleic acid sequence was tested either as mRNA, plasmid DNA, or by viral delivery using adenovirus 5 (Ad5) (Ad5-LMP1-IPS-1).

**Results**

Human dendritic cells transduced with Ad5-LMP1-IPS-1 upregulated CD80 and CD86 and produced large amounts of IL-12 and TNF. 293 cells transfected with LMP1-IPS-1 plasmid DNA produced high levels of IFN-α, IFN-β, and chemokines. By microarray analysis, mRNAs for IFN-associated genes were strongly upregulated. *In vivo*, mice vaccinated with Ad5-LMP1-IPS-1 plus Ad5 expressing HIV Gag were completely protected from vaccinia-Gag challenge virus.

**Conclusions**

LMP1-IPS-1 shares similar activities with activated STING. Whereas STING activators are small molecules, LMP1-IPS-1 is constitutively active and its nucleic acid sequence can be used as mRNA, plasmid DNA, part of a viral vector, or the payload of an “armed” oncolytic virus.

**Acknowledgements**

Supported by NIH grant R21AI093294 and Florida Dept. Health Technology Transfer Feasibility Award 4KF03.

**References**

1. Gupta S, Termini JM, Issac B, Guirado E, Stone GW: **Constitutively Active MAVS Inhibits HIV-1 Replication via Type I Interferon Secretion and Induction of HIV-1 Restriction Factors.** *PLoS ONE* 2016, **11(2)**:e0148929.

### P287 Modulation of Fcγ receptor function by HLA-G

#### Christina Meyer^1^, Laura Helming^2^, Joseph Tumang^3^, Nicholas Wilson^4^, Robert Hofmeister^5^, Laszlo Radvanyi^1^

##### ^1^EMD Serono, Billerica, MA, USA; ^2^Merck KGaA, Darmstadt, Hessen, Germany; ^3^Compass Therapeutics, Cambridge, MA, USA; ^4^Agenus Inc., Lexington, MA, USA; ^5^TCR2 Therapeutics, Cambridge, MA, USA

###### **Correspondence:** Christina Meyer (christina.meyer@emdserono.com)

**Background**

HLA-G is a non-classical major histocompatibility complex (MHC) molecule expressed at the fetal maternal interface and in tumors, where it exerts immunomodulatory effects. These immunosuppressive effects are mediated through interactions of HLA-G with its receptors, ILT2 and ILT4, which are expressed primarily on cells of the myeloid lineage. To further understand the effect of tumor HLA-G expression on myeloid immune suppression, we investigated the effect of HLA-G on Fcγ receptor signaling—a myeloid effector function which has important implications in the immune response to tumors and the effect of antibody-based therapeutics.

**Methods**

Using an inducible HLA-G-expressing tumor cell line and tumor-specific antibody, we examined Fcγ receptor effector functions in a human monocyte-like cell line as well as in primary human monocytes and macrophages.

**Results**

We found that HLA-G inhibits signaling downstream of FcγR activation in a monocytic cell line and in primary monocytes. Furthermore, HLA-G expression on tumor target cells results in inhibition of antibody-dependent phagocytosis by macrophages as well as decreased FcγR-induced production of inflammatory cytokines. These effects were dependent on HLA-G interaction with its receptors, ILT2 and ILT4.

**Conclusions**

Our findings suggest a role for HLA-G in promoting tumor immune escape, as well as a novel mechanism for HLA-G-mediated inhibition of antibody-based tumor cell phagocytosis by macrophages.

### P288 Melanoma cells upregulate innate immune genes in response to CTL attack

#### Natalie J Neubert^1^, Laure Tillé^1^, David Barras^2^, Charlotte Soneson^2^, Petra Baumgaertner^1^, Donata Rimoldi^1^, David Gfeller^1^, Mauro Delorenzi^2^, Silvia A Fuertes Marraco^1^, Daniel E Speiser^1^

##### ^1^University of Lausanne, Ludwig Cancer Research, Epalinges, Vaud, Switzerland; ^2^SIB Swiss Institute of Bioinformatics, Lausanne, Vaud, Switzerland

###### **Correspondence:** Daniel E Speiser (doc@dspeiser.ch)

**Background**

T cell-based immunotherapies have brought great progress for cancer patients. However, very little is known about the cancer cell response to cytotoxic T lymphocyte (CTL) attack.

**Methods**

We studied the dynamic T cell-cancer cell interplay. Low passage melanoma cell lines were cultured with MelanA/MART1-specific CD8+ T cells and characterized using differential gene expression analysis and flow cytometry. Specific gene products were investigated by immunohistochemistry and results were interpreted based on the literature including single cell data from melanoma patients.

**Results**

As expected, significant fractions of melanoma cells died in presence of melanoma-specific CD8+ T cells. However, still many melanoma cells persisted for up to three days of co-culture. Characterization of the surviving melanoma cells revealed increased mRNA levels of genes associated with an antimicrobial immune response: *DDX58, TLR3, AIM2, MyD88, NFKB, IFITM3,* and *ISG15* after co-culture. Upregulation of these genes is known to enhance innate immune responses. DDX58 (also RIG-I) and TLR3 sense intracellular viral RNA, and AIM2 recognizes cytosolic double-stranded DNA. MyD88 acts as an adaptor downstream of TLRs and signals to NFκB. IFITM3, a transmembrane protein, inhibits viral entry into its host and ISG15 has multiple anti-viral roles including induction of NK cell proliferation. These and further gene and protein expression changes were dependent on antigen-specific interaction of CTLs with melanoma cells and were mediated by IFNγ and TNFα, two cytokines secreted by CTLs upon antigen recognition.

**Conclusions**

So far, mainly immunosuppressive mechanisms of cancer cells upon exposure to CTLs have been described, such as increased expression of IDO1 and the inhibitory receptor ligand PD-L1. Here we show that in addition to these immunosuppressive mechanisms, melanoma cells respond to melanoma-specific CTLs with a cytokine-driven upregulation of genes involved in the innate immune response, likely also supporting the T cell responses. Moreover, melanoma cells upregulate further immune genes which we will discuss at the congress. Our findings may play a role in immunotherapy, and might explain why immune reactions in cancer patients are often promoting both pro- and anti-tumor immune mechanisms.

**Acknowledgements**

We thank J. Joyce, T. Petrova, M. De Palma, and R. Debets for discussions and advice, A. Wilson, C. Fumey, S. Winkler, T. Pillonel, N. Montandon and K. Mühlethaler for technical help, and B. van den Eynde for IDO-specific antibody.

## Other

### P289 Identification of T cell receptors targeting the intestinal self-antigen and colorectal cancer target GUCY2C by next-generation sequencing

#### Tara S Abraham, Bo Xiang, Michael S Magee, Scott A Waldman, Adam E Snook

##### Thomas Jefferson University, Philadelphia, PA, USA

###### **Correspondence:** Tara S Abraham (tara.abraham@jefferson.edu)

**Background**

Identifying T cell receptors (TCRs) targeting self-antigens may provide opportunities for effective cancer therapy employing adoptive T cell therapy. However, the enormous diversity of TCRs and low abundance of antigen-specific clones make identification of individual TCRs difficult. Moreover, tolerance further limits self-reactive TCR abundance and affinity.

**Methods**

Here, we define a process to identify TCRs from CD4^+^ T cells recognizing the intestinal self-antigen GUCY2C, an emerging target in colorectal cancer immunotherapy. GUCY2C-specific CD4^+^ T cells were identified and purified from GUCY2C-immunized mice. Next-generation sequencing of TCR transcripts quantified individual TCRs, permitting synthesis and assembly of TCR constructs that were engineered into CD4^+^ T cells.

**Results**

GUCY2C recognition by engineered CD4^+^ T cells *in vitro* confirmed TCR specificity and activity. Further, TCR constructs were engineered into purified mouse hematopoietic stem cells (HSCs) *ex vivo*, which were adoptively transferred to HSC-depleted recipient mice. These engineered HSCs gave rise to naïve T cells expressing GUCY2C-specific TCR transgenes, allowing us to validate our *in vitro* findings of GUCY2C recognition following GUCY2C vaccination.

**Conclusions**

This transgenic TCR model system can be employed to define tolerance mechanisms restricting GUCY2C-specific immunotherapy, facilitating 2^nd^-generation GUCY2C vaccine design. Moreover, this approach to identify and validate self-antigen specific TCRs may be employed for cancer immunotherapies, accelerating therapeutic discovery.

### P290 Clinical significance of immune-related factors on systemic trafficking of bone marrow-derived stem cells in patients with different types of gastric neoplasms

#### Wojciech Blogowski^1^, Ewa Zuba-Surma^2^, Marta Budkowska^3^, Daria Salata^3^, Barbara Dolegowska^4^, Teresa Starzynska^5^

##### ^1^Department of Internal Medicine, University of Zielona Gora, Zielona Gora, Lubuskie, Poland; ^2^Department of Medical Biotechnology, Jagiellonian University, Krakow, Poland, Krakow, Malopolskie, Poland; ^3^Department of Medical Analytics, Pomeranian Medical University, Szczecin, Zachodniopomorskie, Poland; ^4^Department of Microbiology and Immune Diagnostics, Pomeranian Medical University, Szczecin, Zachodniopomorskie, Poland; ^5^Department of Gastroenterology, Pomeranian Medical University, Szczecin, Poland, Szczecin, Zachodniopomorskie, Poland

###### **Correspondence:** Wojciech Blogowski (drannab@wp.pl)

**Background**

Gastric neoplasms constitute some of the most commonly occurring and heterogenic tumors, which are associated with a high percentage of mortality among affected individuals. While the most common is gastric cancer, within the gastric tissue, several other types of malignancy may arise, including lymphomas, gastrointestinal stromal tumors, and neuroendocrine neoplasms, which offer much more favorable prognosis. Recently there has been an increased interest in the potential role of bone marrow-derived stem cells (BMSCs) in the development/progression of malignancies in humans. However, currently very little is known about BMSC homeostasis in patients with different types of gastric neoplasms. Therefore, in this study, we wanted to comprehensively examine and compare the peripheral trafficking of different populations of BMSCs in these patients, as well as to (at least preliminarily) elucidate the immune-related substances that might be associated with this phenomenon.

**Methods**

Using FACS analysis supported by ImageStream technique verification, various populations of BMSCs, such as Lin-CD45-CD133 + CXCR4+ very small embryonic/epiblast-like stem cells (VSELs), CD45-STRO-1 + CD105+ mesenchymal stem cells (MSCs), Lin-CD45 + CD133+ hematopoietic stem/progenitor cells (HSPCs), and CD34 +/ KDR +/ CD31 +/ CD45- endothelial progenitor cells (EPCs), were enumerated and sorted from the peripheral blood samples collected from patients with gastric cancer (n = 25) other gastric tumors (GISTs, NENs, lymphomas; n = 15) and control individuals (n = 20). Plasma levels of stromal-derived factor-1 (SDF-1), complement-derived anaphylatoxins/molecules (C3a, C5a, C5b-9), cytokines (IL-1, IL-6, IL-8, IL-10, IL-17, IL-23, TNFα), growth factors (HGF, FGF, IGF-1) and immunomodulatory bioactive lipids (sphingosine-1-phosphate–S1P and lipoxin A4) were measured using ELISA.

**Results**

Significantly lower numbers of circulating HSPCs and intensified peripheral trafficking of both VSELs and MSCs was observed in patients with gastric cancer but not in those with other types of gastric tumors. The abnormal balance in the peripheral trafficking of BMSCs in patients with gastric cancer was neither associated with the clinical stage of the disease nor with the systemic levels of SDF-1, which were comparable between all analyzed groups. In patients with gastric cancer, significantly increased levels of C5b-9, interleukins (IL-6, IL-8, IL-23), HGF and bioactive lipids (S1P, lipoxin A4) were strongly associated with observed phenomenon of intensified circulation of VSELs and MSCs.

**Conclusions**

Abnormal peripheral trafficking of BMSCs occurs in patients with gastric cancer and is specific only for this group of patients suffering from gastric tumors. Selected interleukins, complement cascade-derived molecules, growth factors and bioactive lipids, but not SDF-1, seem to be significant players associated with this phenomenon in humans.

**Acknowledgements**

Study financed from POIG.01.01.02-00-109/09 and IP2014003273.

### P291 Quantification of natural killer cell-mediated cytotoxicity using Celigo imaging cytometry

#### Leo Chan^1^, Srinivas Somanchi^2^, Kelsey McCulley^1^, Dean Lee^3^

##### ^1^Nexcelom Bioscience, Lawrence, MA, USA; ^2^University of Texas MD Anderson Cancer Center, Houston, TX, USA; ^3^Nexcelom Bioscience, Houston, MA, USA

###### **Correspondence:** Leo Chan (lchan@nexcelom.com)

**Background**

Cytotoxicity assays play a central role in studying the function of immune effector cells such as cytotoxic T lymphocytes (CTL) and natural killer (NK) cells. Traditionally, cytotoxicity assays have been performed using ^51^chromium (^51^Cr) and calcein release assays. The assays involve labeling tumor cells (target) with radioisotope or fluorescent dyes, when the target cells are subjected to cytolysis by CTLs or NK cells (effector), they releases the entrapped labels into the media upon lysis. The amount of labels in the media is measured to determine the level of cytotoxicity the effectors have induced. These traditional methods may generate inconsistent results due to low sensitivity caused by poor loading efficiency and high spontaneous release of the reagents.

**Methods**

In this work, we demonstrate a novel cytotoxicity assay using the Celigo imaging cytometry method. Utilizing imaging cytometry, direct cell counting of live fluorescent target cells can be performed, which is a direct method for assessment of cytotoxicity. Human NK cells from one healthy donor were used as effectors, and K562 (suspension) and IMR32 (adherent) were used as the target cells. Both target cells were first stained with calcein AM, and seeded at 10,000 cells/well in a standard 96-well microplate. The donor NK cells were then added to each well at effector-to-target (E:T) ratios 10:1, 5:1, 2.5:1, 1.25:1, 0.625:1, and 0.3125:1. The 96 well plate was then scanned and analyzed using Celigo imaging cytometer at t = 1, 2, 3, and 4 h to measure the % lysis of target cells.

**Results**

The results showed increasing % lysis as incubation time and E:T ratio increased. For the E:T ratios at 10:1, 5:1, 2.5:1, 1.25:1, 0.625:1, and 0.3125:1, the % lysis were 99, 93, 75, 52, 30, and 7 %, respectively for IMR32. The K562 cells showed similar % lysis results. In addition, the change in % lysis over time was monitored for both cell types, showing increasing % lysis from 1 to 4 hours.

**Conclusions**

The proposed Celigo imaging cytometry is an accurate and simple method for direct quantification of NK cell-mediated cytotoxicity, which can be an attractive method for both academic and clinical research. In addition, plate-based image cytometry enables high-throughput screening platform for more efficient identification of potential antibody drug candidates.

**Fig. 31 (abstract P291).**
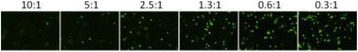
E:T Ratio dependent cytotoxicity fluorescent images. The calcein AM fluorescent images are the K562 Target cells at t = 4 hours, showing decrease in calcein AM positive Target cells as E:T ratio increased. The IMR32 images were similar

**Fig. 32 (abstract P291).**
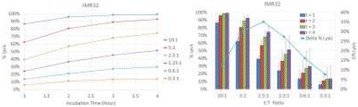
Time course and dose dependent cytotoxicity results. The time course results showed increase in % lysis from 1 to 4 hours. The dose response graphs showed increasing % lysis as E:T ratio increased

### P292 The HLA-associated phosphoproteome as a new target for immunotherapy against hepatocellular carcinoma

#### Nico Buettner^1^, Feng Shi^2^, Paisley T Myers^3^, Stuart Curbishley^4^, Sarah A Penny^4^, Lora Steadman^4^, David Millar^2^, Ellen Speers^3^, Nicola Ruth^4^, Gabriel Wong^4^, Robert Thimme^1^, David Adams^4^, Mark Cobbold^2^

##### ^1^Universitätsklinikum Freiburg, Freiburg, Germany; ^2^Massachusetts General Hospital Cancer Center, Boston, MA, USA; ^3^University of Virginia, Charlottesville, VA, USA; ^4^University of Birmingham, Birmingham, England, UK

###### **Correspondence:** Mark Cobbold (mcobbold@mgh.harvard.edu)

**Background**

Hepatocellular carcinoma (HCC) is the sixth most common cancer with a growing incidence and mortality. Since HCC is believed to be immunogenic, immunotherapy is considered a promising new treatment modality. The identification of novel and specific tumor-associated antigens provides the basis for the development of an efficient immunotherapy. However, only very few HCC-specific tumor antigens have been characterized so far and post-translational modified peptides have been completely overlooked in the past. Dysregulation of signaling pathways in cancers leads to aberrant and augmented protein phosphorylation and in such way modified proteins can be degraded to generate cancer-specific phosphopeptides. These are presented by MHC class I molecules and recognized by T cells. The aim of this study was to identify HCC-associated, MHC class I-bound phosphopeptides (MHC-I-pP) and to assess immunity against this novel class of antigens in patients with chronic liver disease and HCC.

**Methods**

For identification, tissues were lysed and MHC class-I complexes affinity purified. The bound phosphopeptides were eluted, enriched and characterized using mass spectrometry. We compared MHC class I-bound phosphopeptides (MHC-I-pP) found on healthy, cirrhotic liver tissue and HCC from patients undergoing liver transplantation. To assess immunity PBMCs, intrahepatic lymphocytes (IHLs) and tumor-infiltrating lymphocytes (TILs) were extracted from healthy donors, patients with chronic liver disease and HCC, and analyzed using multiplexed intracellular cytokine staining. Additionally, MHC-I-pP-specific CD8+ T cells from IHLs and TILs were expanded in a large scale using a rapid expansion protocol with anti-CD3, IL-2 and irradiated feeder cells.

**Results**

So far we have identified over 300 HCC-associated MHC-I-pP presented by various HLA molecules. Many of the novel identified MHC-I-pP were derived from proteins, which can be directly linked to important cancer-associated signaling-pathways. More MHC-I-pP were displayed in a greater diversity during the course of liver disease from non-cirrhotic liver to precancerous liver cirrhosis and up to HCC. CD8+ T cell responses were found regularly in patients with early chronic liver disease and were much rarer in patients with end-stage liver cirrhosis and after HCC formation.

**Conclusions**

Immune responses against these novel tumor-antigens were comparable in quantity and quality to those seen against viral control epitopes. Our results therefore suggest that MHC-I-pP may be the target of cancer immune surveillance in liver disease. Furthermore, a directed expansion of MHC-I-pP-specific CD8+ T cells was possible in a large scale for a possible application in adoptive T cell therapy. Therefore, MHC class-I bound phosphopeptides represent an attractive novel target for future cancer immunotherapies.

### P293 Cancer testis antigens, immunotherapeutic target antigens in triple negative breast cancer

#### Remy Thomas^1^, Wouter Hendrickx^2^, Mariam Al-Muftah^1^, Julie Decock^1^

##### ^1^Qatar Biomedical Research Instititute (QBRI), Doha, Ar Rayyan, Qatar ^2^Sidra Medical and Research Center, Doha, Ad Dawhah, Qatar

###### **Correspondence:** Julie Decock (jdecock@qf.org.qa)

**Background**

Cancer testis antigens (CTA) represent one type of tumor antigen that can elicit strong immune responses thanks to their restricted expression in normal germ cells and tumor cells. Upregulated expression has been observed in various cancer types including melanoma, bladder, lung, ovarian, and hepatocellular carcinomas. NY-ESO-1 has emerged as a promising target as it is highly immunogenic and associated with poor clinical outcome [1, 2]. Reports on CTA expression in breast cancer are inconclusive and sparse data is available on triple negative breast cancer (TNBC).

**Methods**

Using a bioinformatics approach, we investigated the expression of CTAs that are transcriptionally silent in normal non-germline tissues [3] in both human TNBC cell lines and specimens. Gene and protein analyses were performed *in vitro* to evaluate our *in silico* findings, and functional assays were conducted for 3 CTAs.

**Results**

We mined the TCGA and NCBI GEO repositories for CTA expression in TNBC and found a consensus moderate expression of DKKL1, LDHC, MAGE-A3, PIWIL2, PLAC1, PRAME, PRSS50 and TSGA10 in cell lines and tumor specimens. Interestingly, NY-ESO-1 expression was not found to be upregulated in triple negative breast cancer. Expression profiling of 9 human TNBC cell lines, encompassing all 6 TNBC subtypes, confirmed LDHC, MAGE-A3 and PRAME to be good candidate targets. Their cellular localization, determined by immunocytochemistry, indicates that redirected, rather than chimeric antigen receptor (CAR)-modified T cells, should be developed as tools for adoptive T cell therapy. We are currently identifying the immunogenic peptides to be used for T cell engineering. Furthermore, we are conducting functional assays to determine the role of LDHC, PRAME and MAGE-A3 in TNBC development and progression, in particular in cell metabolism, proliferation, apoptosis, migration and invasion.

**Conclusions**

We found a number of cancer testis antigens to be expressed at moderate to high levels in triple negative breast cancer. Our preliminary findings suggest that LHDC, MAGE-A3 and PRAME could be good targets for adoptive T cell therapy of TNBC.

**References**

1. Gnjatic S, Nishikawa H, Jungbluth AA *et al.*: **NY-ESO-1: review of an immunogenic tumor antigen.** *Adv Cancer Res* 2006, **95**:1–30.

2. Svobodová S, Browning J, MacGregor D, *et al.*: **Cancer-testis antigen expression in primary cutaneous melanoma has independent prognostic value comparable to that of Breslow thickness, ulceration and mitotic rate.** *Eur J Cancer* 2011, **47(3)**:460–469.

3. Rooney MS, Shukla SA, Wu CJ, *et al.*: **Molecular and genetic properties of tumors associated with local immune cytolytic activity.** *Cell* 2015, **160(1–2)**:48–61.

### P294 Overall survival and outcomes of patients treated with high dose IL-2 from the PROCLAIM registry

#### Michael KK Wong^1^, Michael Morse^2^, David F McDermott^3^, Joseph I Clark^4^, Howard L Kaufman^5^, Gregory A Daniels^6^, Hong Hua^7^, Tharak Rao^7^, Janice P Dutcher^8^

##### ^1^University of Texas MD Anderson Cancer Center, Houston, TX, USA; ^2^Duke University Medical Center, Durham, NC, USA; ^3^Beth Israel Deaconess Medical Center, Boston, MA, USA; ^4^Loyola University Medical Center, Maywood, IL, USA; ^5^Rutgers Cancer Institute of New Jersey, New Brunswick, NJ, USA; ^6^UC San Diego Moores Cancer Center, La Jolla, CA, USA; ^7^Prometheus Laboratories Inc., San Diego, CA, USA; ^8^Cancer Research Foundation, Chappaqua, NY, USA

###### **Correspondence:** Hong Hua (hong.hua@prometheuslabs.com)

**Background**

High dose IL-2 (HD IL-2) can provide durable responses in patients with metastatic melanoma (mM) and metastatic renal cell carcinoma (mRCC). PROCLAIM^SM^ [http://www.proclaimregistry.com] is an IL-2 registry that captures real-world patient population survival and outcomes. Herein, we report on patient experience sequencing HD IL-2 with immune checkpoint blockade (ICB) and/or targeted therapy (TT) in mM and mRCC.

**Methods**

Patients were prospectively enrolled into the registry as of 2011 and must have received at least one dose of HD IL-2. Statistics and survival analysis were performed on datasets as of December 17, 2015.

**Results**

The mOS for mM patients (n = 337) was 19.4 months, with a median follow-up (mFU) of 22 months. The overall response rate (ORR) for 323 patients with available data was 13.9 %. The mOS for those with complete response (CR, n = 10) and partial response (PR, n = 35) was not reached (NR). The mOS for patients experiencing stable disease (SD, n = 92) and progressive disease (PD, n = 186) was 29.3 and 12.2 months, respectively. The mOS for all mRCC patients (n = 412) was NR, with a mFU of 21 months. The ORR for the 382 patients with available data was 17.8 %. For patients with CR (n = 14), PR (n = 53), or SD (n = 146) mOS was NR while in patients with PD (n = 169), mOS was 17 months. In mM, two groups were further analyzed; HD IL-2 followed by ipilimumab alone (IL-2 then IPI), and HD IL-2 followed by PD-1/PD-L1 inhibitors with or without ipilimumab (IL-2 then aPD-1 ± IPI, Table 2). In mRCC, survival based on TT or ICB after HD IL-2 was analyzed (Table 3). Survival of patients receiving HD IL-2 as first line with no post therapy was also determined (Table 3).

**Conclusions**

This analysis of the national IL-2 registry suggests that HD IL-2 continues to be a valuable treatment option for eligible mM and mRCC patients. Attainment of SD is clinically relevant and contributes to HD IL-2’s survival benefit. Support for investigation of HD IL-2 in combination or sequence with ICB is warranted.

**Trial Registration**

ClinicalTrials.gov identifier NCT01415167.

**Table 2 (abstract P294).** See text for description

	Metastatic Melanoma	
Groups based on therapies after IL-2	IL-2 then IPI	IL-2 then aPD-1±IPI
N	81	56
mOS (months)	15.8	28.7
1-,2-,3-year survival (%)	64, 28, 18	96, 66, 35

**Table 3 (abstract P294).** See text for description

	mRCC				
Groups based on therapies after IL-2	TT	TT & ICB	ICB	No TT or ICB	IL-2 first line (no prior or post therapy
n	190	14	12	196	149
mOS (months)	35.5	NR	NR	NR	NR
1-, 2-, 3-year survival (%)	81, 63, 49	100, 81, 81	90, 79, 79	76, 65, 65	82, 72, 72

### P295 **Tetrahydrouridine and decitabine combination for p53-independent cytoreduction and immune-priming of NSCLC in vivo**

#### Kai Kang^1^, Yogen Saunthararajah^2^, Vamsidhar Velcheti^2^

##### ^1^Taussig Cancer Institute, Cleveland Clinic, Cleveland, OH, USA; ^2^Cleveland Clinic, Cleveland, OH, USA

###### **Correspondence:** Kai Kang (kangk@ccf.org)

**Background**

Even if a cancer is immune recognized, it may evade immune attack using immune checkpoint. Such immune-recognized cancers are vulnerable to immune checkpoint inhibitors (e.g., anti-PD-1). Unfortunately, the response rate of non-small cell lung cancer to nivolumab is only ~20 %. One important approach to improve immune checkpoint blockade is to increase the rate at which NSCLC is immune-recognized in the first place. A major way in which cancers escape immune recognition is by epigenetic silencing of neo-antigens. A central epigenetic protein mediating such silencing is DNA methyl-transferase 1 (DNMT1), and DNMT1 has been validated as a molecular target to increase NSCLC immune-recognition. Moreover, DNMT1-depletion cytoreduces cancers directly by increasing expression of epithelial-differentiation genes that directly antagonize MYC, to produce p53-independent cell cycle exits that spare immune-effectors. Decitabine (Dec) depletes DNMT1 and can potentially translate this science; however, Dec has a very brief plasma t_1/2_ *in vivo*. Therefore, we combined an inhibitor of CDA, tetrahydrouridine (THU), with reduced doses of Dec, with the objective that depletes DNMT1 while avoiding a high Cmax that causes off-target cytotoxicity.

**Methods**

C57/BL6 mice were inoculated with LL3 cells by tail vein. Mice (n = 15) randomized into 3 groups were treated with PBS, Dec (0.2 mg/kg s.c. 3X/wk), or THU-Dec (10/0.1 mg/kg s.c. 3X/wk). Peripheral blood was collected to detect MDSC and T lymphocytes. Survival time was recorded. At sacrifice, tumor samples were examined by flow cytometry, H&E staining, Western blot and QRT-PCR.

**Results**

**Fig. 33 (abstract P295).**
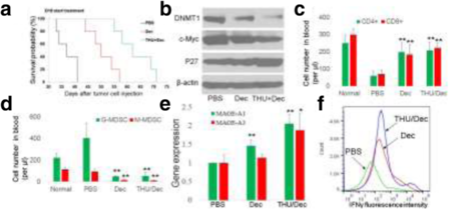
THU-Dec directly cytoreduced lung cancer in vivo by p53-independent mechanisms while preserving immune-effectors and increasing antigen presentation. C57/BL6 mice were inoculated via tail vein with LL3 lung cancer cells. **a** Survival time; **b** Protein expression in tumor; **c** Absolute numbers of CD4/8 T cells in peripheral blood after 2 weeks of treatment; **d** Absolute numbers of MDSC in blood after 2 weeks of treatment; **e** Cancer testis antigen expression in tumor; **f** Intracellular IFNγ expression in tumor. Data were expression as mean ±SD. *p < 0.05, **p < 0.01 vs PBS group

Survival time significantly increased with THU-Dec (62) > Dec (50) > vehicle (37) (median survival days) (Fig. 33a). Tumor DNMT1 and MYC protein decreased to a substantially greater extent with THU-Dec than with Dec alone, concurrent with substantial increase in p27 (Fig. 33b). THU-Dec preserved peripheral blood counts including absolute lymphocyte counts (Fig. 33c), while substantially decreasing the numbers of MDSC (Fig. 33d). THU-Dec also significantly increased expression of immunotherapy antigenic targets MAGE-A1 and MAGE-A3 to a greater extent than Dec (p < 0.01) (Fig. 33e). THU-Dec treatment also decreased regulatory T cells in blood and tumor, and increased the release of INFγ and expression of PD-L1 in tumor cells, compared to Dec alone or PBS (Fig. 33f).

**Conclusions**

Addition of THU to Dec, to address pharmacologic limitations of Dec alone, improved immune-priming and induced p53-independent cell cycle exits in NSCLC *in vivo*. There is scientific and pharmacologic rationale to combine THU-Dec with nivolumab in the clinical trial NCT02664181.

### P296 Umbelliferon-α-D-glucopyranosyl-(2I → 1II)-α-D-glucopyranoside: a potential candidate affords chemoprevention against chemically-induced carcinogenesis through attenuating nuclear factor-κB

#### Vikas Kumar^1^, Firoz Anwar^2^, Amita Verma^1^

##### ^1^Sam Higginbotham Institute of Agriculture, Technology & Sciences, Allahabad, Uttar Pradesh, India; ^2^King Abdulaziz University, Jeddah, Afghanistan

###### **Correspondence:** Vikas Kumar (phvikas@gmail.com)

**Background**

The liver is the principal metabolizing site of the body, which is commonly prone to damage through various toxicants. Diethylnitrosamine (DEN) is a potent hepatocarcinogen and hepatotoxin, which is well known for modulating the inflammatory cytokines and oxidative stress in the liver. The plants are the rich source of umbelliferone, which are the secondary metabolites widely distributed in various species of fruits, vegetables, tea, spices and widely consumed by humans worldwide. The aim of the current investigation was to exemplify the effect of umbelliferon-α-D-glucopyranosyl-(2I → 1II)-α-D-glucopyranoside (UFD) on tissue protection, chronic hepatic inflammation and oxidative stress induced by DEN.

**Methods**

The rats were subjected to hepatic carcinogenesis by treating with a single intraperitoneally injection of DEN (200 mg/kg). The rats were euthanized at the end of the experiment and livers were microscopically scrutinizing. Biomarker of oxidative stress (lipid peroxides, reduced glutathione and conjugated dienes), liver enzyme parameters and nonenzyme liver parameters were also examined. The liver tissue was also invested for the existence of proinflammatory cytokines, including IL-1β, IL-6 and TNF-α.

**Results**

The results clearly demonstrated the UFD dose-dependently inhibited those as mentioned earlier elevated oxidative stress parameters, liver and non-liver parameters dose dependently. Our result revealed the essential repression of the inflammation cascade through modulation of nuclear factor-kappa B (NF-κB) signaling pathway may characterize a novel mechanism of action hepatic tumor inhibitory effect of UFD against experimental toxicant-induced hepatocarcinogenesis.

**Conclusions**

It is evident from the present study that UFD inhibits the DEN-initiated hepatocarcinogenesis in rats through proinflammatory cytokines via the anti-inflammatory mechanism.

**Acknowledgements**

The authors acknowledge the Department of Pharmaceutical Sciences, Faculty of Health Sciences, SHIATS.

**References**

1. Kumar V, Khan R, Kazmi I, Afzal M, Al-Abbasi FA, Anwar F: **Fixed dose combination therapy loperamide and niacin ameliorates diethylnitrosamine-induced liver carcinogenesis in albino wistar rats**. *RSC Advances* 2015, **5**:67996–68002.

2. Kumar V, Anwar F, Sethi N, Al-Abbasi FA: **Umbelliferone β-D-galactopyranoside inhibits chemically induced renal carcinogenesis via alteration of oxidative stress, hyperproliferation and inflammation: possible role of NF-kB**. *Toxicol Res* 2015.

### P297 Novel and shared neoantigen derived from histone 3 variant H3.3 K27M mutation – characterization of the epitope and cloning of a specific T cell receptor for glioma T cell therapy

#### Zinal Chheda^1^, Gary Kohanbash^1^, John Sidney^2^, Kaori Okada^1^, Shruti Shrivastav^1^, Diego A Carrera^1^, Shuming Liu^1^, Naznin Jahan^1^, Sabine Mueller^1^, Ian F Pollack^3^, Angel M Carcaboso^4^, Alessandro Sette^2^, Yafei Hou^1^, Hideho Okada^1^

##### ^1^University of California, San Francisco, San Francisco, CA, USA; ^2^La Jolla Institute for Allergy and Immunology, La Jolla, CA, USA; ^3^University of Pittsburgh, Pittsburgh, PA, USA; ^4^Hospital Sant Joan de Déu Barcelona, Barcelona, Spain

###### **Correspondence:** Hideho Okada (hideho.okada@ucsf.edu)

**Background**

Brain tumors are the leading cause of cancer-related mortality and morbidity in children. Recent genetic studies have revealed that malignant gliomas in children and young adults often show shared missense mutations in the gene encoding the replication-independent histone 3 variant H3.3 [1]. Approximately 70 % of midline gliomas, such as DIPG, harbor the amino-acid substitution from lysine (K) to methionine (M) at the position 27 of H3.3 (the H3.3 K27M mutation hereafter). The K27M mutation in DIPG is universally associated with shorter survival compared with patients with non-mutated H3.3.

**Methods**

We evaluated whether the H3.3 K27M mutation can induce specific cytotoxic T lymphocyte (CTL) responses in human leukocyte antigen (HLA)-A2+ CD8+ T cells as a neoantigen epitope. Competitive binding inhibition assays were performed for evaluation of affinity of synthetic peptides to HLA-A2. α- and β-chain cDNAs from a high-affinity T cell receptor (TCR) were cloned by amplification of cDNA Ends–PCR (RACE-PCR).

**Results**

*In vitro* stimulation of HLA-A2+ donor-derived CD8+ T cells with a synthetic peptide encompassing the H3.3 K27M mutation (the H3.3.K27M epitope hereafter) induced CTL lines which recognized not only the synthetic H3.3.K27M epitope peptide loaded on T2 cells but also lysed HLA-A2+ DIPG cell lines which endogenously harbor the H3.3.K27M mutation. On the other hand, CTL lines did not react to HLA-A2+, H3.3 K27M mutation-negative cells or HLA-A2-negative, H3.3 K27M mutation + cells. The H3.3.K27M epitope peptide, but not the non-mutant counterpart, indicated an excellent affinity (Kd 151 nM) to HLA-A2. Furthermore, CTL clones with high and specific affinities to HLA-A2-H3.3.K27M-tetramer were successfully obtained, and both retroviral and lentiviral vectors have been created to encode α- and β-chain cDNAs from a high-affinity TCR. Human T cells transduced with the lentiviral or retroviral TCR demonstrated antigen-specific reactivity. Furthermore, critically important for safety of clinical application, alanine scanning demonstrated that the key amino-acid sequence motif in the epitope for the TCR reactivity is not shared by any known human protein.

**Conclusions**

These data provide us with a strong basis for developing peptide-based vaccines as well as adoptive transfer therapy using autologous T cells transduced with the TCR.

**Acknowledgements**

This study is supported by the NIH/NINDS (1R01NS096954), V Foundation and Parker Institution for Cancer Immunotherapy.

**References**

1. Schwartzentruber J, Korshunov A, Liu XY, Jones DT, Pfaff E, Jacob K, *et al.*: **Driver mutations in histone H3.3 and chromatin remodelling genes in paediatric glioblastoma.** *Nature* 2012, **482**:226–31.

**Fig. 34 (abstract P297).**
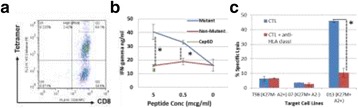
HLA-A2.1+ donor-derived CTLs specifically recognize HLA-A2.1+ K27M+ glioma cells in an HLA-class I-dependent manner. Peripheral blood mononuclear cells from an HLA-A2.1+ donor were stimulated *in vitro* with the H3.3.K27M peptide and evaluated for their reactivity against: **a** HLA-A2.1/H3.3.K27M-specific tetramer and anti-CD8 mAb, and (**b**) T2 cells pulsed with the mutant or non-mutated H3.3 peptide by IFN-γ ELISA. In (**a**), among the CD8+tetramer+ population (64.1% of total lymphocyte-gated cells), there is a tetramer^high^ subpopulation (2.4% of total lymphocyte-gated cells), some of which were used as CTL clones (Aim 2). In (**b**), the Cap1-6D peptide (tested at 5mcg/ml only) is a high avidity HLA-A2.1-binding epitope derived from CEA1 used as an irrelevant negative control. **c** The CTL line was evaluated for cytotoxicity against glioma cell lines T98 (HLA-A2.1+but K27M-negative), HSJD-DIPG-07 (HLA-A2.1-negative but K27M+), and HSJD-DIPG-13 (HLA-A2.1+ and K27M+) lines. CFSE-labeled target cells (10e4/well) were incubated with CTLs at the E/T ratio of 25 for 4 hours. To block the CTL cytotoxicity, anti-HLA-ABC 10μg/ml was added to one group. At the end of incubation, 7-AAD was added into each well and incubated for 10 minutes on ice. The samples were analyzed by flow cytometry, and the killed target cells were identified as CFSE+ and 7-AAD+ cells. The cytotoxicity was calculated as the percentage of CFSE+ and 7-ADD+ cells in total HLA-A2+ CFSE+ cells (*p<0.05 by Wilcoxon rank-sum tests)

**Fig. 35 (abstract P297).**
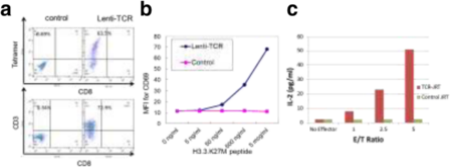
Evaluation of H3.3.K27M-specific TCR. **a** J.RT-T3.5 cells were transduced with lentiviral vector encoding the TCR α- or β-chains derived from H3.3.K27M-specific CTL clone IH5 (J.RT-T3.5-TCR). The J.RT-T3.5-TCR or control non-transduced J.RT-T3.5 cells were evaluated for the surface TCR expression using PE-labeled HLA-A*0201/H3.3.K27M tetramer (upper panel) or PE-labeled anti-CD3 mAb (lower panel) and FITC-labeled anti-human CD8 mAb (upper and lower panels). Since J.RT3-T3.5 cells are CD4+ and CD8-negative, tetramer+ CD8-negative cells are ones expressing the transgene-derived TCR. CD3-upregulation indicates activation of cells. **b** J.RT-T3.5-TCR, but not control J.RT-T3.5 cells, upregulate CD69 expression upon recognition of the H3.3 K27M peptide loaded on T2 cells. **c** DIPG 13 cells [HLA-A*0201+ (albeit dim), K27M mutation+] were incubated with J.RT-T3.5-TCR or control J.RT-T3.5 cells. IL-2 secretion in the culture media was assayed by specific ELISA

**Fig. 36 (abstract P297).**
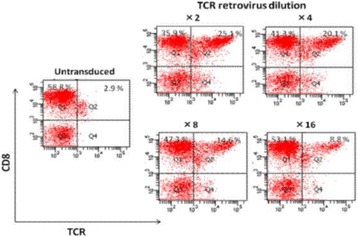
Efficient expression of transgene TCR on human PBMC-derived T cells. Human PBMC-derived T cells were transduced with serially diluted preparation of the retroviral vector encoding H3.3.K27M-specific TCR and evaluated by HLA-A2-H3.3.K27M-specific tetramer and anti-CD8 monoclonal antibody

**Fig. 37 (abstract P297).**
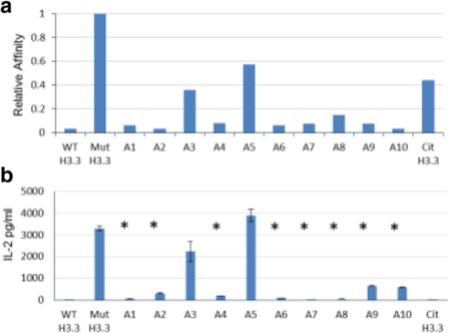
Alanine scanning to determine the key immunogenic AA residues of the H3.3.K27M epitope. **a** Relative HLA-A*0201-binding affinity of each peptide to that of H3.3.K27M (26–35) was determined by cell-free binding assay. **b** J.RT3-T3.5 cells were transduced with lentiviral vector encoding the H3.3.K27M-specific TCR and evaluated for the recognition of each peptide loaded on T2 cells by production of IL-2. Each group was assayed as triplicate *<0.05 by Student-t compared with the mutant H.3.3. In addition to 10 synthetic peptides each containing the substitution with alanine (A1-A10), we also evaluated synthetic peptides designed for citrullinated H3.3. K27M epitope (Cit H3.3; i.e., the first AA of the H3.3.K27M epitope is replaced by citrulline)

### P298 The fucosylation inhibitor 2-fluorofucose exhibits anti-tumor activity and modulates immune cell activity both in vitro and in vivo

#### Jessica J Field, Weiping Zeng, Vincent FS Shih, Che-Leung Law, Peter D Senter, Shyra J Gardai, Nicole M Okeley

##### Seattle Genetics, Inc., Bothell, WA, USA

###### **Correspondence:** Nicole M Okeley (nokeley@seagen.com)

**Background**

2-Fluorofucose (2FF) is a small molecule inhibitor of glycoprotein fucosylation. 2FF-mediated afucosylation of cancer cells results in reduced adhesive properties. Furthermore, T cells treated with 2FF during expansion show increased T cell receptor (TCR) signaling, enhanced maturation of autologous dendritic cells (DCs), and reduced regulatory T cell numbers, demonstrating modulation of immune activity [1]. 2FF has shown promising *in vivo* anti-tumor activity, both alone and in combination with a cancer vaccine, which appears to be dependent on immune cell activity. Specifically, 2FF elicits growth delay in the generally refractory 4 T1 murine breast cancer model, whereas this effect is lost when the 4 T1 model is run in immunodeficient NSG mice.

**Methods**

To further understand the immune modulatory activity of 2FF, tumor infiltrating immune cells in 4 T1 tumors were counted and profiled using multi-color flow cytometry. Additionally, antigen recall function of CMV reactive T cells expanded with 2FF was assessed by culturing T cells and autologous DCs with CMV peptides and monitoring IFNγ and IL-12p70 production. We also used this model to investigate the combinatorial activity of 2FF treated T cells with anti-PD-1 antibodies.

**Results**

2FF treatment in the 4 T1 murine model reduced the number of regulatory T cells and increased activated DCs. These DCs showed a mild increase in the maturation markers CD83, CD86, and MHCII. The effects observed *in vivo* recapitulate our *in vitro* observations with human T cells and T cell/DC co-cultures, and demonstrate the translation of 2FF immune modulatory activity from *in vitro* to *in vivo*. Since 2FF reduces the regulatory T cell population, an effect seen with anti-CTLA-4 antibodies, we postulated that PD-1 checkpoint blockade antibodies would enhance 2FF-treated T cell function. T cells treated with 2FF during expansion showed increased CMV antigen specific response, while co-cultures that contained both 2FF-treated T cells and anti-PD-1 antibody showed increased levels of production of IFNγ and IL-12p70 above those seen with either single agent alone. These data suggest that co-treatment with 2FF and anti-PD-1 antibody can lead to increased antigen specific T cell activation *in vitro*.

**Conclusions**

Overall, we conclude that 2FF may promote a more active tumor immune microenvironment, providing a better opportunity for an effective host anti-tumor immune response alone or in combination with checkpoint blockade. A first-in-human phase I clinical trial evaluating 2FF in advanced solid tumors is planned.

**References**

1. Field JJ, *et al.*: **Understanding the mechanism of 2FF-induced immune modulation [abstract]**. *Proc Am Assoc Cancer Res* 2016, **76(14 Suppl)**: Abstract 4005.

### P299 Phosphopeptides as novel tumor antigens in colorectal cancer

#### Sarah A Penny^1^, Jennifer G Abelin^2^, Abu Z Saeed^1^, Stacy A Malaker^3^, Paisley T Myers^2^, Jeffrey Shabanowitz^2^, Stephen T Ward^1^, Donald F Hunt^2^, Mark Cobbold^4^

##### ^1^University of Birmingham, Birmingham, England, UK; ^2^University of Virginia, Charlottesville, VA, USA; ^3^Stanford University, Stanford, CA, USA; ^4^Massachusetts General Hospital Cancer Center, Boston, MA, USA

###### **Correspondence:** Sarah A Penny (s.a.penny@bham.ac.uk)

**Background**

There is a pressing need for novel immunotherapeutic targets in colorectal cancer (CRC). Memory CD8+ T cell infiltration is now well established as a key prognostic indicator in CRC, and it is known that these tumor infiltrating lymphocytes (TILs) are specifically targeting and killing tumor cells. However, the antigens that these TILs target have not previously been determined. This has limited the use of immunotherapies in CRC, despite their efficacy in other cancer types. Recently, phosphopeptides have emerged as strong candidates for tumor-specific antigens, since dysregulation of signaling in cancers leads to aberrant protein phosphorylation. Here, we identify CRC-associated phosphopeptides and assess the tumor-resident immunity against these novel posttranslationally modified tumor antigens.

**Methods**

We compared tumor and healthy tissue from CRC patients, to identify tumor-specific MHC class I associated phosphopeptides. Phosphopeptides were enriched using immobilized metal affinity chromatography, and characterized using mass spectrometry. TILs, from the same tumors, were extracted and expanded, and their responses to the phosphopeptides assessed using multiplexed intracellular cytokine staining. Cytolytic activity was observed by staining for surface mobilization of CD107a. Healthy donor responses were quantified using interferon-γ ELISpot and functionality assessed using a europium release killing assay.

**Results**

We have identified 133 tumor-associated MHC class I phosphopeptides from CRC, with different HLA-restrictions. There were, on average, 3.1 times more different phosphopeptides identified on primary cancers than healthy tissues, at 6.9-fold higher levels. 35 % of these can be attributed to signaling events in well-defined cancer pathways and are therefore markers of malignancy. Through analysis of TIL’s cytokine responses to these phosphopeptides, we have established that they are playing a key role in tumor-resident immunity. There were multifunctional TILs present in primary and metastatic tumors that recognized and killed in response to these phosphopeptides. Up to 0.7 % of expanded TILs targeted each phosphopeptide, comparable with responses seen to viral epitopes. Immunity to these tumor-associated phosphopeptides represents a biological strategy for distinguishing tumor from healthy tissue. Furthermore, we have shown that healthy donors have pre-existing, memory T cell responses to many (58 %) of these CRC-associated phosphopeptides. These phosphopeptide-specific T cells are readily expanded *ex vivo* and kill CRC cell lines. Thus, MHC class I associated phosphopeptides are ideal immunotherapeutic targets, as immunity must spare healthy tissue.

**Conclusions**

The identification of this novel class of MHC class I antigens in CRC offers new hope for the future of immunotherapy in this malignancy.

### P300 Rapid and durable complete response to atezolizumab (anti-PD-L1), with sloughing of tumor tissue through urethra, in metastatic chemotherapy-resistant urothelial cancer (UC)

#### Pam Profusek, Laura Wood, Dale Shepard, Petros Grivas

##### Cleveland Clinic, Cleveland, OH, USA

###### **Correspondence:** Pam Profusek (profusp@ccf.org)

**Background**

Metastatic UC is usually fatal causing significant morbidity. Atezolizumab, an anti-PD-L1 checkpoint inhibitor, is FDA-approved for locally advanced or metastatic UC after progression on/after platinum-containing chemotherapy based on results of the IMvigor 210 trial.

**Methods**

We present a trial patient with unique sloughing of tumor tissue, outstanding treatment response and tolerance.

**Results**

A 76 year old man was diagnosed with high grade muscle-invasive UC of the bladder in April 2013. Patient completed 3 cycles of ‘neoadjuvant’ gemcitabine/cisplatin but had progression, he then received 6 cycles of docetaxel & investigational agent with progression, followed by 2 cycles of a targeted therapy on a phase I trial also with progression. Patient was then screened for the cohort 2 of the IMVigor210 trial evaluating the use of atezolizumab, 1200 mg every 21 days, in platinum-pretreated advanced UC. Screening CT showed the bladder lumen replaced by multiple masses and a left peri-aortic node 1.1 cm without other clear evidence of metastasis. Patient was experiencing fatigue, and hourly urgent urination with detrimental effect on his sleep, and overall quality of life (ECOG PS 1, normal GFR, Hgb 10.2 on Cycle 1 Day 1(C1D1). Tumor tissue analysis showed high PD-L1 expression in both tumor-infiltrating immune cells (IC2) and tumor cells (TC2), TCGA subtype IV (Basal), and a high mutational load of 18.02 Mut/MB (compared to IMvigor210, Cohort 2 median of 8.11 Mut/MB). At C2D1 visit, he already had major clinical benefit with symptomatic improvement and sloughing of tumor tissue that passed through the urethra; the latter underwent pathology review and showed necrotic debris with highly atypical cells suspicious for carcinoma. Ten days later he presented to the ED due to difficulty voiding; cystoscopy was performed to remove necrotic tissue passing through urethra. Scans at C3D1 demonstrated great response with 83 % tumor reduction, he continued to have very good quality of life. Scans at C10D1 showed that the bladder mass was no longer measurable and the lymph node was stable. Patient continues on trial without notable adverse events (has asymptomatic atrial fibrillation that was deemed possibly related to therapy) and has received 35 cycles. Patient remains very active, riding his bike most days of the week, denies urinary issues, has ECOG PS 0, with durable complete response on scans.

**Conclusions**

Atezolizumab can produce rapid and durable responses in advanced platinum-resistant urothelial cancer and represents a new treatment option. Several clinical trials are ongoing.

**Trial Registration**

ClinicalTrials.gov identifier NCT02108652.

**Fig. 38 (abstract P300).**
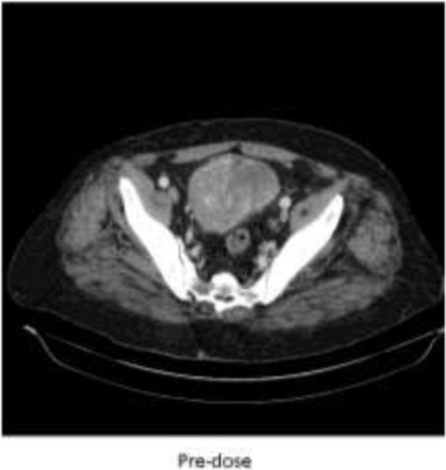
Bladder CT - Baseline

**Fig. 39 (abstract P300).**
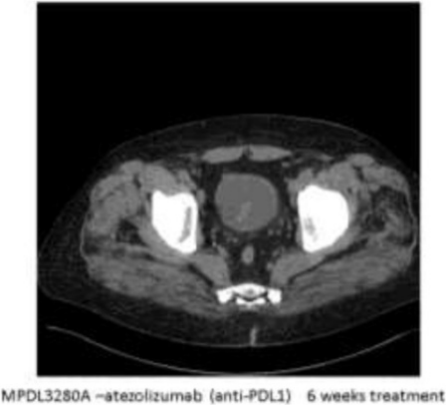
Bladder CT - 9 Weeks of Therapy. 83% Reduction

**Fig. 40 (abstract P300).**
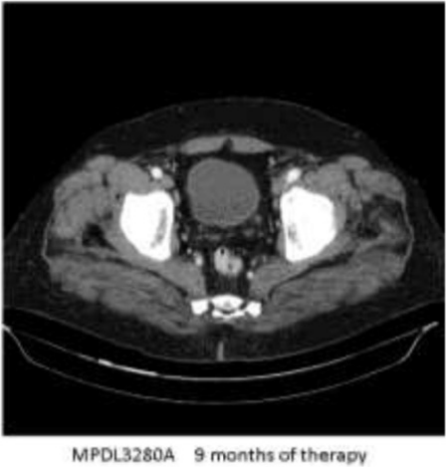
Bladder CT - 9 Months of Therapy. Complete Response

### P301 Preclinical immunological and anti-tumor data of the new enantiomeric TLR9 agonist EnanDIM

#### Kerstin Kapp^1^, Barbara Volz^1^, Detlef Oswald^1^, Burghardt Wittig^2^, Manuel Schmidt^1^

##### ^1^Mologen AG, Berlin, Germany; ^2^Foundation Institute Molecular Biology and Bioinformatics, Freie Universitaet Berlin, Berlin, Germany

###### **Correspondence:** Manuel Schmidt (maschmidt@mologen.com)

**Background**

TLR9 agonists have shown anti-tumor effects, modulating the immune system via cellular and humoral responses. Two different families of DNA molecules containing non-methylated CG-motifs for TLR9 activation have been established so far: i) Dumbbell-shaped dSLIM® molecules protected against exonucleolytic degradation by their covalently-closed, natural phosphodiester (PO) backbone and ii) single-stranded, oligodeoxynucleotides (CpG-ODN) in most cases chemically-stabilized by phosphorothioates (PTO) in their phosphate moieties. However, PTO modifications produce off-target effects in immune cell populations and result in unfavorable risk-to-benefit ratios.

**Methods**

A novel family of TLR9 agonists avoids the off-target effects of PTO-modified CpG-ODN: linear single-stranded ODN synthesized using L-deoxyribonucleotides (natural enantiomers of D-deoxyribonucleotides) at their 3’-ends - EnanDIM®. The vast majority of deoxyribose in organisms consists of D-deoxyribose, thus co-evolved nucleases are blind for L-deoxyribose - thereby leaving L-protected ODN intact. We selected nucleotide sequences of EnanDIM® using high secretion of IFN-alpha and IP-10 from human peripheral blood mononuclear cells as marker. We employed a maximum feasible dose (MFD) approach: Mice received subcutaneous injection of single doses of 10 to 50 mg EnanDIM® to evaluate their acute toxicity and immunomodulatory properties. A pilot study was used to investigate the anti-tumor effect of EnanDIM® in a CT26 tumor model.

**Results**

EnanDIM581 and EnanDIM532 were selected due to their pronounced activation of immune cells (e.g. monocytes, NK cells and pDC) and their prominent induction of IFN-alpha and IP-10 secretion *in vitro*. EnanDIM744, an EnanDIM581 variant with additional 5’-end L-nucleotide protection, was also used for MFD studies. Safety assessments throughout the study revealed no signs of toxicity despite the extremely high doses (300 to 1700 mg/kg). A gross necropsy consisting of a macroscopic organ evaluation at day 15 also revealed no abnormalities. Dose-dependent increase of IP-10 levels in serum was observed between 6 and 24 hours after injection but none after 15 days, confirming that L-nucleotides in EnanDIM® do not alter the kinetic profile known from other TLR9 agonists. First data from the CT26 tumor model showed that EnanDIM532 reduces tumor growth and prolongs survival of mice.

**Conclusions**

EnanDIM®, a new family of TLR9 agonists, broadly activates the immune system. Even maximal feasible doses of EnanDIM® resulted in no signs of toxicity, whereas a reduction of tumor growth was observed in a murine CT26 tumor model. Therefore EnanDIM® compounds have the potential for clinical development as immune surveillance reactivators in the treatment of cancer.

### P302 Loading of recycling MHC class I molecules with antibody-delivered viral peptides leads to efficient CD8+ T cell-mediated tumor cell killing

#### Julian P Sefrin, Lars Hillringhaus, Valeria Lifke, Alexander Lifke

##### Roche Diagnostics GmbH, Penzberg, Bayern, Germany

###### **Correspondence:** Julian P Sefrin (julian.sefrin@roche.com)

**Background**

In the past, antigen-armed antibodies have been used in cancer immunotherapy. Recently, Yu et al.[1] efficiently delivered Epstein-Barr virus (EBV) antigens to lymphoma cells by targeting B cell surface receptors. However, they only obtained CD4^+^ T cell activation, as externally introduced proteins enter the MHC class II antigen processing pathway. Here, we generated antibody-targeted pathogen-derived peptides (ATPPs), which deliver and release mature, virus-derived MHC class I peptides in an endosomal compartment where MHC is loaded with peptide, thereby triggering CD8^+^ T cell activation and tumor cell killing.

**Methods**

We generated antigen-armed antibodies called ATPPs, by coupling virus-derived MHC class I peptides to tumor-associated antigen-specific antibodies. Fluorescence resonance energy transfer (FRET) was performed to demonstrate the supposed mode of action. T cell activation and tumor cell killing was assessed by quantification of interferon-gamma or lactate dehydrogenase (LDH) release. Human PBMCs or expanded peptide-specific T cells were used as effector cells for *in vitro* functionality assays and *in vivo* efficacy in MDA-MB231 breast cancer subcutaneous xenograft model.

**Results**

FRET Imaging revealed that after ATPP binding to the antigen and subsequent internalization, the peptides are released in an early endosomal compartment and loaded onto recycling MHC class I complexes. MHC-peptide complexes are subsequently presented on the tumor cell surface and mediate activation of peptide-specific CD8^+^ T cells. Treatment of various tumor types resulted in efficient activation of peptide-specific CD8^+^ memory T cells and subsequent lysis of target cells *in vitro*. Similar results were obtained when targeting different tumor antigens or using various peptides with differing HLA-restrictions. Intriguingly, a 7200-fold higher amount of free peptide versus ATPP was required for comparable T cell activation. Using an elongated peptide that would require antigen processing for MHC class I binding revealed that the MHC class I antigen processing machinery is not involved. Importantly, PBMCs, where only 0.5 % of CD8^+^ T cells were antigen specific, mediated significant tumor cell lysis at an E:T cell ratio of 1:10. ATPP activated peptide specific CD8^+^ T cells induced tumor growth inhibition *in vivo.*

**Conclusions**

Our results demonstrate potent ATPP-mediated anti-tumor efficacy, independently of the MHC class I antigen processing machinery, by loading tumor cells with viral peptide antigens and redirecting virus-specific cytotoxic T cells against cancer.

**References**

1. Yu X, *et al.*: **Antigen-armed antibodies targeting B lymphoma cells effectively activate antigen-specific CD4+ T cells**. Blood 2015, **125**:1601–1610.

### P303 Treatment of tumor cells with mirvetuximab soravtansine, a FRalpha-targeting antibody-drug conjugate (ADC), activates monocytes through Fc-FcgammaR interaction and immunogenic cell death

#### Anna Skaletskaya, Jose Ponte, Thomas Chittenden, Yulius Setiady

##### ImmunoGen, Inc., Waltham, MA, USA

###### **Correspondence:** Yulius Setiady (ysetiady@immunogen.com)

**Background**

Mirvetuximab soravtansine (IMGN853) is an ADC, comprising a humanized FRα-binding M9346A antibody linked to the tubulin-disrupting maytansinoid, DM4. IMGN853 binds to FRα on cancer cells and is internalized; DM4 is released through enzymatic degradation of the antibody and linker cleavage, resulting in disruption of cell division and cell death. IMGN853 shows promising single-agent activity and a favorable safety profile in FRα-positive ovarian cancer patients in a phase I study. IMGN853 is entering FORWARD I, a phase III monotherapy study and is also being evaluated in combination with other agents including pembrolizumab in a phase Ib/II study, FORWARD II. Here we have explored potential mechanism(s) whereby IMGN853 can show enhanced activity in combination with a checkpoint inhibitor. Specifically, we report pre-clinical studies that examine the impact of IMGN853 treatment of tumor cells on human monocytes *in vitro*.

**Methods**

Peripheral blood mononuclear cells (PBMC) from normal donors were incubated with FRα-expressing KB tumor cells in the presence of test articles for two days. PMBC were then analyzed for activation marker expression by flow cytometry. The level of immunogenic cell death markers were evaluated by flow cytometry or ELISA to examine the direct impact of IMGN853 on KB cells.

**Results**

IMGN853 treatment of PBMC did not affect monocytes. Incubation of PBMC with KB cells decreased the CD86+ monocytes from ~30 % to ~10 %, and addition of DM4, the free payload of IMGN853, reversed the CD86 expression to basal levels. Intriguingly, addition of IMGN853, but not non-targeting ADC, increased the activated monocytes to ~80 %. Similar results were obtained with isolated CD14+ monocytes, indicating that the monocyte activation is independent of other types of peripheral blood cells. Additionally, comparable increases in monocyte activation were observed in co-cultures of monocytes and KB cells treated with a mixture of M9346A and DM4, and not with M9346A or DM4 alone, suggesting both components of the ADC are necessary. Furthermore, a variant of IMGN853 with a point mutation that abrogates the FcγR binding only produced the same degree of monocyte activation as DM4 treatment, suggesting the significance of Fc/FcγR interaction. Finally, treatment of KB cells with IMGN853 increased calreticulin, ATP and HMGB1, three immunogenic cell death markers which can activate monocytes.

**Conclusions**

Treatment of FRα-expressing KB cells with IMGN853 induces activation of co-cultured monocytes through Fc/FcγR interaction and upregulation of immunogenic cell death markers. These data provides a rationale for the clinical evaluation of IMGN853 and a checkpoint inhibitor.

**Trial Registration**

ClinicalTrials.gov identifier NCT01609556, NCT02631876, and NCT02606305.

### P304 CovIsoLink, a new enzymatic conjugation for the development of innovative antibody drug conjugates

#### Sandrine Valsesia-Wittmann^1^, Eva Sivado^1^, Vincent Thomas^2^, Meddy El Alaoui^1^, Sébastien Papot^3^, Charles Dumontet^4^, Mike Dyson^5^, John McCafferty^5^, Said El Alaoui^2^

##### ^1^Centre Léon Bérard, innovations in immunotherapy platform, Lyon, Rhone-Alpes, France; ^2^Covalab, Villeurbanne, Rhone-Alpes, France; ^3^IC2MP, Poitiers, Limousin, France; ^4^CRCL, Lyon, Rhone-Alpes, France; ^5^IONTAS, Cambridge, England, UK

###### **Correspondence:** Sandrine Valsesia-Wittmann (sandrine.wittmann@lyon.unicancer.fr)

**Background**

Monoclonal antibodies coupled to highly toxic agents or ADC (antibody-drug conjugate) are becoming a significant component of anticancer treatment. Currently approved immunoconjugates are heterogeneous in terms of degree of substitution, which is suboptimal both in terms of antitumor efficacy and risk of toxicity. The aim of this project is to bring the *in vivo* proof of concept of a novel immunoconjugate technology using a unique enzymatic coupling of the payload on a substrate for an enzyme site inserted in the antibody core. These enzyme substrates are small unnatural and innovative peptide (patent pending). The major advantage of this method named CovIsoLink™ is to obtain a homogenous immunoconjugate with uniform stoichiometry by controlling: (a) the location of coupling sites on the antibody without affecting its immunoreactivity and (b) the number of molecules coupled per molecule of antibody by controlling the coupling drug antibody ratio (DAR) and consequently the toxicity and efficacy of therapeutic molecules.

**Methods**

Using an in house peptide library, and the transglutaminase colorometric assay, we identified selected tag peptides that were recognized as glutamine donor substrates with improved affinity compared with the known peptides (such as ZQG, LLQG, etc.). As a proof-of-concept, we compare our CovIsolink immunoconjugates with Kadcyla® (targeting HER2) recently approved.

**Results**

We developed and characterized different recombinant anti Her2 IgG1 mAb carrying optimized enzymatic substrate (tag) by genetic insertion in the coding sequence of mAb. We then evaluate the best linkers and conformation to incorporate different compounds through bacterial transglutaminase (mTG) enzymatic reaction. We set up experimental conditions, production, purification, HPLC/MS analysis and control of the immunoreactivity of CovIsoLink™ Her2-ADC. Using mTG, we obtained site specific conjugation of different modified drugs with optimized linker on the antiHer2 IgG1 antibody. By HIC analysis, we validated a specific and reproducible DAR reaching DAR2 depending on drugs and experimental conditions. *In vitro* and *In vivo* characterization and dose response studies of CovIsolink-ADC specificity and reactivity are currently performed in Her2 positive models by comparison with Kadcyla (T-DM1).

**Conclusions**

This technology is potentially applicable to a large variety of antitumor (or anti-stromal) antibodies since it is neither limited by the specificity of antigen targeted by the antibody nor by drugs that can be engrafted. Site specific conjugation will allow to remove barriers for the use of molecules that are too toxic in systemic injection or to enhance the efficacy of antibodies with low anti-tumor activity.

**Acknowledgements**

Metropole Grand Lyon for funding.

### P305 Efficacy of variable dose of gallic acid to combat chemically induced hepatocarcinogenesis by altering the hepatic proinflammatory cytokines and oxidative stress

#### Amita Verma, Vikas Kumar

##### Sam Higginbotham Institute of Agriculture, Technology & Sciences, Allahabad, Uttar Pradesh, India

###### **Correspondence:** Amita Verma (amitavermadr@gmail.com)

**Background**

Hepatocellular carcinoma (HCC), a hypervascular tumor, one of the most cancers worldwide, it causes the cancer related mortality. Hepatic inflammation and oxidative stress plays an important role in the development of HCC, which occur due to viral infections, environmental carcinogens, dietary carcinogens as well as alcohol abuses. We have found that gallic acid, a dietary flavonoids present in various plants avoid diethyl nitrosamine (DENA) induced hepatic carcinoma in experimental rats through inhibit the oxidative stress and repression of inflammation. The aim of the present study to investigate the chemoprotective effect of gallic acid on hepatic cytokines in the DENA induced carcinogenesis rats.

**Methods**

DENA (200 mg/kg) used for the induction of HCC in the Wistar rats. The DENA treated rats were divided into different groups and received the doses of gallic acid (25, 50 and 100 mg/kg) for 22 weeks different biochemical alpha feto-protein (AFP), serum glutamate oxaloacetate transaminase (SGOT), serum glutamate pyruvate transaminase (SGPT), alkaline phosphatase (ALP); hematological parameters viz., red blood cells (RBC), white blood cells (WBC), hemoglobin (Hb), erythrocytes sedimentation rate (ESR); antioxidant markers viz., lipid peroxidation (LPO), superoxide dismutase (SOD), catalase (CAT), reduced glutathione (GSH); inflammatory mediators viz., tumor necrosis factor-α (TNF--α), interlukin-6 (IL-6) and interlukin-1α (IL-1α) were estimated.

**Results**

DENA induced rats received the different doses of the gallic acid at dose dependent manner until 22 weeks. The hepatic serum, antioxidant markers and hematological parameters significantly (P < 0.001) altered at effective dose dependent manner. The level of proinflammatory cytokines viz., TNF-α, IL-6 and IL-1α significantly altered by the gallic acid at dose dependent manner. The histopathology study showed from the test group exhibited almost normal architecture as compared to HCC control rats.

**Conclusions**

It can be concluded that gallic acid mediated chemoprotective effect of DENA induced hepatocarcinogenesis is related to alteration of oxidative stress as well as proinflammatory cytokines.

**References**

1. Kumar V: **Effect of α-magostin on nitrosamine induced hepatic carcinogenicity in neonatal pups.** *Ann Oncol* 2015, **26 (suppl_9)**:1–7.

2. Anwar, *et al.*: **Anticancer effect of rosiglitazone in rats treated with Nnitrosodiethylamine via inhibition of DNA synthesis: an implication for hepatocellular carcinoma**. *RSC Adv* 2015, **5**:68385.

3. Kumar, *et al.*: **Fixed dose combination therapy loperamide and niacin ameliorates diethylnitrosamine-induced liver carcinogenesis in albino wistar rats**. *RSC Advances* 2015, **5**:67996–68002.

## Oncolytic Viruses

### P306 The oncolytic effect of talimogene laherparepvec (T-VEC) can be augmented by MEK inhibition in melanoma cell lines

#### Praveen K Bommareddy^1^, Howard L Kaufman^2^, Andrew Zloza^2^, Frederick Kohlhapp^1^, Ann W Silk^1^, Sachin Jhawar^1^, Tomas Paneque^3^

##### ^1^Rutgers University, New Brunswick, NJ, USA; ^2^Rutgers Cancer Institute of New Jersey, New Brunswick, NJ, USA; ^3^Rutgers Robert Wood Johnson Medical School, Somerset, NJ, USA

###### **Correspondence:** Praveen K Bommareddy **(**pkb38@gsbs.rutgers.edu)

**Background**

Talimogene laherparepevec (T-VEC) is an engineered oncolytic herpes simplex virus, type 1 (HSV-1) encoding GM-CSF that has been approved for treating melanoma. Oncolytic viruses may preferentially replicate in cancer cells due to defects in oncogenic signaling pathways that promote cell survival and allow time for more complete viral replication and assembly by suppressing apoptotic machinery including double-stranded RNA-dependent protein kinase (PKR). It is thought that the MAPK pathway interacts with PKR, but the mechanism is poorly understood. Since the MAPK pathway is frequently mutated in melanoma cells, we sought to determine if mutations in BRAF or NRAS might enhance T-VEC-mediated oncolysis. We further sought to determine if BRAF or MEK inhibition might influence the oncolytic potential of the virus.

**Methods**

Melanoma cell lines were selected for BRAF, NRAS, and other mutations for evaluation. The cells were plated in 96 well plates (10^4^ cells/well) and treated with a range of T-VEC doses (MOI of 1–0.001). Cell viability was assessed by the AlamarBlue assay. After establishing baseline viability results, cells were concurrently treated with T-VEC and MEK inhibitors (trametinib or PD0325901 at doses 100nM-1nM) or BRAF inhibitor (vemurafenib, 100nM-1nM) and cell viability determined by AlamarBlue assay. For *in vivo* viral propagation, NSG mice were challenged with SK-MEL-28 (5x10^6^) at day 0. Mice were treated with T-VEC (3x10^6^ PFU) and/or trametinib (0.3 mg/kg) on day 10 and day 12. Tumors were harvested (day 17) and viral load was determined by immunoblotting for HSV-1 glycoprotein D. Statistical comparisons between treatment groups were determined using the Student’s t test with P < 0.05 being considered statistically significant.

**Results**

All cell lines were highly susceptible to lysis by T-VEC although this effect was reduced when the MOI was decreased to 0.001 for most cells. There was no correlation between BRAF or NRAS mutation status and cell viability at tested doses. There was also no effect of vemurafenib on cell viability following T-VEC infection. However, treatment with the combination of T-VEC and trametinib significantly increased cancer cell death (P < 0.01). A similar effect was seen with a second MEK inhibitor (PD0325901; P < 0.001).

**Conclusions**

The combination of T-VEC with MEK inhibition resulted in enhanced *in vitro* tumor cell killing and viral propagation. Combining MEK inhibitors with T-VEC represents an attractive therapeutic option and further studies are needed to understand if oncolytic viruses *in vivo* might limit resistance to MEK inhibition.

### P307 Oncolytic herpes simplex virus, type 1 (HSV-1) encoding GM-CSF induces HSV-1 glycoprotein B-specific T cell responses that traffic to injected and un-injected melanoma tumors

#### Praveen K Bommareddy^1^, Frederick Kohlhapp^1^, Jenna Newman^1^, Pedro Beltran, Andrew Zloza^2^, Howard L Kaufman^2^

##### ^1^Rutgers University, New Brunswick, NJ, USA; ^2^Rutgers Cancer Institute of New Jersey, New Brunswick, NJ, USA

###### **Correspondence:** Praveen K Bommareddy (pkb38@gsbs.rutgers.edu)

**Background**

The first oncolytic virus using an attenuated oncolytic herpes simplex virus, type 1 (HSV-1) encoding GM-CSF, called talimogene laherparepevec (T-VEC) has been approved for the treatment of accessible and unresectable melanoma. T-VEC enters tumor cells through Nectin cell surface receptors and results in cell lysis and induction of anti-tumor immunity. We previously identified MART-1-specific CD8+ effector T cells within the tumor microenvironment of T-VEC-treated lesions but the contribution of viral-specific T cell responses to the anti-tumor activity following T-VEC treatment has not been reported. To determine if there might be a role for HSV-1-specific T cells, we developed a murine-adapted model in which the complete T-VEC backbone was used with the substitution of murine GM-CSF (mTVEC) in place of the human gene. We hypothesized that anti-viral T cell responses might contribute to the anti-tumor activity.

**Methods**

C57/BL6 mice were challenged with B16-nectin (10^5^) tumor cells on the right and left flank (day 0). mTVEC (3*10^6^ PFU) was injected into the right flank tumor (day 7). Tumor growth was monitored every 2–3 days by caliper measurement. Tumors were harvested at various times and analyzed by flow cytometry for NK cells, antigen-presenting cells, T cells, and activation markers. Antigen specificity was determined using gp100 and HSV-1 glycoprotein B tetramers. Statistics were determined using the Student’s t test with P < 0.05 being considered statistically significant.

**Results**

mTVEC resulted in significant regression of injected, right flank tumors with minimal effects on the growth of un-injected, left flank tumors (P < 0.0001). We observed a significant increase in tumor immune infiltration into the injected lesion, with a lower number of immune cells in the un-injected sites (P < 0.01). The pattern and the timing of immune infiltration mimicked that of acute viral infection with an initial infiltration of NK cells and antigen-presenting cells followed by infiltration of HSV-1 gB-specific CD8+ T cells that peaked at day 7 post injection. Additionally, we observed that HSV-1-specific CD8+ T cells trafficked to un-injected lesions. In this model, gp100 T cell responses were not detected.

**Conclusions**

Our data indicate that oncolytic HSV-1-GM-CSF mediates anti-tumor therapeutic responses in a mouse model of melanoma, treatment induces a typical anti-viral innate and adaptive immune response, and HSV-1-specific T cells can be generated and traffic to both injected and un-injected tumors. These data suggest that anti-viral T cell responses may contribute to the anti-tumor effects of HSV-1-based oncolytic virus immunotherapy.

### P308 A CD47-blocking oncolytic vaccinia virus for cancer therapy

#### Felicia Cao, Bang-Xing Hong, Tania Rodriguez-Cruz, Xiao-Tong Song, Stephen Gottschalk

##### Center for Cell and Gene Therapy, Texas Children’s Hospital, Houston Methodist, Baylor College of Medicine, Houston, TX, USA

###### **Correspondence:**Felicia Cao (feliciac@bcm.edu)

**Background**

Oncolytic vaccinia viruses (VV) have shown antitumor activity in early phase clinical studies but few patients have been cured, likely due to the inability of VVs to kill all tumor cells. Tumor-associated macrophages (TAMs) are key in creating an immunosuppressive tumor microenvironment. However, recent studies have shown that the phagocytic capacity of TAMs can be harnessed to induce antitumor responses by blocking CD47 and providing a phagocytosis signal using a chimeric molecule composed of the high-affinity ectodomain of SIRPα fused to IgG4 Fc (SIRPα-Fc). We propose to adapt this approach to oncolytic VVs and hypothesize that an oncolytic VV genetically modified to express SIRPα-Fc (SIRPα-Fc-VV) has enhanced antitumor activity.

**Methods**

To initially evaluate our strategy we genetically modified CD47+ tumor cells (OV10.315, MC38, Raji) with lentiviral vectors encoding SIRPα or SIRPα-Fc.

**Results**

*In vitro*, OV10.315.SIRPα-Fc cells were readily phagocytosed by M1 and M2 macrophages in comparison to OV10.315.SIRPα and non-transduced (NT) OV10.315 cells (p < 0.05). *In vivo*, Raji.SIRPα-Fc cells had decreased tumorigenicity in comparison to Raji.SIRPα and NT Raji cells post IV injection in NSG mice resulting in a significant survival advantage (p < 0.05). 4/5 C57BL/6 mice rejected MC38.SIRPα and MC38.SIRPα-Fc cells post SC injection in contrast to 0/4 NT MC38 cells (p < 0.05). Having established that SIRPα-Fc expression in tumor cells has the desired therapeutic effect, we generated VVs encoding either SIRPα or SIRPα-Fc (SIRPα-VV or SIRPα-Fc-VV). *In vitro*, both SIRPα-VV and SIRPα-Fc-VV had similar oncolytic activity compared to parental VV (VSC20). The expression and secretion of SIRPα and SIRPα-Fc was confirmed by FACS analysis. In co-culture assays, only SIRPα-Fc-VV was able to induce M1 and M2 macrophage killing. Based on our encouraging *in vitro* experiments, we have initiated *in vivo* studies to test the safety and efficacy of SIRPα-Fc-VV in the MC38 C57BL/6 model.

**Conclusions**

We demonstrate here that tumor cells secreting SIRPα-Fc are readily phagocytosed by M1 and M2 macrophages and have reduced tumorigenicity in xenograft and immune competent animal models. In addition, SIRPα-Fc-VVs readily infect tumor cells and induce phagocytosis by macrophages. Thus, arming oncolytic viruses such as VVs with SIRPα-Fc has the potential to improve their antitumor activity.

### P309 Selective activation of innate immune responses by the Ad11/Ad3 chimeric oncolytic group B adenovirus enadenotucirev

#### Hugo Calderon^1^, Sam Illingworth^1^, Alice Brown^1^, Kerry Fisher^1^, Len Seymour^2^, Brian Champion^1^

##### ^1^PsiOxus Therapeutics Ltd, Abingdon, England, UK; ^2^Oxford University, Oxford, England, UK

###### **Correspondence:** Brian Champion (brian.champion@psioxus.com)

**Background**

Oncolytic viruses (OVs) are characterized by their ability to selectively infect and kill tumor cells. More recently they have been exploited for their capacity to be encoded with, and locally deliver, a variety of payloads including immunotherapeutic transgenes to enhance immune responses against the tumor. Viral properties of OVs may also be able to engage the innate immune system and thus influence the suppressive nature of the tumor microenvironment. A better understanding of these interactions may help guide both the rational design of ‘armed’ viruses as well as the design of strategies for combining with other immunotherapies. Enadenotucirev (EnAd) is a chimeric Ad11/Ad3 group B oncolytic adenovirus under development for the systemic treatment of metastatic carcinomas. Unlike the group C virus Ad5, EnAd does not bind to cells via the CAR receptor but instead uses CD46 which is expressed by innate immune cells.

**Methods**

We have been evaluating the effect of EnAd on innate immune responses using *in vitro* immature human monocyte-derived dendritic cells (DC) as a model suppressive phenotype APC.

**Results**

EnAd induced up-regulation of surface activation markers and induced the production of pro-inflammatory cytokines. Further mechanistic experiments, comparing the effects of EnAd to those of Ad5 indicated that the activation was selective for EnAd, was particle-mediated and dependent on CD46 binding. In order to understand the functional implications downstream of these interactions, T cell activation and phenotype was assessed using an allogeneic mixed lymphocyte reaction approach. EnAd-treated DCs selectively stimulated stronger T cell responses, including enhanced IFNg production. The data supports EnAd as a good candidate OV for steering the response of T cells activated within the tumor towards a Th1 phenotype for enhanced effector responses.

**Conclusions**

Thus, as well as its potent oncolytic properties, EnAd particles may also function in the tumor microenvironment to help drive functional adaptive immune responses by inducing pro-inflammatory phenotype APCs, which should also synergize effectively with other immunotherapy strategies.

### P310 CD40L-armed oncolytic LOAd viruses control growth of CD40+ T24 bladder cancer via both oncolysis and CD40-mediated apoptosis

#### Emma Eriksson^1^, Jessica Wenthe^1^, Ann-Charlotte Hellström^1^, Gabriella Paul-Wetterberg^1^, Angelica Loskog^2^

##### ^1^Uppsala University, Uppsala, Sweden; ^2^Uppsala University, Lokon Pharma AB, Uppsala, Sweden

###### **Correspondence:** Emma Eriksson (emma.eriksson@igp.uu.se)

**Background**

CD40-CD40L signaling is a powerful pathway that can be used in cancer immunotherapy. CD40 stimulation of immune cells drives a Th1 anti-tumor response but CD40 stimulation on tumor cells can lead to enhanced tumor cell apoptosis. This is due to the up-regulation of FASL, tumor necrosis factor alpha (TNFα), and TNF-related apoptosis-inducting ligand (TRAIL) on tumor cells after CD40-CD40L interaction. Also, CD40 signaling in tumor cells can induce activation of caspases through the binding of TRAF 2. LOAd viruses are oncolytic adenoviruses that encode a trimerized form of CD40L (TMZ-CD40L) alone (LOAd700) or in combination with 4-1BBL (LOAd703). In the current study, the role of CD40L on CD40+ tumor cells has been elucidated.

**Methods**

The cell viability post virus infection of CD40+ T24 tumor cells was investigated *in vitro* by MTS assay. To further investigate the cell death induced by CD40L signaling apart from oncolysis due to LOAd virus infection, monocyte-derived dendritic cells were infected with LOAd(−) and LOAd700 and then co-cultured with T24 cells. Apoptosis induction was investigated at 48 hours post co-culture initiation by flow cytometry for Annexin V and 7-AAD. In a T24 xenograft model using Nu/Nu immunodeficient mice, LOAd viruses expressing human TMZ-CD40L that does not cross-react to murine CD40 was used (6x) to evaluate *in vivo* efficacy. LOAd(−) was used as a control of growth control by oncolysis and PBS-treated controls determined normal growth rate.

**Results**

The LOAd viruses induced oncolysis of the CD40+ urinary bladder cancer T24 cell line independently of transgene expression. However, infected T24 showed a significant decrease in cell viability after infection with TMZ-CD40L-expressing LOAd700 compared to LOAd(−). Co-culture of LOAd-infected dendritic cells expressing TMZ-CD40L or not with T24 led to an increased induction of apoptosis when co-cultured with dendritic cells expressing TMZ-CD40L. *In vivo*, both LOAd(−) and LOAd703 treatment led to a decreased tumor growth compared to PBS-treated animals. When TMZ-CD40L was expressed (e.g. LOAd703), tumor control was faster and at end point, only 1/5 animals had tumor growth compared to 3/5 in the LOAd(−)-treated group, demonstrating the additional growth control by CD40-induced tumor cell death. Models with CD40- tumor cells (Panc01, H727, SKOV3) responded similarly to control virus and virus expressing TMZ-CD40L.

**Conclusions**

Oncolytic viruses encoding TMZ-CD40L have an increased killing capacity via CD40L-mediated killing of CD40+ tumor cells.

### P311 A novel oncolytic adenovirus expressing immunostimulatory genes that promotes an anti-tumor response

#### Emma Eriksson^1^, Ioanna Milenova^2^, Jessica Wenthe^1^, Magnus Ståhle^1^, Justyna Jarblad-Leja^3^, Gustav Ullenhag^4^, Anna Dimberg^1^, Rafael Moreno^5^, Ramon Alemany^5^, Angelica Loskog^6^

##### ^1^Uppsala University, Uppsala, Sweden; ^2^Uppsala University, Amsterdam, Netherlands; ^3^Uppsala University, Immuneed AB, Uppsala, Sweden; ^4^Uppsala University, Uppsala University Hospital, Uppsala, Sweden; ^5^Institut Catalá d'Oncologia, Barcelona, Spain; ^6^Uppsala University, Lokon Pharma AB, Uppsala, Sweden

###### **Correspondence:** Emma Eriksson (emma.eriksson@igp.uu.se)

**Background**

Immunotherapies aim to break the tolerance of immune cells seen in cancer patients and redirect the response from a pro-tumor to an anti-tumor response. There are many ways to achieve anti-tumor immunity, for example by stimulation of immunostimulatory pathways. CD40L interactions with its receptor CD40 on dendritic cells leads to maturation of these cells and polarization towards a Th1 response. CD40L can also reduce the level of myeloid suppressor cells and M2 macrophages as well as induce apoptosis in CD40 positive tumor cells. 4-1BB ligand (4-1BBL) interactions with its receptor 4-1BB on T cells leads to activation and survival of the cells and can expand memory T cells. Herein, we present an oncolytic adenovirus with a CMV-driven transgene cassette containing the human transgenes for a trimerized, membrane-bound CD40 ligand (TMZ-CD40L) and the full length 4-1BBL.

**Methods**

Pancreatic cell lines and exocrine cells from healthy donors were infected with LOAd703 and analyzed for cell death 48 and 72 hours post-infection with MTS-assay. Immunodeficient mice with established human Panc01 tumors were treated twice a week for three weeks and evaluated for tumor size. Both the *in vitro* and *in vivo* experiments were repeated in combination with gemcitabine. Dendritic cells were infected with the virus and evaluated by flow cytometry and ProSeek. The dendritic cells were also pulsed with CMV peptides and co-cultured with autologous CD14- cells to investigate the expansion of CMV+ T cells by flow cytometry.

**Results**

LOAd703 decreased the viability of pancreatic tumor cells at both 48 hours and 72 hours as compared to cells infected with a non-replication competent virus but spared healthy exocrine cells. Mice treated with LOAd703 had a decreased tumor burden compared to PBS treated animals. LOAd703 could be successfully combined with gemcitabine without any negative effects on oncolysis both *in vitro* and *in vivo*. Dendritic cells infected with LOAd703 showed a mature phenotype with expression of CD83, CD86, and secretion of cytokines, chemokines including IL12p70 and IFNγ. The dendritic cells were also functional and could expand antigen-specific CMV+ T cells and NK cells.

**Conclusions**

In conclusion, LOAd703 is a novel oncolytic virus that targets both the tumor with oncolysis and the immune system with Th1 type response from dendritic cells and an expansion of antigen-specific T cells. The next step is to bring the virus from the lab bench to the bedside in a clinical trial to elucidate its effect in pancreatic cancer (NCT02705196).

### P312 An oncolytic virus targeting tumor cell survival, desmoplasia and immune activation in pancreatic cancer

#### Emma Eriksson^1^, Ioanna Milenova^2^, Rafael Moreno^3^, Ramon Alemany^3^, Angelica Loskog^4^

##### ^1^Uppsala University, Uppsala, Sweden; ^2^Uppsala University, Amsterdam, Netherlands; ^3^Institut Catalá d'Oncologia, Barcelona, Spain; ^4^Uppsala University, Lokon Pharma AB, Uppsala, Sweden

###### **Correspondence:** Emma Eriksson (emma.eriksson@igp.uu.se)

**Background**

The tumor microenvironment supports the tumor cells. In pancreatic cancer, stellate cells, immune cells and extracellular matrix compose the majority of the lesions and create a condition referred to as desmoplasia. IL6 drives STAT3 activation leading to transforming growth factor (TGF) beta and collagen type 1 production. TGF beta also promotes immunosuppression by inhibition of T cells and expansion of T regulatory cells (Tregs). Hence, IL6, which is overexpressed in pancreatic cancer, is one of the regulators of desmoplasia. Further, IL6 is associated to poor prognosis of pancreatic cancer. In order to target both IL6 and induce immune activation, the oncolytic adenovirus LOAd713 was developed. It is double-armed with an anti-IL6 receptor antibody single chain fragment (aIL6R scFv) aiming to disrupt IL6 signaling and a trimerized membrane-bound CD40 ligand (TMZ-CD40L) that drives Th1 immunity.

**Methods**

LOAd713 is an Ad5/35 virus that replicates only in cells with a dysfunctional retinoblastoma pathway (E1Adelta24). Further, the serotype 5 fiber was changed to a serotype 35 fiber to target CD46 expressed by most tumors. A CMV-driven transgene cassette with the transgenes for TMZ-CD40L and aIL6R scFv was added into the genome. The activity of LOAd713 was evaluated by 1) infecting pancreatic tumor cell lines and evaluating their viability in a MTS cytotoxicity assays (oncolysis), 2) by infecting human dendritic cells (DC) and performing phenotypic assays by flow cytometry, cytokine arrays and lymphocyte stimulation assays (immune activation), and 3) by infecting pancreatic stellate cells and investigating biological changes in a proteomic analysis (ProSeek).

**Results**

LOAd713 had oncolytic capacity in a panel of pancreatic cancer cell lines as shown by the viability analysis post infection while pancreatic stellate cells infected with LOAd713 did not lose viability. However, LOAd713 significantly decreased the expression of hepatocyte growth factor (HGF), TGF beta, fibroblast growth factor-5 (FGF-5) and collagen type I, all connected to stellate cell function and desmoplasia. Nevertheless, LOAd713-infected stellate cells increased their expression of IL1 alpha, IL6, IL8, CXCL10 and CCL20, which may both promote angiogenesis and attract lymphocytes. LOAd713-infected DCs showed an increased level of maturation markers such as CD83 and IL12 as shown by flow cytometry and luminex methodology, and such DCs could expand antigen-specific T cells.

**Conclusions**

LOAd713 is an oncolytic adenovirus aiming to interrupt the IL6/IL6R pathway resulting in reduced factors that drive desmoplasia. Further, via TMZ-CD40L, LOAd713 can activate DCs to drive lymphocyte responses.

### P313 Radiation therapy augments the effect of talimogene laherparepvec (T-VEC) on melanoma cell viability

#### Sachin Jhawar^1^, Sharad Goyal^2^, Praveen K Bommareddy^1^, Tomas Paneque^3^, Howard L Kaufman^2^, Andrew Zloza^2^

##### ^1^Rutgers University, New Brunswick, NJ, USA; ^2^Rutgers Cancer Institute of New Jersey, New Brunswick, NJ, USA; ^3^Rutgers Robert Wood Johnson Medical School, Somerset, NJ, USA

###### **Correspondence:** Sachin Jhawar (jhawarsr@cinj.rutgers.edu)

**Background**

The oncolytic herpes virus talimogene laherparepvec (T-VEC), engineered to express GM-CSF, is the first oncolytic virus approved for treatment of cancer in the US. T-VEC treatment increases median overall survival (OS) in patients with locally advanced and metastatic melanoma; however, a majority of treated patients still progress on this therapy. Radiation therapy (RT) in combination with immunotherapies has been shown to improve response rates in melanoma (compared to either modality alone) and may exhibit different cytotoxic and immunoloregulatory effects on tumors than T-VEC. Therefore, we hypothesized that combination RT and T-VEC may represent a potentially synergistic therapeutic approach and investigated the effect of this combination.

**Methods**

Human melanoma cell lines cultured in 96-well plates (7x10^3^ cells per well) were treated in triplicate with RT (0, 4, or 8 Gy) delivered via the Gammacell 40 exactor. Twelve hours later the cells were further treated with T-VEC (0, 0.01, 0.1, and 1 MOI) for 60 hours. The effects of RT and T-VEC were determined by AlamarBlue cell viability assay performed 72 hours after initial treatment. A P value < 0.05 obtained using the Student’s t test was considered to denote a statistically significant difference between groups.

**Results**

Treatment of UACC62 melanoma cells with T-VEC (1 MOI) alone resulted in a 12 % decrease in cell viability compared to no treatment (P < 0.013), while treatment with RT (8 Gy) did not result in a significant decrease (2.1 %, P = 0.42). However, combination RT (8 Gy) and TVEC (1 MOI) resulted in a significant decrease in cell viability (21 %, P < 0.001) compared with no treatment. This likewise represented a significant decrease compared to RT alone (p = 0.0028) or T-VEC alone (p = 0.0389). Similar findings were noted in experiments utilizing other melanoma cell lines.

**Conclusions**

Treatment with combination RT and T-VEC results in an *in vitro* decrease in melanoma cell viability. Further studies are needed to understand the mechanism underlying the reported synergy, the effect of radiation on viral propagation, the effect of viral replication on radiation sensitivity, and whether this approach can be used in patients resistant to either modality alone or to other single and combination immunotherapies. Studies assessing this combination therapy in other solid tumors and in pre-clinical *in vivo* immune-competent autologous double-humanized mouse models are currently underway.

### P314 Interim results of the CAPRA clinical trial: CAVATAK and pemrolizumab in advanced melanoma

#### Howard L Kaufman^1^, Ann Silk^1^, Janice Mehnert^1^, Nashat Gabrail^2^, Jennifer Bryan^1^, Daniel Medina^1^, Praveen K Bommareddy^1^, Darren Shafren^3^, Mark Grose^3^, Andrew Zloza^1^

##### ^1^Rutgers Cancer Institute of New Jersey, New Brunswick, NJ, USA; ^2^Gabrail Cancer Center, Canton, OH, USA; ^3^Viralytics Limited, Sydney, New South Wales, Australia

###### **Correspondence:** Howard L Kaufman (howard.kaufman@rutgers.edu)

**Background**

CAVATAKä is a novel bio-selected oncolytic, immunotherapeutic Coxsackievirus A21 (CVA21) strain. Intratumoral (i.t.) CAVATAKä injection can induce selective tumor-cell infection, immune-cell infiltration, g-INF response gene up-regulation, increased PD-L1 expression, tumor cell lysis and systemic immune responses. Preclinical studies in an immune-competent mouse model of melanoma have revealed that combinations of i.t. CVA21 and anti-PD-1 blockade mediate significantly greater antitumor activity compared to use of either agent alone. The presented clinical trial evaluates combination CAVATAKä and pembrolizumab based on increased expression of PD-L1 following virus administration and higher response rates of pembrolizumab in patients with increased tumor PD-L1.

**Methods**

This is a phase I trial of i.t. CVA21 and pembrolizumab for treated or untreated unresectable Stage IIIC-IVM1c melanoma. Patients receive up to 3 x 10^8^ TCID_50_ CVA21 i.t. on days 1,3,5,8 and 22, and then every 3w for up to 19 injections. Patients also receive pembrolizumab (2 mg/kg) i.v. every 3w starting day 8. The primary endpoint is safety/tolerability by incidence of dose-limiting toxicity. Secondary endpoints include response rate, immune-related progression-free survival at 12 m, PFS hazard ratio, 1y and overall survival. In addition, quality-of-life, time to initial response, durable response rate, changes in melanoma-specific T cells, PD-L1 expression and Th1/Th2 gene expression profiles, will be determined. Safety will be assessed using NCI CTCAE. Response will utilize Simon's two-stage design. In the first stage, 12 subjects will be accrued. If 2 or fewer responses occur within 12 m of starting treatment, the study will be stopped due to futility of treatment. Otherwise, 18 additional subjects will be accrued.

**Results**

To-date, 10 subjects have been enrolled with one patient leaving the study with PD and another patient with a non-treatment-related adverse event. Overall, adverse events have generally been low-grade constitutional symptoms related to CVA21 and standard pembrolizumab-related side effects. Preliminary observations have revealed reduction in a number of injected and non-injected lesions, with a number of patients displaying evidence of post-injection systemic exposure to CVA21.

**Conclusions**

At present combination CVA21 and pembrolizumab appears to be generally safe and well-tolerated in an interim analysis of patients with advanced melanoma. Early analysis identified reductions in a number of injected and non-injected lesions and we look forward to evaluating a more mature overall response data set. Combination CVA21 and pembrolizumab may represent a new approach for the treatment of advanced melanoma.

**Acknowledgements**

We would like to acknowledge the patients and families that participated in the clinical trial.

### P315 Gene transfer of cytosine deaminase with Toca 511 and subsequent treatment with 5-fluorocytosine induces anti-tumor immunity

#### Leah Mitchell, Kader Yagiz, Fernando Lopez, Daniel Mendoza, Anthony Munday, Harry Gruber, Douglas Jolly

##### Tocagen Inc., San Diego, CA, USA

###### **Correspondence:** Leah Mitchell (lmitchell@tocagen.com)

**Background**

Toca 511 (vocimagene amiretrorepvec) is a gene therapy which utilizes a gamma retroviral replicating vector encoding cytosine deaminase to selectively infect cancer cells. When used in combination with the prodrug, 5-fluorocytosine (5-FC), Toca 511 and 5-FC kill tumor cells by local production of 5-fluorouracil (5-FU), and induce a local immunotherapeutic effect that results in long-term survival after cessation of 5-FC treatment. The work described herein identifies the immune cell populations that change over time following administration of 5-FC as well as the role of T cells in long-term anti-tumor immune memory.

**Methods**

A mouse glioma cell line, Tu-2449SC (2 % pretransduced with Toca 511) was injected subcutaneously in B6C3F1 mice. 5-FC (500 mg/kg, IP, SID) or PBS treatment was initiated once tumors were palpable, for 5 consecutive days followed by 2 days off drug. This treatment cycle was continued for the duration of the study. At 3, 6, 9, and 14 days after 5-FC start, tumors were harvested for immunophenotyping.

**Results**

Tumor burden was significantly reduced within 14 days of treatment in mice that received 5-FC vs. PBS control. By day 6 post 5-FC treatment initiation, tumor associated macrophages (TAM), myeloid derived suppressor cells (MDSC), and monocyte populations were significantly reduced in treated tumors compared to PBS controls. This myeloid cell depletion effect correlates with previous work by others [1] using systemic 5-FU but was complemented by an attractive pharmacokinetic profile with high levels of 5-FU in tumor tissue and undetectable levels of 5-FU in the plasma, thus avoiding systemic myelotoxicity. At 14 days post 5-FC treatment start, TAM and MDSC remained reduced in tumors of treated animals, and both CD4+ and CD8+ T cells were significantly increased. Additionally we noted that treatment with 5-FC induced high expression of IFNγ in CD8+ T cells and polarized CD4+ T helper cells away from Th2 and Th17 differentiation pathways. Tumors were completely cleared from greater than 50 % of animals treated with 5-FC and such animals resisted subsequent rechallenge at a distant site with the virus-free parental cell line. Further, adoptive transfer of splenocytes from these cured and now immunized animals led to clearance of established, orthotopic Tu-2449 tumors in recipient naïve animals as long as the donor cell transfer contained T cells.

**Conclusions**

Toca 511 + 5-FC treatment results in reduced tumor burden and creates a tumor microenvironment that is more permissive to immune activation and ultimately establishment of anti-tumor immune response.

**References**

1. Vincent J, Mignot G, Chalmin F, Ladoire S, Bruchard M, Chevriaux A, *et al.*: **5-Fluorouracil selectively kills tumor-associated myeloid-derived suppressor cells resulting in enhanced T cell-dependent antitumor immunity.** *Cancer Res* 2010, **70(8)**:3052–3061.

### P316 T-Stealth™ technology mitigates antagonism between oncolytic viruses and the immune system through viral evasion of anti-viral T cells

#### Steven Fuhrmann^1^, Sasa Radoja^1^, Wei Tan^1^, Aldo Pourchet^2^, Alan Frey^2^, Ian Mohr^2^, Matthew Mulvey^1^

##### ^1^BeneVir Biopharm, Inc., Gaithersburg, MD, USA; ^2^New York University Langone School of Medicine, New York, NY, USA

###### **Correspondence:** Matthew Mulvey (matt@benevir.com)

**Background**

The immune system is both friend and foe to oncolytic viruses (OV). It is a friend because OV rely on anti-tumor cytotoxic T lymphocytes (CTL) for a major component of their clinical efficacy. It is a foe because CTL that recognize viral antigens can kill infected cells. This blocks viral spread by terminating *in situ* viral progeny production. In this way, anti-viral CTL limit the number of virally killed cancer cells and blunt induction of tumor neo-antigen CTL necessary for achieving durable patient responses. BeneVir’s T-Stealth™ OV arming technology blocks display of viral antigens on the surface of infected cells. This promotes viral spread and persistence in the tumor microenvironment because it renders infected tumor cells invisible to anti-viral CTL. By evading anti-viral CTL, T-Stealth™ armed OV kill more cancer cells in the context of an inflamed tumor microenvironment resulting in enhanced induction of anti-tumor CTL. T-Stealth™ armed OV are designed to combine especially well with immune checkpoint inhibitors (ICI). This is because ICI facilitate both anti-tumor as well as anti-viral CTL effector function in the tumor microenvironment and exacerbate the friend vs. foe dynamic between OV and the immune system.

**Methods**

We generated an attenuated, replication competent HSV-1 OV encoding T-Stealth™ technology as well as viruses that do not encode T-Stealth™ technology or encode murine GM-CSF. These viruses were tested for their ability to control the growth of both virally infected as well as uninfected tumors in multiple syngeneic murine tumor models.

**Results**

Compared to control viruses that do not encode T-Stealth™ technology or express murine GM-CSF, the T-Stealth™ armed OV persisted longer in the tumor microenvironment and enhanced the generation of anti-tumor CTL. Simultaneous treatment of mice with the T-Stealth™ armed HSV-1 and ICI against PD-1 and CTLA-4 yielded synergistic results in an aggressive bi-lateral murine tumor model. Detailed analysis of lymphocytes in untreated distant tumors revealed that the T-Stealth™ armed HSV-1 induces greater infiltration of CD8+ CTL and significantly increases TCR diversity.

**Conclusions**

T-Stealth™ technology allows OV to resist premature clearance by anti-viral CTL. Because ICI enhance the ability of anti-viral CTL to clear OV from the tumor microenvironment, T-Stealth™ armed OV hold great promise for synergism with ICI in simultaneous combination regimens. The first T-Stealth™ armed OV is currently undergoing cGMP manufacturing and a phase I trial in solid tumors will commence in late 2017.

### P317 PeptiCRAd: an innovative oncolytic vaccine platform to tilt immune response from virus to tumor

#### Tuuli Ranki^1^, Sari Pesonen^1^, Cristian Capasso^2^, Erkko Ylösmäki^2^, Vincenzo Cerullo^2^

##### ^1^PeptiCRAd Oy, Helsinki, Uusimaa, Finland; ^2^University of Helsinki, Helsinki, Uusimaa, Finland

###### **Correspondence:** Tuuli Ranki (ranki.tuuli@gmail.com)

**Background**

Several strategies are being evaluated to activate the anti-tumor immunity for combinations with checkpoint blockade. Among these agents, viruses are promising for their natural immunogenicity and the interferon gamma response they induce. Many different viruses are being tested in the clinic as tumor vaccinations, and preliminary clinical data suggests that a local virus vaccination can induce a cellular anti-tumor response. However, virus-induced anti-tumor responses seem to be a rare exception, likely due to the initial host response to the virus overwhelming the initiation of any meaningful responses directed against tumor. Means to divert the immune response from virus towards tumor are needed to fully exploit the potential of viruses to activate clinically relevant anti-tumor immunity.

**Methods**

PeptiCRAd is an oncolytic adenovirus vaccine platform where MHC I-restricted immunogenic tumor peptides, that have been modified to become positively charged, are adsorbed onto the negatively charged viral capsid via electrostatic interactions. We used the classical proof-of-concept SIINFEKL peptide (the chicken ovalbumin model) as well as clinically relevant human melanoma peptides complexed with an oncolytic adenovirus, and analyzed the characteristics and efficacy of our approach in different *in vitro* and immunocompetent *in vivo* models of murine and human melanoma.

**Results**

Our results clearly show that PeptiCRAd is superior to oncolytic virus or peptide vaccinations. Importantly, the adsorption of peptides to the virus capsid is crucial, as virus in combination with non-complexed peptides was significantly less effective than PeptiCRAd. Treatment with PeptiCRAd resulted in clear growth reduction both in treated and distant tumors in aggressive immunocompetent melanoma tumor model. With PeptiCRAd approach the peptides were efficiently cross-presented in an MHC I restricted manner and both the frequency of dendritic cells cross-presenting the PeptiCRAd peptides and the frequency of peptide-specific CD8+ T cells were clearly elevated in PeptiCRAd treated animals compared to virus alone or virus with non-complexed peptide.

**Conclusions**

PeptiCRAd is a novel oncolytic vaccine platform which innovatively combines adjuvant (virus) and tumor epitopes to enable a robust anti-tumor immune response. This simple strategy rapidly and cost-efficiently adapts to different tumors and antigens without the need to manipulate the viral backbone. A phase I/II clinical trial is currently under preparation.

### P318 A phase II multicenter trial to evaluate efficacy and safety of HF10 oncolytic virus immunotherapy and ipilimumab in patients with unresectable or metastatic melanoma

#### Robert HI Andtbacka^1^, Merrick Ross^2^, Sanjiv Agarwala^3^, Kenneth Grossmann^1^, Matthew Taylor^4^, John Vetto^5^, Rogerio Neves^6^, Adil Daud^7^, Hung Khong^1^, Stephanie M Meek^8^, Richard Ungerleider^9^, Scott Welden^9^, Maki Tanaka^10^, Matthew Williams^11^

##### ^1^University of Utah, Huntsman Cancer Institute, Salt Lake City, UT, USA; ^2^Univesity of Texas MD Anderson Cancer Center, Houston, TX, USA; ^3^St. Luke's Hospital, Easton, PA, USA; ^4^Oregon Health & Science University, Portland, OR, USA; ^5^Knight Cancer Institute, Oregon Health and Science University, Portland, OR, USA; ^6^Pennsylvania State University, Hershey Cancer Institute, Hershey, PA, USA; ^7^UCSF Helen Diller Family Comprehensive Cancer Center, San Francisco, CA, USA; ^8^University of Utah School of Medicine, Salt Lake City, UT, USA; ^9^Theradex, Princeton, NJ, USA; ^10^Takara Bio, Inc., Otsu Shiga, Japan; ^11^University of Utah, Salt Lake City, UT, USA

###### **Correspondence:** Scott Welden (swelden@theradex.com)

**Background**

HF10, an attenuated, replication-competent mutant strain of herpes simplex virus type 1 (HSV1), is a promising new oncolytic viral immunotherapy. HF10 (intratumoral injection) shows activity in injected lesions and uninjected metastatic lesions. An ongoing phase II study in melanoma patients (pts) is assessing whether the combination of HF10 and the immune checkpoint inhibitor ipilimumab (ipi) enhances the antitumor effect of HF10.

**Methods**

Ipi naïve pts with stage IIIB, IIIC or IV unresectable melanoma were enrolled. HF10 was administered intratumorally into single or multiple tumors (1x10^7^ TCID_50_/mL, up to 5 mL/dose); 4 injections qwk; then up to 15 injections q3wk. Ipi was administered intravenously (3 mg/kg), q3wk for 4 doses. Tumor responses (irRC) were assessed at 12, 18, 24, 36, and 48wks. Best Overall Response Rate (BORR) was determined at 24wks. Serial peripheral blood and tumor biopsies were obtained and analyzed for changes in cytokines, immune profile and tumor microenvironment. Herein we present the safety, efficacy, and preliminary correlative study results.

**Results**

In total, 46 pts were enrolled, of which 20 % were stage IIIB, 43 % stage IIIC, and 37 % stage IV melanoma. Most HF10-related adverse events (AEs) were ≤ G2, similar to HF10 monotherapy. No DLTs were reported; 3 G4 AEs reported, all not treatment related. 30.4 % had G3 AEs. HF10-related G3 AEs (n = 3) were left groin pain, thromboembolic event, lymphedema, hypoglycemia, and diarrhea. Of 44 efficacy evaluable pts, preliminary BORR at 24 wks was 42 % and overall study BORR including those after 24 wks was 50 % (20 % CR, 30 % PR) with a disease control rate of 68 %. Of 15 evaluable stage IV pts, 8 (53 %) pts were responders. In 24 treatment naïve pts BORR was 58 % (21 % CR, 37 % PR) and in 20 pts who had failed ≥1 therapies, BORR was 40 % (20 % CR, 20 % PR). Preliminary serial peripheral blood analyses demonstrated in 75 % of responders a sustained ≥2 fold induction of the Th1 cytokines IFN-gamma and/or TNF-alpha compared to baseline at day 0. In contrast, 12 % of non-responders demonstrated similar induction. Future efforts will focus on comprehensive statistical analysis of T cell cytokine induction in response to treatment, correlated to disease outcome.

**Conclusions**

Combination HF10 and ipi treatment is safe and well tolerated, with promising responses in both treatment naïve and treatment failure pts. Peripheral blood Th1 cytokine upregulation may be a potential marker for response in HF10 + ipi treatment.

### P319 Phase II CALM extension study: intratumoral CAVATAK™ increases immune-cell infiltrates and up-regulates immune-checkpoint molecules in the microenvironment of lesions from advanced melanoma patients

#### Robert HI Andtbacka^1^, Brendan Curti^2^, Sigrun Hallmeyer^3^, Bernard Fox^4^, Zipei Feng^2^, Christopher Paustian^2^, Carlo Bifulco^4^, Mark Grose^6^, Darren Shafren^6^

##### ^1^University of Utah, Huntsman Cancer Institute, Salt Lake City, UT, USA; ^2^Providence Cancer Center, Portland, OR, USA; ^3^Oncology Specialists, Chicago, IL, USA; ^4^Robert W. Franz Cancer Research Center, Earle A. Chiles Research Institute, Providence Cancer Center, Portland, OR, USA; ^6^Viralytics Limited, Sydney, New South Wales, Australia

###### **Correspondence:** Darren Shafren (darren.shafren@viralytics.com)

**Background**

CAVATAK™, an oncolytic immunotherapy, is a bio-selected strain of Coxsackievirus A21. Intratumoral (IT) injection of CAVATAK can induce preferential tumor cell infection, cell lysis and enhancement of a systemic anti-tumor immune response. The phase II CALM study investigated the efficacy and safety of IT CAVATAK in 57 patients (pts) with advanced melanoma resulting in a confirmed ORR of 28.1 % and DRR (>6 mths) of 21.1 %. Presented is an extension study aimed at understanding the impact of CAVATAK on immune cell infiltrates and immune checkpoint molecules within the tumor-microenvironment (TME) of treated lesions from advanced melanoma pts referenced to tumor response.

**Methods**

In the CALM extension study a cohort of 13 advanced melanoma pts received up to 3 x 10^8^ TCID_50_ CAVATAK IT on study days 1, 3, 5, and 8 and then every three weeks for a further 6 injections. Sequential tumor biopsies of injected lesions (study days 1 and 8) from 9 pts were monitored for evidence of viral-induced changes to immune cell infiltrates and checkpoint molecules being referenced to tumor response.

**Results**

Of the 9 pts evaluable for tissue response assessment in this study, CAVATAK-treated lesions from 6 pts displayed disease control (CR, PR or SD), while injected lesions from 3 pts exhibited disease progression. Multi-spectral immunohistochemistry imaging revealed elevated levels of immune cell infiltrates within the TME of lesions displaying disease control (DC) compared to progressing lesions, in particular elevated levels of CD8+ cells and PD-L1+ cells. NanoString® RNA analysis of pre- and post-treatment biopsy samples identified significant increases in the levels of immune checkpoint molecules, including PD-L1, CTLA-4, IDO, TIM-3 and LAG-3 in lesions exhibiting DC compared to progressing lesions. A similar differential pattern was observed with respect to a number of immune modulation elements, including interferon-induced and viral RNA response genes. Of notable interest was the preferential up-regulation in DC lesions of CD122 (a component of the IL-2 receptor complex), which is postulated to be a potential prognostic marker for anti-tumor activity by anti-CTLA-4 blockade strategies. In addition, CAVATAK treatment initiated the reconstitution of immune cell infiltrates in a number of lesions from pts failing previous immune-checkpoint blockade or other immunotherapies.

**Conclusions**

The changes in TME induced by CAVATAK support combination therapy with T cell checkpoints, especially anti-CTLA-4. There is an ongoing phase Ib study of CAVATAK + ipilimumab showing higher ORR than anticipated with either agent alone supporting the continued study of the combination.

### P320 A fully serotype 3 oncolytic adenovirus coding for CD40L as an enabler of dendritic cell therapy

#### Sadia Zafar^1^, Suvi Parviainen^1^, Mikko Siurala^1^, Otto Hemminki^1^, Riikka Havunen^1^, Siri Tähtinen^1^, Simona Bramante^1^, Lotta Vassilev^1^, Hongjie Wang^3^, Andre Lieber^3^, Silvio Hemmi^4^, Tanja de Gruijl^5^, Anna Kanerva^1^, Akseli Hemminki^1^

##### ^1^University of Helsinki, Helsinki, Uusimaa, Finland; ^3^University of Washington, Seattle, WA, USA; ^4^University of Zurich, Zurich, Switzerland; ^5^VU University Medical Center, Amsterdam, Netherlands

###### **Correspondence:** Sadia Zafar (sadia.zafar@helsinki.fi)

**Background**

Dendritic cell (DC) therapy is currently considered as a promising cancer immunotherapy. Dendritic cells are considered as principal initiators of the immune system. However, tumor induced immunosuppression impairs the biological function of DCs. Therefore, clinical outcomes with DC therapy have often been disappointing. Interestingly, oncolytic adenoviruses have good safety profile in humans. They have been shown to activate immune responses by triggering danger signals at the tumor site and enhancing the release of tumor-specific antigens.

**Methods**

To achieve optimal activation of the transferred dendritic cells, we armed adenoviruses with CD40 ligand (CD40L), best known for its capacity to initiate multifaceted signals in dendritic cells, leading to the activation of cytotoxic T cells. Therefore, we constructed a novel virus Ad3-hTERT-CMV-hCD40L which features Ad3 for enhanced tumor transduction, human telomerase reverse transcriptase (hTERT) promoter for enhancing tumor selectivity and CD40L, a potent stimulator of dendritic cells and to increase antitumor efficacy. The viral particles are produced in 293 cells using a standard calcium phosphate method. Then, HeLa cells were infected with the cell lysate containing Ad3-GFP virus for further virus propagation. The functionality of the viruses is studied by infecting different cell lines with different amount of viral particles and measuring the proportion of surviving cells with MTS assay. To deeply dissect if CD40L encoding adenovirus can modulate the tumor microenvironment, we generated a murine version of the virus (Ad5/3-CMV-mCD40L).

**Results**

The major obstacle with oncolytic adenoviruses is suboptimal systemic delivery, which is circumvented by using a fully Ad3 platform. As human [1] and our animal data have shown, the ability of Ad3 to successfully reach tumors is through the intravenous route. In syngeneic studies in immunocompetent model, DC therapy in combination with Ad5/3-CMV-mCD40L showed potent antitumor activity and triggered significant antitumor immune responses. The improved therapeutic effect by the adenovirus expressing CD40L and DCs combination treatment correlated with increased numbers of tumor infiltrating lymphocytes, induction of the T helper type 1 cytokines IFN-gamma, RANTES, and TNF-alpha and the reduction of immunosuppression in the tumor stroma.

**Conclusions**

Our findings support the development of clinical trials where dendritic cell therapy is enhanced with oncolytic adenovirus.

**References**

1. Hemminki O, Diaconu I, Cerullo V, *et al.*: **Ad3-hTERT-E1A, a Fully Serotype 3 oncolytic adenovirus, in patients with chemotherapy refractory cancer.** *Mol Ther* 2012, **20**:1821–1830.

**Promoting and Measuring Anti-Tumor Immunity**

### P321 A multi-color natural killer-cell mediated cytotoxicity detection using fluorescence and direct cell imaging

#### Tameem Ansari, Srividya Sundararaman, Diana Roen, Paul Lehmann

##### Cellular Technology Ltd, Shaker Hts, OH, USA

###### **Correspondence:** Tameem Ansari (tameem.ansari@immunospot.com)

**Background**

The most essential role of effector immune cells such as CD8+ cells and natural killer (NK) cells is to identify and lyse target cells. NK cell - and antibody dependent cell cytotoxicity (ADCC) - has traditionally been assessed by the release of radioactive chromium from target cells following lysis. These assays are laborious and require substantial quantities of patient blood to detect minor changes in cell lysis. We have previously developed an assay that can visualize individual target cells to detect cytolytic activity within a high signal to noise range, without involving radioactivity, via high-throughput imaging. In order to further reduce the amount of cell material required and detect the effect of NK cells on different target cell lines, we have now developed a multi-color cytotoxicity detection assay.

**Methods**

The assay we developed images individual fluorescence-labeled target cells. K562, A549 and T2 tumor cells were used as targets, and peripheral blood mononuclear cells (PBMC) as effector cells. When performing the assay in 96 well format, the PBMC were plated in serial dilution between 500,000 and 7,500 cells per well with 5,000 target cells per well. Four hours later, the number of viable tumor cells was quantitated using a fluorescence capable ImmunoSpot® Analyzer or the radioactivity released was measured. For multi-color analysis, we stained three different cancer cell lines (one of which had intact MHC receptors) with three different dyes and incubated them in the same well with effector to target ratios that match one cell line per well.

**Results**

The target cell visualization and chromium release assay in a 96-well format required the same number of cells and the results were comparable to each other. While, expectedly, percentage of killing for different donors was highly variable, the assay was highly reproducible for cryopreserved samples between multiple days and when performed by multiple researchers. The results obtained via multi-color assessment show that we can simultaneously detect the cytolytic effect of NK cells on three different target cell types using only a third of the effector cells as previously required. Also, the data show that control target cells with MHC receptors are not susceptible to NK killing.

**Conclusions**

We have demonstrated the feasibility of assessing NK function in a non-radioactive, high-throughput capable system which will benefit clinical immune monitoring. The multi-color analysis should be of particular value when access to PBMC is limited, such as in pediatric, geriatric, and immune deficient populations.

### P322 Intratumoral injection of INT230-6 induces protective T cell immunity

#### Anja C Bloom^1^, Lewis H Bender^2^, Ian B Walters^2^, Masaki Terabe^1^, Jay A Berzofsky^1^

##### ^1^National Cancer Institute, Bethesda, MD, USA; ^2^Intensity Therapeutics, Inc., Westport, CT, USA

###### **Correspondence:** Anja C Bloom (anja.bloom@nih.gov)

**Background**

Standard care for many types of cancer involves systemic administration of cytotoxic agents. This may result in low drug concentration at tumor sites, which limits cell killing. More recently it has been shown that cytotoxic formulations designed for intratumoral delivery improve drug efficacy presumably by increasing drug concentration at the tumor site. Furthermore, it has been revealed that the mechanisms of anticancer agents extend beyond direct tumor cell lysis. One major aspect is that cell death often induces an immune response. Different types of cell death such as necrosis and autophagy induced by cytotoxic agents trigger immune responses with varying degrees of inflammation and involving different types of immune cells. The ideal immune responses that may give maximum benefit to patients would be strong and long lasting anti-tumor T cell responses.

**Methods**

In this study, a novel tissue and cell diffusive cytotoxic formulation, INT230-6, was administered intratumorally over 5 sequential days into subcutaneous 300 mm^3^ murine Colon26 tumors.

**Results**

Treatment resulted in regression from baseline of 100 % of the tumors and up to 80 % complete response (CR). We then sought to analyze the T cell responses in the protection induced by INT230-6. Mice with CR were protected from re-challenge either by subcutaneous or intravenous re-inoculation of the Colon26. The protection was abrogated by CD4/CD8 double depletion prior to the re-challenge, indicating that immunological memory was induced. Colon26 tumors express the endogenous retroviral protein gp70 containing the AH-1 CTL epitope. AH-1-specific CD8+ T cells were detected *ex vivo* in systemic organs such as spleens and peripheral blood of a subset of mice with CR, confirming induction of CD8+ T cell specific responses to tumor cells upon INT230-6 treatment.

**Conclusions**

Hence, INT230-6 given locally to treat tumors induces tumor specific protective T cell immunity.

### P323 Fluorine-19 nuclear magnetic resonance (NMR) to track and quantify human transgenic T cell biodistribution in murine studies of glioblastoma immunotherapy

#### Fanny Chapelin^1^, Hideho Okada^2^, Eric T Ahrens^1^

##### ^1^University of California, San Diego, La Jolla, CA, USA; ^2^University of California, San Francisco, San Francisco, CA, USA

###### **Correspondence:** Fanny Chapelin (chapelinfanny@hotmail.com)

**Background**

Glioblastoma multiforme (GBM) is the most common brain cancer for which classical treatment options remain limited. Recent advances in immunotherapy for other cancers hold great promise for the treatment of GBM. To uncover the mechanisms of such therapies, it is critical to develop tools to quantitatively assay T cell biodistribution and survival after delivery to correlate with putative therapeutic effects. In this study, we used a new probe technology to quantify T cell therapy distribution in intact tissue samples and correlated the results to tumor growth.

**Methods**

Human PBMC-isolated T cells were transduced with a chimeric antigen receptor (CAR) lentiviral vector to express a surface antibody against EGFRvIII, a common receptor in GBM. We compared CAR T cells efficacy and biodistribution to those of naïve T cells. CAR-T cells and naïve T cells were intracellularly labeled with a perfluorocarbon (PFC) emulsion *ex vivo* and injected intravenously in SCID mice bearing bilateral subcutaneous human U87 tumors engineered to express EGFRvIII. Tumor growth was monitored over 7 days with bioluminescence imaging and caliper measurements. Intact organs were then harvested for fluorine-19 NMR measurements to quantify the fluorine content and apparent cell count, followed by processing for histological validation.

**Results**

**Fig. 41 (abstract P323).**
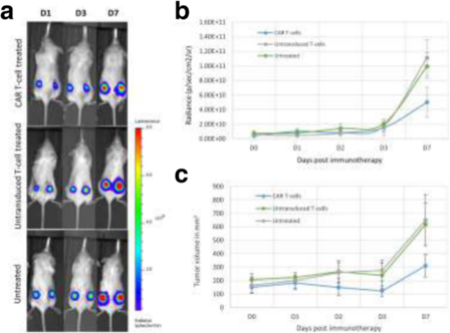
Immunotherapy impact on tumor growth in vivo. **a** Representative BLI images at day 1, 3 and 7 after immunotherapy for all 3 groups. Signals are expressed in radiance (p/sec/cm2/sr) **b** Time course of bioluminescence intensity in treated and untreated groups. CAR-T cell treated animals display a radiance half as high as untransduced-T cell treated animals or untreated animals. C. Corresponding tumor volumes show 50% reduction in tumor growth for the CAR-T cell treated group (p=0.0001) and no significant difference between untransduced T cell treated and untreated groups (p=0.38)

**Fig. 42 (abstract P323).**
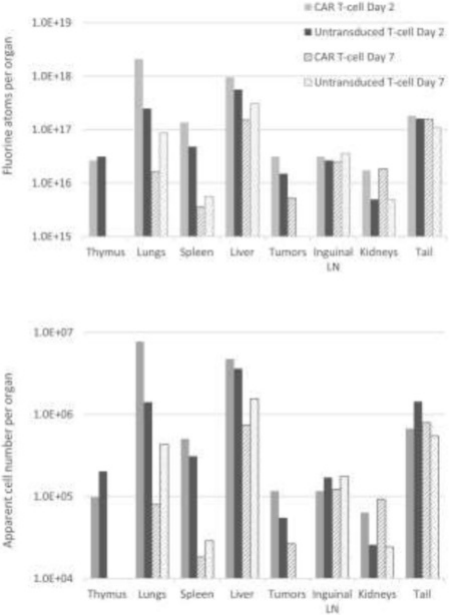
Biodistribution quantification of fixed tissue samples by 19F NMR at 2 days and 7 days post treatment. 19F NMR measurements of organ biodistribution of the PFC-labeled CAR-T cells and untransduced T cells show strong liver, lungs, lymph node, and tail signal but modest signal in the spleen and tumors in both groups. Data is presented as the average fluorine atom content per organ (top) and the corresponding apparent cell number (bottom). These measurements do not account for cell division, thus referred to as “apparent cell number”. The tail signal represents mis-injection

Longitudinal bioluminescence acquisitions and tumor measurements showed considerable tumor regression 7 days after CAR treatment with a radiance average of 5.02 × 10^10^ photons/sec/cm^2^/sr, which was twice lower than the luminescence measured for both naïve T cell treated and untreated groups (p = 0.0001, Fig. 41). NMR measurements in whole organs at days 2 and 7 showed strong signal in the liver, lungs, lymph nodes and tail (injection site) but modest signal in the spleen and tumors in both groups (Fig. 42). We were able to detect as low as 20,000 CAR T cells homing to the tumors but did not find any naïve T cells in the tumors. On average, the liver in naïve T cell recipients had twice the fluorine signal compared to CAR T cells. The liver signals likely represent the dead T cell fraction. We were also able to visualize failure of therapy delivery in a few animals where most of the cells remained in the lungs days after therapy injection.

**Conclusions**

To conclude, ^19^F NMR analysis, in conjunction with bioluminescence imaging, may accelerate the timeline to evaluate new immunotherapeutic cell candidates by providing a rapid and straight-forward method to determine therapy efficacy, cell biodistribution and fate in preclinical studies. We are currently performing *in vivo* ^19^F MRI studies using the PFC technology.

### P324 Immune profiling of an elite responder following checkpoint inhibitor therapy reveals functional anti-tumor antibodies within expanded IgG lineages

#### Jeff DeFalco^1^, Michael Harbell^1^, Amy Manning-Bog^1^, Alexander Scholz^1^, Danhui Zhang^1^, Gilson Baia^1^, Yann Chong Tan^1^, Jeremy Sokolove^2^, Dongkyoon Kim^1^, Kevin Williamson^1^, Xiaomu Chen^1^, Jillian Colrain^1^, Gregg Espiritu Santo^1^, Ngan Nguyen^1^, Wayne Volkmuth^1^, Norman Greenberg^1^, William Robinson^2^, Daniel Emerling^1^

##### ^1^Atreca, Redwood City, CA, USA; ^2^Stanford University School of Medicine, Stanford, CA, USA

###### **Correspondence:** Norman Greenberg (ngreenberg@atreca.com)

**Background**

Anti-tumor antibodies can contribute to effective patient immune responses to cancer, yet we do not fully understand their characteristics or mechanisms of action. To better elucidate the nature and significance of such antibodies we sequenced over 2500 blood plasmablasts (activated B cells) of a patient (DID-08291) with stage IV melanoma during a period of stable disease and characterized activities of resultant monoclonal antibodies.

**Methods**

Patient plasmablasts were collected and antibody sequences obtained from single cells using Atreca’s Immune Repertoire Capture™ (IRC™) technology. IRC™ incorporates complex DNA barcodes with reverse transcription, PCR and next generation sequencing to provide nearly error-free, full-length variable regions of natively paired immunoglobulin heavy and light chain genes. Sequences obtained through IRC™ can subsequently be used to synthesize and express recombinant antibodies for *in vitro* and *in vivo* testing.

**Results**

The IRC™ analysis of two sequential samples, collected 3 months apart while the patient was undergoing ipilumumab therapy, revealed extensive diversity of germline gene usage, CDR lengths, and levels of somatic hypermutation (SHM) across individual B cells. Over 1400 putative clonal antibody families were identified by grouping sequences based on immunoglobulin gene usage and other sequence features. Of these families, over 400 showed evidence of clonal expansion and/or were observed at both blood collection time points. Full length natively paired variable regions were subsequently expressed from IRC™ sequences representing both large and small putative families to generate recombinant antibodies. Multiple antibodies were found to exhibit binding to the surface of cancer cells and were further characterized for their ability to mediate *in vitro* cancer cell killing through various mechanisms including ADCC, ADC, and/or ADCP. Protein arrays are being used to identify the targets of these antibodies, while tumor growth inhibition/regression studies in syngeneic mouse models are underway to better understand the antibodies therapeutic capabilities when delivered alone or in combination with other immunotherapies.

**Conclusions**

These results illustrate that naturally occurring patient antibodies have anti-tumor activity and support their further development as novel immunotherapeutics.

### P325 Cytokine profile of sipuleucel-T in differentiating reactivation of latent immunity from de novo immune responses

#### Charles G Drake^1^, Daniel P Petrylak^2^, Emmanuel S Antonarakis^1^, Adam S Kibel^3^, Nancy N Chang^4^, Tuyen Vu^4^, Dwayne Campogan^4^, Heather Haynes^4^, James B Trager^4^, Nadeem A Sheikh^4^, David I Quinn^5^

##### ^1^Johns Hopkins Sidney Kimmel Comprehensive Cancer Center, Baltimore, MD, USA; ^2^Yale Cancer Center, New Haven, CT, USA; ^3^Urologic Surgery, Brigham and Women’s Hospital, Harvard University, Boston, MA, USA; ^4^Dendreon Pharmaceuticals, Inc., Seattle, WA, USA; ^5^Norris Comprehensive Cancer Center, University of Southern California, Los Angeles, CA, USA

###### **Correspondence:** Charles G Drake (cdrake@jhmi.edu)

**Background**

Sipuleucel-T is an autologous cellular immunotherapy approved by the FDA for treatment of asymptomatic or minimally symptomatic metastatic castration-resistant prostate cancer (mCRPC)[1]; it is manufactured from autologous peripheral blood mononuclear cells (PBMCs) cultured with the immunogen PA2024, a fusion of prostatic acid phosphatase (PAP) conjugated to granulocyte macrophage colony-stimulating factor. Antibody and T cell responses to PA2024 and/or PAP correlate with improved overall survival in sipuleucel-T–treated mCRPC patients [2]. To better understand sipuleucel-T–induced T cell responses, we assessed CD4+ and CD8+ T cells for proliferation, intracellular cytokine production, and cytokine release after PA2024 or PAP stimulation.

**Methods**

PBMCs were obtained from sipuleucel-T–treated mCRPC patients (STRIDE) and nonmetastatic, biochemically recurrent, hormone-sensitive PC patients (STAND). Samples were collected at baseline through month 6 post–sipuleucel-T. PBMCs were cultured in vitro and stimulated with either PA2024 or PAP. CD4+ and CD8+ T cells were assessed (n=19) for proliferation and for intracellular IL-2 and IFN-γ. The cytokine profile was confirmed in supernatant with a meso scale discovery assay. p < 0.10 was statistically significant.

**Results**

Compared with baseline, PA2024-specific proliferating CD4+ and CD8+ T cells had increased intracellular IL-2 and IFN-γ at week 6 and month 6 with a similar trend for PAP-specific proliferating T cells (Table 4). Compared with unstimulated controls, a significant >2-fold increase in PA2024-stimulated IL-2 and IFN-γ in supernatant was observed at wk 6 and mo 6 over baseline (p < 0.001). PAP-stimulated IL-2 and IFN-γ supernatant levels increased over baseline and was significantly elevated for IFN-γ at wk 6 (p < 0.10).

**Conclusions**

Sipuleucel-T therapy generated a de novo PA2024-specific T cell response, as indicated by the cytokine release profile. The PAP-stimulated cytokine profile suggests that pre-existing immunity with terminally differentiated T cells are expanded. Thus, sipuleucel-T reactivated an anti-PAP response in memory T cells, thereby overcoming immunosuppressive mechanisms in PC.

**Acknowledgements**

Medical writing services provided by Brian R. Haas, PhD, were funded by Dendreon Pharmaceuticals, Inc.

**Trial Registration**

NCT01431391 and NCT01981122.

**References**

1. **PROVENGE® (sipuleucel-T) prescribing information** [Internet]. Seattle: Dendreon Corporation; c2014 [last revision 2014 October; cited 2016 April 21]. Available from: http://www.valeant.com/Portals/25/Pdf/PI/Provenge-PI.pdf. Accessed 21 April 2016.

2. Sheikh, *et al.*: *Cancer Immunol Immunother* 2013, **62**:137–147.

**Table 4 (abstract P325).** Elevation in intracellular cytokine levels

		CD4 T cell		CD8 T cell	
		Wk 6	Mo 6	Wk 6	Mo 6
PA2024	IL-2	p < 0.001	p < 0.001	p < 0.05	p < 0.01
	IFN-γ	p < 0.001	p < 0.05	p < 0.10	p < 0.05
PAP	IL-2	p < 0.001	ns	p < 0.05	ns
	IFN-γ	p < 0.05	ns	p<0.05	ns

ns = not significant

### P326 NKTR-255: an IL-15-based therapeutic with optimized biological activity and anti-tumor efficacy

#### Peter Kirk^1^, Murali Addepalli^1^, Thomas Chang^1^, Ping Zhang^1^, Marina Konakova^1^, Katsunobu Hagihara^2^, Steven Pai^3^, Laurie VanderVeen^1^, Palakshi Obalapur^1^, Peiwen Kuo^1^, Phi Quach^1^, Lawrence Fong^3^, Deborah H Charych^1^, Jonathan Zalevsky^1^

##### ^1^Nektar Therapeutics, San Francisco, CA, USA; ^2^University of California, San Francisco School of Medicine, San Francisco, CA, USA; ^3^Hellen Diller Family Comprehensive Cancer Center, San Francisco, CA, USA

###### **Correspondence:** Peter Kirk (pkirk@nektar.com)

**Background**

Interleukin-15 has been identified as a promising candidate for use as an immuno-oncology therapeutic, but the native cytokine has poor drug-like properties. NKTR-255 is a novel immunotherapeutic agent consisting of polymer-engineered IL-15 designed to optimally engage the IL-15 receptor complex and provide durable pathway activation *in vivo*. Here we show that NKTR-255 has greatly improved plasma and tumor exposure relative to IL-15, induces NK and CD8+ T cell activation and proliferation, and has single-agent efficacy in syngeneic tumor models.

**Methods**

Binding kinetics and affinity of NKTR-255 for IL-15Rα were measured by surface plasmon resonance using immobilized IL-15Rα. Cell-based potency was determined by treating CTLL-2 cells with NKTR-255 at a range of concentrations and measuring phosphorylation of STAT5 in cell lysate by immunoassay. Pharmacokinetic analysis was performed following single-dose intra-venous administration of IL-15 and NKTR-255 in normal mice and in mice bearing sub-cutaneous B16F10 and CT-26 tumors, with analytes quantified in tumor and plasma by ELISA. Immunophenotyping studies were performed by flow cytometry on lymphocytes from peripheral blood of NKTR-255-treated and IL-15-treated normal mice and from spleen, tumor and draining lymph node of treated mice carrying sub-cutaneous TRAMP-C2 tumors. Efficacy was determined by measuring tumor volume of sub-cutaneous TRAMP-C2 and CT-26, with q5dx3 treatment with NKTR-255.

**Results**

NKTR-255 binds to IL-15Rα, and induces STAT5 phosphorylation in CTLL-2 cells with sub-nanomolar EC_50_. Following intravenous administration, NKTR-255 demonstrates a greatly reduced clearance rate compared to IL-15, with plasma t_1/2_ of 22-26 h versus <1 h for IL-15. Tumor exposure of NKTR-255 was 50-fold greater than IL-15 in B16F10-bearing C57/Bl6 mice and 110-fold greater in CT-26-bearing Balb/c mice. Immunophenotyping studies in normal mice showed an induction of Ki-67 and CD122 expression in NK cells, indicating proliferation and activation. In tumor-bearing mice, NKTR-255 treatment resulted in an increased CD8:CD4 and CD8:Treg ratio in tumor and spleen, and an increased frequency of CD8^+^TNF^+^IFNγ^+^ T cells. Tumor growth inhibition was observed in both CT-26 and TRAMP-C2 models.

**Conclusions**

NKTR-255 treatment results in sustained IL-15 activity which induces CD8+ T cell and NK cell activation and proliferation, and produces long-lived immunophenotypic changes in tumor-bearing mice. The design of NKTR-255 enables a potential drug-like therapeutic strategy for accessing IL-15-based immunotherapy.

### P327 Anti-tumor activity of NKTR-214; a CD122-biased agonist that promotes immune cell activation in the tumor microenvironment and lymphoid tissues

#### John L Langowski, Murali Addepalli, Yolanda Kirksey, Ravi Nutakki, Shalini Kolarkar, Rhoneil Pena, Ute Hoch, Jonathan Zalevsky, Stephen K Doberstein, Deborah H Charych

##### Nektar Therapeutics, San Francisco, CA, USA

###### **Correspondence:** John L Langowski (jlangowski@nektar.com)

**Background**

NKTR-214 is a novel agonist of the IL-2 pathway that provides a sustained and biased activation signal to the heterodimeric IL-2R complex (IL-2Rbg). Here we examine its biological activity, pharmacodynamic effects and mechanism of action in a murine tumor model.

**Methods**

To determine efficacy, mice bearing established subcutaneous B16F10 melanoma tumors were treated with NKTR-214 (q9dx3) or aldesleukin (qdx5, two cycles). For mechanistic studies, mice were treated once with NKTR-214 or with five daily administrations of aldesleukin. Splenic or tumor-infiltrating lymphocytes (TIL) were assessed by flow cytometry and gene expression analysis was conducted by RNA-Seq 5, 7, and 10 days after treatment initiation. To assess the relative contribution of tumor-resident or migrating lymphocytes to efficacy, the sphingosine-1-phosphate receptor modulator FTY720 was administered daily alone or in combination with NKTR-214.

**Results**

In the aggressive B16F10 model, vehicle-treated tumors grew to the volume endpoint 8 days after initiation, with a tumor volume quadrupling time (TVQT) of 5 days. NKTR-214 showed better efficacy than aldesleukin (TVQT 16.7 versus 10 days). FTY720 significantly decreased blood lymphocytes and when added to treatment, efficacy with NKTR-214 was reduced by 39 % but not completely abrogated. Analysis of TIL demonstrated that both NKTR-214 and aldesleukin led to an increase in activated NK cells. However, NKTR-214 administration led to significant and sustained increases in total and memory CD8+ T cells, while the effects from aldesleukin were transient. NKTR-214 also reduced the percentage of intratumoral Tregs at every time point, while aldesleukin had little effect on this parameter. Consequently, NKTR-214 increased the average CD8+ T cell/Treg ratio to >400, which surpassed that achieved by aldesleukin. Immune cell changes in the spleen followed a similar pattern, however with a lesser magnitude. In addition to changes in cell number, NKTR-214 treatment also induced modulation of immune gene expression networks directly in the tumor microenvironment.

**Conclusions**

Efficacy generated by the sustained and biased signaling of the IL-2 pathway with NKTR-214 cannot be achieved even with multiple daily administrations of aldesleukin. Furthermore, the profound changes in tumor-infiltrating lymphocytes associated with the anti-tumor activity of NKTR-214 arise from T cells stimulated in both the tumor microenvironment and the lymphoid tissues. NKTR-214 is currently being evaluated in a in an ongoing single-agent phase I/II clinical trial to assess safety, efficacy, pharmacokinetics and immune changes in the tumor microenvironment.

### P328 Nanosecond pulsed electric field treatment of murine melanomas initiates an immune response and inhibits metastasis

#### John Cha, Zach Mallon, Myra Perez, Amanda McDaniel, Snjezana Anand, Darrin Uecker, Richard Nuccitelli

##### Pulse Biosciences, Burlingame, CA, USA

###### **Correspondence:** Richard Nuccitelli (rnuccitelli@pulsebiosciences.com)

**Background**

Nano-Pulse Electro-Signaling (NPES) is a non-thermal, localized application of ultrashort electrical pulses in the nanosecond range that can trigger immunogenic cell death in treated tumors. We have demonstrated previously that the application of 2000 pulses 100 ns long and, 30 kV/cm in amplitude completely ablates the treated tumor within 3 weeks via apoptosis and initiates an immune response that inhibits secondary tumor growth [1]. We wanted to determine if this primary tumor treatment also inhibits metastasis by injecting live tumor cells into the tail vein and counting the number of lung metastases 3 weeks later.

**Methods**

14 female B6/J albino mice were given intradermal injections of 500,000 B16-GFP cells in 15uL HBSS. Upon reaching 5 mm in their largest dimension as visualized by epifluorescence, 6 mice had their tumors resected surgically, and the tumors in 8 mice were treated with 2000 pulses of 100 ns and 53 kV/cm. Four weeks after resection or NPES treatment, both the six surgically resected mice and four NPES-treated mice were injected with 200,000 B16-GFP cells into the lateral tail vein. 4 NPES treated mice were not challenged as negative controls. Lung metastases were counted 3 weeks later by epifluorescence imaging.

**Results**

Three weeks after intravenous injection with 200,000 B16-GFP melanoma cells, mice with surgical resection of the primary tumor averaged 17 lung metastases/mouse. Mice with NPES ablation of the primary tumor averaged 3.3 lung metastases/mouse from intravenous challenge. Mice with NPES ablation of the primary tumor and no challenge exhibited no lung metastases.

**Conclusions**

Immunogenic cell death caused by NPES treatment of primary tumors stimulates anti-tumor immunity to a subsequent challenge with intravenous B16-GFP cells, extending the vaccination effect beyond solid secondary malignancies to circulating cancer cells.

**References**

1. Nuccitelli R, Tran K, Lui K, Huynh J, Athos B, Kreis M, Nuccitelli P, De Fabo EC: **Non-thermal nanoelectroablation of UV-Induced murine melanomas stimulates an immune response**. *Pigment Cell Melanoma Res* 2012, **25**:618–629.

**Fig. 43 (abstract P328).**
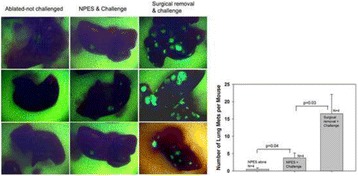
NPES treatment of primary tumor inhibits lung metastases. B16-GFP cell metastasis is greatly inhibited in mice whose primary tumor was treated with NPES

### P329 Nanosecond pulsed electric field treatment of tumor cell lines triggers immunogenic cell death (ICD)

#### Amanda McDaniel, Snjezana Anand, John Cha, Darrin Uecker, Richard Nuccitelli

##### Pulse Biosciences, Burlingame, CA, USA

###### **Correspondence:** Richard Nuccitelli (rnuccitelli@pulsebiosciences.com)

**Background**

Nano-Pulse Electro-Signaling (NPES) is a non-thermal, localized application of ultrashort electrical pulses in the nanosecond range that can trigger immunogenic cell death in treated tumors. We have demonstrated previously that the application of 400 pulses 100 ns long and, 30 kV/cm in amplitude completely ablates treated orthotopic rat liver tumors within 2 weeks via apoptosis and initiates an immune response that inhibits secondary tumor growth in a CD8-dependent manner [1]. Here we determine if NPES treatment results in the expression of three damage-associated molecular patterns (DAMPs) that play significant roles in immune signaling.

**Methods**

We treated three separate cancer cell lines (MCA205, McA-RH7777, Jurkat E6-1) with NPES. One million cells were suspended in 800 μl media and treated in a 4 mm electroporation cuvette. Five total treatments were delivered ranging in energy from 5–50 J/mL. The pulse parameters were fixed (15 kV/cm, 100 ns, 2 pps) and energy delivery was controlled by varying the pulse number. 500,000 cells from each treatment group and untreated cells were seeded into a 24-well plate and incubated at 37 °C for 24-hours. Cell culture supernatants were collected to measure levels of HMGB1 and ATP. Cells were also harvested and the expression levels of cell surface calreticulin were determined using flow cytometry.

**Results**

NPES induced all three markers of ICD in an energy-dependent manner. HMGB1 release and calreticulin expression increased with treatment energy in all three cell lines. Extracellular ATP followed a different pattern, showing a bell-shaped response that peaked at 15 J/mL followed by a drop off at 25 J/mL in both the MCA-205 and McA-RH7777 cell lines. ATP levels in the Jurkat cells remained low across all conditions.

**Conclusions**

We have demonstrated that three key markers of ICD can be induced by treating tumor cells with NPES. This can explain why we see a vaccine-like effect after *in vivo* NPES treatment, inhibiting secondary tumor growth after subsequent challenges with tumor cells.

**References**

1. Nuccitelli R, Berridge JC, Mallon Z, Kreis M, Athos B, Nuccitelli P: **Nanoelectroablation of murine tumors triggers a CD8-dependent inhibition of secondary tumor growth**. *PLoS One* 2015, **10**: e0134364.

**Fig. 44 (abstract P329).**
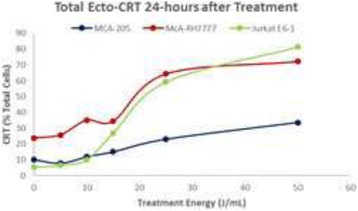
Ecto-Calreticulin 24 h. Ecto-calreticulin on NPES-treated cells 24 h after treatment

**Fig. 45 (abstract P329).**
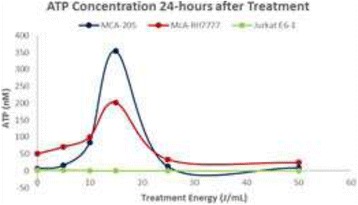
ATP secreted at 24 h. ATP concentration outside cells 24 h after treatment

**Fig. 46 (abstract P329).**
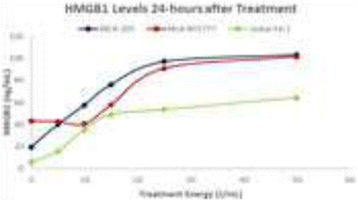
HMGB1 secretion 24 h after treatment. HMGB1 is secreted 24 h post treatment at all energies

### P330 Monitoring the changes in tumor-specific TILs during immunotherapy

#### Nataša Obermajer^1^, Julie Urban^2^, Eva Wieckowski^2^, Ravikumar Muthuswamy^2^, Roshni Ravindranathan^2^, David Bartlett^1^, Pawel Kalinski^3^

##### ^1^Department of Surgery, University of Pittsburgh, Pittsburgh, PA, USA; ^2^University of Pittsburgh, Pittsburgh, PA, USA; ^3^Department of Surgery; University of Pittsburgh Cancer Institute; Department of Infectious Diseases and Microbiology, University of Pittsburgh, Pittsburgh, PA, USA

###### **Correspondence:** Nataša Obermajer (obermajern2@upmc.edu)

**Background**

The development of novel immunotherapeutic approaches need to consider two critical aspects of anti-tumor immunity: generation of high-magnitude effector and memory T cell responses (i.e. cytotoxic CD8+ T, CTLs) and the means to facilitate effective infiltration of CTLs into the tumor microenvironment.

**Methods**

Here we use a novel protocol of evaluating the changing numbers of tumor-specific T cells within tumors of mice receiving different forms of immunotherapy, and strategies to increase numbers of specific CTLs in murine tumors.

**Results**

We report separate requirements for the induction of tumor-specific T cells in the spleen and lymph nodes versus the tumor tissues in the course of combinatorial immunotherapies involving a specialized dendritic cell (DC) vaccine, with augmented ability to enhance systemic numbers of tumor-specific effector CTLs, and the combinatorial strategy to promote the homing of the vaccination-induced CTLs to tumors.

**Conclusions**

In contrast to commonly used tumor models involving highly-immunogenic model antigens, our approach allows for the assessment of local immune responses to more clinically relevant, weakly-immunogenic non-manipulated cancers, facilitating the development and preclinical evaluation of new immunotherapies.

### P331 Bortezomib enhances expression of effector molecules in anti-tumor CD8+ lymphocytes by modulating Notch-NFκB-miR-155 crosstalk

#### Ariana N. Renrick^1^, Menaka Thounaojam^2^, Portia Thomas^1^, Samuel Pellom^1^, Anil Shanker^3^

##### ^1^Meharry Medical College, Nashville, TN, USA; ^2^Medical College of Georgia, Augusta, GA, USA; ^3^Meharry Medical College School of Medicine, Nashville, TN, USA

###### **Correspondence:** Ariana N. Renrick (arenrick14@email.mmc.edu)

**Background**

The immunosuppressive tumor microenvironment disturbs immune regulatory networks and takes over host antitumor immune responses. We have previously reported that tumor interferes with host hematopoietic Notch system, which could result in the inadequate induction of antitumor immunity. Interestingly, we found that tumor-induced decrease in immune Notch could be restored by the FDA-approved proteasome inhibitor bortezomib, which also sensitizes tumors to death signals. We are also elucidating components of microRNA regulation affecting NICD–NFκB crosstalk.

**Methods**

WT Balb/c mice (Harlan) will be used in four different groups with 3 mice per group. The treatment groups are as follows: saline alone, bortezomib alone, tumor (4 T1 breast tumor cells 2x10^6^) alone, or tumor + bortezomib administration. Tumor-bearing mice will be injected sub-cutaneously with the tumor cells. We will then allow for the solid tumor to establish in the mice, which takes approximately 14 days. The tumors should be approximately 125 mm^3^. After the establishment of tumor, the mice will be injected with 1 mg/kg body weight of bortezomib intra-veneously. After 4 hours, the mice will be sacrificed and the spleen and lymph nodes will be harvested and CD8^+^ T cells will be purified. Analysis of T cell activation markers, Notch signaling markers, T cell effector molecules and miR-155 expression will be analyzed by flow cytometry and RT-qPCR.

**Results**

Bortezomib administration modulates Notch system in CD8+ T cells isolated from tumor-bearing mice. Bortezomib improves cytolytic T lymphocyte function. Bortezomib abrogates the negative effect of g-secretase inhibitor (GSI) on T cell effector molecules. Bortezomib abrogates GSI effect and stimulates Notch genes via NICD cleavage and/or NFkB activation. Bortezomib intersects with both canonical (NICD) and non-canonical (NFkB) pathway. Bortezomib upregulates miR-155 expression in the presence of tumor. miR-155 expression is suppressed in T cells when Notch signaling is inhibited by GSI. Notch target gene expression is suppressed when miR-155 is inhibited.

**Conclusions**

Bortezomib modulates Notch signaling at both the Notch receptor and target gene level, thereby improving the expression of T cell effector molecules in tumor-bearing mice. Bortezomib presents the opportunity to sensitize tumors to cell death, while simultaneously improving CD8+ T cell function via NICD and NFkB activation leading to effective antitumor immune function. Bortezomib has the ability to increase miR-155 expression even when Notch signaling is blocked by GSI, suggesting an interplay between Notch and miR-155 affecting expression of T cell effector molecules.

### P332 Bortezomib improves adoptive T cell immunotherapy in solid tumors

#### Samuel Pellom^1^, Menaka Thounaojam^2^, Duafalia Dudimah^3^, Alan Brooks^4^, Thomas J. Sayers^5^, Anil Shanker^3^

##### ^1^Meharry Medical College, Nashville, TN, USA; ^2^Medical College of Georgia, Augusta, GA, USA; ^3^Meharry Medical College School of Medicine, Nashville, TN, USA; ^4^National Cancer Institute-Frederick, Frederick, MD, USA; ^5^CIP, Center for Cancer Research, BSP, Leidos Biomed Research Inc, National Cancer Institute-Frederick, Frederick, MD, USA

###### **Correspondence:** Anil Shanker (ashanker@mmc.edu)

**Background**

Tumor-induced immune suppression is a hallmark feature of tumor growth, which is responsible for the blockade of host antitumor immunity and poor efficacy of anti-cancer immunotherapy. Therefore, restoration of the antitumor immune response is a cornerstone of therapeutic interventions aimed to control tumor growth. The therapeutic proteasome inhibitor bortezomib sensitizes solid tumors to apoptosis in response to TNF-family death ligands.

**Methods**

We investigated the effects of bortezomib on T cell responses in immunotherapy models involving low-avidity antigens. We also investigated the potential of bortezomib in modulating the antitumor immune response in solid tumor mouse models.

**Results**

Bortezomib did not decrease MHC class I/II-associated antigen presentation to cognate T cells. Rather, bortezomib stabilized the expression of T cell receptor CD3ζ and IL2 receptor-α, while maintaining IFNγ secretion to improve FasL-mediated tumor lysis. Notably, bortezomib increased tumor cell surface expression of Fas in mice as well as human melanoma tissue from a responsive patient. In renal tumor-bearing immunodeficient Rag2^−/−^ mice, bortezomib treatment after adoptive T cell immunotherapy reduced lung metastases and enhanced host survival. We observed that bortezomib treatment also resulted in increased CD8^+^ T lymphocyte IFNγ secretion and expression of effector molecules, perforin and granzyme B, as well as the T-box transcription factor eomesodermin in tumor-bearing mice. Moreover, bortezomib promoted CD8^+^ T cell nuclear factor-κB (NFκB) activity by increasing the total and phosphorylated levels of the IκB kinase and IκBα as well as the cytoplasmic and nuclear levels of phosphorylated p65. In addition, Bzb treatment increased the levels of immunostimulatory interleukins IL-2, IL-12, IL-15, and increased prosphorylation of STAT3/5. Along with these immunostimulatory effects, bortezomib administration reduced number of pulmonary tumor nodules in 4 T1.2HA tumor-bearing mice.

**Conclusions**

These findings provide novel insights on using bortezomib not only as an agent to sensitize tumors to cell death but also to enhance antitumor T cell function, provide lymphocyte-stimulatory effects, and help in modulating lymphocyte-stimulatory cytokine signaling, thereby overcoming immunosuppressive actions of tumor on antitumor T cell functions.

### P333 Extracellular vesicles as carriers for the delivery of immunotherapeutic oligonucleotides

#### Yu-Lin Su^1^, Tomasz Adamus^2^, Qifang Zhang^2^, Sergey Nechaev^2^, Marcin Kortylewski^2^

##### ^1^Beckman Research Institute/City of Hope, Monrovia, CA, USA; ^2^City of Hope, Duarte, CA, USA

###### **Correspondence:** Yu-Lin Su (yulsu@coh.org)

**Background**

While oligonucleotide therapeutics (ONTs) allow for targeting of currently undruggable molecular targets, such as oncogenic/tolerogenic STAT3, their delivery to target cells remains major obstacle limiting clinical application. We previously developed a strategy for delivery of STAT3 siRNA, decoy DNA or antisense ONTs to certain immune and cancer cells as conjugates with TLR9 ligand, CpG oligonucleotide. The CpG-STAT3 inhibitors (CSIs) proved effective in systemic administration against mouse models of leukemia. To further improve conjugate nuclease resistance, circulatory half-life and thereby penetration to distant organs or solid tumors, we developed a method for encapsulation of CSIs in extracellular vesicles (EVs).

**Methods**

We tested several types of immune and cancer cells, such as human and mouse macrophages (RAW264.7), leukemia (MV4-11) and prostate cancer cells (DU145, PC3, TRAMP-C2), for their ability to spontaneously encapsulate CSIs into EVs following the ONT uptake. The loaded EVs were then isolated by a standard ultracentrifugation and characterized as for the vesicle size, concentration (Nanosight) as well as loading efficiency (fluorescent assay/FACS). The biodistribution of EV-encapsulated and fluorescently labeled CpG-siRNA^Cy3^ was assessed after intravenous injections into mice.

**Results**

Cells were incubated with various concentrations of CSIs in EV-free medium for 72 h resulting in the near complete penetration of target cells. The EVs were isolated from supernatants collected from donor cells and further characterized. We found that ~20-40 % isolated EVs were loaded with CSIs. The EVs had an average diameter of 100-150 nm depending on donor cell type. For majority of tested cells, concentration of EVs increased following treatment using CSIs which suggests a potential positive effect of TLR9 signaling on EV secretion. The EV/CSIs were internalized mainly through scavenger receptor-mediated endocytosis and not by a fusion with target cell membrane as verified using confocal microscopy. Importantly, the encapsulation of CpG-STAT3siRNA did not prevent target gene silencing or TLR-dependent NF-kB activation. Systemic injection of EV-encapsulated CpG-STAT3siRNA into mice improved the biodistribution of oligonucleotides especially to myeloid cells in bone marrow and other organs.

**Conclusions**

We demonstrate the feasibility of using EV-encapsulated CSIs for improving systemic delivery of immunostimulatory ONTs to TLR9+ immune and cancer cells. Our further studies will verify whether EV/CSIs will allow for improved penetration of solid tumors and their distant metastases.

### P334 Deep profiling of tumor infiltrating immune subsets by mass cytometry

#### Spencer Wei, James Allison

##### University of Texas MD Anderson Cancer Center, Houston, TX, USA

###### **Correspondence:** Spencer Wei (scwei@mdanderson.org)

**Background**

Checkpoint blockade is able to elicit durable responses in a fraction of cancer patients. However, what factors define responsiveness to checkpoint blockade therapy remain to be elucidated [1, 2]. Tumor cell intrinsic properties and the host immune response co-define responsiveness to checkpoint blockade. We sought to identify facets of the host immune response that are regulated by and functionally important for the efficacy of checkpoint blockade.

**Methods**

To accomplish this we utilized mass cytometry to comprehensively profile changes in tumor immune infiltrates following checkpoint blockade. This approach allows for the interrogation of greater than 40 analytes at single cell resolution [3]. We subsequently analyzed mass cytometry data using clustering and dimension reduction algorithms to identify and visualize infiltrating immune populations in an unsupervised manner.

**Results**

Using this approach we analyzed B16BL6 murine melanoma tumors in mice treated with anti-CTLA-4, anti-PD-1, or control antibody. More than 20 distinct tumor infiltrating immune cell populations were identified. Notably, multiple sub-populations within canonical immune compartments were identified as individual clusters. This suggests that this approach can capture, to an extent, the complexity of tumor immune infiltrates. Using these clusters to categorize immune infiltrates, we identified cell types responsive to checkpoint blockade. Of particular interest, we were able to survey the diversity within the T cell compartment and assess the influence of checkpoint blockade on the frequencies of distinct T cell populations. Broadly, both checkpoint blockade responsive and non-responsive immune clusters were identified, including those that expanded and contracted following treatment (n = 6 to 7 per group; p < 0.05).

**Conclusions**

These results indicate that deep profiling of tumor immune infiltrates using mass cytometry can identify biologically relevant populations in a comprehensive and unsupervised manner. These data support our understanding that CTLA-4 and PD-1 regulate T cell activity through distinct mechanisms. Further investigation into the identity and functional requirement of the identified subsets is required and will help to further elucidate the mechanism of action of individual checkpoint blockade therapies.

**Acknowledgements**

We acknowledge the MDACC core facility NCI Support Grant P30CA16672.

**References**

1. Sharma P, Allison JP: **The future of immune checkpoint therapy**. *Science* 2015, **348**:56–61.

2. Topalian SL, Drake CG, Pardoll DM; **Immune checkpoint blockade: a common denominator approach to cancer therapy**. *Cancer Cell* 2015, **27**:450–461.

3. Tanner SD, Baranov VI, Ornatsky OI, Bandura DR, George TC. **An introduction to mass cytometry: fundamentals and applications**. *CeII* 2013, **62**:955–965.

## Survivorship Issues Related to Immunotherapy

### P335 Neutrophil count predicts survival in patients on ipilimumab with radiation

#### Clark Anderson, Chad Tang, Jonathan Schoenhals, Efrosini Tsouko, John Heymach, Patricia de Groot, Joe Chang, Kenneth R Hess, Adi Diab, Padmanee Sharma, James Allison, Aung Naing, David Hong, James Welsh

##### University of Texas MD Anderson Cancer Center, Houston, TX, USA

###### **Correspondence:** Clark Anderson (clark.anderson@ttuhsc.edu)

**Background**

Neutrophils can have immunosuppressive effects, and the neutrophil-to-lymphocyte ratio (NLR) is a negative prognostic marker in some cancers. We analyzed whether immune cells can predict outcome in patients enrolled in an ongoing clinical trial of radiation plus ipilimumab (NCT 02239900). We hypothesized that patients with greater absolute lymphocyte counts (ALC) or decreased neutrophil counts (NC) will have increased survival.

**Methods**

Data were available from 74 patients. Blood samples for NC and ALC were collected at baseline, at the end of treatment, and immediately before every cycle of ipilimumab. Tumor size was measured by CT scan at baseline, between cycles 2 and 3 of ipilimumab, and every 1–3 months thereafter and response was classified by the immune response criteria (ir-RC). Information on body weight was extracted starting 6 months before treatment through the end of treatment. Continuous and discrete variables were analyzed with Spearman correlations and Fisher’s exact test. Overall survival was compared via log-rank test and hazard ratios obtained by Cox proportional analysis. Commonly reported cut-points used were 5 for NLR and 5x10^9^/L for NC. Associations were considered significant at p < 0.05; all tests were two-sided.

**Results**

Baseline NC correlated with tumor growth (rho = 0.312, p = 0.0069). High baseline NC (>5 x 10^9^/L) was a significant risk factor for progressive disease (odds ratio = 4.83, p = 0.0034); 9 out of 28 patients with high baseline NC had a best response of stable disease or partial response versus 32 out of the 46 patients with low baseline NC. Baseline NC predicted survival (HR = 3.108, p = 0.0006), as did baseline NLR (HR = 2.570, p = 0.0049). NC at the time of the second ipilimumab administration predicted survival more strongly than did NC at baseline (HR = 4.598, p < 0.0001). Both end-of-treatment NC and NLR were associated with survival (NC: HR = 4.881, p < 0.0001; NLR: HR = 5.055, p < 0.0001). Weight loss correlated with an increase in tumor growth (rho = 0.26, p = 0.025), a decrease in ALC (rho = −0.34, p = 0.0031), and an increase in NC (rho = 0.394 p = 0.0022).

**Conclusions**

Our findings suggest that having high NC or NLR is a strong negative prognostic indicator in cancer patients receiving radiation with immunotherapy. These outcomes may reflect neutrophils antagonizing the effects of ipilimumab by suppressing lymphocyte proliferation or exacerbating cachexia.

**Trial Registration**

ClinicalTrials.gov identifier NCT02239900.

**Fig. 47 (abstract P335).**
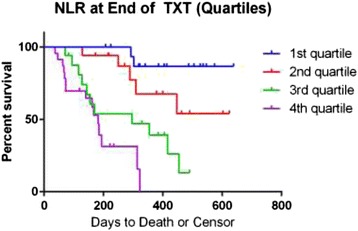
NLR at end of TXT (Quartiles)

## Therapeutic Cancer Vaccines

### P336 Heterologous boosts with an adenoviral vector following a dendritic cell-tropic ZVex® prime generates robust antigen-specific T cell responses and enhanced anti-tumor protection

#### Tina C. Albershardt, Andrea J Parsons, Jardin Leleux, Rebecca S Reeves, Jan ter Meulen, Peter Berglund

##### Immune Design, Seattle, WA, USA

###### **Correspondence:** Tina C Albershardt (tina.albershardt@immunedesign.com)

**Background**

Effective immunization regimens generally require more than one administration, often in the form of prime-boosts. ZVex is an integration-deficient lentiviral vector platform, pseudotyped with a modified Sindbis virus envelope protein to deliver tumor-associated antigens (TAAs) to human dendritic cells (DCs) for optimal priming of TAA-specific CD8+ T cells. We have previously reported that mice immunized once or repeatedly with ZVex/TAA developed strong, dose-dependent, multifunctional, and TAA-specific cytotoxic T cells that critically controlled tumor growth. Here, we show that priming with ZVex/TAA and boosting with adenoviral vector (Ad5) encoding the same antigen strongly increased frequency of TAA-specific T cells and improved anti-tumor efficacy.

**Methods**

To evaluate immunogenicity of ZVex and Ad5 expressing human NY-ESO-1 and murine TRP-1, BALB/c or C57BL/6 female mice were immunized with ZVex/TAA or Ad5/TAA twice, 21 days apart. Splenic T cell responses were assessed 14 days post-last immunization via intracellular cytokine staining. To evaluate therapeutic efficacy of immunization regimens, two murine tumor models were used: 1) a B16 melanoma model, where tumor cells were inoculated in the flank and measured 2–3 per week; and 2) a metastatic CT26 colon carcinoma model expressing human NY-ESO-1, where tumor cells were inoculated intravenously, and lung nodules were enumerated 17–19 days post-tumor inoculation.

**Results**

Repeated ZVex/TAA administration (homologous prime-boost) in mice maintained the frequency of TAA-specific CD8+ T cells at peak levels. While repeat-dose compared to single-dose regimen did not improve anti-tumor control in the CT26 lung metastasis model, it delayed tumor growth in the B16 tumor model, suggesting that homologous prime-boost can be efficacious against selected tumor types. Compared to mice immunized repeatedly with ZVex/TAA, mice primed with ZVex/TAA and boosted with Ad5/TAA (heterologous prime-boost) generated 10-fold increase in frequency of TAA-specific CD8+ T cells capable of producing IFNg, TNF, and/or IL-2. Tumor-bearing mice that received heterologous prime-boost regimen exhibited slower tumor growth or developed fewer metastatic lung nodules than animals that received a homologous regimen. These results demonstrate that a heterologous prime-boost strategy can be used to generate more TAA-specific T cells, leading to more efficacious anti-tumor control.

**Conclusions**

ZVex is a DC-tropic vector platform that efficiently primes robust antigen-specific CD8+ T cell responses that alone can effectively control tumor growth. Heterologous prime-boost regimens, where adenoviral vectors or other modalities are used as booster immunizations, provide exciting opportunities to further enhance this unique DC-tropic gene delivery platform, by further increasing T cell effectors and anti-tumor efficacy.

### P337 Characteristics of adjuvants for therapeutic cancer vaccines

#### Stephane Ascarateil^1^, Marie Eve Koziol^2^

##### ^1^Seppic, Puteaux, Ile-de-France, France; ^2^Seppic Inc., Fairfield, NJ, USA

###### **Correspondence:** Stephane Ascarateil (stephane.ascarateil@airliquide.com)

**Background**

Therapeutic cancer vaccines are an interesting alternative to treat cancer by active immunotherapy. The use of small, highly defined antigens or over-expressed self-antigens is generally linked with weak and too brief immune responses. In order to improve the immune response induced, antigens may be associated with enhancers such as adjuvants. Water-in-oil (W/O) emulsions represent an interesting option for immunotherapy vaccines where potent adjuvants are required. These emulsions, based on Montanide™ ISA 51VG adjuvant, have been successfully used to increase the biological efficacy and immunogenicity of human therapeutic peptides vaccines. Some of the mechanisms of action that allow this potent and prolonged stimulation are brought forward.

**Methods**

Cellular activation mechanisms: 5 C57BL/6 mice per group were vaccinated subcutaneously with 25 μg of nucleoprotein (NP) alone or with the Montanide™ ISA 51 VG at weeks 0 and 3. At week 5, splenocytes are sampled. T cells are put in culture for 48 h and restimulated with NP antigen. IFNɣ response is followed by ELISpot. Cytokine secretions into the medium (supernatant) (TNFα, IL-2, IFNɣ) were measured by ELISA. Distinct populations of memory CD8+ T cells were evaluated by flow cytometric analysis.

**Results**

Mice immunized with NP associated with the Montanide™ ISA 51 VG elicited an increase in anti-NP T cells, CD4+ and CD8+ T cell responses. We observe a significant increase of IFNɣ response in the group vaccinated with adjuvant. Response from total splenocytes is increased 6 times, 5 times for CD4+ population and more than 4 times for CD8+ T cell population. Mice immunized with the NP associated to the Montanide™ ISA 51 VG showed an increase in TNFα, IL-2, and IFNɣ cytokine secretion. Mice immunized with NP antigen associated with the Montanide™ ISA 51 VG elicited a higher amount of effector memory T lymphocytes and central memory T lymphocytes. The higher amount of CD44+ CD62L+ (T_CM_ sub-population) in mice immunized with NP associated to Montanide™ ISA 51 VG showed an increased engraftment and persistence of T cells.

**Conclusions**

Vaccines based on Montanide™ ISA 51 VG are strong inducers of danger signals through an enhancement of interaction between antigen and dendritic cells. They induce an important IFNɣ TH1 polarized response, and potent CD8+ T cell response. Montanide™ ISA 51 VG is an interesting candidate in therapeutic cancer vaccines. Moreover it has been safely administered to almost 20,000 patients in 258 clinical trials, some of them being included in vaccination schedules involving repeated doses over several years.

**Fig. 48 (abstract P337).**
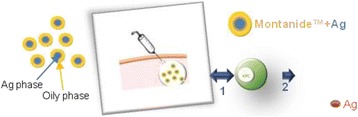
W/O emulsion structure and mechanism of immune stimulation

**Fig. 49 (abstract P337).**
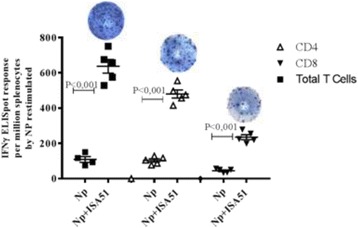
Cytotoxic T Lymphocytes response following vaccination based on Montanide™ ISA 51 VG

**Fig. 50 (abstract P337).**
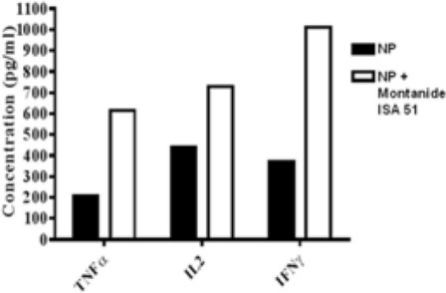
TNFα, IL-2, IFNɣ secretion after in vitro T cells NP restimulation

**Fig. 51 (abstract P337).**
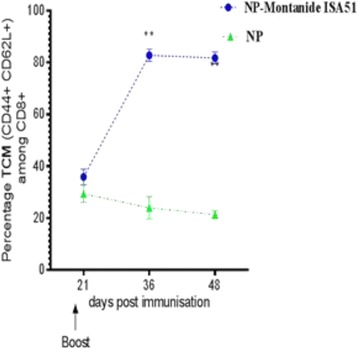
TCM (CD44+ CD62L+) sub-population of CD8+ cells in mice vaccinated with or without Montanide™ ISA 51 VG

### P338 Glycosylated and methylated peptides as neoantigens in leukemia

#### Sarah A Penny^1^, Stacy A Malaker^2^, Lora Steadman^1^, Paisley T Myers^3^, Dina Bai^3^, Jeffrey Shabanowitz^3^, Donald F Hunt^3^, Mark Cobbold^4^

##### ^1^University of Birmingham, Birmingham, England, UK; ^2^Stanford University, Stanford, CA, USA; ^3^University of Virginia, Charlottesville, VA, USA; ^4^Massachusetts General Hospital Cancer Center, Boston, MA, USA

###### **Correspondence:** Mark Cobbold (mcobbold@mgh.harvard.edu)

**Background**

Recent advances have highlighted the importance of the immune response in the fight against cancers. In many cancers, these responses are thought to target mutated peptides; however, leukemia has been shown to have a lower mutational load than many cancers, despite being highly immunogenic. Thus, leukemia-specific antigens may derive from the posttranslational modifications (PTMs) associated with aberrant signaling. Previously, phosphorylated peptides have been identified as potent cancer antigens; here, we identity several peptides with O-linked β-*N*-acetylglucosamine (O-GlcNAc) modifications, with some that also contain methylated arginine residues. O-GlcNAc is a PTM that modulates cellular functions through extensive cross-talk with the signaling cascades also regulated by phosphorylation. Thus, O-GlcNAcylated peptides may represent cancer-specific neoantigens.

**Methods**

We eluted MHC class-I associated peptides from leukemia patient samples to identify O-GlcNAcylated antigens, using enrichment coupled with high-resolution mass spectrometry. Healthy donor immune responses were assessed using IFNγ ELISpot and multiplexed intracellular cytokine staining. Functionality was assessed using a europium-release killing assay.

**Results**

We have identified 36 MHC class I associated O-GlcNAc neoantigens from primary leukemia samples, the first tumor antigens containing this PTM. A subset of these neoantigens is linked to key cancer pathways, including the mitogen activated protein kinase (MAPK) and retinoblastoma (RB1) pathways, and these peptides were shared across all of the patient samples tested. 71 % (5/7) of the HLA-B*0702 O-GlcNAcylated neoantigens tested were immunogenic, with 100 % (5/5) of healthy donors having multifunctional memory CD8+ T cell responses to them. Cells targeting these neoantigens were shown to degranulate. This multifuntionality and degranulation in response to antigen indicated that O-GlcNAc-specific T cells may kill. Indeed, an O-GlcNAc-specific T cell line was grown and these cells specifically killed autologous cells pulsed with the modified peptide, but not the equivalent unmodified peptide (p = 0.015). T cell responses were also identified that specifically targeted the methylated arginine in the peptide with the O-GlcNAc modification.

**Conclusions**

O-GlcNAcylated neoantigens derive from aberrations in key cancer pathways, are shared across patients and are immunogenic. CD8+ T cells targeting these O-GlcNAcylated neoantigens specifically recognize and kill only the PTM antigen. Therefore, these O-GlcNAcylated neoantigens provide logical targets for cancer immunotherapy.

### P339 Intratumoral delivery of modified vaccinia virus Ankara expressing human Flt3L as cancer immunotherapy

#### Peihong Dai^1^, Weiyi Wang^1^, Ning Yang^1^, Stewart Shuman^1^, Taha Merghoub^2^, Jedd D Wolchok^3^, Liang Deng^3^

##### ^1^Memorial Sloan Kettering Cancer Center, New York, NY, USA; ^2^Ludwig Collaborative Laboratory, Memorial Sloan Kettering Cancer Center, New York, NY, USA; ^3^Department of Medicine, Memorial Sloan Kettering Cancer Center, New York, NY, USA

###### **Correspondence:** Liang Deng (dengl@mskcc.org)

**Background**

Modified vaccinia virus Ankara (MVA) is a highly attenuated vaccinia strain that is an important vaccine vector for infectious diseases and cancers. MVA has a 31-kb deletion of the parental vaccinia genome and was shown to be safe for human use during smallpox vaccination. The investigation of MVA as cancer therapeutics has so far been limited to its use as a vaccine vector to express tumor antigens. We hypothesize that intratumoral delivery of recombinant MVAΔE3L (with deletion of vaccinia virulence factor E3) expressing human flt3L (Fms-like tyrosine kinase 3 ligand) would provide *in situ* therapeutic vaccine effects. Flt3L plays a critical role in the development of DC subsets, including CD103^+^/CD8α^+^ DCs, which are critical for cross-presentation of tumor antigens.

**Methods**

We compared the immune responses of B16-F10 murine melanoma cells and MC38 murine colon adenocarcinoma cells as well as dendritic cells to either MVA or MVAΔE3L infection. We compared the efficacy of intratumoral delivery of MVA vs. MVAΔE3L in two syngeneic bilateral tumor implantation models. We also generated recombinant MVAΔE3L-TK^−^-hFlt3L through homologous recombination at the thymidine kinase (TK) locus. We compared the efficacies of intratumoral delivery of MVAΔE3L-TK^−^-hFlt3L vs. MVAΔE3L in bilateral tumor implantation models.

**Results**

We found that MVA∆E3L infection of B16-F10 and MC38 induces higher levels of IFN-β, IL-6, CCL4 and CCL5 than MVA. MVA∆E3L-induction of type I IFN in cDCs is mainly dependent on the cGAS/STING pathway. Intratumoral injection of MVAΔE3L is more efficacious than MVA in tumor eradication and extension of survival in bilateral tumor implantation models, which correlates with stronger induction of activated CD8^+^ and CD4^+^ effector T cells in both injected and non-injected tumors from MVAΔE3L-treated mice compared with MVA-treated mice. Furthermore, intratumoral injection of MVAΔE3L-TK^−^-hFlt3L exerts stronger anti-tumor effects than MVAΔE3L in a murine melanoma bilateral implantation model. B16-F10-tumor bearing mice successfully treated with MVAΔE3L-TK^−^-hFlt3L also rejected a lethal dose of MC38 challenge.

**Conclusions**

Our results show that intratumoral injection of MVA or MVAΔE3L leads to alteration of tumor immune suppressive microenvironment, which facilitates tumor antigen presentation, recruitment and activation of anti-tumor CD8^+^ and CD4^+^ T cells. MVAΔE3L is a stronger immune activator than MVA. Intratumoral delivery of MVAΔE3L-TK^−^-hFlt3L is more efficacious than MVAΔE3L. Current studies focuses on tumor infiltrating immune cells including CD103^+^ DCs and CD8^+^ cytotoxic T cells in MVAΔE3L-TK^−^-hFlt3L vs. MVAΔE3L-treated mice.

**Fig. 52 (abstract P339).**
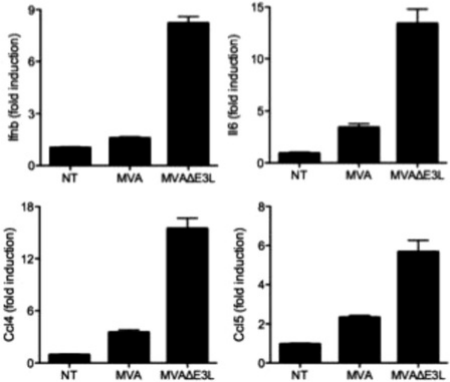
MVA∆E3L infection of B16-F10 cells induces higher levels of Ifnb, Il6, Ccl4 and Ccl5 than MVA. B16-F10 melanoma cells (1 x 10e6) were infected with MVA, and MVA∆E3L viruses at a MOI of 10. Cells were collected at 6 h post infection. Real-time PCR was performed to analyze gene expression

**Fig. 53 (abstract P339).**
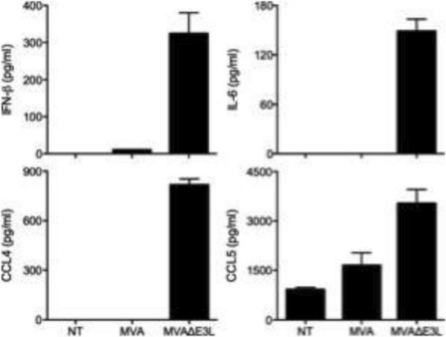
MVA∆E3L infection of MC38 cells induces higher levels of IFN and proinflammatory cytokines and chemokines than MVA. MC38 colon cancer cells (1 x 10e6) were infected with MVA, and MVA∆E3L viruses at a MOI of 10. Supernatants were collected at 22 h post infection. The levels of cytokines were determined by ELISA

**Fig. 54 (abstract P339).**
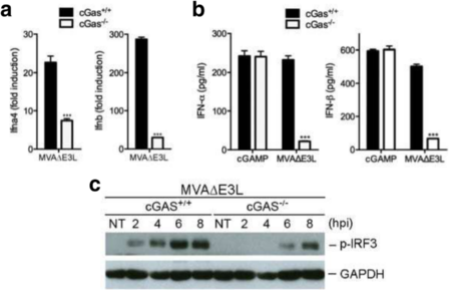
Innate immune sensing of MVA∆E3L virus in cDCs. Shown here is a series of bar graphs showing that cGAS is required for the induction of type I IFN by MVA∆E3L in cDCs. **a** Bar graphs showing mRNA levels of IFNA4 and IFNB in cGAS+/+ and cGAS−/− cDCs infected with MVA∆E3L. **b** Bar graphs showing IFN-α and IFN-β secretion levels in cGAS+/+ and cGAS−/− cDCs infected with MVA∆E3L or treated with cGAMP, an agonist for STING. **c** A scanned image of an immunoblot showing protein levels of p-IRF3 and GAPDH in cGAS+/+ and cGAS−/− cDCs infected with MVA∆E3L

**Fig. 55 (abstract P339).**
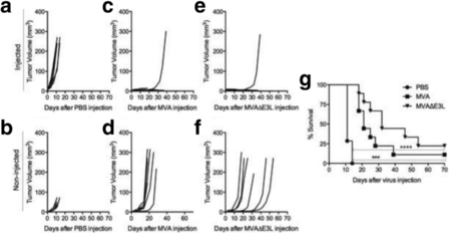
Intratumoral injection of MVA∆E3L or MVA is effective in a B16-F10 bilateral implantation model. B16-F10 bilateral tumor implantation model was used to assess the anti-tumor efficacy of MVA∆E3L vs MVA. Briefly, B16-F10 melanoma cells were implanted intradermally to the left and right flanks of C57B/6 mice (5 x 10e5 to the right flank and 1 x 10e5 to the left flank). 8 days after tumor implantation, the larger tumors on the right flank were intratumorally injected with 2 x 10e7 pfu of MVA or an equivalent amount of MVAΔE3L. The tumor sizes were measured and the tumors were re-injected twice a week. The survival of mice was monitored. Graphs of injected (**a**, **c**, **e**) and non-injected (**b**, **d**, **f**) tumor volume plotted against time (days) after PBS, MVA, or MVAΔE3L injection respectively. **g** is a Kaplan-Meier survival curve of tumor-bearing mice (B16-F10 cells) injected with PBS (filled circles), MVA (filled squares), or MVAΔE3L (filled triangles). ****, p < 0.0001 (MVA∆E3L vs. PBS group); ***, p < 0.001 (MVA vs. PBS group)

**Fig. 56 (abstract P339).**
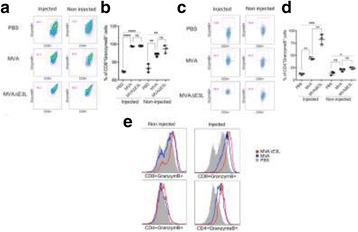
Intratumoral injection of MVA∆E3L and MVA induces activating TILs in injected and non-injected tumors. B16-F10 melanoma cells were implanted intradermally to the left and right flanks of C57B/6 mice (5 x 10e5 to the right flank and 2.5 x 10e5 to the left flank). 7 days after tumor implantation, the larger tumors on the right flank were intratumorally injected with 2 x 10e7 pfu of MVA or an equivalent amount of MVAΔE3L, repeated three days later. Both injected and non-injected tumors were harvested at 3 days post the second injection, and TILs were analyzed by FACS. Shown here is a series of graphical representations of data showing that intratumoral injection of MVA or MVA∆E3L induces activated effector CD8+ and CD4+ T cells in both injected and non-injected tumors in a murine B16-F10 melanoma bilateral implantation model. (A) Dot-plots of flow cytometric analysis of CD8+ cells expressing granzyme B+. (B) %CD8+ granzyme B+ T cells in both injected and non-injected tumors treated with PBS, MVA or MVA∆E3L. (C) Dot-plots of flow cytometric analysis of CD4+ cells expressing granzyme B+. (D) %CD4+ granzyme B+ T cells in both injected and non-injected tumors treated with PBS, MVA or MVA∆E3L. (*, p < 0.05; **, p < 0.01; ***, p < 0.001; ****, p < 0.0001). (E) Histogram of CD8+ granzyme B+ and CD4+ granzyme B+ TILs in both injected and non-injected tumors treated with PBS, MVA or MVA∆E3L

**Fig. 57 (abstract P339).**
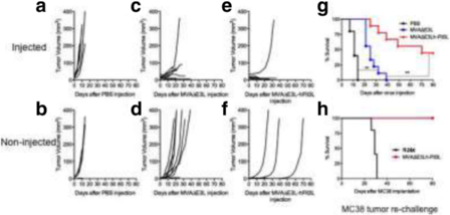
MVA∆E3L-hFlt3L is more efficacious than MVA∆E3L. Shown here are tumor volumes of injected (**a**, **c** and **e**) and non-injected tumors (**b**, **d**, and **f**). (**g**) A Kaplan-Meier survival curve of tumor-bearing mice (B16-F10 cells) injected with PBS (filled circles), MVA∆E3L (filled squares), or MVAΔE3L-hFlt3L (filled triangles). **, p < 0.01 (MVA∆E3L vs. PBS group); **, p < 0.01 (MVA∆E3L-hFlt3L vs. MVA∆E3L group). (H) A Kaplan-Meier survival curve of tumor-bearing mice (B16-F10 cells) successfully treated with MVA∆E3L-hFlt3L vs naive mice challenged with a lethal dose of MC38 cells intradermally (1 x 10e5)

### P340 A pilot study of the immunogenicity of a 9-peptide breast cancer vaccine plus poly-ICLC in stage IB-IIIA breast cancer

#### Patrick Dillon^1^, Gina Petroni^1^, David Brenin^1^, Kim Bullock^1^, Walter Olson^1^, Mark E Smolkin^2^, Kelly Smith^1^, Carmel Nail^1^, Craig L Slingluff Jr^3^

##### ^1^University of Virginia, Charlottesville, VA, USA; ^2^Department of Public Health Sciences, University of Virginia, Charlottesville, VA, USA; ^3^Division of Surgical Oncology, University of Virginia, Charlottesville, VA, USA

###### **Correspondence:** Patrick Dillon (pmd5b@hscmail.mcc.virginia.edu)

**Background**

Breast cancer remains a leading cause of cancer death worldwide. Effective adjuvant therapy exists, but there is evidence that immunotherapy may play a significant role in the eradication of residual disease. Peptide vaccines require adjuvants to achieve durable immune memory. Toll-like receptor agonists and help peptides are two recently optimized adjuvants which are investigated in this trial.

**Methods**

A vaccine consisting of nine-class I MHC-restricted breast cancer-associated peptides was combined with a TLR3, poly-ICLC) along with a helper peptide from tetanus toxoid. The peptides used in the study are encoded by the genes: MAGE-A1, −A3, −A10, CEA, NY-ESO-1, and HER2. The peptides lack tumor-specific mutations. The vaccine was given on days 1, 8, 15, 36, 57, 78 and response was assessed by both direct and stimulated ELISpot. Eleven patients with breast cancer were treated. Five of the patients had estrogen receptor positive disease. None were HER2 amplified.

**Results**

The vaccine was well tolerated with no grade 3 nor dose limiting toxicities. Mild injection site reactions and flu-like symptoms were reported in most patients. The most common toxicities were injection site reaction/induration and fatigue, which were experienced by 100 % and 91 % of participants, respectively. The stimulated ELISpot detected T cell responses in four out of eleven patients. None were detectable in a direct ELISpot assay. Another two patients had borderline immune responses and four had immune response extending 30 days beyond the end of the vaccination series. No difference in immune response was observed between patients receiving endocrine therapy and those not receiving endocrine therapy. The peptides from CEA and MAGE-A1 were immunogenic.

**Conclusions**

The administration of a peptide vaccine in the adjuvant breast cancer setting was safe and feasible. An adjuvant poly-IC plus helper peptide mixture provided modest immune stimulation and should be further optimized for use with peptide vaccines.

**Trial Registration**

ClinicalTrials.gov identifier NCT01532960.

### P341 NKTR-214, an engineered cytokine, synergizes and improves efficacy of anti-cancer vaccination in the treatment of established murine melanoma tumors

#### Meenu Sharma^1^, Faisal Fa'ak^2^, Louise Janssen^1^, Hiep Khong^1^, Zhilan Xiao^1^, Yared Hailemichael^2^, Manisha Singh^1^, Christina Vianden^1^, Adi Diab^1^, Jonathan Zalevsky^3^, Ute Hoch^3^, Willem W Overwijk^1^

##### ^1^University of Texas MD Anderson Cancer Center, Houston, TX, USA; ^2^Department of Melanoma Medical Oncology, University of Texas MD Anderson Cancer Center, Houston, TX, USA; ^3^Nektar Therapeutics, San Francisco, CA, USA

###### **Correspondence:** Faisal Fa'ak (ffaak@mdanderson.org)

**Background**

IL-2 has been used as effective immunotherapy in metastatic renal cell carcinoma and melanoma, and may synergize with other cancer immunotherapies. However, toxicities associated with high dose IL-2 treatment limited its further use in anti-cancer therapies. NKTR-214, which is an engineered IL-2 cytokine, was designed to provide a non-toxic, stable and more efficient alternative to IL-2. NKTR-214 provides sustained activation of the IL-2 pathway through controlled release of active CD122-biased (IL-2Rβɣ) cytokines. Prior preclinical studies demonstrated that NKTR-214 can expand tumor-infiltrating lymphocyte populations resulting in marked tumor growth suppression as single-agent and in combination with checkpoint inhibitors. In this pre-clinical study, we investigated whether NKTR-214 can promote expansion and function of vaccination-induced, tumor specific effector CD8+ T cells using the murine B16 melanoma model. We also studied how NKTR-214 impacts the localization of effector CD8+ T cells and Tregs to tumor and spleen.

**Methods**

To understand the effect of NKTR-214 on antigen-specific CD8+ T cells, we adoptively transferred naïve gp100-specific TCR transgenic pmel-1 CD8+ T cells into mice bearing established subcutaneous B16 tumors, followed by vaccination (gp100 peptide + anti-CD40 mAb + TLR-7 agonist) alone or in combination with NKTR-214 or IL-2. Mice then received NKTR-214 or IL-2 every 8 days. Tumor growth, survival and T cell response in blood was monitored, and localization of effector pmel-1 CD8+ T cells and CD4+ Foxp3+ Tregs in tumor and spleen were analyzed.

**Results**

NKTR-214 efficiently synergized with vaccination, potently suppressing tumor growth and improving survival of mice compared to vaccination with IL-2. NKTR-214 enhanced pmel-1 CD8+ T cell numbers and decreased numbers of immune-suppressive Tregs in tumor. NKTR-214 was able to stably maintain a high ratio of pmel-1 CD8+ T cells over Tregs in tumor for >30 days. Despite the induction of very strong CD8+ T cell responses and anti-tumor activity, no gross toxicity was observed.

**Conclusions**

NKTR-214 synergizes with vaccination by supporting the survival, maintenance and tumor infiltration of effector CD8+ T cells without promoting the intratumoral accumulation of immune-suppressive Tregs. These preclinical results establish that NKTR-214 is highly effective in increasing CD8+ effector T cell responses with potent anti-tumor activity.

### P342 A tumor mitochondria vaccine protects against experimental renal cell carcinoma

#### Andrea Facciabene, Pierini Stefano, Fang Chongyung, Stavros Rafail

##### University of Pennsylvania, Philadelphia, PA, USA

###### **Correspondence:** Andrea Facciabene (facciabe@mail.med.upenn.edu)

**Background**

Mitochondria provide energy for cells via oxidative phosphorylation. Reactive oxygen species, a byproduct of this mitochondrial respiration, can damage mitochondrial DNA (mtDNA), and somatic mtDNA mutations have been found in all colorectal, ovarian, breast, urinary bladder, kidney, lung, and pancreatic tumors studied. The resulting altered mitochondrial proteins or tumor-associated mitochondrial antigens (TAMAs) are potentially immunogenic, suggesting that they may be targetable antigens for cancer immunotherapy.

**Methods**

We generated a cellular tumor vaccine by pulsing dendritic cells with enriched mitochondrial proteins from RENCA cells.

**Results**

Our dendritic cell-based RENCA mitochondrial lysate vaccine elicited a cytotoxic T cell response *in vivo* and conferred durable protection against challenge with RENCA cells when used in a prophylactic or therapeutic setting. By sequencing mtDNA from RENCA cells, we identified two mutated molecules: COX1 and ND5. Peptide vaccines generated from mitochondrial-encoded COX1 but not from ND5 had therapeutic properties similar to RENCA mitochondrial protein preparation.

**Conclusions**

Our results demonstrated that TAMAs can elicit effective antitumor immune responses, potentially providing a new immunotherapeutic strategy to treat cancer.

### P343 Integrin activator 7HP349 enhances anti-CTLA-4 antibody-based cancer therapy

#### Yared Hailemichael^1^, Michael Nielsen^1^, Faisal Fa'ak^1^, Peter Vanderslice^2^, Darren G Woodside^3^, Robert V Market^4^, Ronald J Biediger^4^, Upendra K Marathi^4^, Willem W Overwijk^1^

##### ^1^Department of Melanoma Medical Oncology, University of Texas MD Anderson Cancer Center, Houston, TX, USA; ^2^Texas Heart Institute, Houston, TX, USA; ^3^Department of Molecular Cardiology, Texas Heart Institute, Houston, TX, USA; ^4^7 Hills Pharma LLC, Houston, TX, USA

###### **Correspondence:** Yared Hailemichael (yhailemi@mdanderson.org)

**Background**

Checkpoint blockade therapy has therapeutic benefit in several human cancers [1], but in many patients, checkpoint blockade-induced T cells do not infiltrate tumors [2,3], preventing clinical benefit. One mechanism of intratumoral T cell accumulation is through the activity of the integrins very late antigen-4 (VLA-4) and lymphocyte function-associated antigen-1 (LFA-1) on activated T cells. We evaluated the effect of an integrin agonist, 7HP349, on promoting intratumoral T cell accumulation to potentiate CTLA-4 checkpoint blockade-induced anti-tumor activity.

**Methods**

To evaluate the effect of 7HP349 in promoting checkpoint blockade-induced anti-tumor immunity, we combined it with anti-CTLA-4 therapy in the standard treatment model of established subcutaneous B16 melanoma [4].

**Results**

CTLA-4 checkpoint blockade enhanced vascular cell adhesion molecule-1 (VCAM-1) and intercellular cell adhesion molecule-1 (ICAM-1) expression on tumor vasculature consequently resulting in increased intratumoral accumulation of CD8^lo^CD11a^hi^CD44^hi^ effector T cells (Teff) and inflammatory monocytes (iMO) and granulocytes (Gran). ICAM-1 antibody blockade or genetic ablation caused reduced accumulation of Teff and tumor control. Intratumoral administration of 7HP349 enhanced CD8+ T cell accumulation and tumor control compared to mice treated with anti-CTLA-4 monotherapy or with vehicle control (P < 0.01). Mice treated with anti-CTLA-4 and 7HP349 showed increased depigmentation (vitiligo) suggesting immunity to melanocyte differentiation antigens. Therapeutic efficacy was also observed after systemic administration of 7HP349 in combination with anti-CTLA-4 therapy, resulting in (80 %) tumor-free survival compared to anti-CTLA-4 monotherapy (33 %) (P < 0.01).

**Conclusions**

Activation of integrin cell adhesion molecules with 7HP349 is a promising approach to enhance the anti-cancer activity of checkpoint blockade therapy with antibodies against CTLA-4, and possibly other checkpoint molecules such as PD-1 and PD-L1.

**References**

1. Sharma P, Allison JP: **Immune checkpoint targeting in cancer therapy: toward combination strategies with curative potential**. *Cell* 2015, **161**, 205–214.

2. Gajewski TF: **The Next Hurdle in Cancer Immunotherapy: Overcoming the Non-T-Cell-Inflamed Tumor Microenvironment**. *Sem Oncol* 2015, **42**:663–671.

3. Peske JD, Woods AB, Engelhard VH: **Control of CD8 T-Cell Infiltration into Tumors by Vasculature and Microenvironment**. *Adv Cancer Res* 2015, **128**:263–307.

4. van Elsas A, *et al.*: **Elucidating the autoimmune and antitumor effector mechanisms of a treatment based on cytotoxic T lymphocyte antigen-4 blockade in combination with a B16 melanoma vaccine: comparison of prophylaxis and therapy**. *J Exp Med* 2001, **194**:481–489.

### P344 Intramuscular anti-HER2 antibody gene transfer as an alternative to conventional antibody protein therapy: a pre-clinical proof of concept study in breast cancer

#### Kevin Hollevoet, Nick Geukens, Paul Declerck

##### KU Leuven, Leuven, Vlaams-Brabant, Belgium

###### **Correspondence:** Kevin Hollevoet (kevin.hollevoet@kuleuven.be)

**Background**

Monoclonal antibodies (mAbs) are taking the field of cancer immunotherapy by storm. Despite their growing use, a broader accessibility and implementation is hampered by dismal pharmaco-economics, the practicalities and patient discomfort associated with frequent and long-term high-dose mAb administration, and the often limited therapeutic effect as single agent. To address these issues, the laboratory focuses on the development of a therapeutic DNA platform for non-viral antibody gene transfer. After administration of vectors that carry the encoding mAb sequences, this approach enables the site of delivery (e.g., muscle or tumor) to produce mAbs *in vivo* followed by secretion into circulation for a prolonged period of time. *In vivo* mAb expression presents a labor- and cost-effective alternative to the conventional production, purification and parenteral delivery of mAb proteins. The less frequent administrations and gradual *in vivo* mAb production and buildup are anticipated to improve patient comfort and safety. Also, by making mAb therapy more affordable, more effective combinations could be implemented more easily.

**Methods**

This study aims to demonstrate the feasibility of intramuscular mAb gene electrotransfer in mice using the well-characterized trastuzumab or its murine precursor 4D5 encoded in plasmid DNA (pDNA). Following intramuscular pDNA injection and electroporation, mAb plasma levels were measured with ELISA using HER2-coated plates. *In vivo* therapeutic efficacy of mAb gene transfer was evaluated in a BT474 breast cancer mouse model.

**Results**

Following intramuscular electrotransfer of the encoding pDNA in BALB/c mice, trastuzumab was found at microgram per milliliter concentrations in plasma. In this immune competent strain, however, detection was lost 10 days after pDNA delivery, because of an immune response against the humanized trastuzumab. This was overcome by delivery of trastuzumab pDNA in immune compromised mice (RAG2−/−gammaC−/− and athymic nude mice) or by delivery of 4D5 pDNA, thus matching the mAb sequence with the host species. Both approaches resulted in continued mAb expression at microgram per milliliter concentrations for at least 6 months, the duration of the follow-up. mAb plasma concentration could be adjusted by adapting the pDNA dose or administering additional pDNA doses. In a BT474 xenograft mouse model, intramuscular electrotransfer of 4D5 pDNA induced significant anti-tumor responses compared to the untreated control group.

**Conclusions**

This study achieved proof of concept for prolonged and therapeutically relevant *in vivo* mAb expression in mice using anti-HER2 mAbs as demonstrator. Ongoing work focuses on expanding the DNA platform to immunomodulatory mAb combinations and bridging the gap towards clinical application.

### P345 Direct identification of neoepitopes in tumor tissue

#### Nathalie Joly, Laura McIntosh, Eustache Paramithiotis

##### Caprion Biosciences Inc., Montreal, PQ, Canada

###### **Correspondence:** Laura McIntosh (lmcintosh@caprion.com)

**Background**

Antigen presentation by MHC is central to the development of adaptive immunity. While traditional modeling approaches yield large numbers of candidates and high attrition rates, direct identification of naturally-processed peptides by mass spectrometry can significantly streamline the neo-epitope identification.

**Methods**

To demonstrate the feasibility and advantages of this approach, a pilot study was conducted in clear cell renal cell carcinoma. MHC I-peptide complexes were isolated from tumor and matched adjacent normal tissue from treatment-naïve patients with the same tumor grade but heterogeneous HLA haplotypes. Peptides were eluted from the complexes using mild acid and were analyzed by mass spectrometry and their expression was compared to matched adjacent tissue.

**Results**

Results demonstrated effective enrichment and detection of MHC- associated peptides, with identification of an average of over 6800 peptides per sample and characteristics appropriate for peptides presented by MHC I. Differential expression analysis indicated that approximately 13 % of identified peptides were substantially overexpressed (>3-fold) in the tumor tissue, with approximately 3.5 % uniquely presented in tumors. In many cases multiple HLA allele-specific peptides derived from the same tumor-presented protein were identified, thereby increasing coverage across different haplotypes. A relatively small number of modified peptides presented only by the tumor were identified, consistent with the low mutational load of clear cell renal cell carcinoma. Most of these peptides appeared to be derived from protein fusions (37 %), single amino acid substitutions (25 %) and frameshift mutations (19 %), with a lower contribution from splicing variants (6 %) and post-translational modifications (9 %). Pathway analysis showed significant over-representation of proteins associated with hypoxia and angiogenesis, two processes previously reported to change in clear cell renal carcinoma.

**Conclusions**

Thus tumor-associated antigen presentation reflected protein expression changes previously reported in renal cell carcinoma, and identified multiple novel candidates. Direct identification of naturally processed peptides generated a small but high quality list of candidates for further investigation.

### P346 Intratumorally injected pro-inflammatory allogeneic dendritic cells as immune enhancers - a phase I/II study in patients with advanced hepatocellular carcinoma

#### Magnus Rizell^1^, Malin Sternby^1^, Bengt Andersson^2^, Alex Karlsson-Parra^3^

##### ^1^Transplant Institute, Sahlgrenska Academy at University of Gothenburg, Gothenburg, Sweden; ^2^Department of Clinical Immunology, Gothenburg University, Gothenburg, Sweden; ^3^Department of Immunology, Genetics and Pathology, Uppsala University, Uppsala, Sweden

###### **Correspondence:** Alex Karlsson-Parra (alex.karlsson-parra@igp.uu.se)

**Background**

Accumulating pre-clinical data indicate that the efficient induction of antigen-specific cytotoxic T cells characterizing viral infections is caused by cross-priming where initially infected DCs act as a pure adjuvant, not as antigen presenting cells, producing inflammatory factors that recruit and activate non-infected "bystander" DCs. Accordingly, we have developed a cellular adjuvant consisting of pro-inflammatory monocyte-derived allogeneic DCs producing high levels of DC-recruiting and DC-maturating factors in a sustained fashion.

**Methods**

Eleven patients with advanced hepatocellular carcinoma (HCC) where included in a phase I/II study. Vaccine cells (INTUVAX) were produced from a leukapheresis-product collected from one healthy blood donor and subsequently deep-frozen. Three doses of vaccine cells (10–20 million cells/dose) were injected intratumorally. Using mixes of overlapping peptides for alpha fetoprotein (AFP) and human telomerase reverse transcriptase (hTERT) spanning the entire protein sequence, CD8+ T cell responses could be evaluated, regardless of the patient's HLA-type.

**Results**

Nine out of 11 patients received all 3 vaccine doses. Six of these 9 patients had received prior first-line systemic treatment with sorafenib while 3 patients were naive to systemic treatment. INTUVAX was generally well tolerated but two severe adverse events, both fever episodes, were reported as drug-related. Among the 9 fully treated patients, 6 patients exhibited an increase of AFP and/or hTERT specific and IFN-gamma producing CD8+ T cells 1 week after the third vaccine dose as compared to pre-vaccination levels. Median overall survival (mOS) for the fully treated patient subgroup given INTUVAX as second-line systemic treatment (n = 6) is still not reached but is currently (8AUG2016) 8.2 months while mOS for the three patients receiving INTUVAX as first-line systemic treatment was 11.7 months. These data compare favorably with historical data. Notably, two out of three patients who did not respond with an increase in tumor-specific T cells died within 5 months after the first INTUVAX dose.

**Conclusions**

Our findings indicate that intratumoral administration of proinflammatory allogeneic DCs induces a CTL-mediated systemic anti-tumor response in a majority of HCC patients that this response correlate to prolonged survival.

**Trial Registration**

ClinicalTrials.gov identifier NCT01974661.

### P347 Nanodisc neoantigen vaccination combined with immune checkpoint blockade efficiently eliminates established tumors

#### Rui Kuai, Lukasz Ochyl, Anna Schwendeman, James Moon

##### University of Michigan, Ann Arbor, MI, USA

###### **Correspondence:** Rui Kuai (ruikuai@umich.edu)

**Background**

With the rapid development of next-generation DNA/RNA sequencing technology, patient-specific tumor neo-antigens can now be identified, potentially ushering in the new era of personalized cancer vaccines. Peptide vaccines, known for ease of manufacturing, quality control and human safety, can be easily applied for neo-antigen-based immunotherapy. However, peptide-based cancer vaccines have shown limited therapeutic efficacy in humans, partially due to inefficient co-delivery of antigen (Ag) and adjuvants to lymphoid tissues as well as T cell dysfunction and deletion.

**Methods**

Here we report that synthetic high density lipoprotein (sHDL) nanodiscs, with an established clinical manufacturing procedure and excellent safety profiles in humans, can be simply mixed with Ag peptides and adjuvants, producing homogeneous, stable, and ultrasmall (~10 nm in diameter) nanodiscs in less than 2 hrs for personalized neo-antigen vaccination.

**Results**

Nanodiscs efficiently co-delivered Ag and CpG, a Toll-like receptor-9 agonist, to draining lymph nodes and promoted strong and durable Ag presentation on antigen-presenting cells. Strikingly, nanodiscs elicited up to 47-fold greater frequency of tumor neoantigen-specific CD8+ T lymphocytes (CTLs) than soluble vaccines and even 31-fold greater than arguably the strongest CTL adjuvant in clinical trials (i.e., CpG in Montanide). Moreover, in mice bearing MC-38 colon tumors, therapeutic sHDL vaccination led to significantly enhanced IFN-γ^+^TNF-α^+^ Ag-specific CTL responses that substantially inhibited tumor growth and extended animal survival, compared with soluble vaccines (p < 0.01). When nanodisc vaccination was combined with the immune checkpoint inhibitor, anti-PD-1 (α-PD-1), ~88 % of MC-38 tumor-bearing mice were cured, whereas the soluble peptide + CpG vaccine combined with α-PD-1 therapy cured only 25 % mice. Those cured mice were completely protected against MC-38 cell re-challenge administered on day 70, indicating resistance to tumor relapse. To treat a more aggressive B16F10 melanoma model, multiple MHC class I and class II epitopes were loaded in nanodiscs. Vaccination with multi-epitope nanodiscs generated ~30 % tumor antigen-specific, IFN-γ^+^ CD8+ and CD4+ T cells in peripheral blood, whereas only ~2 % response was observed for the soluble vaccine or Ag + CpG + Montanide group. More strikingly, when multi-epitope nanodisc vaccination was combined with α-PD-1/α-CTLA-4 therapy, ~90 % B16F10 tumor-bearing mice were cured, whereas only ~38 % rate of tumor regression was observed in animals treated with the soluble peptide vaccine plus α-PD-1/α-CTLA-4 therapy.

**Conclusions**

Overall, our approach offers a powerful and convenient platform technology for patient-tailored cancer vaccines, which in combination with the immune checkpoint inhibitor can efficiently eliminate established tumors and prevent relapse.

### P348 Development of personalized, live, attenuated double-deleted Listeria monocytogenes (pLADD) immunotherapy targeting tumor-specific neoantigens to treat cancer

#### Weiwen Deng, Thomas E Hudson, Edward E Lemmens, Bill Hanson, Chris S Rae, Joel Burrill, Justin Skoble, George Katibah, Aimee L Murphy, Michele deVries, Dirk G Brockstedt, Meredith L Leong, Peter Lauer, Thomas W Dubensky, Chan C Whiting

##### Aduro Biotech, Inc., Berkeley, CA, USA

###### **Correspondence:** Meredith L Leong (mleong@aduro.com)

**Background**

Clinical success of checkpoint inhibitors, dendritic cell-based therapies and adoptive T cell transfer studies highlight the importance of tumor-specific neoantigens as critical targets for effective immunotherapy [1, 2]. Advances in genome sequencing and predictive epitope binding algorithms provide an unprecedented opportunity to develop highly personalized immunotherapeutics [3]. Aduro is developing personalized therapy (pLADD) based on the live, attenuated double-deleted *Listeria monocytogenes* (LADD) platform which has been administered to over 300 cancer patients with an acceptable safety profile.

**Methods**

Tumor-bearing C57BL/6 mice were treated with pLADD-MC38 and anti-PD-1. MC38 mutated epitopes [4] were expressed as a synthetic neoantigen protein from the *Listeria* chromosome. Tumor growth was monitored; immune responses measured by IFNg ELISpot.

**Results**

We constructed a pLADD strain expressing tumor-specific neoepitopes from murine MC38 tumor cells (pLADD-MC38). Administration of pLADD-MC38 in mice induces cellular immune responses against encoded neoepitopes but not against native sequences. Moreover, we also showed synergistic anti-tumor efficacy of pLADD-MC38 and anti-PD-1. To effectively translate pLADD into the clinic, we have established an accelerated, small-scale LADD manufacturing process.

**Conclusions**

pLADD is an attractive immunotherapy approach to target multiple tumor-specific neoantigens using the LADD platform to take advantage of rapid construction, manufacture, and release, an established clinical safety profile with repeated administration, and induction of innate and adaptive immunity and favorable tumor microenvironment changes. The FDA has cleared an IND for a phase I trial to evaluate safety and immunogenicity of pLADD in subjects with advanced gastrointestinal cancers, and its progress will be discussed.

**References**

1. Carreno BM, Magrini V, Becker-Hapak M, Kaabinejadian S, Hundal J, Petti AA, *et al.*: **A dendritic cell vaccine increases the breadth and diversity of melanoma neoantigen-specific T cells.** *Cancer Immunother* 2015, **348**:803–808.

2. Tran E, Turcotte S, Gros A, Robbins PF, Lu YC, Dudley ME, *et al.*: **Cancer immunotherapy based on mutation-specific CD4+ T cells in a patient with epithelial cancer.** *Science* 2014, **344**:641–645.

3. Schumacher TN, Schreiber RD: **Neoantigens in cancer immunotherapy.** *Science* 2015, **348**:69–73.

4. Yadav M, Jhunjhunwala S, Phung QT, Lupardus P, Tanguay J, Bumbaca S, *et al.*: **Predicting immunogenic tumour mutations by combining mass spectrometry and exome sequencing.** *Nature* 2014, **515**:572–576.

### P349 Therapeutic efficacy of cancer stem cell-vaccine in the PD-1/PD-L1 blockade

#### Xin Chen^1^, Yangyang Hu^2^, Yang Xia^2^, Li Zhou^1^, Yangyi Bao^3^, Shiang Huang^4^, Xiubao Ren^5^, Elaine Hurt^6^, Robert E Hollingsworth^6^, Alfred E Chang^2^, Max S Wicha^7^, Qiao Li^8^

##### ^1^University of Michigan Cancer Center, Ann Arbor, MI, USA; ^2^University of Michigan, Ann Arbor, MI, USA; ^3^The Third Affiliated Hospital of Anhui Medical University, Heifei, Anhui, People’s Republic of China; ^4^Union Hospital, Tongji Medical College, Huazhong University of Science and Technology, Wuhan, Hubei, People’s Republic of China; ^5^Tianjin University Cancer Institute and Hospital, Tianjin, Hebei, People’s Republic of China; ^6^MedImmune Inc., Gaithersburg, MD, USA; ^7^University of Michigan Medical School, Ann Arbor, MI, USA; ^8^University of Michigan Medical Center, 1150 W. Medical Center Dr., MI, USA

###### **Correspondence:** Qiao Li (qiaoli@umich.edu)

**Background**

We have previously reported the development of a strategy to target the cancer stem cell (CSC) populations in melanoma and squamous cell carcinoma using CSC lysate-pulsed dendritic cells (DCs). Using mouse models we demonstrated that DCs pulsed with CSCs enriched by virtue of their expression of the CSC marker ALDH (termed CSC-DC) significantly inhibited tumor growth. However, CSC-DC vaccine therapy alone may not be sufficient to overcome the immunosuppressive components of the tumor microenvironment. CSC-mediated suppression of T cells has been reported in a process involving the PD-L1/PD-1 axis. Immunologically targeting CSCs while simultaneously blocking PD-L1/PD-1-mediated immune suppression may significantly enhance the outcome of current immunotherapies of cancer.

**Methods**

To test this hypothesis, we investigated the effect of using anti-PD-L1 to block immunosuppression during the CSC-DC vaccination to augment the therapeutic efficacy of using each regimen alone. We used the 4 T1 cell line, a mammary carcinoma syngeneic to BALB/c mice, and found that 4 T1 tumors contain approx. 6-10 % ALDEFLUOR^+^/ALDH^high^ cells, and that these cells are enriched for tumor initiating capacity compared to bulk or ALDEFLUOR^−^/ALDH^low^ cells. Inoculating 4 T1 cells into the mammary fat pad induces the development of spontaneous pulmonary metastases.

**Results**

ALDH^high^ 4 T1 CSCs expressed PD-L1. 4 T1 ALDH^high^ CSC-DC vaccine + anti-PD-L1 administration significantly reduced pulmonary metastases and prolonged survival compared with 4 T1 ALDH^high^ CSC-DC vaccine or anti-PD-L1 administration alone. Anti-PD-L1 administration increased systemic anti-4 T1 CSC immunity induced by CSC-DC vaccine. This is evident by the increased production of total IgG in the serum samples collected from the 4 T1-bearing animals subjected to 4 T1 CSC-DC vaccination with anti-PD-L1 administration. The CSC-reactivity/specificity of the immune sera was demonstrated by their specific binging to the ALDH^high^ *vs*. ALDH^low^ 4 T1 cells in flow cytometry. Importantly, the immunological consequence of such binding was Ab-mediated ALDH^high^ 4 T1 CSC lysis via complement dependent cytotoxicity. In addition, CTLs generated from the splenocytes harvested from the hosts subjected to 4 T1 CSC-DC vaccination with anti-PD-L1 administration killed ALDH^high^ 4 T1 CSCs significantly more than ALDH^low^ 4 T1 cells.

**Conclusions**

These results are supportive of the conclusion that administration of anti-PD-L1 could significantly boost the therapeutic efficacy of the CSC-DC vaccine, and that this effect was due to both antibody and T cell-mediated anti-CSC immunity.

### P350 Immunotherapy with INO-3112 (plasmids encoding HPV16 and HPV18 E6/E7 antigens and IL-12) induces immune responses in peripheral blood and tumor tissue in patients with HPV associated head and neck squamous cell carcinoma (HNSCCa)

#### Charu Aggarwal^1^, Drishty Mangrolia^2^, Roger Cohen^1^, Gregory Weinstein^3^, Matthew Morrow^2^, Joshua Bauml^1^, Kim Kraynyak^2^, Jean Boyer^4^, Jian Yan^2^, Jessica Lee^2^, Laurent Humeau^4^, Sandra Oyola^2^, Susan Duff^2^, David Weiner^5^, Zane Yang^2^, Mark Bagarazzi^2^

##### ^1^University of Pennsylvania, Philadelphia, PA, USA; ^2^Inovio Pharmaceuticals, Plymouth Meeting, PA, USA; ^3^Inovio Pharmaceuticals, Philadelphia, PA, USA; ^4^Inovio Pharmaceuticals, San Diego, CA, USA; ^5^The Wistar Institute, Philadelphia, PA, USA

###### **Correspondence:** Drishty Mangrolia (dmangrolia@inovio.com)

**Background**

We have previously reported interim results of safety and immunogenicity of the INO-3112 in subjects with HPV-associated HNSCCa. INO-3112 was shown to be safe and immunogenic, inducing HPV-specific CD8+ T cell responses [1].

**Methods**

Subjects were enrolled into two cohorts. Cohort 1 received INO-3112 pre- and post-surgery. Cohort 2 received INO-3112 after completion of cisplatin based chemoradiation. Here, we report immune responses post immunotherapy in peripheral blood and tumor tissue obtained from surgery for Cohort 1 subjects. Tumor samples were stained with immunohistochemistry techniques for CD8 and FoxP3. In addition, ELISpot analysis was used to determine the number of cells capable of secreting IFN-γ in response to HPV antigen stimulation.

**Results**

As of August 1 2016, accrual has been completed with 22 enrolled subjects. Cohort 1: n = 6, Cohort 2: n = 16, 20 males, median age 57.5 years; base of tongue cancer = 10, tonsil cancer = 12; never smoker = 10. Six subjects in Cohort 1 received at least one dose of INO-3112 on average 14 days (range 7 to 28 days) prior to definitive surgery. Paired pre- and post-INO-3112 therapy tumor samples were available for five of the 6 subjects. CD8 positive T cell counts increased in tumor tissue in 2 subjects, average 160.6 % increase (range 61.7 % to 259.4 %) from baseline. FoxP3 positive cell counts decreased in tumor tissue in 3 subjects, average 48 % decrease (range 44 % to 53 %). Four of the 5 subjects showed increased CD8:FoxP3 ratio post INO-3112, average 60.3 % increase (range 1.4 % to 209.3 %). Five of 6 subjects had peripheral blood available for analysis of peripheral HPV-specific T cell responses by IFN-γ ELISpot. Four subjects exhibited an increase in ELISpot response magnitude post INO-3112 compared to baseline (range 30.00 to 158.33 SFU). Two subjects with increase in CD8 positive cells in tumor tissue demonstrated the highest increase in ELISpot response (108.33 and 158.33 SFU, respectively). Four of 6 subjects remain progression-free; median PFS of 17 months (range 12 to 23 months) to date. One subject withdrew consent after surgery. One subject demonstrated only marginal increases in ELISpot response magnitude to HPV 16 (3.33 to 16.67 SFU) and no increase in CD8/FoxP3 ratio (0.95 to 0.60) in tumor tissue post INO-3112 developed progressive disease (11 months post INO-3112).

**Conclusions**

These results demonstrate that INO-3112 DNA-based immunotherapy can induce detectable immune responses in peripheral blood and tumor tissue in subjects with HPV associated HNSCCa.

**Trial Registration**

ClinicalTrials.gov identifier NCT02163057.

**References**

1. *J Immunother Cancer* 2015, **3(Suppl 2)**:426.

### P351 DNA vaccine with pembrolizumab elicits anti-tumor responses in patients with metastatic, castration-resistant prostate cancer (mCRPC)

#### Douglas G McNeel^1^, Jens Eickhoff^2^, Robert Jeraj^2^, Mary Jane Staab^1^, Jane Straus^1^, Brian Rekoske^2^, Glenn Liu^1^

##### ^1^University of Wisconsin Carbone Cancer Center, Madison, WI, USA; ^2^University of Wisconsin, Madison, WI, USA

###### **Correspondence:** Douglas G McNeel (dm3@medicine.wisc.edu)

**Background**

In our evaluation of anti-tumor DNA vaccines as treatments for prostate cancer, we have recently shown in murine models that, depending on the duration of antigen expression encoded by the DNA and the strength of MHC:TCR affinity of CD8+ T cells elicited with vaccination, these T cells express higher levels of PD-1 or LAG-3 [1]. Blockade of these regulatory mechanisms at the time of T cell activation with vaccine produced anti-tumor responses *in vivo*. Similarly, we have recently found that patients with prostate cancer previously immunized with a DNA vaccine develop PD-1-regulated T cells [2]. These findings suggested that combined PD-1 blockade with vaccination should elicit superior anti-tumor responses in patients with prostate cancer.

**Methods**

A clinical trial was designed to evaluate the immunological and clinical efficacy of a DNA vaccine encoding PAP (pTVG-HP) when delivered in combination or in sequence with pembrolizumab, in patients with mCRPC. Serial biopsies, blood draws, and exploratory FLT PET/CT imaging are being conducted for correlative analyses.

**Results**

While trial accrual continues, 1 of 14 subjects has experienced a grade 3 adverse event. There have been no grade 4 events. 4 of 6 patients treated with the combination have experienced serum PSA declines, and 3 of 6 have experienced decreases in tumor volume by radiographic imaging at 12 weeks, including one partial response. Expansion of PAP-specific Th1-biased T cells has been detected in peripheral blood samples. Exploratory FLT PET/CT imaging has demonstrated proliferative responses in metastatic lesions and in vaccine-draining lymph nodes.

**Conclusions**

PD-1 pathway inhibitors have demonstrated little clinical activity to date when used as single agents for treating prostate cancer. Our findings suggest that combining this blockade with tumor-targeted T cell activation by a DNA vaccine is safe and can augment tumor-specific T cells, detectable within the peripheral blood and by imaging, and result in objective changes. We are currently exploring expansion of this trial to treat over an extended period of time and in an earlier stage of disease.

**Acknowledgements**

Funding from 2014 Movember Prostate Cancer Foundation Challenge Award and Madison Vaccines, Inc.

**Trial Registration**

ClinicalTrials.gov identifier NCT02499835.

**References**

1. Rekoske BT, Smith HA, Olson BM, Maricque BB, McNeel DG: **PD-1 or PD-L1 blockade restores antitumor efficacy following SSX2 epitope-modified DNA vaccine immunization**. *Cancer Immunol Res* 2015, **3**:946–955.2.

2. Rekoske BT, Olson BM, McNeel DG: **Anti-tumor vaccination of prostate cancer patients elicits PD-1/PD-L1 regulated antigen-specific immune responses.** *Oncoimmunology* 2016, **5**:e1165377.

### P352 A multipeptide vaccine plus toll-like receptor (TLR) agonists LPS or polyICLC in combination with incomplete Freund’s adjuvant (IFA) in melanoma patients

#### Marit Melssen^1^, Gina Petroni^1^, William Grosh^1^, Nikole Varhegyi^1^, Kim Bullock^1^, Mark E Smolkin^1^, Kelly Smith^1^, Nadejda Galeassi^1^, Donna H Deacon^2^, Elizabeth Gaughan^1^, Craig L Slingluff Jr^3^

##### ^1^University of Virginia, Charlottesville, VA, USA; ^2^Department of Surgery, University of Virginia, Charlottesville, VA, USA; ^3^Division of Surgical Oncology, University of Virginia, Charlottesville, VA, USA

###### **Correspondence:** Marit Melssen (mm2xz@virginia.edu)

**Background**

Adjuvants for cancer vaccines have not been optimized. In a murine model, vaccines with IFA may deplete circulating T cells, but vaccination with TLR agonists plus CD40 antibody induces strong, durable CD8 responses. An alternate approach to ligating CD40 is to activate CD4+ T cells, to upregulate CD40L. The present study tests safety and immunogenicity of vaccination with 12 Class I MHC-restricted melanoma peptides (12MP) to activate CD8+ T cells and a tetanus toxoid peptide (Tet) to activate CD4+ T cells, plus either of two TLR agonists, with or without IFA. We hypothesized that vaccines with TLR3 agonist polyICLC or TLR4 agonist lipopolysaccharide (LPS) would be safe and would induce stronger and more durable T cell responses than when IFA was included.

**Methods**

Participants with resected stage IIB-IV melanoma were randomly assigned 2:1 to cohort 1 (LPS dose-escalation, n = 33) or cohort 2 (polyICLC 1 mg, n = 18). Each cohort included 3 subgroups (a-c), receiving 12MP + Tet + TLR agonist (a) without IFA, (b) plus IFA in the first vaccine only (V1), or (c) plus IFA in all six vaccines (V6). Toxicities were recorded (CTCAE v4). T cell responses were measured with IFNγ ELISpot either *ex vivo*, or 14 days after *in vitro* stimulation (IVS).

**Results**

**Fig. 58 (abstract P352).**
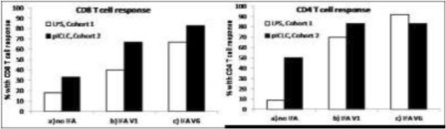
See text for description

There were no DLTs in Cohort 1 (LPS) but two in cohort 2 (1 of 6, subgroups 2b and 2c). CD8+ T cell responses to 12MP were detected *ex vivo* in 43 %, 67 %, 50 %, and 29 % of patients in Cohort 1 with 25, 100, 400, and 1600 EU LPS, respectively, and in 56 % of patients in Cohort 2. Responses to 12MP were detected *ex vivo* in 18 %, 50 %, and 78 % for subgroups (a)-(c), respectively (Fig. 58). Responses were more durable and of highest magnitude for IFA V6. IVS CD8 responses and *ex vivo* CD4 responses were also improved with addition of IFA.

**Conclusions**

LPS is a safe and effective vaccine adjuvant when combined with IFA; the optimal biologic dose may be 100–400 EU. All regimens were deemed safe. Despite recent concerns about IFA, this study demonstrates that in humans, IFA enhanced magnitude and durability of T cell responses to peptide vaccines when added to TLR agonists. Thus, combination strategies with IFA and LPS and/or pICLC offer promise for next generation vaccines.

**Trial Registration**

ClinicalTrials.gov identifier NCT0158535.

### P353 Long-term follow-up of Vigil (DNA engineered bi-shRNA furin GMCSF plasmid/autologous tumor) in recurrent metastatic Ewing’s sarcoma (EWS)

#### Maurizio Ghisoli^1^, Minal Barve^1^, Robert Mennel^2^, Gladice Wallraven^3^, Luisa Manning^4^, Neil Senzer^5^, John Nemunaitis^5^

##### ^1^Mary Crowley Cancer Research Centers, Texas Oncology, P.A., Dallas, TX, USA; ^2^Texas Oncology, P.A., Baylor University Medical Center, Dallas, TX, USA; ^3^Gradalis, Inc., Carrollton, TX, USA; ^4^Gradalis, Inc., Dallas, TX, USA; ^5^Mary Crowley Cancer Research Centers, Gradalis, Inc., Dallas, TX, USA

###### **Correspondence:** John Nemunaitis (jnemunaitis@marycrowley.org)

**Background**

EWS is an aggressive, rare (10 cases per million 10–19 year old children) pediatric cancer of bone and, less frequently, extraskeletal sites. Although first-line intensive chemotherapy has been effective in localized disease, it is less so in metastatic disease and poorly effective in patients with progressive or recurrent disease. Patients relapsing within 2 years of diagnosis, which occurs in 72 % of the patients, have a 2-year survival of 7 %. The outcome for refractory and third-line patients is even worse.

**Methods**

We recently completed a 3-year follow-up of a prospective, non-randomized study of Vigil vaccine (1x10e^6^ - 1x10e^7^ cells/ID injection 1x/mo) in recurrent/refractory EWS patients (n = 16) and compared results to a contemporaneous group (n = 14) not treated with Vigil (Table 5).

**Results**

**Fig. 59 (abstract P353).**
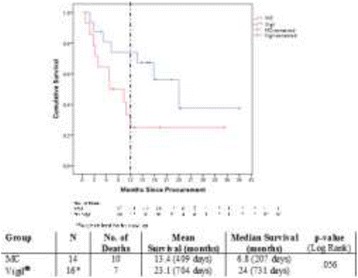
See text for description

Results suggest survival benefit without evidence of Vigil related toxicity (no ≥ grade 3). Specifically, we observed 1-year actual survival of 73 % for Vigil treated patients compared to 23 % in those not treated and a 17.2 month improvement in overall survival (Fig. 59).

**Conclusions**

In conclusion, Vigil appears to confer a survival advantage and enhanced therapeutic index in advanced EWS. A randomized multi-site study comparing Vigil vs. gemcitabine/Taxotere in third-line metastatic EWS has been initiated to see if these exploratory data can be confirmed (n = 62, HR 0.387).

**Table 5 (abstract P353).** Ewing’s Sarcoma Phase I Demographics

	Vigil®	Matched Comparator (MC)^a^
Tum or Location Harvest (Lung/Soft Tissue/Other)	13/0/3	11/2/1
Sex (M/F)	12/4	7/7
Age median (range)	19 (59–12)	17 (30–12)
Performance (ECOG 0, 1)	16	14
Ethnicity (Caucasian/Other)	13/3	12/2
Prior Systemic Tx (Frontline/2nd/=3rd)	1/5/10	3/4/7
General Surgery Harvest (Yes/No)	16/0	14/0

^a^3 insufficient viable tumor cells, 6 contaminants, 5 sought other management

### P354 Vaccination of advanced or relapsed prostate cancer patients with WT1 peptide-pulsed dendritic cells induces immunological and clinical responses

#### Masahiro Ogasawara, Shuichi Ota

##### Sapporo Hokuyu Hospital, Sapporo, Hokkaido, Japan

###### **Correspondence:** Masahiro Ogasawara (ogasawara@hokuyu-aoth.org)

**Background**

Survival of patients with advanced prostate cancer is significantly less than patients with early stage. Immunotherapy is a promising approach for the treatment of patients in advanced stage. In the current study, we have evaluated the clinical and immunological responses in patients with advanced or relapsed prostate cancer who received Wilms’ tumor 1 (WT1) peptide-pulsed dendritic cell (DC) vaccination in combination with a toll-like receptor (TLR) 4 agonist, OK432.

**Methods**

Twelve patients aged 57–82 years were enrolled in the present study. Autologous DCs were generated by culturing adherent mononuclear cells with interleukin-4 and granulocyte-macrophage colony stimulating factor. DCs were then loaded with synthetic peptides derived from WT1 following maturation by prostaglandin E_2_ and OK432. DCs and OK432 were administered intradermally every 2 weeks for 7 times. Induction of vaccine-induced T cell responses was evaluated using a HLA-tetramer assay, an intracellular cytokine staining assay and a flow cytometry analysis.

**Results**

The treatment was well tolerated and none of the patients experienced more than grade 2 adverse events. Of 12 patients, 7 had stable disease (SD) and 5 had disease progression after one course of vaccination. Survival of patients achieving SD after DC vaccination (responder) was longer than those who did not respond to the treatment (non-responder) (median duration of survival; 48 vs 10 months). Increase in positivity of WT1-specific CD8^+^ T cells was observed in both responders and non-responders after one course of vaccination. However, increment in positivity was marked in responders in comparison with non-responders; 53.5 and 2.1 fold in responders and non-responders, respectively. Similarly, intracellular IFNɤ staining assay showed that marked increase in WT1 specific IFNɤ-producing CD8^+^ T cells in responders compared with non-responders (68.2 vs 3.9 fold increase). Decrease in the absolute number of regulatory T cells (Tregs) or myeloid-derived suppressor cells (MDSCs) was observed in responders after vaccination. While the reduction in the absolute number of Tregs and monocytic MDSCs was moderate (9.0 % and 13.5 %, respectively), marked reduction in the absolute number of granulocytic MDSCs (61.0 %) was observed, indicating that DC vaccination may contribute to the reversal of immunosuppression by these cells.

**Conclusions**

DC vaccine-based immunotherapy combined with a TLR agonist was demonstrated to be safe and elicit both innate and acquired cellular immune responses correlated with clinical effects. These results suggest that DC vaccination might be a promising novel strategy for the treatment of patients with advanced or relapsed prostate cancer.

### P355 Phase Ib trial of two folate binding protein peptide booster vaccines (E39 and J65) in breast and ovarian cancer patients

#### Kaitlin M Peace^1^, Diane F Hale^1^, Timothy J Vreeland^2^, Doreen O Jackson^1^, John S Berry^3^, Alfred F Trappey^1^, Garth S Herbert^1^, Guy T Clifton^1^, Mark O Hardin^4^, Anne Toms^5^, Na Qiao^5^, Jennifer Litton^5^, George E Peoples^6^, Elizabeth A Mittendorf^5^

##### ^1^Brooke Army Medical Center, San Antonio, TX, USA; ^2^Womack Army Medical Center, Fayetteville, NC, USA; ^3^Department of Colon and Rectal Surgery, Washington University, St Louis, MO, USA; ^4^Madigan Army Medical Center, Tacoma, WA, USA; ^5^University of Texas MD Anderson Cancer Center, Houston, TX, USA; ^6^Cancer Vaccine Development Program, San Antonio, TX, USA

###### **Correspondence:** Kaitlin M Peace (kaitlin.peace@gmail.com)

**Background**

Folate binding protein (FBP) is over-expressed in multiple cancers. An immunogenic peptide (E39) and an attenuated version (J65) have been shown to stimulate cytotoxic T lymphocytes (CTLs) to recognize and destroy FBP-expressing cancer cells. In addition, previous trials have shown that boosting vaccinations helps maintain long-lasting immunity, though attenuated peptides may be a better choice for boosting due to antigen-induced cell death (AICD) of CTLs after over-stimulation. Here, we report peptide-specific immune response to E39 and J65 after different combinations of vaccination and boosting.

**Methods**

This is a prospective, randomized, non-blinded, single-center phase Ib trial. Patients with breast or ovarian cancer rendered disease-free after standard-of-care therapy were enrolled. HLA-A2+ patients were stratified (breast versus ovarian), and for the primary vaccine series (PVS) received either six inoculations with E39, three E39, then three J65 or three J65, then three E39. *Ex vivo* immunologic recognition of E39 was assessed by clonal expansion of cytotoxic T lymphocytes (CTL) and *in vivo* response by delayed-type hypersensitivity (DTH). The 6-month post-PVS immunologic data was used to assess patients for significant residual immunity (SRI), defined as ≥2-fold increase from pre-PVS in E39-specific CD8 + T cells. Patients were sorted into two groups: with SRI (SRI) and without (nSRI). Patients within each group were randomized to one booster of either J65/E39 resulting in four groups: SRI receiving E39 (SRI-E39), SRI receiving J65 (SRI-J65), nSRI receiving E39 (nSRI-E39), nSRI receiving J65 (nSRI-J65). Immunologic data was gathered at 1- and 6-months post-booster. This immunologic data was then analyzed.

**Results**

28 patients were randomized to booster arms (SRI-E39:n = 9; SRI-J65:n = 7; nSRI-E39:n = 7; nSRI-J65:n = 5). There were no clinicopathologic differences between groups. All related adverse events were grade 1–2. When comparing DTH pre-booster and at 1 and 6-months post-booster there were no significant differences between SRI vs nSRI (p = 0.350, p = 0.276, p = 0.133, respectively), E39 vs. J65 (p = 0.270, p = 0.329, p = 0.228), nor between all four groups (p = 0.394, p = 0.555, p = 0.191). Comparing delta-CTL from pre- and 6-months post-booster, regardless of SRI, patients boosted with J65 had increased CTL (+0.02) while those boosted with E39 had decreased CTL (−0.07, p = 0.077). There was no difference comparing delta-DTH between groups (p = 0.927).

**Conclusions**

Both E39 and J65 are safe, well tolerated boosters. Though numbers were small, patients boosted with the attenuated peptide did appear to have increased CTL response to boosting regardless of SRI after the PVS. This is consistent with the theoretical advantage of boosting with an attenuated peptide, which has a maintained E39 specific immunity.

**Trial Registration**

ClinicalTrials.gov identifier NCT02019524.

### P356 Genome-scale neoantigen screening using ATLAS™ prioritizes candidates for immunotherapy in a non-small cell lung cancer patient

#### Lila Ghamsari^1^, Emilio Flano^1^, Judy Jacques^1^, Biao Liu^1^, Jonathan Havel^2^, Vladimir Makarov^2^, Taha Merghoub^3^, Jedd D Wolchok^4^, Matthew D Hellmann^4^, Timothy A Chan^2^, Jessica B Flechtner^1^

##### ^1^Genocea Biosciences, Cambridge, MA, USA; ^2^Memorial Sloan Kettering Cancer Center, New York, NY, USA; ^3^Ludwig Collaborative Laboratory, Memorial Sloan Kettering Cancer Center, New York, NY, USA; ^4^Department of Medicine, Memorial Sloan Kettering Cancer Center, New York, NY, USA

###### **Correspondence:** Jessica B Flechtner (jessica.flechtner@genocea.com)

**Background**

Despite the unprecedented efficacy of checkpoint inhibitor (CPI) therapy in treating some cancers, the majority of patients fail to respond. Several lines of evidence support that the mutational burden of the tumor influences the outcome of CPI therapies. Capitalizing on neoantigens derived from non-synonymous somatic mutations may be a good strategy for therapeutic immunization. Current approaches to neoantigen prioritization involve mutanome sequencing, *in silico* epitope prediction algorithms, and experimental validation of cancer neoepitopes. We sought to circumvent some of the limitations of prediction algorithms by prioritizing neoantigens empirically using ATLAS™, a technology developed to screen T cell responses from any subject against their entire complement of potential neoantigens.

**Methods**

Exome sequences were obtained from peripheral blood mononuclear cells (PBMC) and tumor biopsies from a non-small cell lung cancer patient who had been successfully treated with pembrolizumab. The tumor exome was sequenced and somatic mutations identified. Individual DNA sequences (399 nucleotides) spanning each mutation site were built, cloned and expressed in *E. coli* co-expressing listeriolysin O. Polypeptide expression was validated using a surrogate T cell assay or by Western blotting. Frozen PBMCs, collected pre- and post-therapy, were used to derive dendritic cells (MDDC), and CD8^+^ T cells were enriched and expanded using microbeads. The *E. coli* clones were pulsed onto MDDC in an ordered array, then co-cultured with CD8^+^ T cells overnight. T cell activation was detected by analyzing cytokines in supernatants. Antigens were identified as clones that induced a cytokine response that exceeded 3 standard deviations of the mean of ten negative controls, then their identities compared with T cell epitopes predicted using previously described algorithms.

**Results**

Peripheral CD8^+^ T cells, screened against 100 mutated polypeptides derived from the patient’s tumor, were responsive to five neoantigens prior to CPI intervention and seven post-treatment. One was identified as a T cell target both pre- and post-CPI therapy. Five neoantigens did not contain epitopes predicted by *in silico* **methods**.

**Conclusions**

These data represent evidence that multiple patient-specific neoantigens can be identified through functional evidence of T cell response from peripheral blood without epitope prediction. By profiling natural and CPI-enhanced immunity to neoantigens, a broad catalog of T cell targets can be identified for development of immunotherapies that engage T cells against cancer to improve outcomes for patients for whom current therapies are insufficient.

### P357 Targeting tumor vasculature with a DNA vaccine against endosialin (TEM1 or CD248)

#### Pierini Stefano, Andrea Facciabene, John Facciponte, Stefano Ugel, Francesco De Sanctis, George Coukos

##### University of Pennsylvania, Philadelphia, PA, USA

###### **Correspondence:** Pierini Stefano (pierinis@mail.med.upenn.edu)

**Background**

Tumor endothelial marker 1 (TEM1; also known as endosialin or CD248) is a protein found on tumor vasculature and in tumor stroma.

**Methods**

Here, we tested whether TEM1 has potential as a therapeutic target for cancer immunotherapy by immunizing immunocompetent mice with Tem1 cDNA fused to the minimal domain of the C fragment of tetanus toxoid (referred to herein as Tem1-TT vaccine).

**Results**

Tem1-TT vaccination elicited CD8+ and/or CD4+ T cell responses against immunodominant TEM1 protein sequences. Prophylactic immunization of animals with Tem1-TT prevented or delayed tumor formation in several murine tumor models. Therapeutic vaccination of tumor-bearing mice reduced tumor vascularity, increased infiltration of CD3+ T cells into the tumor, and controlled progression of established tumors. Tem1-TT vaccination also elicited CD8+ cytotoxic T cell responses against murine tumor-specific antigens. Effective Tem1-TT vaccination did not affect angiogenesis-dependent physiological processes, including wound healing and reproduction.

**Conclusions**

Based on these data and the widespread expression of TEM1 on the vasculature of different tumor types, we conclude that targeting TEM1 has therapeutic potential in cancer immunotherapy.

### P358 Hafnium oxide nanoparticle, a radiation enhancer for in situ cancer vaccine

#### Sébastien Paris^1^, Agnes Pottier^1^, Laurent Levy^1^, Bo Lu^2^

##### ^1^Nanobiotix, Paris, Ile-de-France, France; ^2^Thomas Jefferson University, Philadelphia, PA, USA

###### **Correspondence:** Agnes Pottier (agnes.pottier@nanobiotix.com)

**Background**

**Fig. 60 (abstract P358).**
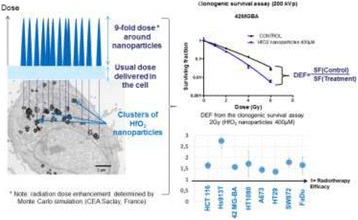
HfO2 nanoparticles: same mode of action than radiotherapy, but amplified

Effective immunotherapy requires optimal combination of immunotherapeutic agents to build a robust immune response against cancer. In this framework, radiotherapy has proven its ability to induce immunogenic cell death (ICD), showing a promising potential for successful combination. Hafnium oxide (HfO_2_) nanoparticles, undergoing clinical trials for enhancing radiotherapy, was designed as high electron density material at the nanoscale to enhance the absorption of radiation delivered within tumors. The nanoparticles are taken up by cancer cells and, when exposed to radiotherapy, locally increase the radiation dose deposit, triggering more cancer cells death when compared to radiotherapy alone (Fig. 60).

**Methods**

Generation of ICD components – namely calreticulin (CALR) surface exposure, release of high mobility group box 1 (HMGB1) protein and liberation of adenosine-5’-triphosphate (ATP) – were examined on human cancer cell lines across human cancer types, 24- to 96-hrs post-treatment with HfO_2_ nanoparticles and exposure to irradiation (from 4Gy to 15 Gy). CT 26 (murine colorectal cancer cells) treated with or without HfO_2_ nanoparticles were exposed to irradiation (6Gy). Irradiated cells (1.10^6^) were inoculated subcutaneously into the flank of BALB/c mice (vaccination phase). Seven days after, mice were challenged with live CT 26 tumor cells (3.10^5^) (challenge phase). The host immune response against these cells was evaluated by the apparition of at least one tumor (vaccination or challenge site).

**Results**

*In vitro*, human cancer cell lines treated with HfO_2_ nanoparticles exposed to irradiation enhanced the quantity of ICD (more than 25 %) when compared to irradiation alone. Interestingly, in tested human cell lines HCT116 (radiosensitive colorectal cancer) and 42MGBA (radioresistant glioblastoma), the generation of HMGB1 from cells treated with HfO_2_ nanoparticles and exposed to 4Gy and 10Gy respectively, was superior to the generation of ICD from cells treated with 6Gy and 15Gy alone respectively. *In vivo*, the percentage of mice protected against live CT 26 challenge was markedly increased for mice vaccinated with cells treated with HfO_2_ nanoparticles exposed to 6Gy versus 6Gy alone (66 % vs 33 % respectively).

**Conclusions**

HfO_2_ nanoparticles exposed to irradiation enhanced cancer cells destruction and ICD compared to irradiation alone, suggesting a strong potential for transforming tumor into an effective *in situ* vaccine. They may contribute to transform “cold” tumor into “hot” tumor and effectively be combined with most of the immunotherapeutic agents across oncology.

### P359 5 T4 oncofetal protein – an old antigen for a novel prostate cancer vaccine

#### Federica Cappuccini^1^, Emily Pollock^1^, Richard Bryant^2^, Freddie Hamdy^2^, Adrian Hill^1^, Irina Redchenko^1^

##### ^1^The Jenner Institute/University of Oxford, Oxford, England, UK; ^2^Nuffield Department of Surgical Sciences/University of Oxford, Oxford, England, UK

###### **Correspondence:** Irina Redchenko(irina.redchenko@ndm.ox.ac.uk)

**Background**

Prostate cancer is the cancer type for which the first therapeutic vaccine was approved by the FDA. Sipuleucel-T is a personalized cell based immunotherapy that costs $93,000 per patient and prolongs life for 4.1 months. Another most clinically advanced prostate cancer vaccine, ProstVac-VF, is based on the two replication competent viral vectors, vaccinia and fowlpox. A global phase III trial of this vaccine has completed enrollment and the results are eagerly awaited by the scientific community. Both Sipuleucel-T and ProstVac-VF were shown to induce cellular immune responses but the responses were of relatively low magnitude, which could be an underlying cause of the modest clinical benefit.

**Methods**

We set out to evaluate an alternative viral based vaccination approach as a novel prostate cancer immunotherapy. The scientific rationale for this endeavor has been underpinned by numerous studies conducted at the Jenner Institute research laboratories over the past decade. They have demonstrated that a prime boost vaccination regime based on two replication deficient viruses - the simian adenovirus and modified vaccinia Ankara virus, MVA, is the most potent strategy for induction of strong, poly-functional, durable and protective cellular immune responses in infectious disease setting. To test this vaccination platform in cancer settings, simian adenovirus, ChAdOx1, and MVA were engineered to express 5 T4 - the tumor-associated antigen that has been previously targeted clinically by homologous vaccinations in a number of tumor types including colorectal, renal and prostate cancer.

**Results**

Following ChAdOx1.5 T4-MVA.5 T4 vaccination, the mice mounted strong T cell responses against 5 T4 and were completely protected against subsequent tumor challenge with the syngeneic B16 melanoma cell line expressing 5 T4. The vaccine was also protective in therapeutic settings delaying progression of already established tumors in vaccinated mice. The ChAd-MVA vaccination platform significantly outperformed 5 T4 targeting homologous vaccinations previously tested by other researchers in terms of both immunogenicity and efficacy. Strikingly, a combination of ChAd-MVA vaccine with anti-PD-1 mAb resulted in 80 % of mice remaining tumor-free while all the control animals succumbed to tumors in this highly aggressive cancer model.

**Conclusions**

Our preclinical data have supported further clinical development of the novel prostate cancer vaccine. Recruitment is currently underway in the UK to test ChAdOx1.5 T4-MVA.5 T4 vaccination regime in a first-in-human “window” trial in low and intermediate risk prostate cancer patients. Preliminary immunogenicity and efficacy data are expected later on this year.

**Acknowledgements**

This work was supported by the European Union’s Seventh Framework Programme under Grant Agreement No. 602705.

**Trial Registration**

ClinicalTrials.gov identifier NCT02390063.

### P360 Peptide vaccines/IL2 complex combination expands high quality endogenous T cell responses that eradicate tumors

#### Hussein Sultan, Takumi Kumai, Valentyna Fesenkova, Esteban Celis

##### Augusta University, Georgia Cancer Center, Augusta, GA, USA

###### **Correspondence:** Hussein Sultan (hsultan@augusta.edu)

**Background**

Cancer vaccines, that generate tumor-reactive cytotoxic T lymphocyte (TR-CTL) responses are promising approach in cancer treatment. Unfortunately, most cancer vaccines induce suboptimal CTL responses (both in the quality and quantity), which are not sufficient to eradicate established tumors. In contrast, CTL adoptive cell therapy (ACT) has shown in many instances great therapeutic success but this therapy is not cost effective and remains technically challenging. We hypothesize that expansion of high quality endogenous TR-CTLs using peptide vaccines will circumvent the technical difficulties of ACT and boost the antitumor efficacy in a cost effective manner.

**Methods**

Our lab developed a novel vaccination strategy using peptides from tumor-associated antigens and poly-IC (BiVax), which showed promising antitumor effects [1]. Mice were injected with BiVax (120 μg of peptide and 50 μg of Poly-IC) on day 0 and 12. Mouse IL-2cx_CD25_, IL-2cx_CD122_, or IL-2Fc (20 μg/mouse) was injected intraperitoneally on day 12, 14, and 16. In some mice, cytokines were injected on day 1 to 4.

**Results**

In this study, we further improved the efficacy of BiVax by utilizing IL-2/anti-IL-2 antibody complexes (IL-2cx). The combination of BiVax with IL-2cx (BiVax_IL-2cx_) induced a robust amount of endogenous TR-CTLs (~40 million TR-CTLs/spleen) in a peptide dose-dependent manner. These cells were able to recognize tumor *in vitro* as shown by ELISPOT assay. Moreover, BiVax_IL-2cx_-expanded TR-CTLs were able to significantly delay B16F10 melanoma growth, enhance the survival of the tumor bearing mice, and eradicate tumors in 20 % of mice. The timing for IL-2cx administration was critical, thus the activation of T cells by peptide vaccines before cytokine administration was crucial to expand the TR-CTLs.

**Conclusions**

In conclusion, our data showed that peptide vaccines have the ability to expand huge number of TR-CTLs with good quality that able to control and in some instances eradicate aggressive tumors. Moreover, the adjuvant and its timing of administration are critical in expanding the TR-CTLs by peptide vaccines. Finally, our findings may pave the way for the development of promising immunologic approach for cancer treatment, which may circumvent lymphodepletion in ACT therapy and enhance the checkpoint blockade inhibitors treatment.

**References**

1. Cho HI, Barrios K, Lee YR, Linowski AK, Celis E: **BiVax: a peptide/poly-IC subunit vaccine that mimics an acute infection elicits vast and effective anti-tumor CD8 T-cell responses.** *Cancer Immunol Immunother*. 2013, **62(4):**787**–**799.

**Fig. 61 (abstract P360).**
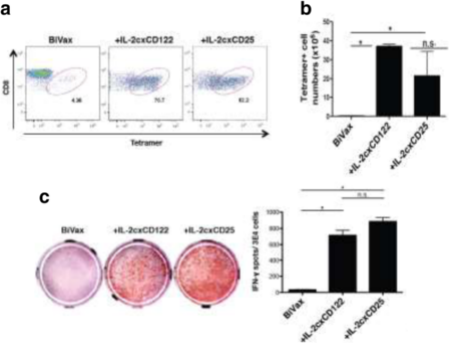
The combination of BiVax with IL-2cx induced a robust amount of endogenous TR-CTLs. **a** C57BL/6 mice were immunized with BiVax on day 0 and 12. IL-2 complexCD122 or IL-2 complexCD25 was administered on day 12, 14, and 16 in the indicated group. The percentage of (TAPDNLGYM) Trp1-tetramer+ cells in blood CD8+ T cells was examined on day 19 (boost). Images of the representative results on day 19 are shown. **b** On day 19, the number of Trp1-tetramer+ cells in spleen was examined. **c** Purified CD8+ T cells from vaccinated mice were used in ELISpot assay. T cells were cultured with B16F10 melanoma cells for overnight. Results are presented as mean ± SD. (*p<0.05, n.s.: not significant)

**Fig. 62 (abstract P360).**
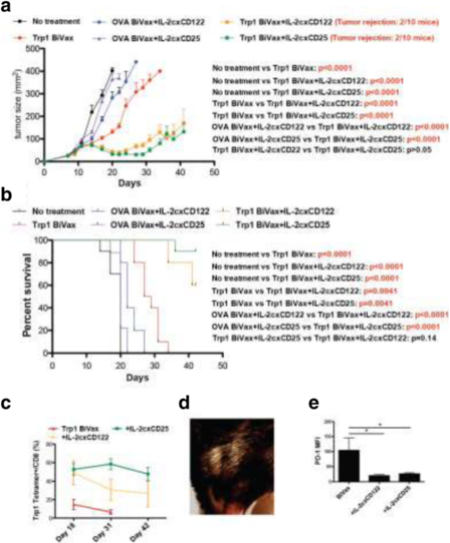
The therapeutic effects of IL-2 complex with peptide vaccine. **a**-**e** C57BL/6 mice were inoculated with B16F10 melanoma cells (5 x 10^5^ cells/mouse). After 7 days, mice received BiVax twice (day 7 and 12). IL-2 complexCD122 or IL-2 complexCD25 was administered on day 12, 14, and 16. **a** The mean sizes of tumor and (**b**) the overall survival of tumor-bearing mice are depicted. **c** The percentage of Trp1-tetramer+ cells in CD8+ T cells was examined on day 18 and 31. **d** The representative image of vitiligo at the tumor-inoculated lesion in the mouse, which received BiVax and IL-2 complexCD25 (day 30). **e** The expression of PD-1 on Trp1-tetramer+ CD8+ T cells was assessed on day 31. Results are presented as mean ± SD. (*p<0.05, n.s.: not significant)

**Fig. 63 (abstract P360).**
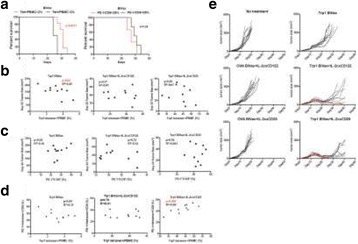
The antitumor effects of BiVax correlate with the percentage of Trp1-tetramer positive CD8+ T cells. C57BL/6 mice were inoculated with B16F10 melanoma cells (5 x 10^5^ cells/mouse). After 7 days, mice received BiVax twice on day 7 and 12 and IL-2 complexCD122 or IL-2 complexCD25 was administered on day 12, 14, and 16. **a** The percentage survival of Trp1-BiVax-received mice. **b** The Pearson correlation analysis of the tumor size (day 22) and the percentage of Trp1-tetramer+ cells in PBMC. **c** The Pearson correlation analysis of the tumor size (day 22) and the percentage of PD-1+ cells in PBMC. **d** The Pearson correlation analysis of the percentage of Trp1-tetramer+ cells and the percentage of PD-1+ cells in PBMC. **e** The sizes of individual tumor are demonstrated

**Fig. 64 (abstract P360).**
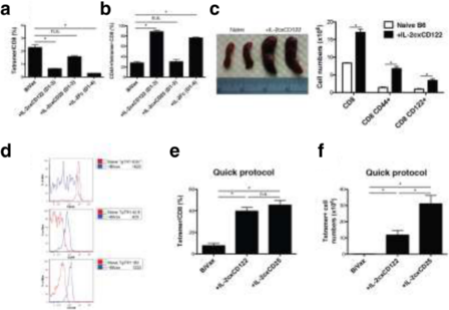
Proper timing of IL-2 complex administration is necessary to induce CD8+ T cell responses. **a** C57BL/6 mice were injected with BiVax on day 0 combined with IL-2 complexCD122 (day 1, 2, and 3), IL-2 complexCD25 (day 1, 2, and 3) or IL-2Fc (day 1 to 4). The percentage of tetramer+ cells in CD8+ T cell or (**b**) CD44+ T cells in Trp1-tetramer- CD8+ T cells was examined in blood on day 7. **c** C57BL/6 mice received IL-2 complexCD122 on day 0, 2, and 4 and the number of CD44+ or CD122+ cells in spleen was examined on day 7. The picture of spleen after the treatment is shown. **d** C57BL/6 mice received TgTR1 cells (2000 cells/mouse) and BiVax. After 7 days, the expression of blood CD62L, CD44, and CD122 on activated TgTR1 cells were compared to naïve TgTR1 cells. **b** C57BL/6 mice were injected with BiVax on day 0 and 5. IL-2 complexCD122 or IL-2 complexCD25 was administered on day 5, 7, and 9. The percentages of tetramer+ cells in blood CD8+ T cells and (**f**) the numbers of tetramer+ cells in spleen on day 12 are shown. Results are presented as mean ± SD. (*p<0.05, n.s.: not significant)

### P361 Identification and characterization of agonist human cytotoxic T cell epitopes of the human papillomavirus type 16 (HPV-16) E6/E7

#### Kwong Tsang^1^, Massimo Fantini^1^, Ingrid Fernando^1^, Claudia Palena^1^, Justin M David^1^, James Hodge^1^, Elizabeth Gabitzsch^2^, Frank Jones^2^, James L Gulley^3^, Jeffrey Schlom^4^

##### ^1^Laboratory of Tumor Immunology and Biology, National Cancer Institute, National Institutes of Health, Bethesda, MD, USA; ^2^Etubics Corporation, Seattle, WA, USA; ^3^Genitourinary Malignancies Branch, Center for Cancer Research, National Cancer Institute, National Institutes of Health, Bethesda, MD, USA; ^4^Center for Cancer Research, National Cancer Institute, National Institutes of Health, Bethesda, MD, USA

###### **Correspondence:** Kwong Tsang (ktsang13@gmail.com)

**Background**

Human papillomavirus (HPV) type 16 is associated with the etiology of cervical cancer, head and neck squamous cell carcinoma (HNSCC), and many other HPV-associated tumors. Current HPV-16 vaccines utilize viral coat proteins or virus-like particles with HPV-16 late gene products. Many HNSCC express early E6/E7 rather than late viral genes such as the viral coat proteins. Thus, vaccines that use late viral proteins may not be effective in treating established tumors. E6/E7, the early proteins of HPV-16, have a transforming capacity. They interfere with cell-cycle control of infected cells and are essential for maintaining the transformed state. An immune-based therapeutic vaccine that targets E6/E7 may prove more effective than a late viral protein vaccine. Identifying and characterizing MHC class I-restricted immunogenic peptides derived from E6/E7 proteins is essential for designing and developing vaccines to treat HPV-16-induced carcinomas, and for monitoring clinical trials and immunotherapeutic approaches for the treatment of these tumors.

**Methods**

We report here the development of immunogenic HLA-A*0201-restricted 9-mer epitopes and agonist epitopes of E6 and E7. We selected two E6- and one E7-derived peptide epitope and the corresponding agonist epitopes with high affinities for HLA-A*0201 molecules. The immunogenicity of these six peptides was evaluated by their ability to activate T cell lines generated from human dendritic cells infected with the Ad5 [E1-, E2b-]–E6 Δ/E7Δ vector and normal human peripheral blood mononuclear cells (PBMC). The Ad5 [E1-, E2b-]–E6 Δ/E7Δ vector contains mutations that render E6/E7 nononcogenic, while preserving antigenicity.

**Results**

Our results show that these peptide-pulsed dendritic cells, as well as Ad5 [E1-, E2b-]–E6 Δ/E7Δ vector-infected dendritic cells, can activate T cell lines generated from human dendritic cells infected with the Ad5 [E1-, E2b-]–E6 Δ/E7Δ vector. Compared to native peptides, the agonist peptides more efficiently (1) enhanced the production of IFN-γ by peptide-activated human T cells and (2) lysed human tumor cells expressing HPV in an MHC-restricted manner. These agonist peptides are highly immunogenic.

**Conclusions**

These studies provide a rationale for the incorporation of these agonist epitopes into therapeutic vaccine platforms and for the *ex vivo* generation of HPV-specific human T cells.

### P362 Liposome-encapsulated doxorubicin is a promising adjuvant to increase the efficacy of mTERT DNA-vaccine

#### Mireia Uribe Herranz, Stavros Rafail, Stefano Ugel, John Facciponte, Pierini Stefano, Andrea Facciabene

##### University of Pennsylvania, Philadelphia, PA, USA

###### **Correspondence:** Mireia Uribe Herranz (mireiau@upenn.edu)

**Background**

Challenging the notion that chemotherapy negatively modulates the immune system of tumor-bearing hosts, recent evidence on the contrary indicates that some cytotoxic drugs control tumor growth in part by facilitating an anti-tumor immune response. The precise mechanism(s) that controls this phenomenon have not been elucidated. Chemotherapy, especially at low doses, may modify the host’s immune system by either augmenting antigen-specific effector cells by rendering tumor cells immunogenic or eliminating immune-suppressive cell populations that limit the anti-tumor immune effect. Doxil (pegylated liposomal doxorubicin) possesses specific immunomodulatory properties such as inducing immunogenic tumor cell apoptosis.

**Methods**

Mice were injected intraperitoneally (i.p.) with 5 x10^6^ ID8 cells. For chemotherapeutic treatment, mice received a single i.p. injection of either 50 mg/m^2^ of Doxil (doxorubicin HCl liposome injection) or 50 mg/m^2^ of doxorubicin. DNA immunization (mTERT-LTB) was performed according to commonly used protocols: 50 micrograms of plasmid DNA was injected into mice quadriceps and then electroporation was carried out with a BTX electroporator intramuscularly at the injection site. For natural killer (NK) cell depletion, tumor-free and ID8 tumor-bearing mice were treated with anti-asialo GM1.

**Results**

Here, we characterize how Doxil treatment is able to improve both the tumor-free and tumor-bearing host immune system by expanding NK cell populations after 5 days from the time of drug administration. Moreover, NK cells isolated from Doxil-treated mice produce greater amounts of interferon (IFN)-γ compared to isolated NK cells from untreated mice, promoting selective Th-1 polarization of naïve CD4+ T cells. These immune modifications mediated by chemotherapy ameliorates the capability of a DNA-vaccine to select and expand an antigen-specific CD8 + T cell population. This synergistic effect between chemotherapy and vaccination was completely mediated by NK cell expansion; in fact, the *in vivo* depletion of this cell subset totally abrogated the Doxil immune adjuvant activity. We combined Doxil with a DNA-vaccine encoding mouse telomerase reverse transcriptase (TERT).

**Conclusions**

TERT is an attractive target antigen for cancer vaccine because its expression is reactivated in tumors of different histology such as ovarian cancer. We verified different vaccination schedules in ID8 ovarian tumor-bearing mice and only combinations that resulted in significant tumor growth inhibition were related to a specific anti-TERT CD8+ T cell response. This data demonstrates “chemo-immune adjuvancy” of a conventional drug and highlights the importance to define the precise time window between treatments to improve their therapeutic synergism.

### P363 Efficient design method for pan-HLA multi-valent cancer vaccine

#### Hiroshi Wada, Atsushi Shimizu, Toshihiro Osada, Satoshi Fukaya, Eiji Sasaki

##### Taiho Pharmaceutical Co., Ltd, Tsukuba, Ibaraki, Japan

###### **Correspondence:** Hiroshi Wada (h-wada@taiho.co.jp)

**Background**

Recently, a lot of tumor antigen peptides are examined for clinical applications. The treatment strategy using multiple peptides is expected to achieve better outcomes than single peptide treatment in terms of HLA restriction. In addition, many advantages administering the synthetic long peptides (SLPs) are reported. Based on this information, and aiming for improvement of popularity coverage, we planned to design SLP vaccines containing multiple cytotoxic T lymphocyte (CTL) epitopes which are restricted HLA-A2, A24, A3 supertype, respectively. However, there are few screening strategies to confirm whether designed SLP vaccines could induce all epitopes specific-CTLs in humans except confirmations using human PBMCs or expensive HLA-expressing mice. In order to improve this issue, we performed the following screening procedures.

**Methods**

Murine-immunoproteasome digestion assay was conducted as follows. Briefly, SLPs were dissolved with buffer and incubated with murine i20s immunoproteasome for 1 h, 2 h and 4 h. Then, digested peptides were separated using an UPLC system, and the eluent was analyzed by mass spectrometry. The sequences of the digested peptides were assigned based on the results of their m/z. Finally, the “digestion maps” were drawn by rearranging digested peptides fragments from N-terminal. In order to compare the digestion maps to the peptide specific CTL induction, the SLPs were administered to HLA-A knock-in mice and the CTL induction was analyzed using IFN-γ ELISPOT assay.

**Results**

We applied 4 SLPs to the murine-immunoproteasome digestion assay. Immunoproteasomal degradations were observed in all SLPs and they were time-dependent. In this study, we focused on the generations of intact epitopes, Arg added epitopes, and Arg-Arg added epitopes to the N-terminus at any time points because proteasomal digestion of exact C-terminus of CTL epitope is known to be an essential process for the antigen presentation. Regarding this criteria, 8 epitopes were determined as a “living epitope,” which would be able to induce specific CTLs, but 4 epitopes were determined as a “dead epitope”. Compared with the IFN-γ ELISPOT assay, the results of murine-immunoproteasome digestion assay were substantially matched the results of CTL induction (11 of 12 epitopes: 91.7 %).

**Conclusions**

Murine-immunoproteasome digestion assay could predict the CTL induction with a high degree of accuracy. We concluded that murine-immunoproteasome digestion assay strategy could be a prognostic approach for IFN-γ ELISPOT assay using an HLA-expressing mice model. In addition, our results suggested that the positions of each epitope peptide are important for the design of SLP vaccines. Murine-immunoproteasome digestion assay is very useful to develop “suitable” multi-valent SLP vaccines.

## Tumor Microenvironment

### P364 Comparative analysis of frequency, phenotypic profile and transcriptome of dendritic cell (DC) subsets in tonsillar cancer (TC) and benign tonsils

#### Milad Abolhalaj^1^, David Askmyr^2^, Kristina Lundberg^1^, Ann-Sofie Albrekt^1^, Lennart Greiff^2^, Malin Lindstedt^1^

##### ^1^Department of Immunotechnology, Lund University, Lund, Skane Lan, Sweden; ^2^ENT Department, Lund University Hospital, Lund, Skane Lan, Sweden

###### **Correspondence:** Milad Abolhalaj (milad.abolhalaj@immun.lth.se)

**Background**

Head and neck cancer (HNC), including TC, is the 6^th^ most common group of cancers worldwide. Survival rates with conventional treatments are unsatisfactory and treatment-associated side effects marked, necessitating development of novel therapeutic approaches. Arguably, DC-mediated immunotherapy is one such option, owing to the outstanding potential of DCs to elicit tumor-specific immune responses. Our work presents frequency as well as transcriptional and phenotypical assessments of myeloid and plasmacytoid DC subsets in TC and benign tonsils to fulfill a descriptive need of DC characterization in DC-mediated immunotherapy context.

**Methods**

From biopsies of TC tissue (n = 4) and benign tonsils (n = 4), DC subsets were identified and sorted through an 8-color flow cytometry Ab panel. Sorted cells were run on a human transcriptome array and gene expression profiles of the subsets in TC were compared to their peers with benign tonsils. Two fold upregulated subset-specific gene signatures were determined by the intersection derived from separate two-group comparison tests against other subsets. They were then analyzed and candidate genes were extracted based on association with cross-presentation, endocytosis, and signaling activities. A set of selected candidate markers was investigated at the protein level through flow cytometry (approximately 15 benign and 10 TC samples).

**Results**

DCs were more frequent among CD45^+^ leukocytes in TC compared to benign tonsils and showed an elevated myeloid CD11c^+^/plasmacytoid CD123^+^ ratio. In agreement, some statistically significant changes were observed in different subsets where CD123^+^ DCs were less frequent in TC compared to benign tonsils while CD1c^−^ CD141^−^ DCs were more frequent. In contrast, no significant differences were detected in DC subsets’ gene expression profiles between TC and benign tonsils. Lists of subset-specific candidate genes were generated and some of them were confirmed at protein level, e.g., CD206 and CD207 on the CD1c^+^ DCs in TC and benign tonsils.

**Conclusions**

DCs are present in TC and benign tonsil tissue with somewhat different subset ratios but similar transcriptional and phenotypic profiles. Surprisingly, DCs may therefore not be markedly affected by the microenvironment in TC (as may be the case for many other cancers). Accordingly, our work suggests a maintained DC functionality and potentially a unique possibility of tailored DC-mediated immunotherapy for TC. This is now facilitated by our present description of subset-selective target molecules for induction of favored cell-mediated anti-tumor responses. Further functional studies are warranted and whether our findings extend to other HNCs remains to be examined.

### P365 IL-10 blockade sensitizes ovarian cancer to anti-PD-1 antibody therapy by editing tumor-associated leukocyte populations

#### Dallas B Flies^1^, Tomoe Higuchi^2^, Wojciech Ornatowski^3^, Jaryse Harris^4^, Sarah F Adams^3^

##### ^1^NextCure, Beltsville, MD, USA; ^2^University of New Mexico Comprehensive Cancer Center, Beltsville, MD, USA; ^3^University of New Mexico Comprehensive Cancer Center, Albuquerque, NM, USA; ^4^University of New Mexico, Albuquerque, NM, USA

###### **Correspondence:** Sarah F Adams (sadams@salud.unm.edu)

**Background**

Our recent results demonstrate that the ovarian tumor environment is characterized by local T cell exhaustion and high levels of immunosuppressive cytokines, including interleukin (IL)-10 [1]. We hypothesized that IL-10 blockade would synergize with immune checkpoint antibodies to promote tumor clearance in ovarian cancer.

**Methods**

Dendritic cells (DC) in mice treated with 300ug of an IL-10 receptor antibody (IL-10Rab) were analyzed in two murine tumor models [2, 3]. In the implantable ID8ova model, mice were treated 7 and 14 days after tumor challenge; MISIIRTag mice were treated at 14 weeks of age. Immune checkpoint antibody treatment was evaluated in wildtype or IL10-knockout (IL10KO) mice treated with 500ug of anti-PD-1 antibody on days 17 and 21 after ID8ova tumor challenge (n = 5/group). Survival was measured from tumor challenge until mice reached 30 g due to ascites accumulation.

**Results**

In both models, IL-10Rab treatment increased stimulatory CD103+ DC (18 % to 30 % in ID8ova; 5 % to 45 % in MISIIRTag), and decreased suppressive Lair1+ DC in the peritoneal tumor environment and in primary ovarian tumors [1]. This was associated with an increase in CD8+ T cells and a decrease in regulatory FoxP3+ CD4+ T cells (45 % to 30 %). The proportion of CD4+ and CD8+ T cells producing interferon-gamma also increased (12 % to 28 %). Long-term survival was observed in 100 % of IL10KO mice treated with PD-1 antibody but treatment did not improve survival in wild-type controls.

**Conclusions**

These results demonstrate an enrichment of stimulatory CD103+ DC in the tumor microenvironment with IL-10R blockade, associated with evidence of increased T cell effector capacity and a reduction in suppressive Treg. This was associated with a significant survival benefit in IL10KO mice receiving anti-PD-1 antibody. These data support combining IL-10Rab with immune checkpoint antibodies for the treatment of ovarian cancer.

**References**

1. Flies DB, Higuchi T, Harris JC, Jha V, Gimotty PA, Adams SF: **Immune checkpoint blockade reveals the stimulatory capacity of tumor-associated CD103+ dendritic cells in late-stage ovarian cancer.** *Oncoimmunology* In press: http://www.tandfonline.com/doi/full/10.1080/2162402X.2016.1185583.

2. Roby KF, Taylor CC, Sweetwood JP, Cheng Y, Pace JL, Tawfik O, *et al.*: **Development of a syngeneic mouse model for events related to ovarian cancer.** *Carcinogenesis* 2000, **21**:585–591.

3. Connolly DC, Bao R, Nikitin AY, Stephens KC, Poole TW, Hua X, *et al.*: **Female mice chimeric for expression of the simian virus 40 TAg under control of the MISIIR promoter develop epithelial ovarian cancer.** *Cancer Res* 2003, **63**:1389–1397.

### P366 Axl tyrosine kinase is a key mediator of immunologic resistance after radiation therapy

#### Todd Aguilera^1^, Marjan Rafat^1^, Laura Castellini^1^, Hussein Shehade^1^, Mihalis Kariolis^1^, Dadi Jang^1^, Rie vonEbyen^1^, Edward Graves^1^, Lesley Ellies^2^, Erinn Rankin^1^, Albert Koong^1^, Amato Giaccia^1^

##### ^1^Stanford Department of Radiation Oncology, Stanford University School of Medicine, Stanford, CA, USA; ^2^Department of Pathology, University of California, San Diego, La Jolla, CA, USA

###### **Correspondence:** Todd Aguilera (toddagu1@stanford.edu)

**Background**

Hypofractionated high dose ionizing radiation (RT) can enhance antitumor immune responses in many cancers. In some cases the combination of RT and checkpoint immunotherapy suppress adaptive resistance leading to a greater immunologic effect. However, many tumors are unresponsive to the combination making it important to understand factors that render tumors immunologically inactive. We previously described immunologically responsive (Py117) and unresponsive (Py8119) syngeneic tumors from the PyMT mammary carcinoma model and used these tumors to determine new targets to reverse T cell exclusion.

**Methods**

A reverse phase protein array was used to study differences between the Py117 and Py8119 tumors. The CRISPR/Cas9 technology was used to knockout Axl, a TAM family tyrosine kinase. We confirmed signal abrogation with the loss of Axl through western blot, measured the intrinsic radiosensitivity by clonogenic survival, determined cellular proliferation, evaluated growth in 3D tissue culture, implanted tumors to determine radiosensitivity in mice, and evaluated the response in immunodeficient mice. Given the presence of an immunologic phenotype we measured the impact of Axl on antigen presentation and cytokine production. Lastly, we defined the antitumor immune response by dissociating tumors then immunophenotyping infiltrates, evaluating T cell function, and tumor cell responses. Lastly, we combined radiation, PD-1, and CTLA-4 therapy to demonstrate that loss of Axl sensitizes tumors to immunotherapy.

**Results**

Through a RPPA, we identified differences in Axl expression a protein associated with the epithelial to mesenchymal transition (EMT). Then we knocked out Axl, which revealed no changes in proliferation or intrinsic radiosenstivity but altered the EMT phenotype, resulted in greater tumor latency and enhanced radiosensitivity after 20 Gy in mice. Key features of the Axl knockouts were enhanced MHCI expression and decreased myeloid promoting cytokines and chemokines. The radiation response was decreased in mice carrying Axl knockout tumors suggesting the importance of the immune response. Profiling the tumor microenvironment revealed greater immune infiltrates in Axl knockout tumors and greater CD8+ T cells at baseline. Ten days after radiation there was increased CD8 and CD4+ T cells and decreased macrophages. T cells remained functional but adaptive immune resistance was supported by increased PD-L1 and FoxP3^+^ T regs in Axl deficient tumors. We hypothesized and confirmed that a greater radiation response was obtained with PD-1 and CTLA-4 inhibition in Axl deficient tumors.

**Conclusions**

These data suggest that Axl may not only mediate invasion and metastasis but can influence immunosurveillance and response to therapy by suppressing an antitumor immune response.

### P367 Impact of immune selection pressure on epithelial cell signaling pathway activation in a syngeneic pancreatic cancer model

#### Reham Ajina^1^, Shangzi Wang^1^, Jill Smith^1^, Mariaelena Pierobon^1^, Sandra Jablonski^1^, Emanuel Petricoin III^2^, Louis M Weiner^1^

##### ^1^Georgetown Lombardi Comprehensive Cancer Center, Washington, DC, USA; ^2^George Mason University, Manassas, VA, USA

###### **Correspondence:** Reham Ajina (rsa56@georgetown.edu)

**Background**

Pancreatic ductal adenocarcinoma (PDAC) is the fourth leading cause of cancer death in the United States [1]. PDAC is characterized by oncogenic *KRAS* mutations and resistance to chemotherapy and immunotherapy [2]. Epidermal growth factor receptor (EGFR) is required for *KRAS*-induced pancreatic tumorigenesis [3]. Although EGFR network activation represents a possible therapy target in PDAC, the anti-EGFR small molecule erlotinib has minimal therapeutic activity [4]. Accumulating evidence suggests that the immune system plays an important but complex role in the development and progression of PDAC [2]. Accordingly, we explored the effect of immune selection pressure on EGFR and related signaling pathways using syngeneic Panc02 pancreatic cancer models.

**Methods**

1 X10^6^ Panc02 cells were injected subcutaneously in immunocompetent B6.CB17 (WT) and immunodeficient B6.CB17-Prkdc^scid^/SzJ (SCID) mice (16mice/group). One cm^3^ tumors were harvested and processed for reverse phase protein array (RPPA) of 125 proteins (18 total proteins, 107 phosphorylated species) to evaluate protein signaling networks. Due to tumor invasiveness it was not possible to perform laser capture microdissection on the specimens. Statistical analysis included Wilcoxon test, Student’s t-test and principal component analysis (PCA) to identify significant hits. Pathways Studio was then used to identify selectively activated signaling pathways.

**Results**

As expected, Panc02 tumors grow more slowly in immunocompetent as opposed to syngeneic immunodeficient mice. Interestingly, PCA of the RPPA data demonstrated a significant difference in cellular protein activity between Panc02 tumors engrafted in the two groups of mice. 32.8 % (41/125) of proteins tested by RPPA were statistically significantly activated in immunocompetent mice as opposed to immunodeficient mice. Pathway analysis of these activated hits revealed selective activation of EGFR, ERK/MAPK, JAK/STAT, AMPK and TGFβ/Smad signaling pathways in immunocompetent mice.

**Conclusions**

Immune selection pressure in syngeneic Panc02 pancreatic cancer models selectively activates multiple, related signaling pathways. These observations lay important groundwork for understanding and therapeutically exploiting the interplay of host immunity and tumor cell signaling.

**References**

1. Siegel RL, Miller KD, Jemal A: **Cancer statistics, 2016.** *CA Cancer J Clin* 2016, **66(1)**:7–30.

2. Brunet LR, Hagemann T, Andrew G, Mudan S, Marabelle A: **Have lessons from past failures brought us closer to the success of immunotherapy in metastatic pancreatic cancer?** *Oncoimmunology* 2016, **5(4):**e1112942.

3. Ardito CM, Grüner BM, Takeuchi KK, Lubeseder-Martellato C, Teichmann N, Mazur PK, *et al.*: **EGF receptor is required for KRAS-induced pancreatic tumorigenesis.** *Cancer Cell* 2012, **22(3)**:304–317.

4. Kelley RK, Ko AH: **Erlotinib in the treatment of advanced pancreatic cancer.** *Biologics* 2008, **2(1)**:83–95.

### P368 Immune cell spatial analysis on FoxP3 and CD8 positive IHC stained T cells within the tumor microenvironment

#### Lorcan Sherry, John Waller, Mark Anderson, Alison Bigley

##### OracleBio, Newhouse, Scotland, UK

###### **Correspondence:** Lorcan Sherry (lorcan.sherry@oraclebio.com)

**Background**

The presence of T cells in the tumor microenvironment and their potential impact on prognosis has been investigated over many years. One implication has been that the presence of CD8 (cytotoxic T cell marker), as well as a high CD8/FoxP3 ratio, indicates a positive effect on patient survival. The forkhead box p3 (FoxP3) regulatory T cell (Tregs) marker has been utilized to investigate how Tregs function in suppressing immune response, in particular their influence on other T cells [1]. Therefore, understanding suppressive mechanisms and interactions between T cell subsets, by exploring spatial interactions, will inevitably provide evidence in support of the development of drugs for effective control of immune responses via Tregs. Using recent developments in histology image analysis techniques, we aimed to quantify CD8 and FoxP3 immune cell relationships in terms of cell infiltrations and cell-cell proximities within the tumor tissue microenvironment.

**Methods**

Tumor tissue was immunohistochemically (IHC) dual labelled for FoxP3 (brown nuclear chromogen) and CD8 (red membrane chromogen). Image analysis was performed within manually annotated regions of interest (ROI) using Indica Labs Halo™ software. Cellular analysis settings and thresholds were established to identify and count FoxP3, CD8, and dual labelled cells. 50 μm margin bands were generated around interface ROI into the active stroma region and out to tumor regions. Application of spatial analysis was performed using cytonuclear analyzed object data outputs to quantify infiltration analysis (number of cells per 50 μm margin band around defined interfaces) and proximity analysis (measure distances between defined cell populations within <50 μm range, using 5 μm bins).

**Results**

Tumor/stroma interface quantification indicated higher FoxP3 to CD8 ratios 100 μm inside the tumor boundary when compared to adjacent active stromal regions, 100 μm outside the tumor. This difference in cell number was also reflected in cell proximity values with shorter FoxP3 to CD8 cell distances in the stroma compared to tumor.

**Conclusions**

These example data highlight the benefits of utilizing tissue-based whole slide image analysis to characterize therapeutic activity using spatial correlations within the tumor microenvironment, which provides distinct advantages over flow cytometry-based approaches where critical information on spatial cellular context is lost.

**References**

1. Sakaguchi S, Wing K, Onishi Y, Prieto-Martin P and Yamaguchi: **Regulatory T cells: how do they suppress immune responses?** *Int Immunol* 2009, **21(10)**:1105–1111.

### P369 A CD122-biased agonist increases CD8 + T Cells and natural killer cells in the tumor microenvironment; making cold tumors hot with NKTR-214

#### Chantale Bernatchez^1^, Cara Haymaker^1^, Nizar M Tannir^1^, Harriet Kluger^2^, Michael Tetzlaff^1^, Natalie Jackson^1^, Ivan Gergel^3^, Mary Tagliaferri^3^, Jonathan Zalevsky^3^, Ute Hoch^3^, Patrick Hwu^1^, Mario Snzol^2^, Michael Hurwitz^2^, Adi Diab^1^

##### ^1^University of Texas MD Anderson Cancer Center, Houston, TX, USA; ^2^Yale Medical Oncology, New Haven, CT, USA; ^3^Nektar Therapeutics, San Francisco, CA, USA

###### **Correspondence:** Ute Hoch (saung@nektar.com)

**Background**

Abundance and functional quality of tumor-infiltrating lymphocytes are positively linked with tumor response and improved survival with checkpoint inhibitors. NKTR-214 provides sustained activation of the IL-2 pathway through controlled release of active CD122-biased (IL-2Rβɣ) cytokines. Preclinical models demonstrated NKTR-214 preferentially expands effector CD8 + T cells and NK cells within the tumor resulting in marked tumor growth suppression as single-agent and in combination with checkpoint inhibitors. A phase I/II trial was initiated to evaluate NKTR-214 safety and efficacy and to assess immune changes in the tumor microenvironment.

**Methods**

This is an ongoing dose-escalation and dose-expansion study of NKTR-214 in patients with locally advanced or metastatic solid tumors with a standard 3 + 3 design. Extensive blood and tumor tissue samples have been collected to measure immune activation using immunophenotyping including flow cytometry, immunohistochemistry (IHC), T cell clonality and gene expression analyses. Safety, pharmacokinetics and preliminary anti-tumor activity of NKTR-214 are being evaluated. NKTR-214 is administered IV q2-q3 weeks in an outpatient setting with initial dosing at 0.003 mg/kg.

**Results**

As of August 2, 2016, 18 patients who received prior standard of care were enrolled (RCC [n = 12], MEL [n = 4], CRC [n = 1]; UBC [n = 1]). 7/12 (58 %) patients had stable disease at initial 6 or 8-week scan with 9 patients still on treatment. Outpatient treatment with NKTR-214 was well tolerated and the MTD has not been reached. One patient experienced DLTs (Grade 3 syncope and hypotension) at 0.012 mg/kg. No immune-related AEs or capillary leak syndrome were observed at any dose. No drug-related AEs led to study discontinuation. 9/18 patients had serial tumor biopsies (pre and post-dose) evaluable. In 6/9 patients, flow cytometry enumeration and/or IHC revealed an up to 10-fold increase from baseline in CD8 + T cells and NK cells in the tumor microenvironment, with minimal changes to Tregs. Most of the infiltrating CD8 + T cells were newly proliferative (Ki67+) and cell-surface PD-1 expression was increased up to 2-fold. Analyses of blood samples showed concordant increases in Ki67+ immune cells, PD-1+ CD8 + T cells, and NK cells 8 days after a single dose of NKTR-214. The study continues to enroll patients.

**Conclusions**

NKTR-214 results in substantial increases in both CD8 + T cells and NK cells in the tumor microenvironment with a favorable outpatient safety profile. These data support continued evaluation of NKTR-214 and the potential advantages of combining NKTR-214 with a variety of immunotherapeutic agents including checkpoint inhibitors. Updated data will be presented.

**Trial Registration**

ClinicalTrials.gov identifier NCT02869295.

### P370 NF-kB p50 promotes the suppressive M2 phenotype of tumor-associated macrophages in a mouse model of glioma

#### Theresa Barberi, Allison Martin, Rahul Suresh, David Barakat, Sarah Harris-Bookman, Charles Drake, Alan Friedman

##### Johns Hopkins University, Baltimore, MD, USA

###### **Correspondence:** Theresa Barberi (tbarber6@jhmi.edu)

**Background**

Glioblastoma multiforme (GBM) brain tumors are nearly uniformly fatal. The GBM microenvironment includes abundant tumor-associated macrophages (TAMs) that predominantly assume a pro-tumor “M2” phenotype rather than a pro-inflammatory “M1” phenotype. The inhibitory p50 subunit of the NF-kB transcription factor exhibits markedly increased nuclear expression in TAMs and M2-polarized macrophages, and p50 knockdown/deletion suppresses expression of M2-associated factors [1, 2]. We hypothesize that absence of p50 will convert TAMs to an M1 phenotype that will reduce glioma growth and prolong survival.

**Methods**

GL261-Luc cells were intracranially implanted into mice. Tumor growth was monitored by IVIS imaging. Brains were removed 13–16 days after implantation for flow cytometry (FC) or RT-qPCR analysis. Depleting antibodies were administered by i.p. injection; clodronate by tail vein injection. Naïve T cells were enriched from spleens, skewed *in vitro* with cytokines and blocking antibodies, expanded in IL-2, then stimulated with PMA/ionomycin. Cells were assessed for Th or Tc1 skewing by FC or ELISA.

**Results**

p50(−/−) mice exhibit significantly slower GL261-Luc tumor growth and prolonged survival. p50(−/−) tumor CD11b + myeloid cells express increased M1-associated and decreased M2-associated mRNAs relative to WT mice. FC indicates glioma-bearing p50(−/−) brains contain fewer TAMs expressing the M2 marker CD206/MRC1, as well as fewer Tregs, increased IFNg-producing CD4+ T cells, and increased granzyme B+ CD8+ T cells. Transplant of p50(−/−) bone marrow into lethally-irradiated WT recipients confers a significant survival advantage upon tumor inoculation. Clodronate-mediated macrophage depletion decreases survival of tumor-bearing p50(−/−) mice but has no effect on WT mice. Depletion of CD4+ T cells markedly reduces survival in tumor-bearing p50(−/−) mice whereas depletion of CD8+ T cells has no effect. We observe no intrinsic defect in the ability of p50(−/−) naïve splenic CD4+ T cells to differentiate into Tregs, Th1, Th17, or Th2 subsets; however, p50(−/−) naïve splenic CD8+ T cells exhibit enhanced ability to produce IFNg, TNFa, and granzyme B.

**Conclusions**

NF-kB p50 is an important modulator of the suppressive immune phenotype in GBM. Both TAM and T cells are more activated and less tumor-permissive when p50 is absent. Targeted deletion of p50 from TAMs and/or T cells may serve as a viable therapeutic for patients with GBM.

**References**

1. Saccani A, *et al.*: **p50 nuclear factor-κB overexpression in tumor-associated macrophages inhibits M1 inflammatory responses and antitumor resistance**. *Cancer Res* 2006, **66(23)**:11432–11440.

2. Porta C, *et al.*: **Tolerance and M2 (alternative) macrophage polarization are related processes orchestrated by p50 nuclear factor κB.** *Proc Natl Acad Sci* 2009, **106(35)**:14978–14983.

### P371 The differentiation of Th17-into-Treg cells in the tumor microenvironment reveals novel targets in cancer immunotherapy

#### Sara Berkey^1^, Stephanie Downs-Canner^1^, Greg M Delgoffe^2^, Robert P Edwards^2^, Tyler Curiel^3^, Kunle Odunsi^4^, David Bartlett^1^, Nataša Obermajer^1^

##### ^1^Department of Surgery, University of Pittsburgh, Pittsburgh, PA, USA; ^2^University of Pittsburgh, Pittsburgh, PA, USA; ^3^University of Texas Health Science Center at San Antonio, San Antonio, TX, USA; ^4^Roswell Park Cancer Institute, Buffalo, NY, USA

###### **Correspondence:** Sara Berkey (berkeyse@upmc.edu)

**Background**

Th17 and regulatory T (T_reg_) cells are integral in maintaining immune homeostasis and the Th17-T_reg_ balance is associated with the inflammatory immunosuppression observed in cancer. Expansion of T_reg_ cells within the tumor is a known barrier to successful cancer immunotherapy. Because of this, T_reg_ cell regulation has become a popular interest for new therapeutic modalities. However, current approaches designed to deplete T_reg_ cells are only variably effective and urge for novel strategies to specifically target the suppressive function of intratumoral T_reg_ cells.

**Methods**

We have used IL17 (*Il17a* ^Cre^ *Rosa26* ^eYFP^) and T_reg_ (B6.129(Cg)-*Foxp3* ^*tm3(DTR/GFP)Ayr*^) reporter mice to study Th17 cell plasticity and Th17-into-T_reg_ cell transdifferentiation as a novel tumor-associated phenomenon supporting immunosuppressive microenvironment in tumor-bearing mice.

**Results**

We demonstrate that in addition to natural (n)T_reg_ and induced (i)T_reg_ cells developed from naïve precursors, Th17 cells are a novel source of tumor-induced forkhead box P3 (Foxp3^+^) cells by progressive direct conversion into immunosuppressive IL17A^+^Foxp3^+^ and ex-Th17 Foxp3^+^ cells. Transcriptome analysis of the Th17-T_reg_ plastic subsets reveals upregulation of 119 genes in the IL17A^+^Foxp3^+^ cells compared to IL17A^+^ Foxp3^neg^ Th17 cells (Th17-T_reg_ plasticity markers). Seven of these plasticity markers identified by transcriptome analysis were confirmed by flow cytometry of plastic IL17^+^Foxp3^+^ cells. The immunometabolism of the plastic IL17A^+^Foxp3^+^ subset revealed an additional level of complexity in controlling the immune function of CD4^+^ T cells and to modulate (trans) differentiation of Th cells, which thereby control their ultimate function and role in diverse environments.

**Conclusions**

Tumor-associated Th17-into-Foxp3^+^ T cell transdifferentiation helps to reconcile the contradictory observations about the role of Th17 cells in tumor immune surveillance, and demonstrates an alternative source for IL17^+^Foxp3^+^ and IL17^neg^Foxp3^+^ T cells in tumors. Further, this newly identified tumor-associated phenomenon warrants strategies to manipulate the Th17-T_reg_ cell plasticity in cancer and identifies novel IL17/T_reg_- associated targets that might be amenable for therapeutic interventions to enhance antitumor immunity.

### P372 Antigen-presenting tumor B cells associate with the phenotype of CD4+ tumor infiltrating T cells in non-small cell lung cancer patients

#### Tullia C Bruno^1^, Brandon Moore^2^, Olivia Squalls^2^, Peggy Ebner^2^, Katherine Waugh^2^, John Mitchell^3^, Wilbur Franklin^4^, Daniel Merrick^4^, Martin McCarter^5^, Brent Palmer^6^, Jeffrey Kern^7^, Dario Vignali^8^, Jill Slansky^2^

##### ^1^Department of Immunology, University of Pittsburgh, Pittsburgh, PA, USA; ^2^Department of Immunology and Microbiology, University of Colorado, Aurora, CO, USA; ^3^Department of Cardiothoracic Surgery, University of Colorado, Aurora, CO, USA; ^4^Department of Pathology, University of Colorado, Aurora, CO, USA; ^5^Department of Surgery, University of Colorado, Aurora, CO, USA; ^6^Department of Medicine, University of Colorado, Aurora, CO, USA; ^7^National Jewish Health, Denver, CO, USA; ^8^University of Pittsburgh, Pittsburgh, PA, USA

###### **Correspondence:** Tullia C Bruno (tbruno@pitt.edu0)

**Background**

Despite improvements in surgical techniques and combined chemo- and immunotherapies, the 5-year survival rate for all stages of non-small cell lung cancer (NSCLC) is only 18 %. The focus of immunotherapy has been on subsets of CD8+ and CD4+ tumor infiltrating lymphocytes (TILs). However, tumor infiltrating B cells (TIL-Bs) have been reported in tertiary lymphoid structures (TLS) with CD4+ TILs, both positively correlating with patient survival. TIL-B function in the tumor microenvironment (TME) has been understudied with no focus on their role as antigen presenting cells (APCs). We hypothesized that TIL-Bs help generate potent, long-term immune responses against cancer by presenting tumor antigens to CD4+ TILs.

**Methods**

All studies were completed on freshly collected, un-manipulated primary human B cells from tumor and tumor adjacent lung tissue. We analyzed the number and phenotype of TIL-Bs via flow cytometery and immunofluorescence. We generated a specific antigen presentation assay *in vitro* to assay APC function.

**Results**

We observed that the total number of B cells at the site of the tumor versus the tumor-adjacent tissue was increased compared to other immune subsets. Further, we found three types of CD4+ TIL responses when TIL-Bs presented autologous tumor antigens. There were activated responder CD4+ TILs that proliferated when combined with TIL-Bs alone, which indicates stimulation with endogenous tumor antigens. There were antigen-associated responders that required exogenous autologous tumor lysate to elicit a CD4+ TIL response, and there were patient CD4+ TILs that did not respond to antigen presentation by TIL-Bs. Within the activated and antigen-associated responders, the TIL-B phenotype associated with the CD4+ TIL phenotype; if the TIL-Bs were activated (CD69 + CD27 + CD21+), the CD4+ TILs were T helper (anti-tumor) CD4+ T cells and if the TIL-Bs were exhausted (CD69-CD27-CD21-), the CD4+ TILs were T regulatory cells (pro-tumor). These data suggest that TIL-Bs affect the phenotype and function of CD4+ TILs in NSCLC patient tumors.

**Conclusions**

In conclusion, TIL-Bs are increased in NSCLC primary tumors and they can present antigen to and associate with CD4+ TILs in the tumor microenvironment. Determining if TIL-Bs are activated or exhausted in NSCLC patients will determine the extent of their anti-tumor function. Ultimately, results from this study will help predict how to target TIL-B functions in future immunotherapies for NSCLC patients.

**Acknowledgements**

T32 grant AI007505, the American Cancer Society grant RSG LIB-114645, Cancer League of Colorado, The University of Colorado Lung Cancer SPORE Career Development Award 5P50CA058187-20, and CCSG CA 046934, and CCTSI UL1 TR 001082.

### P373 Imprime PGG, a novel pathogen associated molecular pattern (PAMP) treatment elicits M1-like transcriptional profile from bone marrow-derived macrophages and tumor associated macrophages (TAMs)

#### Anissa SH Chan, Xiaohong Qiu, Kathryn Fraser, Adria Jonas, Nadine Ottoson, Keith Gordon, Takashi O Kangas, Steven Leonardo, Kathleen Ertelt, Richard Walsh, Mark Uhlik, Jeremy Graff, Nandita Bose

##### Biothera Pharmaceuticals Inc., Eagan, MN, USA

###### **Correspondence:** Anissa SH Chan (achan@biothera.com)

**Background**

The success of cancer immunotherapy is often limited by multiple mechanisms of tumor-induced immunosuppression; M2-like TAMs being one of the critical mediators of immunosuppression. Imprime PGG (Imprime), an intravenously administered soluble yeast β-1,3/1,6 glucan is being clinically developed in combination with tumor-targeting antibodies, anti-angiogenics, and checkpoint inhibitors. Imprime has shown promising results in two randomized phase II studies in non-small cell lung cancer (NSCLC). Imprime acts mechanistically as a PAMP enlisting innate immune functions including cytotoxic effector mechanisms, reversal of immunosuppression and cross-talk with the adaptive immune system. With respect to immunosuppression, Imprime has been shown to repolarize M2 macrophages to an anti-tumor M1-like orientation in human *ex vivo* studies [1]. The objective of this study was to expand on this finding in an *in vivo* setting.

**Methods**

Imprime’s M1-polarization effect was evaluated in tumor-free mice, and xenograft and syngeneic tumor models. Bone marrow-derived macrophages (BMDM) prepared from Imprime- or vehicle-treated tumor-free mice were evaluated by qRT-PCR. Imprime was tested in combination with an anti-angiogenic agent, DC101 (α-VEGFR2 Mab) in H441 NSCLC xenograft model in athymic nude mice, and in combination with anti-TRP1 tumor-targeting antibody TA-99 in B16 experimental lung metastases model. Immunohistochemistry of FFPE tumor tissue or lung tissue were evaluated.

**Results**

qRT-PCR analyses of Imprime-treated BMDM from tumor-free mice revealed an increase in M1 markers (iNOS, PD-L1, IL-12b, TNF-a, CXCL9, CXCL10, and CXCL11) with a coincident decrease in M2 markers (CD206, YM-1, Fizz1, and CCL17). Imprime’s M1-polarization effect was also observed in H441 NSCLC tumor model where Imprime treatment alone upregulated M1-like genes in TAMs as well as significantly suppressed tumor growth when combined with DC101, an agent that also modulates tumor microenvironment. Imprime-mediated M1-polarization was also observed in the B16 experimental lung metastasis model where the combination of Imprime with TA-99 significantly reduced both the number and size of B16 lung metastases. Immunohistochemistry analysis showed an increase in the number of tumor-infiltrating CD11b + cells with an M1 phenotype evidenced by increase in iNOS expression.

**Conclusions**

Collectively, these data indicate that Imprime, by reorienting the M2 macrophages to an M1-like polarization state can remold the suppressive tumor microenvironment to be more sensitive to other immunotherapeutic modalities.

**References**

1. Chan A, Qui X, Jonas AB, Patchen ML, Bose N: **Imprime PGG, a yeast β-glucan immunomodulator, has the potential to repolarize human monocyte-derived M2 macrophages to M1 phenotype.** *JITC* 2014, **2(Suppl 3)**:191.

### P374 Integrated genomics approach of modeling tumors to assess their sensitivity to immune-mediated elimination

#### Ravi Gupta, Nitin Mandloi, Kiran Paul, Ashwini Patil, Rekha Sathian, Aparna Mohan, Malini Manoharan, Amitabha Chaudhuri

##### MedGenome Inc., Foster City, CA, USA

###### **Correspondence:** Amitabha Chaudhuri (amitc@medgenome.com)

**Background**

The somatic mutation burden, together with the immunoregulatory processes active in the tumor microenvironment, can provide powerful predictive biomarkers to facilitate the clinical translation of checkpoint control modulators and therapeutic vaccines. The molecular determinants of a productive anti-tumor immune response is multifactorial, shows significant intersubject variability and is influenced by host genetic and environmental factors. To investigate this complex interaction between the epithelial, stromal and the immune compartments, an unbiased NGS approach is a powerful method that can complement other conventional techniques, such as immunohistochemistry and cell sorting.

**Methods**

In this study, we have used our proprietary integrated NGS-based immuno-genomics platform OncoPept™, to analyze the TCGA somatic mutation and gene expression data and produced novel biological insights of therapeutic relevance. The combined expression of genes present in a signature was used to calculate an expression score that captured the relative abundance of specific cell types within the tumor. We also analyzed 9345 tumors from 33 cancers for T cell neo-epitope burden using our proprietary neo-epitope prioritization pipeline and combined the abundance of T cell neo-epitopes with the infiltration of specific immune cell types present in the tumor microenvironment to examine potential relationship between these two separate biological events.

**Results**

Our signature-based analysis of immune cell abundance identified core signaling pathways associated with high CD8+ T cell infiltration. These core pathways are enriched across many different cancers. Components from these core pathways can be combined with other immune cell markers to create a comprehensive response signature to select patients for treatment with checkpoint control modulators. Several recent studies have highlighted the significance of T cell neo-epitopes in enhancing the therapeutic benefit of cancer immunotherapy drugs, specifically in tumors with low mutation burden. We assessed the T cell neo-epitope burden using our neo-epitope prioritization pipeline and observed that in some cancers the neo-epitope load is associated with higher inflammation, higher CD8+ T cell infiltration, IFN-g signaling and high PD-1 and PD-L1 level providing a basis for superior clinical response.

**Conclusions**

In conclusion, our analysis supports the idea that application of NGS in a clinical setting has the potential to generate deep biological insights of the tumor and its microenvironment to enable cancer immunotherapy treatment effective and personalized.

### P375 Classification of gastric cancer based on tumor microenvironment expression of PD-L1 and CD8+ T cell infiltration

#### Yu Chen, Jing Lin, Yun-bin Ye, Chun-wei Xu, Gang Chen, Zeng-qing Guo

##### Fujian Provincial Cancer Hospital, Fuzhou, Fujian, People’s Republic of China

###### **Correspondence:** Yu Chen (13859089836@139.com)

**Background**

Previous data has shown that a positive response to immunotherapy usually relies on active interactions between tumor cells and immunomodulators inside the tumor microenvironment (TME). The aim of this study was to classify gastric cancer subsets based on the TME immune status according to the expression of PD-L1 and infiltration of CD8+ T cells.

**Methods**

186 gastric cancer patients with a curative D2 gastrectomy were enrolled (Table 6). PD-L1 and CD8+ T cell status were evaluated with immunohistochemistry using specific antibodies (SP142, SP16). The samples were classified into four TME immune types and associated with different clinicopathological features and outcomes.

**Results**

**Fig. 65 (abstract P375).**
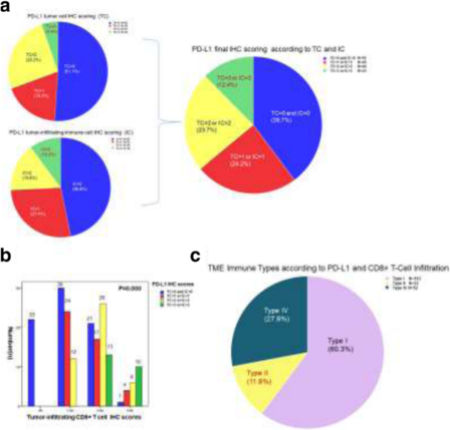
**a** Distribution (%) of 186 patients with gastric cancer according to the expression of PD-L1 on tumor cells (TC) and tumor-infiltrating immune cells (IC). **b** PD-L1 expression by tumor cells (TC) and tumor-infiltrating immune cells (IC) is reduced with fewer infiltrating CD8+ T cells. **c** Distribution (%) of tumor microenvironment immune type based on the presence of tumor-infiltrating CD8+ T cells and PD-L1 status. Tumor-infiltrating CD8+ T cell IHC score=1-3 were considered positive. PD-L1 in tumor samples by TC1/2/3 or IC1/2/3 was considered positive

**Fig. 66 (abstract P375).**
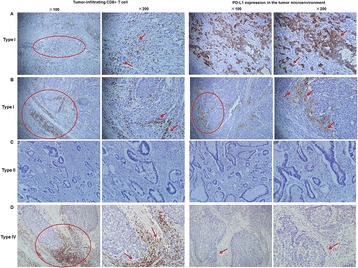
Representative images of immunohistochemistry (IHC) staining for tumor-infiltrating CD8+ T cells and PD-L1 status from the 186 patients with gastric cancer. **a** Type I, adaptive immune resistance. More than 50% of the tumor cells (TC=3) demonstrated cell membrane PD-L1 expression with a “severe” grade of CD8+ T cell infiltration. **b** Type I, adaptive immune resistance. About 1-3% of the tumor cells (TC=1) and 3-5% tumor-infiltrating immune cells (IC=1) in the invasive tumor margin demonstrated cell membrane PD-L1 expression with a “moderate” grade of CD8+ T cell infiltration. **c** Type II, immune ignorance. PD-L1 negative (TC=0 and IC=0) with no CD8+ T cell infiltration. **d** Type IV, other suppressor. PD-L1 negative (TC=0 and IC=0) with a “severe” grade of CD8+ T cell infiltration

**Fig. 67 (abstract P375).**
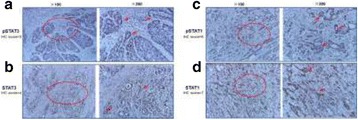
Representative images of IHC staining for STAT3, pSTAT3, STAT1, and pSTAT1 from the 186 patients with gastric cancer. **a** pSTAT3 positive cells with nuclear staining in the invasive tumor margin, IHC score=3. **b** STAT3 positive cells with cytoplasmic staining in tumor foci and in the invasive tumor margin, IHC score=4. **c** pSTAT1 positive nuclear staining in tumor cells and partly in stroma lymphocyte, IHC score=6. **d** STAT1 positive cytoplasmic staining in tumor cells and partly in stroma lymphocyte, IHC score=7

**Fig. 68 (abstract P375).**
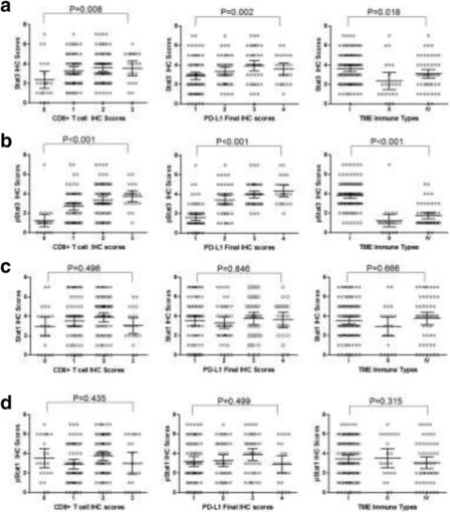
Relation between immunohistochemical staining for STAT3 (**a**), pSTAT3 (**b**), STAT1 (**c**), and pSTAT1 (**d**) in 186 tumor specimens and tumor-infiltrating CD8+ T cell, PD-L1 status and TME immune type

**Fig. 69 (abstract P375).**
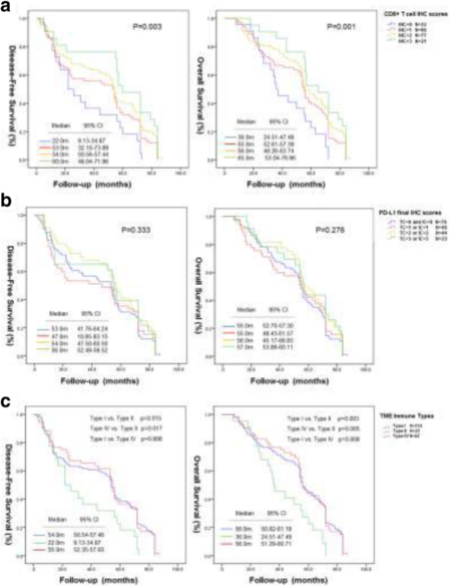
Kaplan-Meier survival curves for Disease-Free-Survival (DFS) and Overall Survival (OS) in 186 patients according to tumor-infiltrating CD8+ T cells, PD-L1 status and TME immune type. **a** DFS and OS associations with four grades of tumor-infiltrating CD8+ T cells. **b** DFS and OS associations with PD-L1 expression by the TC and IC. **c** DFS and OS associations with TME immune type

**Fig. 70 (abstract P375).**
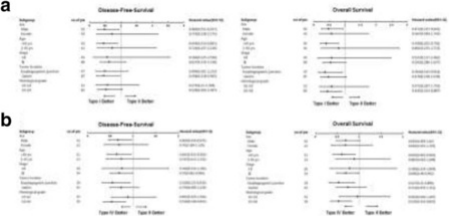
Prespecified subgroup analysis of Disease-Free-Survival and Overall Survival, according to the TME immune type

Among 186 samples, there was positive PD-L1 expression (TC1/2/3 or IC1/2/3) in 60.3 % (112/186) of patients (Fig. 65a). A significant correlation between the PD-L1 expression and the intensity of CD8+ T cell infiltration (p = 0.000, Fig. 65b) was found. According to the immune-related classification, the TME was divided into both PD-L1+ and CD8+ T cell positive (type I), both PD-L1 and CD8+ T cell negative (type II), PD-L1 positive but CD8+ T cell negative (type III), and PD-L1 negative but CD8+ T cell positive (type IV). Types I, II, III, IV were 60.3 %, 11.8 %, 0 %, and 27.9 %, respectively (Fig. 65c, Fig. 66, Table 7). The expression of STAT3, and pSTAT3, rather than STAT1 and pSTAT1, was significantly correlated with the CD8+ T cell infiltration, and PD-L1 status (Fig. 67, Fig. 68). CD8+ T cell infiltration was significantly associated with disease-free survival (DFS) and overall survival (OS) (p = 0.003 and p = 0.001, Table 8, Table 9, Fig. 69a). The DFS and OS of patients with immune type II tumors was significantly worse compared to immune types I and IV; there was no significant difference in DFS and OS between type I and IV (type I vs. type II, p = 0.015, p = 0.003; type IV vs. type II, p = 0.017, p = 0.005; type I vs. type IV, p = 0.806, p = 0.808; Fig. 69c). The hazard ratios of DFS and OS numerically favored type I and type IV compared with type II across most subgroups (Fig. 70).

**Conclusions**

This is the first reported stratification of TME based on PD-L1 expression and CD8+ T cell infiltration in gastric cancer. The PD-L1 expression was significantly correlated with the CD8+ T cell infiltration. Immune type III was absent, and type II patients have a worst prognosis compared with type I and IV patients. Our results may be useful for the development of clinical treatments for the blockade of immune checkpoints.

**Table 6 (abstract P375).** Baseline characteristics (N=186)

Characteristics		Total (n=186)
Age	Mean-59.5 (27–79)	
	<65	127 (68.3%)
	≥65	59 (31.7%)
Sex	Male	128 (68.8%)
	Female	58 (31.2%)
Histological grade	G1-G2	78 (41.9%)
	G3-G4	108 (58.1%)
Stage	I	18 (9.7%)
	II	43 (23.1%)
	III	125 (67.2%)
Tumor location	Esophagogastric junction	70 (37.6%)
	Gastric	116 (62.4%)

**Table 7 (abstract P375).** Correlation of Tumor-infiltrating CD8+ T cells, PD-L1 status, and TME Immune Types with Clinicopathologic Features in 186 patients

Characteristics		Tumor-infiltrating CD8+ T cell					PD-L1 status			TME Immune Types			
		IHC=0 (n)	IHC=1 (n)	IHC=2 (n)	IHC=3 (n(	P-value	TC=0 and IC=0 (n)	TC=1/2/3 or IC-1/2/3 (n)	P-value	Type I	Type II	Type IV	P-value
Sex	Male	16	45	54	13	0.874	52	76	0.728	76	16	36	0.901
	Female	6	21	23	8		22	36		36	6	16	
Age	<65	17	42	55	13	0.527	51	76	0.879	76	17	34	0.597
	≥65	5	24	22	8		23	36		36	5	18	
Histological grade	G1-G2	9	35	29	5	0.080	35	43	0.228	43	9	26	0.372
	G3-G4	13	31	48	16		39	69		69	13	26	
Stage	I	2	6	6	4	0.637	6	12	0.395	12	2	4	0.671
	II	3	15	21	4		14	29		29	3	11	
	III	17	45	50	13		54	71		71	17	37	
Tumor location	EGJ	6	29	29	6	0.414	29	41	0.722	41	6	23	0.364
	Gastric	16	37	48	15		45	71		71	16	29

**Table 8 (abstract P375).** Univariate analysis of clinicopathologic prognostic factors for survival in 186 patients

Variable		n	m DFS	p-value	m OS	p-value
Sex	Male	127	53.0	0.335	54	0.225
	Female	59	57.0		63.0	
Age	<65	128	55.0	0.196	56.0	0.259
	≥65	58	43.0		54.0	
Histological guide	G1-G2	78	55.0	0.375	57.0	0.452
	G3-G4	108	53.0		55.0	
Stage	I	18	72.0	0.024	72.0	0.040
	II	43	55.0		64.0	
	III	125	34.0		54.0	
Tumor location	EGJ	70	43.0	0.123	54.0	0.136
	Gastric	116	56.0		57.0	
CD8+ T-cells	IHC=0	22	22.0	0.003	36.0	0.001
	IHC=1	66	53.0		55.0	
	IHC=2	77	54.0		56.0	
	IHC=3	21	60.0		65.0	
PD-P1 status	TC=0 and IC=0	74	53.0	0.333	55.0	0.276
	TC=1 or IC=1	45	47.0		55.0	
	TC=2 or IC=2	44	54.0		56.0	
	TC=3 or IC=3	23	56.0		57.0

**Table 9 (abstract P375).** Multivariate analysis of prognostic factors for survival in 186 patients

Variable		DFS			OS	
	P value	Hazard ratio	95% CI	P value	Hazard ratio	95% CI
CD8+ T-cells	0.006	0.779	0.652-0.930	0.013	0.747	0.593-0.941
Stage	0.033	1.289	1.020-1.628	0.042	1.277	1.009-1.616
TME Immune Types	0.095	0.853	0.707-1.028	0.108	0.859	0.714-1.034
PD-L1	0.420	0.896	0.686-1.170	0.452	0.903	0.692-1.178

### P376 Molecular profiling of tumor microenvironment for biomarker discovery

#### Andrey Komarov, Alex Chenchik, Michael Makhanov, Costa Frangou

##### Cellecta, Inc., Mountain View, CA, USA

###### **Correspondence:** Andrey Komarov (akomarov@cellecta.com)

**Background**

Human carcinomas consist of a complex mixture of neoplastic epithelial cells, endothelial cells, fibroblasts, myofibroblasts, and immune cells, which collectively form the tumor stroma. The presence of stromal cell types within carcinomas is a powerful component of the biology of a tumor through its ability to have profound influences on cancer progression, metastasis, and drug resistance. Correspondingly, the clinical importance of tumor immune infiltrates is now recognized as an emerging area of triple-negative breast cancer (TNBC) research, where increased immune infiltrate predicts both responses to chemotherapy and improved survival. Quantitative molecular profiles of tumor-associated normal cells may provide valuable insights into tumor biology and facilitate the discovery of new biomarkers and therapeutic targets.

**Methods**

To this end, we have developed GeneNet™, a genome-wide targeted RNA expression assay that profiles ~19,000 genes using multiplex RT-PCR followed by NGS. The GeneNet 19 K assay provides comprehensive cellular composition analysis of tumor stroma and allows human hematopoietic cell phenotypes, including naïve and memory B cells, seven T cell types, dendritic cells, plasma cells, natural killer (NK) cells and myeloid subsets to be distinguished. The developed targeted RNA expression platform profiles in 10^5-fold dynamic range the complete set of genes involved in checkpoint blockade and immunotherapy biomarkers, immune cell activation, and canonical immune pathway genes for both innate adaptive and humoral immune responses.

**Results**

Proof-of-principle studies demonstrate the assay's unparalleled specificity and sensitivity resulting in unique detection of low abundance mRNA transcripts as well as an improved cost-effectiveness for high-throughput clinical applications.

**Conclusions**

In this study, we present a molecular assay that unambiguously characterizes the cellular composition of the tumor microenvironment and determines the activation status of infiltrating immune cells in primary tumor tissues from TNBC patients.

### P377 A fully-automated staining assay for probing the tumor microenvironment using fluorescent multiplex immunohistochemistry

#### Yi Zheng, Carla Coltharp, Darryn Unfricht, Ryan Dilworth, Leticia Fridman, Linying Liu, Milind Rajopadhye, Peter Miller

##### PerkinElmer, Hopkinton, MA, USA

###### **Correspondence:** Yi Zheng (yi.zheng@perkinelmer.com)

**Background**

In recent years, cancer immunotherapy research has increasingly leveraged knowledge about the tumor microenvironment for the development of novel therapeutics and targets. Advancing our current understanding of the tumor microenvironment will involve continued characterization of the interactions that occur among immune cells and cancer cells that reside within the tumor and its periphery. Fluorescent multiplex immunohistochemistry (mIHC) assays are uniquely suited to characterizing and quantifying these complex interactions *in situ*. In response, we commercialized manual Opal™ mIHC and Opal™ cancer immunology staining kits that are optimized for multispectral imaging. Here we describe a robust, fully-automated 7-color mIHC procedure that streamlines Opal™ staining. We coupled this automated staining procedure with a multispectral imaging system for simultaneous detection of up to 6 tissue biomarkers plus nuclear counterstain, providing the ability to visualize interactions between specific immune and cancer cells within the context of the tumor microenvironment.

**Methods**

Formalin-fixed paraffin-embedded samples of primary tumors were immunostained using Opal™ reagent kits on a fully automated Leica BOND RX™ stainer, imaged with a Vectra 3.0® automated imaging system, and analyzed with inForm® software. Equivalent assays using manual staining protocols were also performed for comparison.

**Results**

We developed a fully-automated staining protocol and assessed its robustness with respect to fluorescence signal degradation, impact on antigen epitope availability, influence on tissue morphology, and potential cross-labeling due to incomplete inactivation between staining steps. We observed minimal degradation of fluorescence signal, epitope availability, or tissue morphology throughout the automated staining process. We achieved comparable signal contrast and cross-talk levels to those obtained with manual protocols while substantially reducing hands-on time.

**Conclusions**

The fully-automated staining assay that we developed for Opal™ 7-color mIHC is a robust method to increase the throughput of multiplex tissue staining.

### P378 EGFR/JAK2 inhibition downregulates immunosuppressive cytokine secretion in head and neck cancer

#### Fernando Concha-Benavente, Julie Bauman, Sumita Trivedi, Raghvendra Srivastava, James Ohr, Dwight Heron, Uma Duvvuri, Seungwon Kim, William Gooding, Robert L Ferris

##### University of Pittsburgh, Pittsburgh, PA, USA

###### **Correspondence:** Fernando Concha-Benavente (conchabenaventef@upmc.edu)

**Background**

The EGFR, which is overexpressed in more than 90 % of head and neck cancers (HNC), activates the Janus kinase 2 (JAK2)/signal transducer and activator of transcription 3 (STAT3) pathway leading to carcinogenesis. STAT3 plays a major role in promoting tumor immune escape, including production of immunosuppressive cytokines such as vascular endothelial growth factor (VEGF), IL-6, IL-10 and TGFβ, which in turn activate STAT3 in tumor-associated suppressive immune cells, providing a feed forward mechanism to ensure a STAT3-dominated microenvironment. Cetuximab, a specific EGFR blocking mAb, downregulates EGFR-mediated STAT3 activation; however, other STAT3 activating pathways exist. The IL-6 receptor (IL-6R) constitutes a major EGFR-independent STAT3 activating pathway in HNC. Given that JAK2 is common signaling molecule to both EGFR and IL-6R pathways we hypothesized that combined EGFR and JAK2 inhibition may downregulate STAT3-dependent production of immunosuppressive cytokines.

**Methods**

mRNA expression for the cytokines analyzed in this study was accessed and downloaded from the cancer genome atlas (TCGA) repository. Protein concentration of cytokines in plasma from head and neck cancer patients enrolled in cetuximab single agent on a neoadjuvant trial (UPCI #08-013, NCT #01218048) were determined by ELISA. Data analysis was done using Graphpad v6.0.

**Results**

Herein we show that tumors of HNC patients annotated in The Cancer Genome Atlas (TCGA) express higher immunosuppressive cytokines including TGFβ, IL-10, VEGFA and IDO than control tissues and have lower expression of inflammatory cytokines such as IL-12A and IL-17A, confirming the view of a dominant immunosuppressive tumor microenvironment that prevents proper immune effector cell activation. In addition, EGFR and JAK2 inhibition downregulate STAT3 activation and secretion of these immunosuppressive STAT3-dependent cytokines *in vitro*, providing evidence that supports targeting the EGFR/JAK2/STAT3 mediated suppressive tumor microenvironment.

**Conclusions**

Importantly, our findings are clinically relevant since HNC patients that were treated with cetuximab single agent on a neoadjuvant trial (UPCI #08-013, NCT #01218048) who are resistant to cetuximab therapy have significantly higher TGFβ concentration and a lower immunostimulatory cytokine profile in plasma, which endorses the use of combined therapy in those patients where EGFR blockade is not sufficient to reverse production of immunosuppressive cytokines and chemokines that feed the tolerant cellular network in the tumor microenvironment.

**Trial Registration**

ClinicalTrials.gov identifier NCT01218048.

### P379 Direct oncogene-targeted cancer killing and selective tumor Treg killing through the TNFR2 receptor via dominant antibody antagonists

#### Heather Torrey, Toshi Mera, Yoshiaki Okubo, Eva Vanamee, Rosemary Foster, Denise Faustman

##### Massachusetts General Hospital/Harvard Medical, Boston, MA, USA

###### **Correspondence:** Denise Faustman (faustman@helix.mgh.harvard.edu)

**Background**

A major barrier to cancer immunotherapy is lack of selective inhibitors of the regulatory T cells (Tregs) in the cancer microenvironment. New methods to directly kill tumors through novel surface oncogenes are also desirable. Tumor necrosis factor receptor 2 (TNFR2) is a target protein with restricted expression on the most potent tumor-infiltrating Tregs. On human tumors, it is a newly-discovered and broadly-expressed human oncogene.

**Methods**

We characterized the expression and the functional effects of the newly-created TNFR2 antibody antagonists on the tumor infiltrating Tregs of ovarian ascites compared to Tregs of peripheral blood from patients with ovarian cancer and healthy controls. We also investigated if well-known ovarian tumor cells lines express the TNFR2 oncogene and the effects of the TNFR2 antagonistic antibody on direct cancer killing.

**Results**

TNFR2 antagonists inhibited Treg proliferation with exponential potency and selectivity for the tumor microenvironment Tregs. Furthermore, common ovarian cancer cell lines such as OVCAR3 express the TNFR2 oncogene and were rapidly and completely killed by TNFR2 antagonistic antibodies, even at low concentrations.

**Conclusions**

Dominant TNFR2 antagonists demonstrate tumor-specific Treg depletion with heightened specificity for the tumor microenvironment over the Tregs of peripheral blood. Blocking TNFR2 signaling with antagonist antibodies also creates a novel tool to directly eliminate ovarian tumors expressing the TNFR2 oncogene.

### P380 Defining critical features of the immune microenvironment in melanoma

#### Robyn Gartrell^1^, Edward Stack^2^, Yan Lu^1^, Daisuke Izaki^3^, Kristen Beck^4^, Dan Tong Jia^4^, Paul Armenta^4^, Ashley White-Stern^4^, Yichun Fu^4^, Zoe Blake^1^, Douglas Marks^1^, Howard L Kaufman^5^, Bret Taback^1^, Basil Horst^1^, Yvonne M Saenger^6^

##### ^1^Columbia University Medical Center, New York, NY, USA; ^2^Perkin Elmer, Hopkinton, MA, USA; ^3^Columbia University, New York, NY, USA; ^4^Columbia University College of Physicians and Surgeons, New York, NY, USA; ^5^Rutgers Cancer Institute of New Jersey, New Brunswick, NJ, USA; ^6^New York Presbyterian/Columbia University Medical Center, New York, NY, USA

###### **Correspondence:** Robyn Gartrell (rdg2129@columbia.edu)

**Background**

Precise biomarkers are urgently needed to characterize the tumor immune micro-environment, both for prognostication and to predict the benefit of immunotherapeutic intervention. Multiplex immunohistochemistry (mIHC) allows for automated quantitation of phenotypes and spatial distributions of immune cells within formalin-fixed paraffin-embedded (FFPE) tissues. In early stage melanoma, it has been established that tumor infiltrating lymphocytes (TILs) confer a favorable prognosis but assessment of TILs is subjective and the correlation has not been robust enough to incorporate into American Joint Committee on Cancer (AJCC) guidelines.

**Methods**

**Fig. 71 (abstract P380).**
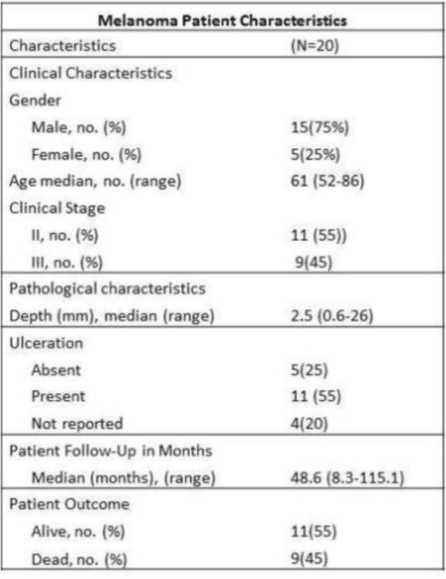
Patient Demographics

**Fig. 72 (abstract P380).**
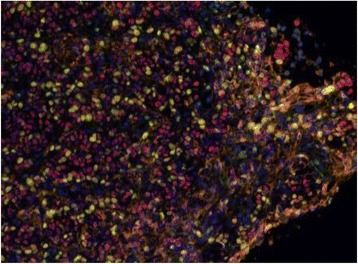
(Multiplex IHC of Melanoma Tumor. Melanoma tumor stained with DAPI (nuclear cell marker, blue) + CD68 (myeloid, green) + CD3 (T Cells, cyan) + CD8 (cytotoxic T Cells, magenta) + SOX10 (tumor, red) + Ki67 (proliferation marker, yellow) + HLA-DR (MHC II, orange)

**Fig. 73 (abstract P380).**
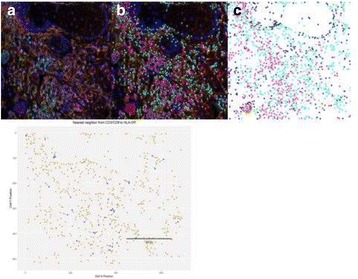
Analysis of images using algorithm’s within Inform software. **a** Multiplex IHC image stained for DAPI + SOX10 (tumor marker, red) + CD3 (T cell marker, cyan) + CD8 (cytotoxic T cell marker, magenta) + CD68 (myeloid marker, green) + Ki67 (marker of proliferation, yellow) + HLA-DR (marker of MHC-II, orange). **b** Phenotyped image with CD68 (green), CD3 (magenta), CD3/CD8 (light pink), HLA-DR (cyan), Tumor (red), other (blue). **c** Phenotype Map – data from Inform is exported to provide an X and Y coordinate for every cell in each image. **d** The X and Y coordinates can be analyzed using R Studio for Nearest Neighbor Analysis. In this image distance between CD3/CD8 (blue) and HLA-DR (orange) cells are being evaluated. Distance between cells is illustrated by a line

In order to test whether mIHC can improve on the accuracy of TIL quantitation for the purpose of biomarker development, we screened databases at the Herbert Irving Cancer Center (HICC) at Columbia University for early stage melanoma patients with available FFPE primary melanoma tissue and documented clinical follow up. We identified a preliminary population of 20 stage II-III melanoma patients diagnosed between 2000 and 2012, with characteristics shown in Fig. 71. At last follow up, 11 patients were alive and recurrence-free and 9 patients died with melanoma recurrence. 5 μm slides from either the primary biopsy or subsequent wide local excision procedure were stained using Opal multiplex IHC for DAPI, CD3 (LN10, Leica), CD8 (4B11, Leica), CD68 (KP1, Biogenex), SOX10 (BC34, Biocare), HLA-DR (LN-3, Abcam) and Ki67 (MIB1, Abcam) (Fig. 72). Cell phenotypes within representative fields were pre-selected by a trained dermato-pathologist and visualized using the Mantra quantitative pathology workstation (Perkin Elmer), and analysis of spatial distribution of CD3 + CD8+ cells analyzed as shown in Fig. 73 using inForm® image analysis software (Perkin Elmer), and Spotfire software (TIBCO).

**Results**

**Fig. 74 (abstract P380).**
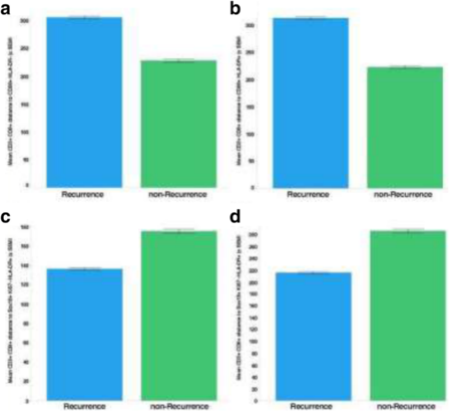
Spatial Distributions of CD3+CD8+ Cells Within Primary Melanoma Tumors. 20 primary melanoma tumors were stained as in Fig. 71 and mean distances between cell populations are shown with recurrent patients in blue and non-recurrent patients in green. Mean distances between CD3+CD8+ and CD68+HLA-DR- cells (**a**), CD3+CD8+ and CD68+HLA-DR+(**b**), CD3+CD8+ and SOX10+Ki67-HLA-DR-(**c**), and CD3+CD8+ and SOX10+Ki67-HLA-DR+(**d**) are shown

We find that CD3 + CD8+ cells are closer to CD68+ cells in patients who were recurrence-free at follow up (p < 0.0001). Conversely, CD3 + CD8+ cells are further from SOX10 + Ki67- tumor cells in recurrence-free patients (p < 0.0001). HLA-DR status of CD68+ or SOX10+ cells did not alter these spatial distributions (Fig. 74). Density of CD3 + CD8+ cells did not differ significantly between recurrent and non-recurrent groups in this small patient sample (p > 0.05).

**Conclusions**

Using proximity as a surrogate for interaction, these data would indicate that contact between T cells and CD68+ antigen presenting cells is more favorable to protective immunity than is contact between T cells and tumor cells. Further staining and analysis of 137 annotated tumor samples from the complete HICC cohort 2000–2012 is ongoing and results will be updated at time of presentation.

### P381 Induction of potent and durable anti-tumor immunity by intratumoral injection of STING-activating synthetic cyclic dinucleotides

#### Laura Hix Glickman^1^, David B Kanne^1^, Kelsey S Gauthier^1^, Anthony L Desbien^1^, Brian Francica^2^, George Katibah^1^, Leticia P Corrales^3^, Justin L Leong^1^, Leonard Sung^1^, Ken Metchette^1^, Shailaja Kasibhatla^4^, Anne Marie Pferdekamper^4^, Lianxing Zheng^5^, Charles Cho^4^, Yan Feng^5^, Jeffery M McKenna^5^, John Tallarico^5^, Steven Bender^4^, Chudi Ndubaku^1^, Sarah M McWhirter^1^, Charles G Drake^6^, Thomas F Gajewski^7^, Thomas W Dubensky^1^

##### ^1^Aduro Biotech, Berkeley, CA, USA; ^2^Johns Hopkins University School of Medicine, Baltimore, MD, USA; ^3^The University of Chicago, Chicago, IL, USA; ^4^Genomics Institute of the Novartis Research Foundation (GNF), San Diego, CA, USA; ^5^Novartis Institutes for BioMedical Research, Inc., Cambridge, MA, USA; ^6^Johns Hopkins University Cancer Center, Baltimore, MD, USA; ^7^University of Chicago Medical Center, Chicago, IL, USA

###### **Correspondence:** Laura Hix Glickman (lhixglickman@aduro.com)

**Background**

In human melanoma, spontaneous T cell infiltration into the tumor microenvironment (TME) is correlated with a type I interferon (IFN) transcriptional signature. Induction of IFN-β in this context is dependent upon activation of stimulator of interferon genes (STING), a critical component of the cytosolic DNA sensing pathway of the innate immune system. STING is activated by binding of cyclic dinucleotides (CDNs) produced by an intracellular enzyme, cGAS, in response to the presence of cytosolic pathogen or tumor-derived DNA. STING induction of IFN within the TME leads to the priming and activation of tumor antigen-specific CD8^+^ T cell immunity. We hypothesized that direct activation of STING in the TME by intratumoral (IT) injection of synthetic CDNs would induce potent anti-tumor immunity against a broad repertoire of an individual’s tumor antigenic milieu.

**Methods**

Through screening a panel of synthetic CDNs, we selected ADU-S100 (MIW815) for clinical development for its ability to broadly and potently activate all human STING alleles, elicit profound tumor regression of injected and distal lesions in aggressive mouse tumor models, and promote durable anti-tumor immunity.

**Results**

A bell-shaped ADU-S100 dose response curve was found to delineate regression of injected tumor, induction of tumor-specific CD8^+^ T cell immunity, and protection against autologous tumor challenge. *In vivo* mechanistic studies demonstrate that STING-mediated anti-tumor immunity is due in part to an acute pro-inflammatory TNF-α-mediated cytokine response, as well as a tumor-specific CD8^+^ T cell response. Studies in chimeric wild-type and STING^−/−^ mice showed that while STING signaling in the tumor stromal compartment contributes to acute tumor rejection, STING signaling in the hematopoietic compartment is required for induction of CD8^+^ T cell mediated anti-tumor immunity. Anti-tumor efficacy is enhanced by combination with immune checkpoint inhibitors, including α-PD-1 and α-CTLA-4, informing future clinical development.

**Conclusions**

The ability to elicit innate and T cell-mediated anti-tumor immunity via activation of STING in the TME demonstrates that CDNs have high translational potential for the treatment of patients with advanced/metastatic solid tumors. To this end, a phase I clinical study is in progress to evaluate the safety and tolerability and possible anti-tumor effects in subjects with cutaneously-accessible non UV-induced and UV-induced malignancies or lymphomas given repeated IT doses of ADU-S100.

**Trial Registration**

ClinicalTrials.gov identifier NCT02675439.

**Consent**

Written informed consent was obtained from the patient for publication of this abstract and any accompanying images. A copy of the written consent is available for review by the editor of this journal.

### P382 Modulation of NK cell exhaustion in metastatic melanoma tumors

#### Elena Gonzalez Gugel^1^, Charles JM Bell^2^, Adiel Munk^1^, Luciana Muniz^1^, Nina Bhardwaj^1^

##### ^1^Tish Cancer Institute, Icahn School of Medicine at Mount Sinai, New York, NY, USA; ^2^Murray Edwards College and School of Clinical Medicine, University of Cambridge, Cambridge, England, UK

###### **Correspondence:** Elena Gonzalez Gugel (elena.gonzalezgugel@gmail.com)

**Background**

Interventions such as checkpoint blockade have made a high impact in melanoma treatment, but a significant number of patients fail to respond or become resistant to therapy [1]. The tumor microenviroment in malignant melanoma promotes NK cell exhaustion, a state characterized by i) up-regulation of inhibitory receptors (TIM-3, KIRB1 and KIRNKAT2), ii) down-regulation of activating receptors (NKG2D and NKp46), IL-2R and transcription factors (T-bet and Eomes) and iii) loss of IFN-g production, proliferation and cytotoxicity. Interestingly, this exhaustion can be partially reversed by checkpoint blockade of Tim-3 [2]. Most strikingly, however, we also found that patients who have a clinical response to Ipilimumab treatment spontaneously restored their NK cell function, despite the fact that these cells express little or no CTLA-4 (or PD-1 or PDL-1) [3]. Here we investigate further aspects of NK cell exhaustion in human and animal models of melanoma.

**Methods**

Our results show that NK cells from human melanoma donors (MDs) are exhausted – a phenotype which is characterized by reduction of: (i) cytotoxicity (ii) IFN-γ production and iii) proliferation. In addition we show that NK cells from healthy donors (HDs) cultured with IL-2 increase expression levels of epigenetic modification indicators (HIST1H1C, HIST1H4I, ZNF286B, HIST2H2BE) whereas IL-2/IL-12 stimulated NK cells up regulated mRNA encoding cytokines (IFN-γ, IL-1β, IL-6 and TNF) and chemokines (CCL3, CXCL1, CXCL3), as well as the expression of different activating and inhibitory NK cells receptors. This is consistent with an activation phenotype that is deficient in MDs samples. We are also able to demonstrate that NK cells can became exhausted *in vitro* through a pathway driven by Ceacam-1 and found evidence of NK cell exhaustion in a B16BL6 melanoma model.

**Results**

The methodology used on this study included assays such as Mass cytometry measurement (CyTOF), Flow cytometry, Poly (A) RNAseq, Quantitative RT-PCR, Western Blot and tumor melanoma mice model.

**Conclusions**

We hypothesize that NK cell exhaustion becomes evident as melanomas progress in human and mouse models possibly driven by specific ligands within the tumor microenviroment. Fully appreciating the factors and immune suppressive mechanisms that contribute towards NK cell exhaustion in these patients will impact the development of drugs that facilitate effective anti-tumor immune responses.

NK cell exhaustion in murine melanoma model

Here we show how NK cells from B16BL6 tumor bearing mice became dysfunctional in progressive melanoma. NK cells expressed high levels of Tim-3 and Ceacam-1 when isolated from B16BL6 tumors compared with C57BL/6 J non-tumor mice blood after IL-2 overnight stimulation. In addition, NK cells from tumors showed reduced cytotoxicity against YAC-1 target cells compared with NK cells from blood WT non-tumor bearing mice. These findings of dysfunctional activity are consistent with data obtained in human melanoma patients.

**Fig. 75 (abstract P382).**
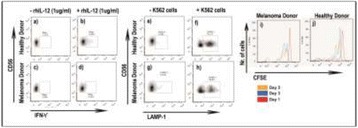
Features of exhaustion were detected on NK cells isolated from fresh melanoma donor (MD) blood. NK cells from MD show reduced cytotoxicity capacity (expression of LAMP-1/CD107a in response to co-culture with K562 cells), IFN-γ production in response to IL-12, and proliferation after IL-2 stimulation, compared with NK cells isolated from fresh healthy donor (HD) blood. These reduced functional properties are consistent with the concept that advanced disease is progressively associated with systemic immune suppression

**Fig. 76 (abstract P382).**
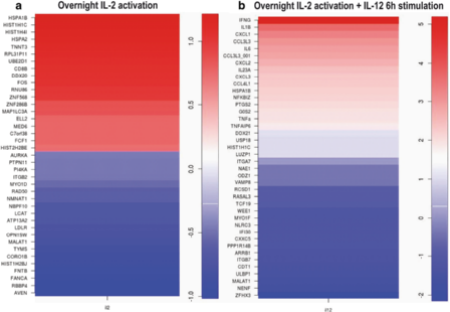
Transcriptome analysis in NK cells. Purified NK cells from HD were isolated and transcriptome analysis was performed under two different conditions **a**) IL-2 stimulated NK cells that showed evidence of priming through epigenetic modification indicators (HIST1H1C, HIST1H4I, ZNF286B, HIST2H2BE); **b** IL-2/IL-12 stimulated NK cells that up-regulated mRNA encoding cytokines (IFN-γ, IL-1ß, IL-6 and TNF) and chemokines (CCL3, CXCL1, CXCL3) consistent with an activation phenotype. This approach will be useful to show which pathways and genes are modulated during NK cell exhaustion when the analysis will be repeated in NK cells from MD as represented in Fig. 75

**Fig. 77 (abstract P382).**
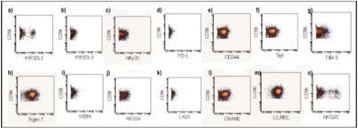
NK cell Phenotype analysis by CyTOF. Purified NK cells from HD were isolated and stained with a cocktail antibody containing a complete panel of 33 activating and inhibitory NK cell receptors including checkpoint molecules such as Tim-3, PD-1, DNAM-1, VISTA, LAG-3, TIGIT and Siglec-7 among others, as represented in this figure. This approach will be useful to determine the distribution of inhibitory receptors that impact NK cell exhaustion when the analysis will be repeated in NK cells from MD as represented in Fig. 75

**Fig. 78 (abstract P382).**
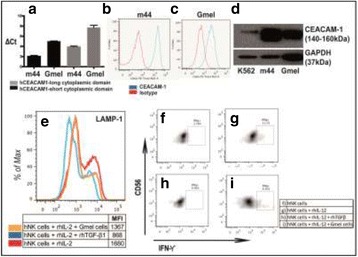
In vitro NK cell exhaustion. CEACAM-1 was recently discovered as a necessary co-receptor and ligand of Tim-3 on T cells [4] functioning both in cis and in trans to potentiate the inhibitory activity of Tim-3. Up regulated in primary melanoma, it is associated with poor prognosis for survival and increased invasive behavior [5]. CEACAM-1 also increases on IL-2 activated CD56hi NK cells [6] and functions to inhibit cytolytic activity after exposure to CEACAM-1-bearing tumor cells [7]. Here we first show high expression of short cytoplasmic Ceacam-1 isoform in the human melanoma cell lines m44 and Gmel. Interestingly, we found that similar to TGFb exposed NK cells, Gmel co-cultured NK cells also displayed clear signals of exhaustion such as significant loss of cytotoxic activity and reduced IFN-g production following IL-12 stimulation. Indicating the ability of tumors to suppress NK cells driven anti-tumor activity. This approach will be useful for gaining insight into mechanisms of NK cell exhaustion induced by melanoma cells

### P383 Stromal fibroblasts promote Wnt5a expression and suppress responses to anti-PD-1 antibody therapy in an autochthonous melanoma model

#### Fei Zhao^1^, Kathy Evans^1^, Christine Xiao^1^, Alisha Holtzhausen^2^, Brent A Hanks^1^

##### ^1^Duke University Medical Center, Durham, NC, USA; ^2^Lineberger Comprehensive Cancer Center, University of North Carolina, Chapel Hill, NC, USA

###### **Correspondence:** Brent A Hanks (hanks004@mc.duke.edu)

**Background**

TGF-β is a multifunctional cytokine that suppresses many facets of cellular immunity. These immunosuppressive properties have led to the development of immunotherapy regimens to evaluate the ability of type I TGF-β receptor (TBRI) inhibitors to augment the efficacy of checkpoint inhibition. The TGF-β cytokine also plays a role in cellular homeostasis and suppresses fibroblast expansion while promoting melanoma expression of Wnt5a, a ligand previously associated with resistance to anti-PD-1 antibody therapy in melanoma.

**Methods**

Using the BRAF^V600E^PTEN^−/−^ transgenic melanoma model, we investigated the impact of TBRI inhibition on anti-CTLA-4 antibody and anti-PD-1 antibody immunotherapy. Primary/metastatic tumor progression was correlated with the generation of anti-tumor immunity. Immunohistochemistry/flow cytometry was performed to assess the effect of TBRI inhibition on the tumor stroma. Melanoma and melanoma-associated fibroblast (MAF) lines were generated and utilized in co-transplant assays to examine the influence of MAFs on anti-PD-1 antibody therapy. Melanoma-MAF transwell assays were performed to investigate the underlying mechanism of anti-PD-1 antibody resistance.

**Results**

**Fig. 79 (abstract P383).**
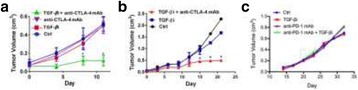
TBRI inhibition augments anti-CTLA-4 antibody but not anti-PD-1 antibody immunotherapy in the BRAF(V600E)-PTEN−/− transgenic melanoma model. **a** Assay 1. Short duration combination TGFBi (TGF-beta inhibitor)-anti-CTLA-4 antibody therapy. **b** Assay 2. Longer duration TGFBi-anti-CTLA-4 antibody therapy. **c** Combination TGFBi-anti-PD-1 antibody therapy

**Fig. 80 (abstract P383).**
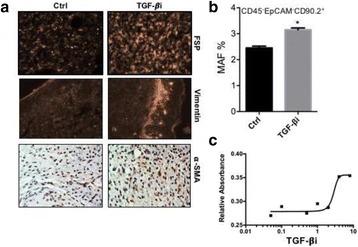
TBRI Inhibition Induces Melanoma-associated Fibroblast (MAF) Expansion. **a** Immunofluoresence and immunohistochemical analysis of MAF markers in resected BRAF(V600E)PTEN−/− tumors treated with vehicle ctrl vs TGFBi. **b** Flow cytometry quantitation of MAFs based on the CD45, EpCAM, and CD90.2 surface markers. **c** TGFBi promotes MAF proliferation in culture

**Fig. 81 (abstract P383).**
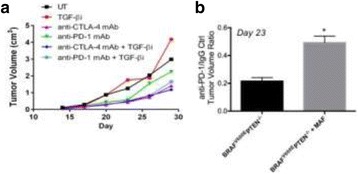
TBRI Inhibition Augments anti-PD-1 Antibody Therapy in a Stroma-poor BRAF(V600E)-PTEN−/− Transplant Model of Melanoma that is Reversed with MAF Co-Transplantation. A. Anti-PD-1 and Anti-CTLA-4 antibody therapy in combination with TGFBi in a cell line-derived transplant model of BRAF(V600E)PTEN−/− melanoma. B. Co-transplantation of MAFs with the BRAF(V600E)PTEN−/− melanoma cell line suppresses the efficacy of anti-PD-1 antibody therapy

**Fig. 82 (abstract P383).**
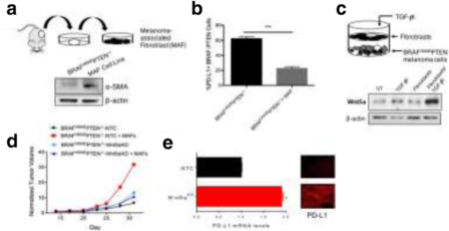
MAFs Suppress PD-L1 Expression and Promote Melanoma Progression in a Wnt5a-dependent Manner. **a** Generation of MAFs from resected BRAF(V600E)PTEN−/− melanoma tissues. **b** Transwell cultures of MAFs and BRAF(V600E)PTEN−/− melanoma cells followed by PD-L1 flow cytometry. **c** Transwell culture of MAFs and BRAF(V600E)PTEN−/− melanoma cells +/− TGFBi followed by Wnt5a Western blot analysis. **d** Primary tumor growth of co-transplanted MAFs with control BRAF(V600E)PTEN−/− melanoma cells (NTC) vs Wnt5a-silenced BRAF(V600E)PTEN−/− melanoma cells (Wnt5aKD). **e** qrt-PCR and immunofluorescence of PD-L1 expression by BRAF(V600E)PTEN−/−NTC melanomas vs BRAF(V600E)PTEN−/−Wnt5aKD melanomas

TBRI inhibition synergistically enhances anti-CTLA-4 antibody immunotherapy in the BRAF^V600E^PTEN^−/−^ transgenic melanoma model but fails to augment anti-PD-1 antibody therapy in this same model (Fig. 79). Immunohistochemistry and flow cytometry of TBRI-treated melanomas reveals an expansion of the MAF population while *in vitro* experiments show TBRI inhibition to enhance fibroblast proliferation (Fig. 80). TBRI inhibition augments anti-PD-1 antibody therapy in a stromal-poor BRAF^V600E^PTEN^−/−^ transplant tumor model while the co-transplantation of a BRAF^V600E^PTEN^−/−^ melanoma-derived MAF cell line reverses this effect (Fig. 81). MAFs were found to express high levels of TGF-β which correlates with melanoma expression of Wnt5a in resected melanoma tissues. Melanoma-MAF co-cultures revealed that TBRI-treated MAFs promote melanoma expression of Wnt5a and suppress PD-L1 expression both *in vitro* and *in vivo* (Fig. 82). Silencing of Wnt5a expression by the BRAF^V600E^PTEN^−/−^ melanoma model enhances PD-L1 expression and eliminates the impact of MAFs on responses to anti-PD-1 antibody therapy.

**Conclusions**

This work illustrates the importance of the tumor stroma on the efficacy of anti-PD-1 antibody therapy and that pharmacologic modulation of the melanoma microenvironment can have significant effects on anti-PD-1 antibody therapy outcomes. TGF-β-mediated upregulation of Wnt5a represents an important paracrine signaling pathway that can impact checkpoint inhibitor efficacy in the melanoma microenvironment. TBRI inhibition should be further evaluated in combination with anti-CTLA-4 antibody therapy and Wnt signaling blockade represents a promising approach for augmenting anti-PD-1 antibody therapy in future clinical trials.

### P384 Inhibitors of B cell activation reduce tumor growth and attenuate pro-tumorigenic phenotype of tumor immune infiltrates in a syngeneic mouse model of ovarian cancer

#### Nathalie Scholler^1^, Catherine Yin^1^, Pien Van der Meijs^2^, Andrew M Prantner^3^, Cecile M Krejsa^4^, Leia Smith^4^, Brian Johnson^5^, Daniel Branstetter^6^, Paul L Stein^1^

##### ^1^Stanford Research Institute, Menlo Park, CA, USA; ^2^Rijksuniversiteit Groningen, Groningen, Amsterdam, Netherlands; ^3^University of Pennsylvania, Philadelphia, PA, USA; ^4^Acerta Pharma, Bellevue, WA, USA; ^5^University of Washington, Seattle, WA, USA; ^6^Cynosure Clinical Research LLC, North Bend, WA, USA

###### **Correspondence:** Nathalie Scholler (nathalie.scholler@sri.com)

**Background**

Recent findings support a pro-tumorigenic role for regulatory B cells [1]. B cell signaling is dependent on Bruton's tyrosine kinase (BTK) and phosphoinositide 3-kinase delta (PI3Kδ) activity. Acalabrutinib is a highly selective, potent BTK inhibitor in clinical development for treatment of hematologic malignancies, solid tumors, and autoimmune diseases; ACP-319 is a selective inhibitor of PI3Kδ. We hypothesized that these agents, which inhibit B cell activation and alter myeloid cell signaling, might improve antitumor responses with checkpoint inhibition (anti-PD-L1 mAb) for tumors containing regulatory B cell infiltrates.

**Methods**

To assess regulatory B cells in the syngeneic ID8 ovarian tumor model, we adoptively transferred IL-10/GFP reporter B cells into mice deficient for functional B cells (μMT) then orthotopically implanted the mice with ID8 cells; GFP expression was analyzed by histology. Next, C57BL/6 mice orthotopically implanted with luciferase-transduced ID8 cells were treated with acalabrutinib +/− ACP-319 in combination with anti-PD-L1. Both oral agents were dosed 15 mg/kg twice daily; anti-PD-L1 (10 F.9G2) was dosed intraperitoneally 200 μg every 3 days. Tumor growth was monitored by *in vivo* bioluminescence imaging and *ex vivo* caliper measurements of ovaries and fallopian tubes. Tumor-immune infiltrates were characterized by flow cytometry (peritoneal lavages) and histologic analysis (tumor sections).

**Results**

In μMT mice reconstituted with IL-10/GFP, many tumor-infiltrating cells expressed GFP, consistent with a regulatory B cell phenotype in the ID8 tumor microenvironment, whereas GFP expression was not observed in paired spleens. Significant tumor growth reductions (>50 %) were observed in mice treated for 10 weeks with acalabrutinib (p = 0.027), acalabrutinib + anti-PD-L1 (p = 0.037), acalabrutinib/ACP-319 (p = 0.012) or acalabrutinib/ACP-319 + anti-PD-L1 (p = 0.036), but not with anti-PD-L1 alone (p = 0.104), compared with vehicle-treated controls. All anti-PD-L1 groups had increased CD8+ T cells in peritoneal lavages. Acalabrutinib/ACP-319 treatment decreased B cells in peritoneal lavages by 70 % compared with vehicle, and acalabrutinib synergized with anti-PD-L1 to decrease PD-L1+ macrophages in bone marrow (90 %). In mice treated with acalabrutinib/ACP-319 + anti-PD-L1, histology scores were significantly higher (p ≤ 0.031) for immune infiltrates and fibrosis, compared with controls. Tumor-infiltrating B cells, macrophages, T cells, and FoxP3-positive cells were decreased in the acalabrutinib/ACP-319 group. Treatment with acalabrutinib/ACP-319 + anti-PD-L1 increased tumor-infiltrating CD8+ T cells (p = 0.026).

**Conclusions**

These results suggest that inhibition of BTK and PI3Kδ targets both regulatory B cell subsets and myeloid cells in the tumor microenvironment. Combined with anti-PD-L1, acalabrutinib alone or with ACP-319 promotes an anti-tumor response against ovarian cancer.

**References**

1. Gunderson: **BTK–Dependent Immune Cell Cross-talk Drives Pancreas Cancer.** *Cancer Discov* 2016, **6(3)**: 270–285.

### P385 Use of experimental tumor model to characterize the effects of small-molecule CD73 inhibitors on tumor biology and immune infiltrate

#### Juan C Jaen, Joanne BL Tan, Ada Chen, Yu Chen, Timothy Park, Jay P Powers, Holly Sexton, Guifen Xu, Steve W Young, Ulrike Schindler

##### Arcus Biosciences, Hayward, CA, USA

###### **Correspondence:** Juan C Jaen (jjaen@arcusbio.com)

**Background**

High levels of extracellular ATP (eATP) have been documented in many solid tumors. eATP, which enhances activation of antigen-presenting cells, is efficiently hydrolyzed to adenosine by extra-cellular CD39 (ATP → AMP) and CD73 (AMP → adenosine). Adenosine is a potent inhibitor of T cell and NK activation. Thus, CD73 has drawn interest as a next-generation immune-oncology target. We describe the CT26 tumor model to characterize the effects of CD73 inhibition on tumor biology and plasma AMP/adenosine levels as markers of systemic CD73 inhibition.

**Methods**

Human PBMC and mouse spleen/blood/tumor leukocytes were isolated using standard methods. Cell markers were quantified by flow cytometry. Therapeutic treatment of subcutaneous (106 cells) CT26 tumors was initiated at volumes ~100 mm3; Cohorts: vehicle + isotype control; CD73i A000830 + isotype control; vehicle + α-PD-1; A000830 + α-PD-1).

**Results**

CD73 expression was high in all subsets of human blood CD8+ T cells, B cells, and Treg, but not Treg-depleted CD4+ T cells. CD39 was broadly expressed on lymphoid and myeloid cells, with highest levels on effector Treg, monocytes, neutrophils, B cells, and activated CD8+ T cells. CD73 expression was higher on C57BL/6 than Balb/c mouse spleen and blood T cells (all subsets). On B and NK cells, CD73 expression was similar between both mouse strains. In Balb/c mice carrying CT26 tumors, CD73 expression in TILs was limited to T cells and MDSC; CD73 expression was increased on CD8+ TIL relative to splenic CD8+ T cells from tumor-bearing mice, and both were higher than in normal splenic CD8+ T cells. Human blood CD8+ T cells exhibit robust CD39 and CD73 activity, which can be blocked by CD39 and CD73 inhibitors, respectively. Mouse spleen CD8+ T cells also showed a lower but detectable level of CD39 and CD73 activity. Plasma AMP:adenosine ratios were ~15X higher in CD73 KO than in gender- and age-matched WT mice. Similarly, plasma AMP:adenosine ratios were increased in WT mice dosed with a CD73 inhibitor. Administration of a novel small-molecule CD73 inhibitor (A000830) to CT26 tumor-bearing mice resulted in significant reduction in tumor volume, with an even larger effect observed in combination with α-PD-1. Co-administration of A000830 with α-PD-1antibody increased the number of TIL relative to control or single-treatment tumors.

**Conclusions**

Syngeneic CT26 colon tumors provide an excellent model to assess the effects of CD73 inhibition on tumor growth and immune infiltration. Plasma levels of AMP/adenosine provide a simple way of monitoring systemic CD73 inhibition.

### P386 WISP1 stimulates melanoma cell invasion by promoting epithelial–mesenchymal transition (EMT) through both autocrine and paracrine signaling

#### Wentao Deng, David John Klinke

##### West Virginia University, Morgantown, WV, USA

###### **Correspondence:** David John Klinke (david.klinke@mail.wvu.edu)

**Background**

A dire prognosis is associated with the metastasis of human melanoma. Melanoma invasion and metastasis is coordinated by soluble signals present within the tumor microenvironment. One soluble signal is WNT1 inducible signaling pathway protein 1 (WISP1), a secreted matricellular protein that is elevated in a variety of cancers. Functionally, Wisp1 is secreted in response to disruption of adherens junctions [1], such as occurs during invasion, but also represses anti-tumor immunity through a paracrine mechanism [2]. Here, our goal was to parse autocrine, that is the impact on intrinsic tumor cell behavior, from paracrine, that is a mediator of intercellular communication, effects of WISP1 on tumor growth.

**Methods**

Wisp1 in mouse melanoma B16 cells was knocked out using the CRISPR/Cas9 system to evaluate its role in melanoma progression and metastasis using *in vitro* and *in vivo* assays. We also knocked out or over-expressed Wisp1 in mouse fibroblasts to compare the autocrine and paracrine effects on *in vitro* tumor cell invasion assays using fibroblast conditioned medium.

**Results**

*In vitro*, Wisp1 disruption increased tumor cell proliferation and anchorage-independent growth but repressed its wound healing, migration and invasion. *In vivo*, B16 cells without Wisp1 exhibited a lower propensity for spontaneous metastasis to the lung. With Wisp1 knockout, certain epithelial–mesenchymal transition (EMT) markers were down-regulated. This suppression could be partially rescued by supplementation of Wisp1, suggesting an autocrine mechanism of Wisp1 for EMT gene activation and enhanced tumor invasion. Further work with multiple metastatic melanoma lines using conditional medium from mouse fibroblast NIH3T3 cells with Wisp1 knockout or over-expression also supported a paracrine scenario in which fibroblast-secreted Wisp1 promotes tumor invasion. Wisp1 from fibroblasts activated both Akt and MEK/ERK signaling, repressed E-cadherin, and upregulated certain EMT genes. Kinase inhibition confirmed the importance of both pathways for EMT gene regulation.

**Conclusions**

Collectively, the results support a pleiotropic regulation of melanoma invasion and metastasis by WISP1 through directly induction of EMT in tumor cells and indirect promotion of tumor invasion by fibroblasts within the tumor microenvironment.

**References**

1. Klinke DJ, Horvath N, Cuppett V, Wu Y, Deng W, Kanj R: **Interlocked positive and negative feedback network motifs regulate β-catenin activity in the adherens junction pathway**. *Mol Biol Cell* 2015, **26**:4135–4148.

2. Kulkarni YM, Chambers E, McGray AJR, Ware JS, Bramson JL, Klinke DJ: **A quantitative systems approach to identify paracrine mechanisms that locally suppress immune response to Interleukin-12 in the B16 melanoma model**. *Integr Biol (Camb)* 2012, **4**:925–936.

### P387 Jak/STAT signaling as a mediator of immune suppression from pancreatic stellate cells in vitro and caerulein-induced pancreatitis in vivo

#### Hannah M Komar^1^, Thomas Mace^1^, Gregory Serpa^1^, Omar Elnaggar^1^, Darwin Conwell^1^, Philip Hart^2^, Carl Schmidt^2^, Mary Dillhoff^2^, Ming Jin^2^, Michael C Ostrowski^1^, Gregory B Lesinski^1^

##### ^1^The Ohio State University, Columbus, OH, USA; ^2^The Ohio State University Wexner Medical Center, Columbus, OH, USA

###### **Correspondence:** Hannah M Komar (hnnhkmr@gmail.com)

**Background**

Pancreatic stellate cells (PSC) are implicated in the pathogenesis of pancreatic ductal adenocarcinoma (PDAC) and chronic pancreatitis (CP). These cells orchestrate fibrosis and contribute to immune suppression in the PDAC microenvironment. As such, PSC may facilitate resistance to immunotherapy. Soluble factors from these cells can activate inflammatory Jak/STAT and MAPK signaling in an autocrine and paracrine manner. We hypothesized that inhibiting the Jak/STAT or MAPK pathways may limit features of inflammation and immune suppression in the diseased pancreas.

**Methods**

*In vitro* studies utilized immortalized PSC lines, and primary PSC from patients with CP and PDAC. Cytokine/chemokine expression was analyzed in supernatants via multiplex immunoassay. PSC supernatants were utilized to generate functionally suppressive MDSC *in vitro*. PSC were treated with ruxolitinib (Jak1/2 inhibitor), or MEK162 (MEK inhibitor) purchased commercially. Cell viability was assessed via MTT assay. Immunoblot analysis was utilized to evaluate the effect of inhibitors on STAT3, MAPK, and apoptotic pathways. To model pancreatic inflammation, we used the well-characterized *in vivo* model of caerulein-induced pancreatitis. This involved 6 hourly intraperitoneal injections of 50 μg/kg caerulein 3 days/week for 5 weeks. During the final week of caerulein, mice received oral gavage of ruxolitinib at 90 mg/kg 2X/day. Pancreata from these mice were stained for H&E, Masson’s Trichrome, and αSMA.

**Results**

All PSC displayed constitutive activation of Jak/STAT and MAPK pathways. PSC supernatants contained robust levels of soluble factors including MCP-1, IL-6, and VEGF (up to 31,000, 19,000, and 800 pg/mL, respectively). Culture of PBMC from healthy human donors with PSC supernatants promoted differentiation of MDSC (CD33^+^CD11b^+^HLADR^lo^) that functionally suppressed proliferation of autologous, CD3/CD28-stimulated T cells. This effect was blocked by adding IL-6 neutralizing antibody or STAT3 inhibitor to culture media, indicating a role for IL-6/STAT3 signaling in PSC-derived immune suppression. Treatment of PSC with ruxolitinib reduced STAT3 phosphorylation, decreased growth (40 % growth after 72 hours) but did not induce apoptosis. Instead, PSC exhibited a dose-dependent decrease in αSMA, a marker of PSC activation. In contrast, treatment with MEK162 had no effect on growth or activation. In a murine model of caerulein-induced inflammation and pancreatitis, short-term ruxolitinib treatment resulted in significantly less acinar loss and fibrosis.

**Conclusions**

These data suggest that the Jak/STAT pathway promotes immune suppressive features via stellate cells in the pancreatic microenvironment. These findings support future studies whereby Jak/STAT inhibition may reduce desmoplasia and augment the response to immunotherapy.

### P388 Role of STAT1 induced CXCL10 in tumor progression and the immune microenvironment of high-grade grade serous ovarian cancer

#### Madhuri Koti, Katrina Au, Nichole Peterson, Peter Truesdell, Gillian Reid-Schachter, Charles Graham, Andrew Craig, Julie-Ann Francis

##### Queen's University, Kingston, ON, Canada

###### **Correspondence:** Madhuri Koti (kotim@queensu.ca)

**Background**

We previously reported the presence of a STAT1 associated distinct T helper type I tumor immune microenvironment (TME) associated with chemotherapy response in high-grade serous ovarian cancer in a cohort of chemotherapy naïve tumors from 734 patients. We also demonstrated that STAT1 enhanced the prognostic relevance of cytotoxic tumor infiltrating T lymphocytes. The current study was performed with an aim to elucidate the role of STAT1 induced CXCL10 in HGSC tumor immune microenvironment in the established ID8 mouse model of HGSC.

**Methods**

ID8 mouse ovarian cancer cells were transduced with lentiviral constructs to overexpress CXCL10. *In vitro* cell proliferation and migration assays were performed to determine cancer cell intrinsic effects of increased CXCL10 expression. For *in vivo* studies, the CXCL10 overexpressing and control ID8 cells were implanted via intra-peritoneal injection into immunocompetent C57BL/6 mice. Ascites samples collected at each endpoint were subjected to multiplex cytokine analysis using a mouse 31-plex assay. RNA isolated from tumor tissues was subjected to immune transcriptome profiling using the NanoString mouse pan cancer immune profiling panel consisting of over 500 immune and 40 reference genes. The effect of CXCL10 on the tumor vasculature of HGSC TME was evaluated by CD31 immunofluorescence staining.

**Results**

A significant decrease in tumor progression and metastatic tumor nodule formation in mice injected with ID8 CXCL10 overexpressing cells compared to those with ID8 vector control cells was observed. Multiplex cytokine analysis of malignant ascites showed differential expression of IL-6, VEGF and CXCL9 between the two groups. CD31 endothelial cell marker staining showed differences in tumor vasculature between the two groups. Immune transcriptomic profiling of tumors from both groups identified distinct expression profiles in genes associated with cytokines, chemokines, interferons, T cell function and apoptosis between the two groups.

**Conclusions**

Our findings provide evidence that the differential expression of CXCL10 in HGSC tumors potentially contributes to altering tumor progression and associated tumor immune microenvironment. Findings from this study provide the basis for future studies aimed at understanding mechanisms underlying differential tumor STAT1 and CXCL10 expression and its role in pre-existing tumor immunologic diversity and aid in design of novel immunomodulatory therapies in HGSC.

**Acknowledgements**

Funding for the study was provided by the Cancer Research Society.

### P389 Pre-implantation factor (PIF) – blocks proliferation and enhances tumor footprint in cultured human metastatic melanoma lymph nodes – translational aspects

#### Beatrix Kotlan^1^, Timea Balatoni^1^, Emil Farkas^1^, Laszlo Toth^1^, Mihaly Ujhelyi^1^, Akos Savolt^1^, Zoltan Doleschall^1^, Szabolcs Horvath^1^, Klara Eles^1^, Judit Olasz^1^, Orsolya Csuka^1^, Miklos Kasler^1^, Gabriella Liszkay^1^, Eytan Barnea^2^

##### ^1^National Institute of Oncology, Budapest, Hungary; ^2^BioIncept, Cherry Hill, NJ, USA

###### **Correspondence:** Beatrix Kotlan (kotlanb@netscape.net)

**Background**

Embryonic and cancer cell phenotypes share common features. Whereas during embryogenesis proliferation/differentiation is regulated, in cancer altered programming leads to invasion and immortality. Our premise is that embryo-derived, immune-regulatory PIF regulates this process. Synthetic PIF peptide transposes endogenous regulatory features in clinically-relevant models and is in FDA fast track clinical trial for autoimmune disease (NCT02239562). Having shown that PIF reduces metastatic melanoma (NSG/H199 model), human melanoma cultures were examined, towards its translation to therapeutic use.

**Methods**

Following ethical committee approval (ETT TUKEB 1642-02/2010), minor tissue samples from surgically removed lymph nodes of patients with metastatic melanoma were investigated. Effect on proliferation was examined in cell cultures with or without PIF and further analyzed by molecular and immunological assays.

**Results**

PIF treatment (250 nm) delayed tumor outgrowth in a time-dependent manner in the 8 investigated cultures. Importantly, PIF increased the expression of disialylated glycosphingolipid tumor associated antigens in melanoma cells in the majority of the tested cases, as evidenced by immunofluorescence FACS cell sorting and confocal laser microscopy (enhanced footprint). Assessment of PIF effect on tumor associated changes in gene expression is ongoing.

**Conclusions**

PIF reduces proliferation and may directly or indirectly promote glycosphingolipid-type tumor associated antigen expression. By this enhanced tumor footprint visibility, PIF may help trigger immune-improved anticancer response. Overall, PIF use for tumor targeting and improved antigen expression may facilitate cancer treatment.

**Acknowledgements**

HJLCT Melanoma Research Award 2010, Fulbright Research Grant No: 1214104/2014, BioIncept LLC sPIF (proprietary).

**References**

1. Perry JK, Lins RJ, Lobie PE, Mitchell MD: **Regulation of invasive growth: similar epigenetic mechanisms underpin tumour progression and implantation in human pregnancy.** *Clin Sci* 2009, **118**:451–457.

2. Shainer R, Almogi-Hazan O, Berger A, *et al.*: **PIF Provides Comprehensive Protection against Radiation Induced Pathologies.** *Oncotarget* 2016, DOI:10.18632/oncotarget.10635.

3. Kotlan B, Liszkay G, Blank M, Csuka O, Balatoni T, Toth L, *et al.*: **The novel panel assay to define tumor-associated antigen-binding antibodies in patients with metastatic melanomas may have diagnostic value.** *Immunol Res* 2015, **61**:11–23.

4. Mueller M, Zhou J, Yang L, Gao Y, Wu F, Schoeberlein A, *et al.*: **PreImplantation Factor Promotes Neuroprotection by Targeting microRNA Let-7.** *Proc Natl Acad Sci USA* 2014, **111(38)**:13882–13887.

### P390 PD-L1 expressing myeloid cells limit response to CSF-1R targeted therapy

#### Sushil Kumar^1^, Takahiro Tsujikawa^1^, Collin Blakely^2^, Patrick Flynn^1^, Reid Goodman^1^, Raphael Bueno^3^, David Sugarbaker^4^, David Jablons^5^, V. Courtney Broaddus^2^, Brian West^6^, Lisa M. Coussens^1^

##### ^1^Oregon Health & Science University, Portland, OR, USA; ^2^University of California, San Francisco School of Medicine, San Francisco, CA, USA; ^3^Brigham and Women's Hospital, Boston, MA, USA; ^4^Baylor Clinic, Houston, TX, USA; ^5^UCSF Helen Diller Family Comprehensive Cancer Center, San Francisco, CA, USA; ^6^Plexxikon Inc., Berkeley, CA, USA

###### **Correspondence:** Lisa M. Coussens (coussenl@ohsu.edu)

**Background**

Malignant mesothelioma (MM) is an inflammation-associated cancer that is markedly resistant to “standard-of-care” chemotherapy with extremely poor prognosis.

**Methods**

We utilized MM patient biopsies and an immunocompetent MM mouse model to investigate the role of tumor-infiltrating leukocytes in regulating response to therapy.

**Results**

We report herein that chemotherapy leads to increased frequency of colony stimulating factor-1 receptor (CSF-1R)^+^ macrophages in human MM, as well as in an immunocompetent mouse model of MM. While therapeutic blockade of CSF-1R instigated activation of CD8^+^ T cells in primary tumors resulting in ~50 % decreased primary tumor burden, combined therapy did not improve animal survival as therapy failed to reduce pulmonary metastasis. To investigate mechanisms underlying the tissue-specific response to combined chemo/CSF1R inhibition, we identified programmed death ligand 1 (PD-L1) as an abundant immune checkpoint molecule expressed by alveolar macrophages in lung metastases. Based on this, we hypothesized that combination blockade of PD-L1 with CSF-1R would improve survival by driving CD8^+^ T cell activation and cytotoxicity in both primary and metastatic tumors thereby extending survival and improving outcome. Indeed, combination therapy using CSF-1R/PD-L1 inhibitors enhanced activated CD8^+^ T cell frequency and further reduced tumor burden at both primary and metastatic sites resulting in increased animal survival. Activated CD8^+^ T cells, in both primary and metastatic tumors, exhibited biomarkers of “reinvigoration” marked by co-expression of Ki-67 with PD-1 and EOMES. CD8^+^ T cells were essential for therapeutic benefit of CSF-1R/PD-L1 combination therapy since their depletion subverted therapy response.

**Conclusions**

Tissue-specific tumor niches pose distinct requirements for generating adaptive immune responses and/or perturbing/abrogating tumor growth. Results from these studies highlight a rationale for combination therapy where individual constituents target different immunosuppressive microenvironments to impact BOTH primary tumor responses as well as thwarting secondary tumor growth.

**Acknowledgements**

SK acknowledges support from the OHSU Knight Cancer institute and Collins Medical Trust. LMC acknowledges support from the NIH/NCI, DOD BCRP Era of Hope Scholar Expansion Award, Susan G. Komen Foundation, Stand Up To Cancer – Lustgarten Foundation Pancreatic Cancer Convergence Dream Team Translational Research Grant, Breast Cancer Research Foundation, and the Brenden-Colson Center for Pancreatic Health.

### P391 CD56+ cell infiltration correlates with a worse prognosis in cholangiocarcinoma

#### Paul R. Kunk^1^, Joseph M Obeid^1^, Kevin Winters^1^, Patcharin Pramoonjago^1^, Mark E Smolkin^2^, Edward B Stelow^1^, Todd W Bauer^1^, Craig L Slingluff Jr^3^, Osama E Rahma^4^

##### ^1^University of Virginia, Charlottesville, VA, USA; ^2^Department of Public Health Sciences, University of Virginia, Charlottesville, VA, USA; ^3^Division of Surgical Oncology, University of Virginia, Charlottesville, VA, USA; ^4^Dana Farber Cancer Institute/Harvard University, Boston, MA, USA

###### **Correspondence:** Paul R. Kunk (prk5r@virginia.edu)

**Background**

Cholangiocarcinoma (CC) is an uncommon malignancy increasing in incidence and characterized by rapid progression. The tumor immune microenvironment (TME) has been extensively studied in a variety of cancers, but characterization of the CC TME is not well understood. We aimed to perform a comprehensive analysis of the TME of CC in order to identify new potential immunotherapy targets.

**Methods**

A retrospective analysis was conducted of CC tumor samples at the University of Virginia from 2000–2014. Tissue microarrays (TMAs) were constructed of 3–4 cores from each tumor and were stained by immunohistochemistry (IHC) for a number of immune cells and markers of immune activation or inhibition (17 total). These were grouped into a positive effector signal (CD4, CD8, CD14, CD40, CD45, CD45RO, CD56, CD68, CD69, CD83, granzyme, and OX40) and a negative suppressor signal (FoxP3, CD163, TIM3, LAG3, and IDO). TMAs were scanned using the Leica SCN400 and was analyzed using the Digital Image Hub software. Stain intensity thresholds for determining positive cells were determined by two users and recorded as an average of all cores from each tumor. Correlation with overall survival was assessed using Cox-proportional hazard with p-values < 0.05 being considered as statistically significant.

**Results**

Ninety-nine CC tumors were available for analysis. Median age was 68 years old with 26 intrahepatic, 37 hilar and 36 distal CC. In univariate analysis, increasing CD56^+^ (HR: 1.75; CI: 1.24-2.46), p + (HR: 1.07; CI: 1.02-1.12, p = 0.007) infiltrations significantly correlated with worse survival. CD163^+^ infiltration trended towards a significant decrease in survival (HR: 1.19; CI: 0.9-1.43, p = 0.06) while CD40^+^ infiltration trended towards a significant increase in survival (HR: 0.66; CI: 0.42-1.05, p = 0.08).

**Conclusions**

CD56+ cell infiltration into cholangiocarcinoma correlates with a worse prognosis. Further studies including a multivariate analysis and multicolored IHC are planned to better understand to the role of CD56^+^ cells in CC.

### P392 Defining the immune microenvironment in patients with acute myeloid leukemia

#### Adam Lamble^1^, Yoko Kosaka^1^, Fei Huang^2^, Kate A Saser^2^, Homer Adams^2^, Christina E Tognon^1^, Ted Laderas^1^, Shannon McWeeney^1^, Marc Loriaux^1^, Jeffery W Tyner^1^, Brian J Druker^3^, Evan F. Lind^1^

##### ^1^Oregon Health & Science University, Portland, OR, USA; ^2^Janssen Pharmaceutical R&D, Spring House, PA, USA; ^3^Knight Cancer Institute/Howard Hughes Medical Institute, Portland, OR, USA

###### **Correspondence:** Evan F. Lind (linde@ohsu.edu)

**Background**

The aim of our project is to identify the phenotypic and functional status of immune cells in the bone marrow of patients with acute myeloid leukemia (AML). The goal of the immunophenotyping is to identify the relevant myeloid and lymphoid cell subsets present in the marrow of patients with AML, understand their differentiation and activation status, and to quantify the density of immune modifiers on cells of interest. The goal of the functional assays is to further define the status of T cells in the AML microenvironment by evaluating the responsiveness of these cells to stimulation. Additionally, these studies assess the impact of costimulatory or immune checkpoint inhibitors on the proliferative and cytokine production capacity of T cells, as well as tumor cells, resident in AML bone marrow.

**Methods**

Immunophenotyping was performed using mass cytometry (CyTOF). Primary AML bone marrow samples were stained with four distinct antibody panels to identify the status of over 70 cell surface and intracellular proteins and cytokines. Functional assays measured both the proliferative capacity and cytokine production profile of AML bone marrow T cells in response to CD3 stimulation in the context of the tumor microenviroment. Analysis of the changes to the baseline T cell functional profile in the presence of various checkpoint inhibitors and immune stimulatory antibodies was measured.

**Results**

Our initial studies have begun to identify the major myeloid and lymphoid subsets present in the marrow of patients with AML with a focus on the detailed description of T cell subsets and activation status. We have identified the cellular expression of various immune checkpoint proteins and inhibitory cytokines on both myeloid and lymphoid cellular subsets. The functional studies have identified a subset of AML patient samples where T cells are impaired in their ability to proliferate in response to CD3 ligation. We have found differential effects of several immune checkpoint inhibitor antibodies on T cell proliferation and cytokine production.

**Conclusions**

These results provide the beginning of a functional and phenotypic description of the immune status in the marrow of patients with AML. We have identified potential immune targets in AML. This is a report of initial findings of our study; accrual of patient samples is ongoing to expand the power of our observations.

### P393 Tim-3 + CD4 + CD25hiFOXP3+ regulatory T cells underlies enhanced suppressive function in HNSCC patients

#### Zhuqing Liu^1^, Shanhong Lu^1^, Lawrence P Kane^2^, Robert L Ferris^3^

##### ^1^University of Pittsburgh Medical Center, Hillman Cancer Center, Pittsburgh, PA, USA; ^2^Department of Immunology, University of Pittsburgh, Pittsburgh, PA, USA; ^3^University of Pittsburgh, Pittsburgh, PA, USA

###### **Correspondence:** Zhuqing Liu (liuz5@upmc.edu)

**Background**

Regulatory T (Treg) cells are essential for maintenance of immune homeostasis, and are important suppressive cells among tumor infiltrating lymphocytes (TIL). Tim-3 was first identified on Th1 T cells as a negative regulator of type 1 immunity. Tim-3 can also be expressed by some Tregs, but the functional status of human Tregs expressing Tim-3 remains unclear.

**Methods**

Treg were sorted from fresh HNSCC TIL based on Tim-3 expression. Gene expression profiling and NanoString RNA analysis were used to compare differences between these populations. Functional and phenotypic features of Tim-3^+^ and Tim-3^−^ Treg in freshly isolated HNSCC patient TILs were tested by multi-color flow cytometry.

**Results**

Tim-3^+^ Treg from human HNSCC TIL display a more effector-like phenotype, based on gene expression profiling and NanoString analysis. Compared with Tim-3^−^ Treg, Tim-3^+^ Treg exhibited more robust expression of CD39, CTLA-4, PD-1, granzyme B, IFN-γ receptor and IFN-γ by flow cytometry. Despite this phenotype, Tim-3^+^ TIL Treg displayed a greater capacity for inhibiting naïve CD8^+^ T cell proliferation and subsequent cytokine production than Tim-3^−^ Treg. In addition, suppression mediated by Tim-3^+^ Treg was partially reversed by exogenous IFN-g treatment.

Conclusions

Tim-3 expression on HNSCC TIL Treg cells identifies a phenotypically and functionally distinct population of cells that is highly effective in inhibiting T cell proliferation and cytokine production. Induction of IFN-g by successful immunotherapy may overcome suppressive features of this Treg population. Anti-Tim-3 directed therapies may yield immunotherapeutic benefits.

**Fig. 83 (abstract P393).**
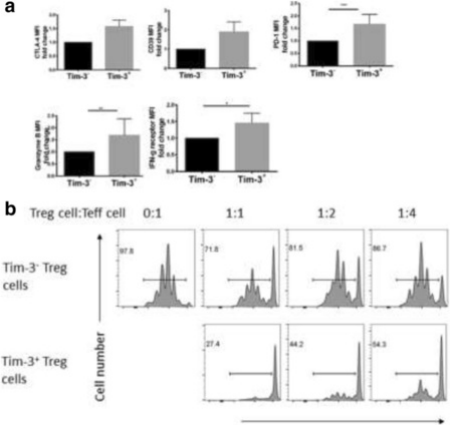
* p<0.05, ** p<0.01, *** p<0.001 Data expressed as mean ± standard deviation (SD)

### P394 PD-1+/OX40+ Tregs in head and neck squamous cell cancer suppress responder T cell responses

#### Zhuqing Liu^1^, Gulidanna Shayan^1^, Shanhong Lu^1^, Robert L Ferris^2^

##### ^1^University of Pittsburgh Medical Center, Hillman Cancer Center, Pittsburgh, PA, USA; ^2^University of Pittsburgh, Pittsburgh, PA, USA

###### **Correspondence:** Zhuqing Liu (liuz5@upmc.edu)

**Background**

FOXP3^+^ regulatory T cells (Tregs) are central for the maintenance of self-tolerance and are important suppressive components in the tumor microenvironment. The aim of this study was to investigate the phenotypic and functional characteristics of tumor infiltrating lymphocytes (TIL) with multiple immune checkpoint receptor expression, including PD-1 and OX40, directly from head and neck squamous carcinoma (HNSCC) tumors.

**Methods**

Lymphocytes were extracted from freshly excised HNSCC tumors and matched peripheral blood. Flow cytometry was conducted using HNSCC lymphocytes and *in vitro* functional assays were performed using sorted TIL or circulating T cells.

**Results**

**Fig. 84 (abstract P394).**
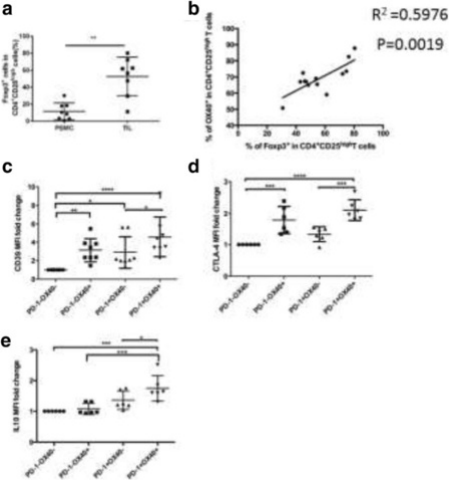
* p<0.05, ** p<0.01, *** p<0.001, **** p<0.0001 Data expressed as mean ± standard deviation (SD)

CD4^+^CD25^hi^FOXP3^+^ Tregs were more frequent in HNSCC TIL vs PBL (52.7 % vs 11.5 % of CD4^+^CD25^hi^ immune infiltrate, n = 10) (Fig. 84a). HNSCC Tregs expressed significantly higher levels of OX40 (70.8 % of FOXP3^+^ cell population, n = 10 tumors) and PD-1 (26.6 %, n = 9) than Tregs from peripheral blood (p = 0 · 01). We found a significant positive correlation (p + CD25^high^) (Fig. 84b). Double-positive (PD-1^+^OX40^+^) TIL Tregs had a more suppressive phenotype than single-positive (PD-1^+^ or OX40^+^) TIL Tregs (Fig. 84c, 84d), including the ability to proliferate and to express IL-10 and TGF-b–associated LAP (Fig. 84e). Double-positive (PD-1^+^OX40^+^) TIL Tregs also significantly inhibited effector T cells. Triggering OX40 with the an agonistic mAb (MEDI0562) overcame the suppression exerted by Tregs, leading to increased effector CD4^+^ or CD8^+^ T cell proliferation.

**Conclusions**

These findings contribute to ongoing efforts to improve PD-1 or OX40-based immunotherapy in HNSCC patients, and suggest that dual targeting of these pathways may reverse suppressive function of Tregs in the clinic.

### P395 Lymphatic vessels as biomarkers of in situ immunity in melanoma

#### Julia Femel, Takahiro Tsujikawa, Ryan Lane, Jamie Booth, Amanda W. Lund

##### Oregon Health & Science University, Portland, OR, USA

###### **Correspondence:** Amanda W Lund (lunda@ohsu.edu)

**Background**

Lymphatic vessel remodeling is correlated with melanoma progression and lymph node metastasis. While lymphatic vessels provide an important route for disseminating tumor cells, their role in responding to immunological challenge has yet to be appreciated with respect to tumor progression. We recently published that in the absence of dermal lymphatic vessels, the tumor microenvironment of murine melanoma remains completely uninflamed and fails to induce a robust T cell response. Consistently, in an analysis of human cutaneous metastatic melanoma (TCGA), we identified positive correlations between lymphatic endothelial cell gene expression and immune genes suggesting a relationship between lymphatic vessel remodeling and local immunity. We therefore hypothesize that lymphatic vessels are a biomarker of *in situ* immune responsiveness and response to therapy.

**Methods**

In this work, we present the simultaneous evaluation of immune and vascular components in human primary melanoma samples using a multiplex-immunohistochemistry-based approach. We have optimized a staining panel for simultaneous detection of 10 biomarkers (S-100, CD8a, CD68, CD31, D2-40, Lyve1, Prox1, FasL, PD-L1, PD-1) within a single FFPE section. Tissue regions that include tumor/stroma borders and show high CD8^+^ T cell infiltrates are selected for analysis, followed by tissue segmentation and automated detection of cell populations within intra-tumoral regions and stroma. We have identified a cohort of 18 human primary cutaneous melanomas with clinical annotation for protocol optimization and validation of hypotheses.

**Results**

Multiplexed immunohistochemistry of the validation cohort has identified poorly inflamed and inflamed primary melanomas. Interestingly, those tumors with enhanced hematopoietic infiltrate (CD68 and CD8) also appear to demonstrate increased vasculature, both blood and lymphatic. Preliminary data demonstrates that lymphatic vessels, blood vessels and CD8^+^ T cells are enriched in stroma, while CD68^+^ cells are homogenously distributed throughout tissue. Furthermore, blood and lymphatic endothelial cells are positively correlated in stroma as are, interestingly, intratumoral CD8^+^ T cells and stromal lymphatic vessels.

**Conclusions**

Intratumoral CD8^+^ T cells are predictive of survival. Lymphatic vessels, however, are required for transport of antigen-bearing cells to lymph nodes for induction of adaptive immunity and as such may be a prerequisite to antigen-specific T cell infiltrates and the *in situ* anti-tumor response. Our data, across multiple model systems, provides strong experimental evidence to indicate that the lymphatic vasculature is an important, active component of the anti-tumor immune response. Furthermore, lymphatic remodeling and local dysfunction may represent a biomarker to stratify patient response and survival for effective clinical immunotherapy.

### P396 CD103 and CD49a expression on melanoma-derived tumor infiltrating lymphocytes depend on location and a small fraction expresses memory T cell markers

#### Marit Melssen^1^, Anthony Rodriguez^1^, Craig L Slingluff Jr^2^, Victor H Engelhard^1^

##### ^1^University of Virginia, Charlottesville, VA, USA; ^2^Division of Surgical Oncology, University of Virginia, Charlottesville, VA, USA

###### **Correspondence:** Marit Melssen (mm2xz@virginia.edu)

**Background**

Tissue resident memory (TRM) cells are found in certain peripheral tissues long after resolution of infections that generate them. TRM formation is induced by TGFβ and has been associated with upregulation of CD103 and CD49a. Interaction of CD103 and CD49a with their respective ligands is thought to allow long term persistence of TRM cells in peripheral tissues. In melanoma patients, increased expression of CD103 and CD49a has been observed on tumor infiltrating lymphocytes (TIL) compared to PBMCs. These phenotypic similarities suggest that a subset of TIL might function as TRM. However, the impact of continuous antigen presentation and/or differential TGFβ expression levels on this property of TIL is currently unknown.

**Methods**

For *in vivo* tumor studies C57BL/6 mice were injected with 4x10^5^ B16-OVA or B16-F1-shTGFb/shNEG cells, either subcutaneous or intraperitoneal. Tumors were grown for 14 days, harvested, processed in single cell suspensions and stained for multicolor flow cytometry. Human tumors were recovered from frozen single cell suspensions, counted and stained for multicolor flow cytometry. For *in vitro* studies, bulk splenocytes (OT-1 mice) or PBMC (human normal donor) were cultured for 2–7 days in presence of CD3 and CD28 stimulating antibodies and/or 5 ng/ml recombinant TGFβ.

**Results**

Here, we demonstrate that, in murine B16-OVA and human melanoma tumors, CD8+ TIL co-express CD49a and CD103. Additionally, relative expression of CD49a and CD103 differs on TIL in subcutaneous versus intraperitoneal murine tumors, as well as between skin and small bowel human melanoma metastases. Furthermore, we identified a small subset of CD103^pos^ CD8+ TIL that express CD127 and CD69. However, the majority of CD103^pos^ CD8+ TIL express no CD127. Lastly, we show that, despite established knowledge on the induction of CD103 expression by TGFβ, secretion of this cytokine by tumor cells is not required for CD103 expression.

**Conclusions**

Our data indicate that the capacity of T cells to persist in tumors potentially changes based on tumor location, which may be dependent on TGFβ levels. Furthermore, we showed that a small subset of CD103^pos^ CD8+ TIL appear to be authentic TRM, however the majority may be either terminal effectors or inactive, potentially anergic/exhausted, TIL. Taken together, our results raise important questions about the role of CD103 on non-TRM-like TIL and the importance of TGFβ in the establishment of these TIL.

### P397 Cancer promotion and immune tolerance via cancer cell - intrinsic surface expression of GARP

#### Alessandra Metelli, Bill X Wu, Caroline W Fugle, Rachidi Saleh, Shaoli Sun, Jennifer Wu, Bei Liu, Zihai Li

##### Medical University of South Carolina, Charleston, SC, USA

###### **Correspondence:** Alessandra Metelli (metellia@musc.edu)

**Background**

The role of TGF-β in oncogenesis and immune evasion is well recognized [1]. Membrane Latent TGF-β is highly expressed on the surface of regulatory T cells and platelets by binding its receptor Glycoprotein-A Repetitions Predominant Protein (GARP). Several lines of evidence suggest that GARP enhances the conversion of the membrane Latent TGF-β in its active form [2].

**Methods**

We performed an immunohistochemistry evaluation of human breast, prostate, colon, prostate, head and neck cancer tissue microarrays and correlation with disease stage and survival. Analysis of human serum was performed by ELISA. GARP function in metastatic and non-metastatic breast cancer was evaluated by preclinical models where GARP expression was either enforced or knocked down. Therapeutic role of anti-GARP antibody was assessed using metastatic 4 T1 breast cancer model.

**Results**

We herein report that GARP is aberrantly expressed by human cancers, including colon, prostate, head and neck, and breast cancer, in addition to a well-characterized murine prostate cancer model. Importantly, expression of GARP inversely correlates with overall survival. We also identified the presence of soluble GARP in prostate cancer patients and observed that the concentration of soluble GARP is higher in cancer patients compared to healthy controls. Through genetic strategies, we found that GARP expression in multiple animal tumor models increases the bioavailability of active TGF-β, promoted regulatory T lymphocytes in tumor environment, favored the immune evasion of cancer cells, and contributed to the formation of secondary tumors. Finally, we tested a panel of anti-GARP antibodies, alone and in combination with cyclophosphamide, in a mouse model of mammary carcinoma, and we found that they have significant anti-tumor activity especially by inhibiting the formation of lung metastasis.

**Conclusions**

We conclude that GARP is expressed on several human cancers, and its expression correlates with poor prognosis. Mechanistically, GARP increases the availability of active TGF-β that promotes an immune tolerant tumor environment. Based on these observations, we propose GARP-TGF-β as a novel oncogenic axis that can be exploited for diagnosis, prognosis and treatment of cancer.

**References**

1. Derynck R, Akhurst RJ, Balmain A: **TGF-beta signaling in tumor suppression and cancer progression**. *Nat Genet* 2001, **29**:117–129.

2. Tran DQ, *et al.*: **GARP (LRRC32) is essential for the surface expression of latent TGF-beta on platelets and activated FOXP3+ regulatory T cells.** *PNAS* 2009, **106**:13445–13450.

### P398 Concomitant immune tolerance in mice with tumors at two sites exhibits reciprocal tumor specificity and requires regulatory T cells

#### Zachary S. Morris^1^, Emily I Guy^1^, Clinton Heinze^1^, Jasdeep Kler^1^, Monica M Gressett^1^, Lauryn R Werner^1^, Stephen D Gillies^2^, Alan J Korman^3^, Hans Loibner^4^, Jacquelyn A Hank^1^, Alexander L Rakhmilevich^1^, Paul M Harari^1^, Paul M Sondel^1^

##### ^1^University of Wisconsin School of Medicine and Public Health, Madison, WI, USA; ^2^Provenance Biopharmaceuticals Corp., Carlisle, MA, USA; ^3^Bristol-Myers Squibb, Princeton, NJ, USA; ^4^Apeiron Biologics, Vienna, Austria

###### **Correspondence:** Zachary S. Morris (zmorris@humonc.wisc.edu)

**Background**

We reported a cooperative interaction between local radiation (RT) and intratumoral (IT) injection of hu14.18-IL2 immunocytokine (IC, anti-GD2 antibody linked to IL2) in mice bearing a single subcutaneous tumor, resulting in 71 % complete regression [1]. However, when two tumors of the same type are present and one is treated with RT and IT-IC, the enhanced response is not seen. The non-treated tumor induces a systemic suppressive effect on the efficacy of RT and IT-IC. We hypothesize that this “concomitant immune tolerance” is a tumor-specific effect of regulatory T cells (Tregs).

**Methods**

C57BL/6 mice were implanted with one or two syngeneic B78(GD2+) melanomas or Panc02(GD2- & GD2+ subclones) pancreatic tumors. Five weeks later, mice were treated at one tumor site with single fraction (12 Gy) RT followed by IT-IC or IT-control IgG (50 μg days 6–10 after RT). C57BL/6 DEREG mice were used to test the necessity of Tregs for concomitant immune tolerance by specific depletion using intraperitoneal injection of diphtheria toxin. Tumor volumes were measured and mixed-effect-models were used to estimate and compare the tumor growth curves.

**Results**

In mice bearing single B78(GD2+) or Panc02(GD2+) tumors, combined treatment with RT and IT-IC resulted in markedly improved tumor response compared to single modality treatments. In contrast, in mice bearing two B78 tumors or two Panc02 tumors, treatment with RT and IT-IC to the GD2+ tumor did not improve tumor response compared to RT alone. This effect was tumor-specific and was not observed in mice bearing a treated B78 tumor and an untreated distant Panc02(GD2-) tumor or those bearing a treated Panc02(GD2+) tumor and an untreated distant B78(GD2+) tumor. In these mice with two histologically distinct types of tumors, response of the treated tumor to combined RT and IT-IC was similar to that observed in mice bearing a single tumor treated with RT and IT-IC. In mice bearing two B78 tumors, one treated with RT and IT-IC, depletion of Tregs (in DEREG mice or by anti-CTLA-4 IgG2a mAb in C57Bl/6 mice) facilitated a response at the treated tumor comparable to that observed with RT and IT-IC in mice bearing a single B78 tumor.

**Conclusions**

Our findings suggest that concomitant immune tolerance is a tumor-specific, Treg-dependent, suppressive effect of distant, untreated tumors on the anti-tumor efficacy of RT and IT-IC treatment of a separate tumor.

**References**

1. Morris ZS, *et al.*: **In situ tumor vaccination by combining local radiation and tumor-specific antibody**. *Cancer Res* 2016, **76**:3929–3941.

### P399 Local but not distant viral infection improves cancer outcomes: implications for cancer immunotherapy

#### Jenna Newman^1^, Andrew Zloza^2^, Erica Huelsmann^3^, Joseph Broucek^3^, Howard L Kaufman^2^

##### ^1^Rutgers University, New Brunswick, NJ, USAl; ^2^Rutgers Cancer Institute of New Jersey, New Brunswick, NJ, USA; ^3^Rush University, Chicago, IL, USA

###### **Correspondence:** Jenna Newman (jhn33@gsbs.rutgers.edu)

**Background**

Patients with cancer are at increased risk of infections; however, there is a paucity of information available regarding the effects of concomitant local or distant non-oncogenic infection on cancer and response to therapy. Further, studies interrogating the antigen-specificity of tumor-infiltrating T cells have demonstrated the existence of CD8+ T cells within the tumor that are not specific to the antigens expressed by the tumor. A portion of these CD8+ T cells are specific to antigens expressed by pathogens to which patients are commonly exposed. However, how such cells effect tumor growth is not well understood.

**Methods**

Therefore, we challenged C57BL/6 mice with B16-F10 (10^5^) tumor cells via intradermal injection of the right flank (for tumor growth distal to the infection) or via intravenous injection (for tumor growth local [within the lung] to the infection). B6 mice were infected with influenza (A/H1N1/PR8) expressing OVA_257–264_ (FLU-OVA; 10,000 pfu; intranasal administration). Every 2–3 days tumor growth was monitored by caliper measurement and influenza infection was monitored by body weight measurement. Tumors were harvested at various times and analyzed by flow cytometry for OVA-specific CD8+ T cells (representing influenza-specific cells) using tetramer staining. Statistics were determined using the Student’s t test and comparisons with P < 0.05 were considered statistically significant.

**Results**

Infection of the lung with FLU-OVA expectedly lead to the expansion of CD8+ T cells specific for OVA_257–264_ in the lung. Unexpectedly, such infection likewise led to the distal accumulation of OVA_257–264_-specific T cells (up to 23 % of all CD8+ T cells; P < 0.01) in the tumor microenvironment (skin of the flank) and accelerated of tumor growth (P < 0.001). Importantly, in the context of B16 melanoma in the lung, infection of the lung with FLU-OVA resulted in reduced B16 melanoma foci compared to no infection (38 % reduction; P < 0.01). Melanoma foci were further reduced (81 %) in the lung with combination FLU-OVA and PD-1 blocking anybody (P < 0.01).

**Conclusions**

Infection local to the tumor site (lung infection in the context of lung melanoma) but not distal to the site (lung infection in the context of skin melanoma) improves cancer outcome. This finding may have implications for viral infections used as immunotherapeutic strategies (including oncolytic viruses), which may demonstrate maximum efficacy when administered local to the tumor and when offsite distant infection is minimized. Further studies utilizing the engagement of pathogen-specific CD8+ T cells in the tumor microenvironment as a novel immunotherapeutic approach are currently underway.

### P400 "Enriched-in-renal-carcinoma dendritic cells” (ercDCs) are unique macrophages with a gene signature indicative of immune escape

#### Dorothee Brech^1^, Tobias Straub^2^, Martin Irmler^3^, Johannes Beckers^3^, Florian Buettner^4^, Elke Schaeffeler^4^, Matthias Schwab^5^, Elfriede Noessner^6^

##### ^1^Helmholtz Zentrum München, Immunoanalytics- Tissue Control of Immunocytes, Munich, Bayern, Germany; ^2^Bioinformatics Core Unit, Biomedical Center, Ludwig-Maximilians-University, Planegg-Martinsried Munich, Bayern, Germany; ^3^Helmholtz Zentrum München, Institute of Experimental Genetics, Neuherberg, Bayern, Germany; ^4^Dr. Margarete Fischer-Bosch-Institute of Clinical Pharmacology, Stuttgart, Baden-Wurttemberg, Germany; ^5^Dr. Margarete Fischer-Bosch-Institute of Clinical Pharmacology, Stuttgart, University of Tuebingen; Department of Clinical Pharmacology, University Hospital Tuebingen,, Stuttgart, Baden-Wurttemberg, Germany; ^6^Helmholtz Zentrum München, Immunoanalytics-Tissue Control of Immunocytes, Munich, Bayern, Germany

###### **Correspondence:** Elfriede Noessner (noessner@helmholtz-muenchen.de)

**Background**

Clear cell renal cell carcinoma (ccRCC) is considered immuno-sensitive and tumors typically show a strong immune cell infiltrate. Yet, tumor control is not achieved. Previously, we have described that infiltrating CD8^+^ T effector cells and NK cells are unresponsive to stimulation with a signature of anergy and cell cycle arrest [1, 2]. These features were caused by the tumor microenvironment as they were not observed in cells from non-tumor kidney (NKC). Many T cells in the tumor tissue were in close contact with CD209^+^ myeloid cells, which is suggestive of intense intercellular communication. Further analysis revealed unusual co-expression of DC and macrophage markers. Those unusual cells were highly enriched in tumor tissue compared to non-tumor kidney cortex (NKC), thus they were referred to as “enriched-in-renal-carcinoma DCs” (ercDCs) [3]. For a deeper understanding of the role which this unusual myeloid subset might play and to potentially identify targets for therapeutic intervention we conducted genome-wide transcriptional profiling of sorted ercDCs.

**Methods**

*In situ* analysis using triple-marker immunofluorescence with confocal microcopy; *ex vivo* analysis using polychromatic flow cytometry; cell sorting from ccRCC tissue suspensions and blood; *in vitro* generation of reference myeloid subsets; transcriptional profiling using Affymetrix microarrays; bioinformatics using data from sorted cells and publicly available repositories, including The Cancer Genome Atlas (TCGA; http://cancergenome.nih.gov/).

**Results**

ErcDCs were found to strongly express macrophage core genes, macrophage-associated transcription factors and growth factor receptors. They combined characteristics of M1- and M2-macrophages and exhibited a pro-angiogenic and invasive gene expression profile. A unique transcriptional signature could be identified that distinguished ercDCs from all other analyzed myeloid subsets except inflammatory macrophages from ascites of ovarian cancer. Applying the gene signature to TCGA ccRCC expression data (RNA Seq) revealed a significant association between a high ercDC score and advanced tumor grade and shorter patient survival. The gene expression profile contains targets with promise for repolarization of ercDCs into an immunocompetent cell type.

**Conclusions**

The transcriptional profile of ercDCs identified them as a unique myeloid subset within the macrophage spectrum. A high ercDC score is associated with poor patient survival. The gene expression profile contains promising targets for intervention.

**Acknowledgements**

We appreciate the cooperation with clinicians and pathologists, and express our gratitude to all patients for their tissue donation.

**References**

1. Prinz P, *et al.*: *J Immunol* 2012, **188(12)**:5990–6000.

2. Prinz P, *et al.*: *Int J Cancer* 2014, **135(8)**:1832–1841.

3. Figel A-M, Brech D, *et al.*: *Am J Pathol* 2011, **179(1)**:436–451.

### P401 Nano-Pulse Electro-Signaling treatment of murine tumors significantly reduces the percentage of regulatory T cells in the treated tumor

#### Snjezana Anand, Amanda McDaniel, John Cha, Darrin Uecker, Richard Nuccitelli

##### Pulse Biosciences, Burlingame, CA, USA

###### **Correspondence:** Richard Nuccitelli (rnuccitelli@pulsebiosciences.com)

**Background**

Nano-Pulse Electro-Signaling (NPES) refers to the application of ultrashort electric pulses in the nanosecond range and can be used to initiate a variety of cellular responses. This physical treatment is drug-free, non-thermal and highly localized between electrodes. We have previously found that murine cells and tumors exposed to the appropriate NPES can be stimulated to undergo immunogenic apoptosis. When these mice are injected with a challenge secondary tumor they reject that tumor in a CD8-dependent manner. In addition, we can vaccinate a mouse against specific tumors with a sub-dermal injection of NPES-treated tumor cells [1]. We are using flow cytometry to examine changes in the T cell population in the primary and challenge tumor microenvironment before and after NPES treatment.

**Methods**

Murine MCA205 fibrosarcoma cells were injected subdermally into syngeneic B6 mice to form a tumor. Once the tumor reached a diameter of 5 mm, we treated it *in vivo* with NPES (1300 pulses, 300 ns, 30 kV/cm). Some of these tumors were removed surgically and dissociated into single cells for analysis by flow cytometry. Others were allowed to undergo apoptosis and three weeks later a challenge tumor was injected into the contralateral flank of the mouse. 2 weeks later, those mice were sacrificed and their spleens and lymph nodes were removed and dissociated for flow cytometry analysis.

**Results**

**Fig. 85 (abstract P401).**
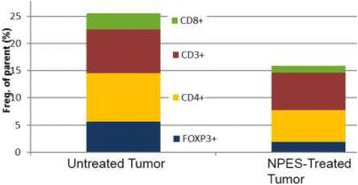
T cell population in NPES-treated and untreated tumors. There is a 50% reduction in Tregs in tumors treated with NPES

Tumor treatment by NPES results in a significant reduction in both Tregs and CD8+ T cells within the tumor (Fig. 85). In contrast, the spleen and lymph nodes of the treated mice had slightly higher levels of Treg and CD8+ T cells than those with untreated tumors. Ongoing flow cytometry studies of the changes in the secondary challenge tumors will be also be presented.

**Conclusions**

We conclude that NPES treatment reduces the number of regulatory T cells in the treated primary tumor and are currently examining the changes in the T cell population in challenge tumors.

**References**

1. Nuccitelli R, Berridge JC, Mallon Z, Kreis M, Athos B, Nuccitelli P: **Nanoelectroablation of Murine Tumors Triggers a CD8-Dependent Inhibition of Secondary Tumor Growth**. *PLoS One* 2015, **10(7):**e0134364.

### P402 Targeting colony stimulating factor-1 receptor (CSF-1R) with SNDX-6352, a novel anti-CSF-1R targeted antibody

#### Peter Ordentlich^1^, Alison Wolfreys^2^, Andre Da Costa^3^, John Silva^2^, Andrea Crosby^2^, Ludovicus Staelens^3^, Graham Craggs^2^, Annick Cauvin^3^, Sean Mason^2^

##### ^1^Syndax Pharmaceuticals, Inc., Waltham, MA, USA; ^2^UCB Biopharma, Slough, England, UK; ^3^UCB Biopharma, Braine-l'Alleud, Brabant Wallon, Belgium

###### **Correspondence:** Peter Ordentlich (pordentlich@syndax.com)

**Background**

CSF-1R is expressed on immunosuppressive tumor associated macrophages (TAMs) that accumulate within the tumor microenvironment. High levels of TAMs correlate with poor prognosis for certain cancers and inhibition of TAMs can enhance anti-tumor immune responses. SNDX-6352 is a humanized IgG4 monoclonal antibody with high affinity against CSF-1R under investigation for the treatment of neoplastic diseases.

**Methods**

*In vitro* binding studies, functional cell based assays, and mouse tumor models were utilized to characterize SNDX-6352 and a surrogate rodent antibody, Ab535. Reported toxicology and pharmacodynamics studies for SNDX-6352 were in cynomolgus monkeys.

**Results**

SNDX-6352 binds to human CSF-1R (KD 4-8pM, Fc-tagged construct) and cross reacts with cynomolgus monkey CSF-1R but not rodent CSF-1R. SNDX-6352 has been shown to inhibit both CSF-1 (IC_50_ 270pM) and IL-34 (IC_50_ 100pM) induced MCP-1 release from human monocytes) and the viability of macrophages during tCSF-1-mediated differentiation process *in vitro* (IC_50_ 455pM). A dual ligand blocking surrogate rodent antibody, Ab535, was generated for preclinical studies in mice and shown to inhibit tumor growth in colon (MC38), breast (MCF-7, MDA-MB-231) and prostate (PC-3) cancer models and enhance immune checkpoint blockade in the MC38 model. A 13-week toxicology study in cynomolgus monkeys indicated that weekly intravenous administration of SNDX-6352 at 10, 30, and 100 mg/kg was well tolerated. Swelling around the eyes was observed at the mid and high dose groups, occurring from Day 85 onward and may be related to CSF-1R pharmacology, as it has been observed with other anti-CSF-1R molecules, including in clinical trials at supra-therapeutic doses. Other changes were also all related to the mode of action of SNDX-6352. Clinical pathology changes included dose-related increases in aspartate transferase, alanine transferase and glutamate dehydrogenase levels correlating with SNDX-6352 exposure but not translating into histopathological liver changes. These changes resulted from pharmacological inhibition of CSF-1R on Kupffer cells within the liver, which are involved in the clearance of these hepatic enzymes and as such, considered to be non-adverse. Dose-related increases in CSF-1 plasma concentration and decreases in non-classical (CD14 + CD16+) monocytes and markers of bone formation and resorption were observed during the treatment phase and were rapidly reversible upon SNDX-6352 clearance. Changes in bone markers were not associated with histological changes or changes in bone densitometry in the context of this study. Upon SNDX-6352 clearance, all parameters rapidly returned to control values.

**Conclusions**

SNDX-6352 is a novel, high affinity humanized IgG4 anti-CSF-1R antibody available for initiation of first-in-human studies.

### P403 Anti-CD47 monoclonal antibody SRF231 is a potent inducer of macrophage-mediated tumor cell phagocytosis and has anti-tumor activity in preclinical models

#### Alison M. Paterson, Andrew C Lake, Caroline M Armet, Rachel W O'Connor, Jonathan A Hill, Emmanuel Normant, Ammar Adam, Detlev M Biniszkiewicz, Scott C Chappel, Vito J Palombella, Pamela M Holland

##### Surface Oncology, Cambridge, MA, USA

###### **Correspondence:** Alison M. Paterson (apaterson@surfaceoncology.com)

**Background**

CD47 has been identified as a tumor antigen on human ovarian cancer cells and is expressed on multiple tumor types. CD47 negatively regulates phagocytosis by interacting with signal regulatory protein alpha (SIRPα), an inhibitory protein expressed on macrophages. High expression of CD47 on tumors contributes to tumor immune evasion by blocking phagocytic uptake. Agents that block the CD47-SIRPα interaction are promising therapeutics in that they restore phagocytic uptake of CD47^+^ tumor cells *in vitro* and attenuate tumor growth *in vivo*. Here we characterize SRF231, one of a panel of fully human CD47 antibodies, and demonstrate that it displays the desired criteria for development as a therapeutic.

**Methods**

Anti-CD47 antibodies were generated using transgenic mice carrying human V regions and SRF231 was subsequently engineered as several Fc-region variants. *In vitro* phagocytosis assays were performed using primary human macrophages differentiated from peripheral blood-derived monocytes. Phagocytosis was measured by flow cytometric analysis of CFSE-labeled target cells after incubation with CD14^+^ macrophages. For *in vivo* studies, SCID mice were injected subcutaneously with tumor cells and treated with the indicated antibodies.

**Results**

SRF231 binds with high affinity to human CD47 and is a potent blocker of the CD47-SIRPα interaction. SRF231 enhances phagocytic uptake of primary tumor cells and tumor cell lines *in vitro*, and does so irrespective of macrophage polarization. SRF231-mediated tumor cell phagocytosis is also enhanced in the presence of opsonizing antibodies. Importantly, since loss of CD47 expression serves as a marker of aged/damaged red blood cells (RBC) and promotes their phagocytic clearance, SRF231 does not enhance phagocytosis or induce hemagglutination of human or cynomolgus RBC, despite binding to these cells. To better understand the relative contribution of Fc receptor (FcR) interactions on SRF231 mediated activity, we generated and characterized SRF231 isotypes both with and without effector function. Potency in the phagocytosis assay is mildly decreased in isotypes with lower or no ability to bind FcR. In a tumor xenograft model, antibodies display isotype-specific differences in anti-tumor activity despite having equivalent systemic exposures.

**Conclusions**

SRF231 binds with high affinity to CD47 and potently disrupts the CD47-SIRPα interaction. SRF231 is unique in its ability to promote robust tumor cell phagocytosis without inducing RBC phagocytosis or hemagglutination. In addition, SRF231 shows potent *in vivo* anti-tumor efficacy in preclinical models either as monotherapy or in combination settings. SRF231 is currently in IND-enabling studies and is expected to enter clinical trials in 2017.

### P404 Selective small molecule inhibitors of CD73 promote human CD8+ T cell activation and positively impact tumor growth and immune parameters in experimental tumor models

#### Jay P. Powers, Annette Becker, Ada Chen, Manmohan R Leleti, Eric Newcomb, Holly Sexton, Ulrike Schindler, Joanne BL Tan, Steve W Young, Juan C Jaen

##### Arcus Biosciences, Hayward, CA, USA

###### **Correspondence:** Jay P. Powers (jpowers@arcusbio.com)

**Background**

In the tumor micro-environment extracellular ATP is sequentially hydrolyzed to adenosine by the ecto-nucleotidases CD39 (ATP → AMP) and CD73 (AMP → adenosine). Adenosine is a potent inhibitor of T cell activation, resulting in an immunosuppressed phenotype, and thus inhibition of CD73 has recently generated much interest in immuno-oncology. We present here novel, selective, and highly potent small molecule inhibitors of CD73.

**Methods**

CD73 inhibitor *in vitro* potency was assessed by measuring the hydrolysis of AMP to adenosine in the presence of varying amounts of inhibitors in endogenously expressing (hCD73/SKOV-3; mCD73/E771) and stably over-expressing (hCD73/CHO; hCD39/CHO) cells. Selectivity was assessed in transiently expressing CHO cells (mCD73, NTPDase2, NTPDase3, and NTPDase8). Activity in a CD8+ T cell functional assay was assessed in human and mouse CD8+ T cells which were pretreated with inhibitor, buffer control, or CD73-inactive control followed by stimulation (3 days; CD3/CD28 ± 50 mM AMP). Cell activation, proliferation, and effector function were quantified by flow cytometry. CD73 inhibition in a tumor setting was assessed in a subcutaneous CT26 tumor model in Balb/c mice. Treatment was initiated when tumor volumes reached ~100 mm^3^ and mice were divided into four cohorts (vehicle control + isotype control, CD73 inhibitor A000830 + isotype control, vehicle control + anti-PD-1 Ab, and A000830 + anti-PD-1 Ab.

**Results**

A000830 and A001190 are representative members of a series of potent and selective inhibitors of CD73 (hCD73/CHO IC_50_ = 1 nM and 0.03 nM respectively). A000830 and A001190 potently blocked the conversion of AMP to adenosine in human CD8+ T cells (IC_50_ = 0.2 and 0.008 nM respectively) and robustly reversed adenosine-mediated inhibition of proliferation, CD25 expression, and IFN-g and granzyme B production in human CD8+ T cells. The compounds are highly selective (>10,000-fold) against related ecto-nucleotidases (CD39, NTPDases2/3/8) as well as a large panel of unrelated targets. *In vivo*, A000830 was well tolerated in mice at plasma concentrations sustained well over IC_90_ (21 days). Therapeutic dosing of A000830 in mice in combination with an anti-PD-1 antibody resulted in robust inhibition of CT26 tumor growth, greater than either treatment alone.

**Conclusions**

We have designed a series of potent and selective small molecule inhibitors of mouse and human CD73 exemplified by A000830 and A001190. These inhibitors block the generation of adenosine from AMP, reverse adenosine-mediated inhibition of human and mouse T cell activation, and demonstrate promising anti-tumor activity when dosed in combination with PD-1 blockade.

### P405 Observation of immunobiology landscape in mucosal melanoma

#### Suthee Rapisuwon^1^, Arash Radfar^2^, Kellie Gardner^3^, Geoffrey Gibney^3^, Michael Atkins^3^

##### ^1^Georgetown Lombardi Comprehensive Cancer Center, Washington, DC, USA; ^2^Washington Hospital Center, Washington, DC, USA; ^3^Lombardi Comprehensive Cancer Center, Washington, DC, USA

###### **Correspondence:** Suthee Rapisuwon (sr905@georgetown.edu)

**Background**

Both increased number of pre-treatment CD8+ T lymphocytes and PD-L1 expression at the invasive tumor margin of advanced cutaneous melanoma metastases predicted improved treatment outcome in patients receiving single agent therapy with the PD-1 inhibitor, pembrolizumab [1, 2]. Association between CD8+ T cell numbers or PD-L1 expression and response to combined immune-checkpoint blockade in mucosal melanoma has not been established.

**Methods**

Four cases of advanced mucosal melanoma were each stained with antibodies against CD8, and PD-L1 (SP-142; rabbit monoclonal antibody, Spring Bioscience). A pan-melanoma (HMB-45, MART-1, and tyrosinase), mouse monoclonal antibody (Biocare) was used to identify the tumor area and margins. The pathologist was blinded to the diagnosis and clinical information. Intensity of immunostaining was measured with average optical density (OD). CD8+ T cells and PD-L1 cells density were measured using ImageJ with semi-automated Nuclei Segmentation-IHC Tool Box plugin developed by Shu, et al. [3].

**Results**

**Fig. 86 (abstract P405).**
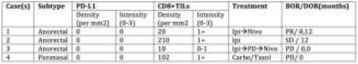
Discrepancy of CD8+TILs in mucosal melanoma (BOR: Best Overall Response, DOR: Duration of Response)

The breakdown of PD-L1 and CD8 immunohistochemistry (IHC) from three anorectal melanoma and one paranasal melanoma are described in Fig. 86. Tumor PD-L1 staining was negative in all tumors measured. CD8+ T cells are non-brisk in all tumors measured. There is a discrepancy in density of total CD8+ T cells. CD8+ T cells at the invasive margin are scarce.

**Conclusions**

This preliminary data is unable to demonstrate any definitive pattern of CD8+ T cells or PD-L1 expression in a small case series of mucosal melanoma. To further address the immunobiology of mucosal melanoma and its microenvironment, the Melanoma Research Foundation Breakthrough Consortium, is conducting, “A Study to Estimate the Anti-tumor Activity and Identify Potential Predictors of Response in Patients with Advanced Mucosal or Acral Lentiginous Melanoma Receiving Standard Nivolumab in Combination with Ipilimumab Followed by Nivolumab Monotherapy.” The study will assess whether pre-existing immune cell infiltrates and PD-L1-expressing cells at the invasive tumor margin correlate with clinical response to combination checkpoint blockade in these uncommon melanoma subtypes.

**References**

1. Messina JL, Fenstermacher DA, Eschrich S, Qu X, Berglund AE, Lloyd MC, *et al.*: **12-Chemokine gene signature identifies lymph node-like structures in melanoma: potential for patient selection for immunotherapy?** *Sci Rep* 2012, **2**:765.

2. Tumeh PC, Harview CL, Yearley JH, Shintaku IP, Taylor EJ, Robert L, *et al.*: **PD-1 blockade induces responses by inhibiting adaptive immune resistance**. *Nature* 2014, **515(7528)**:568–571.

3. Shu J, Fu H, Qiu G, Kaye P, Ilyas M: **Segmenting overlapping cell nuclei in digital histopathology images**. *Conf Proc IEEE Eng Med Biol Soc* 2013, **2013**:5445–5448.

### P406 Leukocyte chemoattractant chemerin upregulates PTEN activity in human tumors via CMKLR1

#### Keith R. Rennier, Robert Crowder, Ping Wang, Russell K Pachynski

##### Washington University School of Medicine in St. Louis, St. Louis, MO, USA

###### **Correspondence:** Keith R. Rennier (keith.rennier@wustl.edu)

**Background**

The balance between anti-tumor effector and immunosuppressive immune cells in the tumor microenvironment (TME) is a key determinant of response to cancer treatment. Phosphatase and tensin homolog (PTEN) modulation can directly affect T cell mediated immunotherapies. Specifically, the loss of PTEN has been shown to promote resistance to this type of immunotherapy, supporting the significance of this oncogenic pathway in immunotherapy responses and suggesting upregulation of PTEN activity may have a favorable impact. Chemerin (*RARRES2*; retinoic acid receptor responder 2) is a recently identified endogenous leukocyte chemoattractant shown to recruit innate immune cells. Previous studies in mouse tumor models suggest that chemerin is a tumor suppressive chemoattractant cytokine, capable of recruiting immune effector cells into the TME. *RARRES2* is commonly downregulated across multiple tumor types compared to normal tissue counterparts in microarray studies. Several methylome-wide studies in various tumor types have identified *RARRES2* as one of the most hypermethylated genes, potentially leading to decreased chemerin expression. Therefore, we hypothesized that augmentation of chemerin in the TME might inhibit tumor progression and activity.

**Methods**

To test this, we exposed human cancer cell lines to exogenous chemerin *in vitro*.

**Results**

Surprisingly, we found recombinant chemerin was able to upregulate PTEN expression, a key cell survival and proliferation checkpoint. Specifically, mRNA and protein analyses show a significant upregulation of PTEN after 48 hour chemerin exposure, without significant changes in tumor cell proliferation or apoptosis. Additionally, we found that treatment with chemerin was also able to significantly decrease tumor cell migration. Using siRNA, we found that knockdown of CMKLR1 – the main chemerin receptor that mediates leukocyte chemotaxis – abrogated chemerin-induced upregulation of PTEN, suggesting chemerin’s effect on PTEN expression in tumor cells is mediated through this G-protein coupled receptor. These studies help delineate the role of chemerin/*RARRES2* suppression within the tumor microenvironment, and elucidate a novel chemerin-PTEN pathway that may have implications on tumor progression and migration. Importantly, these effects of chemerin are independent of its ability to recruit leukocytes into the TME, suggesting chemerin has multiple mechanisms of anti-tumor action.

**Conclusions**

Overall, our studies show a novel finding that chemerin augments PTEN activity via CMKLR1. Thus, chemerin-driven increased PTEN expression and activity may help facilitate improved immunotherapy, not only by recruiting immune effector cells into, but also by augmenting PTEN expression within the TME. Future experimental studies will focus on the chemerin-driven upregulation of PTEN activity and its effects on immunotherapy strategies.

### P407 Regulation of IL-15 in the tumor microenvironment by the STING pathway

#### Rosa M. Santana Carrero^1^, Sarai Rivas^2^, Figen Beceren-Braun^2^, Scott Anthony^3^, Kimberly S Schluns^2^

##### ^1^The University of Texas Graduate School of Biomedical Sciences at Houston, Houston, TX, USA; ^2^Department of Immunology, University of Texas MD Anderson Cancer Center, Houston, TX, USA; ^3^Department of Microbiology, University of Iowa, Iowa City, IA

###### **Correspondence:** Rosa M. Santana Carrero (rosa.santanacarrero@uth.tmc.edu)

**Background**

Expression of IL-15 within the tumor correlates with increased tumor infiltrating T cells, decreased metastasis and increased patient survival [1]. Unfortunately, the regulation of IL-15 within the tumor microenvironment is not clear. In our recent study we showed that agonists of the Stimulator of Interferon Genes (STING) pathway are potent inducers of soluble IL-15 complexes (sIL-15c) *in vivo* [2]. Since the STING pathway is important for regulation of type I interferons (IFNs) in tumors, we set out to determine if sIL-15c are produced in the tumor microenvironment and are regulated by STING signaling.

**Methods**

C57BL/6, IL-15Ra−/−, IFNAR1−/− and Tmem173−/− mice were challenged with 300,000 B16-F10 melanoma cells by s.c. injection. Tumors of various sizes were isolated and sIL-15c were measured in homogenates from tumors and spleens using ELISAs. For stimulation of STING pathway, mice were administered c-di-GMP (25ug/mouse) via intratumoral injection every three days. To measure proliferation, OT-I CD8+ T cells were CFSE labelled and adoptively transferred into wild-type and IL-15Ra−/− mice. Tumors, spleens and tumor-draining lymph nodes were processed and stained for analysis via flow cytometry.

**Results**

Levels of sIL-15c were high in homogenates from small tumors and low in larger tumors, but were absent in homogenates from IL-15Ra −/− mice indicating sIL-15c were produced by the tumor stroma. Tumors isolated from IFNAR1−/− mice contained decreased levels of sIL-15c, but interestingly, those isolated from mice with a defective STING pathway had normal levels of sIL-15c suggesting that type I IFN-dependent, but STING-independent pathway was required for intratumoral IL-15 regulation. Nonetheless, intratumoral delivery of STING agonists enhanced production of sIL-15c in B16 tumors. Whereas treatment of tumors with STING agonists promoted tumor regression and enhanced proliferation of tumor-specific CD8+ T cells, these effects were severely blunted in IL-15Ra−/− mice.

**Conclusions**

These findings indicate that IL-15 is a critical factor mediating the anti-tumor responses induced by STING signaling.

**References**

1. Mlecnik B, Bindea G, Angell HK, Sasso MS, Obenauf AC, Fredriksen T, *et al.*: **Functional network pipeline reveals genetic determinants associated with in situ lymphocyte proliferation and survival of cancer patients.** *Sci Transl Med* 2014, **6**:228ra37.

2. Anthony SM, Howard ME, Hailemichael Y, Overwijk WW, Schluns KS: **Soluble interleukin-15 complexes are generated in vivo by type i interferon dependent and independent pathways.** *PLoS One* 2015, **10**:e0120274.

### P408 Regulatory T cell derived inhibitory cytokines IL35 and IL10 promote T cell dysfunction in the tumor microenvironment

#### Deepali Sawant, Maria Chikina, Hiroshi Yano, Creg Workman, Dario Vignali

##### University of Pittsburgh, Pittsburgh, PA, USA

###### **Correspondence:** Deepali Sawant (sawant.deepali@gmail.com)

**Background**

Regulatory T cells (T_regs_) are a specialized suppressive CD4^+^ T cell sub-population capable of restricting deleterious immune responses to self and foreign antigens that underlie autoimmune and chronic inflammation. Conversely, T_regs_ block beneficial anti-tumor immune responses and thereby pose severe impediment to effective tumor immunotherapies. Although T_reg_ depletion reduces tumor burden, the ensuing autoimmune sequelae limits its utility in the clinic. Thus targeted approaches limiting T_reg_ cell activity specific to the tumor microenvironment are warranted. One of the major mechanism by which T_regs_ exert their suppressive effects is via secretion of inhibitory cytokines. However, the physiological relevance of this mode of suppression to the tumor microenvironment remains largely unclear.

**Methods**

To interrogate the role of the two predominant T_reg_ suppressive cytokines, IL35 and IL10, in the tumor setting, we injected B16F10 tumors intradermally into *Foxp3* ^Cre-YFP^ mice bearing reporters for IL35 and IL10 (*Foxp3* ^CreYFP^.*Ebi3* ^tdTom^ *.IL10* ^GFP^). This enabled tracking of T_regs_ expressing/co-expressing IL35 and IL10 in the tumor microenvironment. We also performed tumor experiments in mice bearing IL35-deficient T_regs_ (*Foxp3* ^Cre-YFP^ *.Ebi3* ^L/L^) and IL10-deficient T_regs_ (*Foxp3* ^Cre-YFP^ *.IL10* ^L/L^) to assess the impact of T_reg_-specific deletion of the two suppressive cytokines on tumor growth and CD8^+^ T cell effector function.

**Results**

Preliminary studies using the dual cytokine reporter mice reveal distinct populations of T_regs_ expressing IL35 and IL10 in the B16 tumor microenvironment, with a preferential enrichment of IL35^+^ T_regs_ and little to no co-expression of the two cytokines on T_regs_. Reduced tumor burden was noted in both *Foxp3* ^Cre-YFP^ *.Ebi3* ^L/L^ and *Foxp3* ^Cre-YFP^ *.IL10* ^L/L^ mice. Interestingly, this was associated with a significant loss of multiple inhibitory receptors (IRs) on CD8^+^ T cells in both floxed environments. These findings suggest a potential role for the T_reg_-derived inhibitory cytokines in inducing multi-IR^+^ exhausted state on CD8^+^ T cells in tumors.

**Conclusions**

The dramatic loss of IR^+^ T cells in mice bearing IL35 and IL10-deficient T_regs_ highlights the possibility of targeting these inhibitory cytokines to limit T cell exhaustion in tumors. Our study uncovers a novel mechanism of T_reg_ suppression within the tumor microenvironment wherein T_reg_-derived inhibitory cytokines induce a state of dysfunction on the effector T cells thereby contributing to tumor immune evasion.

### P409 Human melanomas and ovarian cancers overexpressing mechanical barrier molecule genes lack immune signatures and have increased patient mortality risk

#### Elise Salerno^1^, Davide Bedognetti^2^, Ileana Mauldin^3^, Donna Deacon^3^, Sofia Shea^4^, Joel Pinczewski^5^, Joseph M Obeid^6^, George Coukos^7^, Ena Wang^2^, Thomas Gajewski^8^, Franco M Marincola^2^, Craig L Slingluff Jr^3^

##### ^1^University of Alabama, Birmingham, AL, USA; ^2^Sidra Medical and Research Center, Doha, Ad Dawhah, Qatar; ^3^University of Virginia, Charlottesville, VA, USA; ^4^Hunter Holmes McGuire Veterans Administration Medical Center, Richmond, VA, USA; ^5^Dorevitch Pathology, Heidelberg West, Victoria, Australia; ^6^Department of Surgery, University of Virginia, Charlottesville, VA, USA; ^7^Ludwig Institute for Cancer Research, Epalinges, Vaud, Switzerland; ^8^The University of Chicago, Chicago, IL, USA

###### **Correspondence:** Craig L. Slingluff Jr (cls8h@virginia.edu)

**Background**

Immune signatures of T cell infiltration for melanoma and ovarian cancer are associated with improved survival. Mechanisms enabling and regulating T cell infiltration in the tumor microenvironment are being elucidated. We hypothesized that genes overexpressed in tumors without T cell infiltration may limit T cell infiltration and have prognostic implications.

**Methods**

Gene expression profiling of 44 human melanoma metastases had previously identified subsets with or without Th1 immune signatures. We identified genes overexpressed five-fold or more in tumors lacking immune signatures, at a significance level of p < 0.01. These findings were further validated in gene expression data from melanoma metastases and ovarian cancers. To assess associations with survival, RNAseq data and patient survival data from The Cancer Genome Atlas (TCGA) were assessed with Kaplan-Meier curves, and log-rank tests.

**Results**

We identified 8 genes overexpressed in human melanomas and ovarian cancers lacking Th1 immune signatures, and which encode molecules with mechanical barrier function in normal tissues: filaggrin (FLG), tumor-associated calcium signal transducer 2 (TACSTD2), and 6 desmosomal proteins (DST, DSC3, DSP, PPL, PKP3, and JUP). This association was validated in another 114 melanoma metastases, where DST expression alone was sufficient to identify melanomas without immune signatures, while FLG and the other 6 putative barrier molecules were overexpressed in a different subset of melanomas lacking immune signatures. Similar associations were identified in a set of 186 ovarian cancers. RNA-seq data from 471 melanomas and 307 ovarian cancers in the TCGA further supported these findings and also revealed that overexpression of barrier molecules was largely independent of β-catenin/WNT and endothelin receptor B gene expression. Overexpression of barrier molecules was associated with early patient mortality for melanoma (p = 0.0002) and ovarian cancer (p < 0.01). This was true for FLG for melanoma (p = 0.012) and ovarian cancer (p = 0.006), whereas DST overexpression was negatively associated with CD8 gene expression, but not with patient survival.

**Conclusions**

Overexpression of FLG or DST identifies 2 distinct patient populations with low immune cell infiltration in melanoma and ovarian cancers, but with different prognostic implications for each. These data raise the possibility that molecules with mechanical barrier function in skin and other tissues may be used by cancer cells to protect them from immune cell infiltration and immune-mediated destruction.

**Acknowledgements**

Gene expression data from melanoma metastases were provided by Steven A Rosenberg (NCI). Some results here are based upon data generated by the TCGA Research Network: http://cancergenome.nih.gov (Cerami, *et al*., PMID 22588877; Gao J, *et al*., PMID 23550210).

### P410 Direct visualization of tumor immune evasion against CD8+ effector T cells using intravital microscopy

#### Stefani Spranger^1^, Brendan Horton^1^, Thomas F Gajewski^2^

##### ^1^Department of Pathology, The University of Chicago, Chicago, IL, USA; ^2^University of Chicago Medical Center, Chicago, IL, USA

###### **Correspondence:** Stefani Spranger (sspranger@bsd.uchicago.edu)

**Background**

CD8^+^ T cell infiltration into the tumor microenvironment appears to be correlative for clinical response to checkpoint blockade immunotherapy. Understanding the molecular mechanisms which cause lack of T cell infiltration will be instrumental for the development of novel treatment combinations to expand clinical efficacy. Recent studies have demonstrated that tumor cell-intrinsic signaling pathways, including the Wnt/β-catenin pathway, result in a lack of T cell infiltration in patients and genetically engineered mouse (GEM) models. Mechanistically, activation of the Wnt/β-catenin signaling pathway in the primary tumor mediates inhibition of priming of tumor-antigen-specific T cells through failed recruitment of Batf3-lineage DCs. However, it was not clear whether activation of a tumor cell-intrinsic signaling pathway could also mediate secondary resistance in the presence of a systemic anti-tumor immune response. In addition, it was not known whether adoptive T cell transfer would be a potential therapeutic option for non-T cell inflamed tumors.

**Methods**

To address those questions we used GEMs for murine melanoma with or without activation of the Wnt/β-catenin signaling pathway and engineered to expresses the model antigen SIY (SIYRYYGL).

**Results**

By using these GEMs we now show that tumor cell-intrinsic activation of β-catenin was capable to mediate secondary resistance by excluding effector T cell from the tumor microenvironment. Effector T cell infiltration in β-catenin negative tumors was further associated with the emergence of immuno-edited tumor escape variants, which were never observed in β-catenin positive non-T cell inflamed tumors. Consistently, adoptive T cell transfer of antigen-specific T cells failed to provide therapeutic benefit in tumors with activated β-catenin signaling. Using intravital-microscopy we found that, while effector T cells in β-catenin negative tumors made direct tumor cell contact and showed a dynamic and directed migratory behavior, the few effector T cells found in β-catenin positive tumor failed to engage close contact with tumor cells and were immobile within areas having the appearance of stroma. Mechanistically, we identified a lack of expression of the chemokines CXCL9 and CXCL10 in tumor with activated b-catenin signaling, which a are normally produced by CD103^+^ Batf3-driven DCs.

**Conclusions**

Our data support the notion that CD103^+^ dendritic cells within the tumor microenvironment are essential for effector CD8^+^ T cell recruitment and mobility. Strategies to promote CD103^+^ dendritic cell accumulation and activation within the tumor microenvironment could be essential for expanding efficacy to checkpoint blockade immunotherapy.

### P411 Inhibition of matrix metalloproteinases by cysteamine regulates tumor invasion and metastasis in mouse models of human ovarian cancer

#### Akiko Suzuki, Pamela Leland, Bharat H Joshi, Raj K Puri

##### Center for Biologics Evaluation and Research, Food and Drug Administration, Silver Spring, MD, USA

###### **Correspondence:** Akiko Suzuki (akiko.suzuki@fda.hhs.gov)

**Background**

Previously, we have reported that cysteamine, an anti-oxidant aminothiol, inhibited the tumor invasion *in vitro* and metastasis of pancreatic cancer *in vivo* in mouse models of human pancreatic cancer. We have also shown that subcutaneous cysteamine treatment improves the survival of mice bearing orthotopically transplanted pancreatic tumors. Herein, we examined whether cysteamine can inhibit invasion and metastasis of other human cancers.

**Methods**

We examined cysteamine’s anti-invasion effects in 6 human ovarian cancer cell lines by a matrigel invasion assay. To study the mechanism of action, we evaluated the cytotoxic activity of cysteamine *in vitro* and measured the effect of cysteamine on matrix metalloproteinases (MMP), which are known to be involved in metastasis. We examined the anti-metastatic effect of cysteamine in two orthotopic murine models of human ovarian cancer by monitoring peritoneal metastases. A2780 and IGROV-1 ovarian cancer cells were surgically implanted orthotopically in the ovary followed by monitoring tumor growth and peritoneal metastasis using a high-resolution ultrasound system. Four days after tumor implantation, increasing doses of cysteamine (0, 100, or 250 mg/kg/day) were subcutaneously injected twice a day until the end of the experiment.

**Results**

We observed that cysteamine inhibited cell invasion *in vitro* in a concentration-dependent manner. Similar to invasion, MMP activity was inhibited by cysteamine in a concentration-dependent manner. We next examined the anti-metastatic effect of cysteamine on peritoneal metastasis in two orthotopic murine models of human ovarian cancer. Cysteamine significantly decreased the number of metastases and total weight of metastatic lesions in these mice (p < 0.01 in A2780 and p < 0.05 in IGROV-1 xenografts compared to untreated control mice), while the primary tumor volumes and weights were not changed. We did not observe any symptoms of general toxicity (such as general appearance, body weight, or mortality) in animals treated at the highest dose of cysteamine. Similar to the *in vitro* results, MMP activity was significantly decreased in primary tumors in both A2780 & IGROV-1 xenograft models treated with cysteamine (p < 0.05). Tumor growth was also monitored weekly by ultrasound volumetric analyses. We observed that tumor volumes measured by ultrasound imaging correlated positively with the *ex vivo* tumor weight measurements following dissection of the primary tumor at the end of experiment (Spearman r = 0.7870 in A2780 and 0.9800 in IGROV-1 xenografts).

**Conclusions**

Based on these results, we conclude that cysteamine may be a useful therapeutic option for ovarian cancer as a mono- or combination-therapy with other anti-cancer agents such as cancer vaccines and immunotherapy.

### P412 Validation of FGFR3 activation in non-T cell-inflamed bladder cancer

#### Randy F. Sweis^1^, Riyue Bao^1^, Jason Luke^1^, Thomas F Gajewski^2^

##### ^1^University of Chicago, Chicago, IL, USA; ^2^University of Chicago Medical Center, Chicago, IL, USA

###### **Correspondence:** Randy F. Sweis (rsweis@uchicago.edu)

**Background**

Immune checkpoint therapy recently led to the first regulatory approval in metastatic bladder cancer in well over a decade. Despite remarkable responses observed in some patients, the objective response rate remains 15 %. We recently reported a characterization of bladder cancers lacking a T cell-inflamed tumor microenvironment, which has been associated with lack of response to immune checkpoint therapy. We observed that FGFR3 activating mutations were exclusively present in non-T cell-inflamed tumors. In order to further substantiate our findings, we analyzed an independent cohort of non-T cell inflamed bladder cancers.

**Methods**

Ninety-three samples were analyzed from a validation data set (GSE 31684) with gene expression profiling. Affymetrix microarray files were downloaded from the GEO database and preprocessed to generate normalized gene expression values for 20,390 genes. Consensus clustering of samples was conducted using 725 genes from our prior TCGA analysis, and repeated using a refined 160 gene set developed across multiple cancer types by Methods previously described. Immune subtypes were called based on gene expression. Differentially expressed genes were filtered by FDR-adjusted P-value < 0.01 and fold change ≥ 1.5.

**Results**

We first evaluated the performance of our refined gene expression signature, which has recently been adapted for application across all solid tumor types. Comparing previous and current gene sets derived from our 13 gene T cell-inflamed signature, we found our classification to be robust with 89 % (83/93) concordance in immune subtype calling. We noted a higher proportion of samples were non-T cell-inflamed compared with bladder cancers in TCGA (48 % vs. 33 %). We confirmed a strong inverse correlation between expression of CD8A and FGFR3 (P < 0.0001). Furthermore, FGFR3 overexpressing tumors by median dichotomization had significantly lower expression of PD-L1, IDO1, LAG3, and TIM3 (all P ≤ 0.01). The association between FGFR3 and FOXP3 was not significant (P = 0.41). CD8A was concordantly expressed with PD-L1, IDO1, LAG3, FOXP3, and TIM3 (P < 0.0001), consistent with data from TCGA. Finally, differentially expressed genes were compiled and enriched pathways were identified by Ingenuity Pathway Analysis. In non-T cell-inflamed tumors from this data set, activation of β-catenin, PPARG and SMARCA4 pathways was observed (P < 0.0001).

**Conclusions**

In summary, we confirm the fidelity of our gene expression classification of non-T cell-inflamed bladder cancers across different gene expression platforms and data sets. In addition to activating mutations, FGFR3 overexpression occurs preferentially in the non-T cell-inflamed immune subtype lacking PD-L1 expression.

### P413 Reprogramming of the ovarian cancer microenvironment by poly-I:C and selective TLR3 ligand, rintatolimod

#### Marie-Nicole Theodoraki^1^, Frances-Mary Mogundo^2^, Robert P Edwards^2^, Pawel Kalinski^3^

##### ^1^University of Pittsburgh, Pittsburgh, PA, USA; ^2^Magee-Womens Research Institute, Ovarian Cancer Center of Excellence, and Peritoneal/Ovarian Cancer Specialty Care Center, Hillman Cancer Center, University of Pittsburgh, Pittsburgh, PA, USA; ^3^Department of Surgery; University of Pittsburgh Cancer Institute; Department of Infectious Diseases and Microbiology, University of Pittsburgh, Pittsburgh, PA, USA

###### **Correspondence:** Marie-Nicole Theodoraki (marie.nicole.theodorakis@gmail.com)

**Background**

Despite aggressive surgery and chemotherapy, the 5-year survival rate for patients with ovarian cancer is very low, 37 % for stage III and 25 % for stage IV [1]. Previously, we have demonstrated a high level of synergy among TLR-3 ligands, IFNa and COX-2 blockers in inducing chemokines, which attract the desirable cytotoxic T cells, Th1-cells and NK cells in colorectal cancer microenvironments and limited local production of CCL22, the chemokine implicated in the accumulation of T regulatory cells [2]. In this study, we investigated an optimized combinatorial adjuvant to reprogram the tumor microenvironment (TME) of ovarian cancer.

**Methods**

Ovarian cancer specimens were divided into 4-mm biopsies and cultured for 24 hours in the presence of IFNa, indomethacin (COX-1/2 inhibitor) or/and one of two synthetic TLR3 ligands Poly-I:C (non-selective activator of TLR3) rintatolimod (Ampligen), or their combinations. Biopsies were harvested for mRNA as well as for confocal microscopy and culture supernatants were analyzed for CCL5, CXCL10 and CCL22 concentration. Alternatively, established ovarian cancer cell lines (PE01 and PE04) or monocyte-derived macrophages were used in analogous experiments. Chemotaxis assays were performed using pre-activated CD8+ T cells and supernatants from the differentially treated ovarian cancer specimens.

**Results**

We observed that the combination of TLR-3 ligands, IFNa and COX1/2 blockers selectively induce the desirable chemokines CCL5 and CXCL10 and suppressed CCL22 in ovarian cancer with similar results in the macrophages and ovarian cancer cell lines. Unexpectedly, IFNa + poly-I:C but not IFNa + rintatolimod, also promoted the expression of the MDSC attractant CXCL12, which was reversed through addition of COX-2 inhibitors. Furthermore, the induction of CCL5 was enhanced by the addition of COX1/2 inhibitor. Compared to poly-I:C, the positive effects of the selective TLR-3 ligand, rintatolimod, were not dependent on the addition of indomethacin.

**Conclusions**

We demonstrate the feasibility of selective modulation of the ovarian TME, using combinations of different clinical reagents, in order to improve the local balance between tumor-infiltrating T effector and T regulatory cells. We show for the first time, that poly-I:C induces undesirable COX2-dependent suppressive factors, which can be eliminated through addition of the COX-1/2 inhibitor, indomethacin, or through usage of the selective TLR-3 ligand, rintatolimod.

**References**

1. Holschneider CH, Berek JS: **Ovarian cancer: epidemiology, biology, and prognostic factors**. *Semin Surg Oncol* 2000, **19**:3–10.

2. Muthuswamy R, Berk E, Junecko BF *et al.*: **NF-kappaB hyperactivation in tumor tissues allows tumor-selective reprogramming of the chemokine microenvironment to enhance the recruitment of cytolytic T effector cells**. *Cancer Res* 2012, **72**:3735–3743.

### P414 TLR9 expression in prostate cancer cells results in leukemia inhibitory factor (LIF)-mediated accumulation of pSTAT3+ myeloid-derived suppressor cells

#### Haejung Won, Dayson Moreira, Chan Gao, Xingli Zhao, Priyanka Duttagupta, Jeremy Jones, Massimo D’Apuzzo, Sumanta Pal, Marcin Kortylewski

##### City of Hope, Duarte, CA, USA

###### **Correspondence:** Haejung Won (hjwon@coh.org)

**Background**

Inflammation plays pivotal roles in carcinogenesis and formation of the immunosuppressive microenvironment of prostate tumors. However, molecular mechanisms of crosstalk between cancer cells and immune cells at the tumor site are only partly understood. Our recent study demonstrated that advanced, castration-resistant prostate cancer (CRPC) cells express innate immune receptor, Toll-Like Receptor 9 (TLR9). TLR9 signaling through NF-kB/RELA and STAT3 promoted tumor propagating potential and self-renewal of prostate cancer cells. Here, we evaluated the effect of cancer cell-intrinsic TLR9 signaling on the composition of tumor microenvironment.

**Methods**

For temporal control of TLR9 expression, we introduced tetracycline-inducible TLR9 expression system (Tet-On) into mouse Ras/Myc-driven (RM9) prostate cancer cells.

**Results**

The induction of TLR9 expression in RM9 tumors *in vivo* correlated with the accumulation of polymorphonuclear myeloid-derived suppressor cells (PMN-MDSCs; CD11b^+^Ly6C^INT^Ly6G^HI^). The PMN-MDSC in TLR9^HI^-tumors showed elevated levels of activated STAT3, a master regulator of immunosuppressive MDSC activities. Accordingly, tumor-associated PMN-MDSCs isolated from TLR9^HI^-, but not from TLR9^LO^- RM9 tumors, expressed high level of immunosuppressive mediators such as Arginase-1 and IL-10, and showed augmented suppressive effects on T cell proliferation. TLR9 expression in mouse and human (LNCaP and PC3) prostate cancer cells resulted in altered cytokine/chemokine secretion with significant upregulation of an IL-6 family cytokine, leukemia inhibitory factor (LIF). Our preliminary analysis confirmed elevated levels of LIF in plasma from prostate cancer patients compared to healthy subjects. On molecular level, TLR9 triggered direct binding of the NF-kB and STAT3 to the LIF promoter as verified by chromatin immunoprecipitation (ChIP) assays. The antibody-mediated neutralization of LIF significantly inhibited tumor growth *in vivo* with concomitant reduction in the accumulation of pSTAT3^+^ PMN-MDSCs. Finally, we used an oligonucleotide-based approach to inhibit STAT3 in TLR9 expressing RM9 tumor cells and in tumor-associated myeloid cells such as PMN-MDSCs. Local injection of CpG-STAT3 inhibitor reduced growth of RM9 prostate tumors at the treated and in the distant site suggesting generation of systemic antitumor immune responses.

**Conclusions**

Collectively, TLR9 signaling in prostate cancer cells induces tolerogenic tumor microenvironment through altering cytokine and chemokine and subsequent activation of STAT3 in tumor-infiltrated myeloid cells. In addition, the results suggest therapeutic potential of targeting TLR9+ prostate cancer cells and tumor-associated PMN-MDSC to eliminate both tumorigenic and tolerogenic effects of STAT3 signaling in the tumor microenvironment.

### P415 Immune modulation of the cancer microenvironment for enhancing cancer immunotherapy in ovarian clear cell carcinoma

#### Tomonori Yaguchi, Juri Sugiyama, Hiroshi Nishio, Taeko Hayakawa, Yutaka Kawakami

##### Division of Cellular Signaling, Institute for Advanced Medical Research, Keio University School of Medicine, Shinjuku-ku, Tokyo, Japan

###### **Correspondence:** Tomonori Yaguchi (beatless@rr.iij4u.or.jp)

**Background**

To enhance the anti-tumor effects of current immunotherapies including immune checkpoint blockade therapies, it is important to reverse the cancer-induced immunosuppression. Ovarian clear cell carcinoma (OCCC), the second most major subtype of ovarian cancer in Japan, produces high amounts of immunosuppressive cytokines such as IL-6 and IL-8, which were correlated with poor prognoses. A transcriptional factor HNF-1β preferentially activated in human OCCC contributes to various malignant phenotypes including chemoresistance and glucose metabolism. In this study, we have investigated roles of HNF-1β in the immunosuppressive activity of human OCCC.

**Methods**

We have evaluated the number of tumor-infiltrating T cells in human OCCC tissues. We have evaluated the contribution of HNF-1β activation in OCCC to the suppression of DC function through the production of IL-6 *in vitro* and *in vivo*. Finally, we have screened an existing drug library for agents which have an activity to suppress IL-6 production from OCCC and evaluated their effects *in vivo*.

**Results**

Tumor-infiltrating T cells in OCCC were significantly fewer than those in other types of cancers including serous ovarian cancers. HNF-1β knockdown and overexpression experiments revealed that HNF-1β induced IL-6 and IL-8 production via NF-κB and STAT3 activation in OCCC. Suppressive activities of human OCCC cell line culture supernatants on human monocyte-derived dendritic cells (DC) were reduced *in vitro* by knockdown of HNF-1β partly through decrease of IL-6. Knockdown of HNF-1β in a human OCCC cell line resulted in the restoration of T cell stimulatory activity of murine DC in nude mice implanted with human OCCC partly through decrease of human IL-6 *in vivo*. From the drug screening, various existing drugs having activity to inhibit STAT-3 or NF-κB were found to suppress IL-6 production from OCCC and to restore immuno-competence of cancer-bearing mice.

**Conclusions**

HNF-1β activation in human OCCC is a possible upstream event for induction of immunosuppression by IL-6 and IL-8 production via NF-κB and STAT3 activation and that combination of drugs targeting HNF-1β/STAT3/NF-kB pathway may enhance the efficacy of current immunotherapies for OCCC patients.

### P416 Targeting fibroblast activation protein in combination with radiation

#### Kayla McCarty, David Friedman, Benjamin Cottam, Pippa Newell, Michael Gough, Marka Crittenden, Kristina Young

##### Earle A. Chile Research Institute, Portland, OR, USA

###### **Correspondence:** Kristina Young (kristina.young@providence.org)

**Background**

Pancreatic ductal adenocarcinoma (PDAC) is characterized by a fibrotic stroma and poor immune infiltrate, in part driven by cancer-associated fibroblasts (CAFs). CAFs, which selectively express fibroblast activation protein (FAP), contribute to immune escape via sequestration of anti-tumor CD8+ T cells, upregulation of immune checkpoint ligand expression, and immunosuppressive cytokine production and polarization of tumor infiltrating immune cells.

**Methods**

We established syngeneic pancreatic tumors in immune competent C57BL/6 mice using Panc02 cells. From days 7-20, mice were treated with FAP inhibitor UAMC-1110 or control. Radiation (RT) was delivered exclusively to the tumor using a 1cm collimator on the Small Animal Radiation Research Platform, 10Gy x 3 daily fractions on day 14, 15, and 16. FAP knockout mice were challenged with Panc02 or Panc02-SIY cells and radiated 10Gy x 3 on days 14-16. Tumors were measured, and mice followed for survival. Using the same treatment groups above, tumors were harvested for flow cytometry and multiparameter immunofluorescence on day 14, 23, and 43. Splenocytes were harvested and pulsed with SIY peptide and tested for intracellular cytokine production on day 14 and 23. Tumor infiltrating macrophages were FACs sorted and western blot was performed to determine M1 vs M2 polarization. Tumor cytokines were assessed using cytokine bead assay.

**Results**

UAMC-1110 alone had no effect on tumor growth or survival. RT caused a transient growth delay, resulting in a survival advantage. Combination treatment with radiation and UAMC-1110 resulted in two temporally distinct growth delays: an initial tumor growth delay significant over radiation alone at day 22, and a second late response at day 43; but did not translate to a survival advantage over RT. At day 14, UAMC-1110 treatment resulted in fewer macrophages. At day 23, RT increased CD11b+ tumor infiltrates, MDSCs, and CD3+ infiltrates. Tumor immune infiltrates were equivalent at day 43. Radiation and combination treatment with UAMC-1110 increased collagen deposition. FAP knockout mice had equivalent response to radiation compared to wildtype control. FAP KO tumors had fewer macrophages and MDSCs, and more antigen-specific T cells following radiation, but not prior to radiation.

**Conclusions**

We tested a novel specific FAP inhibitor with radiation in a murine PDAC model. We found FAP inhibition favorably altered tumor immune infiltrate, and increase antigen-specific T cells following radiation, but was insufficient to result in a survival advantage. While FAP may be selectively expressed by CAFs, inhibition of its function is not seem sufficient to influence in vivo radiosensitivity. We propose a more comprehensive targeting of CAFs may be required to influence radiation response.

**Acknowledgements**

KHY received an ASCO Young Investigator Award and an RSNA Research Seed Grant which funded this work.

### P417 Arginase 2 blockage and reduction of PD-L1 expression in renal cell carcinoma

#### Sufyan Jarushi^1^, Brashonda Ross^2^, Sarah Normand^3^, Matthew Nguyen^4^, Arnold Zea^1^

##### ^1^LSUHSC-NO, New Orleans, LA, USA; ^2^Dillard University-NO, New Orleans, LA, USA; ^3^Rhodes College, New Orleans, LA, USA; ^4^Tulane University, New Orleans, LA, USA

###### **Correspondence:** Arnold Zea (azea@lsuhsc.edu)

**Background**

Tumors may evade the immune system, in part by exploiting interactions between T cells and the tumor. Renal cell carcinoma (RCC) is a poorly responsive tumor to conventional cytotoxic chemotherapies, although it is sensitive to IL-2, which still remains as the treatment of choice. The FDA has approved novel PD-1 immunotherapies, which have shown survival benefits for patients with metastatic RCC. However, preclinical studies and early phase clinical trials suggest that resistance mechanisms exploited by tumors may limit the effectiveness of these therapies. We believe that the heterogeneity of RCC tumors can contribute to the poor responses in RCC. In this work, we are studying the role the enzyme arginase 2 (ARG2) plays in the expression of PD-L1 in RCC tumors. Different levels of ARG2 expression in RCC contributes proportionally to the generation of T cell dysfunction. The understanding of this mechanism will be crucial for probing human immune responses and tumor biology in order to understand what distinguishes responder vs. non-responder patients. Therefore, our hypothesis is that the expression of PD-L1 in RCC tumors is regulated by ARG2 and blocking this enzyme will reduce its expression, resulting in improved antitumor activity.

**Methods**

Three murine RCC cell lines, CL-2 (low-ARG2), CL-19 (high-ARG2), and Renca (median-ARG2) were cultured at different time points: 1, 3, 6, and 24 h in the presence or absence of IFNγ and ARG2 inhibitor BEC. Expression of PD-L1 was assessed by flow cytometry, tumor cell survival by MTT assay, ARG activity by enzymatic assays and supernatants for L-arginine by HPLC.

**Results**

PD-L1 expression was similar between CL-2 and CL-19 cell lines (17-20 %) that significantly increased up to 95 % after 6 h of IFNγ stimulation (2.5 U/ML). Interestingly, we observed a constitutive high expression of PD-L1 in Renca cells (85-90 %) in non-stimulated cells. When cells were treated with ARG2 inhibitor, PD-L1 expression decreased 5 fold in CL-2 and CL19 cells but had not effect on Renca cells. High concentrations of IFNγ (10U/ML) had cytotoxic effects in CL-2 and CL-19 but not in Renca cells.

**Conclusions**

These results suggest that 1) RCC heterogeneity plays an important role in the expression of immune checkpoints and in pathways that can disable T cells. This finding can contribute to determine what patients may benefit or not from anti PD-1 therapy. 2) The role of IFNγ in this process is unclear and is currently being investigated.

